# Supplementary material for: Repeated LPS induces training and tolerance of microglial responses across brain regions
Source: J Neuroinflammation. 2024 Sep 20;21:233. doi: 10.1186/s12974-024-03198-1 (PMC11414187; doi:10.1186/s12974-024-03198-1)
Supplement: Supplementary file 1 — Supplementary Material 1 [file 12974_2024_3198_MOESM1_ESM.pdf]

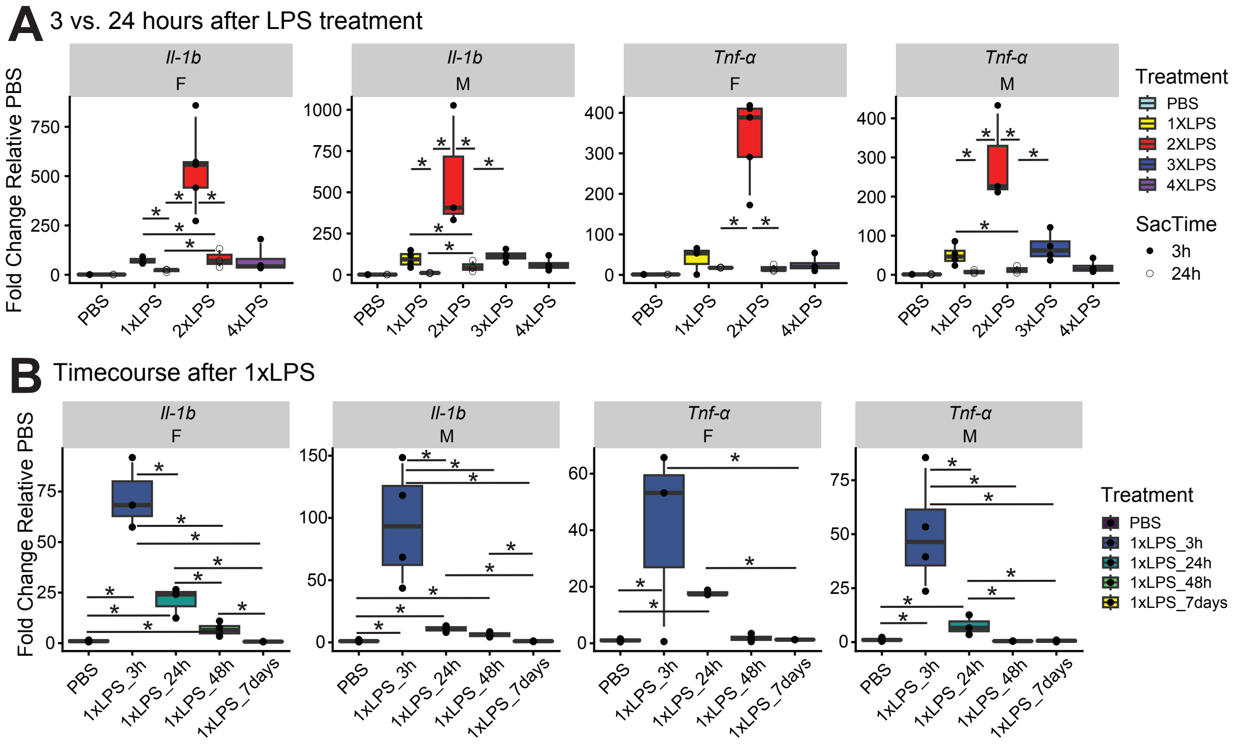

Supplementary Figure S1: **Temporal dynamics of cytokine expression after LPS treatment.** RT-qPCR fold change comparisons for pro-inflammatory cytokines *Il-1β and Tnf-α* (*$p<0.05$, BH-corrected) from frontal cortex tissue isolated from female and male mice (A) 3 and 24 hours after LPS treatments or (B) at different timepoints after 1xLPS.

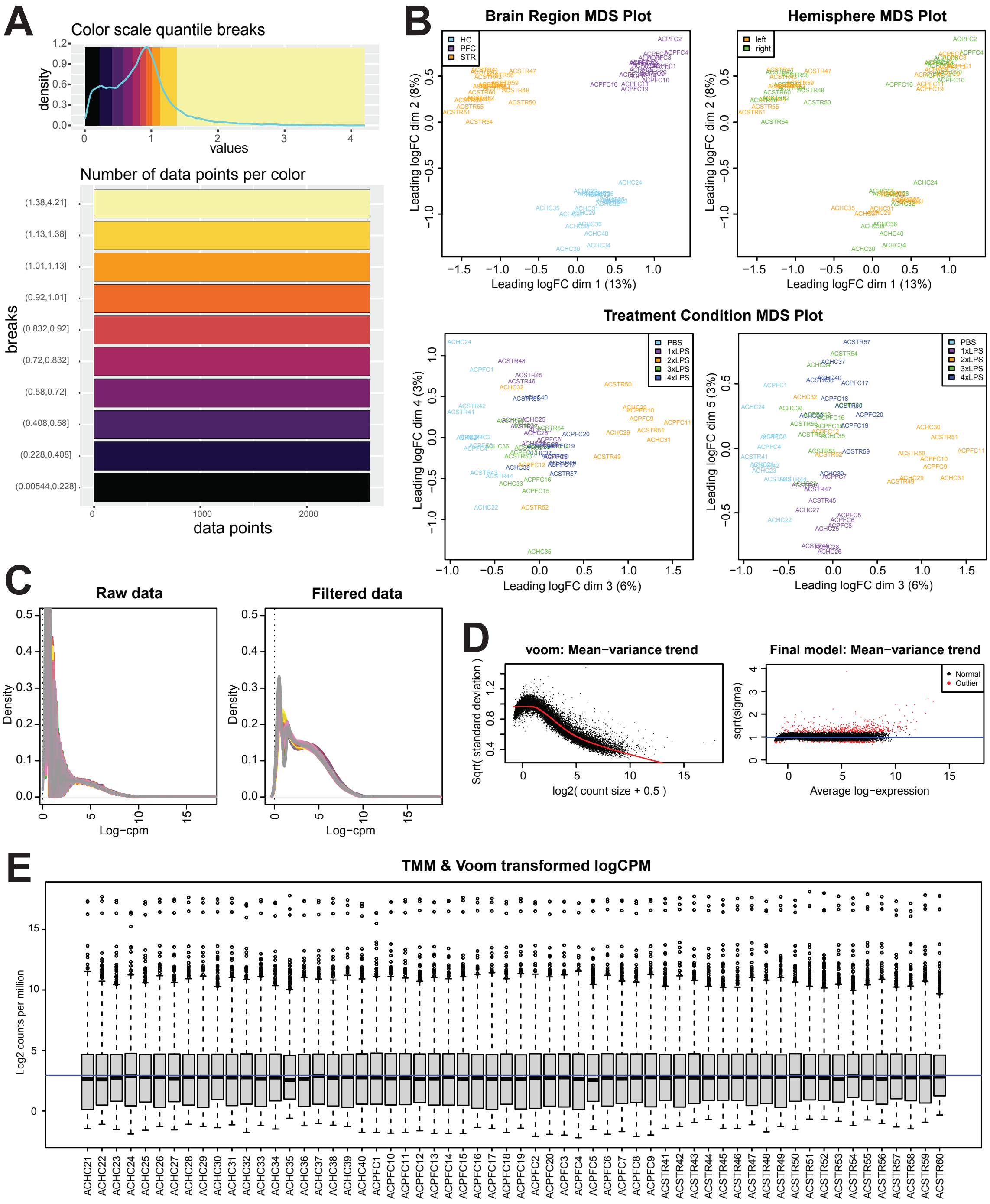

***Supplementary Figure S2:* Differential gene expression analysis using lima-voom.** (A) Reference for color scale for scaled gene expression in DEG heatmap in Figure 3. Counts per million (CPM) for each DEG was scaled within each brain region first, then scaled within genes across brain regions (rows) in the final heatmap (Fig. 3A). Breaks between each color of the heatmap scale were adjusted to fit the distribution of the scaled gene expression values such that 10% of the data was contained within each color break. (B) MDS plots colored by brain region and hemisphere along dimensions 1 and 2 and colored by treatment along dimensions 3, 4, and 5. (C) Distribution of log CPM for each sample before and after filtering. (D) Mean-variance trend of counts and final statistical model after limma-voom normalization. (E) Boxplots of Log2CPM values for each sample after TMM and limma-voom normalization.

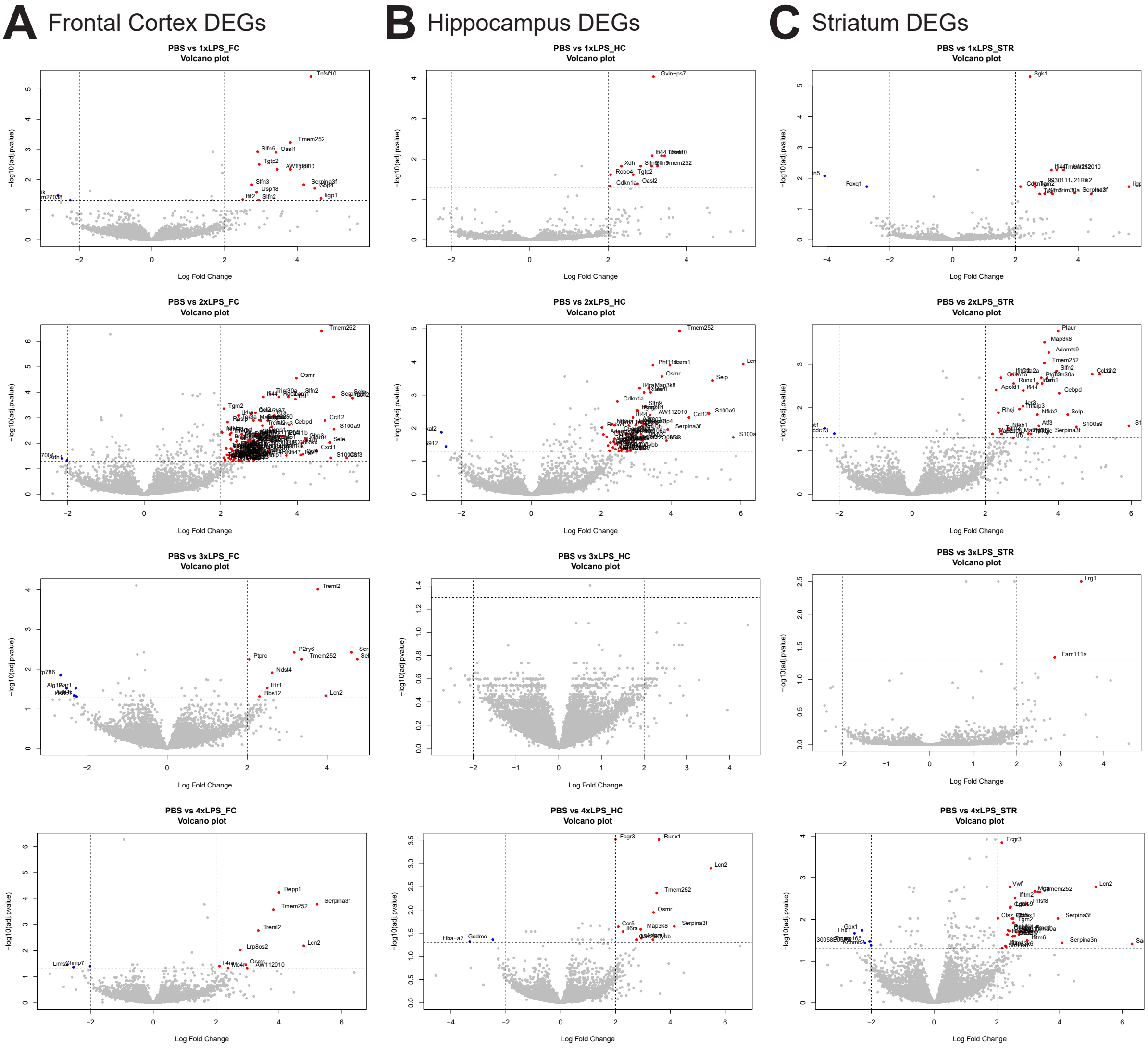

Supplementary Figure S3: **DEGs within each brain region for each LPS versus PBS comparison.** Volcano plots for Log Fold Change versus -log(corrected pvalue) for PBS versus each LPS treatment condition for (A) Frontal Cortex, (B) Hippocampus, and (C) Striatum. Genes with BH-corrected p-value < 0.05 and fold change>+/- 2 for each comparison are colored red (increased) or blue (decreased) and labeled with the gene name.

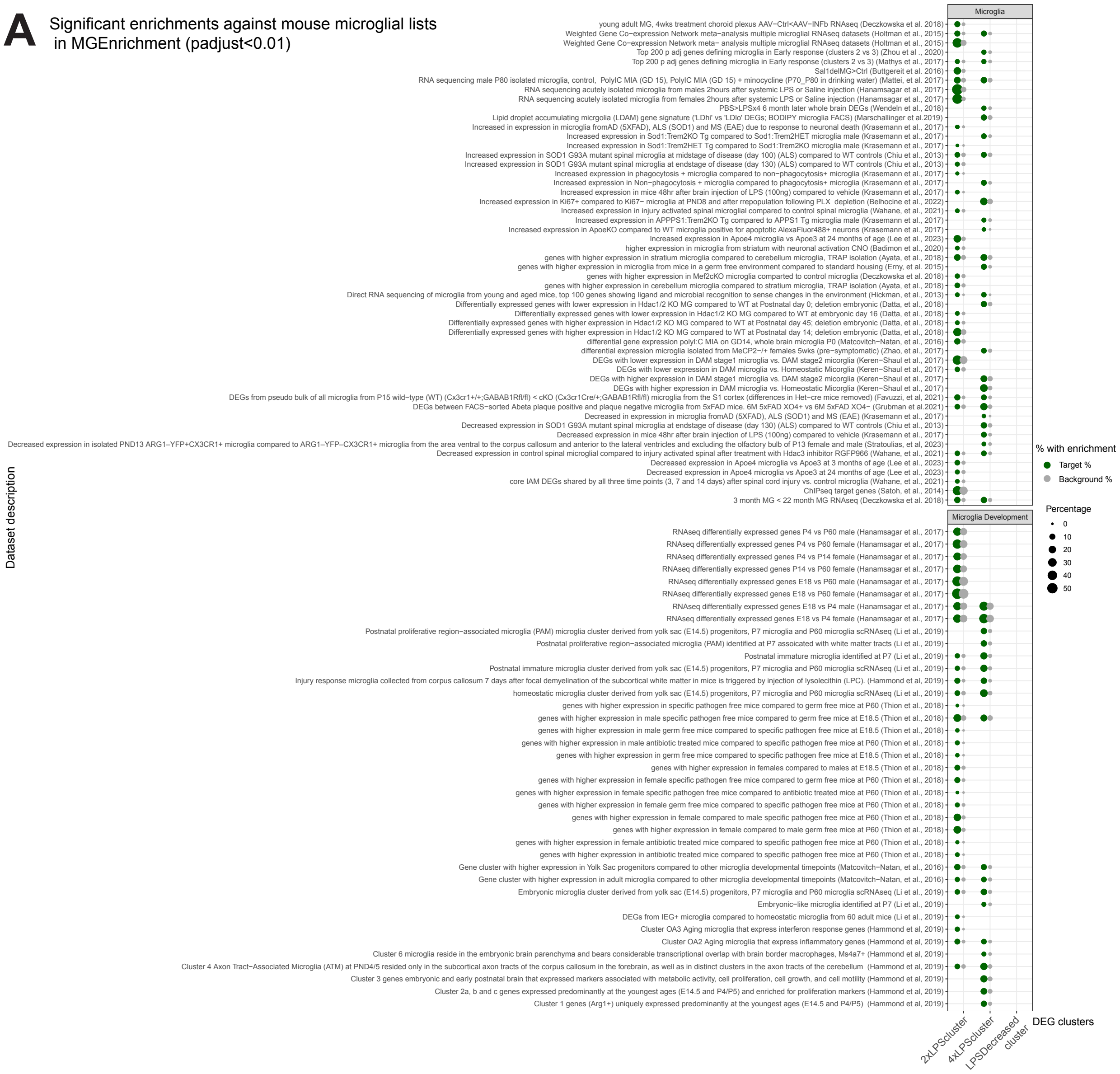

Supplementary Figure S4: **2xLPS-sensitive and 4xLPS-sensitive cluster DEGs are enriched for microglia-relevant genes.** (A) Significant enrichments against mouse microglial gene lists in MGEnrichment (36). Green circles denote a significant enrichment in the percent of DEGs for a given gene list compared to that of the background of all genes detected in the RNA-Seq experiment (grey circles). Only significant enrichments are shown (*p<0.01, BH-corrected).

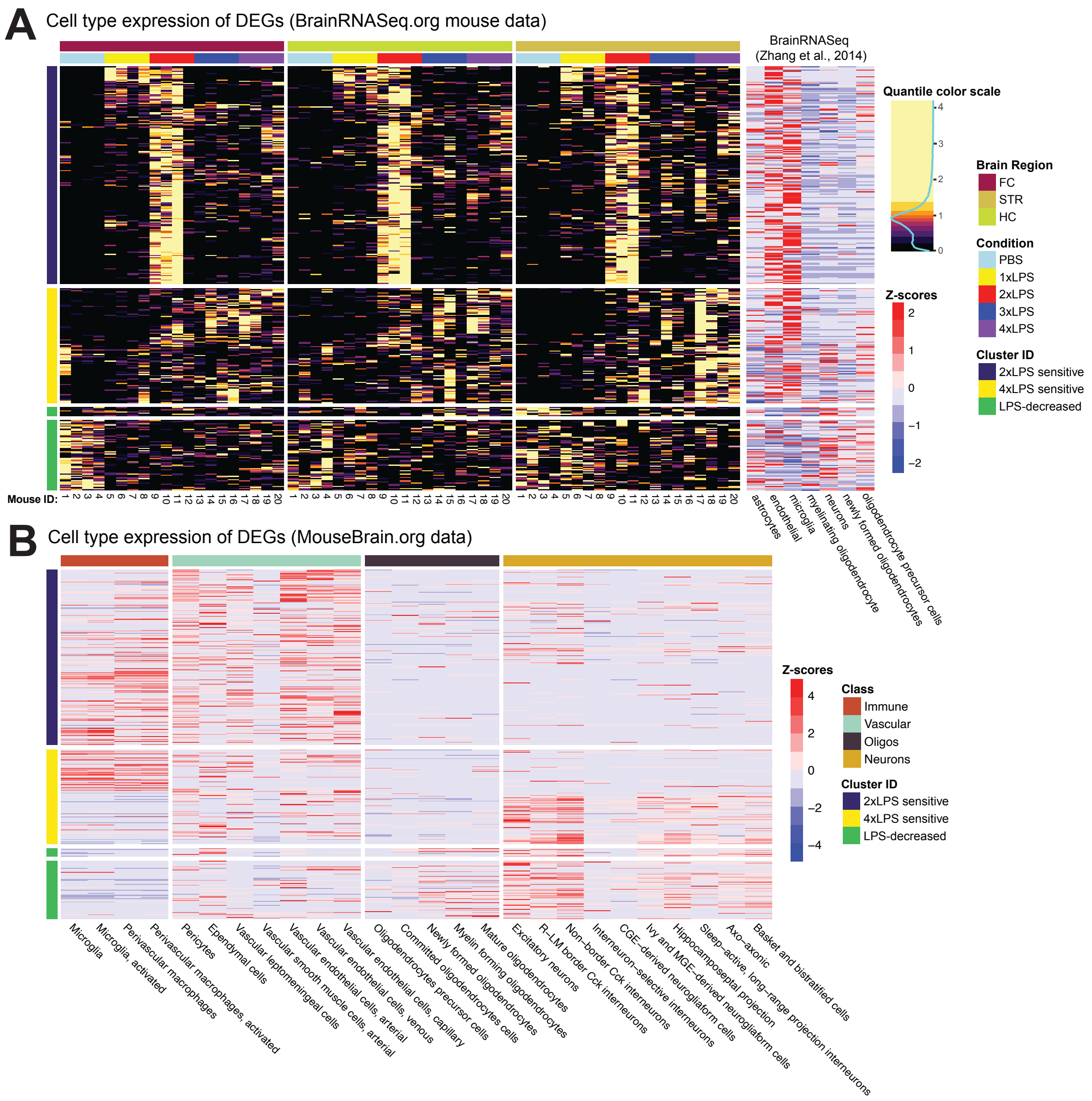

Supplementary Figure S5: **Cell type-specific expression of DEGs.** (A) Heatmap of scaled gene expression of LPS cluster DEGs from Figure 3 and their baseline expression values (FPKM scaled by row) across different brain cell types from BrainRNASeq.org mouse brain data (Zhang et al., 2014) (44). (B) Normalized gene expression values of LPS cluster DEGs in (A), across brain cell types from MouseBrain.org dataset (Zeisel et al., 2018) (45).

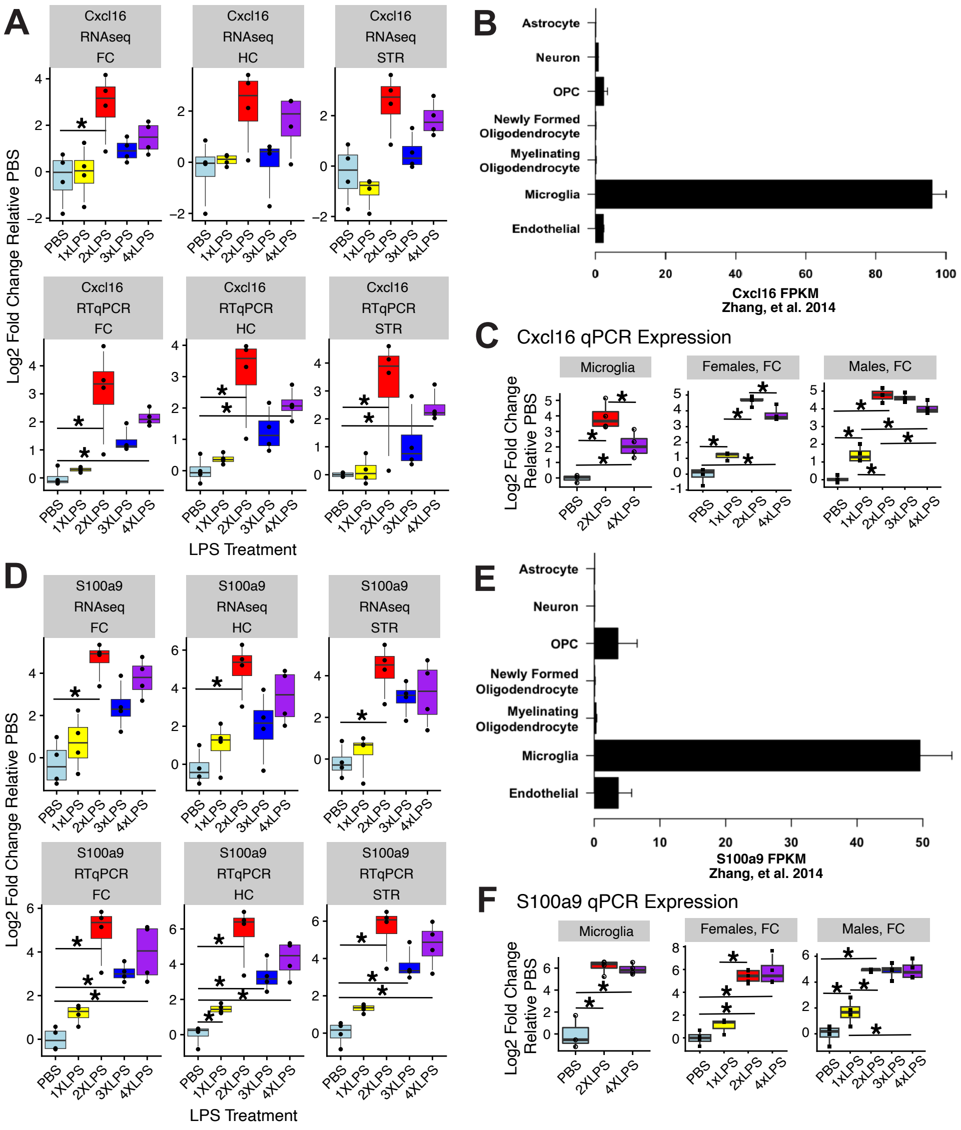

***Supplementary Figure S6:* Cxcl16 and S100a9 expression in microglia is dynamic in response to repeated LPS**. (A) Log2 fold change relative to PBS controls for each LPS treatment condition for *Cxcl16* in RNAseq (top row) compared to RT-qPCR expression (bottom row) from the same mice. Patterns of expression were conserved across the two techniques for each brain region (Frontal Cortex, FC; Hippocampus, HC; and Striatum, STR). For RNAseq *p<0.05, BH-corrected for multiple comparison across all genes in the RNAseq. For RT-qPCR *p<0.05, corrected for multiple comparisons within the gene of interest. (B) Expression of *Cxcl16* within different isolated brain cell types from Zhang et al., 2014 (https://web.stanford.edu/group/barres_lab/brain_rnaseq.html). (C) Expression of *Cxcl16* by RT-qPCR in isolated cortical microglia and frontal cortex tissue following LPS treatments. *p<0.05, BH-corrected for multiple comparisons for 1-4xLPS treatment versus PBS. (D) Log2 fold change relative to PBS controls for each LPS treatment condition for *S100a9* in RNAseq (top row) compared to RT-qPCR expression (bottom row) from the same mice. Patterns of expression were conserved across the two techniques for each brain region (Frontal Cortex, FC; Hippocampus, HC; and Striatum, STR). For RNAseq *p<0.05, BH-corrected for multiple comparison across all genes in the RNAseq. For RT-qPCR *p<0.05, corrected for multiple comparisons within the gene of interest. (E) Expression of *S100a9* in isolated brain cell types from Zhang et al., 2014. (F) Expression of *S100a9* by RT-qPCR in isolated cortical microglia and frontal cortex tissue following LPS treatments. *p<0.05, BH-corrected for multiple comparisons for 1-4xLPS treatment versus PBS.

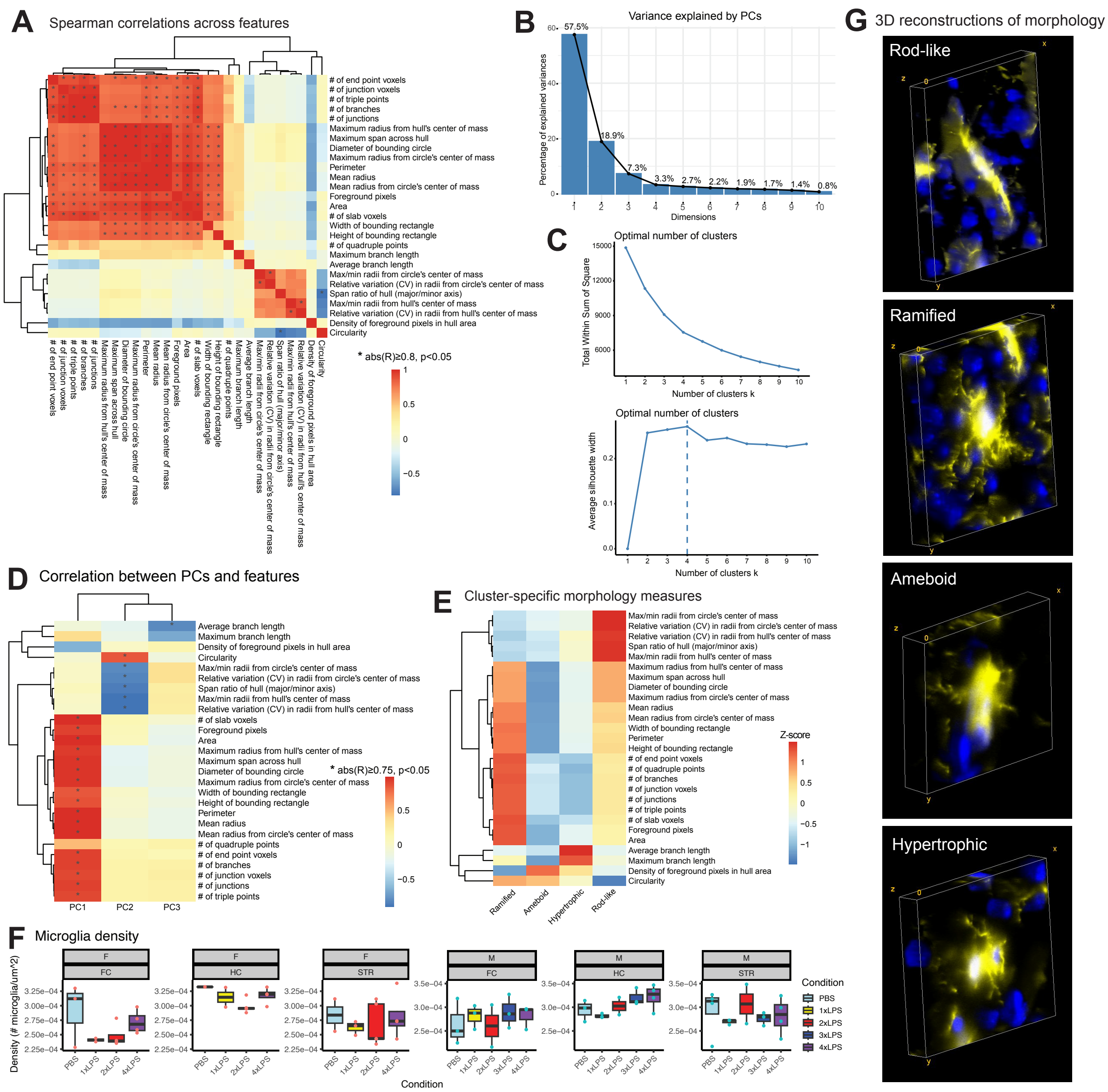

Supplementary Figure S7: **Analysis of morphology measures and clusters using MicrogliaMorphology and MicrogliaMorphologyR.** (A) Spearman's correlation matrix of 27 features measured by MicrogliaMorphology. (*abs(R)≥0.8, p<0.05) (B) Variance in dataset described by first 10 principal components. (C) Optimal k-means clustering parameters determined using within sum of squares and gap statistic techniques. (D) Spearman's correlation of morphology measures to first 3 PCs after dimensionality reduction. (*abs(R)≥0.75, p<0.05) (E) Average values for all 27 morphology features, centered and scaled across clusters. (F) Quantification of microglia density with repeated LPS across brain regions within sexes. No significant differences between groups. (*p<0.05, BH-corrected). (G) 3D reconstructions of individual microglia labeled as rod-like, ramified, ameboid, and hypertrophic after clustering.

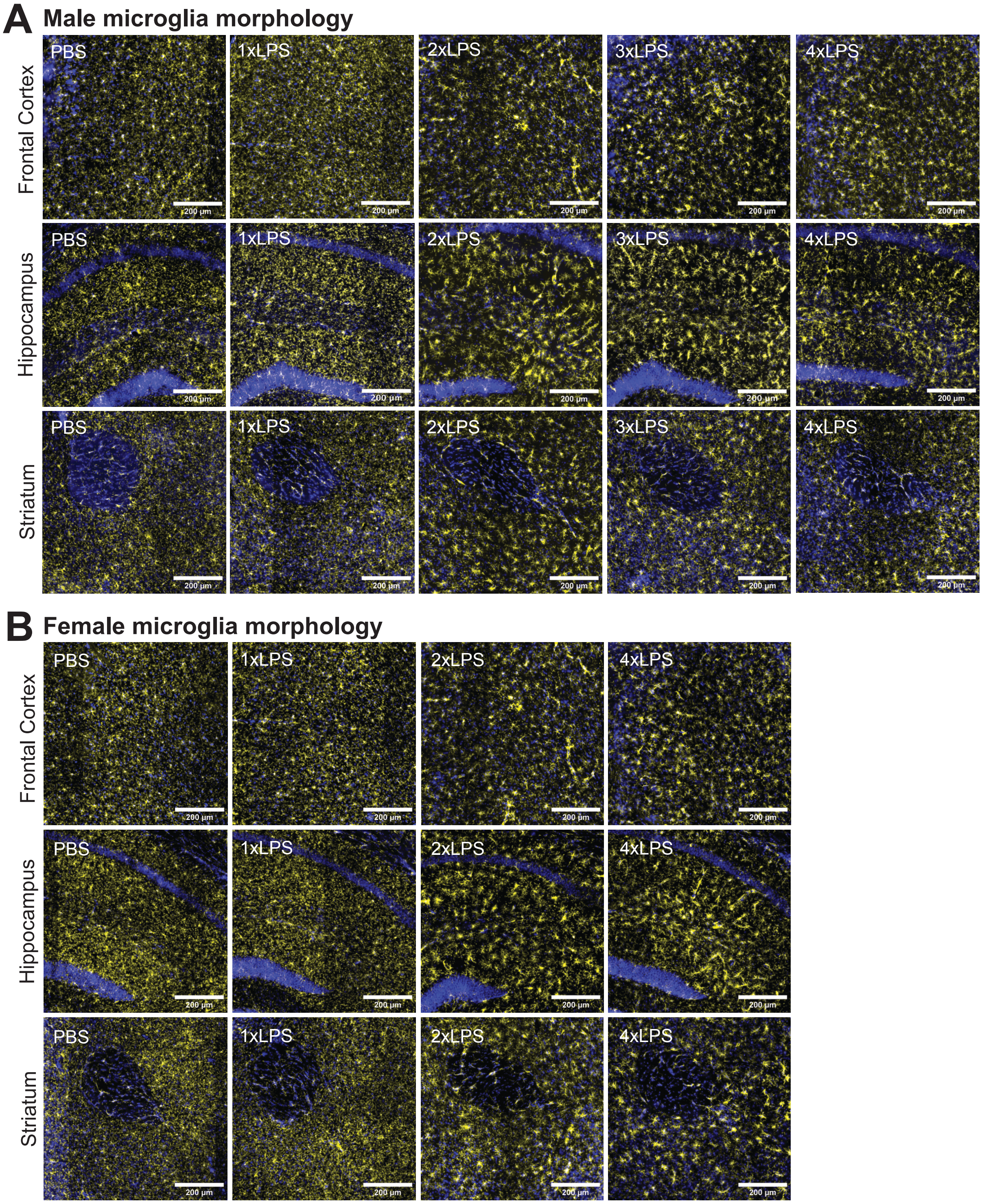

Supplementary Figure S8: **Microglia morphology visualized across brain regions, LPS treatments, and sexes.** Example immunofluorescent images of frontal cortex, hippocampus, and striatum microglial cells stained with P2ry12 (yellow) across all LPS conditions for males (A) and females (B). Nuclei are stained with DAPI (blue). Scale bars are 200um. Hippocampal images in (A) are an extension of Figure 5B.

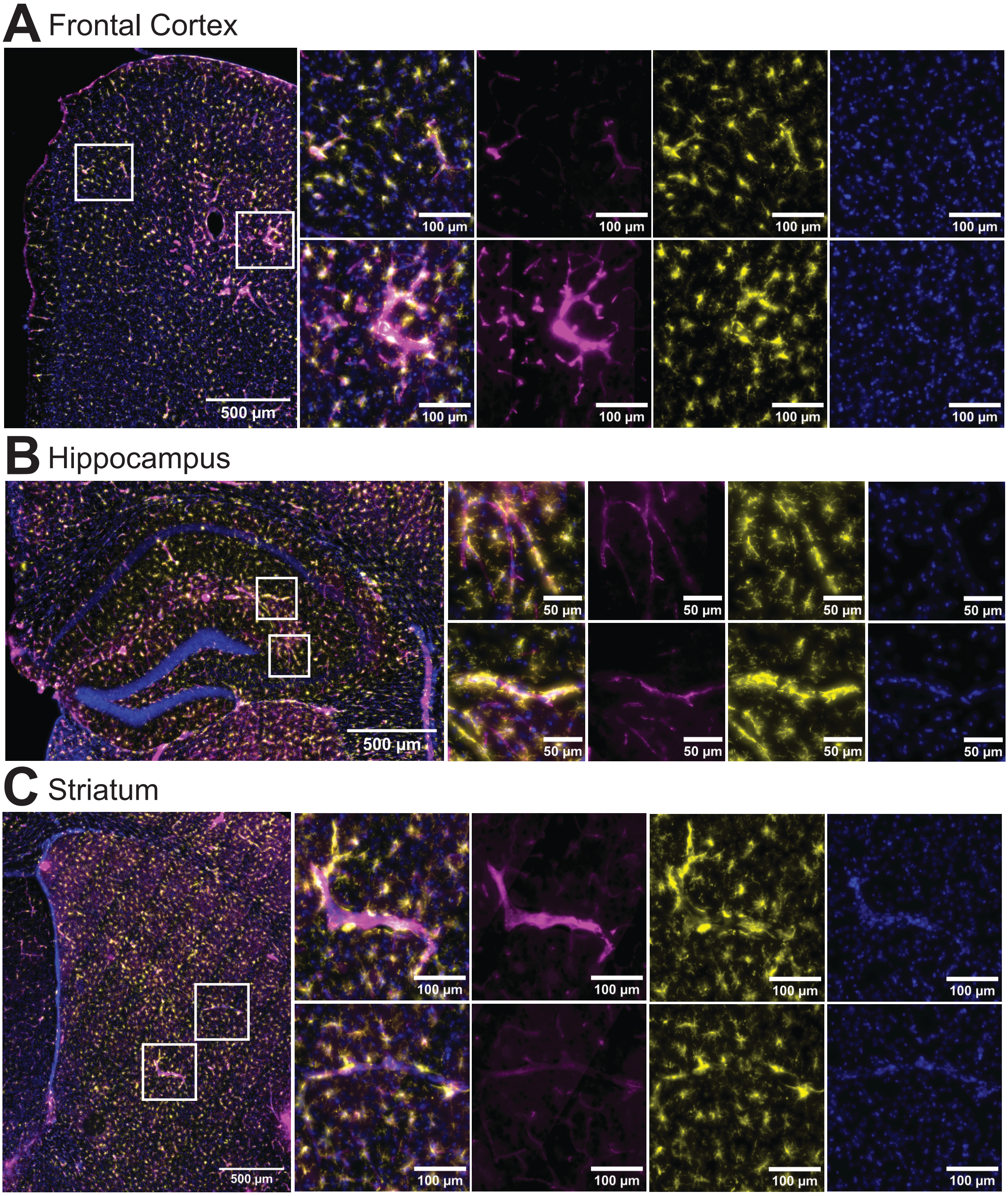

Supplementary Figure S9: **Microglia align end-to-end and wrap around blood vessels.** Example immunofluorescent images from 4xLPS animals of frontal cortex (A), hippocampus (B), and striatum (C) microglial cells stained with Iba1 (yellow) and blood vessels stained with PECAM-1 (magenta). Nuclei are stained with DAPI (blue). Scale bars in (A) are 500um and 100um, (B) are 500um and 50um, and (C) are 500um and 100um.
