# Supplementary material for: Repeated LPS induces training and tolerance of microglial responses across brain regions
Source: J Neuroinflammation. 2024 Sep 20;21:233. doi: 10.1186/s12974-024-03198-1 (PMC11414187; doi:10.1186/s12974-024-03198-1)
Supplement: Supplementary file 7 — Supplementary Material 7. File S1: Homer software output for transcription factor motif analysis of 2xLPS-sensitive cluster gene promoters. Related to Figures 4A and C. [file 12974_2024_3198_MOESM7_ESM.zip › 2xLPS_cluster_genes_output/geneOntology.html]

Gene Ontology Results

# Gene Ontology Enrichment Results

Homer *de novo* Motif Enrichment Results  
Known Motif Enrichment Results  

#### Text file version of complete results (i.e. open with Excel) - biological process: Functional groupings of proteins (Gene Ontology) - molecular function: Mechanistic actions of proteins (Gene Ontology) - cellular component: Protein localization (Gene Ontology) - chromosome location: Genes with similar chromosome localization (NCBI Gene) - KEGG pathways: Groups of proteins in the same pathways (KEGG) - protein interactions: "Proteins interacting with a common protein (BIND, EcoCyc, HPRD)" (NCBI Gene) - interpro domains: Proteins with similar domains and features (Interpro) - pfam domains: Proteins with similar domains and features (Pfam) - smart domains: Proteins with similar domains and features (SMART) - gene3d domains: Proteins with similar domains and features (Gene3D) - prosite domains: Proteins with similar domains and features (Prosite) - prints domains: Proteins with similar domains and features (PRINTS) - MSigDB lists: "Genes sets for pathways, factor/miRNA target predictions, expression patterns, etc." (MSigDB) - BIOCYC pathways: Groups of proteins in the same pathways (BIOCYC) - COSMIC cancer mutations: Genes mutated in similar cancers (COSMIC) - GWAS genes: Genes mutated in similar diseases (GWAS Catalog) - Lipid Maps pathways: Groups of proteins in the same lipid pathways (Lipid Maps/Biosystems) - Pathway Interaction DB: Groups of proteins in the same pathways (Pathway Interaction Database) - REACTOME pathways: Groups of proteins in the same pathways (REACTOME) - SMPDB pathways: Groups of proteins in the same pathways (SMPDB) - WikiPathways: Groups of proteins in the same pathways (Wikipathways) Enriched Categories | | | | | | | | | | | | --- | --- | --- | --- | --- | --- | --- | --- | --- | --- | | P-value | ln(P) | Term | GO Tree | GO ID | # of Genes in Term | # of Target Genes in Term | # of Total Genes | # of Target Genes | Common Genes | | 3.985e-49 | -111.44 | ALTEMEIER\_RESPONSE\_TO\_LPS\_WITH\_MECHANICAL\_VENTILATION | MSigDB lists | ALTEMEIER\_RESPONSE\_TO\_LPS\_WITH\_MECHANICAL\_VENTILATION | 104 | 41 | 12187 | 179 | Irgm2,C5ar1,Gem,Irf7,Slfn4,Nfkbia,Casp4,Plek,Csf2rb,Plaur,Socs3,Timp1,Il1rn,Atf3,Cxcl1,Bcl3,Osmr,Cmpk2,Gbp3,Ifit2,Map3k8,Maff,Ccl4,Gpr84,Usp18,Cybb,Hcar2,Ccl12,Tnfaip3,Il6,Selp,Isg15,Cd14,Slfn3,Thbs1,Nfkbiz,Gadd45g,Il1a,Akap12,Ms4a6d,Cebpd | | 6.593e-49 | -110.94 | response to biotic stimulus | biological process | GO:0009607 | 738 | 82 | 13711 | 214 | Gbp6,Wfdc21,Irgm2,AA467197,Bcl3,Usp18,S100a9,Cxcl16,Irf2,Ccl12,S100a8,Tnip3,Ifitm2,Gbp3,Ifi204,Lcn2,Adamts9,Tnfaip8,Irak3,Il1rn,Ccl2,Cxcl9,Tnfaip3,Il17ra,Trim56,Rtp4,Irgm1,Nfkb1,Slfn4,Gbp4,Lgals9,Mx1,Iigp1,Ifitm3,Oasl1,Cybb,Ifit2,Noct,Gbp7,Ifit3b,Serpina3f,Mt2,Hp,Hspa5,C5ar1,Cxcl1,Trim30a,Cflar,Cd14,Ncf1,Zfp36,Saa1,Ifi44,Acod1,Tnf,Trim25,Resf1,Ier3,Ptgs2,Mx2,Ccl4,Herc6,Ifi211,Ifitm6,Stx11,Parp9,Sbno2,Isg15,Slfn2,Cmpk2,Tgtp1,Trib1,Parp14,Ccl7,Dtx3l,Irf7,Il6,Nfkbia,Oasl2,Il4ra,Slfn9,Casp4 | | 9.230e-49 | -110.60 | response to other organism | biological process | GO:0051707 | 718 | 81 | 13711 | 214 | Casp4,Il4ra,Oasl2,Slfn9,Nfkbia,Il6,Irf7,Dtx3l,Ccl7,Parp14,Trib1,Tgtp1,Slfn2,Cmpk2,Sbno2,Isg15,Parp9,Ifitm6,Stx11,Ifi211,Herc6,Ccl4,Mx2,Ptgs2,Ier3,Trim25,Resf1,Acod1,Tnf,Ifi44,Zfp36,Saa1,Ncf1,Cd14,Cflar,Trim30a,C5ar1,Cxcl1,Hp,Mt2,Serpina3f,Ifit3b,Gbp7,Noct,Ifit2,Cybb,Oasl1,Ifitm3,Iigp1,Mx1,Lgals9,Gbp4,Slfn4,Nfkb1,Irgm1,Rtp4,Trim56,Il17ra,Tnfaip3,Cxcl9,Ccl2,Il1rn,Irak3,Tnfaip8,Adamts9,Lcn2,Ifi204,Gbp3,Ifitm2,Tnip3,S100a8,Irf2,Ccl12,Cxcl16,Usp18,S100a9,Bcl3,AA467197,Irgm2,Wfdc21,Gbp6 | | 1.148e-48 | -110.39 | response to external biotic stimulus | biological process | GO:0043207 | 720 | 81 | 13711 | 214 | Ncf1,Cflar,Cd14,Trim30a,Acod1,Tnf,Ifi44,Saa1,Zfp36,Serpina3f,Ifit3b,Gbp7,Noct,Ifit2,Cxcl1,C5ar1,Mt2,Hp,Irf7,Dtx3l,Ccl7,Trib1,Parp14,Casp4,Slfn9,Oasl2,Il4ra,Nfkbia,Il6,Ifitm6,Stx11,Ifi211,Herc6,Ccl4,Mx2,Ptgs2,Ier3,Trim25,Resf1,Tgtp1,Slfn2,Cmpk2,Sbno2,Isg15,Parp9,Il1rn,Irak3,Adamts9,Tnfaip8,Lcn2,Ifi204,Tnfaip3,Cxcl9,Ccl2,S100a9,Usp18,Bcl3,AA467197,Irgm2,Wfdc21,Gbp6,Gbp3,Ifitm2,Tnip3,S100a8,Ccl12,Irf2,Cxcl16,Oasl1,Ifitm3,Iigp1,Mx1,Lgals9,Cybb,Irgm1,Rtp4,Trim56,Il17ra,Gbp4,Slfn4,Nfkb1 | | 2.572e-41 | -93.46 | HALLMARK\_INTERFERON\_GAMMA\_RESPONSE | MSigDB lists | HALLMARK\_INTERFERON\_GAMMA\_RESPONSE | 175 | 43 | 12187 | 179 | Nfkb1,Rnf213,Isg15,Herc6,Ifitm3,Selp,Tap1,Il6,Ccl12,Rtp4,Cmpk2,Gbp3,Ifi44,Ifit3b,Vcam1,Socs3,Ifitm2,Ptgs2,Csf2rb,Nfkbia,Xaf1,Parp12,Gbp4,Gbp6,Ptpn1,Irf2,Il4ra,Ifit2,Usp18,Ripk1,Tnfaip3,Arid5b,Cdkn1a,Samd9l,Znfx1,Trim25,Icam1,Irf9,Casp4,Parp14,Oasl1,Irf7,Tnfsf10 | | 8.508e-40 | -89.96 | immune system process | biological process | GO:0002376 | 1296 | 93 | 13711 | 214 | Ncf1,Sele,Trim30a,Pik3ap1,Acod1,Msn,Gbp7,Selp,C5ar1,Parp14,Ccl7,Hcar2,Nfkbia,Slfn9,Il4ra,Mx2,Herc6,Ifi211,Cebpd,Trim25,Runx1,Tiparp,Isg15,Csf3,Zbtb16,Il1rn,C3ar1,Retnlg,Lcn2,Cxcl9,Tnfaip3,Ccl2,S100a9,Vcam1,Ifitm2,Nfkbiz,Cxcl16,Tnip3,Iigp1,Oasl1,Lgals9,Gadd45g,Nfkb2,Fyb,Icam1,Rtp4,Irgm1,Map3k8,Cd14,Tnip1,Tnf,Ifi44,Ifit3b,Ifit2,Cxcl1,Hp,Prg4,Dtx3l,Irf7,Pik3r1,Il1a,Oasl2,Casp4,Il6,Rbm47,Ccl4,Spi1,Ifitm6,Stx11,Ifi209,Parp9,Sbno2,Tgtp1,Irak3,Ifi204,Tnfsf10,Irf9,Gbp6,Irgm2,Samd9l,Bcl3,Gbp3,Ccl12,Irf2,Tap1,S100a8,Ifitm3,Mx1,Cybb,Il17ra,Trim56,Gbp4,Nfkb1 | | 8.577e-40 | -89.95 | defense response | biological process | GO:0006952 | 731 | 73 | 13711 | 214 | Il1rn,C3ar1,Tnfaip8,Ifi47,Lcn2,Tnfaip3,Cxcl9,Ccl2,S100a9,Bcl3,Gbp6,Irgm2,Nfkbiz,Gbp3,Ifitm2,S100a8,Tap1,Cxcl16,Ccl12,Irf2,Thbs1,Ifitm3,Gm4841,Oasl1,Iigp1,Mx1,Icam1,Cybb,Irgm1,Rtp4,Trim56,Il17ra,Gbp4,F830016B08Rik,Ncf1,Sele,Cd14,Tnip1,Trim30a,Acod1,Tnf,Saa1,Nfe2l2,Ifit3b,Gbp7,Selp,Ifit2,C5ar1,Cxcl1,Hp,Dtx3l,Irf7,Parp14,Ccl7,Oasl2,Il4ra,Slfn9,Casp4,Il1a,Gm5431,Il6,Tgtp2,Ifitm6,Stx11,Mx2,Herc6,Ccl4,Ptgs2,Trim25,Timp1,Tgtp1,Parp9,Isg15 | | 2.651e-37 | -84.22 | GSE13485\_DAY3\_VS\_DAY7\_YF17D\_VACCINE\_PBMC\_DN | MSigDB lists | GSE13485\_DAY3\_VS\_DAY7\_YF17D\_VACCINE\_PBMC\_DN | 136 | 37 | 12187 | 179 | Phf11d,Ifi211,Dtx3l,Rtp4,Cmpk2,Usp18,Gpr84,Ccl4,Ifit2,Gadd45b,Ccl12,Slfn5,Isg15,Herc6,Rnf213,Ifitm3,Tap1,Parp9,Phf11b,Irf7,C3ar1,Tnfsf10,Xaf1,Parp14,Tnf,Oasl1,Parp12,Ifitm2,Trim25,Ifit3b,Ifi44,Ifi209,Znfx1,Ifi207,Samd9l,Il1rn,Ifi204 | | 3.843e-37 | -83.85 | response to cytokine | biological process | GO:0034097 | 603 | 65 | 13711 | 214 | Irgm2,Wfdc21,Gbp6,Ccl12,Cxcl16,Gbp3,Ifitm2,Ifi204,Il1rn,Arid5b,Irak3,Ifi47,Csf3,Ccl2,Cxcl9,Trim56,Il17ra,Irgm1,Nfkb1,F830016B08Rik,Gbp4,Birc3,Osmr,Gm4841,Ifitm3,Iigp1,Icam1,Gbp7,Csf2rb,Selp,Ifit2,Serpina3f,Nfe2l2,Cxcl1,Hspa5,Sele,Ifi207,Cd14,Ripk1,Zfp36,Acod1,Tnf,Ptgs2,Trim25,Ifitm6,Stx11,Ifi211,Xaf1,Spi1,Ccl4,Tgtp1,Isg15,Sbno2,Timp1,Ifi209,Fzd4,Socs3,Irf7,Ccl7,Il6,Gm5431,Tgtp2,Il4ra,Il1a,Nfkbia | | 1.321e-35 | -80.31 | response to external stimulus | biological process | GO:0009605 | 1456 | 93 | 13711 | 214 | Dtx3l,Irf7,Trib1,Oasl2,Casp4,Il6,Cdkn1a,Stx11,Ifitm6,Ccl4,Ptgs2,Ier3,Cmpk2,Tgtp1,Parp9,Sbno2,Cd14,Cflar,Fstl1,Ifi44,Tnf,Zfp36,Saa1,Ifit3b,Ifit2,Cxcl1,Rrp8,Mt2,Hp,Thbs1,Ifitm3,Mx1,Cybb,Trim56,Il17ra,Slfn4,Gbp4,Nfkb1,Irak3,Adamts9,Ifi204,Usp18,AA467197,Bcl3,Gbp6,Irgm2,Gbp3,S100a8,Ccl12,Irf2,Parp14,Ccl7,Slfn9,Il4ra,Nfkbia,Ifi211,Mx2,Herc6,Trim25,Resf1,Fam107a,Slfn2,Isg15,Ncf1,Trim30a,Acod1,Atf3,Nfe2l2,Serpina3f,Angptl4,Noct,Gbp7,C5ar1,Hspa5,Oasl1,Iigp1,Lgals9,Irgm1,Rtp4,Il1rn,C3ar1,Tnfaip8,Retnlg,Lcn2,Tnfaip3,Cxcl9,Ccl2,Vcam1,S100a9,Wfdc21,Ifitm2,Tnip3,Cxcl16 | | 4.114e-35 | -79.18 | response to stimulus | biological process | GO:0050896 | 5062 | 167 | 13711 | 214 | Oasl1,Iigp1,Gadd45g,Lgals9,Mt1,Icam1,Rgs16,Fyb,Nfkb2,Rassf4,Irgm1,Rtp4,Pik3r5,Map3k8,Birc3,Rhoj,Nfkbie,C3ar1,Il1rn,Csf3,Tnfaip8,Lcn2,Retnlg,Tnfaip3,Cxcl9,Ccl2,Vcam1,S100a9,Wfdc21,Nfkbiz,Ifitm2,Ptpn1,Tnip3,Iqgap1,Col4a1,Cxcl16,Ccl7,Parp14,Hcar2,Ccnd2,Il4ra,Slfn9,Akap12,Nfkbia,Ip6k2,Gm5431,Rasip1,Xaf1,Ifi211,Herc6,Mx2,Runx1,Resf1,Trim25,Timp1,Tiparp,Fam107a,Map3k6,Slfn2,Isg15,Sele,Ncf1,Trim30a,Cp,Acod1,Pik3ap1,Atf3,Rhou,Serpina3f,Nfe2l2,Rasd1,Gbp7,Noct,Selp,Angptl4,Msn,Tgm2,C5ar1,Clic4,Hspa5,Gm4841,Thbs1,Ifitm3,Mx1,P2ry6,Errfi1,Cybb,Bach1,Kcna5,Trim56,Plaur,Il17ra,Gbp4,Slfn4,Osmr,Nfkb1,F830016B08Rik,Pygm,Irak3,Arid5b,Ifi47,Adamts9,Ifi204,Plek,Ksr1,Tnfsf10,Xdh,Sgk3,Usp18,Bcl3,AA467197,Irgm2,Gbp6,Gbp3,S100a8,Tap1,Irf2,Ccl12,Irf7,Socs3,Dtx3l,Trib1,Pik3r1,Casp4,Oasl2,Gem,Il1a,Il6,Tgtp2,Cdkn1a,Stx11,Ifitm6,Ccl4,Spi1,Sgk1,Ptgs2,Ier3,Ifi209,Fzd4,Tgtp1,Cmpk2,Sdc4,Sbno2,Parp9,Hspb1,Slc24a4,Ifi207,Cd14,Cflar,Gpr84,Ripk1,Tnip1,Fstl1,Rhoc,Ifi44,Tnf,Saa1,Zfp36,Ifit3b,Csf2rb,Ifit2,Dnajb1,Rrp8,Cxcl1,Prg4,Hp,Mt2 | | 1.167e-34 | -78.13 | response to bacterium | biological process | GO:0009617 | 379 | 52 | 13711 | 214 | Lgals9,Iigp1,Nfkb1,Slfn4,Irgm1,Ccl2,Tnfaip3,Cxcl9,Lcn2,Ifi204,Irak3,Il1rn,Adamts9,Tnfaip8,Tnip3,Ccl12,Cxcl16,Gbp3,Bcl3,AA467197,Irgm2,Wfdc21,Gbp6,Usp18,Il6,Nfkbia,Trib1,Tgtp1,Cmpk2,Slfn2,Sbno2,Isg15,Ptgs2,Resf1,Ifi211,Herc6,Saa1,Zfp36,Tnf,Acod1,Ifi44,Trim30a,Ncf1,Cflar,Cd14,Hp,Mt2,Cxcl1,C5ar1,Gbp7,Noct,Serpina3f | | 4.823e-34 | -76.71 | GSE38681\_WT\_VS\_LYL1\_KO\_LYMPHOID\_PRIMED\_MULTIPOTENT\_PROGENITOR\_DN | MSigDB lists | GSE38681\_WT\_VS\_LYL1\_KO\_LYMPHOID\_PRIMED\_MULTIPOTENT\_PROGENITOR\_DN | 177 | 38 | 12187 | 179 | Usp18,Ifit2,Map3k8,Nfe2l2,Ripk1,Gbp7,Gbp3,Rtp4,Cmpk2,Nfkbiz,Hspa5,Gadd45g,Slfn9,Isg15,Sgk3,Tap1,Irf2,Parp9,Slfn3,Tor3a,Parp14,Lgals9,Birc3,Parp12,Oasl1,Gbp4,Irf7,Gbp6,Gem,Irgm2,Tnfsf10,Slfn4,Ifit3b,Socs3,Znfx1,Irf9,Clic4,Trim25 | | 1.394e-33 | -75.65 | GSE9316\_IL6\_KO\_VS\_IFNG\_KO\_INVIVO\_EXPANDED\_CD4\_TCELL\_DN | MSigDB lists | GSE9316\_IL6\_KO\_VS\_IFNG\_KO\_INVIVO\_EXPANDED\_CD4\_TCELL\_DN | 168 | 37 | 12187 | 179 | Rtp4,Cxcl1,Ptges,Csf3,Gadd45b,Tnfaip3,Ccl12,Gpr84,Ccl2,Ccl4,Maff,Rhou,Tap1,Cd14,Selp,Ifitm3,Ier3,Gadd45g,Nfkbiz,Tnfsf10,Xdh,Irf7,Cflar,Parp12,Oasl1,Nfkbia,Tor3a,Tnf,Clic4,Icam1,Saa1,Atf3,Col4a1,Timp1,Il1rn,Socs3,Cdkn1a | | 2.229e-33 | -75.18 | HALLMARK\_TNFA\_SIGNALING\_VIA\_NFKB | MSigDB lists | HALLMARK\_TNFA\_SIGNALING\_VIA\_NFKB | 170 | 37 | 12187 | 179 | Tap1,Sgk1,Tiparp,Nfkb1,Il1a,Ier3,Cebpd,Nfkbie,Cxcl1,Bcl3,Tnfaip8,Ifit2,Map3k8,Maff,Ccl4,Ccl12,Tnfaip3,Nfe2l2,Il6,Gadd45b,Ptgs2,Tnip1,Icam1,Zfp36,Plek,Plaur,Cdkn1a,Socs3,Atf3,Cflar,Gem,Nfkb2,Tnf,Birc3,Sdc4,Trib1,Nfkbia | | 5.590e-33 | -74.26 | GSE18281\_CORTICAL\_VS\_MEDULLARY\_THYMOCYTE\_UP | MSigDB lists | GSE18281\_CORTICAL\_VS\_MEDULLARY\_THYMOCYTE\_UP | 174 | 37 | 12187 | 179 | Gadd45g,Lcn2,Cebpd,Ifitm3,Isg15,Cd14,Slfn3,Tap1,Ifit2,Ccnd2,Tgm2,Ccl2,Usp18,Gadd45b,Cxcl1,Bcl3,Osmr,Cmpk2,Gbp3,Ifit3b,Il1rn,Znfx1,Rasd1,Atf3,Ptgs2,Saa1,Icam1,Plaur,Clic4,Irf9,Tnf,Casp4,Nfkbia,Irgm2,Irf7,Slfn4,Tnfsf10 | | 1.829e-32 | -73.08 | multi-organism process | biological process | GO:0051704 | 1450 | 89 | 13711 | 214 | Gbp3,A2m,S100a8,Irf2,Ccl12,Usp18,Bcl3,AA467197,Irgm2,Gbp6,Irak3,Adamts9,Ifi204,Gbp4,Slfn4,Nfkb1,Adamts1,Trim56,Il17ra,Cybb,Ifitm3,Mx1,Cxcl1,Hp,Mt2,Ifit3b,Ifit2,Ifi44,Tnf,Saa1,Zfp36,Cd14,Cflar,Ripk1,Tnip1,Tgtp1,Cmpk2,Sbno2,Parp9,Ifitm6,Stx11,Ccl4,Ptgs2,Ier3,Casp4,Oasl2,Il6,Irf7,Dtx3l,Trib1,Ifitm2,Tnip3,Cxcl16,Vcam1,S100a9,Wfdc21,Tnfaip3,Cxcl9,Ccl2,Il1rn,Zbtb16,Tnfaip8,Lcn2,Irgm1,Rtp4,Icam1,Oasl1,Iigp1,Lgals9,C5ar1,Clic4,Serpina3f,Noct,Gbp7,Acod1,Ncf1,Trim30a,Slfn2,Isg15,Ifi211,Herc6,Mx2,Trim25,Resf1,Slfn9,Il4ra,Nfkbia,Ccl7,Parp14 | | 2.051e-32 | -72.96 | GSE19888\_ADENOSINE\_A3R\_INH\_PRETREAT\_AND\_ACT\_BY\_A3R\_VS\_TCELL\_MEMBRANES\_ACT\_MAST\_CELL\_UP | MSigDB lists | GSE19888\_ADENOSINE\_A3R\_INH\_PRETREAT\_AND\_ACT\_BY\_A3R\_VS\_TCELL\_MEMBRANES\_ACT\_MAST\_CELL\_UP | 166 | 36 | 12187 | 179 | Tap1,Parp9,Pik3ap1,Slfn9,Slfn5,Herc6,Isg15,Rnf213,Ifitm3,Gbp3,Cmpk2,Rtp4,Gbp7,Dtx3l,Ccl12,Usp18,Ifit2,Irf9,Trim25,Znfx1,Samd9l,Timp1,Ifit3b,Ifi44,Tnfsf10,Irf7,Gbp6,Irgm2,Parp12,Oasl1,Gbp4,Tubb6,Casp4,Xaf1,Parp14,Tor3a | | 6.410e-32 | -71.82 | GSE36527\_CD69\_NEG\_VS\_POS\_TREG\_CD62L\_LOS\_KLRG1\_NEG\_UP | MSigDB lists | GSE36527\_CD69\_NEG\_VS\_POS\_TREG\_CD62L\_LOS\_KLRG1\_NEG\_UP | 171 | 36 | 12187 | 179 | Icam1,Trim25,Irf9,Clic4,Ifi44,Ifit3b,Socs3,Znfx1,Gbp6,Irgm2,Irf7,Slfn4,Tnfsf10,Casp4,Parp14,Tor3a,Parp12,Oasl1,Gbp4,Isg15,Selp,Slfn9,Slfn3,Tap1,Parp9,Gadd45g,Gbp7,Bcl3,Cmpk2,Rtp4,Gbp3,Ccnd2,Ifit2,Map3k8,Usp18,Ripk1 | | 1.247e-31 | -71.16 | GSE35825\_UNTREATED\_VS\_IFNG\_STIM\_MACROPHAGE\_UP | MSigDB lists | GSE35825\_UNTREATED\_VS\_IFNG\_STIM\_MACROPHAGE\_UP | 174 | 36 | 12187 | 179 | Slfn5,Irak3,Parp9,Il1a,Nfkbie,Csf3,Pik3r5,Ptges,Bcl3,Rtp4,Gbp3,Map3k8,Cybb,Hcar2,Ptgs2,Saa1,Plek,Clic4,Irf9,Cdkn1a,Vcam1,Ifit3b,Stx11,Il1rn,Hp,Znfx1,Cflar,Gbp6,Irf7,Igsf6,Birc3,Tor3a,Nfkbia,Casp4,Tubb6,Gbp4 | | 5.279e-31 | -69.72 | GSE13484\_UNSTIM\_VS\_YF17D\_VACCINE\_STIM\_PBMC\_DN | MSigDB lists | GSE13484\_UNSTIM\_VS\_YF17D\_VACCINE\_STIM\_PBMC\_DN | 153 | 34 | 12187 | 179 | Tnfsf10,Irf7,Cflar,Oasl1,Parp12,Xaf1,Casp4,Tnf,Irf9,Trim25,Atf3,Ifi204,Il1rn,Ifi207,Socs3,Ifit3b,Ifi209,Ifi44,Sbno2,Tnfaip8,Rtp4,Ifi211,Phf11d,Arid5b,Gadd45b,Il6,Ccl4,Usp18,Ifit2,Tap1,Ifitm3,Herc6,Isg15,Phf11b | | 2.139e-30 | -68.32 | GSE13485\_CTRL\_VS\_DAY7\_YF17D\_VACCINE\_PBMC\_DN | MSigDB lists | GSE13485\_CTRL\_VS\_DAY7\_YF17D\_VACCINE\_PBMC\_DN | 159 | 34 | 12187 | 179 | Oasl1,Parp12,Xaf1,Parp14,Tnfsf10,Irf7,C3ar1,Ifi204,Il1rn,Samd9l,Ifi207,Ifit3b,Ifi209,Ifi44,Irf9,Trim25,Ifitm2,Usp18,Ifit2,Msr1,Gbp3,Rtp4,Cmpk2,Ifi211,Phf11d,Dtx3l,Phf11b,Parp9,Tap1,Pik3ap1,Ifitm3,Herc6,Rnf213,Isg15 | | 2.328e-30 | -68.23 | GSE21360\_SECONDARY\_VS\_QUATERNARY\_MEMORY\_CD8\_TCELL\_UP | MSigDB lists | GSE21360\_SECONDARY\_VS\_QUATERNARY\_MEMORY\_CD8\_TCELL\_UP | 146 | 33 | 12187 | 179 | Ccl12,Tnfaip3,Il6,Gadd45b,Ccl4,Map3k8,Sbno2,Ptges,Cxcl1,Pik3r5,Bcl3,Lcn2,Nfkb1,Il1a,Nfkbiz,Irak3,Cd14,Nfkbia,Casp4,Tnf,Nfkb2,S100a9,Cflar,Tnip3,Il1rn,Stx11,Socs3,Vcam1,Clic4,Zfp36,Plek,Tnip1,Ptgs2 | | 2.451e-30 | -68.18 | cellular response to cytokine stimulus | biological process | GO:0071345 | 529 | 55 | 13711 | 214 | F830016B08Rik,Nfkb1,Birc3,Osmr,Gbp4,Il17ra,Irgm1,Icam1,Iigp1,Gm4841,Ifitm3,Ccl12,Ifitm2,Gbp3,Irgm2,Gbp6,Ccl2,Cxcl9,Ifi204,Ifi47,Csf3,Il1rn,Irak3,Arid5b,Sbno2,Tgtp1,Fzd4,Ifi209,Trim25,Spi1,Ccl4,Stx11,Ifitm6,Ifi211,Tgtp2,Il6,Gm5431,Il1a,Nfkbia,Il4ra,Ccl7,Irf7,Socs3,Hspa5,Cxcl1,Ifit2,Selp,Gbp7,Csf2rb,Nfe2l2,Zfp36,Tnf,Acod1,Ripk1,Ifi207 | | 2.666e-30 | -68.10 | GSE18281\_SUBCAPSULAR\_VS\_CENTRAL\_CORTICAL\_REGION\_OF\_THYMUS\_DN | MSigDB lists | GSE18281\_SUBCAPSULAR\_VS\_CENTRAL\_CORTICAL\_REGION\_OF\_THYMUS\_DN | 174 | 35 | 12187 | 179 | Cxcl16,Tap1,Slfn3,Nfkbiz,Lcn2,Mmp8,Gbp7,Nfkbie,S100a8,Cmpk2,Usp18,Ifit2,Tnfaip3,Cybb,Hcar2,Clic4,Socs3,Ifit3b,Cdkn1a,Ifi44,Znfx1,Timp1,Hp,S100a9,Irf7,Irgm2,Cflar,Slfn4,Parp14,Tor3a,Casp4,Tnf,Lgals9,Oasl1,Tubb6 | | 2.827e-30 | -68.04 | GSE18791\_CTRL\_VS\_NEWCASTLE\_VIRUS\_DC\_8H\_DN | MSigDB lists | GSE18791\_CTRL\_VS\_NEWCASTLE\_VIRUS\_DC\_8H\_DN | 134 | 32 | 12187 | 179 | Ptgs2,Errfi1,Trim25,Ifit3b,Ifi44,Znfx1,Samd9l,Trim56,Irf7,Tnfsf10,Xaf1,Parp14,Tnf,Parp12,Oasl1,Slfn5,Ifitm3,Isg15,Herc6,Rnf213,Parp9,Tap1,Phf11b,Phf11d,Bcl3,Dtx3l,Gbp3,Cmpk2,Rtp4,Usp18,Ifit2,Gadd45b | | 3.772e-30 | -67.75 | GSE2706\_UNSTIM\_VS\_2H\_R848\_DC\_DN | MSigDB lists | GSE2706\_UNSTIM\_VS\_2H\_R848\_DC\_DN | 148 | 33 | 12187 | 179 | Plek,Icam1,Plaur,Ptgs2,Stx11,Ifit3b,Socs3,Gem,Cflar,AA467197,Birc3,Zfp189,Sdc4,Nfkbia,Tap1,Ptpn1,Herc6,Isg15,Il1a,Nfkb1,Nfkbiz,Cmpk2,Tnfaip8,Bcl3,Pik3r5,Cxcl1,Il6,Gadd45b,Tnfaip3,Maff,Map3k8,Ifit2,Ccl4 | | 6.067e-30 | -67.27 | GSE2706\_UNSTIM\_VS\_2H\_LPS\_DC\_DN | MSigDB lists | GSE2706\_UNSTIM\_VS\_2H\_LPS\_DC\_DN | 150 | 33 | 12187 | 179 | Irf7,Gem,Cflar,Oasl1,AA467197,Nfkbia,Parp14,Birc3,Plaur,Icam1,Tnip1,Atf3,Znfx1,Stx11,Tnip3,Ifit3b,Socs3,Ifi44,Cdkn1a,Tnfaip8,Pik3r5,Cxcl1,Il6,Gadd45b,Tnfaip3,Map3k8,Tap1,Ptpn1,Herc6,Isg15,Il1a,Nfkb1,Nfkbiz | | 8.118e-30 | -66.98 | defense response to other organism | biological process | GO:0098542 | 473 | 52 | 13711 | 214 | Ifit2,Gbp7,Ifit3b,Hp,C5ar1,Trim30a,Cd14,Ncf1,Acod1,Tnf,Trim25,Ccl4,Herc6,Mx2,Stx11,Ifitm6,Isg15,Parp9,Tgtp1,Ccl7,Parp14,Irf7,Dtx3l,Il6,Casp4,Il4ra,Oasl2,Slfn9,Irgm2,Gbp6,Bcl3,S100a9,Irf2,Ccl12,Cxcl16,S100a8,Ifitm2,Gbp3,Lcn2,Tnfaip8,Ccl2,Cxcl9,Il17ra,Trim56,Rtp4,Irgm1,Gbp4,Mx1,Iigp1,Oasl1,Ifitm3,Cybb | | 1.558e-29 | -66.33 | GSE45365\_NK\_CELL\_VS\_CD11B\_DC\_DN | MSigDB lists | GSE45365\_NK\_CELL\_VS\_CD11B\_DC\_DN | 168 | 34 | 12187 | 179 | S100a9,Sdc4,Nfkbia,Birc3,AA467197,Tubb6,Tnip1,Saa1,Msn,Csf2rb,Clic4,Zfp36,Sele,Icam1,Il1rn,Timp1,Stx11,Ptges,Cxcl1,Csf3,S100a8,Osmr,Adamts9,Sntb2,Spi1,Ccl4,Tgm2,Maff,Il6,Tnfaip3,Cd14,Nfkbiz,Ier3,Tiparp | | 2.139e-29 | -66.01 | immune response | biological process | GO:0006955 | 625 | 58 | 13711 | 214 | Prg4,Cxcl1,Gbp7,Ifit2,Ifi44,Acod1,Tnf,Trim30a,Cd14,Tgtp1,Parp9,Isg15,Sbno2,Trim25,Stx11,Ifitm6,Mx2,Herc6,Ccl4,Il6,Il4ra,Oasl2,Casp4,Il1a,Dtx3l,Irf7,Parp14,Ccl7,S100a8,Tap1,Cxcl16,Ccl12,Gbp3,Ifitm2,Bcl3,Gbp6,Irgm2,S100a9,Tnfsf10,Ccl2,Tnfaip3,Cxcl9,Lcn2,Il1rn,C3ar1,Csf3,Gbp4,Trim56,Irgm1,Cybb,Icam1,Fyb,Nfkb2,Gadd45g,Mx1,Ifitm3,Oasl1,Iigp1 | | 4.442e-29 | -65.28 | GO\_DEFENSE\_RESPONSE | MSigDB lists | GO\_DEFENSE\_RESPONSE | 718 | 59 | 12187 | 179 | Ip6k2,Irf2,Il4ra,Ifit2,Ccl2,Tnfaip3,Bcl3,Cxcl1,Ifi211,S100a8,Ifi209,Hp,Ifi204,Saa1,Icam1,Trim25,Irf9,Casp4,Oasl1,S100a9,Irf7,Thbs1,Nfkbiz,Il1a,Nfkb1,Lcn2,Isg15,Ifitm3,Selp,Cd14,Il17ra,Tap1,Cxcl16,Ccl4,Cybb,Nfe2l2,Il6,Ccl12,Ptges,Ifit3b,Vcam1,Ifi207,Il1rn,Tnip3,Ptgs2,Ifitm2,Ncf1,Tnip1,Sele,Zfp189,Tnf,Xaf1,Gbp4,Gbp6,C3ar1,Irgm2,C5ar1,Trim56,Nfkb2 | | 6.901e-29 | -64.84 | GSE19888\_ADENOSINE\_A3R\_INH\_VS\_ACT\_WITH\_INHIBITOR\_PRETREATMENT\_IN\_MAST\_CELL\_UP | MSigDB lists | GSE19888\_ADENOSINE\_A3R\_INH\_VS\_ACT\_WITH\_INHIBITOR\_PRETREATMENT\_IN\_MAST\_CELL\_UP | 147 | 32 | 12187 | 179 | Ifitm3,Isg15,Rnf213,Herc6,Slfn5,Slfn9,Pik3ap1,Parp9,Tap1,Dtx3l,Gbp7,Cmpk2,Rtp4,Gbp3,Ifit2,Ccl2,Usp18,Clic4,Irf9,Ifi44,Ifit3b,Samd9l,Znfx1,Irgm2,Gbp6,Irf7,Tnfsf10,Parp14,Xaf1,Gbp4,Parp12,Oasl1 | | 1.364e-28 | -64.16 | GSE21546\_UNSTIM\_VS\_ANTI\_CD3\_STIM\_ELK1\_KO\_DP\_THYMOCYTES\_UP | MSigDB lists | GSE21546\_UNSTIM\_VS\_ANTI\_CD3\_STIM\_ELK1\_KO\_DP\_THYMOCYTES\_UP | 124 | 30 | 12187 | 179 | Ifit3b,Ifi44,Ifi209,Znfx1,Ifi207,Ifi204,Irf9,Csf2rb,Trim25,Xaf1,Parp14,Parp12,Oasl1,Trim56,Irf7,C5ar1,Phf11b,Rnf213,Herc6,Isg15,Ifitm3,Tap1,Parp9,Usp18,Ifit2,Ifi211,Phf11d,Dtx3l,Rtp4,Cmpk2 | | 5.749e-28 | -62.72 | NEMETH\_INFLAMMATORY\_RESPONSE\_LPS\_UP | MSigDB lists | NEMETH\_INFLAMMATORY\_RESPONSE\_LPS\_UP | 84 | 26 | 12187 | 179 | Slfn4,C3ar1,C5ar1,Cflar,Nfkbia,Casp4,Tnf,Trim25,Icam1,Tnip1,Ptgs2,Atf3,Ifi207,Il1rn,Ifi204,Cdkn1a,Ifi209,Rgs16,Ifi211,Rhoc,Msr1,Tap1,Slfn3,Ier3,Cebpd,Nfkb1 | | 8.219e-28 | -62.37 | GO\_IMMUNE\_SYSTEM\_PROCESS | MSigDB lists | GO\_IMMUNE\_SYSTEM\_PROCESS | 1290 | 75 | 12187 | 179 | C3ar1,Gem,Gbp6,C5ar1,Irgm2,Trim56,Igsf6,Nfkb2,Birc3,Tnf,Nfkbia,Xaf1,Gbp4,Ifitm2,Ncf1,Tnip1,Rbm47,Sele,Msn,Ifit3b,Vcam1,Zbtb16,Ifi207,Il1rn,Tnip3,Csf3,Sbno2,Map3k8,Ccl4,Cybb,Il6,Ccl12,Herc6,Isg15,Selp,Ifitm3,Cd14,Pik3ap1,Il17ra,Irak3,Tap1,Cxcl16,Thbs1,Il1a,Nfkb1,Tiparp,Lcn2,Irf7,S100a9,Tnfsf10,Runx1,Casp4,Oasl1,Saa1,Plek,Trim25,Icam1,Irf9,Ifi209,Hp,Ifi204,Bcl3,Cxcl1,Ifi211,S100a8,Il4ra,Pik3r1,Ifit2,Spi1,Ccl2,Hcar2,Ripk1,Tnfaip3,Ip6k2,Irf2 | | 1.308e-27 | -61.90 | GSE33424\_CD161\_INT\_VS\_NEG\_CD8\_TCELL\_UP | MSigDB lists | GSE33424\_CD161\_INT\_VS\_NEG\_CD8\_TCELL\_UP | 175 | 33 | 12187 | 179 | Dtx3l,Gbp7,Rtp4,Cmpk2,Usp18,Ccnd2,Ifit2,Cybb,Slfn9,Slfn5,Herc6,Isg15,Rnf213,Ifitm3,Parp9,Slfn3,Hspa5,Irf7,Gbp6,Irgm2,Tnfsf10,Slfn4,Casp4,Tor3a,Parp14,Xaf1,Oasl1,Parp12,Gbp4,Irf9,Ifit3b,Ifi44,Samd9l | | 1.533e-27 | -61.74 | GSE2706\_UNSTIM\_VS\_2H\_LPS\_AND\_R848\_DC\_DN | MSigDB lists | GSE2706\_UNSTIM\_VS\_2H\_LPS\_AND\_R848\_DC\_DN | 147 | 31 | 12187 | 179 | Cxcl1,Tnfaip8,Cmpk2,Rtp4,Map3k8,Ifit2,Tnfaip3,Il6,Isg15,Herc6,Nfkbiz,Nfkb1,Il1a,Cflar,Gem,Irf7,Birc3,Nfkbia,AA467197,Oasl1,Ptgs2,Tnip1,Icam1,Plaur,Cdkn1a,Ifi44,Socs3,Ifit3b,Stx11,Znfx1,Atf3 | | 1.933e-27 | -61.51 | GSE35685\_CD34POS\_CD38NEG\_VS\_CD34POS\_CD10POS\_BONE\_MARROW\_DN | MSigDB lists | GSE35685\_CD34POS\_CD38NEG\_VS\_CD34POS\_CD10POS\_BONE\_MARROW\_DN | 177 | 33 | 12187 | 179 | Tiparp,Nfkb1,Gadd45g,Ier3,Cebpd,Nfkbiz,Il17ra,Ptpn1,Tap1,Selp,Isg15,Ccl12,Ifit2,Map3k8,Il4ra,Ccl4,Ccl2,Usp18,Gbp3,Gbp7,Bcl3,Timp1,Znfx1,Aff1,Zfp36,Csf2rb,Irf9,Bach1,Lgals9,Tor3a,Nfkbia,Casp4,Irgm2 | | 2.948e-27 | -61.09 | GO\_RESPONSE\_TO\_BIOTIC\_STIMULUS | MSigDB lists | GO\_RESPONSE\_TO\_BIOTIC\_STIMULUS | 553 | 51 | 12187 | 179 | Hspa5,S100a8,Bcl3,Cxcl1,Ifi211,Tnfaip3,Il4ra,Ifit2,Irf9,Icam1,Trim25,Hp,Ifi204,Ifi209,S100a9,Irf7,Oasl1,Trib1,Cxcl16,Il17ra,Irak3,Cd14,Isg15,Ifitm3,Selp,Nfkb1,Sbno2,Cmpk2,Ptges,Csf3,Il6,Ccl12,Ccl4,Sele,Hspb1,Ifitm2,Ptgs2,Ifi207,Tnip3,Ifit3b,Vcam1,Socs3,Ifi44,Nfkb2,Trim56,Gbp6,C5ar1,Gbp4,Nfkbia,Zfp189,Tnf | | 5.658e-27 | -60.44 | GSE18791\_UNSTIM\_VS\_NEWCATSLE\_VIRUS\_DC\_10H\_DN | MSigDB lists | GSE18791\_UNSTIM\_VS\_NEWCATSLE\_VIRUS\_DC\_10H\_DN | 126 | 29 | 12187 | 179 | Oasl1,Xaf1,Parp14,Tnf,Tnfsf10,Irf7,Znfx1,Samd9l,Stx11,Ifit3b,Ifi44,Ptgs2,Gadd45b,Il6,Usp18,Map3k8,Ifit2,Gbp3,Rtp4,Cmpk2,Phf11d,Dtx3l,Nfkbiz,Phf11b,Tap1,Parp9,Slfn5,Isg15,Herc6 | | 9.982e-27 | -59.87 | GO\_IMMUNE\_RESPONSE | MSigDB lists | GO\_IMMUNE\_RESPONSE | 594 | 52 | 12187 | 179 | Irf9,Trim25,Icam1,Saa1,Ifi204,Ifi209,Tnfsf10,Irf7,S100a9,Oasl1,Casp4,Irf2,Ip6k2,S100a8,Bcl3,Ifi211,Cxcl1,Ccl2,Il4ra,Ifit2,Ncf1,Ifitm2,Ifi207,Il1rn,Ifit3b,Vcam1,Nfkb2,Igsf6,Trim56,C3ar1,Gem,Gbp6,Irgm2,C5ar1,Gbp4,Xaf1,Tnf,Tap1,Cxcl16,Cd14,Isg15,Ifitm3,Lcn2,Il1a,Nfkb1,Thbs1,Sbno2,Csf3,Il6,Ccl12,Cybb,Ccl4 | | 1.376e-26 | -59.55 | ZHANG\_RESPONSE\_TO\_IKK\_INHIBITOR\_AND\_TNF\_UP | MSigDB lists | ZHANG\_RESPONSE\_TO\_IKK\_INHIBITOR\_AND\_TNF\_UP | 172 | 32 | 12187 | 179 | Tnfsf10,Nfkb2,Oasl1,AA467197,Birc3,Nfkbia,Sdc4,Icam1,Irf9,Plaur,Tnip1,Il1rn,Atf3,Znfx1,Cdkn1a,Ifit3b,Socs3,Cmpk2,Tnfaip8,Nfkbie,Bcl3,Cxcl1,Tnfaip3,Maff,Usp18,Tap1,Cxcl16,Slfn5,Il1a,Nfkb1,Sgk1,Nfkbiz | | 2.730e-26 | -58.86 | response to organic substance | biological process | GO:0010033 | 1870 | 92 | 13711 | 214 | Isg15,Tiparp,Timp1,Fam107a,Runx1,Trim25,Xaf1,Ifi211,Ip6k2,Gm5431,Il4ra,Nfkbia,Ccl7,C5ar1,Hspa5,Gbp7,Noct,Selp,Msn,Serpina3f,Nfe2l2,Atf3,Acod1,Sele,Birc3,Irgm1,Icam1,Lgals9,Iigp1,Tnip3,Iqgap1,Col4a1,Cxcl16,Ifitm2,Ptpn1,Wfdc21,Vcam1,Ccl2,Tnfaip3,Cxcl9,Il1rn,Csf3,Tgtp1,Cmpk2,Sbno2,Ifi209,Fzd4,Sgk1,Ptgs2,Stx11,Ifitm6,Ccl4,Spi1,Il6,Tgtp2,Casp4,Il1a,Pik3r1,Irf7,Socs3,Trib1,Hp,Cxcl1,Csf2rb,Ifit2,Zfp36,Tnf,Hspb1,Ifi207,Cd14,Cflar,Ripk1,Nfkb1,F830016B08Rik,Gbp4,Osmr,Trim56,Il17ra,P2ry6,Thbs1,Ifitm3,Gm4841,Ccl12,Gbp3,Irgm2,Gbp6,Usp18,Ifi204,Arid5b,Pygm,Irak3,Ifi47 | | 4.981e-26 | -58.26 | GSE21546\_WT\_VS\_SAP1A\_KO\_DP\_THYMOCYTES\_UP | MSigDB lists | GSE21546\_WT\_VS\_SAP1A\_KO\_DP\_THYMOCYTES\_UP | 149 | 30 | 12187 | 179 | Phf11b,Ifitm3,Rnf213,Isg15,Herc6,Irf2,Parp9,Tap1,Ifit2,Usp18,Dtx3l,Ifi211,Phf11d,Cmpk2,Ifi209,Ifi44,Ifit3b,Ifi204,Samd9l,Ifi207,Znfx1,Ifitm2,Trim25,Irf9,Parp14,Xaf1,Parp12,Oasl1,Irf7,Trim56 | | 9.856e-26 | -57.58 | response to chemical | biological process | GO:0042221 | 2591 | 108 | 13711 | 214 | Ptpn1,Ifitm2,Cxcl16,Col4a1,Iqgap1,Tnip3,S100a9,Vcam1,Wfdc21,Cxcl9,Tnfaip3,Ccl2,Csf3,C3ar1,Il1rn,Retnlg,Lcn2,Birc3,Nfkbie,Rtp4,Irgm1,Icam1,Mt1,Iigp1,Lgals9,Clic4,Hspa5,C5ar1,Nfe2l2,Serpina3f,Msn,Gbp7,Noct,Selp,Acod1,Atf3,Ncf1,Sele,Cp,Fam107a,Tiparp,Timp1,Isg15,Xaf1,Ifi211,Trim25,Runx1,Nfkbia,Il4ra,Gm5431,Ip6k2,Ccl7,Gbp3,Ccl12,S100a8,Usp18,Gbp6,Irgm2,Xdh,Ifi47,Arid5b,Pygm,Irak3,Ifi204,Osmr,Gbp4,F830016B08Rik,Nfkb1,Bach1,Il17ra,Trim56,Cybb,Gm4841,Thbs1,Ifitm3,P2ry6,Cxcl1,Mt2,Hp,Ifit2,Csf2rb,Tnf,Zfp36,Saa1,Ripk1,Cflar,Cd14,Ifi207,Hspb1,Fzd4,Ifi209,Sbno2,Cmpk2,Tgtp1,Ccl4,Spi1,Stx11,Ifitm6,Sgk1,Ptgs2,Il1a,Casp4,Tgtp2,Il6,Trib1,Irf7,Socs3,Pik3r1 | | 1.717e-25 | -57.02 | cellular response to chemical stimulus | biological process | GO:0070887 | 1836 | 90 | 13711 | 214 | Iigp1,Icam1,Mt1,Irgm1,Birc3,Csf3,Il1rn,Retnlg,Lcn2,Cxcl9,Tnfaip3,Ccl2,S100a9,Vcam1,Ptpn1,Ifitm2,Cxcl16,Iqgap1,Col4a1,Tnip3,Ccl7,Nfkbia,Il4ra,Gm5431,Ip6k2,Ifi211,Trim25,Runx1,Fam107a,Tiparp,Ncf1,Acod1,Atf3,Nfe2l2,Msn,Selp,Gbp7,Hspa5,Clic4,C5ar1,Thbs1,Gm4841,Ifitm3,P2ry6,Cybb,Bach1,Il17ra,Osmr,Gbp4,F830016B08Rik,Nfkb1,Ifi47,Irak3,Arid5b,Ifi204,Gbp6,Irgm2,Gbp3,Ccl12,S100a8,Irf7,Socs3,Pik3r1,Il1a,Casp4,Tgtp2,Il6,Ccl4,Spi1,Ifitm6,Stx11,Sgk1,Fzd4,Ifi209,Sbno2,Cmpk2,Tgtp1,Ripk1,Cflar,Cd14,Ifi207,Hspb1,Tnf,Zfp36,Saa1,Ifit2,Csf2rb,Cxcl1,Mt2,Hp | | 3.503e-25 | -56.31 | GSE18791\_UNSTIM\_VS\_NEWCATSLE\_VIRUS\_DC\_6H\_DN | MSigDB lists | GSE18791\_UNSTIM\_VS\_NEWCATSLE\_VIRUS\_DC\_6H\_DN | 144 | 29 | 12187 | 179 | Tnfsf10,Trim56,Irf7,Oasl1,Xaf1,Parp14,Irf9,Trim25,Znfx1,Ifi204,Samd9l,Ifi207,Ifit3b,Ifi209,Ifi44,Rtp4,Cmpk2,Ifi211,Phf11d,Dtx3l,Ripk1,Usp18,Ifit2,Parp9,Tap1,Herc6,Isg15,Rnf213,Phf11b | | 5.015e-25 | -55.95 | response to stress | biological process | GO:0006950 | 2282 | 100 | 13711 | 214 | Gbp3,S100a8,Tap1,Ccl12,Irf2,Bcl3,Gbp6,Irgm2,Plek,Pygm,Ifi47,Ifi204,Gbp4,Nfkb1,F830016B08Rik,Bach1,Trim56,Il17ra,Cybb,Errfi1,Ifitm3,Thbs1,Gm4841,Mx1,Rrp8,Dnajb1,Cxcl1,Mt2,Hp,Ifit3b,Ifit2,Tnf,Zfp36,Saa1,Hspb1,Ripk1,Cd14,Cflar,Fstl1,Tnip1,Tgtp1,Parp9,Sdc4,Ifitm6,Stx11,Ccl4,Ptgs2,Sgk1,Ier3,Oasl2,Casp4,Il1a,Il6,Cdkn1a,Tgtp2,Dtx3l,Irf7,Trib1,Pik3r1,Nfkbiz,Ptpn1,Ifitm2,Cxcl16,S100a9,Tnfaip3,Cxcl9,Ccl2,Il1rn,C3ar1,Tnfaip8,Lcn2,Map3k8,Irgm1,Rtp4,Icam1,Mt1,Oasl1,Iigp1,C5ar1,Hspa5,Nfe2l2,Angptl4,Selp,Gbp7,Acod1,Atf3,Ncf1,Sele,Trim30a,Timp1,Isg15,Mx2,Herc6,Runx1,Trim25,Il4ra,Slfn9,Gm5431,Parp14,Ccl7 | | 5.461e-25 | -55.87 | GO\_INFLAMMATORY\_RESPONSE | MSigDB lists | GO\_INFLAMMATORY\_RESPONSE | 283 | 37 | 12187 | 179 | Il1a,Nfkb1,Nfkbiz,Thbs1,Cd14,Selp,Il6,Nfe2l2,Tnfaip3,Ccl12,Cybb,Ccl2,Ccl4,Il4ra,S100a8,Ptges,Cxcl1,Ifi211,Hp,Ifi207,Tnip3,Il1rn,Ifi204,Vcam1,Ifi209,Sele,Icam1,Tnip1,Saa1,Ptgs2,Casp4,Tnf,Nfkb2,S100a9,C3ar1,Irgm2,C5ar1 | | 5.654e-25 | -55.83 | GSE19888\_ADENOSINE\_A3R\_INH\_VS\_TCELL\_MEMBRANES\_ACT\_MAST\_CELL\_UP | MSigDB lists | GSE19888\_ADENOSINE\_A3R\_INH\_VS\_TCELL\_MEMBRANES\_ACT\_MAST\_CELL\_UP | 161 | 30 | 12187 | 179 | Gbp7,Dtx3l,Cmpk2,Rtp4,Gbp3,Ifit2,Usp18,Sgk3,Isg15,Rnf213,Herc6,Slfn5,Slfn9,Tap1,Parp9,Gbp6,Irgm2,Trim56,Tnfsf10,Casp4,Parp14,Xaf1,Parp12,Gbp4,Trim25,Irf9,Clic4,Ifi44,Ifit3b,Samd9l | | 6.564e-25 | -55.68 | GSE9988\_ANTI\_TREM1\_VS\_LOW\_LPS\_MONOCYTE\_DN | MSigDB lists | GSE9988\_ANTI\_TREM1\_VS\_LOW\_LPS\_MONOCYTE\_DN | 147 | 29 | 12187 | 179 | Nfkbiz,Nfkb1,Il1a,Ier3,Pik3ap1,Map3k8,Rhou,Ccl4,Gpr84,Tnfaip3,Il6,Nfe2l2,Csf3,Cxcl1,Tnfaip8,Socs3,Stx11,Tnip3,Ptgs2,Tnip1,Icam1,Clic4,Tnf,Birc3,Nfkbia,Sdc4,Oasl1,Cflar,Igsf6 | | 6.738e-25 | -55.66 | response to interferon-beta | biological process | GO:0035456 | 45 | 20 | 13711 | 214 | Tgtp2,Gm5431,Acod1,Ifi204,Iigp1,Ifi47,Gm4841,Ifi207,Ifitm3,F830016B08Rik,Tgtp1,Ifitm2,Gbp3,Ifi209,Gbp6,Irgm2,Ifi211,Irgm1,Xaf1,Ifitm6 | | 8.302e-25 | -55.45 | GSE26030\_TH1\_VS\_TH17\_DAY5\_POST\_POLARIZATION\_UP | MSigDB lists | GSE26030\_TH1\_VS\_TH17\_DAY5\_POST\_POLARIZATION\_UP | 163 | 30 | 12187 | 179 | Hspa5,Parp9,Slfn3,Slfn9,Slfn5,Ifitm3,Herc6,Isg15,Rnf213,Usp18,Ifit2,Cmpk2,Rtp4,Gbp7,Dtx3l,Samd9l,Ifit3b,Ifi44,Irf9,Gbp4,Parp12,Oasl1,Parp14,Tor3a,Xaf1,Tnfsf10,Slfn4,Irf7,Irgm2,Gbp6 | | 3.730e-24 | -53.95 | GSE2706\_UNSTIM\_VS\_8H\_R848\_DC\_DN | MSigDB lists | GSE2706\_UNSTIM\_VS\_8H\_R848\_DC\_DN | 141 | 28 | 12187 | 179 | Ifit3b,Socs3,Ifi44,Samd9l,Stx11,Tnfsf10,Xaf1,Parp14,Birc3,Tnf,AA467197,Slfn5,Isg15,Rnf213,Herc6,Parp9,Nfkbiz,Thbs1,Sox11,Nfkb1,Cmpk2,Tnfaip8,Usp18,Ifit2,Map3k8,Il6,Gadd45b,Tnfaip3 | | 4.377e-24 | -53.79 | GSE43863\_TFH\_VS\_LY6C\_LOW\_CXCR5NEG\_EFFECTOR\_CD4\_TCELL\_UP | MSigDB lists | GSE43863\_TFH\_VS\_LY6C\_LOW\_CXCR5NEG\_EFFECTOR\_CD4\_TCELL\_UP | 172 | 30 | 12187 | 179 | Nfkb1,Ms4a6d,Rnf213,Slfn9,Pik3ap1,Parp9,Tap1,Ifit2,Cybb,Il6,Gadd45b,Dtx3l,Bcl3,Cmpk2,Rtp4,Gbp3,Socs3,Ksr1,Znfx1,Ncf1,Irf9,Lgals9,Xaf1,Parp14,Nfkbia,Gbp4,Parp12,Irgm2,Gbp6,Irf7 | | 5.611e-24 | -53.54 | GO\_RESPONSE\_TO\_CYTOKINE | MSigDB lists | GO\_RESPONSE\_TO\_CYTOKINE | 487 | 45 | 12187 | 179 | Oasl1,Irf7,Icam1,Trim25,Irf9,Ripk1,Ifit2,Il4ra,Ccl2,Cxcl1,Hspa5,Ip6k2,Irf2,Gbp4,Tnf,Birc3,Xaf1,Nfkbia,Nfkb2,Gbp6,Trim56,Timp1,Socs3,Vcam1,Ifit3b,Sele,Csf2rb,Ptgs2,Ifitm2,Ccl12,Nfe2l2,Il6,Ccl4,Osmr,Sbno2,Csf3,Ptges,Nfkb1,Il1a,Irak3,Il17ra,Cxcl16,Ifitm3,Isg15,Cd14 | | 8.466e-24 | -53.13 | GSE42021\_TREG\_VS\_TCONV\_PLN\_UP | MSigDB lists | GSE42021\_TREG\_VS\_TCONV\_PLN\_UP | 160 | 29 | 12187 | 179 | Oasl1,Parp12,Xaf1,Tnfsf10,Irf7,Ifi207,Ifi204,Atf3,Ifi44,Ifi209,Ifit3b,Irf9,Plaur,Ifitm2,Ccl12,Maff,Ifit2,Usp18,Rtp4,Osmr,Phf11d,Cxcl1,Ifi211,Cebpd,Phf11b,Tap1,Herc6,Isg15,Ifitm3 | | 1.035e-23 | -52.93 | GSE18791\_CTRL\_VS\_NEWCASTLE\_VIRUS\_DC\_10H\_DN | MSigDB lists | GSE18791\_CTRL\_VS\_NEWCASTLE\_VIRUS\_DC\_10H\_DN | 146 | 28 | 12187 | 179 | Dtx3l,Phf11d,Rtp4,Cmpk2,Gbp3,Ifit2,Map3k8,Usp18,Il6,Tnfaip3,Herc6,Rnf213,Isg15,Slfn9,Tap1,Irf2,Phf11b,Tnfsf10,Tnf,Xaf1,Parp14,Oasl1,Ptgs2,Ifi44,Ifit3b,Samd9l,Stx11,Znfx1 | | 1.771e-23 | -52.39 | GSE22140\_GERMFREE\_VS\_SPF\_MOUSE\_CD4\_TCELL\_UP | MSigDB lists | GSE22140\_GERMFREE\_VS\_SPF\_MOUSE\_CD4\_TCELL\_UP | 164 | 29 | 12187 | 179 | Tnf,Trib1,Casp4,Xaf1,Oasl1,Gem,Irf7,Tnfsf10,Ifi44,Cdkn1a,Ifi209,Ifit3b,Socs3,Ifi207,Ifi204,Plek,Zfp36,Icam1,Trim25,Irf9,Csf2rb,Ifit2,Ccl2,Gadd45b,Bcl3,Ifi211,Sbno2,Isg15,Tap1 | | 1.872e-23 | -52.33 | GSE18791\_CTRL\_VS\_NEWCASTLE\_VIRUS\_DC\_6H\_DN | MSigDB lists | GSE18791\_CTRL\_VS\_NEWCASTLE\_VIRUS\_DC\_6H\_DN | 149 | 28 | 12187 | 179 | Ifit2,Usp18,Dtx3l,Bcl3,Phf11d,Angptl4,Cmpk2,Rtp4,Phf11b,Rnf213,Herc6,Isg15,Tap1,Parp9,Parp14,Xaf1,Parp12,Oasl1,Trim56,Irf7,Tnfsf10,Ifi44,Ifit3b,Samd9l,Stx11,Znfx1,Trim25,Errfi1 | | 2.389e-23 | -52.09 | GALINDO\_IMMUNE\_RESPONSE\_TO\_ENTEROTOXIN | MSigDB lists | GALINDO\_IMMUNE\_RESPONSE\_TO\_ENTEROTOXIN | 74 | 22 | 12187 | 179 | Tnip1,Cd14,Ptgs2,Errfi1,Csf2rb,Plaur,Icam1,Slfn3,Socs3,Ier3,Nfkb1,Il1rn,Cxcl1,Bcl3,Csf3,Nfkb2,Slfn4,Ccl4,Nfkbia,Tnf,Tnfaip3,Gadd45b | | 5.879e-23 | -51.19 | GSE9601\_NFKB\_INHIBITOR\_VS\_PI3K\_INHIBITOR\_TREATED\_HCMV\_INF\_MONOCYTE\_DN | MSigDB lists | GSE9601\_NFKB\_INHIBITOR\_VS\_PI3K\_INHIBITOR\_TREATED\_HCMV\_INF\_MONOCYTE\_DN | 155 | 28 | 12187 | 179 | Tubb6,Sdc4,Trib1,Tnfsf10,Irf7,Gem,Znfx1,Il1rn,Timp1,Ifit3b,Cdkn1a,Clic4,Saa1,Ptgs2,Ccl12,Cybb,Usp18,Ccl2,Ccl4,Maff,Ifit2,Map3k8,Rhou,Rtp4,Csf3,Il1a,Cd14,Selp | | 6.131e-23 | -51.15 | GSE26343\_UNSTIM\_VS\_LPS\_STIM\_NFAT5\_KO\_MACROPHAGE\_DN | MSigDB lists | GSE26343\_UNSTIM\_VS\_LPS\_STIM\_NFAT5\_KO\_MACROPHAGE\_DN | 171 | 29 | 12187 | 179 | Cflar,Nfkb2,Igsf6,Casp4,Tnip1,Saa1,Csf2rb,Errfi1,Icam1,Zfp36,Socs3,Cdkn1a,Il1rn,Cxcl1,Pik3r5,Bcl3,Nfkbie,Gbp3,Gpr84,Map3k8,Maff,Nfe2l2,Gadd45b,Il6,Cd14,Cxcl16,Nfkbiz,Ier3,Il1a | | 1.025e-22 | -50.63 | GSE34392\_ST2\_KO\_VS\_WT\_DAY8\_LCMV\_EFFECTOR\_CD8\_TCELL\_DN | MSigDB lists | GSE34392\_ST2\_KO\_VS\_WT\_DAY8\_LCMV\_EFFECTOR\_CD8\_TCELL\_DN | 174 | 29 | 12187 | 179 | Gadd45b,Il6,Nfe2l2,Hcar2,Tgm2,Gpr84,Ccl2,Usp18,Ifit2,Cmpk2,Cxcl1,Bcl3,Nfkbie,Nfkbiz,Irak3,Isg15,Tor3a,Parp14,Casp4,Birc3,Nfkb2,Cflar,Gem,Znfx1,Atf3,Stx11,Plek,Tagln2,Saa1 | | 1.465e-22 | -50.28 | ICHIBA\_GRAFT\_VERSUS\_HOST\_DISEASE\_D7\_UP | MSigDB lists | ICHIBA\_GRAFT\_VERSUS\_HOST\_DISEASE\_D7\_UP | 91 | 23 | 12187 | 179 | Il1rn,Ifi204,Ifi207,Col4a1,Vcam1,Ifit3b,Ifi209,Icam1,Nfkbia,Xdh,Irf7,Irgm2,Nfkbiz,Ptpn1,Tap1,Cd14,Isg15,Ccl12,Usp18,Msr1,Gbp3,Ifi211,Gbp7 | | 1.646e-22 | -50.16 | HALLMARK\_INTERFERON\_ALPHA\_RESPONSE | MSigDB lists | HALLMARK\_INTERFERON\_ALPHA\_RESPONSE | 80 | 22 | 12187 | 179 | Trim25,Parp9,Irf2,Irf9,Tap1,Ifitm3,Herc6,Ifitm2,Isg15,Samd9l,Ifi44,Ifit3b,Rtp4,Cmpk2,Gbp3,Irf7,Oasl1,Parp12,Ifit2,Il4ra,Parp14,Usp18 | | 1.796e-22 | -50.07 | GSE18791\_CTRL\_VS\_NEWCASTLE\_VIRUS\_DC\_4H\_DN | MSigDB lists | GSE18791\_CTRL\_VS\_NEWCASTLE\_VIRUS\_DC\_4H\_DN | 131 | 26 | 12187 | 179 | Dtx3l,Phf11d,Rtp4,Cmpk2,Ifit2,Usp18,Gadd45b,Rnf213,Herc6,Isg15,Tap1,Parp9,Phf11b,Irf7,Trim56,Tnfsf10,Nfkb2,Xaf1,Parp14,Oasl1,Trim25,Errfi1,Ifi44,Ifit3b,Samd9l,Znfx1 | | 1.870e-22 | -50.03 | SEKI\_INFLAMMATORY\_RESPONSE\_LPS\_UP | MSigDB lists | SEKI\_INFLAMMATORY\_RESPONSE\_LPS\_UP | 60 | 20 | 12187 | 179 | Cxcl1,Nfkbie,Nfkb2,Gbp3,Osmr,Nfkbia,Casp4,Maff,Birc3,Ccl12,Tnfaip3,Gadd45b,Tnip1,Cxcl16,Icam1,Sele,Vcam1,Nfkbiz,Ier3,Stx11 | | 2.978e-22 | -49.57 | MCLACHLAN\_DENTAL\_CARIES\_UP | MSigDB lists | MCLACHLAN\_DENTAL\_CARIES\_UP | 164 | 28 | 12187 | 179 | Msr1,Maff,Cybb,Il6,Tnfaip3,Ccl12,Bcl3,S100a8,Tnfaip8,Ms4a6d,Ier3,Cebpd,Cd14,Birc3,Trib1,C3ar1,C5ar1,S100a9,Socs3,Vcam1,Il1rn,Timp1,Ptgs2,Plek,Sele,Icam1,Csf2rb,Plaur | | 3.844e-22 | -49.31 | GSE35685\_CD34POS\_CD10NEG\_CD62LPOS\_VS\_CD34POS\_CD10POS\_BONE\_MARROW\_DN | MSigDB lists | GSE35685\_CD34POS\_CD10NEG\_CD62LPOS\_VS\_CD34POS\_CD10POS\_BONE\_MARROW\_DN | 182 | 29 | 12187 | 179 | Timp1,Csf2rb,Zfp36,Casp4,Lgals9,Slfn4,Irgm2,Ier3,Cebpd,Gadd45g,Nfkbiz,Hspa5,Ptpn1,Tap1,Slfn3,Ifitm3,Selp,Isg15,Ccl12,Ccl4,Ccl2,Map3k8,Ifit2,Pik3r1,Il4ra,Gbp3,Tnfaip8,Bcl3,Gbp7 | | 4.067e-22 | -49.25 | GSE9988\_LOW\_LPS\_VS\_VEHICLE\_TREATED\_MONOCYTE\_UP | MSigDB lists | GSE9988\_LOW\_LPS\_VS\_VEHICLE\_TREATED\_MONOCYTE\_UP | 135 | 26 | 12187 | 179 | Nfkbia,Sdc4,Birc3,Tnf,Gem,Cflar,Socs3,Tnip3,Ptgs2,Plaur,Errfi1,Plek,Icam1,Gpr84,Ccl4,Maff,Map3k8,Nfe2l2,Gadd45b,Tnfaip3,Cxcl1,Nfkbiz,Ier3,Il1a,Nfkb1,Pik3ap1 | | 4.210e-22 | -49.22 | GSE19198\_CTRL\_VS\_IL21\_TREATED\_TCELL\_24H\_UP | MSigDB lists | GSE19198\_CTRL\_VS\_IL21\_TREATED\_TCELL\_24H\_UP | 166 | 28 | 12187 | 179 | Tnf,Trib1,Xdh,Ifi44,Cdkn1a,Cp,Il1rn,Timp1,Atf3,Tnip1,Icam1,Errfi1,Maff,Ccl2,Tgm2,Ccl4,Nfe2l2,Il6,Gadd45b,Csf3,Rhoj,Cxcl1,Gbp3,Gadd45g,Akap12,Il1a,Map3k6,Ifitm3 | | 4.997e-22 | -49.05 | GSE34006\_WT\_VS\_A2AR\_KO\_TREG\_DN | MSigDB lists | GSE34006\_WT\_VS\_A2AR\_KO\_TREG\_DN | 167 | 28 | 12187 | 179 | Irf7,Gbp6,Irgm2,Tnfsf10,Slfn4,Parp14,Tor3a,Lgals9,Parp12,Oasl1,Gbp4,Irf9,Trim25,Ifit3b,Znfx1,Gbp7,Gbp3,Rtp4,Cmpk2,Usp18,Ccnd2,Ifit2,Ripk1,Slfn9,Isg15,Tap1,Parp9,Slfn3 | | 4.997e-22 | -49.05 | GSE5589\_LPS\_AND\_IL10\_VS\_LPS\_AND\_IL6\_STIM\_IL6\_KO\_MACROPHAGE\_45MIN\_UP | MSigDB lists | GSE5589\_LPS\_AND\_IL10\_VS\_LPS\_AND\_IL6\_STIM\_IL6\_KO\_MACROPHAGE\_45MIN\_UP | 167 | 28 | 12187 | 179 | Gbp6,Cflar,Tnfsf10,Xdh,Slfn4,Sdc4,Casp4,Tor3a,Parp14,Parp12,Gbp4,Irf9,Cdkn1a,Znfx1,Samd9l,Gbp7,Dtx3l,Ccl2,Msr1,Ccl12,Isg15,Ifitm3,Tap1,Cxcl16,Parp9,Pik3ap1,Slfn3,Ms4a6d | | 6.056e-22 | -48.86 | GSE21546\_ELK1\_KO\_VS\_SAP1A\_KO\_AND\_ELK1\_KO\_DP\_THYMOCYTES\_UP | MSigDB lists | GSE21546\_ELK1\_KO\_VS\_SAP1A\_KO\_AND\_ELK1\_KO\_DP\_THYMOCYTES\_UP | 137 | 26 | 12187 | 179 | Ifitm3,Herc6,Rnf213,Isg15,P2ry6,Parp9,Phf11b,Dtx3l,Phf11d,Ifi211,Adamts9,Cmpk2,Ifit2,Usp18,Ifitm2,Trim25,Irf9,Ifi209,Ifit3b,Ifi204,Ifi207,Samd9l,Irf7,Parp14,Oasl1,Parp12 | | 7.036e-22 | -48.71 | GSE13485\_PRE\_VS\_POST\_YF17D\_VACCINATION\_PBMC\_DN | MSigDB lists | GSE13485\_PRE\_VS\_POST\_YF17D\_VACCINATION\_PBMC\_DN | 153 | 27 | 12187 | 179 | Pik3ap1,Parp9,Tap1,Ifitm3,Herc6,Isg15,Ifit2,Msr1,Usp18,Rtp4,Cmpk2,Dtx3l,Ifi211,Ifi204,Samd9l,Ifi207,Ifi209,Ifi44,Ifit3b,Irf9,Ifitm2,Parp12,Oasl1,Xaf1,Parp14,C3ar1,Irf7 | | 1.034e-21 | -48.32 | MODULE\_84 | MSigDB lists | MODULE\_84 | 373 | 38 | 12187 | 179 | S100a9,A2m,C3ar1,Tnfsf10,Olfml2b,Igsf6,Parp14,Birc3,Tnf,Parp12,Plek,Icam1,Vcam1,Ifi44,Ifi209,Cdkn1a,Ifi207,Col4a1,Ifi204,Il1rn,Kcna5,Stx11,Ifi211,S100a8,Tnfaip8,Ccl4,Tgm2,Il4ra,Ccnd2,Il6,Tnfaip3,Ccl12,Cybb,Cd14,Isg15,Tap1,Cxcl16,Ier3 | | 1.316e-21 | -48.08 | GSE9988\_LOW\_LPS\_VS\_CTRL\_TREATED\_MONOCYTE\_UP | MSigDB lists | GSE9988\_LOW\_LPS\_VS\_CTRL\_TREATED\_MONOCYTE\_UP | 141 | 26 | 12187 | 179 | Nfkb1,Il1a,Ier3,Nfkbiz,Tnfaip3,Nfe2l2,Il6,Gadd45b,Map3k8,Maff,Ccl4,Gpr84,Csf3,Cxcl1,Tnip3,Socs3,Icam1,Plek,Errfi1,Plaur,Ptgs2,Tnf,Birc3,Sdc4,Nfkbia,Cflar | | 1.363e-21 | -48.04 | GSE32986\_CURDLAN\_HIGHDOSE\_VS\_GMCSF\_AND\_CURDLAN\_HIGHDOSE\_STIM\_DC\_DN | MSigDB lists | GSE32986\_CURDLAN\_HIGHDOSE\_VS\_GMCSF\_AND\_CURDLAN\_HIGHDOSE\_STIM\_DC\_DN | 173 | 28 | 12187 | 179 | Socs3,Stx11,Ptgs2,Tnip1,Zfp36,Icam1,Birc3,Tnf,Nfkbia,Sdc4,Casp4,Gem,Cflar,Slfn4,Nfkb2,Nfkbiz,Il1a,Nfkb1,Isg15,Slfn3,Maff,Gpr84,Ccl4,Rassf4,Gadd45b,Tnfaip3,Nfkbie,Bcl3 | | 1.363e-21 | -48.04 | GSE22601\_IMMATURE\_CD4\_SINGLE\_POSITIVE\_VS\_CD8\_SINGLE\_POSITIVE\_THYMOCYTE\_DN | MSigDB lists | GSE22601\_IMMATURE\_CD4\_SINGLE\_POSITIVE\_VS\_CD8\_SINGLE\_POSITIVE\_THYMOCYTE\_DN | 173 | 28 | 12187 | 179 | Tnfaip8,Rtp4,Dtx3l,Nfe2l2,Map3k8,Tgm2,Ptpn1,Selp,Ifitm3,Slfn9,Tiparp,Cebpd,Sgk1,Nfkbiz,Irgm2,Gbp6,Gem,Irf7,Gbp4,Birc3,Xaf1,Parp14,Casp4,Trim25,Zfp36,Irf9,Stx11,Ksr1 | | 1.656e-21 | -47.85 | innate immune response | biological process | GO:0045087 | 351 | 38 | 13711 | 214 | Oasl2,Casp4,Cybb,Ifitm3,Dtx3l,Oasl1,Irf7,Parp14,Ccl7,Iigp1,Mx1,Gbp4,Tgtp1,Parp9,Isg15,Irgm1,Ifitm6,Stx11,Mx2,Ccl4,Herc6,Trim56,Trim25,Acod1,Ccl2,Cd14,Lcn2,Trim30a,Gbp3,Ifitm2,S100a8,Cxcl16,Ccl12,S100a9,Gbp7,Gbp6,Irgm2,Ifit2 | | 1.934e-21 | -47.69 | cellular response to stimulus | biological process | GO:0051716 | 4061 | 130 | 13711 | 214 | Fzd4,Ifi209,Sbno2,Parp9,Tgtp1,Cmpk2,Spi1,Ccl4,Ifitm6,Stx11,Ier3,Sgk1,Ptgs2,Gem,Il1a,Casp4,Cdkn1a,Tgtp2,Il6,Trib1,Irf7,Socs3,Dtx3l,Pik3r1,Rrp8,Cxcl1,Dnajb1,Hp,Mt2,Ifit2,Csf2rb,Tnf,Zfp36,Saa1,Cflar,Cd14,Gpr84,Ripk1,Hspb1,Ifi207,Rhoc,Tnip1,Osmr,Gbp4,F830016B08Rik,Nfkb1,Kcna5,Bach1,Plaur,Il17ra,Errfi1,Cybb,Thbs1,Ifitm3,Gm4841,P2ry6,Gbp3,Ccl12,S100a8,Sgk3,Irgm2,Gbp6,Bcl3,Plek,Ksr1,Ifi47,Arid5b,Irak3,Ifi204,Fam107a,Map3k6,Tiparp,Isg15,Rasip1,Ifi211,Trim25,Runx1,Akap12,Nfkbia,Il4ra,Ip6k2,Gm5431,Ccl7,Ccnd2,Hcar2,Clic4,Hspa5,Tgm2,C5ar1,Rasd1,Nfe2l2,Gbp7,Selp,Msn,Angptl4,Pik3ap1,Acod1,Rhou,Atf3,Ncf1,Birc3,Map3k8,Rhoj,Irgm1,Pik3r5,Rgs16,Nfkb2,Fyb,Mt1,Icam1,Rassf4,Iigp1,Lgals9,Ifitm2,Ptpn1,Nfkbiz,Cxcl16,Tnip3,Col4a1,Iqgap1,S100a9,Vcam1,Cxcl9,Tnfaip3,Ccl2,Csf3,C3ar1,Il1rn,Lcn2,Retnlg | | 2.110e-21 | -47.61 | FOSTER\_TOLERANT\_MACROPHAGE\_DN | MSigDB lists | FOSTER\_TOLERANT\_MACROPHAGE\_DN | 357 | 37 | 12187 | 179 | Sgk3,Selp,Slfn5,Irf2,Nfkbiz,Gadd45g,Il1a,Nfkb1,Tiparp,Cebpd,Gbp7,Bcl3,Ifi211,Rgs16,Gbp3,Il4ra,Ccnd2,Map3k8,Gpr84,Il6,Tnfaip3,Ptgs2,Zfp36,Plaur,Errfi1,Ifi209,Cdkn1a,Aff1,Socs3,Vcam1,Cp,Ifi207,Ifi204,Tnfsf10,Sdc4,Parp14,Tubb6 | | 2.217e-21 | -47.56 | GSE14769\_UNSTIM\_VS\_40MIN\_LPS\_BMDM\_DN | MSigDB lists | GSE14769\_UNSTIM\_VS\_40MIN\_LPS\_BMDM\_DN | 176 | 28 | 12187 | 179 | Socs3,Atf3,Ptgs2,Zfp36,Errfi1,Tnf,Birc3,Sdc4,Nfkbia,Cflar,Slfn4,Sgk1,Nfkbiz,Tiparp,Ier3,Cd14,Slfn3,Map3k8,Maff,Ccl4,Ccl2,Gpr84,Usp18,Ccl12,Tnfaip3,Gadd45b,Nfkbie,Cxcl1 | | 2.217e-21 | -47.56 | GSE34006\_A2AR\_KO\_VS\_A2AR\_AGONIST\_TREATED\_TREG\_UP | MSigDB lists | GSE34006\_A2AR\_KO\_VS\_A2AR\_AGONIST\_TREATED\_TREG\_UP | 176 | 28 | 12187 | 179 | Slfn9,Isg15,Tap1,Parp9,Slfn3,Usp18,Ifit2,Ripk1,Gbp7,Gbp3,Rtp4,Cmpk2,Ifit3b,Ifi44,Znfx1,Irf9,Trim25,Parp14,Tor3a,Lgals9,Parp12,Oasl1,Gbp4,Irf7,Gbp6,Irgm2,Tnfsf10,Slfn4 | | 2.560e-21 | -47.41 | GO\_INNATE\_IMMUNE\_RESPONSE | MSigDB lists | GO\_INNATE\_IMMUNE\_RESPONSE | 336 | 36 | 12187 | 179 | Xaf1,Casp4,Gbp4,Oasl1,Irf7,S100a9,Trim56,Irgm2,Gbp6,Nfkb2,Vcam1,Ifit3b,Ifi209,Ifi204,Ifi207,Ncf1,Ifitm2,Saa1,Irf9,Trim25,Icam1,Ccl4,Ccl2,Ifit2,Ccl12,Cybb,Ifi211,S100a8,Lcn2,Nfkb1,Cd14,Ifitm3,Isg15,Irf2,Cxcl16,Ip6k2 | | 2.873e-21 | -47.30 | GSE19888\_CTRL\_VS\_TCELL\_MEMBRANES\_ACT\_MAST\_CELL\_PRETREAT\_A3R\_INH\_DN | MSigDB lists | GSE19888\_CTRL\_VS\_TCELL\_MEMBRANES\_ACT\_MAST\_CELL\_PRETREAT\_A3R\_INH\_DN | 161 | 27 | 12187 | 179 | Irf7,Parp14,Tor3a,Tubb6,AA467197,Trim25,Irf9,Clic4,Vcam1,Samd9l,Dtx3l,Gbp7,Bcl3,Pik3r5,Rtp4,Cmpk2,Rgs16,Ifit2,Usp18,Il6,Ccl12,Ifitm3,Slfn5,Slfn9,Pik3ap1,Parp9,Nfkb1 | | 2.873e-21 | -47.30 | GSE42021\_CD24HI\_VS\_CD24INT\_TREG\_THYMUS\_DN | MSigDB lists | GSE42021\_CD24HI\_VS\_CD24INT\_TREG\_THYMUS\_DN | 161 | 27 | 12187 | 179 | Ifitm2,Plaur,Irf9,Trim25,Zfp36,Ifit3b,Ifi209,Ifi44,Ifi204,Ifi207,Irf7,Tnfsf10,Nfkbia,Parp12,Oasl1,Bach1,Herc6,Isg15,Tap1,Phf11b,Cxcl1,Ifi211,Phf11d,Rtp4,Tgm2,Usp18,Ifit2 | | 2.961e-21 | -47.27 | cellular response to organic substance | biological process | GO:0071310 | 1457 | 74 | 13711 | 214 | Ptpn1,Ifitm2,Gbp3,Ccl12,Cxcl16,Tnip3,Iqgap1,Col4a1,Vcam1,Irgm2,Gbp6,Cxcl9,Tnfaip3,Ccl2,Ifi47,Csf3,Arid5b,Il1rn,Irak3,Ifi204,Birc3,Osmr,Gbp4,F830016B08Rik,Nfkb1,Irgm1,Il17ra,Icam1,Iigp1,Gm4841,Ifitm3,P2ry6,Hspa5,Cxcl1,Nfe2l2,Ifit2,Gbp7,Csf2rb,Selp,Msn,Tnf,Acod1,Atf3,Zfp36,Cd14,Ripk1,Hspb1,Ifi207,Fam107a,Fzd4,Tiparp,Ifi209,Sbno2,Tgtp1,Cmpk2,Ccl4,Spi1,Stx11,Ifitm6,Ifi211,Trim25,Sgk1,Runx1,Il1a,Nfkbia,Casp4,Il4ra,Tgtp2,Il6,Gm5431,Ccl7,Irf7,Socs3,Pik3r1 | | 4.502e-21 | -46.85 | GSE21546\_UNSTIM\_VS\_ANTI\_CD3\_STIM\_SAP1A\_KO\_AND\_ELK1\_KO\_DP\_THYMOCYTES\_UP | MSigDB lists | GSE21546\_UNSTIM\_VS\_ANTI\_CD3\_STIM\_SAP1A\_KO\_AND\_ELK1\_KO\_DP\_THYMOCYTES\_UP | 118 | 24 | 12187 | 179 | Oasl1,Parp12,Parp14,Usp18,Cmpk2,Phf11d,Ifi211,Irf7,C5ar1,Dtx3l,Ifi204,Ifi207,Ifit3b,Ifi209,Phf11b,Parp9,Irf9,Trim25,P2ry6,Ifitm3,Herc6,Ifitm2,Rnf213,Isg15 | | 4.817e-21 | -46.78 | GSE9988\_ANTI\_TREM1\_VS\_LPS\_MONOCYTE\_DN | MSigDB lists | GSE9988\_ANTI\_TREM1\_VS\_LPS\_MONOCYTE\_DN | 148 | 26 | 12187 | 179 | Socs3,Stx11,Tnip3,Ptgs2,Clic4,Icam1,Sdc4,Nfkbia,Birc3,Tnf,Oasl1,Cflar,Nfkbiz,Ier3,Il1a,Nfkb1,Pik3ap1,Ccl4,Map3k8,Rhou,Il6,Nfe2l2,Tnfaip3,Cxcl1,Csf3,Tnfaip8 | | 5.074e-21 | -46.73 | regulation of response to external stimulus | biological process | GO:0032101 | 697 | 51 | 13711 | 214 | Tap1,S100a8,A2m,Tnip3,Ccl12,Nfkbiz,Irgm2,Usp18,S100a9,Ccl2,Tnfaip3,Plek,Ifi204,Irak3,C3ar1,Nfkb1,Gbp4,Il17ra,Irgm1,Lgals9,Thbs1,Cxcl1,C5ar1,Tgm2,Selp,Nfe2l2,Mmp8,Zfp36,Acod1,Tnf,Pik3ap1,Tnip1,Trim30a,Hspb1,Cd14,Parp9,Sbno2,Ifi209,Ptgs2,Ier3,Ifi211,Ccl4,Il6,Cdkn1a,Nfkbia,Dtx3l,Irf7,Socs3,Ctla2a,Parp14,Trib1 | | 9.215e-21 | -46.13 | GSE26890\_CXCR1\_NEG\_VS\_POS\_EFFECTOR\_CD8\_TCELL\_UP | MSigDB lists | GSE26890\_CXCR1\_NEG\_VS\_POS\_EFFECTOR\_CD8\_TCELL\_UP | 168 | 27 | 12187 | 179 | Ifit2,Usp18,Cybb,Dtx3l,Gbp7,Cmpk2,Rtp4,Ifitm3,Rnf213,Isg15,Herc6,Slfn5,Slfn9,Parp9,Parp14,Tor3a,Xaf1,Gbp4,Parp12,Oasl1,Gbp6,Irf7,Xdh,Tnfsf10,Ifi44,Samd9l,Irf9 | | 1.279e-20 | -45.81 | TAKEDA\_TARGETS\_OF\_NUP98\_HOXA9\_FUSION\_3D\_UP | MSigDB lists | TAKEDA\_TARGETS\_OF\_NUP98\_HOXA9\_FUSION\_3D\_UP | 123 | 24 | 12187 | 179 | Ifi204,Samd9l,Ifi207,Ifi209,Ifi44,Ifit3b,Trim25,Irf9,Ptgs2,Oasl1,Parp12,Xaf1,Parp14,Irf7,Parp9,Isg15,Herc6,Rnf213,Ifit2,Usp18,Cmpk2,Dtx3l,Ifi211,Arid5b | | 2.285e-20 | -45.23 | GSE42021\_CD24INT\_VS\_CD24LOW\_TREG\_THYMUS\_DN | MSigDB lists | GSE42021\_CD24INT\_VS\_CD24LOW\_TREG\_THYMUS\_DN | 141 | 25 | 12187 | 179 | Ifitm3,Isg15,Herc6,Phf11b,Thbs1,Mmp8,Phf11d,Ifi211,Rtp4,Ifit2,Usp18,Ifitm2,Ifi209,Ifi44,Ifit3b,Ifi204,Ifi207,Cflar,Robo4,Irf7,Tnfsf10,Xaf1,Nfkbia,Parp12,Oasl1 | | 3.063e-20 | -44.93 | GO\_RESPONSE\_TO\_EXTERNAL\_STIMULUS | MSigDB lists | GO\_RESPONSE\_TO\_EXTERNAL\_STIMULUS | 1251 | 64 | 12187 | 179 | Hspa5,Tnfaip3,Ifit2,Il4ra,Ccl2,S100a8,Cxcl1,Ifi211,Bcl3,Ifi204,Hp,Cdkn1a,Ifi209,Fstl1,Trim25,Icam1,Irf9,Saa1,Oasl1,Trib1,Irf7,S100a9,Nfkb1,Il17ra,Irak3,Cxcl16,Ifitm3,Selp,Isg15,Cd14,Ccl12,Il6,Nfe2l2,Ccl4,Cmpk2,Sbno2,Csf3,Ptges,Rrp8,Tnip3,Kcna5,Cp,Ifi207,Atf3,Adamts1,Ifi44,Vcam1,Socs3,Ifit3b,Hspb1,Sele,Zfp36,Plaur,Ptgs2,Ifitm2,Gbp4,Tnf,Zfp189,Nfkbia,Nfkb2,C5ar1,C3ar1,Gbp6,Trim56 | | 3.620e-20 | -44.77 | REACTOME\_CYTOKINE\_SIGNALING\_IN\_IMMUNE\_SYSTEM | MSigDB lists | REACTOME\_CYTOKINE\_SIGNALING\_IN\_IMMUNE\_SYSTEM | 213 | 29 | 12187 | 179 | Ifitm2,Csf2rb,Irf9,Trim25,Icam1,Vcam1,Socs3,Ifit3b,Il1rn,Irf7,Gbp6,Nfkb2,Xaf1,Gbp4,Oasl1,Ifitm3,Isg15,Irf2,Ptpn1,Irak3,Ip6k2,Il1a,Gbp7,Gbp3,Usp18,Map3k8,Ifit2,Pik3r1,Il6 | | 3.803e-20 | -44.72 | ZWANG\_CLASS\_3\_TRANSIENTLY\_INDUCED\_BY\_EGF | MSigDB lists | ZWANG\_CLASS\_3\_TRANSIENTLY\_INDUCED\_BY\_EGF | 177 | 27 | 12187 | 179 | Tnfaip3,Il6,Map3k8,Maff,Sbno2,Pygm,Adamts9,Cxcl1,Bcl3,Cebpd,Ier3,Nfkb1,Tiparp,Il1a,Nfkbiz,Trib1,Runx1,Birc3,Nfkb2,Gem,Atf3,Il1rn,Stx11,Socs3,Aff1,Plaur,Ptgs2 | | 3.912e-20 | -44.69 | cellular response to interferon-beta | biological process | GO:0035458 | 36 | 16 | 13711 | 214 | Irgm1,Ifi211,Gbp6,Irgm2,Ifi209,Gbp3,Tgtp1,F830016B08Rik,Gm4841,Ifi207,Iigp1,Ifi47,Ifi204,Acod1,Gm5431,Tgtp2 | | 4.428e-20 | -44.56 | GSE42021\_TCONV\_PLN\_VS\_TREG\_PRECURSORS\_THYMUS\_DN | MSigDB lists | GSE42021\_TCONV\_PLN\_VS\_TREG\_PRECURSORS\_THYMUS\_DN | 178 | 27 | 12187 | 179 | Gbp7,Ptges,Angptl4,Cxcl1,Bcl3,Nfkb2,Map3k8,Ccnd2,Il4ra,Msr1,Birc3,Ccl2,Selp,Ptgs2,Cd14,Icam1,Errfi1,Plaur,Cdkn1a,Hspa5,Socs3,Nfkbiz,Il1rn,Nfkb1,Il1a,Cebpd,Lcn2 | | 5.198e-20 | -44.40 | MARKEY\_RB1\_ACUTE\_LOF\_DN | MSigDB lists | MARKEY\_RB1\_ACUTE\_LOF\_DN | 197 | 28 | 12187 | 179 | C5ar1,C3ar1,Irf7,Slfn4,Xdh,Tor3a,Casp4,Saa1,Irf9,Ifi209,Socs3,Ifit3b,Ifi204,Ifi207,Hp,Znfx1,Gbp7,Ifi211,Cxcl1,Iqgap1,Cmpk2,Gbp3,Ifit2,Ccl4,Usp18,Isg15,Slfn3,Tap1 | | 6.950e-20 | -44.11 | OSWALD\_HEMATOPOIETIC\_STEM\_CELL\_IN\_COLLAGEN\_GEL\_UP | MSigDB lists | OSWALD\_HEMATOPOIETIC\_STEM\_CELL\_IN\_COLLAGEN\_GEL\_UP | 181 | 27 | 12187 | 179 | Cflar,C3ar1,Trib1,Nfkbia,Runx1,Tnf,Birc3,Icam1,Zfp36,Ptgs2,Luc7l3,Atf3,Il1rn,Cdkn1a,Cxcl1,Ccl12,Tnfaip3,Gadd45b,Il6,Rassf4,Ccl4,Maff,Cd14,Ier3,Tiparp,Il1a,Sgk1 | | 7.884e-20 | -43.99 | GSE13485\_DAY1\_VS\_DAY7\_YF17D\_VACCINE\_PBMC\_DN | MSigDB lists | GSE13485\_DAY1\_VS\_DAY7\_YF17D\_VACCINE\_PBMC\_DN | 148 | 25 | 12187 | 179 | C3ar1,Irf7,Tnfsf10,Parp14,Xaf1,Parp12,Oasl1,Ifi44,Ifit3b,Il1rn,Samd9l,Dtx3l,Phf11d,Rtp4,Cmpk2,Gbp3,Ifit2,Usp18,Ifitm3,Herc6,Isg15,Pik3ap1,Parp9,Tap1,Phf11b | | 8.941e-20 | -43.86 | GSE9988\_ANTI\_TREM1\_VS\_ANTI\_TREM1\_AND\_LPS\_MONOCYTE\_DN | MSigDB lists | GSE9988\_ANTI\_TREM1\_VS\_ANTI\_TREM1\_AND\_LPS\_MONOCYTE\_DN | 133 | 24 | 12187 | 179 | Tnfaip8,Csf3,Cxcl1,Il6,Tnfaip3,Map3k8,Gpr84,Ccl4,Il1a,Ier3,Nfkbiz,Cflar,Birc3,Tnf,Sdc4,Nfkbia,Plek,Zfp36,Icam1,Plaur,Clic4,Ptgs2,Tnip3,Socs3 | | 9.279e-20 | -43.82 | response to virus | biological process | GO:0009615 | 171 | 27 | 13711 | 214 | Lcn2,Trim30a,Irak3,Cxcl9,Tnf,Irgm2,Ifit2,Bcl3,Ifit3b,Irf2,Ifitm2,Lgals9,Mx1,Dtx3l,Ifitm3,Irf7,Oasl1,Il6,Oasl2,Slfn9,Trim25,Trim56,Mx2,Rtp4,Ifitm6,Parp9,Isg15 | | 9.349e-20 | -43.82 | MODULE\_75 | MSigDB lists | MODULE\_75 | 240 | 30 | 12187 | 179 | Nfkbia,Oasl1,Gem,C3ar1,C5ar1,Irf7,Igsf6,Tnfsf10,Ifi44,Cdkn1a,Hp,Timp1,Ifitm2,Saa1,Csf2rb,Il4ra,Map3k8,Ccl2,Ccl4,Cybb,Il6,Ccl12,Csf3,Cxcl1,Osmr,Il1a,Isg15,Ifitm3,Selp,Cd14 | | 1.382e-19 | -43.43 | FULCHER\_INFLAMMATORY\_RESPONSE\_LECTIN\_VS\_LPS\_DN | MSigDB lists | FULCHER\_INFLAMMATORY\_RESPONSE\_LECTIN\_VS\_LPS\_DN | 354 | 35 | 12187 | 179 | Sgk1,Hspa5,Tiparp,Ms4a6d,Cebpd,Ifitm3,Herc6,Rnf213,Slfn5,Cd14,Pik3ap1,Map3k8,Ifit2,Usp18,Gadd45b,Ifi211,Arid5b,Cmpk2,Ifi209,Aff1,Ifi44,Socs3,Ifit3b,Ifi204,Ifi207,Samd9l,Ifitm2,Zfp36,Rbm47,Xaf1,Bach1,Oasl1,C3ar1,Olfml2b,Tnfsf10 | | 1.592e-19 | -43.28 | GSE8384\_CTRL\_VS\_B\_ABORTUS\_4H\_MAC\_CELL\_LINE\_DN | MSigDB lists | GSE8384\_CTRL\_VS\_B\_ABORTUS\_4H\_MAC\_CELL\_LINE\_DN | 169 | 26 | 12187 | 179 | Slfn3,Nfkbiz,Il1a,Ier3,Csf3,Bcl3,Sntb2,Ccl2,Ccl4,Cybb,Gadd45b,Ptgs2,Tnip1,Zfp36,Msn,Plaur,Cdkn1a,Aff1,Socs3,Il1rn,Znfx1,C5ar1,Slfn4,Nfkb2,Tnf,Nfkbia | | 1.761e-19 | -43.18 | GO\_CELLULAR\_RESPONSE\_TO\_CYTOKINE\_STIMULUS | MSigDB lists | GO\_CELLULAR\_RESPONSE\_TO\_CYTOKINE\_STIMULUS | 406 | 37 | 12187 | 179 | Csf3,Cxcl1,Osmr,Sbno2,Il4ra,Ifit2,Ccl2,Ccl4,Nfe2l2,Il6,Ccl12,Ripk1,Isg15,Ifitm3,Ip6k2,Il17ra,Irak3,Irf2,Hspa5,Il1a,Nfkb1,Gbp6,Irf7,Birc3,Tnf,Nfkbia,Xaf1,Oasl1,Gbp4,Ifitm2,Icam1,Trim25,Irf9,Csf2rb,Ifit3b,Socs3,Vcam1 | | 1.808e-19 | -43.16 | NUYTTEN\_EZH2\_TARGETS\_UP | MSigDB lists | NUYTTEN\_EZH2\_TARGETS\_UP | 853 | 52 | 12187 | 179 | Ifi44,Ifi207,Atf3,Ptgs2,Errfi1,Birc3,Xaf1,Nfkbia,Parp12,Gem,Robo4,Phf11b,Thbs1,Herc6,Parp9,Tap1,Tgm2,Rhoc,Il6,Nfkbie,Angptl4,Phf11d,Rtp4,Gbp3,Cdkn1a,Ifi209,Ifi204,Samd9l,Znfx1,Saa1,Trim25,Icam1,Clic4,Parp14,Bach1,AA467197,Oasl1,Cflar,Tnfsf10,Hspa5,Sgk1,Slfn5,Ptpn1,Irf2,Rhou,Usp18,Tnfaip3,Dtx3l,Cxcl1,Ifi211,Arid5b,Tnfaip8 | | 1.856e-19 | -43.13 | GSE14000\_UNSTIM\_VS\_4H\_LPS\_DC\_TRANSLATED\_RNA\_DN | MSigDB lists | GSE14000\_UNSTIM\_VS\_4H\_LPS\_DC\_TRANSLATED\_RNA\_DN | 137 | 24 | 12187 | 179 | Tnfsf10,Trim56,Irf7,Cflar,Oasl1,Xaf1,Birc3,Errfi1,Atf3,Stx11,Socs3,Ifit3b,Ifi44,Rtp4,Cmpk2,Dtx3l,Ccl4,Usp18,Ifit2,Parp9,Slfn5,Herc6,Isg15,Nfkbiz | | 1.856e-19 | -43.13 | GSE9988\_LPS\_VS\_VEHICLE\_TREATED\_MONOCYTE\_UP | MSigDB lists | GSE9988\_LPS\_VS\_VEHICLE\_TREATED\_MONOCYTE\_UP | 137 | 24 | 12187 | 179 | Nfkbiz,Nfkb1,Il1a,Ier3,Pik3ap1,Map3k8,Maff,Ccl4,Gpr84,Tnfaip3,Gadd45b,Il6,Cxcl1,Socs3,Tnip3,Ptgs2,Icam1,Plek,Plaur,Tnf,Birc3,Nfkbia,Cflar,Gem | | 2.545e-19 | -42.82 | GSE2770\_TGFB\_AND\_IL4\_ACT\_VS\_ACT\_CD4\_TCELL\_2H\_DN | MSigDB lists | GSE2770\_TGFB\_AND\_IL4\_ACT\_VS\_ACT\_CD4\_TCELL\_2H\_DN | 155 | 25 | 12187 | 179 | Tnfsf10,Irf7,Trim56,Parp12,Oasl1,Xaf1,Trim25,Irf9,Ifitm2,Ifi204,Ifi207,Samd9l,Znfx1,Ifi209,Ifi44,Dtx3l,Phf11d,Ifi211,Ifit2,Irf2,Parp9,Ifitm3,Isg15,Tiparp,Phf11b | | 2.926e-19 | -42.68 | GSE34156\_NOD2\_LIGAND\_VS\_TLR1\_TLR2\_LIGAND\_6H\_TREATED\_MONOCYTE\_UP | MSigDB lists | GSE34156\_NOD2\_LIGAND\_VS\_TLR1\_TLR2\_LIGAND\_6H\_TREATED\_MONOCYTE\_UP | 173 | 26 | 12187 | 179 | Irf7,Irgm2,Xdh,Tnfsf10,Slfn4,Tor3a,Casp4,Lgals9,Clic4,Irf9,Trim25,Zfp36,Ifit3b,Znfx1,Gbp7,Gbp3,Tnfaip8,Osmr,Usp18,Ifit2,Il6,Ifitm3,Isg15,Tap1,Slfn3,Gadd45g | | 3.942e-19 | -42.38 | GSE19198\_1H\_VS\_6H\_IL21\_TREATED\_TCELL\_DN | MSigDB lists | GSE19198\_1H\_VS\_6H\_IL21\_TREATED\_TCELL\_DN | 175 | 26 | 12187 | 179 | Tiparp,Akap12,Il1a,Thbs1,Tap1,Ifitm3,Map3k6,Il6,Hcar2,Ccl4,Ccnd2,Maff,Ptges,Rhoj,Timp1,Hp,Socs3,Ifit3b,Cdkn1a,Clic4,Irf9,Tubb6,Bach1,Parp14,Casp4,Irf7 | | 3.966e-19 | -42.37 | regulation of defense response | biological process | GO:0031347 | 460 | 40 | 13711 | 214 | Tap1,S100a8,A2m,Tnip3,Cxcl1,C5ar1,Tgm2,Nfkbiz,Irgm2,S100a9,Mmp8,Usp18,Zfp36,Tnf,Tnfaip3,Acod1,Pik3ap1,Tnip1,Ifi204,Trim30a,Irak3,Cd14,Nfkb1,Parp9,Sbno2,Ifi209,Gbp4,Ptgs2,Il17ra,Ier3,Ifi211,Irgm1,Il6,Nfkbia,Lgals9,Dtx3l,Irf7,Socs3,Ctla2a,Parp14 | | 4.836e-19 | -42.17 | GSE37533\_PPARG1\_FOXP3\_VS\_PPARG2\_FOXP3\_TRANSDUCED\_CD4\_TCELL\_PIOGLITAZONE\_TREATED\_DN | MSigDB lists | GSE37533\_PPARG1\_FOXP3\_VS\_PPARG2\_FOXP3\_TRANSDUCED\_CD4\_TCELL\_PIOGLITAZONE\_TREATED\_DN | 159 | 25 | 12187 | 179 | Trib1,Xaf1,Parp12,Bach1,Ifit3b,Socs3,Ifi44,Aff1,Atf3,Irf9,Sntb2,Usp18,Ifit2,Ripk1,Fam107a,Bcl3,Phf11d,Sbno2,Rtp4,Dnajb1,Phf11b,Herc6,Isg15,Tap1,Irf2 | | 5.302e-19 | -42.08 | GSE42724\_NAIVE\_BCELL\_VS\_PLASMABLAST\_UP | MSigDB lists | GSE42724\_NAIVE\_BCELL\_VS\_PLASMABLAST\_UP | 143 | 24 | 12187 | 179 | Dtx3l,Irf7,Rtp4,Cmpk2,Tnfsf10,Gbp3,Ifit2,Usp18,Parp14,Xaf1,Oasl1,Parp12,Ripk1,AA467197,Herc6,Isg15,Trim25,Tap1,Irf9,Parp9,Ifi44,Ifit3b,Samd9l,Znfx1 | | 7.437e-19 | -41.74 | GSE14000\_UNSTIM\_VS\_4H\_LPS\_DC\_DN | MSigDB lists | GSE14000\_UNSTIM\_VS\_4H\_LPS\_DC\_DN | 145 | 24 | 12187 | 179 | Tnfsf10,Irf7,Cflar,Oasl1,Nfkbia,Xaf1,Parp14,Zfp189,Irf9,Plek,Atf3,Znfx1,Ifit3b,Ifi44,Rtp4,Cmpk2,Dtx3l,Usp18,Ccl4,Ifit2,Parp9,Irf2,Isg15,Nfkbiz | | 1.273e-18 | -41.20 | regulation of immune system process | biological process | GO:0002682 | 953 | 56 | 13711 | 214 | Irgm2,Vcam1,Ccl12,A2m,Tnip3,Tap1,Nfkbiz,Ifi204,Zbtb16,Csf3,C3ar1,Irak3,Ccl2,Tnfaip3,Irgm1,Gbp4,Lgals9,Thbs1,Sox11,Fyb,Icam1,Selp,Msn,Mmp8,Nfe2l2,Cxcl1,C5ar1,Trim30a,Tnip1,Cd14,Ripk1,Sele,Zfp36,Pik3ap1,Tnf,Acod1,Runx1,Spi1,Ccl4,Ifi211,Isg15,Sdc4,Parp9,Ifi209,Hcar2,Pik3r1,Ctla2a,Trib1,Parp14,Irf7,Dtx3l,Cdkn1a,Il6,Il1a,Nfkbia,Il4ra | | 2.119e-18 | -40.70 | GSE9988\_LPS\_VS\_CTRL\_TREATED\_MONOCYTE\_UP | MSigDB lists | GSE9988\_LPS\_VS\_CTRL\_TREATED\_MONOCYTE\_UP | 135 | 23 | 12187 | 179 | Il1a,Ier3,Nfkbiz,Il6,Gadd45b,Tnfaip3,Maff,Map3k8,Gpr84,Ccl4,Csf3,Cxcl1,Tnip3,Cdkn1a,Socs3,Plek,Icam1,Plaur,Ptgs2,Birc3,Tnf,Nfkbia,Cflar | | 2.332e-18 | -40.60 | GSE42021\_TREG\_PLN\_VS\_TREG\_PRECURSORS\_THYMUS\_DN | MSigDB lists | GSE42021\_TREG\_PLN\_VS\_TREG\_PRECURSORS\_THYMUS\_DN | 152 | 24 | 12187 | 179 | Ifitm2,Irf9,Trim25,Ifit3b,Ifi44,Ifi209,Ifi207,Ifi204,Irf7,Tnfsf10,Xaf1,Parp12,Oasl1,Isg15,Herc6,Ifitm3,Tap1,Phf11b,Ifi211,Phf11d,Osmr,Usp18,Maff,Ifit2 | | 2.453e-18 | -40.55 | GRAESSMANN\_APOPTOSIS\_BY\_SERUM\_DEPRIVATION\_UP | MSigDB lists | GRAESSMANN\_APOPTOSIS\_BY\_SERUM\_DEPRIVATION\_UP | 439 | 37 | 12187 | 179 | Cebpd,Sgk1,Slfn3,Cxcl16,Irf2,Parp9,Isg15,Slfn9,Gadd45b,Ccl12,Ifit2,Usp18,Osmr,Cmpk2,Rtp4,Gbp7,Cxcl1,Ifi211,Cp,Ifi207,Ifi204,Ifi44,Ifi209,Ifit3b,Fstl1,Trim25,Hspb1,Irf9,Saa1,Parp12,Oasl1,Runx1,Tor3a,Slfn4,Xdh,Irgm2,Irf7 | | 2.732e-18 | -40.44 | GSE22886\_CTRL\_VS\_LPS\_24H\_DC\_DN | MSigDB lists | GSE22886\_CTRL\_VS\_LPS\_24H\_DC\_DN | 153 | 24 | 12187 | 179 | Ifitm3,Herc6,Ifitm2,Isg15,Tnip1,Icam1,Tap1,Irf9,Phf11b,Ifi44,Ifit3b,Nfkb1,Phf11d,Irf7,Tnfaip8,Rtp4,Nfkb2,Tnfsf10,Ifit2,Birc3,Xaf1,Usp18,Tnfaip3,Oasl1 | | 5.092e-18 | -39.82 | MODULE\_46 | MSigDB lists | MODULE\_46 | 233 | 28 | 12187 | 179 | Osmr,Cxcl1,Csf3,Ccl12,Il6,Cybb,Ccl4,Ccl2,Il4ra,Cd14,Selp,Ifitm3,Isg15,Il1a,Tnfsf10,Igsf6,Irf7,C5ar1,Gem,C3ar1,Oasl1,Nfkbia,Csf2rb,Ifitm2,Saa1,Timp1,Hp,Ifi44 | | 6.436e-18 | -39.58 | inflammatory response | biological process | GO:0006954 | 302 | 32 | 13711 | 214 | Il1a,Casp4,Icam1,Il4ra,Il6,Cybb,Ccl7,Thbs1,Timp1,Ccl4,Ptgs2,Cxcl9,Tnf,Acod1,Tnfaip3,Ccl2,Saa1,Cd14,Sele,Il1rn,C3ar1,Ncf1,Tnip1,Nfkbiz,Cxcl1,C5ar1,Ccl12,S100a8,Hp,S100a9,Nfe2l2,Selp | | 6.994e-18 | -39.50 | GSE42021\_TREG\_PLN\_VS\_CD24INT\_TREG\_THYMUS\_UP | MSigDB lists | GSE42021\_TREG\_PLN\_VS\_CD24INT\_TREG\_THYMUS\_UP | 177 | 25 | 12187 | 179 | Socs3,Cdkn1a,Il1rn,Ptgs2,Errfi1,Plaur,Icam1,Birc3,Nfkbiz,Cebpd,Lcn2,Il1a,Nfkb1,Cd14,Selp,Sntb2,Ccl2,Il4ra,Msr1,Map3k8,Ccnd2,Gadd45b,Cxcl1,Ptges,Gbp7 | | 7.200e-18 | -39.47 | GO\_RESPONSE\_TO\_BACTERIUM | MSigDB lists | GO\_RESPONSE\_TO\_BACTERIUM | 302 | 31 | 12187 | 179 | Nfkb1,Isg15,Selp,Cd14,Irak3,Cxcl16,Il6,Tnfaip3,Ccl12,Csf3,Bcl3,Ptges,Cxcl1,Cmpk2,S100a8,Sbno2,Vcam1,Socs3,Hp,Tnip3,Ptgs2,Sele,Icam1,Tnf,Trib1,Nfkbia,Gbp4,Gbp6,C5ar1,S100a9,Nfkb2 | | 8.611e-18 | -39.29 | regulation of response to stress | biological process | GO:0080134 | 1063 | 58 | 13711 | 214 | Tnfaip3,Plek,Xdh,Ccl2,Il1rn,Irak3,Ifi204,Nfkbiz,Ptpn1,S100a8,Tap1,A2m,Tnip3,S100a9,Usp18,Irgm2,Errfi1,Thbs1,Gadd45g,Lgals9,Gbp4,Nfkb1,Irgm1,Il17ra,Acod1,Tnf,Pik3ap1,Zfp36,Ncf1,Hspb1,Ripk1,Cd14,Tnip1,Trim30a,Cxcl1,C5ar1,Tgm2,Nfe2l2,Mmp8,Selp,Nfkbia,Il1a,Il6,Cdkn1a,Dtx3l,Socs3,Irf7,Parp14,Ctla2a,Gadd45b,Pik3r1,Ifi209,Fzd4,Parp9,Sbno2,Ifi211,Ptgs2,Ier3 | | 8.819e-18 | -39.27 | GSE36891\_POLYIC\_TLR3\_VS\_PAM\_TLR2\_STIM\_PERITONEAL\_MACROPHAGE\_UP | MSigDB lists | GSE36891\_POLYIC\_TLR3\_VS\_PAM\_TLR2\_STIM\_PERITONEAL\_MACROPHAGE\_UP | 112 | 21 | 12187 | 179 | Maff,Birc3,Rhou,Sdc4,Trib1,Il6,Ccl12,Tnfaip3,Gem,Cxcl1,Sgk1,Adamts1,Nfkbiz,Il1a,Akap12,Atf3,Ier3,Cebpd,Ptgs2,Zfp36,Apold1 | | 1.414e-17 | -38.80 | GO\_CYTOKINE\_MEDIATED\_SIGNALING\_PATHWAY | MSigDB lists | GO\_CYTOKINE\_MEDIATED\_SIGNALING\_PATHWAY | 286 | 30 | 12187 | 179 | Isg15,Ifitm3,Ip6k2,Irak3,Il17ra,Irf2,Il1a,Csf3,Cxcl1,Osmr,Il4ra,Ifit2,Ccl2,Ccl4,Il6,Ripk1,Ccl12,Ifitm2,Trim25,Icam1,Irf9,Csf2rb,Ifit3b,Vcam1,Socs3,Irf7,Birc3,Tnf,Xaf1,Oasl1 | | 1.536e-17 | -38.71 | GSE13485\_CTRL\_VS\_DAY3\_YF17D\_VACCINE\_PBMC\_DN | MSigDB lists | GSE13485\_CTRL\_VS\_DAY3\_YF17D\_VACCINE\_PBMC\_DN | 147 | 23 | 12187 | 179 | C3ar1,Irf7,Oasl1,Parp12,Xaf1,Parp14,Ifitm2,Samd9l,Ifi44,Ifit3b,Cmpk2,Rtp4,Dtx3l,Phf11d,Ifit2,Usp18,Pik3ap1,Parp9,Tap1,Ifitm3,Herc6,Isg15,Phf11b | | 2.221e-17 | -38.35 | GSE3982\_CTRL\_VS\_LPS\_4H\_MAC\_DN | MSigDB lists | GSE3982\_CTRL\_VS\_LPS\_4H\_MAC\_DN | 167 | 24 | 12187 | 179 | Oasl1,Tubb6,Bach1,Nfkb2,Cflar,Il1rn,Ifi204,Ifi207,Ifi209,Ifi44,Clic4,Trim25,Plek,Tnfaip3,Gadd45b,Usp18,Ifit2,Maff,Ifi211,Pik3r5,Arid5b,Cebpd,Tiparp,Irf2 | | 2.559e-17 | -38.20 | GSE14769\_UNSTIM\_VS\_80MIN\_LPS\_BMDM\_DN | MSigDB lists | GSE14769\_UNSTIM\_VS\_80MIN\_LPS\_BMDM\_DN | 168 | 24 | 12187 | 179 | Nfkbie,Usp18,Gpr84,Ccl2,Tgm2,Ccl4,Maff,Map3k8,Gadd45b,Isg15,Slfn3,Tiparp,Cflar,Rbm39,Slfn4,Birc3,Plaur,Plek,Sele,Icam1,Adamts1,Atf3,Stx11,Il1rn | | 2.603e-17 | -38.19 | REACTOME\_IMMUNE\_SYSTEM | MSigDB lists | REACTOME\_IMMUNE\_SYSTEM | 682 | 44 | 12187 | 179 | Tap1,Irf2,Ptpn1,Ip6k2,Pik3ap1,Irak3,Cd14,Isg15,Ifitm3,Il1a,Hspa5,Gbp3,Gbp7,Dtx3l,Nfkbie,Il6,Tnfaip3,Ripk1,Cybb,Usp18,Pik3r1,Map3k8,Ifit2,Irf9,Csf2rb,Trim25,Icam1,Saa1,Ifitm2,Il1rn,Ifit3b,Zbtb16,Socs3,Vcam1,Cdkn1a,Nfkb2,Irf7,Gbp6,Oasl1,Gbp4,Casp4,Nfkbia,Xaf1,Birc3 | | 3.271e-17 | -37.96 | REACTOME\_INTERFERON\_SIGNALING | MSigDB lists | REACTOME\_INTERFERON\_SIGNALING | 119 | 21 | 12187 | 179 | Ifitm2,Isg15,Ifitm3,Ip6k2,Trim25,Icam1,Irf9,Ptpn1,Irf2,Ifit3b,Vcam1,Socs3,Gbp7,Gbp6,Irf7,Gbp3,Ifit2,Usp18,Xaf1,Oasl1,Gbp4 | | 3.388e-17 | -37.92 | GSE14769\_UNSTIM\_VS\_60MIN\_LPS\_BMDM\_DN | MSigDB lists | GSE14769\_UNSTIM\_VS\_60MIN\_LPS\_BMDM\_DN | 170 | 24 | 12187 | 179 | Nfkbie,Gadd45b,Map3k8,Ccl4,Ccl2,Gpr84,Slfn3,Cd14,Tiparp,Ier3,Sgk1,Slfn4,Rbm39,Cflar,Gem,Birc3,Icam1,Plek,Sele,Plaur,Errfi1,Ptgs2,Stx11,Atf3 | | 3.499e-17 | -37.89 | WALLACE\_PROSTATE\_CANCER\_RACE\_UP | MSigDB lists | WALLACE\_PROSTATE\_CANCER\_RACE\_UP | 189 | 25 | 12187 | 179 | C5ar1,Xaf1,Parp12,Ifitm2,Irf9,Csf2rb,Sele,Plek,Apold1,Ifit3b,Ifi44,Ifi209,Adamts1,Ifi207,Ifi204,Ifi211,Rgs16,Ccl4,Herc6,Isg15,Selp,Ifitm3,Tap1,Cebpd,Gadd45g | | 4.307e-17 | -37.68 | HECKER\_IFNB1\_TARGETS | MSigDB lists | HECKER\_IFNB1\_TARGETS | 55 | 16 | 12187 | 179 | Cmpk2,Rtp4,Dtx3l,C3ar1,Irf7,Ccl12,Parp12,Oasl1,Ifit2,Xaf1,Parp9,Isg15,Herc6,Samd9l,Ifi44,Ifit3b | | 4.477e-17 | -37.64 | GSE43863\_TFH\_VS\_LY6C\_INT\_CXCR5POS\_MEMORY\_CD4\_TCELL\_UP | MSigDB lists | GSE43863\_TFH\_VS\_LY6C\_INT\_CXCR5POS\_MEMORY\_CD4\_TCELL\_UP | 154 | 23 | 12187 | 179 | Socs3,Hspa5,Ifi44,Atf3,Il1rn,Slfn9,Isg15,Ifitm3,Tap1,Icam1,Nfkbia,Usp18,Xaf1,Parp14,Lgals9,Il6,Ripk1,Gbp4,Ptges,Dtx3l,Gbp6,Irgm2,Rtp4 | | 4.948e-17 | -37.54 | GO\_REGULATION\_OF\_RESPONSE\_TO\_STRESS | MSigDB lists | GO\_REGULATION\_OF\_RESPONSE\_TO\_STRESS | 1121 | 56 | 12187 | 179 | Saa1,Plek,Ifi209,Hp,Ifi204,Fzd4,S100a9,Irf7,Casp4,Map3k6,Ptpn1,Hspa5,Ier3,Ifi211,S100a8,Pik3r1,Ccl2,Usp18,Gadd45b,Ripk1,Tnfaip3,Ptgs2,Tnip1,Sele,Hspb1,Plaur,Errfi1,Socs3,Ifi207,Tnip3,C3ar1,C5ar1,A2m,Xdh,Birc3,Zfp189,Tnf,Nfkbia,Selp,Cd14,Pik3ap1,Irak3,Il17ra,Tap1,Parp9,Thbs1,Dnajb1,Il1a,Gadd45g,Nfkb1,Osmr,Sbno2,Ccl4,Il6,Nfe2l2,Ccl12 | | 5.161e-17 | -37.50 | response to molecule of bacterial origin | biological process | GO:0002237 | 198 | 26 | 13711 | 214 | C5ar1,Cxcl1,Ccl12,Cxcl16,Tnip3,Irgm2,Wfdc21,Gbp6,Noct,Cxcl9,Tnfaip3,Tnf,Acod1,Ccl2,Zfp36,Cd14,Irak3,Il1rn,Sbno2,Nfkb1,Cmpk2,Ptgs2,Nfkbia,Il6,Trib1,Lgals9 | | 5.192e-17 | -37.50 | HALLMARK\_INFLAMMATORY\_RESPONSE | MSigDB lists | HALLMARK\_INFLAMMATORY\_RESPONSE | 155 | 23 | 12187 | 179 | Sele,Icam1,Plaur,Timp1,Cdkn1a,Tnfsf10,C3ar1,C5ar1,Irf7,Nfkbia,Cd14,Il1a,Nfkb1,Osmr,Rtp4,Rgs16,Csf3,Pik3r5,Cybb,Il6,Ccl12,Msr1,Il4ra | | 5.343e-17 | -37.47 | GSE18791\_CTRL\_VS\_NEWCASTLE\_VIRUS\_DC\_12H\_DN | MSigDB lists | GSE18791\_CTRL\_VS\_NEWCASTLE\_VIRUS\_DC\_12H\_DN | 138 | 22 | 12187 | 179 | Ifit2,Map3k8,Il6,Dtx3l,Cmpk2,Rtp4,Gbp3,Il1a,Isg15,Rnf213,Herc6,Slfn5,Tap1,Tnf,Parp14,Xaf1,Irf7,Ifi44,Ifit3b,Samd9l,Znfx1,Trim25 | | 5.870e-17 | -37.37 | GSE27434\_WT\_VS\_DNMT1\_KO\_TREG\_DN | MSigDB lists | GSE27434\_WT\_VS\_DNMT1\_KO\_TREG\_DN | 174 | 24 | 12187 | 179 | Tnf,Birc3,Nfkbia,Casp4,Icam1,Zfp36,Errfi1,Plaur,Ptgs2,Il1rn,Cdkn1a,Socs3,Tnfaip8,Cxcl1,Nfe2l2,Gadd45b,Map3k8,Il4ra,Ccl2,Selp,Cd14,Il1a,Sgk1,Nfkbiz | | 5.957e-17 | -37.36 | TNF signaling pathway | KEGG pathways | ko04668 | 94 | 21 | 5248 | 107 | Ifi47,Map3k8,Ccl2,Pik3r1,Cflar,Nfkbia,Sele,Cxcl1,Ccl12,Tnfaip3,Birc3,Icam1,Tnf,Vcam1,Ripk1,Socs3,Il6,Nfkb1,Gm5431,Ptgs2,Bcl3 | | 5.957e-17 | -37.36 | TNF signaling pathway | KEGG pathways | mmu04668 | 94 | 21 | 5248 | 107 | Vcam1,Ripk1,Birc3,Tnf,Icam1,Nfkb1,Socs3,Il6,Gm5431,Bcl3,Ptgs2,Cflar,Ccl2,Pik3r1,Ifi47,Map3k8,Nfkbia,Sele,Cxcl1,Ccl12,Tnfaip3 | | 8.192e-17 | -37.04 | response to lipopolysaccharide | biological process | GO:0032496 | 183 | 25 | 13711 | 214 | Nfkbia,Il6,Trib1,Lgals9,Nfkb1,Cmpk2,Sbno2,Ptgs2,Tnfaip3,Tnf,Acod1,Cxcl9,Zfp36,Ccl2,Il1rn,Irak3,Cd14,Cxcl1,Tnip3,Ccl12,Cxcl16,Noct,Wfdc21,Irgm2,Gbp6 | | 9.197e-17 | -36.93 | MOSERLE\_IFNA\_RESPONSE | MSigDB lists | MOSERLE\_IFNA\_RESPONSE | 30 | 13 | 12187 | 179 | Ifi204,Ifi207,Samd9l,Ifi209,Ifi44,Ifit3b,Cmpk2,Rtp4,Tnfsf10,Ifi211,Oasl1,Ifit2,Usp18 | | 9.459e-17 | -36.90 | ZHOU\_INFLAMMATORY\_RESPONSE\_LPS\_UP | MSigDB lists | ZHOU\_INFLAMMATORY\_RESPONSE\_LPS\_UP | 260 | 28 | 12187 | 179 | Ccl4,Usp18,Gpr84,Ifit2,Maff,Ripk1,Il6,Cxcl1,Arid5b,Csf3,Rtp4,Ier3,Nfkb1,Slfn5,Isg15,Ptpn1,Sdc4,Tnf,Oasl1,Irf7,Fzd4,Nfkb2,Ifit3b,Ifi44,Znfx1,Ptgs2,Plaur,Icam1 | | 1.562e-16 | -36.40 | GO\_REGULATION\_OF\_RESPONSE\_TO\_WOUNDING | MSigDB lists | GO\_REGULATION\_OF\_RESPONSE\_TO\_WOUNDING | 265 | 28 | 12187 | 179 | C3ar1,C5ar1,A2m,S100a9,Birc3,Tnf,Casp4,Plek,Sele,Plaur,Ptgs2,Saa1,Tnip1,Socs3,S100a8,Osmr,Sbno2,Nfe2l2,Il6,Tnfaip3,Ccl12,Ccl2,Ccl4,Pik3ap1,Il17ra,Selp,Nfkb1,Thbs1 | | 1.634e-16 | -36.35 | GSE37533\_PPARG1\_FOXP3\_VS\_FOXP3\_TRANSDUCED\_CD4\_TCELL\_DN | MSigDB lists | GSE37533\_PPARG1\_FOXP3\_VS\_FOXP3\_TRANSDUCED\_CD4\_TCELL\_DN | 163 | 23 | 12187 | 179 | Phf11b,Tap1,Irf2,Herc6,Isg15,Ripk1,Usp18,Ifit2,Rtp4,Phf11d,Ifi211,Ifi207,Ifi204,Ifit3b,Ifi44,Ifi209,Irf9,Ifitm2,Parp12,Oasl1,Xaf1,Tnfsf10,Irf7 | | 1.775e-16 | -36.27 | GO\_REGULATION\_OF\_CYTOKINE\_PRODUCTION | MSigDB lists | GO\_REGULATION\_OF\_CYTOKINE\_PRODUCTION | 389 | 33 | 12187 | 179 | Cd14,Isg15,Il17ra,Irak3,Thbs1,Il1a,Nfkb1,Bcl3,Ifi211,Iqgap1,Pik3r1,Il4ra,Il6,Tnfaip3,Ripk1,Ccl12,Ptgs2,Saa1,Errfi1,Zfp36,Hspb1,Trim25,Ifi209,Ifi207,Ifi204,Irf7,Trim56,C3ar1,C5ar1,Nfkb2,Birc3,Tnf,Runx1 | | 1.877e-16 | -36.21 | GSE41978\_ID2\_KO\_VS\_BIM\_KO\_KLRG1\_LOW\_EFFECTOR\_CD8\_TCELL\_UP | MSigDB lists | GSE41978\_ID2\_KO\_VS\_BIM\_KO\_KLRG1\_LOW\_EFFECTOR\_CD8\_TCELL\_UP | 164 | 23 | 12187 | 179 | Rtp4,Phf11d,Ifi211,Cxcl1,Arid5b,Ccl12,Maff,Tap1,Ifitm3,Isg15,Cebpd,Phf11b,Irf7,Bach1,Parp12,Oasl1,Icam1,Zfp36,Irf9,Ifi204,Ifi207,Ifi209,Ifit3b | | 2.154e-16 | -36.07 | GSE21546\_WT\_VS\_SAP1A\_KO\_AND\_ELK1\_KO\_DP\_THYMOCYTES\_UP | MSigDB lists | GSE21546\_WT\_VS\_SAP1A\_KO\_AND\_ELK1\_KO\_DP\_THYMOCYTES\_UP | 165 | 23 | 12187 | 179 | Nfkbia,Tor3a,Xaf1,Ifit3b,Tagln2,Icam1,Ifit2,Map3k8,Gpr84,Ccl2,Ccl4,Cybb,Nfe2l2,Nfkbie,Gbp7,Pik3r5,Ptges,Cmpk2,Rtp4,Nfkb1,Lcn2,Ptpn1,Parp9 | | 2.283e-16 | -36.02 | regulation of multi-organism process | biological process | GO:0043900 | 468 | 37 | 13711 | 214 | Irak3,Cd14,Tnip1,Trim30a,Ifi204,Acod1,Tnfaip3,Tnf,Pik3ap1,Zfp36,Irgm2,Cxcl1,Ifitm2,A2m,Tnip3,Tap1,Irf7,Oasl1,Ifitm3,Dtx3l,Trib1,Parp14,Lgals9,Oasl2,Nfkbia,Ifitm6,Ifi211,Irgm1,Ccl4,Trim56,Resf1,Trim25,Ifi209,Gbp4,Timp1,Isg15,Parp9 | | 2.888e-16 | -35.78 | GSE42021\_CD24HI\_VS\_CD24LOW\_TREG\_THYMUS\_DN | MSigDB lists | GSE42021\_CD24HI\_VS\_CD24LOW\_TREG\_THYMUS\_DN | 149 | 22 | 12187 | 179 | Usp18,Ifit2,Tnfaip3,Ifi211,Phf11d,Phf11b,Ifitm3,Isg15,Herc6,Xaf1,Oasl1,Parp12,Irf7,Tnfsf10,Ifit3b,Ifi209,Ifi44,Atf3,Ifi204,Ifi207,Ifitm2,Irf9 | | 3.057e-16 | -35.72 | GO\_REGULATION\_OF\_DEFENSE\_RESPONSE | MSigDB lists | GO\_REGULATION\_OF\_DEFENSE\_RESPONSE | 538 | 38 | 12187 | 179 | Nfkb1,Tap1,Parp9,Ptpn1,Pik3ap1,Il17ra,Irak3,Cd14,Il6,Ripk1,Tnfaip3,Ccl12,Usp18,Ccl2,Ccl4,Sbno2,S100a8,Osmr,Ifi211,Ifi207,Tnip3,Ifi204,Socs3,Ifi209,Sele,Tnip1,Saa1,Ptgs2,Casp4,Nfkbia,Birc3,Tnf,Zfp189,Irf7,S100a9,A2m,C3ar1,C5ar1 | | 6.958e-16 | -34.90 | RASHI\_RESPONSE\_TO\_IONIZING\_RADIATION\_2 | MSigDB lists | RASHI\_RESPONSE\_TO\_IONIZING\_RADIATION\_2 | 105 | 19 | 12187 | 179 | Cxcl1,Nfkb2,Nfkbia,Birc3,Rhou,Map3k8,Nfe2l2,Tnfaip3,Cd14,Tagln2,Tnip1,Icam1,Nfkbiz,Thbs1,Cdkn1a,Cebpd,Lcn2,Atf3,Hp | | 7.045e-16 | -34.89 | REACTOME\_INTERFERON\_ALPHA\_BETA\_SIGNALING | MSigDB lists | REACTOME\_INTERFERON\_ALPHA\_BETA\_SIGNALING | 43 | 14 | 12187 | 179 | Irf7,Usp18,Xaf1,Ifit2,Oasl1,Ifitm2,Isg15,Ifitm3,Irf9,Irf2,Ptpn1,Ip6k2,Ifit3b,Socs3 | | 9.205e-16 | -34.62 | GSE37301\_MULTIPOTENT\_PROGENITOR\_VS\_GRAN\_MONO\_PROGENITOR\_DN | MSigDB lists | GSE37301\_MULTIPOTENT\_PROGENITOR\_VS\_GRAN\_MONO\_PROGENITOR\_DN | 176 | 23 | 12187 | 179 | Tap1,Clic4,Icam1,Isg15,Lcn2,Znfx1,Gadd45g,Ifit3b,Nfkb2,Xdh,Gbp3,Osmr,Cmpk2,Irf7,Gbp7,Irgm2,Ripk1,Tubb6,Ccl2,Usp18,Casp4,Birc3,Ifit2 | | 1.232e-15 | -34.33 | MODULE\_5 | MSigDB lists | MODULE\_5 | 336 | 30 | 12187 | 179 | Tap1,Cd14,Lcn2,Ier3,Thbs1,S100a8,Cxcl1,Ifi211,Cybb,Il6,Tnfaip3,Ccl12,Maff,Il4ra,Tgm2,Icam1,Irf9,Csf2rb,Plaur,Col4a1,Hp,Ifi207,Ifi204,Il1rn,Cdkn1a,Ifi209,Socs3,Tnfsf10,S100a9,Casp4 | | 1.363e-15 | -34.23 | defense response to virus | biological process | GO:0051607 | 133 | 21 | 13711 | 214 | Trim30a,Mx1,Dtx3l,Ifitm3,Oasl1,Irf7,Il6,Cxcl9,Slfn9,Oasl2,Trim25,Ifit2,Trim56,Mx2,Ifit3b,Rtp4,Ifitm6,Parp9,Irf2,Isg15,Ifitm2 | | 1.479e-15 | -34.15 | positive regulation of immune system process | biological process | GO:0002684 | 612 | 41 | 13711 | 214 | Runx1,Ccl4,Ifi211,Irgm1,Parp9,Isg15,Ifi209,Lgals9,Hcar2,Trib1,Thbs1,Irf7,Cdkn1a,Il6,Nfkbia,Fyb,Il1a,Il4ra,Icam1,Irgm2,Selp,Mmp8,Vcam1,Ccl12,Tnip3,C5ar1,Cxcl1,Nfkbiz,Ifi204,Tnip1,Zbtb16,Ripk1,Csf3,Cd14,C3ar1,Irak3,Sele,Ccl2,Pik3ap1,Tnf,Acod1 | | 1.533e-15 | -34.11 | Immune System | REACTOME pathways | R-MMU-168256 | 1188 | 56 | 6297 | 105 | Lgals9,Serpina3f,Ksr1,Il17ra,Csf2rb,Cxcl1,Osmr,Iqgap1,Irak3,Fyb,Rnf213,Vcam1,C5ar1,Ptpn1,Lcn2,Ier3,Csf3,Birc3,Pik3ap1,Map3k8,Irf2,Ifitm3,Plaur,Mmp8,Il4ra,Nfkb2,Icam1,Socs3,Usp18,C3ar1,Hp,Isg15,Herc6,Tnf,Ncf1,Nfkbia,Nfkbie,S100a8,Tap1,Tnfaip3,Il1a,Cd14,Irf7,Nfkb1,Xdh,Ifitm2,Trim56,Irf9,Pik3r1,Gpr84,S100a9,Dtx3l,Il1rn,Cybb,Il6,Cdkn1a | | 1.715e-15 | -34.00 | positive regulation of response to external stimulus | biological process | GO:0032103 | 341 | 31 | 13711 | 214 | Ccl4,Ifi211,Irgm1,Il17ra,Ptgs2,Ifi209,Parp9,Irf7,Thbs1,Lgals9,Nfkbia,Il6,S100a9,Mmp8,Irgm2,Nfkbiz,Tgm2,Cxcl1,C5ar1,Tnip3,S100a8,Cd14,Hspb1,Irak3,C3ar1,Ifi204,Tnip1,Pik3ap1,Tnf,Acod1,Ccl2 | | 2.171e-15 | -33.76 | Immunity-related\_GTPase-like | interpro domains | IPR007743 | 11 | 9 | 13788 | 212 | Iigp1,F830016B08Rik,Ifi47,Irgm1,9930111J21Rik2,Irgm2,Tgtp1,Gm5431,Gm4841 | | 2.260e-15 | -33.72 | positive regulation of response to stimulus | biological process | GO:0048584 | 1637 | 69 | 13711 | 214 | Thbs1,Gadd45g,P2ry6,Lgals9,Icam1,Fyb,Sox11,Irgm1,Pik3r5,Il17ra,Plaur,Map3k8,Nfkb1,C3ar1,Irak3,Il1rn,Csf3,Ifi204,Tnfaip3,Plek,Xdh,Tnfsf10,Ksr1,Ccl2,S100a9,Irgm2,Nfkbiz,Ptpn1,S100a8,Iqgap1,Tnip3,Ccl12,Dtx3l,Irf7,Parp14,Ccl7,Gadd45b,Pik3r1,Il4ra,Casp4,Nfkbia,Il1a,Akap12,Il6,Ifi211,Ccl4,Runx1,Ptgs2,Trim25,Ifi209,Map3k6,Fzd4,Parp9,Ncf1,Hspb1,Ripk1,Cflar,Cd14,Tnip1,Tnf,Acod1,Pik3ap1,Atf3,Nfe2l2,Mmp8,Selp,C5ar1,Cxcl1,Tgm2 | | 2.456e-15 | -33.64 | IIGP | pfam domains | PF05049 | 11 | 9 | 12881 | 201 | Tgtp1,Irgm2,Irgm1,Gm4841,Iigp1,Ifi47,9930111J21Rik2,Gm5431,F830016B08Rik | | 2.466e-15 | -33.64 | regulation of cytokine production | biological process | GO:0001817 | 504 | 37 | 13711 | 214 | Irf7,Thbs1,Lgals9,Pik3r1,Il1a,Akap12,Casp4,Il4ra,Il6,Errfi1,Cybb,Ccl4,Ifi211,Il17ra,Ptgs2,Runx1,Gbp4,Isg15,Nfkb1,Cd14,Ripk1,Hspb1,C3ar1,Il1rn,Irak3,Trim30a,Ifi204,Tnfaip3,Tnf,Acod1,Ccl2,Zfp36,Mmp8,Bcl3,C5ar1,Prg4,Iqgap1 | | 2.747e-15 | -33.53 | GO\_CELLULAR\_RESPONSE\_TO\_ORGANIC\_SUBSTANCE | MSigDB lists | GO\_CELLULAR\_RESPONSE\_TO\_ORGANIC\_SUBSTANCE | 1393 | 60 | 12187 | 179 | Nfkb1,Tiparp,Il1a,Cxcl16,Irak3,Il17ra,Cd14,Ifitm3,Isg15,Ccl12,Il6,Nfe2l2,Ccl4,Msr1,Sbno2,Iqgap1,Cmpk2,Osmr,Csf3,Atf3,Tnip3,Vcam1,Socs3,Ifit3b,Adamts1,Errfi1,Csf2rb,Msn,Hspb1,Ifitm2,Ptgs2,Gbp4,Xaf1,Nfkbia,Tnf,Birc3,Gbp6,Sgk1,Hspa5,Irf2,Ptpn1,Ip6k2,P2ry6,Tnfaip3,Ripk1,Ccl2,Spi1,Ifit2,Pik3r1,Il4ra,Cxcl1,Col4a1,Fstl1,Irf9,Trim25,Icam1,Oasl1,Casp4,Irf7,Fzd4 | | 2.995e-15 | -33.44 | GRAESSMANN\_RESPONSE\_TO\_MC\_AND\_SERUM\_DEPRIVATION\_UP | MSigDB lists | GRAESSMANN\_RESPONSE\_TO\_MC\_AND\_SERUM\_DEPRIVATION\_UP | 166 | 22 | 12187 | 179 | Tor3a,Parp14,Usp18,Ifit2,Ccl12,Parp12,Irf7,Irgm2,Gbp7,Slfn4,Rtp4,Cmpk2,Fstl1,Ifit3b,Ifi44,Cp,Isg15,Irf2,Parp9,Irf9,Trim25,Slfn3 | | 3.090e-15 | -33.41 | ZHANG\_INTERFERON\_RESPONSE | MSigDB lists | ZHANG\_INTERFERON\_RESPONSE | 22 | 11 | 12187 | 179 | Ifi209,Ifi44,Ifi204,Ifi207,Ifitm3,Isg15,Irf9,Ifit2,Oasl1,Ifi211,Rtp4 | | 3.097e-15 | -33.41 | GSE7509\_UNSTIM\_VS\_IFNA\_STIM\_IMMATURE\_DC\_DN | MSigDB lists | GSE7509\_UNSTIM\_VS\_IFNA\_STIM\_IMMATURE\_DC\_DN | 130 | 20 | 12187 | 179 | Gbp6,Phf11d,Tnfsf10,Ifit2,Xaf1,Tgm2,Nfkbia,Usp18,Casp4,Gbp4,Parp12,Isg15,Icam1,Trim25,Phf11b,Ifi44,Vcam1,Ifit3b,Stx11,Samd9l | | 3.156e-15 | -33.39 | GO\_RESPONSE\_TO\_MOLECULE\_OF\_BACTERIAL\_ORIGIN | MSigDB lists | GO\_RESPONSE\_TO\_MOLECULE\_OF\_BACTERIAL\_ORIGIN | 228 | 25 | 12187 | 179 | Nfkb1,Cd14,Selp,Cxcl16,Irak3,Ccl12,Tnfaip3,Il6,Cxcl1,Ptges,Csf3,Sbno2,S100a8,Cmpk2,Vcam1,Socs3,Tnip3,Ptgs2,Icam1,Sele,Nfkbia,Trib1,Tnf,C5ar1,Nfkb2 | | 3.549e-15 | -33.27 | GO\_REGULATION\_OF\_INFLAMMATORY\_RESPONSE | MSigDB lists | GO\_REGULATION\_OF\_INFLAMMATORY\_RESPONSE | 187 | 23 | 12187 | 179 | Sele,Tnip1,Saa1,Ptgs2,Socs3,A2m,S100a9,C3ar1,C5ar1,Casp4,Birc3,Tnf,Pik3ap1,Il17ra,Nfkb1,Sbno2,Osmr,S100a8,Il6,Tnfaip3,Ccl12,Ccl2,Ccl4 | | 3.965e-15 | -33.16 | GO\_DEFENSE\_RESPONSE\_TO\_OTHER\_ORGANISM | MSigDB lists | GO\_DEFENSE\_RESPONSE\_TO\_OTHER\_ORGANISM | 276 | 27 | 12187 | 179 | Gbp4,Oasl1,Zfp189,Tnf,Trim56,S100a9,Irf7,C5ar1,Gbp6,Ifi204,Hp,Ifi207,Ifit3b,Ifi209,Irf9,Trim25,Ifitm2,Il6,Ifit2,Il4ra,S100a8,Ifi211,Bcl3,Il17ra,Selp,Ifitm3,Isg15 | | 5.651e-15 | -32.81 | GSE42021\_CD24HI\_TREG\_VS\_CD24HI\_TCONV\_THYMUS\_DN | MSigDB lists | GSE42021\_CD24HI\_TREG\_VS\_CD24HI\_TCONV\_THYMUS\_DN | 171 | 22 | 12187 | 179 | Gadd45g,Il1a,Ier3,Sgk1,Nfkbiz,Selp,Cd14,Gadd45b,Map3k8,Ccnd2,Il4ra,Ccl2,Cxcl1,Il1rn,Cdkn1a,Socs3,Icam1,Plaur,Errfi1,Ptgs2,Birc3,Nfkbia | | 5.848e-15 | -32.77 | TIAN\_TNF\_SIGNALING\_VIA\_NFKB | MSigDB lists | TIAN\_TNF\_SIGNALING\_VIA\_NFKB | 23 | 11 | 12187 | 179 | Nfkbia,Sdc4,Nfkb1,Il6,Tnfaip3,Nfkbie,Ptgs2,Cxcl1,Tnip1,Zfp36,Nfkb2 | | 7.702e-15 | -32.50 | LIANG\_SILENCED\_BY\_METHYLATION\_2 | MSigDB lists | LIANG\_SILENCED\_BY\_METHYLATION\_2 | 31 | 12 | 12187 | 179 | Ifi44,Ifit3b,Lcn2,Ptgs2,Icam1,Ifit2,Xaf1,Tnfaip3,Il6,Cxcl1,Irf7,Tnfsf10 | | 8.569e-15 | -32.39 | G\_IRG\_dom | interpro domains | IPR030385 | 12 | 9 | 13788 | 212 | 9930111J21Rik2,Irgm2,Tgtp1,Gm5431,Gm4841,F830016B08Rik,Iigp1,Ifi47,Irgm1 | | 8.794e-15 | -32.36 | GSE7348\_UNSTIM\_VS\_LPS\_STIM\_MACROPHAGE\_DN | MSigDB lists | GSE7348\_UNSTIM\_VS\_LPS\_STIM\_MACROPHAGE\_DN | 137 | 20 | 12187 | 179 | Plek,Icam1,Tnip1,Ptgs2,Isg15,Atf3,Il1a,Nfkb1,Stx11,Vcam1,Socs3,Igsf6,Arid5b,Ptges,Cflar,Tnfaip3,Casp4,Ccl4,Birc3,Map3k8 | | 8.794e-15 | -32.36 | GSE35825\_IFNA\_VS\_IFNG\_STIM\_MACROPHAGE\_UP | MSigDB lists | GSE35825\_IFNA\_VS\_IFNG\_STIM\_MACROPHAGE\_UP | 137 | 20 | 12187 | 179 | Zfp36,Tap1,Errfi1,Slfn5,Nfkb1,Il1rn,Atf3,Ier3,Vcam1,Rtp4,Cmpk2,Gbp3,Gbp6,Cflar,Irf7,Cxcl1,Parp12,Gbp4,Ifit2,Parp14 | | 9.655e-15 | -32.27 | GSE46606\_IRF4MID\_VS\_WT\_CD40L\_IL2\_IL5\_DAY1\_STIMULATED\_BCELL\_DN | MSigDB lists | GSE46606\_IRF4MID\_VS\_WT\_CD40L\_IL2\_IL5\_DAY1\_STIMULATED\_BCELL\_DN | 156 | 21 | 12187 | 179 | Socs3,Nfkbiz,Nfkb1,Cebpd,Tnip1,Icam1,Irak3,Plek,Ptpn1,Tnf,Birc3,Maff,Ccl4,Nfkbia,Hcar2,Tnfaip3,Cflar,Csf3,Nfkbie,Pik3r5,Nfkb2 | | 1.049e-14 | -32.19 | PHONG\_TNF\_TARGETS\_UP | MSigDB lists | PHONG\_TNF\_TARGETS\_UP | 51 | 14 | 12187 | 179 | Icam1,Zfp36,Ier3,Atf3,Cebpd,Adamts9,Nfkb2,Gem,Cxcl1,Tnfaip3,Il6,Birc3,Nfkbia,Sdc4 | | 1.080e-14 | -32.16 | MODULE\_45 | MSigDB lists | MODULE\_45 | 419 | 32 | 12187 | 179 | Ifi209,Ifi44,Ifit3b,Stx11,Kcna5,Il1rn,Ifi204,Ifi207,Plek,Csf2rb,Tnf,Birc3,Casp4,Oasl1,S100a9,Olfml2b,Igsf6,Xdh,Nfkb2,Tnfsf10,Ier3,Cd14,Tap1,Ccnd2,Il4ra,Spi1,Cybb,Tnfaip3,Ifi211,Tnfaip8,S100a8,Sbno2 | | 1.171e-14 | -32.08 | GSE35825\_UNTREATED\_VS\_IFNA\_STIM\_MACROPHAGE\_DN | MSigDB lists | GSE35825\_UNTREATED\_VS\_IFNA\_STIM\_MACROPHAGE\_DN | 139 | 20 | 12187 | 179 | Parp12,Il6,Tnfaip3,Usp18,Xaf1,Tgm2,Nfkb2,Gbp3,Cmpk2,Pik3r5,Nfkbie,Cflar,Atf3,Znfx1,Timp1,Il1rn,Nfkbiz,Ifit3b,Tap1,Isg15 | | 1.182e-14 | -32.07 | immune effector process | biological process | GO:0002252 | 316 | 29 | 13711 | 214 | Icam1,Oasl2,Slfn9,Il4ra,Il6,Oasl1,Irf7,Dtx3l,Ifitm3,Mx1,Gadd45g,Sbno2,Isg15,Parp9,Rtp4,Mx2,Stx11,Ifitm6,Trim25,Trim56,Cxcl9,Tnfaip3,Ncf1,Trim30a,Ifitm2,Irf2,Ifit3b,Ifit2,Bcl3 | | 1.201e-14 | -32.05 | BASSO\_CD40\_SIGNALING\_UP | MSigDB lists | BASSO\_CD40\_SIGNALING\_UP | 76 | 16 | 12187 | 179 | Nfkb2,Tnfaip8,Arid5b,Nfkbie,Cflar,Gadd45b,Tnfaip3,Nfkbia,Ccl4,Map3k8,Tnf,Irf9,Tap1,Plek,Icam1,Ier3 | | 2.350e-14 | -31.38 | GSE42021\_TREG\_PLN\_VS\_CD24LO\_TREG\_THYMUS\_DN | MSigDB lists | GSE42021\_TREG\_PLN\_VS\_CD24LO\_TREG\_THYMUS\_DN | 144 | 20 | 12187 | 179 | Ifi204,Il1rn,Ifi207,Ifit3b,Phf11b,Ifi209,Ifi44,Irf9,Tap1,Zfp36,Ifitm3,Herc6,Ifitm2,Isg15,Oasl1,Parp12,Usp18,Ifi211,Phf11d,Irf7 | | 2.350e-14 | -31.38 | GSE360\_CTRL\_VS\_M\_TUBERCULOSIS\_DC\_DN | MSigDB lists | GSE360\_CTRL\_VS\_M\_TUBERCULOSIS\_DC\_DN | 144 | 20 | 12187 | 179 | Tnip1,Ifitm2,Irf9,Csf2rb,Icam1,Dnajb1,Ifi44,Ier3,Il1rn,Stx11,S100a9,C3ar1,Tnfsf10,Sbno2,S100a8,Nfkbia,Trib1,Sntb2,Ccl4,Ifit2 | | 2.692e-14 | -31.25 | GSE6259\_FLT3L\_INDUCED\_DEC205\_POS\_DC\_VS\_CD8\_TCELL\_DN | MSigDB lists | GSE6259\_FLT3L\_INDUCED\_DEC205\_POS\_DC\_VS\_CD8\_TCELL\_DN | 145 | 20 | 12187 | 179 | Parp12,Parp14,Ccl4,Map3k8,Sbno2,Gbp3,Cmpk2,Rtp4,Phf11d,Irf7,Cflar,Ifit3b,Phf11b,Cdkn1a,Irf9,Trim25,Icam1,Slfn5,Isg15,Rnf213 | | 3.034e-14 | -31.13 | GSE37534\_UNTREATED\_VS\_PIOGLITAZONE\_TREATED\_CD4\_TCELL\_PPARG1\_AND\_FOXP3\_TRASDUCED\_DN | MSigDB lists | GSE37534\_UNTREATED\_VS\_PIOGLITAZONE\_TREATED\_CD4\_TCELL\_PPARG1\_AND\_FOXP3\_TRASDUCED\_DN | 165 | 21 | 12187 | 179 | Ifit3b,Ifi44,Atf3,Ifitm2,Isg15,Herc6,Ifitm3,Tap1,Irf9,Trim25,Usp18,Xaf1,Ifit2,Il6,Oasl1,Parp12,Gadd45b,Ccl12,Irf7,Cflar,Rtp4 | | 3.036e-14 | -31.13 | DER\_IFN\_BETA\_RESPONSE\_UP | MSigDB lists | DER\_IFN\_BETA\_RESPONSE\_UP | 95 | 17 | 12187 | 179 | Iqgap1,Ifi211,Il6,Oasl1,Rhoc,Ifit2,Irf9,Tap1,Plaur,Irf2,Isg15,Cebpd,Ifi207,Ifi204,Ifit3b,Ifi44,Ifi209 | | 3.752e-14 | -30.91 | HELLER\_SILENCED\_BY\_METHYLATION\_UP | MSigDB lists | HELLER\_SILENCED\_BY\_METHYLATION\_UP | 187 | 22 | 12187 | 179 | Sbno2,Olfml2b,C5ar1,Usp18,Birc3,Msr1,Map3k8,Irf9,Csf2rb,Plek,Icam1,Ncf1,Herc6,Isg15,Map3k6,Ier3,Akap12,Ifit3b,Socs3,Vcam1,Ifi44,Cdkn1a | | 3.985e-14 | -30.85 | HINATA\_NFKB\_TARGETS\_KERATINOCYTE\_UP | MSigDB lists | HINATA\_NFKB\_TARGETS\_KERATINOCYTE\_UP | 68 | 15 | 12187 | 179 | Nfkbia,Tnf,Ccl12,Tnfaip3,Il6,Cflar,Tnfaip8,Cdkn1a,Il1rn,Nfkb1,Il1a,Tnip1,Saa1,Tap1,Icam1 | | 4.363e-14 | -30.76 | G\_IRG | prosite domains | PS51716 | 12 | 9 | 8845 | 164 | Irgm2,Gm5431,F830016B08Rik,Tgtp1,9930111J21Rik2,Gm4841,Irgm1,Iigp1,Ifi47 | | 4.366e-14 | -30.76 | GO\_REGULATION\_OF\_IMMUNE\_SYSTEM\_PROCESS | MSigDB lists | GO\_REGULATION\_OF\_IMMUNE\_SYSTEM\_PROCESS | 947 | 47 | 12187 | 179 | Csf3,Ccl4,Map3k8,Nfe2l2,Il6,Ccl12,Cd14,Isg15,Selp,Tap1,Parp9,Pik3ap1,Irak3,Thbs1,Nfkb1,A2m,C3ar1,C5ar1,Nfkbia,Birc3,Zfp189,Tnf,Tnip1,Msn,Vcam1,Socs3,Zbtb16,Ifi207,Tnip3,Cxcl1,Ifi211,Spi1,Usp18,Pik3r1,Il4ra,Tnfaip3,Ripk1,Hcar2,Ptpn1,Sox11,Irf7,Trib1,Runx1,Icam1,Ifi209,Cdkn1a,Ifi204 | | 4.421e-14 | -30.75 | BOSCO\_INTERFERON\_INDUCED\_ANTIVIRAL\_MODULE | MSigDB lists | BOSCO\_INTERFERON\_INDUCED\_ANTIVIRAL\_MODULE | 56 | 14 | 12187 | 179 | Parp12,Oasl1,Ccl12,Xaf1,Tnfsf10,Gbp3,Irf7,Gbp7,Ms4a6d,Samd9l,Tnip3,Ifit3b,Ifi44,Ifitm3 | | 4.756e-14 | -30.68 | GO\_POSITIVE\_REGULATION\_OF\_IMMUNE\_SYSTEM\_PROCESS | MSigDB lists | GO\_POSITIVE\_REGULATION\_OF\_IMMUNE\_SYSTEM\_PROCESS | 596 | 37 | 12187 | 179 | Hcar2,Tnfaip3,Ripk1,Ccl12,Il6,Map3k8,Il4ra,Pik3r1,Ccl4,Csf3,Ifi211,Cxcl1,Nfkb1,Thbs1,Irak3,Pik3ap1,Tap1,Selp,Isg15,Cd14,Tnf,Runx1,Birc3,Trib1,Nfkbia,C5ar1,C3ar1,Irf7,Ifi204,Tnip3,Ifi207,Ifi209,Cdkn1a,Zbtb16,Vcam1,Icam1,Tnip1 | | 4.917e-14 | -30.64 | cellular response to lipopolysaccharide | biological process | GO:0071222 | 123 | 19 | 13711 | 214 | Cd14,Il1rn,Nfkbia,Cxcl9,Acod1,Tnf,Tnfaip3,Ccl2,Il6,Zfp36,Gbp6,Irgm2,Cxcl1,Cxcl16,Ccl12,Sbno2,Cmpk2,Tnip3,Nfkb1 | | 4.931e-14 | -30.64 | GSE37533\_PPARG1\_FOXP3\_VS\_FOXP3\_TRANSDUCED\_CD4\_TCELL\_PIOGLITAZONE\_TREATED\_UP | MSigDB lists | GSE37533\_PPARG1\_FOXP3\_VS\_FOXP3\_TRANSDUCED\_CD4\_TCELL\_PIOGLITAZONE\_TREATED\_UP | 169 | 21 | 12187 | 179 | Rtp4,Tnfsf10,Phf11d,Ifi211,Irf7,Parp12,Oasl1,Ifit2,Xaf1,Usp18,Trim25,Ifitm3,Isg15,Ifitm2,Herc6,Ifi204,Ifi207,Ifi209,Phf11b,Ifi44,Ifit3b | | 5.224e-14 | -30.58 | GSE21360\_PRIMARY\_VS\_QUATERNARY\_MEMORY\_CD8\_TCELL\_UP | MSigDB lists | GSE21360\_PRIMARY\_VS\_QUATERNARY\_MEMORY\_CD8\_TCELL\_UP | 150 | 20 | 12187 | 179 | Rnf213,Ifitm3,Clic4,Trim25,Ifit3b,Ifi44,Atf3,Znfx1,Samd9l,Tnip3,Il1rn,Mmp8,Irf7,Cmpk2,Usp18,Xaf1,Ifit2,Ccl12,Rassf4,Cybb | | 5.224e-14 | -30.58 | GSE46606\_IRF4\_KO\_VS\_WT\_UNSTIM\_BCELL\_DN | MSigDB lists | GSE46606\_IRF4\_KO\_VS\_WT\_UNSTIM\_BCELL\_DN | 150 | 20 | 12187 | 179 | Birc3,Tnf,Nfkbia,Ccl4,Hcar2,Cybb,Tnfaip3,Nfkbie,Csf3,Cflar,Irf7,Ptges,Nfkb2,Nfkbiz,Socs3,Lcn2,Cebpd,Tnip1,Plek,Irak3 | | 5.224e-14 | -30.58 | GSE16755\_CTRL\_VS\_IFNA\_TREATED\_MAC\_DN | MSigDB lists | GSE16755\_CTRL\_VS\_IFNA\_TREATED\_MAC\_DN | 150 | 20 | 12187 | 179 | Parp12,Il6,Sdc4,Nfkbia,Cmpk2,Tnfaip8,Tnfsf10,Dtx3l,Trim56,Samd9l,Stx11,Tnip3,Ifi44,Cdkn1a,Socs3,Vcam1,Icam1,Herc6,Ptgs2,Isg15 | | 5.902e-14 | -30.46 | SARRIO\_EPITHELIAL\_MESENCHYMAL\_TRANSITION\_DN | MSigDB lists | SARRIO\_EPITHELIAL\_MESENCHYMAL\_TRANSITION\_DN | 115 | 18 | 12187 | 179 | S100a8,Ifi211,Gem,Nfe2l2,Tnfaip3,Nfkbia,Birc3,Errfi1,Ifitm2,Ifitm3,Atf3,Gadd45g,Ifi207,Ifi204,Il1rn,Zbtb16,Ifi44,Ifi209 | | 6.898e-14 | -30.30 | GSE37605\_TREG\_VS\_TCONV\_NOD\_FOXP3\_FUSION\_GFP\_UP | MSigDB lists | GSE37605\_TREG\_VS\_TCONV\_NOD\_FOXP3\_FUSION\_GFP\_UP | 116 | 18 | 12187 | 179 | Adamts9,Fzd4,Slc24a4,Ccl12,Birc3,Maff,Trib1,Zfp36,Sele,Apold1,Ptgs2,Il1a,Akap12,Cebpd,Atf3,Ier3,Sgk1,Adamts1 | | 7.908e-14 | -30.17 | GO\_RESPONSE\_TO\_VIRUS | MSigDB lists | GO\_RESPONSE\_TO\_VIRUS | 173 | 21 | 12187 | 179 | Oasl1,Il6,Ccl4,Tnf,Ifit2,Irf7,Trim56,Bcl3,Ifi211,Ifi207,Ifi204,Ifit3b,Ifi44,Ifi209,Irf9,Hspb1,Irak3,Trim25,Isg15,Ifitm2,Ifitm3 | | 8.376e-14 | -30.11 | PHONG\_TNF\_RESPONSE\_NOT\_VIA\_P38 | MSigDB lists | PHONG\_TNF\_RESPONSE\_NOT\_VIA\_P38 | 287 | 26 | 12187 | 179 | Arid5b,Bcl3,Ptges,Phf11d,Sbno2,Tnfaip8,Adamts9,Irf2,Dnajb1,Hspa5,Sgk1,Phf11b,Nfkb1,Gem,Cflar,Nfkb2,Trib1,Nfkbia,Sdc4,Birc3,Parp12,Tnip1,Plaur,Rbm47,Icam1,Vcam1 | | 8.463e-14 | -30.10 | GSE41867\_NAIVE\_VS\_DAY30\_LCMV\_CLONE13\_EXHAUSTED\_CD8\_TCELL\_UP | MSigDB lists | GSE41867\_NAIVE\_VS\_DAY30\_LCMV\_CLONE13\_EXHAUSTED\_CD8\_TCELL\_UP | 135 | 19 | 12187 | 179 | Plek,Errfi1,Rnf213,Herc6,Ptgs2,Tnip3,Stx11,Samd9l,Znfx1,Cflar,Pik3r5,Ripk1,Il6,Oasl1,Map3k8,Parp14,Ccl4,Usp18,Sdc4 | | 8.497e-14 | -30.10 | MODULE\_44 | MSigDB lists | MODULE\_44 | 239 | 24 | 12187 | 179 | Ifi211,S100a9,Sbno2,Tnfaip8,S100a8,Tgm2,Casp4,Il4ra,Pik3r1,Birc3,Tnfaip3,Fam107a,Cd14,Isg15,Tap1,Irf9,Fstl1,Vcam1,Ifit3b,Ifi209,Ifi44,Ifi204,Col4a1,Ifi207 | | 8.716e-14 | -30.07 | GSE21360\_NAIVE\_VS\_QUATERNARY\_MEMORY\_CD8\_TCELL\_DN | MSigDB lists | GSE21360\_NAIVE\_VS\_QUATERNARY\_MEMORY\_CD8\_TCELL\_DN | 154 | 20 | 12187 | 179 | Ripk1,Parp12,Oasl1,Xaf1,Usp18,Casp4,Ifit2,Rtp4,Phf11d,Irf7,Nfkbie,Ifit3b,Phf11b,Ifi44,Tap1,Irf9,Ifitm3,Ifitm2,Isg15,Herc6 | | 8.716e-14 | -30.07 | GSE41176\_UNSTIM\_VS\_ANTI\_IGM\_STIM\_BCELL\_1H\_UP | MSigDB lists | GSE41176\_UNSTIM\_VS\_ANTI\_IGM\_STIM\_BCELL\_1H\_UP | 154 | 20 | 12187 | 179 | Il6,Gadd45b,Casp4,Trib1,Nfkbia,Ccl4,Tnfaip8,Nfkbie,Bcl3,Cxcl1,Il1a,Nfkb1,Tnip3,Ier3,Socs3,Icam1,Clic4,Plaur,Ptgs2,Tnip1 | | 8.881e-14 | -30.05 | GSE36527\_CD62L\_HIGH\_VS\_CD62L\_LOW\_TREG\_CD69\_NEG\_KLRG1\_NEG\_UP | MSigDB lists | GSE36527\_CD62L\_HIGH\_VS\_CD62L\_LOW\_TREG\_CD69\_NEG\_KLRG1\_NEG\_UP | 174 | 21 | 12187 | 179 | Parp12,Oasl1,Map3k8,Ifit2,Usp18,Parp14,Tor3a,Rtp4,Irgm2,Irf7,Znfx1,Hspa5,Ifit3b,Socs3,Trim25,Tap1,Irf9,Parp9,Clic4,Isg15,Slfn9 | | 1.042e-13 | -29.89 | GO\_POSITIVE\_REGULATION\_OF\_DEFENSE\_RESPONSE | MSigDB lists | GO\_POSITIVE\_REGULATION\_OF\_DEFENSE\_RESPONSE | 265 | 25 | 12187 | 179 | S100a8,Osmr,Ifi211,Il6,Ripk1,Ccl12,Tnfaip3,Ccl2,Ccl4,Pik3ap1,Il17ra,Irak3,Cd14,Nfkb1,Irf7,S100a9,Nfkbia,Birc3,Tnf,Tnip1,Ptgs2,Ifi207,Ifi204,Tnip3,Ifi209 | | 1.114e-13 | -29.83 | GSE25123\_WT\_VS\_PPARG\_KO\_MACROPHAGE\_UP | MSigDB lists | GSE25123\_WT\_VS\_PPARG\_KO\_MACROPHAGE\_UP | 137 | 19 | 12187 | 179 | Cxcl1,Ptges,S100a8,Map3k8,Oasl1,Il6,Tnfaip3,AA467197,Ifitm2,Slfn5,Irak3,Msn,Ifi44,Thbs1,Nfkbiz,Socs3,Tnip3,Lcn2,Cebpd | | 1.275e-13 | -29.69 | GSE6269\_HEALTHY\_VS\_FLU\_INF\_PBMC\_DN | MSigDB lists | GSE6269\_HEALTHY\_VS\_FLU\_INF\_PBMC\_DN | 138 | 19 | 12187 | 179 | Ifi211,Sbno2,Igsf6,Xaf1,Usp18,Spi1,Oasl1,Tagln2,Ifitm3,Isg15,Ifitm2,Herc6,Msn,Trim25,Ifit3b,Ifi209,Ifi44,Ifi204,Ifi207 | | 1.286e-13 | -29.68 | response to interferon-gamma | biological process | GO:0034341 | 97 | 17 | 13711 | 214 | Irgm1,Ifitm6,Stx11,Ccl4,Gbp7,Gbp6,Irgm2,Gbp4,Gbp3,Ifitm2,Tgtp1,Cxcl16,Ccl12,Ifitm3,Ccl7,Acod1,Ccl2 | | 1.390e-13 | -29.60 | cellular response to molecule of bacterial origin | biological process | GO:0071219 | 130 | 19 | 13711 | 214 | Cxcl1,Cxcl16,Sbno2,Ccl12,Cmpk2,Tnip3,Nfkb1,Gbp6,Irgm2,Cxcl9,Nfkbia,Tnf,Tnfaip3,Acod1,Ccl2,Il6,Zfp36,Cd14,Il1rn | | 1.618e-13 | -29.45 | GSE42021\_TREG\_PLN\_VS\_CD24INT\_TREG\_THYMUS\_DN | MSigDB lists | GSE42021\_TREG\_PLN\_VS\_CD24INT\_TREG\_THYMUS\_DN | 159 | 20 | 12187 | 179 | Cxcl1,Ifi211,Phf11d,Rtp4,Casp4,Tgm2,Parp12,Oasl1,Isg15,Zfp36,Trim25,Tap1,Irf9,Plaur,Ifi44,Ifi209,Phf11b,Ifit3b,Ifi207,Ifi204 | | 1.622e-13 | -29.45 | cellular response to biotic stimulus | biological process | GO:0071216 | 149 | 20 | 13711 | 214 | Cd14,Il1rn,Ccl2,Zfp36,Il6,Nfkbia,Cxcl9,Tnf,Tnfaip3,Acod1,Gbp6,Irgm2,Cxcl16,Ccl12,Sbno2,Cmpk2,Tnip3,Nfkb1,Hspa5,Cxcl1 | | 1.826e-13 | -29.33 | GSE46606\_UNSTIM\_VS\_CD40L\_IL2\_IL5\_1DAY\_STIMULATED\_IRF4HIGH\_SORTED\_BCELL\_DN | MSigDB lists | GSE46606\_UNSTIM\_VS\_CD40L\_IL2\_IL5\_1DAY\_STIMULATED\_IRF4HIGH\_SORTED\_BCELL\_DN | 160 | 20 | 12187 | 179 | Errfi1,Cxcl16,Plek,Zfp36,Icam1,Ptgs2,Ier3,Nfkbiz,Socs3,Cdkn1a,Gem,Il6,Tnfaip3,Bach1,Nfkbia,Trib1,Ccl4,Maff,Map3k8,Tnf | | 1.887e-13 | -29.30 | GO\_POSITIVE\_REGULATION\_OF\_RESPONSE\_TO\_STIMULUS | MSigDB lists | GO\_POSITIVE\_REGULATION\_OF\_RESPONSE\_TO\_STIMULUS | 1397 | 57 | 12187 | 179 | Hspb1,Plaur,Ptgs2,Tnip1,Ifi207,Tnip3,Atf3,Socs3,Xdh,C3ar1,C5ar1,Birc3,Tnf,Nfkbia,Pik3ap1,Irak3,Il17ra,Selp,Cd14,Gadd45g,Akap12,Il1a,Nfkb1,Thbs1,Osmr,Csf3,Pik3r5,Rhoc,Nfe2l2,Il6,Ccl12,Map3k8,Ccl4,Plek,Icam1,Trim25,Saa1,Ifi204,Ksr1,Ifi209,Tnfsf10,Cflar,Fzd4,Irf7,S100a9,Ptpn1,Map3k6,Sox11,S100a8,Cxcl1,Ifi211,Gadd45b,Ripk1,Tnfaip3,Il4ra,Pik3r1,Ccl2 | | 2.083e-13 | -29.20 | negative regulation of multi-organism process | biological process | GO:0043901 | 170 | 21 | 13711 | 214 | Ifitm2,Timp1,Isg15,Tap1,A2m,Ccl4,Ifitm6,Resf1,Trim25,Trim56,Tnf,Tnfaip3,Acod1,Oasl2,Zfp36,Trib1,Parp14,Irak3,Ifitm3,Oasl1,Lgals9 | | 2.360e-13 | -29.08 | GSE36891\_UNSTIM\_VS\_POLYIC\_TLR3\_STIM\_PERITONEAL\_MACROPHAGE\_UP | MSigDB lists | GSE36891\_UNSTIM\_VS\_POLYIC\_TLR3\_STIM\_PERITONEAL\_MACROPHAGE\_UP | 107 | 17 | 12187 | 179 | Nfkbiz,Adamts1,Rasd1,Atf3,Ier3,Cebpd,Akap12,Ptgs2,Apold1,Zfp36,Sdc4,Maff,Ccl12,Tnfaip3,Il6,Cxcl1,Adamts9 | | 2.610e-13 | -28.97 | GSE21927\_SPLEEN\_C57BL6\_VS\_4T1\_TUMOR\_BALBC\_MONOCYTES\_DN | MSigDB lists | GSE21927\_SPLEEN\_C57BL6\_VS\_4T1\_TUMOR\_BALBC\_MONOCYTES\_DN | 163 | 20 | 12187 | 179 | Herc6,Isg15,Ifitm2,Ifitm3,Irf9,Ifit3b,Ifi44,Ifi209,Phf11b,Ifi207,Ifi204,Irf7,Phf11d,Ifi211,Rtp4,Usp18,Xaf1,Ifit2,Oasl1,Parp12 | | 2.808e-13 | -28.90 | GROSS\_HYPOXIA\_VIA\_ELK3\_DN | MSigDB lists | GROSS\_HYPOXIA\_VIA\_ELK3\_DN | 144 | 19 | 12187 | 179 | Nfkbia,Ccl12,Il6,Csf3,Cxcl1,Ifi211,S100a8,Aff1,Ifi209,Hspa5,Ifi204,Mmp8,Akap12,Ifi207,Atf3,Ptgs2,Zfp36,Plek,Errfi1 | | 2.935e-13 | -28.86 | GSE37605\_C57BL6\_VS\_NOD\_FOXP3\_FUSION\_GFP\_TREG\_DN | MSigDB lists | GSE37605\_C57BL6\_VS\_NOD\_FOXP3\_FUSION\_GFP\_TREG\_DN | 164 | 20 | 12187 | 179 | Cd14,Ptgs2,Plaur,Apold1,Zfp36,Nfkbiz,Adamts1,Cebpd,Atf3,Ier3,Cp,Il1a,Akap12,C3ar1,Sdc4,Sntb2,Maff,Tnfaip3,Ccl12,Il6 | | 2.935e-13 | -28.86 | GSE10325\_MYELOID\_VS\_LUPUS\_MYELOID\_DN | MSigDB lists | GSE10325\_MYELOID\_VS\_LUPUS\_MYELOID\_DN | 164 | 20 | 12187 | 179 | Xaf1,Usp18,Ifit2,Ccl12,Oasl1,Parp12,Ifi211,Irf7,Igsf6,Rtp4,Ifit3b,Ifi209,Ifi44,Ifi204,Il1rn,Ifi207,Isg15,Herc6,Tap1,Plek | | 3.191e-13 | -28.77 | GSE10325\_CD4\_TCELL\_VS\_LUPUS\_CD4\_TCELL\_DN | MSigDB lists | GSE10325\_CD4\_TCELL\_VS\_LUPUS\_CD4\_TCELL\_DN | 145 | 19 | 12187 | 179 | Ifit3b,Ifi44,Ifi209,Phf11b,Ifi207,Ifi204,Isg15,Herc6,Selp,Tap1,Usp18,Xaf1,Oasl1,Parp12,Irf7,Arid5b,Phf11d,Ifi211,Rtp4 | | 3.298e-13 | -28.74 | GSE19401\_NAIVE\_VS\_IMMUNIZED\_MOUSE\_PLN\_FOLLICULAR\_DC\_UP | MSigDB lists | GSE19401\_NAIVE\_VS\_IMMUNIZED\_MOUSE\_PLN\_FOLLICULAR\_DC\_UP | 165 | 20 | 12187 | 179 | Ifit3b,Dnajb1,Cdkn1a,Thbs1,Ifi44,Atf3,Tiparp,Nfkb1,Trim25,Icam1,P2ry6,Zfp36,Ccl4,Tnf,Oasl1,Cxcl1,S100a9,Bcl3,Gem,S100a8 | | 3.298e-13 | -28.74 | GSE44649\_WT\_VS\_MIR155\_KO\_NAIVE\_CD8\_TCELL\_DN | MSigDB lists | GSE44649\_WT\_VS\_MIR155\_KO\_NAIVE\_CD8\_TCELL\_DN | 165 | 20 | 12187 | 179 | Irf7,Gbp6,Gbp4,Gadd45b,Oasl1,Parp12,Usp18,Sdc4,Nfkbia,Ccnd2,Pik3r1,Clic4,Slfn5,Tagln2,Ifitm2,Saa1,Isg15,Il1rn,Socs3,Cdkn1a | | 4.108e-13 | -28.52 | GSE9960\_GRAM\_NEG\_VS\_GRAM\_POS\_SEPSIS\_PBMC\_UP | MSigDB lists | GSE9960\_GRAM\_NEG\_VS\_GRAM\_POS\_SEPSIS\_PBMC\_UP | 147 | 19 | 12187 | 179 | Xaf1,Usp18,Trib1,Cflar,Dtx3l,Bcl3,Irf7,Tnfaip8,Cdkn1a,Ifi44,Ifit3b,Samd9l,Ifitm3,Ifitm2,Tagln2,Icam1,Pik3ap1,Plaur,Csf2rb | | 4.108e-13 | -28.52 | GSE8835\_CD4\_VS\_CD8\_TCELL\_CLL\_PATIENT\_UP | MSigDB lists | GSE8835\_CD4\_VS\_CD8\_TCELL\_CLL\_PATIENT\_UP | 147 | 19 | 12187 | 179 | Oasl1,Gbp4,Usp18,Ccl2,Ifit2,Tnfsf10,Cmpk2,Rtp4,Irf7,Gbp6,Irgm2,Samd9l,Stx11,Ifit3b,Hspa5,Slfn9,Herc6,Isg15,Rnf213 | | 4.150e-13 | -28.51 | positive regulation of defense response | biological process | GO:0031349 | 240 | 24 | 13711 | 214 | Nfkbia,Il6,Irf7,Lgals9,Ifi209,Parp9,Irgm1,Ifi211,Il17ra,Ptgs2,Pik3ap1,Tnf,Cd14,Irak3,Ifi204,Tnip1,Cxcl1,Tgm2,Nfkbiz,S100a8,Tnip3,S100a9,Mmp8,Irgm2 | | 4.279e-13 | -28.48 | response to oxygen-containing compound | biological process | GO:1901700 | 969 | 48 | 13711 | 214 | Pygm,Irak3,Il1rn,Lcn2,Tnfaip3,Cxcl9,Ccl2,Vcam1,Gbp6,Irgm2,Wfdc21,Ptpn1,Col4a1,Tnip3,Cxcl16,Ccl12,Thbs1,P2ry6,Lgals9,Icam1,Mt1,Nfkb1,Ncf1,Ripk1,Cflar,Cd14,Acod1,Tnf,Zfp36,Nfe2l2,Serpina3f,Msn,Noct,C5ar1,Cxcl1,Hp,Trib1,Ccl7,Pik3r1,Casp4,Nfkbia,Il6,Ip6k2,Runx1,Ptgs2,Sgk1,Cmpk2,Sbno2 | | 4.654e-13 | -28.40 | GSE3982\_CTRL\_VS\_LPS\_48H\_DC\_DN | MSigDB lists | GSE3982\_CTRL\_VS\_LPS\_48H\_DC\_DN | 148 | 19 | 12187 | 179 | Irf7,Cflar,Ccl4,Nfkbia,Trib1,Tnfaip3,Parp12,Gadd45b,Tnip1,Ifitm3,Ifitm2,Isg15,Plaur,Irf9,P2ry6,Ifit3b,Ifi44,Atf3,Gadd45g | | 5.496e-13 | -28.23 | leukocyte migration | biological process | GO:0050900 | 140 | 19 | 13711 | 214 | Vcam1,S100a9,Ccl4,Selp,Msn,Il17ra,Cxcl1,C5ar1,S100a8,Ccl12,Cxcl16,Sele,Il1rn,Ccl7,Retnlg,Icam1,Tnf,Cxcl9,Ccl2 | | 5.955e-13 | -28.15 | GSE6269\_FLU\_VS\_STREP\_PNEUMO\_INF\_PBMC\_UP | MSigDB lists | GSE6269\_FLU\_VS\_STREP\_PNEUMO\_INF\_PBMC\_UP | 150 | 19 | 12187 | 179 | Xaf1,Usp18,Parp12,Oasl1,Phf11d,Ifi211,Irf7,Rtp4,Phf11b,Ifi209,Ifi44,Ifit3b,Ifi204,Ifi207,Ifitm3,Isg15,Ifitm2,Herc6,Irf9 | | 6.447e-13 | -28.07 | GO\_POSITIVE\_REGULATION\_OF\_CYTOKINE\_PRODUCTION | MSigDB lists | GO\_POSITIVE\_REGULATION\_OF\_CYTOKINE\_PRODUCTION | 262 | 24 | 12187 | 179 | Ripk1,Ccl12,Il6,Pik3r1,Il4ra,Ifi211,Bcl3,Nfkb1,Il1a,Thbs1,Il17ra,Cd14,Runx1,Tnf,Nfkb2,Irf7,C5ar1,C3ar1,Ifi204,Ifi207,Ifi209,Hspb1,Saa1,Ptgs2 | | 6.656e-13 | -28.04 | cytokine-mediated signaling pathway | biological process | GO:0019221 | 201 | 22 | 13711 | 214 | Ccl12,Cxcl1,Ifitm2,Osmr,Fzd4,Csf2rb,Ifitm6,Spi1,Ccl4,Il6,Ccl2,Tnf,Il4ra,Il1a,Cxcl9,Nfkbia,Irf7,Il1rn,Irak3,Ifitm3,Iigp1,Ccl7 | | 6.726e-13 | -28.03 | GSE39382\_IL3\_VS\_IL3\_IL33\_TREATED\_MAST\_CELL\_DN | MSigDB lists | GSE39382\_IL3\_VS\_IL3\_IL33\_TREATED\_MAST\_CELL\_DN | 151 | 19 | 12187 | 179 | Il1rn,Stx11,Nfkb1,Gadd45g,Icam1,Ifitm3,Isg15,Herc6,Tnip1,Tagln2,Tnfaip3,Ifit2,Il4ra,Birc3,Usp18,Sbno2,Nfkb2,Cflar,Irf7 | | 6.726e-13 | -28.03 | GSE36826\_WT\_VS\_IL1R\_KO\_SKIN\_STAPH\_AUREUS\_INF\_DN | MSigDB lists | GSE36826\_WT\_VS\_IL1R\_KO\_SKIN\_STAPH\_AUREUS\_INF\_DN | 151 | 19 | 12187 | 179 | Socs3,Fstl1,Phf11b,Ptpn1,Tap1,Hspb1,Icam1,Ifitm2,Herc6,Isg15,Tnfaip3,Gadd45b,Oasl1,Tgm2,Usp18,Tnf,Rtp4,Phf11d,Cflar | | 7.591e-13 | -27.91 | GSE36888\_UNTREATED\_VS\_IL2\_TREATED\_STAT5\_AB\_KNOCKIN\_TCELL\_2H\_UP | MSigDB lists | GSE36888\_UNTREATED\_VS\_IL2\_TREATED\_STAT5\_AB\_KNOCKIN\_TCELL\_2H\_UP | 152 | 19 | 12187 | 179 | Tnf,Gpr84,Ccl4,Ccl12,AA467197,Tnfaip3,S100a9,Pik3r5,Cxcl1,Tnfaip8,Nfkb2,Nfkbiz,Socs3,Il1a,Tnip3,Tnip1,Icam1,Errfi1,Plaur | | 7.792e-13 | -27.88 | GO\_REGULATION\_OF\_INTRACELLULAR\_SIGNAL\_TRANSDUCTION | MSigDB lists | GO\_REGULATION\_OF\_INTRACELLULAR\_SIGNAL\_TRANSDUCTION | 1271 | 53 | 12187 | 179 | Selp,Pik3ap1,Irak3,Thbs1,Gadd45g,Il1a,Akap12,Csf3,Rhoj,Pik3r5,Banp,Map3k8,Ccl4,Rhoc,Nfe2l2,Il6,Ccl12,Ptgs2,Tnip1,Hspb1,Errfi1,Plaur,Socs3,Tnip3,Atf3,C5ar1,A2m,Xdh,Birc3,Tnf,Nfkbia,Map3k6,Ptpn1,Sox11,Ier3,Bcl3,S100a8,Pik3r1,Rhou,Ccl2,Gadd45b,Ripk1,Tnfaip3,Saa1,Plek,Icam1,Trim25,Ksr1,Cflar,Fzd4,S100a9,Tnfsf10,Trib1 | | 7.839e-13 | -27.87 | negative regulation of biological process | biological process | GO:0048519 | 4173 | 115 | 13711 | 214 | Fam107a,Tiparp,Timp1,Isg15,Slfn2,Xaf1,Ifi211,Rasip1,Cebpd,Trim25,Resf1,Runx1,Nfkbia,Il4ra,Slfn3,Ip6k2,Parp14,Ccnd2,Hcar2,Hspa5,Clic4,C5ar1,Rasd1,Mmp8,Nfe2l2,Serpina3f,Angptl4,Noct,Pik3ap1,Acod1,Atf3,Ncf1,Trim30a,Rnf213,Birc3,Nfkbie,Rgs16,Icam1,Mt1,Bbs12,Msr1,Oasl1,Lgals9,Gadd45g,Ifitm2,Ptpn1,Iqgap1,Tnip3,Wfdc21,Znfx1,Tnfaip3,Ccl2,Csf3,Tnfaip8,Zbtb16,Il1rn,Fzd4,Ifi209,Parp9,Sbno2,Ptges,Sdc4,Spi1,Ccl4,Ifitm6,Ier3,Sgk1,Ptgs2,Gem,Il1a,Oasl2,Cdkn1a,Il6,Trib1,Ctla2a,Dtx3l,Socs3,Gadd45b,Pik3r1,Rrp8,Dnajb1,Hp,Prg4,Tnf,Robo4,Zfp36,Ripk1,Cflar,Ifi207,Hspb1,Tnip1,Gbp4,Adamts1,Nfkb1,Kcna5,Bach1,Plaur,Trim56,Sox11,Mxd4,Errfi1,Thbs1,Ifitm3,Banp,Ccl12,Tap1,A2m,Sgk3,Bcl3,Plek,Xdh,Adamts9,Irak3,Arid5b,Ifi204 | | 8.143e-13 | -27.84 | GSE15930\_STIM\_VS\_STIM\_AND\_IFNAB\_48H\_CD8\_T\_CELL\_DN | MSigDB lists | GSE15930\_STIM\_VS\_STIM\_AND\_IFNAB\_48H\_CD8\_T\_CELL\_DN | 173 | 20 | 12187 | 179 | Ifit3b,Znfx1,Ncf1,Ifitm3,Isg15,Clic4,Tap1,Irf9,Slfn3,Tgm2,Casp4,Usp18,Ifit2,Map3k8,Lgals9,Irf7,Irgm2,Nfkb2,Slfn4,Cmpk2 | | 9.754e-13 | -27.66 | GO\_RESPONSE\_TO\_TYPE\_I\_INTERFERON | MSigDB lists | GO\_RESPONSE\_TO\_TYPE\_I\_INTERFERON | 44 | 12 | 12187 | 179 | Isg15,Ifitm2,Ifitm3,Ip6k2,Irf9,Irf2,Ifit3b,Trim56,Irf7,Ifit2,Xaf1,Oasl1 | | 1.006e-12 | -27.63 | extracellular region part | cellular component | GO:0044421 | 1048 | 49 | 13825 | 212 | Prg4,A2m,Csf3,Serpina3f,Timp1,Icam1,Wfdc21,Il1a,Gbp7,Sele,Vcam1,Olfml2b,Cp,Cxcl9,Cxcl16,Msr1,Thbs1,Tnf,Ccl2,Lcn2,Ccl4,Retnlg,Adamts1,S100a8,Gbp6,S100a9,Tnfsf10,Il6,Ccl12,Hp,Fstl1,Lgals9,Ccl7,Mmp8,Xdh,Irgm2,Runx1,Gbp3,Col4a1,Angptl4,Adamts9,Il1rn,Cd14,Cxcl1,Iigp1,Tgm2,Selp,Il4ra,Saa1 | | 1.042e-12 | -27.59 | regulation of response to biotic stimulus | biological process | GO:0002831 | 274 | 25 | 13711 | 214 | Irgm2,Cxcl1,Tnip3,A2m,Tap1,Cd14,Irak3,Trim30a,Ifi204,Tnip1,Pik3ap1,Tnfaip3,Tnf,Acod1,Ifi211,Irgm1,Ifi209,Gbp4,Parp9,Parp14,Trib1,Irf7,Dtx3l,Lgals9,Nfkbia | | 1.071e-12 | -27.56 | VERHAAK\_AML\_WITH\_NPM1\_MUTATED\_UP | MSigDB lists | VERHAAK\_AML\_WITH\_NPM1\_MUTATED\_UP | 117 | 17 | 12187 | 179 | Tnfsf10,Nfkb2,Cxcl1,C3ar1,C5ar1,Il6,Tnfaip3,Trib1,Nfkbia,Ccl4,Tnf,Runx1,Plaur,Plek,Cd14,Ier3,Thbs1 | | 1.071e-12 | -27.56 | GERY\_CEBP\_TARGETS | MSigDB lists | GERY\_CEBP\_TARGETS | 117 | 17 | 12187 | 179 | Gadd45b,Ifi211,Angptl4,Slfn4,S100a8,Xdh,Rgs16,Ifi209,Dnajb1,Ifi204,Gadd45g,Hp,Ifi207,Atf3,Lcn2,Slfn3,Plaur | | 1.071e-12 | -27.56 | GSE7218\_IGM\_VS\_IGG\_SIGNAL\_THGOUGH\_ANTIGEN\_BCELL\_DN | MSigDB lists | GSE7218\_IGM\_VS\_IGG\_SIGNAL\_THGOUGH\_ANTIGEN\_BCELL\_DN | 117 | 17 | 12187 | 179 | Parp12,Gbp4,Usp18,Parp14,Birc3,Ifit2,Rgs16,Tnfsf10,Rtp4,Nfkbie,Gbp6,Gem,Lcn2,Ifit3b,Ifi44,Icam1,Hspb1 | | 1.184e-12 | -27.46 | WONG\_ADULT\_TISSUE\_STEM\_MODULE | MSigDB lists | WONG\_ADULT\_TISSUE\_STEM\_MODULE | 594 | 35 | 12187 | 179 | Cxcl1,Pygm,Tgm2,Rhou,Il6,Hcar2,Selp,Ifitm3,Isg15,Nfkbiz,Thbs1,Ier3,Cebpd,Gadd45g,Irf7,Gem,Nfkbia,Casp4,Tubb6,Bach1,Ptgs2,Errfi1,Clic4,Msn,Icam1,Plek,Vcam1,Fstl1,Socs3,Adamts1,Cdkn1a,Ifi44,Rasd1,Atf3,Col4a1 | | 1.220e-12 | -27.43 | GSE13485\_DAY7\_VS\_DAY21\_YF17D\_VACCINE\_PBMC\_UP | MSigDB lists | GSE13485\_DAY7\_VS\_DAY21\_YF17D\_VACCINE\_PBMC\_UP | 156 | 19 | 12187 | 179 | Parp14,Xaf1,Usp18,Oasl1,Parp12,Phf11d,Irf7,Dtx3l,Ifit3b,Phf11b,Cdkn1a,Znfx1,Ksr1,Samd9l,Isg15,Herc6,Parp9,Trim25,Pik3ap1 | | 1.313e-12 | -27.36 | GAURNIER\_PSMD4\_TARGETS | MSigDB lists | GAURNIER\_PSMD4\_TARGETS | 45 | 12 | 12187 | 179 | Cxcl1,C3ar1,C5ar1,Nfkb2,Cxcl16,Il17ra,Ccl2,Tnf,Il6,Ccl12,Il1a,Nfkb1 | | 1.321e-12 | -27.35 | GSE36888\_UNTREATED\_VS\_IL2\_TREATED\_TCELL\_6H\_UP | MSigDB lists | GSE36888\_UNTREATED\_VS\_IL2\_TREATED\_TCELL\_6H\_UP | 137 | 18 | 12187 | 179 | Ptgs2,Cd14,Pik3ap1,P2ry6,Nfkbiz,Socs3,Tnip3,Ksr1,Ier3,C5ar1,S100a9,Ptges,S100a8,Nfkb2,Il4ra,Runx1,Trib1,Slc24a4 | | 1.370e-12 | -27.32 | GSE37605\_FOXP3\_FUSION\_GFP\_VS\_IRES\_GFP\_TREG\_C57BL6\_UP | MSigDB lists | GSE37605\_FOXP3\_FUSION\_GFP\_VS\_IRES\_GFP\_TREG\_C57BL6\_UP | 157 | 19 | 12187 | 179 | Cxcl1,Gem,Sdc4,Sntb2,Maff,Ccl12,Ptgs2,Plaur,Zfp36,Apold1,Trim25,Sgk1,Thbs1,Adamts1,Atf3,Ier3,Cebpd,Il1a,Akap12 | | 1.370e-12 | -27.32 | GSE43955\_TH0\_VS\_TGFB\_IL6\_TH17\_ACT\_CD4\_TCELL\_1H\_UP | MSigDB lists | GSE43955\_TH0\_VS\_TGFB\_IL6\_TH17\_ACT\_CD4\_TCELL\_1H\_UP | 157 | 19 | 12187 | 179 | Csf2rb,Errfi1,Ptpn1,Sele,Il17ra,Saa1,Selp,Cebpd,Rasd1,Gadd45g,Il1rn,Socs3,Gbp3,Il6,Bach1,Ccl4,Il4ra,Birc3,Map3k8 | | 1.424e-12 | -27.28 | STK33\_UP | MSigDB lists | STK33\_UP | 223 | 22 | 12187 | 179 | Samd9l,Il1a,Ifi207,Ifi204,Sgk1,Cdkn1a,Ifi209,Zfp36,Icam1,Irf9,Bbs12,Rhoc,AA467197,Maff,Tnf,Trib1,Ccl4,C5ar1,Bcl3,Arid5b,Cxcl1,Ifi211 | | 1.470e-12 | -27.25 | CHEN\_METABOLIC\_SYNDROM\_NETWORK | MSigDB lists | CHEN\_METABOLIC\_SYNDROM\_NETWORK | 963 | 45 | 12187 | 179 | Tagln2,Plek,Ifi209,Col4a1,Ifi204,Bach1,Oasl1,P2ry6,Ptpn1,Ms4a6d,Ifi211,Cxcl1,S100a8,Tnfaip8,Ifit2,Spi1,Gadd45b,Tnip1,Plaur,Ifit3b,Vcam1,Ifi207,Tnip3,Atf3,C3ar1,Igsf6,Slfn4,Nfkb2,Tnf,Tubb6,Cd14,Slfn3,Pik3ap1,Cxcl16,Phf11b,Rhoj,Phf11d,Msr1,Map3k8,Ccl4,Rassf4,Rhoc,Cybb,Nfe2l2,Ccl12 | | 1.500e-12 | -27.23 | GSE18791\_CTRL\_VS\_NEWCASTLE\_VIRUS\_DC\_16H\_DN | MSigDB lists | GSE18791\_CTRL\_VS\_NEWCASTLE\_VIRUS\_DC\_16H\_DN | 138 | 18 | 12187 | 179 | Oasl1,Tnf,Ifit2,Xaf1,Parp14,Cmpk2,Rtp4,Gbp3,Irf7,Phf11d,Ifi44,Phf11b,Ifit3b,Trim25,Ptgs2,Isg15,Herc6,Slfn5 | | 1.766e-12 | -27.06 | ICHIBA\_GRAFT\_VERSUS\_HOST\_DISEASE\_35D\_UP | MSigDB lists | ICHIBA\_GRAFT\_VERSUS\_HOST\_DISEASE\_35D\_UP | 103 | 16 | 12187 | 179 | Gbp3,Iqgap1,Slfn4,S100a8,S100a9,Irgm2,Ccl12,Cybb,Ccl4,Msr1,Ptpn1,Tap1,Slfn3,Cd14,Vcam1,Nfkbiz | | 1.928e-12 | -26.97 | GSE9988\_ANTI\_TREM1\_AND\_LPS\_VS\_CTRL\_TREATED\_MONOCYTES\_UP | MSigDB lists | GSE9988\_ANTI\_TREM1\_AND\_LPS\_VS\_CTRL\_TREATED\_MONOCYTES\_UP | 140 | 18 | 12187 | 179 | Ptgs2,Plaur,Errfi1,Sgk1,Cdkn1a,Nfkbiz,Socs3,Il1a,Ier3,Csf3,Gem,Cflar,Banp,Cxcl1,Map3k8,Tnf,Gadd45b,Il6 | | 2.164e-12 | -26.86 | GSE19888\_ADENOSINE\_A3R\_ACT\_VS\_A3R\_ACT\_WITH\_A3R\_INH\_PRETREATMENT\_IN\_MAST\_CELL\_DN | MSigDB lists | GSE19888\_ADENOSINE\_A3R\_ACT\_VS\_A3R\_ACT\_WITH\_A3R\_INH\_PRETREATMENT\_IN\_MAST\_CELL\_DN | 161 | 19 | 12187 | 179 | Gbp4,Usp18,Parp14,Tor3a,Xaf1,Lgals9,Tnfsf10,Cmpk2,Slfn4,Irf7,Gbp6,Irgm2,Ifit3b,Slfn3,Ip6k2,Saa1,Rnf213,Isg15,Herc6 | | 2.209e-12 | -26.84 | GO\_RESPONSE\_TO\_INTERFERON\_GAMMA | MSigDB lists | GO\_RESPONSE\_TO\_INTERFERON\_GAMMA | 88 | 15 | 12187 | 179 | Irf7,Gbp6,Ccl2,Ccl4,Oasl1,Gbp4,Ccl12,Ifitm2,Ifitm3,Irf9,Cxcl16,Irf2,Trim25,Icam1,Vcam1 | | 2.318e-12 | -26.79 | IL-17 signaling pathway | KEGG pathways | mmu04657 | 67 | 15 | 5248 | 107 | Cxcl1,Ccl12,Tnfaip3,Ccl7,S100a8,Ccl2,Nfkbia,S100a9,Il17ra,Lcn2,Csf3,Ptgs2,Tnf,Il6,Nfkb1 | | 2.318e-12 | -26.79 | IL-17 signaling pathway | KEGG pathways | ko04657 | 67 | 15 | 5248 | 107 | Il6,Nfkb1,Tnf,Ptgs2,Csf3,Lcn2,Nfkbia,S100a9,Il17ra,Ccl7,S100a8,Ccl2,Tnfaip3,Ccl12,Cxcl1 | | 2.356e-12 | -26.77 | BOYLAN\_MULTIPLE\_MYELOMA\_C\_D\_DN | MSigDB lists | BOYLAN\_MULTIPLE\_MYELOMA\_C\_D\_DN | 183 | 20 | 12187 | 179 | Ifi209,Vcam1,Zbtb16,Ifi204,Mmp8,Cp,Ifi207,Hp,Lcn2,Ifitm3,Plek,Ccnd2,Casp4,AA467197,Ifi211,S100a9,Igsf6,Rtp4,S100a8,Xdh | | 2.420e-12 | -26.75 | GSE32034\_LY6C\_HIGH\_VS\_LOW\_ROSIGLIZATONE\_TREATED\_MONOCYTE\_DN | MSigDB lists | GSE32034\_LY6C\_HIGH\_VS\_LOW\_ROSIGLIZATONE\_TREATED\_MONOCYTE\_DN | 162 | 19 | 12187 | 179 | Bcl3,Rgs16,Ccl2,Casp4,Ccl4,Map3k8,Ccl12,Bach1,Ifitm3,Ptpn1,Errfi1,Zfp36,Il17ra,Nfkbiz,Aff1,Cebpd,Gadd45g,Tiparp,Timp1 | | 2.493e-12 | -26.72 | LINDGREN\_BLADDER\_CANCER\_CLUSTER\_2B | MSigDB lists | LINDGREN\_BLADDER\_CANCER\_CLUSTER\_2B | 305 | 25 | 12187 | 179 | Nfkbie,Adamts9,Ccl2,Cybb,Ccl12,Tnfaip3,Gadd45b,Ifitm3,Nfkbiz,Il1a,Akap12,Gem,C3ar1,A2m,Birc3,Nfkbia,Ifitm2,Icam1,Sele,Plaur,Clic4,Msn,Aff1,Vcam1,Socs3 | | 2.551e-12 | -26.69 | response to lipid | biological process | GO:0033993 | 476 | 32 | 13711 | 214 | Cflar,Cd14,Irak3,Il1rn,Ccl2,Zfp36,Cxcl9,Acod1,Tnfaip3,Tnf,Wfdc21,Irgm2,Gbp6,Noct,Msn,Ccl12,Cxcl16,Tnip3,Cxcl1,Lgals9,P2ry6,Pik3r1,Trib1,Il6,Nfkbia,Sgk1,Ptgs2,Runx1,Sbno2,Nfkb1,Cmpk2,Fam107a | | 2.552e-12 | -26.69 | regulation of protein metabolic process | biological process | GO:0051246 | 2256 | 77 | 13711 | 214 | Spi1,Ccl4,Rasip1,Ier3,Ptgs2,Map3k6,Fzd4,Fam107a,Tiparp,Timp1,Parp9,Sdc4,Isg15,Trib1,Parp14,Ctla2a,Ccl7,Dtx3l,Socs3,Gadd45b,Ccnd2,Pik3r1,Nfkbia,Il1a,Akap12,Casp4,Cdkn1a,Il6,Mmp8,Serpina3f,Msn,Hspa5,C5ar1,Prg4,Ripk1,Cflar,Ncf1,Hspb1,Trim30a,Tnip1,Pik3ap1,Tnf,Atf3,Zfp36,Plaur,Pik3r5,Birc3,Map3k8,Gbp4,Nfkb1,Thbs1,P2ry6,Lgals9,Gadd45g,Banp,Icam1,Cybb,Errfi1,S100a9,Irgm2,Wfdc21,Samd9l,Bcl3,Ptpn1,Ccl12,S100a8,Iqgap1,A2m,Tnfaip8,Csf3,Il1rn,Irak3,Tnfaip3,Ccl2,Xdh,Ksr1,Tnfsf10 | | 2.631e-12 | -26.66 | GO\_RESPONSE\_TO\_LIPID | MSigDB lists | GO\_RESPONSE\_TO\_LIPID | 645 | 36 | 12187 | 179 | Tnf,Nfkbia,Trib1,C5ar1,Fzd4,Nfkb2,Adamts1,Cdkn1a,Vcam1,Socs3,Tnip3,Il1rn,Ptgs2,Sele,Icam1,Trim25,Msn,Errfi1,Il4ra,Il6,Ccl12,Tnfaip3,Csf3,Cxcl1,Ptges,Cmpk2,S100a8,Sbno2,Thbs1,Sgk1,Nfkb1,Selp,Cd14,P2ry6,Irak3,Cxcl16 | | 2.663e-12 | -26.65 | STK33\_NOMO\_UP | MSigDB lists | STK33\_NOMO\_UP | 230 | 22 | 12187 | 179 | Zfp36,Icam1,Bbs12,Samd9l,Ifi207,Il1a,Ifi204,Ier3,Sgk1,Ifi209,C3ar1,C5ar1,A2m,Arid5b,Bcl3,Cxcl1,Ifi211,AA467197,Maff,Map3k8,Sdc4,Ccl4 | | 3.040e-12 | -26.52 | STK33\_SKM\_UP | MSigDB lists | STK33\_SKM\_UP | 208 | 21 | 12187 | 179 | Tnf,Ccl4,Nfkbia,Trib1,Rhoc,Cybb,C5ar1,Ifi211,Bcl3,Arid5b,Ifi209,Cdkn1a,Sgk1,Nfkbiz,Ifi204,Il1a,Ifi207,Bbs12,Zfp36,Errfi1,Irf9 | | 3.062e-12 | -26.51 | REACTOME\_INTERFERON\_GAMMA\_SIGNALING | MSigDB lists | REACTOME\_INTERFERON\_GAMMA\_SIGNALING | 48 | 12 | 12187 | 179 | Gbp3,Irf7,Gbp6,Gbp7,Gbp4,Oasl1,Irf2,Ptpn1,Irf9,Icam1,Socs3,Vcam1 | | 3.062e-12 | -26.51 | LINDSTEDT\_DENDRITIC\_CELL\_MATURATION\_A | MSigDB lists | LINDSTEDT\_DENDRITIC\_CELL\_MATURATION\_A | 48 | 12 | 12187 | 179 | Plek,Icam1,Plaur,Isg15,Nfkbie,Cxcl1,Il1rn,Il6,Ccl12,Ifit2,Tnf,Ccl4 | | 3.145e-12 | -26.49 | JAATINEN\_HEMATOPOIETIC\_STEM\_CELL\_DN | MSigDB lists | JAATINEN\_HEMATOPOIETIC\_STEM\_CELL\_DN | 144 | 18 | 12187 | 179 | S100a9,Pik3r5,C5ar1,S100a8,Igsf6,Sntb2,Ccl4,Tnfaip3,Cybb,Cd14,Cxcl16,Thbs1,Sgk1,Cebpd,Ms4a6d,Il1rn,Stx11,Mmp8 | | 3.204e-12 | -26.47 | signal transduction | biological process | GO:0007165 | 2703 | 86 | 13711 | 214 | Pik3r1,Dtx3l,Irf7,Socs3,Trib1,Il6,Cdkn1a,Casp4,Gem,Il1a,Sgk1,Ier3,Ifitm6,Ccl4,Spi1,Fzd4,Tnip1,Rhoc,Hspb1,Ripk1,Cd14,Gpr84,Zfp36,Tnf,Csf2rb,Hp,Mt2,Cxcl1,Rrp8,P2ry6,Ifitm3,Cybb,Errfi1,Plaur,Kcna5,Nfkb1,Osmr,Ifi204,Arid5b,Irak3,Ksr1,Plek,Bcl3,Irgm2,Sgk3,Ccl12,Hcar2,Ccl7,Il4ra,Nfkbia,Akap12,Rasip1,Isg15,Tiparp,Map3k6,Atf3,Rhou,Pik3ap1,Selp,Nfe2l2,Rasd1,C5ar1,Tgm2,Hspa5,Lgals9,Iigp1,Rassf4,Mt1,Fyb,Nfkb2,Rgs16,Pik3r5,Rhoj,Map3k8,Lcn2,Il1rn,C3ar1,Ccl2,Cxcl9,Vcam1,Col4a1,Iqgap1,Tnip3,Nfkbiz,Ifitm2,Ptpn1 | | 3.352e-12 | -26.42 | MODULE\_170 | MSigDB lists | MODULE\_170 | 75 | 14 | 12187 | 179 | Cybb,Ccl12,Ccnd2,Tnfsf10,C3ar1,A2m,Timp1,Cebpd,Ier3,Thbs1,Vcam1,Ifitm2,Ifitm3,Cd14 | | 3.674e-12 | -26.33 | regulation of response to stimulus | biological process | GO:0048583 | 2962 | 91 | 13711 | 214 | Il6,Cdkn1a,Casp4,Il1a,Pik3r1,Gadd45b,Irf7,Socs3,Dtx3l,Ctla2a,Trib1,Sbno2,Parp9,Ifi209,Fzd4,Ptgs2,Ier3,Ccl4,Zfp36,Tnf,Tnip1,Fstl1,Hspb1,Cd14,Cflar,Ripk1,Cxcl1,Errfi1,Sox11,P2ry6,Thbs1,Nfkb1,Gbp4,Plaur,Il17ra,Ksr1,Tnfsf10,Xdh,Plek,Ifi204,Irak3,A2m,S100a8,Tap1,Ccl12,Bcl3,Irgm2,Sgk3,Usp18,Il4ra,Akap12,Nfkbia,Ccl7,Parp14,Timp1,Map3k6,Runx1,Trim25,Rasip1,Ifi211,Atf3,Acod1,Pik3ap1,Trim30a,Rnf213,Ncf1,Tgm2,C5ar1,Hspa5,Selp,Nfe2l2,Mmp8,Icam1,Rgs16,Fyb,Gadd45g,Lgals9,Map3k8,Birc3,Pik3r5,Irgm1,Ccl2,Tnfaip3,Il1rn,C3ar1,Csf3,Tnip3,Iqgap1,Nfkbiz,Ptpn1,S100a9 | | 3.759e-12 | -26.31 | GSE7219\_UNSTIM\_VS\_LPS\_AND\_ANTI\_CD40\_STIM\_NIK\_NFKB2\_KO\_DC\_DN | MSigDB lists | GSE7219\_UNSTIM\_VS\_LPS\_AND\_ANTI\_CD40\_STIM\_NIK\_NFKB2\_KO\_DC\_DN | 166 | 19 | 12187 | 179 | Tnfsf10,Rgs16,Nfkbie,Gadd45b,Tnf,Nfkbia,Trib1,Ccl4,Zfp36,Pik3ap1,Icam1,Clic4,Rnf213,Ifitm2,Ifitm3,Atf3,Ifi44,Cdkn1a,Dnajb1 | | 3.993e-12 | -26.25 | GSE360\_HIGH\_DOSE\_B\_MALAYI\_VS\_M\_TUBERCULOSIS\_DC\_DN | MSigDB lists | GSE360\_HIGH\_DOSE\_B\_MALAYI\_VS\_M\_TUBERCULOSIS\_DC\_DN | 146 | 18 | 12187 | 179 | Timp1,Il1rn,Nfkb1,Stx11,Ier3,Ifi44,Vcam1,Icam1,Csf2rb,Cybb,Gadd45b,Ccl12,Ifit2,Nfkbia,Ccl4,S100a8,Sbno2,S100a9 | | 3.993e-12 | -26.25 | GSE17721\_PAM3CSK4\_VS\_GADIQUIMOD\_1H\_BMDC\_UP | MSigDB lists | GSE17721\_PAM3CSK4\_VS\_GADIQUIMOD\_1H\_BMDC\_UP | 146 | 18 | 12187 | 179 | Cxcl1,Gem,Igsf6,Parp14,Nfkbia,Ccl2,Casp4,Birc3,Maff,Il6,Tnip1,Errfi1,Icam1,Plek,Zfp36,Fstl1,Nfkbiz,Atf3 | | 4.465e-12 | -26.13 | positive regulation of cytokine production | biological process | GO:0001819 | 318 | 26 | 13711 | 214 | C5ar1,Bcl3,Mmp8,Ccl2,Tnf,Ifi204,Ripk1,Cd14,Il1rn,C3ar1,Hspb1,Il17ra,Runx1,Ptgs2,Ccl4,Ifi211,Cybb,Il6,Il1a,Akap12,Il4ra,Casp4,Lgals9,Pik3r1,Thbs1,Irf7 | | 5.374e-12 | -25.95 | regulation of inflammatory response | biological process | GO:0050727 | 246 | 23 | 13711 | 214 | Tnip1,Pik3ap1,Acod1,Tnfaip3,Tnf,Zfp36,Usp18,S100a9,Mmp8,C5ar1,Nfkbiz,Tgm2,S100a8,Ctla2a,Socs3,Lgals9,Nfkbia,Il6,Il17ra,Ier3,Ptgs2,Sbno2,Nfkb1 | | 5.914e-12 | -25.85 | DAUER\_STAT3\_TARGETS\_UP | MSigDB lists | DAUER\_STAT3\_TARGETS\_UP | 39 | 11 | 12187 | 179 | Icam1,Zfp36,Bcl3,Ccl12,Atf3,Cebpd,Akap12,Socs3,Trib1,Thbs1,Maff | | 6.111e-12 | -25.82 | GO\_REGULATION\_OF\_CELL\_DEATH | MSigDB lists | GO\_REGULATION\_OF\_CELL\_DEATH | 1126 | 48 | 12187 | 179 | Nfkb1,Il1a,Gadd45g,Thbs1,Sgk3,Ccl12,Il6,Nfe2l2,Tgm2,Angptl4,Csf3,Atf3,Timp1,Zbtb16,Socs3,Ifit3b,Plaur,Hspb1,Ptgs2,Nfkbia,Tnf,Birc3,Xdh,C5ar1,Robo4,Ier3,Sox11,Hspa5,Sgk1,Ptpn1,Ip6k2,Tnfaip3,Ripk1,Gadd45b,Hcar2,Ifit2,Pik3r1,Tnfaip8,S100a8,Bcl3,Hp,Cdkn1a,Icam1,Casp4,Tnfsf10,Irf7,S100a9,Cflar | | 6.814e-12 | -25.71 | CHARAFE\_BREAST\_CANCER\_LUMINAL\_VS\_BASAL\_DN | MSigDB lists | CHARAFE\_BREAST\_CANCER\_LUMINAL\_VS\_BASAL\_DN | 347 | 26 | 12187 | 179 | Ptgs2,Clic4,Msn,Icam1,Zbtb16,Fstl1,Ifit3b,Ifi209,Ifi44,Ifi204,Col4a1,Ifi207,Cflar,Casp4,Birc3,Tubb6,Cd14,Sgk1,Cebpd,Il1a,Ifi211,Cxcl1,Tnfaip8,Osmr,Tnfaip3,Nfe2l2 | | 7.112e-12 | -25.67 | GSE25085\_FETAL\_BM\_VS\_ADULT\_BM\_SP4\_THYMIC\_IMPLANT\_UP | MSigDB lists | GSE25085\_FETAL\_BM\_VS\_ADULT\_BM\_SP4\_THYMIC\_IMPLANT\_UP | 172 | 19 | 12187 | 179 | Cmpk2,Rtp4,Gbp3,Irf7,Hcar2,Oasl1,Parp12,Nfe2l2,Ifit2,Sdc4,Ccl4,Parp14,Isg15,Herc6,Slfn9,Samd9l,Atf3,Ifit3b,Socs3 | | 7.113e-12 | -25.67 | extracellular region | cellular component | GO:0005576 | 1307 | 54 | 13825 | 212 | A2m,Prg4,Wfdc21,Il1a,Icam1,Timp1,Serpina3f,Csf3,Vcam1,Gbp7,Sele,Olfml2b,Tnf,Thbs1,Cxcl9,Cp,Cxcl16,Msr1,Ccl2,Ccl4,Lcn2,Retnlg,Adamts1,Gbp6,S100a8,Isg15,Ctla2a,Plaur,Il6,S100a9,Tnfsf10,Ccl12,Hp,Xdh,Mmp8,Ccl7,Lgals9,Fstl1,Irgm2,Angptl4,Gbp3,Col4a1,Runx1,Tgm2,Casp4,Sdc4,Cd14,Il1rn,Iigp1,Cxcl1,Adamts9,Selp,Il4ra,Saa1 | | 7.991e-12 | -25.55 | GSE17721\_CTRL\_VS\_LPS\_1H\_BMDC\_DN | MSigDB lists | GSE17721\_CTRL\_VS\_LPS\_1H\_BMDC\_DN | 152 | 18 | 12187 | 179 | Cflar,Arid5b,Igsf6,Maff,Map3k8,Ccl2,Tgm2,Nfe2l2,Oasl1,Zfp36,Icam1,Errfi1,Hspa5,Cdkn1a,Fstl1,Tiparp,Atf3,Ier3 | | 8.745e-12 | -25.46 | GSE42021\_TREG\_PLN\_VS\_CD24HI\_TREG\_THYMUS\_UP | MSigDB lists | GSE42021\_TREG\_PLN\_VS\_CD24HI\_TREG\_THYMUS\_UP | 174 | 19 | 12187 | 179 | Nfkbiz,Socs3,Sgk1,Cdkn1a,Ier3,Il1a,Il1rn,Cd14,Ptgs2,Plaur,Errfi1,Icam1,Ccl2,Nfkbia,Birc3,Tnf,Gadd45b,Nfe2l2,Cxcl1 | | 8.745e-12 | -25.46 | GSE14769\_UNSTIM\_VS\_120MIN\_LPS\_BMDM\_DN | MSigDB lists | GSE14769\_UNSTIM\_VS\_120MIN\_LPS\_BMDM\_DN | 174 | 19 | 12187 | 179 | Vcam1,Il1rn,Tiparp,Atf3,Znfx1,Isg15,Tnip1,Zfp36,Sele,Plaur,Clic4,Ccl2,Sdc4,Tgm2,Parp14,AA467197,Cxcl1,Igsf6,Gbp3 | | 8.819e-12 | -25.45 | GSE18791\_UNSTIM\_VS\_NEWCATSLE\_VIRUS\_DC\_18H\_DN | MSigDB lists | GSE18791\_UNSTIM\_VS\_NEWCATSLE\_VIRUS\_DC\_18H\_DN | 114 | 16 | 12187 | 179 | Samd9l,Nfkbiz,Ifi44,Phf11b,Herc6,Ptgs2,Gadd45b,Oasl1,Parp14,Xaf1,Ifit2,Map3k8,Tnfsf10,Cmpk2,Irf7,Phf11d | | 8.942e-12 | -25.44 | GSE21360\_PRIMARY\_VS\_TERTIARY\_MEMORY\_CD8\_TCELL\_DN | MSigDB lists | GSE21360\_PRIMARY\_VS\_TERTIARY\_MEMORY\_CD8\_TCELL\_DN | 153 | 18 | 12187 | 179 | Gem,Irf7,Rrp8,Sbno2,Tnf,Sdc4,Casp4,Trib1,Il6,Gadd45b,Zfp36,Plek,Trim25,Icam1,Tap1,Cdkn1a,Socs3,Gadd45g | | 9.419e-12 | -25.39 | SMID\_BREAST\_CANCER\_LUMINAL\_B\_DN | MSigDB lists | SMID\_BREAST\_CANCER\_LUMINAL\_B\_DN | 352 | 26 | 12187 | 179 | Ifi204,Ifi207,Adamts1,Ifi209,Vcam1,Icam1,Sele,Clic4,Msn,Ptgs2,Birc3,Gem,S100a9,Lcn2,Sox11,Tap1,Ccl12,Fam107a,Tnfaip3,Il6,Ccl2,Tnfaip8,S100a8,Cxcl1,Ifi211,Angptl4 | | 1.048e-11 | -25.28 | regulation of metabolic process | biological process | GO:0019222 | 4733 | 122 | 13711 | 214 | Icam1,Nfkb2,Oasl1,Iigp1,Msr1,Gadd45g,Lgals9,Map3k8,Birc3,Nfkbie,Irgm1,Pik3r5,Znfx1,Tnfaip3,Depp1,Ccl2,C3ar1,Il1rn,Aff1,Zbtb16,Tnfaip8,Csf3,Lcn2,Ptpn1,Iqgap1,S100a9,Wfdc21,Il4ra,Nfkbia,Akap12,Parp14,Ccl7,Ccnd2,Hcar2,Timp1,Tiparp,Map3k6,Fam107a,Isg15,Ifi211,Cebpd,Rasip1,Runx1,Trim25,Resf1,Acod1,Pik3ap1,Atf3,Ncf1,Sele,Trim30a,C5ar1,Maff,Hspa5,Nfe2l2,Serpina3f,Mmp8,Rasd1,Angptl4,Msn,Noct,Mxd4,Sox11,Cybb,Errfi1,Thbs1,Rbm39,Banp,P2ry6,Gbp4,Nfkb1,Bach1,Plaur,Plek,Xdh,Ksr1,Tnfsf10,Arid5b,Irak3,Ifi204,S100a8,A2m,Ccl12,Irf2,Irf9,Bcl3,Samd9l,Irgm2,Oasl2,Casp4,Il1a,Il6,Cdkn1a,Dtx3l,Irf7,Socs3,Trib1,Ctla2a,Gadd45b,Pik3r1,Ifi209,Fzd4,Parp9,Sdc4,Sbno2,Spi1,Ccl4,Ptgs2,Ier3,Tnf,Zfp36,Ifi207,Hspb1,Ripk1,Cflar,Fstl1,Tnip1,Cxcl1,Dnajb1,Rrp8,Hp,Prg4 | | 1.095e-11 | -25.24 | GO\_CELL\_DEATH | MSigDB lists | GO\_CELL\_DEATH | 787 | 39 | 12187 | 179 | Thbs1,Sgk1,Ier3,Lcn2,Nfkb1,Gadd45g,Il1a,Cd14,Spi1,Map3k8,Ifit2,Pik3r1,Tnfaip3,Ripk1,Il6,Gadd45b,Hcar2,Ifi211,Rrp8,Bcl3,Tnfaip8,S100a8,Zbtb16,Cdkn1a,Ifi209,Ifi204,Ifi207,Plaur,Clic4,Xaf1,Nfkbia,Casp4,Tnf,Birc3,S100a9,Cflar,C5ar1,Xdh,Tnfsf10 | | 1.098e-11 | -25.24 | BOWIE\_RESPONSE\_TO\_TAMOXIFEN | MSigDB lists | BOWIE\_RESPONSE\_TO\_TAMOXIFEN | 22 | 9 | 12187 | 179 | Irf7,Ifi211,Isg15,Irf9,Ifit3b,Ifit2,Ifi209,Ifi207,Ifi204 | | 1.098e-11 | -25.24 | BENNETT\_SYSTEMIC\_LUPUS\_ERYTHEMATOSUS | MSigDB lists | BENNETT\_SYSTEMIC\_LUPUS\_ERYTHEMATOSUS | 22 | 9 | 12187 | 179 | Ifit3b,Xaf1,Oasl1,Isg15,Ifitm3,Irf7,S100a8,Tnfsf10,Tap1 | | 1.117e-11 | -25.22 | EGFR\_UP.V1\_UP | MSigDB lists | EGFR\_UP.V1\_UP | 155 | 18 | 12187 | 179 | Rgs16,Nfkb2,S100a8,Tnfaip8,S100a9,Bcl3,Arid5b,A2m,Angptl4,Ccl12,Il4ra,Tap1,P2ry6,Icam1,Cebpd,Atf3,Akap12,Ifit3b | | 1.120e-11 | -25.22 | GO\_POSITIVE\_REGULATION\_OF\_RESPONSE\_TO\_WOUNDING | MSigDB lists | GO\_POSITIVE\_REGULATION\_OF\_RESPONSE\_TO\_WOUNDING | 98 | 15 | 12187 | 179 | Thbs1,Ptgs2,Selp,Tnip1,Plek,Il17ra,Tnf,Ccl2,Ccl4,Nfe2l2,Il6,Ccl12,S100a9,S100a8,Osmr | | 1.216e-11 | -25.13 | BOYLAN\_MULTIPLE\_MYELOMA\_PCA1\_UP | MSigDB lists | BOYLAN\_MULTIPLE\_MYELOMA\_PCA1\_UP | 67 | 13 | 12187 | 179 | S100a9,Ifi211,Xdh,S100a8,Igsf6,Zbtb16,Ifi209,Lcn2,Cp,Ifi207,Hp,Ifi204,Ifitm3 | | 1.239e-11 | -25.11 | ZHOU\_INFLAMMATORY\_RESPONSE\_LIVE\_UP | MSigDB lists | ZHOU\_INFLAMMATORY\_RESPONSE\_LIVE\_UP | 328 | 25 | 12187 | 179 | Il6,Tnfaip3,Gpr84,Ccl4,Maff,Rgs16,Cxcl1,Csf3,Ier3,Il1a,Akap12,Mmp8,Nfkb1,Ptpn1,Rnf213,Nfkbia,Sdc4,Tnf,Nfkb2,Cflar,Col4a1,Plaur,Icam1,Tnip1,Ptgs2 | | 1.247e-11 | -25.11 | GSE5679\_PPARG\_LIGAND\_ROSIGLITAZONE\_VS\_RARA\_AGONIST\_AM580\_TREATED\_DC\_DN | MSigDB lists | GSE5679\_PPARG\_LIGAND\_ROSIGLITAZONE\_VS\_RARA\_AGONIST\_AM580\_TREATED\_DC\_DN | 156 | 18 | 12187 | 179 | Samd9l,Znfx1,Sgk1,Irak3,Irf9,Rnf213,Pik3r1,Il4ra,Xaf1,Usp18,Rtp4,Cmpk2,Tnfsf10,Irgm2,Gbp7,Pik3r5,Irf7,Bcl3 | | 1.390e-11 | -25.00 | GSE7509\_DC\_VS\_MONOCYTE\_WITH\_FCGRIIB\_STIM\_DN | MSigDB lists | GSE7509\_DC\_VS\_MONOCYTE\_WITH\_FCGRIIB\_STIM\_DN | 157 | 18 | 12187 | 179 | Oasl1,Il6,Ripk1,Ccl12,Ccl2,Xaf1,Ifit2,Sbno2,Irf7,Atf3,Ifit3b,Ifi44,Irf9,Zfp36,P2ry6,Trim25,Isg15,Ifitm3 | | 1.396e-11 | -24.99 | DIRMEIER\_LMP1\_RESPONSE\_EARLY | MSigDB lists | DIRMEIER\_LMP1\_RESPONSE\_EARLY | 54 | 12 | 12187 | 179 | Nfkbiz,Icam1,Zfp36,Tnfaip3,Gadd45b,Tnf,Birc3,Ccl4,Nfkbia,Nfkb2,Nfkbie,Irf7 | | 1.472e-11 | -24.94 | YANG\_BCL3\_TARGETS\_UP | MSigDB lists | YANG\_BCL3\_TARGETS\_UP | 303 | 24 | 12187 | 179 | Herc6,Sgk3,Tap1,Rbm47,Ifit3b,Timp1,Akap12,Hp,Cp,Angptl4,Irf7,A2m,Dtx3l,Gbp3,Rtp4,Cmpk2,Parp14,Usp18,Ccnd2,Tnf,Ifit2,Ccl12,Il6,Oasl1 | | 1.638e-11 | -24.83 | NF-kappa B signaling pathway | KEGG pathways | mmu04064 | 76 | 15 | 5248 | 107 | Cflar,Gadd45b,Nfkbia,Cd14,Ccl4,Tnfaip3,Vcam1,Ripk1,Icam1,Tnf,Birc3,Nfkb1,Trim25,Nfkb2,Ptgs2 | | 1.638e-11 | -24.83 | NF-kappa B signaling pathway | KEGG pathways | ko04064 | 76 | 15 | 5248 | 107 | Cflar,Nfkbia,Gadd45b,Ccl4,Cd14,Tnfaip3,Tnf,Icam1,Birc3,Ripk1,Vcam1,Trim25,Nfkb1,Nfkb2,Ptgs2 | | 1.679e-11 | -24.81 | cell communication | biological process | GO:0007154 | 3040 | 91 | 13711 | 214 | Akap12,Nfkbia,Il4ra,Hcar2,Ccl7,Isg15,Fam107a,Map3k6,Tiparp,Rasip1,Rhou,Atf3,Pik3ap1,Hspa5,Tgm2,C5ar1,Selp,Angptl4,Rasd1,Nfe2l2,Rassf4,Rgs16,Nfkb2,Fyb,Mt1,Lgals9,Iigp1,Rhoj,Map3k8,Pik3r5,Ccl2,Cxcl9,Lcn2,Il1rn,C3ar1,Tnip3,Iqgap1,Col4a1,Ptpn1,Ifitm2,Nfkbiz,S100a9,Vcam1,Cdkn1a,Il6,Gem,Il1a,Casp4,Pik3r1,Trib1,Socs3,Irf7,Dtx3l,Fzd4,Ier3,Sgk1,Spi1,Ccl4,Ifitm6,Stx11,Zfp36,Tnf,Rhoc,Tnip1,Gpr84,Cd14,Ripk1,Hspb1,Mt2,Hp,Cxcl1,Rrp8,Csf2rb,Errfi1,Cybb,P2ry6,Ifitm3,Nfkb1,Osmr,Plaur,Kcna5,Ksr1,Plek,Ifi204,Irak3,Arid5b,Ccl12,S100a8,Irgm2,Bcl3,Sgk3 | | 1.726e-11 | -24.78 | GSE2706\_R848\_VS\_R848\_AND\_LPS\_2H\_STIM\_DC\_DN | MSigDB lists | GSE2706\_R848\_VS\_R848\_AND\_LPS\_2H\_STIM\_DC\_DN | 119 | 16 | 12187 | 179 | Ifit3b,Socs3,Ifi44,Znfx1,Il1a,Tnip3,Isg15,Ptgs2,Herc6,Tap1,Pik3ap1,Ifit2,Tnf,Oasl1,Tnfaip3,Cxcl1 | | 1.746e-11 | -24.77 | signaling | biological process | GO:0023052 | 2939 | 89 | 13711 | 214 | Sgk3,Bcl3,Irgm2,S100a8,Ccl12,Arid5b,Irak3,Ifi204,Plek,Ksr1,Kcna5,Plaur,Osmr,Nfkb1,Ifitm3,P2ry6,Errfi1,Cybb,Csf2rb,Cxcl1,Rrp8,Mt2,Hp,Hspb1,Cd14,Gpr84,Ripk1,Tnip1,Rhoc,Tnf,Zfp36,Ifitm6,Stx11,Ccl4,Spi1,Sgk1,Ier3,Fzd4,Irf7,Socs3,Dtx3l,Trib1,Pik3r1,Casp4,Il1a,Gem,Il6,Cdkn1a,Vcam1,S100a9,Nfkbiz,Ifitm2,Ptpn1,Tnip3,Col4a1,Iqgap1,C3ar1,Il1rn,Lcn2,Cxcl9,Ccl2,Pik3r5,Map3k8,Rhoj,Iigp1,Lgals9,Mt1,Rgs16,Fyb,Nfkb2,Rassf4,Nfe2l2,Rasd1,Selp,Tgm2,C5ar1,Hspa5,Pik3ap1,Atf3,Rhou,Rasip1,Tiparp,Map3k6,Isg15,Ccl7,Hcar2,Il4ra,Akap12,Nfkbia | | 1.805e-11 | -24.74 | DER\_IFN\_GAMMA\_RESPONSE\_UP | MSigDB lists | DER\_IFN\_GAMMA\_RESPONSE\_UP | 69 | 13 | 12187 | 179 | Ifit3b,Ifi209,Cebpd,Ifi204,Ifi207,Isg15,Tap1,Irf9,Icam1,Ifit2,Il6,Rhoc,Ifi211 | | 1.805e-11 | -24.74 | DER\_IFN\_ALPHA\_RESPONSE\_UP | MSigDB lists | DER\_IFN\_ALPHA\_RESPONSE\_UP | 69 | 13 | 12187 | 179 | Ifi211,Isg15,Irf2,Irf9,Tap1,Ifit3b,Ifi209,Ifi44,Oasl1,Il6,Rhoc,Ifi204,Ifi207 | | 1.854e-11 | -24.71 | GSE7768\_OVA\_ALONE\_VS\_OVA\_WITH\_LPS\_IMMUNIZED\_MOUSE\_WHOLE\_SPLEEN\_6H\_DN | MSigDB lists | GSE7768\_OVA\_ALONE\_VS\_OVA\_WITH\_LPS\_IMMUNIZED\_MOUSE\_WHOLE\_SPLEEN\_6H\_DN | 139 | 17 | 12187 | 179 | Nfkbiz,Samd9l,Nfkb1,Ptgs2,Cd14,Plek,Icam1,Plaur,Rhou,Nfkbia,Casp4,Ccl4,Cybb,Nfe2l2,Nfkbie,Pik3r5,Igsf6 | | 1.890e-11 | -24.69 | regulation of innate immune response | biological process | GO:0045088 | 214 | 21 | 13711 | 214 | Parp9,Tap1,Tnip3,A2m,Ifi209,Irgm2,Irgm1,Ifi211,Nfkbia,Pik3ap1,Tnfaip3,Tnf,Acod1,Ifi204,Trim30a,Lgals9,Tnip1,Parp14,Cd14,Irak3,Irf7 | | 1.908e-11 | -24.68 | regulation of primary metabolic process | biological process | GO:0080090 | 4246 | 113 | 13711 | 214 | Plek,Tnfsf10,Ksr1,Xdh,Irak3,Arid5b,Ifi204,Irf2,Ccl12,A2m,S100a8,Irf9,Irgm2,Bcl3,Samd9l,Sox11,Mxd4,Errfi1,Cybb,Thbs1,P2ry6,Rbm39,Banp,Gbp4,Nfkb1,Bach1,Plaur,Tnf,Zfp36,Cflar,Ripk1,Hspb1,Ifi207,Tnip1,Fstl1,Dnajb1,Rrp8,Prg4,Il1a,Casp4,Oasl2,Cdkn1a,Il6,Trib1,Ctla2a,Irf7,Socs3,Dtx3l,Pik3r1,Gadd45b,Fzd4,Ifi209,Sbno2,Sdc4,Parp9,Ccl4,Spi1,Ier3,Ptgs2,Znfx1,Tnfaip3,Ccl2,Tnfaip8,Csf3,Zbtb16,Aff1,C3ar1,Il1rn,Ptpn1,Iqgap1,S100a9,Wfdc21,Nfkb2,Icam1,Oasl1,Lgals9,Gadd45g,Birc3,Map3k8,Nfkbie,Pik3r5,Pik3ap1,Acod1,Atf3,Ncf1,Trim30a,Hspa5,C5ar1,Maff,Mmp8,Rasd1,Serpina3f,Nfe2l2,Noct,Msn,Angptl4,Akap12,Nfkbia,Ccl7,Parp14,Hcar2,Ccnd2,Fam107a,Map3k6,Tiparp,Timp1,Isg15,Cebpd,Rasip1,Ifi211,Resf1,Trim25,Runx1 | | 2.000e-11 | -24.64 | regulation of tumor necrosis factor production | biological process | GO:0032680 | 131 | 17 | 13711 | 214 | Bcl3,Mmp8,Ccl4,Pik3r1,Lgals9,Trim30a,Hspb1,Irak3,Thbs1,Cd14,Ripk1,Zfp36,Errfi1,Cybb,Ccl2,Tnfaip3,Akap12 | | 2.063e-11 | -24.60 | positive regulation of protein metabolic process | biological process | GO:0051247 | 1402 | 56 | 13711 | 214 | Bcl3,Irgm2,S100a9,S100a8,Iqgap1,Ccl12,Ptpn1,Il1rn,Csf3,Tnfsf10,Ksr1,Xdh,Ccl2,Tnfaip3,Pik3r5,Plaur,Map3k8,Birc3,Gadd45g,Lgals9,P2ry6,Thbs1,Cybb,Icam1,Msn,Mmp8,C5ar1,Hspa5,Tnip1,Trim30a,Hspb1,Ncf1,Cflar,Ripk1,Tnf,Ptgs2,Ier3,Ccl4,Sdc4,Parp9,Tiparp,Fam107a,Map3k6,Fzd4,Ccnd2,Pik3r1,Gadd45b,Ccl7,Parp14,Trib1,Il6,Cdkn1a,Casp4,Akap12,Il1a,Nfkbia | | 2.083e-11 | -24.59 | GSE13484\_3H\_UNSTIM\_VS\_YF17D\_VACCINE\_STIM\_PBMC\_DN | MSigDB lists | GSE13484\_3H\_UNSTIM\_VS\_YF17D\_VACCINE\_STIM\_PBMC\_DN | 140 | 17 | 12187 | 179 | Oasl1,Bach1,Xaf1,Tor3a,Ccl4,Igsf6,Tnfaip8,Cflar,Gem,Il1a,Aff1,Plaur,Clic4,Irf9,Trim25,Icam1,Ptgs2 | | 2.134e-11 | -24.57 | GSE5679\_CTRL\_VS\_PPARG\_LIGAND\_ROSIGLITAZONE\_AND\_RARA\_AGONIST\_AM580\_TREATED\_DC\_UP | MSigDB lists | GSE5679\_CTRL\_VS\_PPARG\_LIGAND\_ROSIGLITAZONE\_AND\_RARA\_AGONIST\_AM580\_TREATED\_DC\_UP | 161 | 18 | 12187 | 179 | Clic4,Tap1,Icam1,Slfn3,Isg15,Znfx1,Ifit3b,Aff1,Gbp3,Slfn4,S100a8,Irf7,Irgm2,Cflar,Nfkbie,Gbp7,Cybb,Usp18 | | 2.186e-11 | -24.55 | positive regulation of biological process | biological process | GO:0048518 | 4720 | 121 | 13711 | 214 | Atf3,Acod1,Pik3ap1,Trim30a,Sele,Ncf1,Tgm2,C5ar1,Maff,Hspa5,Selp,Noct,Angptl4,Msn,Nfe2l2,Mmp8,Ip6k2,Il4ra,Akap12,Nfkbia,Hcar2,Ccnd2,Ccl7,Parp14,Isg15,Tiparp,Timp1,Fam107a,Map3k6,Runx1,Resf1,Trim25,Rasip1,Cebpd,Ifi211,Ccl2,Tnfaip3,Cxcl9,Lcn2,Aff1,Il1rn,C3ar1,Tnfaip8,Zbtb16,Csf3,Tnip3,Iqgap1,Cxcl16,Nfkbiz,Ptpn1,Vcam1,S100a9,Icam1,Fyb,Nfkb2,Gadd45g,Lgals9,Msr1,Rhoj,Map3k8,Birc3,Pik3r5,Irgm1,Zfp36,Tnf,Tnip1,Rhoc,Hspb1,Cflar,Cd14,Ripk1,Hp,Rrp8,Cxcl1,Ifit2,Il6,Cdkn1a,Casp4,Il1a,Pik3r1,Gadd45b,Irf7,Socs3,Dtx3l,Trib1,Sbno2,Sdc4,Parp9,Ifi209,Fzd4,Sgk1,Ptgs2,Ier3,Spi1,Ccl4,Ksr1,Tnfsf10,Xdh,Plek,Ifi204,Arid5b,Irak3,Adamts9,S100a8,Ccl12,Irf2,Bcl3,Irgm2,Cybb,Sox11,Banp,Rbm39,P2ry6,Thbs1,Nfkb1,Adamts1,Osmr,Plaur,Il17ra,Bach1,Kcna5 | | 2.189e-11 | -24.54 | HALLMARK\_IL6\_JAK\_STAT3\_SIGNALING | MSigDB lists | HALLMARK\_IL6\_JAK\_STAT3\_SIGNALING | 70 | 13 | 12187 | 179 | Il6,Il4ra,Tnf,Map3k8,Osmr,A2m,Pik3r5,Socs3,Il17ra,Irf9,Csf2rb,Ptpn1,Cd14 | | 2.228e-11 | -24.53 | MODULE\_1 | MSigDB lists | MODULE\_1 | 309 | 24 | 12187 | 179 | Ier3,Tiparp,Akap12,Gadd45g,Sgk1,Cd14,Tnfaip3,Ccl12,Nfe2l2,Il6,Tgm2,Ccnd2,Maff,Iqgap1,Ifi211,Atf3,Ifi204,Ifi207,Col4a1,Ifi209,Cdkn1a,Irf9,Sele,Gem | | 2.264e-11 | -24.51 | regulation of tumor necrosis factor superfamily cytokine production | biological process | GO:1903555 | 132 | 17 | 13711 | 214 | Cybb,Errfi1,Zfp36,Ccl2,Tnfaip3,Akap12,Pik3r1,Trim30a,Lgals9,Thbs1,Irak3,Hspb1,Ripk1,Cd14,Bcl3,Ccl4,Mmp8 | | 2.339e-11 | -24.48 | GSE40685\_TREG\_VS\_FOXP3\_KO\_TREG\_PRECURSOR\_UP | MSigDB lists | GSE40685\_TREG\_VS\_FOXP3\_KO\_TREG\_PRECURSOR\_UP | 141 | 17 | 12187 | 179 | Znfx1,Samd9l,Ifit3b,Irf9,Tap1,Parp9,Il17ra,Parp12,Oasl1,Casp4,Parp14,Xaf1,Ifit2,Tnfsf10,Gbp3,Rtp4,Bcl3 | | 2.369e-11 | -24.47 | response to tumor necrosis factor | biological process | GO:0034612 | 114 | 16 | 13711 | 214 | Birc3,Cxcl16,Ccl12,Nfkb1,Ccl4,Nfe2l2,Wfdc21,Nfkbia,Tnf,Acod1,Ccl2,Zfp36,Il6,Ripk1,Ccl7,Cd14 | | 2.918e-11 | -24.26 | GSE43863\_DAY6\_EFF\_VS\_DAY150\_MEM\_TH1\_CD4\_TCELL\_DN | MSigDB lists | GSE43863\_DAY6\_EFF\_VS\_DAY150\_MEM\_TH1\_CD4\_TCELL\_DN | 164 | 18 | 12187 | 179 | Socs3,Nfkbiz,Nfkb1,Ksr1,Ms4a6d,Tagln2,Ncf1,Irf9,Birc3,Gbp4,Tnfaip3,Gadd45b,Il6,Bcl3,Cflar,Irgm2,Gbp6,Gbp3 | | 2.935e-11 | -24.25 | cellular response to oxygen-containing compound | biological process | GO:1901701 | 686 | 37 | 13711 | 214 | Vcam1,Nfe2l2,Gbp6,Irgm2,Msn,Ptpn1,Cxcl1,Cxcl16,Ccl12,Col4a1,Tnip3,Ripk1,Cflar,Cd14,Il1rn,Ncf1,Lcn2,Cxcl9,Acod1,Tnfaip3,Tnf,Ccl2,Zfp36,Sgk1,Sbno2,Cmpk2,Nfkb1,Ccl7,Thbs1,P2ry6,Pik3r1,Nfkbia,Icam1,Mt1,Casp4,Ip6k2,Il6 | | 2.940e-11 | -24.25 | GSE43955\_TH0\_VS\_TGFB\_IL6\_TH17\_ACT\_CD4\_TCELL\_30H\_UP | MSigDB lists | GSE43955\_TH0\_VS\_TGFB\_IL6\_TH17\_ACT\_CD4\_TCELL\_30H\_UP | 143 | 17 | 12187 | 179 | Gbp3,Gbp7,Bach1,Gadd45b,Ccl12,Il4ra,Map3k8,Casp4,Ccl4,Il17ra,Errfi1,Ptpn1,Csf2rb,Ptgs2,Selp,Tiparp,Socs3 | | 3.015e-11 | -24.22 | positive regulation of metabolic process | biological process | GO:0009893 | 2865 | 87 | 13711 | 214 | Cybb,Sox11,P2ry6,Banp,Rbm39,Thbs1,Nfkb1,Plaur,Bach1,Xdh,Ksr1,Tnfsf10,Plek,Arid5b,Ccl12,Irf2,S100a8,Irgm2,Bcl3,Cdkn1a,Il6,Il1a,Casp4,Gadd45b,Pik3r1,Trib1,Dtx3l,Irf7,Parp9,Sbno2,Sdc4,Fzd4,Ier3,Ptgs2,Spi1,Ccl4,Zfp36,Tnf,Tnip1,Ripk1,Cflar,Hspb1,Cxcl1,Nfkb2,Icam1,Lgals9,Gadd45g,Birc3,Map3k8,Pik3r5,Irgm1,Ccl2,Tnfaip3,Lcn2,Csf3,Zbtb16,Il1rn,Aff1,C3ar1,Iqgap1,Ptpn1,S100a9,Nfkbia,Akap12,Il4ra,Ccnd2,Parp14,Ccl7,Map3k6,Fam107a,Tiparp,Resf1,Runx1,Cebpd,Atf3,Acod1,Trim30a,Ncf1,Sele,Hspa5,C5ar1,Maff,Msn,Angptl4,Noct,Mmp8,Nfe2l2 | | 3.121e-11 | -24.19 | GO\_NEGATIVE\_REGULATION\_OF\_MULTI\_ORGANISM\_PROCESS | MSigDB lists | GO\_NEGATIVE\_REGULATION\_OF\_MULTI\_ORGANISM\_PROCESS | 105 | 15 | 12187 | 179 | Tnfaip3,Oasl1,Tnf,Ccl4,Trib1,Ifi211,Timp1,Ifi204,Ifi207,Ifi209,Trim25,Ifitm3,Ifitm2,Isg15,Tnip1 | | 3.141e-11 | -24.18 | response to protozoan | biological process | GO:0001562 | 23 | 9 | 13711 | 214 | Iigp1,Gbp6,Irgm2,Ier3,Gbp7,Bcl3,Il4ra,Gbp3,Il6 | | 3.188e-11 | -24.17 | MODULE\_128 | MSigDB lists | MODULE\_128 | 72 | 13 | 12187 | 179 | C3ar1,A2m,Cybb,Ccl12,Ccnd2,Ifitm3,Ifitm2,Cd14,Timp1,Ier3,Cebpd,Thbs1,Vcam1 | | 3.511e-11 | -24.07 | UZONYI\_RESPONSE\_TO\_LEUKOTRIENE\_AND\_THROMBIN | MSigDB lists | UZONYI\_RESPONSE\_TO\_LEUKOTRIENE\_AND\_THROMBIN | 34 | 10 | 12187 | 179 | Ptgs2,Gem,Arid5b,Cxcl1,Sele,Zfp36,Apold1,Map3k8,Adamts1,Atf3 | | 3.829e-11 | -23.99 | MODULE\_79 | MSigDB lists | MODULE\_79 | 73 | 13 | 12187 | 179 | Timp1,Cebpd,Ier3,Thbs1,Vcam1,Ifitm3,Ifitm2,Cd14,Cybb,Ccl12,Ccnd2,C3ar1,A2m | | 3.963e-11 | -23.95 | GSE46606\_UNSTIM\_VS\_CD40L\_IL2\_IL5\_3DAY\_STIMULATED\_IRF4MID\_SORTED\_BCELL\_DN | MSigDB lists | GSE46606\_UNSTIM\_VS\_CD40L\_IL2\_IL5\_3DAY\_STIMULATED\_IRF4MID\_SORTED\_BCELL\_DN | 167 | 18 | 12187 | 179 | Gbp4,Hcar2,Casp4,Nfkbia,Nfkb2,Rtp4,Gbp6,Gem,Znfx1,Samd9l,Tiparp,Socs3,Cdkn1a,Tnip1,Rnf213,Ptgs2,Isg15,Ifitm3 | | 3.984e-11 | -23.95 | regulation of cellular process | biological process | GO:0050794 | 7419 | 162 | 13711 | 214 | Ptpn1,Ifitm2,Nfkbiz,Cxcl16,Tnip3,Col4a1,Iqgap1,S100a9,Vcam1,Wfdc21,Cxcl9,Tnfaip3,Znfx1,Ccl2,Depp1,Tnfaip8,Zbtb16,Csf3,C3ar1,Aff1,Il1rn,Lcn2,Birc3,Map3k8,Nfkbie,Apold1,Rhoj,Irgm1,Pik3r5,Rgs16,Nfkb2,Fyb,Mt1,Icam1,Rassf4,Msr1,Iigp1,Bbs12,Oasl1,Lgals9,Gadd45g,Hspa5,Clic4,Tgm2,C5ar1,Maff,Mmp8,Rasd1,Serpina3f,Nfe2l2,Noct,Selp,Angptl4,Msn,Pik3ap1,Acod1,Rhou,Atf3,Sele,Ncf1,Trim30a,Rnf213,Fam107a,Map3k6,Tiparp,Timp1,Isg15,Slfn2,Cebpd,Rasip1,Xaf1,Ifi211,Resf1,Trim25,Runx1,Akap12,Nfkbia,Il4ra,Slfn3,Ip6k2,Ccl7,Parp14,Hcar2,Ccnd2,Ccl12,Irf2,A2m,S100a8,Sgk3,Irf9,Irgm2,Bcl3,Plek,Ksr1,Tnfsf10,Xdh,Adamts9,Arid5b,Irak3,Ifi204,Osmr,Gbp4,Adamts1,Nfkb1,Kcna5,Bach1,Plaur,Il17ra,Sox11,Mxd4,Errfi1,Cybb,Ifitm3,Thbs1,P2ry6,Banp,Rbm39,Dnajb1,Rrp8,Cxcl1,Prg4,Mt2,Hp,Ifit2,Csf2rb,Robo4,Tnf,Zfp36,Gpr84,Cd14,Cflar,Ripk1,Hspb1,Ifi207,Rhoc,Tnip1,Fstl1,Fzd4,Ifi209,Sdc4,Ptges,Sbno2,Parp9,Spi1,Ccl4,Ifitm6,Ier3,Sgk1,Ptgs2,Il1a,Gem,Casp4,Oasl2,Cdkn1a,Il6,Ctla2a,Trib1,Irf7,Socs3,Dtx3l,Pik3r1,Gadd45b | | 4.022e-11 | -23.94 | RASHI\_NFKB1\_TARGETS | MSigDB lists | RASHI\_NFKB1\_TARGETS | 17 | 8 | 12187 | 179 | Tnip1,Cxcl1,Nfkb2,Birc3,Map3k8,Nfkbia,Lcn2,Tnfaip3 | | 4.277e-11 | -23.88 | MODULE\_76 | MSigDB lists | MODULE\_76 | 59 | 12 | 12187 | 179 | Cxcl1,S100a9,C3ar1,Ccl12,Cybb,Ccl4,Ccl2,Cd14,Ptgs2,Saa1,Nfkb1,Il1a | | 4.395e-11 | -23.85 | regulation of nitrogen compound metabolic process | biological process | GO:0051171 | 4126 | 110 | 13711 | 214 | Il1rn,Aff1,C3ar1,Csf3,Tnfaip8,Zbtb16,Ccl2,Znfx1,Tnfaip3,Wfdc21,S100a9,Iqgap1,Ptpn1,Gadd45g,Lgals9,Oasl1,Icam1,Nfkb2,Pik3r5,Nfkbie,Map3k8,Birc3,Trim30a,Ncf1,Atf3,Acod1,Pik3ap1,Msn,Noct,Nfe2l2,Serpina3f,Rasd1,Mmp8,C5ar1,Maff,Hspa5,Ccnd2,Parp14,Ccl7,Nfkbia,Akap12,Runx1,Resf1,Trim25,Ifi211,Cebpd,Rasip1,Isg15,Timp1,Tiparp,Map3k6,Fam107a,Ifi204,Arid5b,Irak3,Xdh,Tnfsf10,Ksr1,Bcl3,Samd9l,Irgm2,Irf9,S100a8,A2m,Irf2,Ccl12,Banp,Rbm39,P2ry6,Thbs1,Cybb,Errfi1,Mxd4,Sox11,Plaur,Bach1,Nfkb1,Gbp4,Fstl1,Tnip1,Ifi207,Hspb1,Ripk1,Cflar,Zfp36,Tnf,Prg4,Rrp8,Dnajb1,Gadd45b,Pik3r1,Dtx3l,Socs3,Irf7,Trib1,Ctla2a,Il6,Cdkn1a,Oasl2,Casp4,Il1a,Ptgs2,Ier3,Ccl4,Spi1,Parp9,Sbno2,Sdc4,Ifi209,Fzd4 | | 4.593e-11 | -23.80 | HALLMARK\_APOPTOSIS | MSigDB lists | HALLMARK\_APOPTOSIS | 147 | 17 | 12187 | 179 | Gadd45b,Il6,Tnf,Ccnd2,Birc3,Casp4,Tnfsf10,Cflar,Timp1,Il1a,Atf3,Ier3,Cdkn1a,Hspb1,Tap1,Ifitm3,Cd14 | | 4.782e-11 | -23.76 | regulation of cellular metabolic process | biological process | GO:0031323 | 4418 | 115 | 13711 | 214 | Ifi209,Fzd4,Sdc4,Sbno2,Parp9,Ccl4,Spi1,Ptgs2,Ier3,Casp4,Oasl2,Il1a,Il6,Cdkn1a,Irf7,Socs3,Dtx3l,Trib1,Ctla2a,Pik3r1,Gadd45b,Rrp8,Dnajb1,Cxcl1,Prg4,Hp,Tnf,Zfp36,Hspb1,Ifi207,Cflar,Ripk1,Tnip1,Fstl1,Gbp4,Nfkb1,Bach1,Plaur,Mxd4,Sox11,Errfi1,Cybb,Thbs1,Banp,Rbm39,P2ry6,A2m,S100a8,Ccl12,Irf2,Irf9,Bcl3,Irgm2,Plek,Tnfsf10,Ksr1,Xdh,Irak3,Arid5b,Ifi204,Timp1,Fam107a,Map3k6,Isg15,Rasip1,Cebpd,Ifi211,Runx1,Resf1,Trim25,Akap12,Nfkbia,Ccl7,Parp14,Ccnd2,C5ar1,Maff,Hspa5,Serpina3f,Nfe2l2,Rasd1,Mmp8,Noct,Msn,Acod1,Pik3ap1,Atf3,Sele,Ncf1,Trim30a,Map3k8,Birc3,Nfkbie,Irgm1,Pik3r5,Icam1,Nfkb2,Oasl1,Iigp1,Gadd45g,Lgals9,Ptpn1,Iqgap1,S100a9,Wfdc21,Tnfaip3,Znfx1,Ccl2,Depp1,Aff1,C3ar1,Il1rn,Csf3,Tnfaip8,Zbtb16 | | 4.844e-11 | -23.75 | GSE20715\_0H\_VS\_24H\_OZONE\_TLR4\_KO\_LUNG\_DN | MSigDB lists | GSE20715\_0H\_VS\_24H\_OZONE\_TLR4\_KO\_LUNG\_DN | 169 | 18 | 12187 | 179 | Saa1,Thbs1,Hspa5,Cdkn1a,Socs3,Zbtb16,Col4a1,Hp,Gadd45g,Timp1,Lcn2,Bcl3,Cxcl1,Angptl4,Osmr,Maff,Rhou,Tubb6 | | 5.411e-11 | -23.64 | GO\_DEFENSE\_RESPONSE\_TO\_VIRUS | MSigDB lists | GO\_DEFENSE\_RESPONSE\_TO\_VIRUS | 109 | 15 | 12187 | 179 | Ifi211,Trim56,Irf7,Oasl1,Il6,Ifit2,Irf9,Trim25,Ifitm3,Ifitm2,Isg15,Ifi204,Ifi207,Ifit3b,Ifi209 | | 5.510e-11 | -23.62 | positive regulation of cellular process | biological process | GO:0048522 | 4254 | 112 | 13711 | 214 | Sele,Ncf1,Acod1,Pik3ap1,Atf3,Nfe2l2,Mmp8,Selp,Noct,Msn,Tgm2,C5ar1,Maff,Hspa5,Ccl7,Parp14,Ccnd2,Hcar2,Il4ra,Akap12,Nfkbia,Ip6k2,Rasip1,Cebpd,Runx1,Trim25,Resf1,Timp1,Fam107a,Map3k6,Isg15,Il1rn,C3ar1,Aff1,Csf3,Tnfaip8,Zbtb16,Lcn2,Tnfaip3,Cxcl9,Ccl2,Vcam1,S100a9,Nfkbiz,Ptpn1,Iqgap1,Cxcl16,Msr1,Gadd45g,Lgals9,Icam1,Nfkb2,Irgm1,Pik3r5,Map3k8,Birc3,Rhoj,Hspb1,Cd14,Cflar,Ripk1,Tnip1,Rhoc,Tnf,Zfp36,Ifit2,Rrp8,Cxcl1,Hp,Socs3,Irf7,Dtx3l,Trib1,Pik3r1,Gadd45b,Casp4,Il1a,Il6,Cdkn1a,Spi1,Ccl4,Sgk1,Ptgs2,Fzd4,Sbno2,Sdc4,Parp9,Arid5b,Adamts9,Ifi204,Plek,Ksr1,Tnfsf10,Xdh,Bcl3,Irgm2,S100a8,Ccl12,Irf2,Thbs1,Rbm39,Banp,P2ry6,Sox11,Cybb,Bach1,Kcna5,Plaur,Il17ra,Osmr,Nfkb1,Adamts1 | | 5.643e-11 | -23.60 | GO\_POSITIVE\_REGULATION\_OF\_INTRACELLULAR\_SIGNAL\_TRANSDUCTION | MSigDB lists | GO\_POSITIVE\_REGULATION\_OF\_INTRACELLULAR\_SIGNAL\_TRANSDUCTION | 645 | 34 | 12187 | 179 | Birc3,Tnf,Fzd4,S100a9,Cflar,C5ar1,Tnfsf10,Xdh,Socs3,Ksr1,Saa1,Ptgs2,Icam1,Trim25,Ccl2,Ccl4,Map3k8,Il6,Gadd45b,Ripk1,Ccl12,Rhoc,Pik3r5,Csf3,S100a8,Thbs1,Sox11,Gadd45g,Il1a,Akap12,Map3k6,Selp,Ptpn1,Pik3ap1 | | 6.360e-11 | -23.48 | GSE14000\_UNSTIM\_VS\_16H\_LPS\_DC\_TRANSLATED\_RNA\_DN | MSigDB lists | GSE14000\_UNSTIM\_VS\_16H\_LPS\_DC\_TRANSLATED\_RNA\_DN | 150 | 17 | 12187 | 179 | Parp12,Birc3,Ifit2,Usp18,Ccl4,Cmpk2,Tnfsf10,Cflar,Trim56,Irf7,Phf11d,Samd9l,Atf3,Phf11b,Ifit3b,Nfkbiz,Herc6 | | 6.360e-11 | -23.48 | GSE22886\_NAIVE\_BCELL\_VS\_NEUTROPHIL\_DN | MSigDB lists | GSE22886\_NAIVE\_BCELL\_VS\_NEUTROPHIL\_DN | 150 | 17 | 12187 | 179 | C5ar1,Cflar,Bcl3,S100a9,Igsf6,S100a8,Tnfsf10,Trib1,Casp4,Ifitm3,Ptgs2,Ifitm2,Plek,Csf2rb,Ifit3b,Ier3,Cebpd | | 6.360e-11 | -23.48 | GSE8515\_CTRL\_VS\_IL1\_4H\_STIM\_MAC\_DN | MSigDB lists | GSE8515\_CTRL\_VS\_IL1\_4H\_STIM\_MAC\_DN | 150 | 17 | 12187 | 179 | Tnip1,Icam1,Plek,Nfkb1,Tnip3,Ier3,Nfkbie,Cxcl1,Bcl3,Nfkb2,Tnf,Ccl4,Nfkbia,Sdc4,Tnfaip3,Gadd45b,Il6 | | 6.901e-11 | -23.40 | DEBIASI\_APOPTOSIS\_BY\_REOVIRUS\_INFECTION\_UP | MSigDB lists | DEBIASI\_APOPTOSIS\_BY\_REOVIRUS\_INFECTION\_UP | 271 | 22 | 12187 | 179 | Oasl1,Tnfaip3,Bach1,Nfkbia,Ifit2,Zfp189,Irf7,Cxcl1,Ifi211,Gem,Atf3,Ifi207,Ifi204,Tiparp,Ifit3b,Ifi44,Ifi209,Tap1,Irf9,Zfp36,Isg15,Ifitm3 | | 7.076e-11 | -23.37 | GSE22589\_HEALTHY\_VS\_HIV\_INFECTED\_DC\_DN | MSigDB lists | GSE22589\_HEALTHY\_VS\_HIV\_INFECTED\_DC\_DN | 151 | 17 | 12187 | 179 | Irf9,Tap1,Herc6,Ifitm3,Slfn5,Cybb,Gbp4,Tnf,Tor3a,Parp14,Rtp4,Gbp3,Gbp7,Gbp6,Gem,C5ar1,Irf7 | | 7.076e-11 | -23.37 | GSE360\_L\_DONOVANI\_VS\_M\_TUBERCULOSIS\_DC\_DN | MSigDB lists | GSE360\_L\_DONOVANI\_VS\_M\_TUBERCULOSIS\_DC\_DN | 151 | 17 | 12187 | 179 | Plek,Sele,Isg15,Cd14,Mmp8,Ifi44,Olfml2b,S100a8,Tnfsf10,Pygm,C3ar1,S100a9,Irf7,Ccl12,Oasl1,Ifit2,Ccl4 | | 7.080e-11 | -23.37 | NUYTTEN\_NIPP1\_TARGETS\_UP | MSigDB lists | NUYTTEN\_NIPP1\_TARGETS\_UP | 580 | 32 | 12187 | 179 | Sgk1,Slfn5,Rnf213,Isg15,Tap1,Parp9,Ccdc58,Ptpn1,Tgm2,Tnfaip3,Cxcl1,Ifi211,Csf3,Rtp4,Ifi44,Ifi209,Atf3,Luc7l3,Znfx1,Ifi207,Samd9l,Ifi204,Ptgs2,Casp4,Parp14,Xaf1,Birc3,Parp12,Oasl1,Gem,Robo4,Tnfsf10 | | 7.327e-11 | -23.34 | COLINA\_TARGETS\_OF\_4EBP1\_AND\_4EBP2 | MSigDB lists | COLINA\_TARGETS\_OF\_4EBP1\_AND\_4EBP2 | 299 | 23 | 12187 | 179 | Irgm2,Sdc4,Tor3a,Parp12,Trim25,Irf9,Ifi44,Adamts1,Vcam1,Cp,Il1rn,Rtp4,Gbp3,Ifit2,Usp18,Tgm2,Isg15,Ifitm3,Cd14,Thbs1,Akap12,Nfkb1,Lcn2 | | 8.738e-11 | -23.16 | GSE22886\_DAY1\_VS\_DAY7\_MONOCYTE\_IN\_CULTURE\_UP | MSigDB lists | GSE22886\_DAY1\_VS\_DAY7\_MONOCYTE\_IN\_CULTURE\_UP | 153 | 17 | 12187 | 179 | Ptgs2,Tnip1,Icam1,Irak3,Il1a,Tnip3,Ier3,Cflar,Irf7,Banp,Tnfaip8,Runx1,Nfkbia,Oasl1,Il6,Gadd45b,Tnfaip3 | | 9.698e-11 | -23.06 | GSE37301\_MULTIPOTENT\_PROGENITOR\_VS\_GRAN\_MONO\_PROGENITOR\_UP | MSigDB lists | GSE37301\_MULTIPOTENT\_PROGENITOR\_VS\_GRAN\_MONO\_PROGENITOR\_UP | 154 | 17 | 12187 | 179 | Il17ra,Ptpn1,Isg15,Cd14,Col4a1,Cebpd,Znfx1,Cmpk2,Xdh,Gbp7,Irgm2,C5ar1,Bcl3,Cxcl1,Ifit2,Usp18,Tor3a | | 9.698e-11 | -23.06 | GSE37533\_PPARG2\_FOXP3\_VS\_FOXP3\_TRANSDUCED\_CD4\_TCELL\_DN | MSigDB lists | GSE37533\_PPARG2\_FOXP3\_VS\_FOXP3\_TRANSDUCED\_CD4\_TCELL\_DN | 154 | 17 | 12187 | 179 | Rtp4,Irf7,Phf11d,Parp12,Oasl1,Ripk1,Usp18,Xaf1,Ifit2,Irf9,Tap1,Irf2,Herc6,Isg15,Ifit3b,Ifi44,Phf11b | | 9.698e-11 | -23.06 | GSE360\_LOW\_DOSE\_B\_MALAYI\_VS\_M\_TUBERCULOSIS\_DC\_DN | MSigDB lists | GSE360\_LOW\_DOSE\_B\_MALAYI\_VS\_M\_TUBERCULOSIS\_DC\_DN | 154 | 17 | 12187 | 179 | Icam1,Plaur,Tnip1,Ier3,Atf3,Ifi44,Hspa5,S100a8,Cflar,C3ar1,Gem,S100a9,Cybb,Ccl12,Oasl1,Ifit2,Birc3 | | 1.076e-10 | -22.95 | GSE8515\_IL1\_VS\_IL6\_4H\_STIM\_MAC\_UP | MSigDB lists | GSE8515\_IL1\_VS\_IL6\_4H\_STIM\_MAC\_UP | 155 | 17 | 12187 | 179 | Cxcl1,Nfkb2,Ccl4,Tor3a,Nfkbia,Sdc4,Tnf,Maff,Tnfaip3,Il6,Tnip1,Ptgs2,Icam1,Plek,Ier3,Nfkb1,Stx11 | | 1.078e-10 | -22.95 | GSE41176\_UNSTIM\_VS\_ANTI\_IGM\_STIM\_BCELL\_3H\_UP | MSigDB lists | GSE41176\_UNSTIM\_VS\_ANTI\_IGM\_STIM\_BCELL\_3H\_UP | 134 | 16 | 12187 | 179 | Irak3,Plaur,Rgs16,Cflar,Ptgs2,Tnip1,Cxcl1,Tnip3,Il1a,Tnfaip3,Ier3,Il6,Tnf,Maff,Socs3,Ccl4 | | 1.095e-10 | -22.94 | regulation of protein phosphorylation | biological process | GO:0001932 | 1172 | 49 | 13711 | 214 | Iqgap1,Ccl12,Ptpn1,Irgm2,Xdh,Ksr1,Ccl2,Tnfaip3,Il1rn,Irak3,Csf3,Map3k8,Gbp4,Pik3r5,Plaur,Errfi1,Icam1,Gadd45g,P2ry6,Lgals9,Thbs1,C5ar1,Mmp8,Atf3,Tnf,Pik3ap1,Tnip1,Ncf1,Hspb1,Ripk1,Cflar,Parp9,Sdc4,Map3k6,Fzd4,Ptgs2,Rasip1,Ccl4,Il6,Cdkn1a,Akap12,Il1a,Gadd45b,Ccnd2,Pik3r1,Socs3,Trib1,Parp14,Ccl7 | | 1.203e-10 | -22.84 | MCDOWELL\_ACUTE\_LUNG\_INJURY\_UP | MSigDB lists | MCDOWELL\_ACUTE\_LUNG\_INJURY\_UP | 38 | 10 | 12187 | 179 | Akap12,Lcn2,Il4ra,Cdkn1a,Nfkbia,Zfp36,Osmr,S100a8,Ptges,Angptl4 | | 1.203e-10 | -22.84 | RADAEVA\_RESPONSE\_TO\_IFNA1\_UP | MSigDB lists | RADAEVA\_RESPONSE\_TO\_IFNA1\_UP | 38 | 10 | 12187 | 179 | Tnfsf10,Isg15,Ifitm2,Ifitm3,Irf7,Rrp8,Mxd4,Ifi44,Ifit2,Vcam1 | | 1.225e-10 | -22.82 | regulation of macromolecule metabolic process | biological process | GO:0060255 | 4362 | 113 | 13711 | 214 | Zfp36,Tnf,Fstl1,Tnip1,Ifi207,Hspb1,Ripk1,Cflar,Prg4,Dnajb1,Rrp8,Il6,Cdkn1a,Oasl2,Casp4,Il1a,Gadd45b,Pik3r1,Dtx3l,Irf7,Socs3,Ctla2a,Trib1,Parp9,Sdc4,Sbno2,Ifi209,Fzd4,Ptgs2,Ier3,Ccl4,Spi1,Xdh,Ksr1,Tnfsf10,Ifi204,Arid5b,Irak3,S100a8,A2m,Ccl12,Irf2,Samd9l,Bcl3,Irgm2,Irf9,Cybb,Errfi1,Mxd4,Sox11,Banp,Rbm39,P2ry6,Thbs1,Nfkb1,Gbp4,Plaur,Bach1,Atf3,Acod1,Pik3ap1,Trim30a,Ncf1,Sele,C5ar1,Maff,Hspa5,Msn,Noct,Nfe2l2,Serpina3f,Rasd1,Mmp8,Nfkbia,Akap12,Ccnd2,Parp14,Ccl7,Isg15,Tiparp,Timp1,Map3k6,Fam107a,Runx1,Resf1,Trim25,Ifi211,Rasip1,Cebpd,Ccl2,Tnfaip3,Znfx1,Lcn2,C3ar1,Il1rn,Aff1,Tnfaip8,Csf3,Zbtb16,Iqgap1,Ptpn1,Wfdc21,S100a9,Icam1,Nfkb2,Gadd45g,Lgals9,Oasl1,Msr1,Nfkbie,Map3k8,Birc3,Pik3r5 | | 1.320e-10 | -22.75 | GSE19888\_ADENOSINE\_A3R\_INH\_VS\_INH\_PRETREAT\_AND\_ACT\_WITH\_TCELL\_MEMBRANES\_MAST\_CELL\_UP | MSigDB lists | GSE19888\_ADENOSINE\_A3R\_INH\_VS\_INH\_PRETREAT\_AND\_ACT\_WITH\_TCELL\_MEMBRANES\_MAST\_CELL\_UP | 157 | 17 | 12187 | 179 | Isg15,Rnf213,Samd9l,Ms4a6d,Akap12,Gbp3,Rtp4,Gbp6,Gbp7,Dtx3l,Irgm2,Parp12,Gbp4,Usp18,Casp4,Tor3a,Parp14 | | 1.413e-10 | -22.68 | ST\_TUMOR\_NECROSIS\_FACTOR\_PATHWAY | MSigDB lists | ST\_TUMOR\_NECROSIS\_FACTOR\_PATHWAY | 28 | 9 | 12187 | 179 | Nfkb2,Nfkbie,Cflar,Ripk1,Tnfaip3,Nfkb1,Nfkbia,Birc3,Tnf | | 1.460e-10 | -22.65 | GSE39864\_WT\_VS\_GATA3\_KO\_TREG\_UP | MSigDB lists | GSE39864\_WT\_VS\_GATA3\_KO\_TREG\_UP | 158 | 17 | 12187 | 179 | Timp1,Ptgs2,Selp,Ifitm3,Slfn3,Tap1,Il4ra,Ifit2,Map3k8,Ccl2,Casp4,Usp18,Ccl12,Gbp7,Irgm2,Slfn4,Gbp3 | | 1.598e-10 | -22.56 | GO\_NEGATIVE\_REGULATION\_OF\_VIRAL\_GENOME\_REPLICATION | MSigDB lists | GO\_NEGATIVE\_REGULATION\_OF\_VIRAL\_GENOME\_REPLICATION | 39 | 10 | 12187 | 179 | Tnip1,Ifi211,Isg15,Ifitm2,Ifitm3,Tnf,Ifi209,Oasl1,Ifi207,Ifi204 | | 1.614e-10 | -22.55 | GSE45365\_WT\_VS\_IFNAR\_KO\_CD8A\_DC\_DN | MSigDB lists | GSE45365\_WT\_VS\_IFNAR\_KO\_CD8A\_DC\_DN | 159 | 17 | 12187 | 179 | Tap1,Ptpn1,Tnip1,Herc6,Ifi44,Phf11b,Irf7,Cxcl1,Phf11d,Robo4,Csf3,Parp12,Tnfaip3,Rassf4,Nfkbia,Sdc4,Il4ra | | 1.719e-10 | -22.48 | GSE17974\_IL4\_AND\_ANTI\_IL12\_VS\_UNTREATED\_48H\_ACT\_CD4\_TCELL\_DN | MSigDB lists | GSE17974\_IL4\_AND\_ANTI\_IL12\_VS\_UNTREATED\_48H\_ACT\_CD4\_TCELL\_DN | 118 | 15 | 12187 | 179 | Atf3,Ksr1,Ifi204,Ifi207,Cdkn1a,Ifi209,Phf11b,Ifi44,Parp9,Parp14,Map3k8,Pik3r5,Phf11d,Ifi211,Irf7 | | 1.720e-10 | -22.48 | GO\_NEGATIVE\_REGULATION\_OF\_VIRAL\_PROCESS | MSigDB lists | GO\_NEGATIVE\_REGULATION\_OF\_VIRAL\_PROCESS | 66 | 12 | 12187 | 179 | Ifi207,Ifi204,Ifi209,Trim25,Tnip1,Ifitm2,Isg15,Ifitm3,Oasl1,Ccl4,Tnf,Ifi211 | | 1.727e-10 | -22.48 | regulation of signal transduction | biological process | GO:0009966 | 2258 | 73 | 13711 | 214 | Parp9,Timp1,Map3k6,Fzd4,Ptgs2,Runx1,Ier3,Trim25,Rasip1,Ccl4,Il6,Casp4,Il1a,Akap12,Nfkbia,Pik3r1,Gadd45b,Irf7,Socs3,Ccl7,Parp14,Trib1,Tgm2,C5ar1,Hspa5,Selp,Nfe2l2,Mmp8,Atf3,Tnf,Acod1,Pik3ap1,Tnip1,Fstl1,Trim30a,Rnf213,Hspb1,Ncf1,Cd14,Cflar,Ripk1,Nfkb1,Map3k8,Birc3,Pik3r5,Plaur,Irgm1,Errfi1,Icam1,Rgs16,Sox11,Gadd45g,Lgals9,P2ry6,Thbs1,Tnip3,S100a8,Iqgap1,Ccl12,Ptpn1,Bcl3,Irgm2,Sgk3,S100a9,Ksr1,Tnfsf10,Xdh,Ccl2,Tnfaip3,Plek,Il1rn,Irak3,Csf3 | | 1.735e-10 | -22.48 | BOQUEST\_STEM\_CELL\_CULTURED\_VS\_FRESH\_UP | MSigDB lists | BOQUEST\_STEM\_CELL\_CULTURED\_VS\_FRESH\_UP | 341 | 24 | 12187 | 179 | Nfkbia,Trib1,Oasl1,Gem,A2m,Tnfsf10,Ifi44,Vcam1,Zbtb16,Atf3,Apold1,Zfp36,Map3k8,Pik3r1,Tgm2,Tnfaip3,Ccl12,Gadd45b,Angptl4,Cxcl1,Rgs16,Thbs1,Dnajb1,Gadd45g | | 1.765e-10 | -22.46 | positive regulation of macromolecule metabolic process | biological process | GO:0010604 | 2653 | 81 | 13711 | 214 | Nfe2l2,Mmp8,Msn,Noct,C5ar1,Maff,Hspa5,Ncf1,Hspb1,Sele,Ripk1,Cflar,Tnip1,Trim30a,Tnf,Atf3,Zfp36,Cebpd,Ccl4,Spi1,Runx1,Ptgs2,Resf1,Ier3,Tiparp,Fzd4,Map3k6,Fam107a,Parp9,Sdc4,Sbno2,Dtx3l,Irf7,Parp14,Trib1,Ccl7,Gadd45b,Pik3r1,Ccnd2,Casp4,Nfkbia,Il1a,Akap12,Il6,Cdkn1a,S100a9,Bcl3,Irgm2,Ptpn1,S100a8,Iqgap1,Irf2,Ccl12,Arid5b,C3ar1,Il1rn,Aff1,Zbtb16,Csf3,Lcn2,Tnfaip3,Xdh,Tnfsf10,Ksr1,Ccl2,Bach1,Pik3r5,Plaur,Map3k8,Birc3,Nfkb1,Thbs1,Banp,Gadd45g,Rbm39,P2ry6,Lgals9,Icam1,Nfkb2,Sox11,Cybb | | 1.766e-10 | -22.46 | Type II interferon signaling (IFNG) | WikiPathways | WP1253 | 25 | 10 | 3756 | 96 | Socs3,Irf2,Ifit2,Tap1,Icam1,Spi1,Isg15,Cxcl9,Cybb,Irf9 | | 2.069e-10 | -22.30 | JACKSON\_DNMT1\_TARGETS\_UP | MSigDB lists | JACKSON\_DNMT1\_TARGETS\_UP | 67 | 12 | 12187 | 179 | Isg15,Ptgs2,Trim25,Hspb1,Zfp36,Cdkn1a,Socs3,Ifit3b,Irf7,Rgs16,Ccl12,Nfe2l2 | | 2.078e-10 | -22.29 | regulation of phosphorylation | biological process | GO:0042325 | 1276 | 51 | 13711 | 214 | Tnip1,Hspb1,Ncf1,Cflar,Ripk1,Atf3,Tnf,Pik3ap1,Mmp8,C5ar1,Pik3r1,Ccnd2,Gadd45b,Socs3,Ccl7,Parp14,Trib1,Il6,Cdkn1a,Akap12,Il1a,Ptgs2,Ier3,Rasip1,Ccl4,Sdc4,Parp9,Fzd4,Map3k6,Il1rn,Irak3,Csf3,Ksr1,Xdh,Ccl2,Tnfaip3,Irgm2,Iqgap1,Ccl12,Ptpn1,Gadd45g,Lgals9,P2ry6,Thbs1,Errfi1,Icam1,Pik3r5,Plaur,Gbp4,Map3k8,Birc3 | | 2.098e-10 | -22.28 | GSE30083\_SP1\_VS\_SP2\_THYMOCYTE\_DN | MSigDB lists | GSE30083\_SP1\_VS\_SP2\_THYMOCYTE\_DN | 140 | 16 | 12187 | 179 | Irf9,Tap1,Il17ra,Slfn5,Herc6,Samd9l,Ifi44,Sox11,Rgs16,Cmpk2,Gbp6,Dtx3l,Irgm2,Gbp4,Parp14,Ccnd2 | | 2.191e-10 | -22.24 | GSE21063\_CTRL\_VS\_ANTI\_IGM\_STIM\_BCELL\_8H\_UP | MSigDB lists | GSE21063\_CTRL\_VS\_ANTI\_IGM\_STIM\_BCELL\_8H\_UP | 120 | 15 | 12187 | 179 | Nfkb2,Tnfsf10,Parp14,Trib1,Nfkbia,Birc3,Tnfaip3,Gadd45b,Isg15,Clic4,Plaur,Icam1,Zfp36,Nfkbiz,Nfkb1 | | 2.394e-10 | -22.15 | GSE2128\_CTRL\_VS\_MIMETOPE\_NEGATIVE\_SELECTION\_DP\_THYMOCYTE\_NOD\_UP | MSigDB lists | GSE2128\_CTRL\_VS\_MIMETOPE\_NEGATIVE\_SELECTION\_DP\_THYMOCYTE\_NOD\_UP | 163 | 17 | 12187 | 179 | Ccl4,Gpr84,Sdc4,Casp4,Lgals9,Gbp3,Rtp4,Bcl3,C3ar1,Gbp7,Atf3,Ms4a6d,Irak3,Cd14,Ifitm3,Ifitm2,Ptgs2 | | 2.394e-10 | -22.15 | GSE9960\_HEALTHY\_VS\_GRAM\_NEG\_SEPSIS\_PBMC\_DN | MSigDB lists | GSE9960\_HEALTHY\_VS\_GRAM\_NEG\_SEPSIS\_PBMC\_DN | 163 | 17 | 12187 | 179 | Phf11b,Nfkb1,Tnip3,Tagln2,Ifitm3,Csf2rb,Plaur,Clic4,Irf2,Pik3ap1,Gpr84,Nfkbia,Phf11d,Bcl3,Irf7,Cflar,Tnfaip8 | | 2.395e-10 | -22.15 | CHEN\_HOXA5\_TARGETS\_9HR\_UP | MSigDB lists | CHEN\_HOXA5\_TARGETS\_9HR\_UP | 186 | 18 | 12187 | 179 | Ifi209,Ifi207,Ifi204,Tiparp,Atf3,Ptgs2,Ip6k2,Maff,Birc3,Nfkbia,Bach1,Gadd45b,Tnfaip3,Gem,Nfkbie,Irf7,Ifi211,Rbm39 | | 2.600e-10 | -22.07 | GSE360\_L\_DONOVANI\_VS\_B\_MALAYI\_HIGH\_DOSE\_DC\_UP | MSigDB lists | GSE360\_L\_DONOVANI\_VS\_B\_MALAYI\_HIGH\_DOSE\_DC\_UP | 142 | 16 | 12187 | 179 | Tnfaip3,Gadd45b,Ccl4,Nfkbia,Rgs16,Irf7,Cflar,Ier3,Stx11,Nfkb1,Il1rn,Timp1,Gadd45g,Vcam1,Csf2rb,Isg15 | | 2.780e-10 | -22.00 | FOSTER\_TOLERANT\_MACROPHAGE\_UP | MSigDB lists | FOSTER\_TOLERANT\_MACROPHAGE\_UP | 122 | 15 | 12187 | 179 | Il1rn,Hp,Cp,Lcn2,Thbs1,Sgk1,Ifi44,Irak3,Pik3ap1,Msr1,Xaf1,Igsf6,Gem,Ptges,Irf7 | | 2.903e-10 | -21.96 | GSE21360\_NAIVE\_VS\_QUATERNARY\_MEMORY\_CD8\_TCELL\_UP | MSigDB lists | GSE21360\_NAIVE\_VS\_QUATERNARY\_MEMORY\_CD8\_TCELL\_UP | 165 | 17 | 12187 | 179 | Parp12,Oasl1,Ripk1,Ifit2,Usp18,Xaf1,Rtp4,Irf7,Phf11d,Ifi44,Phf11b,Ifit3b,Trim25,Irf9,Ifitm2,Herc6,Isg15 | | 2.949e-10 | -21.94 | GO\_RESPONSE\_TO\_OXYGEN\_CONTAINING\_COMPOUND | MSigDB lists | GO\_RESPONSE\_TO\_OXYGEN\_CONTAINING\_COMPOUND | 1042 | 43 | 12187 | 179 | Csf3,Ptges,Cxcl1,Cmpk2,S100a8,Pygm,Sbno2,Pik3r1,Spi1,Cybb,Nfe2l2,Il6,Tnfaip3,Ccl12,Selp,Cd14,P2ry6,Irak3,Cxcl16,Ptpn1,Thbs1,Nfkb1,C5ar1,Fzd4,Nfkb2,Tnf,Casp4,Nfkbia,Trib1,Ptgs2,Sele,Icam1,Msn,Errfi1,Cdkn1a,Adamts1,Socs3,Vcam1,Col4a1,Hp,Timp1,Tnip3,Kcna5 | | 2.954e-10 | -21.94 | extracellular space | cellular component | GO:0005615 | 869 | 40 | 13825 | 212 | Lcn2,Ccl4,Retnlg,Selp,Il4ra,S100a8,Saa1,Angptl4,Tnf,Thbs1,Col4a1,Cp,Cxcl9,Cxcl16,Msr1,Ccl2,Cd14,Il1rn,Cxcl1,Adamts9,Xdh,Vcam1,Ccl7,Mmp8,Fstl1,Sele,Olfml2b,A2m,Il6,Prg4,S100a9,Tnfsf10,Ccl12,Wfdc21,Hp,Il1a,Icam1,Timp1,Csf3,Serpina3f | | 2.982e-10 | -21.93 | regulation of MAPK cascade | biological process | GO:0043408 | 605 | 33 | 13711 | 214 | Ccl12,Iqgap1,Ptpn1,C5ar1,Mmp8,Ccl2,Atf3,Ksr1,Xdh,Pik3ap1,Tnf,Tnip1,Cflar,Ripk1,Irak3,Il1rn,Ncf1,Fzd4,Map3k6,Map3k8,Pik3r5,Ccl4,Il6,Errfi1,Akap12,Il1a,Icam1,P2ry6,Gadd45g,Gadd45b,Ccl7,Trib1,Thbs1 | | 3.126e-10 | -21.89 | TAKEDA\_TARGETS\_OF\_NUP98\_HOXA9\_FUSION\_10D\_UP | MSigDB lists | TAKEDA\_TARGETS\_OF\_NUP98\_HOXA9\_FUSION\_10D\_UP | 123 | 15 | 12187 | 179 | Oasl1,AA467197,Usp18,Xaf1,Ifit2,Rtp4,Cmpk2,Irf7,Gem,Samd9l,Ifit3b,Ifi44,Thbs1,Ptgs2,Isg15 | | 3.193e-10 | -21.86 | GSE19923\_WT\_VS\_HEB\_AND\_E2A\_KO\_DP\_THYMOCYTE\_DN | MSigDB lists | GSE19923\_WT\_VS\_HEB\_AND\_E2A\_KO\_DP\_THYMOCYTE\_DN | 166 | 17 | 12187 | 179 | Cflar,Gem,Map3k8,Tnf,Ccl4,Ccl2,Nfkbia,Il6,Zfp36,Errfi1,Sgk1,Socs3,Nfkbiz,Dnajb1,Tiparp,Ier3,Atf3 | | 3.542e-10 | -21.76 | regulation of cellular protein metabolic process | biological process | GO:0032268 | 2101 | 69 | 13711 | 214 | P2ry6,Lgals9,Gadd45g,Thbs1,Errfi1,Icam1,Plaur,Pik3r5,Birc3,Map3k8,Gbp4,Csf3,Tnfaip8,Irak3,Il1rn,Ccl2,Xdh,Ksr1,Tnfsf10,Tnfaip3,Irgm2,Wfdc21,Bcl3,S100a9,Ccl12,S100a8,Iqgap1,A2m,Ptpn1,Gadd45b,Ccnd2,Pik3r1,Parp14,Trib1,Ctla2a,Ccl7,Dtx3l,Socs3,Cdkn1a,Il6,Nfkbia,Il1a,Akap12,Casp4,Ptgs2,Ccl4,Spi1,Rasip1,Parp9,Sdc4,Isg15,Map3k6,Fzd4,Fam107a,Timp1,Tnip1,Ripk1,Cflar,Ncf1,Hspb1,Atf3,Zfp36,Pik3ap1,Tnf,Msn,Mmp8,Serpina3f,Hspa5,C5ar1 | | 3.564e-10 | -21.76 | GSE360\_CTRL\_VS\_T\_GONDII\_DC\_DN | MSigDB lists | GSE360\_CTRL\_VS\_T\_GONDII\_DC\_DN | 145 | 16 | 12187 | 179 | Nfkb2,Cflar,Tnfaip3,Gadd45b,Nfkbia,Trib1,Csf2rb,Tap1,Ifitm3,Tagln2,Stx11,Nfkb1,Cp,Cebpd,Socs3,Ifit3b | | 3.692e-10 | -21.72 | regulation of biological process | biological process | GO:0050789 | 7871 | 166 | 13711 | 214 | Cxcl1,Dnajb1,Rrp8,Mt2,Hp,Prg4,Ifit2,Csf2rb,Tnf,Robo4,Zfp36,Ripk1,Cd14,Gpr84,Cflar,Ifi207,Hspb1,Rhoc,Fstl1,Tnip1,Fzd4,Ifi209,Parp9,Sbno2,Ptges,Sdc4,Ccl4,Spi1,Ifitm6,Ier3,Ptgs2,Sgk1,Il1a,Gem,Oasl2,Casp4,Cdkn1a,Il6,Ctla2a,Trib1,Dtx3l,Irf7,Socs3,Gadd45b,Pik3r1,Ccl12,Irf2,Tap1,S100a8,A2m,Usp18,Sgk3,Irf9,Irgm2,Bcl3,Samd9l,Plek,Xdh,Tnfsf10,Ksr1,Adamts9,Irak3,Arid5b,Ifi204,Osmr,Gbp4,Adamts1,Nfkb1,Kcna5,Bach1,Il17ra,Plaur,Trim56,Sox11,Mxd4,Cybb,Errfi1,Ifitm3,Thbs1,P2ry6,Banp,Rbm39,Clic4,Hspa5,Maff,C5ar1,Tgm2,Rasd1,Mmp8,Nfe2l2,Serpina3f,Angptl4,Msn,Noct,Selp,Pik3ap1,Acod1,Rhou,Atf3,Ncf1,Sele,Rnf213,Trim30a,Map3k6,Fam107a,Tiparp,Timp1,Isg15,Slfn2,Ifi211,Xaf1,Cebpd,Rasip1,Trim25,Resf1,Runx1,Nfkbia,Akap12,Il4ra,Slfn3,Ip6k2,Parp14,Ccl7,Ccnd2,Hcar2,Ptpn1,Ifitm2,Nfkbiz,Cxcl16,Iqgap1,Col4a1,Tnip3,S100a9,Vcam1,Wfdc21,Cxcl9,Znfx1,Tnfaip3,Depp1,Ccl2,Tnfaip8,Zbtb16,Csf3,Il1rn,C3ar1,Aff1,Lcn2,Birc3,Map3k8,Nfkbie,Rhoj,Apold1,Irgm1,Pik3r5,Fyb,Nfkb2,Rgs16,Icam1,Mt1,Rassf4,Bbs12,Iigp1,Msr1,Oasl1,Lgals9,Gadd45g | | 3.717e-10 | -21.71 | GO\_NEGATIVE\_REGULATION\_OF\_IMMUNE\_SYSTEM\_PROCESS | MSigDB lists | GO\_NEGATIVE\_REGULATION\_OF\_IMMUNE\_SYSTEM\_PROCESS | 268 | 21 | 12187 | 179 | Cd14,Irak3,Pik3ap1,Tap1,Ifi209,Sox11,Thbs1,Zbtb16,Ifi204,Ifi207,Ifi211,A2m,Runx1,Tnf,Il4ra,Pik3r1,Trib1,Nfkbia,Tnfaip3,Ccl12,Nfe2l2 | | 3.856e-10 | -21.68 | HALLMARK\_HYPOXIA | MSigDB lists | HALLMARK\_HYPOXIA | 168 | 17 | 12187 | 179 | Cdkn1a,Hspa5,Tiparp,Akap12,Cp,Ier3,Atf3,Zfp36,Errfi1,Plaur,Maff,Tgm2,Sdc4,Tnfaip3,Il6,Angptl4,Pygm | | 3.856e-10 | -21.68 | GSE7548\_NAIVE\_VS\_DAY7\_PCC\_IMMUNIZATION\_CD4\_TCELL\_DN | MSigDB lists | GSE7548\_NAIVE\_VS\_DAY7\_PCC\_IMMUNIZATION\_CD4\_TCELL\_DN | 168 | 17 | 12187 | 179 | Oasl1,Ripk1,Xaf1,Ifit2,Irf7,Ifi211,Ifi207,Ifi204,Ifit3b,Ifi44,Ifi209,Tap1,Csf2rb,Zfp36,Ifitm2,Isg15,Ifitm3 | | 3.952e-10 | -21.65 | GSE2706\_UNSTIM\_VS\_8H\_LPS\_DC\_DN | MSigDB lists | GSE2706\_UNSTIM\_VS\_8H\_LPS\_DC\_DN | 146 | 16 | 12187 | 179 | Sox11,Socs3,Nfkb1,Stx11,Rnf213,Tnip1,Icam1,Parp14,Trib1,Tnfaip3,AA467197,Il6,Dtx3l,Irf7,Tnfaip8,Rtp4 | | 4.039e-10 | -21.63 | positive regulation of signal transduction | biological process | GO:0009967 | 1217 | 49 | 13711 | 214 | C5ar1,Tgm2,Selp,Mmp8,Atf3,Pik3ap1,Tnf,Ripk1,Cd14,Cflar,Ncf1,Parp9,Fzd4,Map3k6,Trim25,Ccl4,Il6,Akap12,Il1a,Casp4,Gadd45b,Pik3r1,Parp14,Ccl7,Irf7,Ccl12,Iqgap1,S100a8,Ptpn1,Irgm2,S100a9,Ccl2,Xdh,Tnfsf10,Ksr1,Tnfaip3,Csf3,Il1rn,Nfkb1,Map3k8,Plaur,Pik3r5,Irgm1,Sox11,Icam1,P2ry6,Lgals9,Gadd45g,Thbs1 | | 4.048e-10 | -21.63 | positive regulation of response to biotic stimulus | biological process | GO:0002833 | 158 | 17 | 13711 | 214 | Irgm2,Ifi211,Irgm1,Parp9,Tnip3,Ifi209,Cxcl1,Lgals9,Ifi204,Tnip1,Cd14,Irf7,Irak3,Pik3ap1,Nfkbia,Tnf,Acod1 | | 4.127e-10 | -21.61 | DEBOSSCHER\_NFKB\_TARGETS\_REPRESSED\_BY\_GLUCOCORTICOIDS | MSigDB lists | DEBOSSCHER\_NFKB\_TARGETS\_REPRESSED\_BY\_GLUCOCORTICOIDS | 14 | 7 | 12187 | 179 | Il6,Icam1,Sele,Vcam1,Ccl2,Tnf,Ptgs2 | | 4.233e-10 | -21.58 | GSE43955\_TH0\_VS\_TGFB\_IL6\_TH17\_ACT\_CD4\_TCELL\_4H\_UP | MSigDB lists | GSE43955\_TH0\_VS\_TGFB\_IL6\_TH17\_ACT\_CD4\_TCELL\_4H\_UP | 169 | 17 | 12187 | 179 | Cebpd,Gadd45g,Socs3,Adamts1,Sox11,Errfi1,Sele,Selp,Saa1,Ptgs2,Il6,Bach1,Ccl4,Map3k8,Il4ra,Birc3,Gbp3 | | 4.233e-10 | -21.58 | GSE22140\_HEALTHY\_VS\_ARTHRITIC\_GERMFREE\_MOUSE\_CD4\_TCELL\_DN | MSigDB lists | GSE22140\_HEALTHY\_VS\_ARTHRITIC\_GERMFREE\_MOUSE\_CD4\_TCELL\_DN | 169 | 17 | 12187 | 179 | Gem,Rgs16,Nfkb2,Tnf,Ifit2,Trib1,Ccl4,Oasl1,Trim25,Icam1,Thbs1,Ifi44,Cdkn1a,Ifit3b,Nfkb1,Timp1,Ier3 | | 4.233e-10 | -21.58 | GSE22935\_WT\_VS\_MYD88\_KO\_MACROPHAGE\_12H\_MBOVIS\_BCG\_STIM\_DN | MSigDB lists | GSE22935\_WT\_VS\_MYD88\_KO\_MACROPHAGE\_12H\_MBOVIS\_BCG\_STIM\_DN | 169 | 17 | 12187 | 179 | Slfn4,Igsf6,Ptges,Nfe2l2,Tnfaip3,Gpr84,Parp9,Clic4,Slfn3,Plek,Isg15,Lcn2,Ier3,Timp1,Ksr1,Nfkbiz,Aff1 | | 4.378e-10 | -21.55 | GSE30971\_CTRL\_VS\_LPS\_STIM\_MACROPHAGE\_WBP7\_HET\_2H\_UP | MSigDB lists | GSE30971\_CTRL\_VS\_LPS\_STIM\_MACROPHAGE\_WBP7\_HET\_2H\_UP | 147 | 16 | 12187 | 179 | Tnf,Nfkbia,Ccl4,Il6,Ptges,Cxcl1,Rgs16,Socs3,Il1a,Nfkb1,Tnip3,Ier3,Ptgs2,Irak3,Clic4,Plaur | | 4.494e-10 | -21.52 | cytokine activity | molecular function | GO:0005125 | 100 | 14 | 13516 | 211 | Ccl7,Ccl12,Tnf,Tnfsf10,Cxcl1,Timp1,Il1rn,Csf3,Il1a,Cxcl16,Ccl2,Il6,Cxcl9,Ccl4 | | 4.644e-10 | -21.49 | GSE43863\_DAY6\_EFF\_VS\_DAY150\_MEM\_TFH\_CD4\_TCELL\_UP | MSigDB lists | GSE43863\_DAY6\_EFF\_VS\_DAY150\_MEM\_TFH\_CD4\_TCELL\_UP | 170 | 17 | 12187 | 179 | Xaf1,Usp18,Ifit2,Lgals9,Parp12,Bach1,Irf7,Rtp4,Cmpk2,Rbm39,Ifit3b,Ifi44,Hspa5,Znfx1,Rasd1,Saa1,Pik3ap1 | | 4.847e-10 | -21.45 | GSE42724\_MEMORY\_VS\_B1\_BCELL\_DN | MSigDB lists | GSE42724\_MEMORY\_VS\_B1\_BCELL\_DN | 148 | 16 | 12187 | 179 | AA467197,Tnfaip3,Gadd45b,Sdc4,Nfkbia,Gbp3,Cflar,Arid5b,Nfkb1,Znfx1,Cdkn1a,Socs3,Nfkbiz,Parp9,Ptpn1,Tap1 | | 4.847e-10 | -21.45 | GSE6259\_DEC205\_POS\_DC\_VS\_BCELL\_DN | MSigDB lists | GSE6259\_DEC205\_POS\_DC\_VS\_BCELL\_DN | 148 | 16 | 12187 | 179 | Znfx1,Ifi44,Phf11b,Pik3ap1,Icam1,Isg15,Tnip1,Gadd45b,Msr1,Ifit2,Nfkbia,Xaf1,Parp14,Gbp3,Irf7,Phf11d | | 4.847e-10 | -21.45 | GSE12845\_PRE\_GC\_VS\_DARKZONE\_GC\_TONSIL\_BCELL\_UP | MSigDB lists | GSE12845\_PRE\_GC\_VS\_DARKZONE\_GC\_TONSIL\_BCELL\_UP | 148 | 16 | 12187 | 179 | Cd14,Ifitm2,Ifitm3,Tap1,Zfp36,Rbm47,Il17ra,Ifi44,Ier3,Cxcl1,Nfkbia,Ccnd2,Il6,Parp12,Tubb6,Rhoc | | 4.969e-10 | -21.42 | BOWIE\_RESPONSE\_TO\_EXTRACELLULAR\_MATRIX | MSigDB lists | BOWIE\_RESPONSE\_TO\_EXTRACELLULAR\_MATRIX | 22 | 8 | 12187 | 179 | Ifi209,Ifit2,Ifit3b,Ifi204,Ifi207,Isg15,Ifi211,Irf7 | | 5.091e-10 | -21.40 | GSE17301\_CTRL\_VS\_48H\_ACD3\_ACD28\_STIM\_CD8\_TCELL\_DN | MSigDB lists | GSE17301\_CTRL\_VS\_48H\_ACD3\_ACD28\_STIM\_CD8\_TCELL\_DN | 171 | 17 | 12187 | 179 | Igsf6,Nfkbie,Pik3r5,Rassf4,Tnfaip3,Tnf,Map3k8,Spi1,Gpr84,Nfkbia,Casp4,Irak3,Icam1,Tnip1,Stx11,Nfkb1,Nfkbiz | | 5.139e-10 | -21.39 | positive regulation of cellular protein metabolic process | biological process | GO:0032270 | 1309 | 51 | 13711 | 214 | Xdh,Ksr1,Tnfsf10,Ccl2,Tnfaip3,Il1rn,Csf3,S100a8,Iqgap1,Ccl12,Ptpn1,Bcl3,Irgm2,S100a9,Icam1,Gadd45g,P2ry6,Lgals9,Thbs1,Map3k8,Birc3,Pik3r5,Plaur,Tnf,Tnip1,Ncf1,Ripk1,Cflar,C5ar1,Hspa5,Msn,Mmp8,Il6,Cdkn1a,Casp4,Nfkbia,Akap12,Il1a,Gadd45b,Ccnd2,Pik3r1,Parp14,Trib1,Ccl7,Parp9,Sdc4,Map3k6,Fzd4,Fam107a,Ptgs2,Ccl4 | | 5.222e-10 | -21.37 | GO\_REGULATION\_OF\_INNATE\_IMMUNE\_RESPONSE | MSigDB lists | GO\_REGULATION\_OF\_INNATE\_IMMUNE\_RESPONSE | 273 | 21 | 12187 | 179 | A2m,Irf7,Ifi211,Nfkbia,Usp18,Birc3,Ripk1,Tnfaip3,Cd14,Tnip1,Tap1,Ptpn1,Parp9,Pik3ap1,Irak3,Socs3,Ifi209,Ifi207,Nfkb1,Tnip3,Ifi204 | | 5.252e-10 | -21.37 | RODWELL\_AGING\_KIDNEY\_UP | MSigDB lists | RODWELL\_AGING\_KIDNEY\_UP | 360 | 24 | 12187 | 179 | Ccl12,Map3k8,Ccnd2,Msr1,Osmr,Dtx3l,Ms4a6d,Nfkbiz,Cxcl16,Tap1,Il17ra,Cd14,Rnf213,AA467197,Parp12,Parp14,Birc3,A2m,Timp1,Samd9l,Vcam1,Socs3,Irf9,Tagln2 | | 5.282e-10 | -21.36 | defense response to protozoan | biological process | GO:0042832 | 21 | 8 | 13711 | 214 | Gbp3,Il4ra,Il6,Iigp1,Bcl3,Gbp7,Irgm2,Gbp6 | | 5.793e-10 | -21.27 | positive regulation of intracellular signal transduction | biological process | GO:1902533 | 762 | 37 | 13711 | 214 | Il6,Akap12,Sox11,Il1a,Icam1,P2ry6,Gadd45b,Gadd45g,Ccl7,Thbs1,Fzd4,Map3k6,Map3k8,Trim25,Pik3r5,Ccl4,Ccl2,Xdh,Tnfsf10,Ksr1,Pik3ap1,Tnf,Csf3,Ripk1,Cd14,Cflar,Il1rn,Ncf1,Ccl12,S100a8,Iqgap1,Ptpn1,C5ar1,Tgm2,Selp,Mmp8,S100a9 | | 5.926e-10 | -21.25 | GSE1432\_CTRL\_VS\_IFNG\_6H\_MICROGLIA\_DN | MSigDB lists | GSE1432\_CTRL\_VS\_IFNG\_6H\_MICROGLIA\_DN | 150 | 16 | 12187 | 179 | Ifi44,Phf11b,Akap12,Stx11,Isg15,Herc6,Tap1,Csf2rb,Plek,Icam1,Casp4,Parp12,Irf7,Phf11d,Tnfsf10,Sbno2 | | 5.926e-10 | -21.25 | GSE21360\_NAIVE\_VS\_SECONDARY\_MEMORY\_CD8\_TCELL\_UP | MSigDB lists | GSE21360\_NAIVE\_VS\_SECONDARY\_MEMORY\_CD8\_TCELL\_UP | 150 | 16 | 12187 | 179 | Gadd45b,Cybb,Sdc4,Nfkbia,Tnf,Bcl3,Pik3r5,Csf3,Nfkbie,Ier3,Cp,Nfkb1,Il1rn,Ptpn1,Irak3,Ifitm2 | | 6.018e-10 | -21.23 | positive regulation of MAPK cascade | biological process | GO:0043410 | 427 | 27 | 13711 | 214 | Il1a,Akap12,Icam1,Il6,Ccl7,Thbs1,P2ry6,Gadd45b,Gadd45g,Fzd4,Map3k6,Map3k8,Ccl4,Pik3r5,Tnf,Ccl2,Xdh,Ksr1,Ripk1,Cflar,Ncf1,Il1rn,Ptpn1,C5ar1,Ccl12,Iqgap1,Mmp8 | | 6.096e-10 | -21.22 | positive regulation of protein phosphorylation | biological process | GO:0001934 | 800 | 38 | 13711 | 214 | Tnf,Ccl2,Xdh,Ksr1,Ripk1,Csf3,Cflar,Il1rn,Ncf1,Ptpn1,C5ar1,Ccl12,Iqgap1,Mmp8,Irgm2,Akap12,Il1a,Icam1,Cdkn1a,Il6,Parp14,Ccl7,Thbs1,P2ry6,Lgals9,Gadd45b,Pik3r1,Gadd45g,Ccnd2,Map3k6,Fzd4,Map3k8,Parp9,Sdc4,Ccl4,Plaur,Pik3r5,Ptgs2 | | 6.585e-10 | -21.14 | GO\_POSITIVE\_REGULATION\_OF\_PROTEIN\_METABOLIC\_PROCESS | MSigDB lists | GO\_POSITIVE\_REGULATION\_OF\_PROTEIN\_METABOLIC\_PROCESS | 1155 | 45 | 12187 | 179 | Map3k6,Ptpn1,Hspa5,Bcl3,Ifi211,S100a8,Ccl2,Ccnd2,Gadd45b,Ripk1,Tnfaip3,Saa1,Plek,Icam1,Ifi209,Cdkn1a,Ksr1,Ifi204,Fzd4,S100a9,Tnfsf10,Trib1,Thbs1,Gadd45g,Il1a,Tiparp,Pik3r5,Csf3,Iqgap1,Ccl4,Map3k8,Nfe2l2,Il6,Ccl12,Msn,Plaur,Hspb1,Socs3,Ifi207,C5ar1,Xdh,Sdc4,Nfkbia,Birc3,Tnf | | 6.684e-10 | -21.13 | GSE7509\_DC\_VS\_MONOCYTE\_DN | MSigDB lists | GSE7509\_DC\_VS\_MONOCYTE\_DN | 174 | 17 | 12187 | 179 | Ifit3b,Znfx1,Hp,Cd14,Saa1,Clic4,Tap1,Usp18,Spi1,Nfkbia,Casp4,Tnf,Cybb,Ptges,Irgm2,Cmpk2,S100a8 | | 6.684e-10 | -21.13 | GSE339\_EX\_VIVO\_VS\_IN\_CULTURE\_CD8POS\_DC\_DN | MSigDB lists | GSE339\_EX\_VIVO\_VS\_IN\_CULTURE\_CD8POS\_DC\_DN | 174 | 17 | 12187 | 179 | Trim25,Csf2rb,Isg15,Ifitm2,Tnip1,Tiparp,Znfx1,Cdkn1a,Hspa5,Cmpk2,Nfkb2,Irgm2,Bcl3,Gadd45b,Pik3r1,Usp18,Nfkbia | | 6.895e-10 | -21.10 | regulation of catalytic activity | biological process | GO:0050790 | 1714 | 60 | 13711 | 214 | Tnfaip3,Plek,Xdh,Tnfsf10,Ksr1,Ccl2,Il1rn,Irak3,Tnfaip8,Ptpn1,Iqgap1,S100a8,A2m,Ccl12,S100a9,Irgm2,Wfdc21,Icam1,Rgs16,Errfi1,Thbs1,Oasl1,Gadd45g,Lgals9,Map3k8,Birc3,Nfkb1,Pik3r5,Plaur,Tnf,Zfp36,Ncf1,Hspb1,Sele,Ripk1,Cflar,Rhoc,Dnajb1,C5ar1,Hp,Serpina3f,Angptl4,Oasl2,Casp4,Il6,Cdkn1a,Dtx3l,Trib1,Ccl7,Gadd45b,Ccnd2,Pik3r1,Timp1,Map3k6,Fzd4,Parp9,Sdc4,Rasip1,Ccl4,Ptgs2 | | 7.023e-10 | -21.08 | positive regulation of nitrogen compound metabolic process | biological process | GO:0051173 | 2525 | 77 | 13711 | 214 | Atf3,Zfp36,Tnf,Tnip1,Trim30a,Ncf1,Hspb1,Ripk1,Cflar,Maff,C5ar1,Hspa5,Msn,Nfe2l2,Mmp8,Il6,Cdkn1a,Casp4,Nfkbia,Akap12,Il1a,Gadd45b,Ccnd2,Pik3r1,Dtx3l,Irf7,Trib1,Parp14,Ccl7,Parp9,Sbno2,Sdc4,Tiparp,Fzd4,Map3k6,Fam107a,Runx1,Ptgs2,Ier3,Cebpd,Spi1,Ccl4,Xdh,Ksr1,Tnfsf10,Ccl2,Tnfaip3,Il1rn,Arid5b,Aff1,C3ar1,Zbtb16,Csf3,Iqgap1,S100a8,Ccl12,Irf2,Ptpn1,Bcl3,Irgm2,S100a9,Cybb,Icam1,Nfkb2,Sox11,Banp,Rbm39,Gadd45g,P2ry6,Lgals9,Thbs1,Nfkb1,Map3k8,Birc3,Pik3r5,Plaur,Bach1 | | 7.221e-10 | -21.05 | GSE2706\_UNSTIM\_VS\_8H\_LPS\_AND\_R848\_DC\_DN | MSigDB lists | GSE2706\_UNSTIM\_VS\_8H\_LPS\_AND\_R848\_DC\_DN | 152 | 16 | 12187 | 179 | Irf7,Cmpk2,Tnfaip8,Map3k8,Usp18,Parp14,Gadd45b,AA467197,Herc6,Tnip1,Icam1,Socs3,Tnip3,Nfkb1,Stx11,Znfx1 | | 7.963e-10 | -20.95 | GSE9509\_LPS\_VS\_LPS\_AND\_IL10\_STIM\_IL10\_KO\_MACROPHAGE\_10MIN\_UP | MSigDB lists | GSE9509\_LPS\_VS\_LPS\_AND\_IL10\_STIM\_IL10\_KO\_MACROPHAGE\_10MIN\_UP | 153 | 16 | 12187 | 179 | Cd14,Pik3ap1,Plaur,Ptpn1,Socs3,Gadd45g,Gem,Cxcl1,Rhoj,Cmpk2,Rhou,Ccl4,Trib1,Tubb6,Ccl12,Oasl1 | | 7.963e-10 | -20.95 | GSE9988\_LOW\_LPS\_VS\_ANTI\_TREM1\_AND\_LPS\_MONOCYTE\_UP | MSigDB lists | GSE9988\_LOW\_LPS\_VS\_ANTI\_TREM1\_AND\_LPS\_MONOCYTE\_UP | 153 | 16 | 12187 | 179 | Thbs1,Nfkbiz,Tnip3,Stx11,Tnip1,Pik3ap1,Clic4,Rhou,Map3k8,Birc3,Cybb,Tnfaip3,Nfe2l2,Oasl1,Csf3,Pik3r5 | | 8.334e-10 | -20.91 | granulocyte chemotaxis | biological process | GO:0071621 | 56 | 11 | 13711 | 214 | Il1rn,Ccl7,Cxcl9,Ccl2,Ccl4,S100a9,Il17ra,C5ar1,Cxcl1,S100a8,Ccl12 | | 8.591e-10 | -20.88 | positive regulation of protein modification process | biological process | GO:0031401 | 1001 | 43 | 13711 | 214 | Tnf,Ksr1,Xdh,Ccl2,Ncf1,Il1rn,Cflar,Ripk1,Csf3,Tnip1,C5ar1,Ptpn1,Hspa5,Iqgap1,Ccl12,Mmp8,Irgm2,Icam1,Akap12,Il1a,Il6,Cdkn1a,Thbs1,Ccl7,Trib1,Parp14,Pik3r1,Gadd45g,Ccnd2,Gadd45b,Lgals9,P2ry6,Map3k8,Fam107a,Birc3,Fzd4,Map3k6,Sdc4,Parp9,Ccl4,Ptgs2,Pik3r5,Plaur | | 8.773e-10 | -20.85 | GSE30083\_SP1\_VS\_SP4\_THYMOCYTE\_DN | MSigDB lists | GSE30083\_SP1\_VS\_SP4\_THYMOCYTE\_DN | 154 | 16 | 12187 | 179 | Tap1,Il17ra,Rnf213,Herc6,Ms4a6d,Samd9l,Zbtb16,Sgk1,Ifi44,Xdh,Irf7,Gbp7,Dtx3l,Irgm2,Oasl1,Sntb2 | | 8.773e-10 | -20.85 | GSE6092\_IFNG\_VS\_IFNG\_AND\_B\_BURGDORFERI\_INF\_ENDOTHELIAL\_CELL\_DN | MSigDB lists | GSE6092\_IFNG\_VS\_IFNG\_AND\_B\_BURGDORFERI\_INF\_ENDOTHELIAL\_CELL\_DN | 154 | 16 | 12187 | 179 | Phf11b,Socs3,Stx11,Cp,Znfx1,Ncf1,Cd14,Ifit2,Sdc4,Usp18,Parp12,Cflar,Phf11d,Igsf6,Cmpk2,Tnfsf10 | | 8.782e-10 | -20.85 | regulation of reactive oxygen species metabolic process | biological process | GO:2000377 | 166 | 17 | 13711 | 214 | Hp,Birc3,Cxcl1,Ier3,Ptgs2,Mmp8,Nfe2l2,Cdkn1a,Il6,Xdh,Icam1,Tnf,Acod1,Trim30a,Cflar,Ripk1,Thbs1 | | 8.860e-10 | -20.84 | NOD-like receptor signaling pathway | KEGG pathways | mmu04621 | 133 | 17 | 5248 | 107 | Cybb,Ifi204,Nfkb1,Il6,Irf7,Ripk1,Irf9,Birc3,Tnf,Tnfaip3,Gbp7,Cxcl1,Ccl12,Nfkbia,Ccl2,Gbp3,Casp4 | | 8.860e-10 | -20.84 | NOD-like receptor signaling pathway | KEGG pathways | ko04621 | 133 | 17 | 5248 | 107 | Nfkbia,Ccl2,Gbp3,Casp4,Tnfaip3,Gbp7,Cxcl1,Ccl12,Nfkb1,Il6,Ripk1,Irf7,Irf9,Birc3,Tnf,Ifi204,Cybb | | 9.258e-10 | -20.80 | SANA\_TNF\_SIGNALING\_UP | MSigDB lists | SANA\_TNF\_SIGNALING\_UP | 60 | 11 | 12187 | 179 | Tnfaip3,Ccl12,Samd9l,Vcam1,Parp14,Nfkbia,Birc3,Icam1,Cmpk2,Cxcl1,Tnip1 | | 9.744e-10 | -20.75 | Malaria | KEGG pathways | mmu05144 | 35 | 10 | 5248 | 107 | Thbs1,Il6,Sele,Ccl2,Vcam1,Tnf,Icam1,Selp,Ccl12,Csf3 | | 9.744e-10 | -20.75 | Malaria | KEGG pathways | ko05144 | 35 | 10 | 5248 | 107 | Il6,Sele,Thbs1,Tnf,Icam1,Ccl2,Vcam1,Selp,Csf3,Ccl12 | | 1.010e-09 | -20.71 | CHIARADONNA\_NEOPLASTIC\_TRANSFORMATION\_CDC25\_UP | MSigDB lists | CHIARADONNA\_NEOPLASTIC\_TRANSFORMATION\_CDC25\_UP | 113 | 14 | 12187 | 179 | Errfi1,Cebpd,Ifi207,Ifi204,Vcam1,Thbs1,Ifi209,Xdh,Osmr,Ifi211,Il6,Ccl12,Bach1,Casp4 | | 1.023e-09 | -20.70 | GSE45365\_CD8A\_DC\_VS\_CD11B\_DC\_IFNAR\_KO\_MCMV\_INFECTION\_DN | MSigDB lists | GSE45365\_CD8A\_DC\_VS\_CD11B\_DC\_IFNAR\_KO\_MCMV\_INFECTION\_DN | 94 | 13 | 12187 | 179 | Nfkb2,Cxcl1,Tnfaip3,Ccl12,Slc24a4,Nfkbia,Sdc4,Map3k8,Ptgs2,Atf3,Ier3,Nfkbiz,Ifi44 | | 1.115e-09 | -20.61 | MODULE\_263 | MSigDB lists | MODULE\_263 | 24 | 8 | 12187 | 179 | Plaur,Cxcl1,C5ar1,C3ar1,Ccl12,Il1a,Ccl4,Ccl2 | | 1.116e-09 | -20.61 | KEGG\_APOPTOSIS | MSigDB lists | KEGG\_APOPTOSIS | 77 | 12 | 12187 | 179 | Irak3,Csf2rb,Nfkb1,Il1a,Tnfsf10,Cflar,Pik3r5,Ripk1,Tnf,Birc3,Pik3r1,Nfkbia | | 1.116e-09 | -20.61 | WINZEN\_DEGRADED\_VIA\_KHSRP | MSigDB lists | WINZEN\_DEGRADED\_VIA\_KHSRP | 77 | 12 | 12187 | 179 | Cxcl1,Gem,Sdc4,Ifit2,Tnf,Il6,Ptgs2,Errfi1,Plaur,Zfp36,Sele,Nfkbiz | | 1.136e-09 | -20.60 | cellular response to lipid | biological process | GO:0071396 | 321 | 23 | 13711 | 214 | Gbp6,Irgm2,Msn,Cxcl1,Cxcl16,Ccl12,Tnip3,Cd14,Il1rn,Cxcl9,Acod1,Tnf,Tnfaip3,Ccl2,Zfp36,Sgk1,Fam107a,Sbno2,Cmpk2,Nfkb1,P2ry6,Nfkbia,Il6 | | 1.176e-09 | -20.56 | BROWNE\_INTERFERON\_RESPONSIVE\_GENES | MSigDB lists | BROWNE\_INTERFERON\_RESPONSIVE\_GENES | 47 | 10 | 12187 | 179 | Stx11,Oasl1,Ifi44,Ifit2,Ifit3b,Xaf1,Tnfsf10,Tap1,Isg15,Irf7 | | 1.176e-09 | -20.56 | BURTON\_ADIPOGENESIS\_PEAK\_AT\_2HR | MSigDB lists | BURTON\_ADIPOGENESIS\_PEAK\_AT\_2HR | 47 | 10 | 12187 | 179 | Errfi1,Zfp36,Rhoj,Ptgs2,Cebpd,Il6,Ier3,Gadd45g,Dnajb1,Thbs1 | | 1.176e-09 | -20.56 | LINDSTEDT\_DENDRITIC\_CELL\_MATURATION\_B | MSigDB lists | LINDSTEDT\_DENDRITIC\_CELL\_MATURATION\_B | 47 | 10 | 12187 | 179 | Tnip1,Cflar,Nfkb2,Tap1,Sdc4,Nfkbia,Maff,Birc3,Tnfaip3,Nfkb1 | | 1.177e-09 | -20.56 | GSE17974\_IL4\_AND\_ANTI\_IL12\_VS\_UNTREATED\_72H\_ACT\_CD4\_TCELL\_DN | MSigDB lists | GSE17974\_IL4\_AND\_ANTI\_IL12\_VS\_UNTREATED\_72H\_ACT\_CD4\_TCELL\_DN | 135 | 15 | 12187 | 179 | Parp14,Rhou,Birc3,Maff,Gbp3,Tnfsf10,Arid5b,Dtx3l,Stx11,Ksr1,Gadd45g,Samd9l,Parp9,Tap1,Isg15 | | 1.190e-09 | -20.55 | positive regulation of cellular metabolic process | biological process | GO:0031325 | 2654 | 79 | 13711 | 214 | Tnip1,Ncf1,Sele,Hspb1,Ripk1,Cflar,Atf3,Zfp36,Acod1,Tnf,Msn,Nfe2l2,Mmp8,C5ar1,Maff,Cxcl1,Hspa5,Gadd45b,Pik3r1,Ccnd2,Dtx3l,Irf7,Trib1,Parp14,Ccl7,Il6,Cdkn1a,Casp4,Nfkbia,Il1a,Akap12,Runx1,Ptgs2,Cebpd,Spi1,Ccl4,Parp9,Sbno2,Sdc4,Map3k6,Fzd4,Fam107a,Il1rn,Aff1,C3ar1,Arid5b,Zbtb16,Csf3,Xdh,Ksr1,Tnfsf10,Ccl2,Tnfaip3,Plek,Bcl3,Irgm2,S100a9,Iqgap1,S100a8,Irf2,Ccl12,Ptpn1,Rbm39,Gadd45g,Banp,P2ry6,Lgals9,Thbs1,Cybb,Icam1,Nfkb2,Sox11,Pik3r5,Plaur,Bach1,Irgm1,Nfkb1,Map3k8,Birc3 | | 1.302e-09 | -20.46 | GO\_CELLULAR\_RESPONSE\_TO\_INTERFERON\_GAMMA | MSigDB lists | GO\_CELLULAR\_RESPONSE\_TO\_INTERFERON\_GAMMA | 78 | 12 | 12187 | 179 | Icam1,Trim25,Irf2,Irf9,Gbp6,Irf7,Gbp4,Ccl12,Oasl1,Ccl4,Vcam1,Ccl2 | | 1.342e-09 | -20.43 | WANG\_TNF\_TARGETS | MSigDB lists | WANG\_TNF\_TARGETS | 16 | 7 | 12187 | 179 | Nfkbia,Vcam1,Csf3,Selp,Il6,Tnfaip3,Sele | | 1.345e-09 | -20.43 | GO\_LEUKOCYTE\_MIGRATION | MSigDB lists | GO\_LEUKOCYTE\_MIGRATION | 182 | 17 | 12187 | 179 | Vcam1,Selp,Saa1,Cxcl16,Msn,Icam1,Il17ra,Sele,Ccl4,Ccl2,Tnf,Pik3r1,Ccl12,Il6,S100a9,C5ar1,S100a8 | | 1.365e-09 | -20.41 | positive regulation of cell communication | biological process | GO:0010647 | 1389 | 52 | 13711 | 214 | Iqgap1,S100a8,Ccl12,Ptpn1,Irgm2,S100a9,Ksr1,Tnfsf10,Xdh,Ccl2,Tnfaip3,Il1rn,Csf3,Nfkb1,Map3k8,Pik3r5,Plaur,Irgm1,Icam1,Sox11,Gadd45g,Lgals9,P2ry6,Thbs1,Tgm2,C5ar1,Selp,Mmp8,Atf3,Tnf,Pik3ap1,Ncf1,Cd14,Cflar,Ripk1,Parp9,Map3k6,Fzd4,Ptgs2,Runx1,Trim25,Ccl4,Il6,Casp4,Il1a,Akap12,Hcar2,Pik3r1,Gadd45b,Irf7,Ccl7,Parp14 | | 1.408e-09 | -20.38 | regulation of protein modification process | biological process | GO:0031399 | 1521 | 55 | 13711 | 214 | Akap12,Il1a,Cdkn1a,Il6,Ccl7,Trib1,Parp14,Socs3,Dtx3l,Pik3r1,Ccnd2,Gadd45b,Fam107a,Map3k6,Fzd4,Sdc4,Isg15,Parp9,Spi1,Ccl4,Rasip1,Ptgs2,Pik3ap1,Tnf,Atf3,Cflar,Ripk1,Hspb1,Ncf1,Tnip1,Hspa5,C5ar1,Mmp8,Icam1,Errfi1,Thbs1,Lgals9,P2ry6,Gadd45g,Birc3,Gbp4,Map3k8,Plaur,Pik3r5,Tnfaip3,Ccl2,Ksr1,Xdh,Csf3,Irak3,Il1rn,Ptpn1,Ccl12,Iqgap1,Irgm2 | | 1.409e-09 | -20.38 | GSE29618\_PDC\_VS\_MDC\_DN | MSigDB lists | GSE29618\_PDC\_VS\_MDC\_DN | 159 | 16 | 12187 | 179 | Ifitm3,Ifitm2,Ptgs2,Irak3,Msn,Sgk1,Tiparp,Il1rn,Stx11,Timp1,Cebpd,S100a9,Iqgap1,Xaf1,Trib1,Spi1 | | 1.546e-09 | -20.29 | GSE46606\_DAY1\_VS\_DAY3\_CD40L\_IL2\_IL5\_STIMULATED\_IRF4HIGH\_BCELL\_DN | MSigDB lists | GSE46606\_DAY1\_VS\_DAY3\_CD40L\_IL2\_IL5\_STIMULATED\_IRF4HIGH\_BCELL\_DN | 160 | 16 | 12187 | 179 | Dtx3l,Tnfsf10,Ccnd2,Tnf,Trib1,Ripk1,Il6,Oasl1,Plaur,Clic4,Hspa5,Zbtb16,Nfkbiz,Tiparp,Znfx1,Atf3 | | 1.555e-09 | -20.28 | ZHOU\_INFLAMMATORY\_RESPONSE\_FIMA\_UP | MSigDB lists | ZHOU\_INFLAMMATORY\_RESPONSE\_FIMA\_UP | 349 | 23 | 12187 | 179 | Csf3,Rhoj,Angptl4,Cxcl1,Rgs16,Maff,Gpr84,Ccl4,Il6,Tnfaip3,Ptpn1,Il1a,Nfkb1,Ier3,Gem,Fzd4,Nfkb2,Tnf,Sdc4,Nfkbia,Ptgs2,Icam1,Plaur | | 1.604e-09 | -20.25 | GSE22589\_SIV\_VS\_HIV\_AND\_SIV\_INFECTED\_DC\_UP | MSigDB lists | GSE22589\_SIV\_VS\_HIV\_AND\_SIV\_INFECTED\_DC\_UP | 138 | 15 | 12187 | 179 | Cmpk2,Rtp4,Bcl3,Irf7,Tnfaip3,Oasl1,Cybb,Tor3a,Usp18,Nfkbia,Ifit2,Parp9,Irf9,Trim25,Ifit3b | | 1.669e-09 | -20.21 | positive regulation of signaling | biological process | GO:0023056 | 1397 | 52 | 13711 | 214 | Thbs1,P2ry6,Lgals9,Gadd45g,Sox11,Icam1,Irgm1,Plaur,Pik3r5,Map3k8,Nfkb1,Csf3,Il1rn,Tnfaip3,Ccl2,Xdh,Ksr1,Tnfsf10,S100a9,Irgm2,Ptpn1,Ccl12,S100a8,Iqgap1,Parp14,Ccl7,Irf7,Gadd45b,Hcar2,Pik3r1,Akap12,Il1a,Casp4,Il6,Ccl4,Trim25,Runx1,Ptgs2,Map3k6,Fzd4,Parp9,Ripk1,Cd14,Cflar,Ncf1,Pik3ap1,Tnf,Atf3,Mmp8,Selp,C5ar1,Tgm2 | | 1.712e-09 | -20.19 | GO\_NEGATIVE\_REGULATION\_OF\_CELL\_DEATH | MSigDB lists | GO\_NEGATIVE\_REGULATION\_OF\_CELL\_DEATH | 658 | 32 | 12187 | 179 | Nfkbia,Birc3,Tnf,Tnfsf10,Irf7,C5ar1,Cflar,Timp1,Ifit3b,Socs3,Cdkn1a,Plaur,Hspb1,Icam1,Nfe2l2,Il6,Ripk1,Tnfaip3,Ccl12,Pik3r1,Tnfaip8,Bcl3,Angptl4,Csf3,Ier3,Il1a,Nfkb1,Hspa5,Thbs1,Sgk1,Sox11,Ptpn1 | | 1.731e-09 | -20.17 | KIM\_WT1\_TARGETS\_UP | MSigDB lists | KIM\_WT1\_TARGETS\_UP | 185 | 17 | 12187 | 179 | Plaur,Sgk1,Thbs1,Ier3,Akap12,Nfkb1,Arid5b,Nfkbie,Gem,Nfkb2,Trib1,Sdc4,Nfkbia,Il4ra,Maff,Tnfaip3,Ccl12 | | 1.857e-09 | -20.10 | GSE14000\_UNSTIM\_VS\_16H\_LPS\_DC\_DN | MSigDB lists | GSE14000\_UNSTIM\_VS\_16H\_LPS\_DC\_DN | 162 | 16 | 12187 | 179 | Irf2,Parp9,Msn,Irf9,Ifi44,Fstl1,Nfkbiz,Cmpk2,Tnfsf10,Cflar,Dtx3l,Irf7,Rassf4,Gadd45b,Parp12,Nfkbia | | 1.858e-09 | -20.10 | GO\_REGULATION\_OF\_IMMUNE\_RESPONSE | MSigDB lists | GO\_REGULATION\_OF\_IMMUNE\_RESPONSE | 551 | 29 | 12187 | 179 | Tnf,Birc3,Nfkbia,C5ar1,C3ar1,A2m,Irf7,Tnip3,Ifi204,Ifi207,Ifi209,Socs3,Vcam1,Icam1,Tnip1,Ripk1,Tnfaip3,Il6,Pik3r1,Il4ra,Usp18,Ifi211,Nfkb1,Irak3,Pik3ap1,Parp9,Ptpn1,Tap1,Cd14 | | 1.963e-09 | -20.05 | GSE24634\_IL4\_VS\_CTRL\_TREATED\_NAIVE\_CD4\_TCELL\_DAY5\_DN | MSigDB lists | GSE24634\_IL4\_VS\_CTRL\_TREATED\_NAIVE\_CD4\_TCELL\_DAY5\_DN | 140 | 15 | 12187 | 179 | Ifit3b,Phf11b,Ifitm2,Herc6,Tap1,Casp4,Xaf1,Ccnd2,Ifit2,Parp12,Ripk1,Irf7,Arid5b,Phf11d,Nfkb2 | | 1.963e-09 | -20.05 | GSE21360\_NAIVE\_VS\_SECONDARY\_MEMORY\_CD8\_TCELL\_DN | MSigDB lists | GSE21360\_NAIVE\_VS\_SECONDARY\_MEMORY\_CD8\_TCELL\_DN | 140 | 15 | 12187 | 179 | Iqgap1,Irf7,Parp12,Xaf1,Ifit2,Clic4,Trim25,Slfn5,Rnf213,Atf3,Luc7l3,Znfx1,Samd9l,Ifit3b,Ifi44 | | 1.981e-09 | -20.04 | regulation of cytokine biosynthetic process | biological process | GO:0042035 | 76 | 12 | 13711 | 214 | Bcl3,Hspb1,Thbs1,Zfp36,Il6,Prg4,Errfi1,Nfkb1,Cybb,Tnf,Akap12,Il1a | | 1.981e-09 | -20.04 | GO\_EXTRACELLULAR\_SPACE | MSigDB lists | GO\_EXTRACELLULAR\_SPACE | 738 | 34 | 12187 | 179 | S100a8,Adamts9,Csf3,Angptl4,Cxcl1,Il6,Ccl12,Msr1,Il4ra,Ccl2,Ccl4,Cxcl16,Selp,Cd14,Il1a,Mmp8,Lcn2,Thbs1,Tnfsf10,Xdh,A2m,S100a9,Tnf,Sele,Icam1,Hspb1,Msn,Saa1,Hp,Cp,Timp1,Il1rn,Vcam1,Fstl1 | | 2.090e-09 | -19.99 | regulation of intracellular signal transduction | biological process | GO:1902531 | 1320 | 50 | 13711 | 214 | Akap12,Il1a,Il6,Ccl7,Trib1,Gadd45b,Map3k6,Fzd4,Rasip1,Ccl4,Ptgs2,Trim25,Tnf,Pik3ap1,Atf3,Hspb1,Ncf1,Cd14,Cflar,Ripk1,Tnip1,Tgm2,C5ar1,Nfe2l2,Mmp8,Selp,Icam1,Sox11,Errfi1,Thbs1,Gadd45g,P2ry6,Map3k8,Birc3,Pik3r5,Plaur,Tnfaip3,Tnfsf10,Ksr1,Xdh,Ccl2,Irak3,Il1rn,Csf3,Ptpn1,Tnip3,S100a8,Iqgap1,Ccl12,S100a9 | | 2.186e-09 | -19.94 | granulocyte migration | biological process | GO:0097530 | 61 | 11 | 13711 | 214 | C5ar1,Cxcl1,S100a8,Ccl12,S100a9,Ccl4,Il17ra,Cxcl9,Ccl2,Il1rn,Ccl7 | | 2.237e-09 | -19.92 | LENAOUR\_DENDRITIC\_CELL\_MATURATION\_DN | MSigDB lists | LENAOUR\_DENDRITIC\_CELL\_MATURATION\_DN | 100 | 13 | 12187 | 179 | S100a8,S100a9,Irf7,Cxcl1,C3ar1,C5ar1,Cybb,Nfkbia,Tnf,Plaur,Cd14,Isg15,Ier3 | | 2.243e-09 | -19.92 | leukocyte chemotaxis | biological process | GO:0030595 | 94 | 13 | 13711 | 214 | Ccl2,Cxcl9,Retnlg,Ccl7,Il1rn,Cxcl16,Ccl12,S100a8,C5ar1,Cxcl1,Il17ra,Ccl4,S100a9 | | 2.432e-09 | -19.83 | GSE20715\_0H\_VS\_48H\_OZONE\_LUNG\_DN | MSigDB lists | GSE20715\_0H\_VS\_48H\_OZONE\_LUNG\_DN | 165 | 16 | 12187 | 179 | Map3k6,Saa1,Ptgs2,Cd14,Hspb1,Rbm47,Errfi1,Cdkn1a,Hspa5,Socs3,Dnajb1,Timp1,Lcn2,Banp,Rhou,Il6 | | 2.534e-09 | -19.79 | Influenza A | KEGG pathways | mmu05164 | 124 | 16 | 5248 | 107 | Nfkbia,Ccl2,Pik3r1,Il1a,Ccl12,Socs3,Il6,Trim25,Nfkb1,Icam1,Tnf,Tnfsf10,Irf9,Irf7,Dnajb1,Mx2 | | 2.534e-09 | -19.79 | Influenza A | KEGG pathways | ko05164 | 124 | 16 | 5248 | 107 | Tnfsf10,Irf9,Irf7,Icam1,Tnf,Nfkb1,Socs3,Il6,Trim25,Mx2,Dnajb1,Pik3r1,Ccl2,Nfkbia,Ccl12,Il1a | | 2.641e-09 | -19.75 | positive regulation of phosphorylation | biological process | GO:0042327 | 843 | 38 | 13711 | 214 | Cdkn1a,Il6,Il1a,Akap12,Icam1,P2ry6,Lgals9,Gadd45b,Ccnd2,Pik3r1,Gadd45g,Parp14,Ccl7,Thbs1,Parp9,Sdc4,Fzd4,Map3k6,Map3k8,Plaur,Pik3r5,Ptgs2,Ccl4,Ccl2,Xdh,Ksr1,Tnf,Ripk1,Csf3,Cflar,Ncf1,Il1rn,Ccl12,Iqgap1,Ptpn1,C5ar1,Irgm2,Mmp8 | | 2.698e-09 | -19.73 | activation of innate immune response | biological process | GO:0002218 | 78 | 12 | 13711 | 214 | Tnip3,Ifi209,Irgm2,Ifi211,Tnf,Pik3ap1,Nfkbia,Tnip1,Lgals9,Ifi204,Irak3,Cd14 | | 2.698e-09 | -19.73 | negative regulation of viral process | biological process | GO:0048525 | 78 | 12 | 13711 | 214 | Zfp36,Isg15,Tnf,Oasl2,Ifitm2,Trim56,Resf1,Trim25,Ifitm3,Oasl1,Ifitm6,Ccl4 | | 2.713e-09 | -19.73 | GTPase activity | molecular function | GO:0003924 | 253 | 20 | 13516 | 211 | Rhoc,Gbp6,Ifi47,Gm5431,Irgm1,Gbp7,Irgm2,Tubb6,Rhoj,Mx1,Rhou,Mx2,Gbp3,Iigp1,Gem,Tgtp1,F830016B08Rik,Gm4841,Rasd1,Tgtp2 | | 2.901e-09 | -19.66 | GSE45365\_HEALTHY\_VS\_MCMV\_INFECTION\_CD8A\_DC\_IFNAR\_KO\_DN | MSigDB lists | GSE45365\_HEALTHY\_VS\_MCMV\_INFECTION\_CD8A\_DC\_IFNAR\_KO\_DN | 167 | 16 | 12187 | 179 | Casp4,Tnf,Gadd45b,Phf11d,Irf7,Cflar,C3ar1,Phf11b,Ifi44,Cebpd,Il1a,Ifitm2,Plaur,Irf9,Tap1,Rbm47 | | 2.901e-09 | -19.66 | GSE22886\_DAY0\_VS\_DAY1\_MONOCYTE\_IN\_CULTURE\_DN | MSigDB lists | GSE22886\_DAY0\_VS\_DAY1\_MONOCYTE\_IN\_CULTURE\_DN | 167 | 16 | 12187 | 179 | Cxcl1,Nfkbie,Ccl4,Sdc4,Runx1,Birc3,Maff,Ccl12,Il6,Tubb6,Tnip1,Ptgs2,Cdkn1a,Ier3,Il1rn,Gadd45g | | 2.906e-09 | -19.66 | biological regulation | biological process | GO:0065007 | 8325 | 170 | 13711 | 214 | Trim56,Il17ra,Plaur,Bach1,Kcna5,Nfkb1,Adamts1,Gbp4,Osmr,Rbm39,Banp,P2ry6,Thbs1,Ifitm3,Cybb,Errfi1,Mxd4,Sox11,Samd9l,Bcl3,Irgm2,Sgk3,Irf9,Usp18,Tap1,S100a8,A2m,Irf2,Ccl12,Ifi204,Arid5b,Irak3,Pygm,Adamts9,Xdh,Tnfsf10,Ksr1,Plek,Ptgs2,Sgk1,Ier3,Stx11,Ifitm6,Ccl4,Spi1,Parp9,Sbno2,Sdc4,Ptges,Ifi209,Fzd4,Gadd45b,Pik3r1,Dtx3l,Irf7,Socs3,Trib1,Ctla2a,Il6,Cdkn1a,Oasl2,Casp4,Il1a,Gem,Csf2rb,Ifit2,Hp,Mt2,Prg4,Rrp8,Cxcl1,Dnajb1,Fstl1,Tnip1,Rhoc,Ifi207,Slc24a4,Hspb1,Ripk1,Cflar,Cd14,Gpr84,Zfp36,Tnf,Robo4,Pik3r5,Irgm1,Rhoj,Apold1,Nfkbie,Map3k8,Birc3,Gadd45g,Lgals9,Oasl1,Bbs12,Msr1,Iigp1,Rassf4,Icam1,Mt1,Nfkb2,Fyb,Rgs16,Wfdc21,Vcam1,S100a9,Iqgap1,Col4a1,Tnip3,Cxcl16,Nfkbiz,Ptpn1,Ifitm2,Lcn2,Aff1,Il1rn,C3ar1,Zbtb16,Tnfaip8,Csf3,Depp1,Ccl2,Znfx1,Tnfaip3,Cxcl9,Runx1,Trim25,Resf1,Xaf1,Ifi211,Rasip1,Cebpd,Slfn2,Isg15,Tiparp,Timp1,Map3k6,Fam107a,Ccnd2,Hcar2,Parp14,Ccl7,Ip6k2,Slfn3,Il4ra,Nfkbia,Akap12,Angptl4,Msn,Selp,Noct,Nfe2l2,Serpina3f,Mmp8,Rasd1,C5ar1,Maff,Tgm2,Clic4,Hspa5,Cp,Rnf213,Trim30a,Ncf1,Sele,Atf3,Rhou,Acod1,Pik3ap1 | | 2.911e-09 | -19.65 | GSE1432\_CTRL\_VS\_IFNG\_24H\_MICROGLIA\_DN | MSigDB lists | GSE1432\_CTRL\_VS\_IFNG\_24H\_MICROGLIA\_DN | 144 | 15 | 12187 | 179 | Icam1,Plek,Tap1,Ifitm3,Isg15,Phf11b,Ifi44,Ifit3b,Rtp4,Sbno2,Tnfsf10,Phf11d,Irf7,Parp12,Tgm2 | | 2.925e-09 | -19.65 | cellular response to tumor necrosis factor | biological process | GO:0071356 | 96 | 13 | 13711 | 214 | Nfe2l2,Ccl4,Nfkb1,Ccl12,Birc3,Ccl7,Ripk1,Il6,Zfp36,Ccl2,Acod1,Tnf,Nfkbia | | 2.939e-09 | -19.65 | DAUER\_STAT3\_TARGETS\_DN | MSigDB lists | DAUER\_STAT3\_TARGETS\_DN | 38 | 9 | 12187 | 179 | Parp12,Oasl1,Usp18,Ifit3b,Ifi44,Tnfsf10,Irf7,Herc6,Isg15 | | 2.939e-09 | -19.65 | WORSCHECH\_TUMOR\_REJECTION\_UP | MSigDB lists | WORSCHECH\_TUMOR\_REJECTION\_UP | 38 | 9 | 12187 | 179 | Ifi211,Cxcl1,Ifi207,Il1a,Ifi204,Msr1,Ifi209,Nfkbiz,Ccl4 | | 3.089e-09 | -19.60 | GO\_REGULATION\_OF\_RESPONSE\_TO\_EXTERNAL\_STIMULUS | MSigDB lists | GO\_REGULATION\_OF\_RESPONSE\_TO\_EXTERNAL\_STIMULUS | 674 | 32 | 12187 | 179 | Socs3,Plaur,Hspb1,Plek,Sele,Tnip1,Saa1,Ptgs2,Casp4,Trib1,Tnf,Zfp189,Birc3,S100a9,A2m,C5ar1,C3ar1,Nfkb1,Thbs1,Il17ra,Pik3ap1,Selp,Ccl12,Tnfaip3,Il6,Nfe2l2,Ccl4,Ccl2,Sbno2,Osmr,S100a8,Cxcl1 | | 3.129e-09 | -19.58 | GSE34156\_UNTREATED\_VS\_6H\_NOD2\_AND\_TLR1\_TLR2\_LIGAND\_TREATED\_MONOCYTE\_DN | MSigDB lists | GSE34156\_UNTREATED\_VS\_6H\_NOD2\_AND\_TLR1\_TLR2\_LIGAND\_TREATED\_MONOCYTE\_DN | 123 | 14 | 12187 | 179 | Rnf213,Slfn5,Pik3ap1,Tap1,Ifi44,Ifit3b,Samd9l,Irf7,Rtp4,Tnfsf10,Ifit2,Usp18,Xaf1,Parp12 | | 3.129e-09 | -19.58 | GSE41867\_DAY6\_VS\_DAY15\_LCMV\_ARMSTRONG\_EFFECTOR\_CD8\_TCELL\_UP | MSigDB lists | GSE41867\_DAY6\_VS\_DAY15\_LCMV\_ARMSTRONG\_EFFECTOR\_CD8\_TCELL\_UP | 123 | 14 | 12187 | 179 | Rnf213,Ptgs2,Errfi1,Stx11,Nfkb1,Il1a,Samd9l,Gem,Map3k8,Gpr84,Usp18,Ripk1,Il6,Oasl1 | | 3.166e-09 | -19.57 | GSE19401\_PLN\_VS\_PEYERS\_PATCH\_FOLLICULAR\_DC\_DN | MSigDB lists | GSE19401\_PLN\_VS\_PEYERS\_PATCH\_FOLLICULAR\_DC\_DN | 168 | 16 | 12187 | 179 | Ifit3b,Cdkn1a,Ifi44,Stx11,Ifitm3,Plaur,Tap1,Xaf1,Tgm2,Ccl4,Ifit2,Maff,Ccl12,Il6,Oasl1,Cflar | | 3.166e-09 | -19.57 | GSE3337\_CTRL\_VS\_4H\_IFNG\_IN\_CD8POS\_DC\_DN | MSigDB lists | GSE3337\_CTRL\_VS\_4H\_IFNG\_IN\_CD8POS\_DC\_DN | 168 | 16 | 12187 | 179 | Gbp7,Slfn4,Gbp3,Ifit2,Tnf,Casp4,Usp18,Tor3a,Tnfaip3,Slfn3,Tap1,Ifit3b,Gadd45g,Ksr1,Ier3,Znfx1 | | 3.176e-09 | -19.57 | HESS\_TARGETS\_OF\_HOXA9\_AND\_MEIS1\_DN | MSigDB lists | HESS\_TARGETS\_OF\_HOXA9\_AND\_MEIS1\_DN | 67 | 11 | 12187 | 179 | C3ar1,C5ar1,Cxcl1,Plek,Zfp36,Slfn3,Slfn4,Msr1,Mmp8,Cybb,Lcn2 | | 3.205e-09 | -19.56 | GSE9037\_CTRL\_VS\_LPS\_1H\_STIM\_BMDM\_DN | MSigDB lists | GSE9037\_CTRL\_VS\_LPS\_1H\_STIM\_BMDM\_DN | 145 | 15 | 12187 | 179 | Casp4,Ccl4,Parp14,Tgm2,Msr1,Maff,Map3k8,Gadd45b,Rtp4,Rbm39,Slfn4,Vcam1,Tnip1,Errfi1,Slfn3 | | 3.210e-09 | -19.56 | HORIUCHI\_WTAP\_TARGETS\_UP | MSigDB lists | HORIUCHI\_WTAP\_TARGETS\_UP | 245 | 19 | 12187 | 179 | Rnf213,Cxcl16,Sele,Icam1,Apold1,Ifit3b,Vcam1,Ifi44,Atf3,Cebpd,Col4a1,Arid5b,Cxcl1,Osmr,Sdc4,Birc3,Ifit2,Ccl12,Tnfaip3 | | 3.240e-09 | -19.55 | SCHOEN\_NFKB\_SIGNALING | MSigDB lists | SCHOEN\_NFKB\_SIGNALING | 27 | 8 | 12187 | 179 | Il1a,Ccl12,Gadd45b,Thbs1,Birc3,Nfkbia,Ptgs2,Cxcl1 | | 3.240e-09 | -19.55 | GROSS\_ELK3\_TARGETS\_DN | MSigDB lists | GROSS\_ELK3\_TARGETS\_DN | 27 | 8 | 12187 | 179 | Socs3,Adamts1,Ifi44,Maff,Il6,Cxcl1,Ptgs2,Plek | | 3.241e-09 | -19.55 | GSE17974\_IL4\_AND\_ANTI\_IL12\_VS\_UNTREATED\_24H\_ACT\_CD4\_TCELL\_DN | MSigDB lists | GSE17974\_IL4\_AND\_ANTI\_IL12\_VS\_UNTREATED\_24H\_ACT\_CD4\_TCELL\_DN | 103 | 13 | 12187 | 179 | Tnfsf10,Rhou,Ifit2,Usp18,Ccl4,Xaf1,Oasl1,Isg15,Herc6,Ifi44,Ifit3b,Samd9l,Atf3 | | 3.316e-09 | -19.52 | Nfkb1 (nuclear factor of kappa light polypeptide gene enhancer in B cells 1, p105) | protein interactions | 18033 | 16 | 7 | 6802 | 115 | Nfkbiz,Il6,Map3k8,Nfkb2,Nfkb1,Nfkbia,Sele | | 3.482e-09 | -19.48 | BROWN\_MYELOID\_CELL\_DEVELOPMENT\_UP | MSigDB lists | BROWN\_MYELOID\_CELL\_DEVELOPMENT\_UP | 124 | 14 | 12187 | 179 | Slfn3,Plaur,Samd9l,Il1rn,Mmp8,Lcn2,Cebpd,C5ar1,Slfn4,Iqgap1,Xdh,Ccnd2,Tnf,Cybb | | 3.482e-09 | -19.48 | GSE27241\_CTRL\_VS\_DIGOXIN\_TREATED\_CD4\_TCELL\_IN\_TH17\_POLARIZING\_CONDITIONS\_UP | MSigDB lists | GSE27241\_CTRL\_VS\_DIGOXIN\_TREATED\_CD4\_TCELL\_IN\_TH17\_POLARIZING\_CONDITIONS\_UP | 124 | 14 | 12187 | 179 | Gadd45b,Tnfaip3,Trib1,Nfkbia,Parp14,Birc3,Map3k8,Nfkb2,Arid5b,Ier3,Stx11,Nfkb1,Nfkbiz,Icam1 | | 3.499e-09 | -19.47 | regulation of phosphate metabolic process | biological process | GO:0019220 | 1427 | 52 | 13711 | 214 | Pik3r1,Ccnd2,Gadd45b,Socs3,Ccl7,Parp14,Trib1,Il6,Cdkn1a,Akap12,Il1a,Ptgs2,Ier3,Rasip1,Ccl4,Sdc4,Parp9,Map3k6,Fzd4,Tnip1,Hspb1,Ncf1,Cflar,Ripk1,Atf3,Tnf,Pik3ap1,Mmp8,C5ar1,Gadd45g,Lgals9,P2ry6,Thbs1,Errfi1,Icam1,Pik3r5,Plaur,Gbp4,Map3k8,Birc3,Irak3,Il1rn,Csf3,Ksr1,Xdh,Ccl2,Tnfaip3,Plek,Irgm2,Iqgap1,Ccl12,Ptpn1 | | 3.584e-09 | -19.45 | regulation of phosphorus metabolic process | biological process | GO:0051174 | 1428 | 52 | 13711 | 214 | Socs3,Trib1,Parp14,Ccl7,Gadd45b,Ccnd2,Pik3r1,Akap12,Il1a,Il6,Cdkn1a,Rasip1,Ccl4,Ptgs2,Ier3,Map3k6,Fzd4,Parp9,Sdc4,Ncf1,Hspb1,Ripk1,Cflar,Tnip1,Tnf,Pik3ap1,Atf3,Mmp8,C5ar1,Thbs1,Gadd45g,P2ry6,Lgals9,Icam1,Errfi1,Pik3r5,Plaur,Map3k8,Gbp4,Birc3,Irak3,Il1rn,Csf3,Tnfaip3,Plek,Xdh,Ksr1,Ccl2,Irgm2,Ptpn1,Iqgap1,Ccl12 | | 3.657e-09 | -19.43 | TAKEDA\_TARGETS\_OF\_NUP98\_HOXA9\_FUSION\_16D\_UP | MSigDB lists | TAKEDA\_TARGETS\_OF\_NUP98\_HOXA9\_FUSION\_16D\_UP | 104 | 13 | 12187 | 179 | Ptgs2,Isg15,Thbs1,Ifi44,Ifit3b,Gem,Cmpk2,Tnfsf10,Ifit2,Usp18,Xaf1,Oasl1,AA467197 | | 3.763e-09 | -19.40 | GSE15930\_STIM\_VS\_STIM\_AND\_TRICHOSTATINA\_48H\_CD8\_T\_CELL\_DN | MSigDB lists | GSE15930\_STIM\_VS\_STIM\_AND\_TRICHOSTATINA\_48H\_CD8\_T\_CELL\_DN | 170 | 16 | 12187 | 179 | Irf9,Tap1,Slfn3,Trim25,Isg15,Atf3,Znfx1,Nfkb1,Cmpk2,Slfn4,Irf7,Irgm2,Usp18,Lgals9,Ccnd2,Tnf | | 3.878e-09 | -19.37 | GSE12392\_IFNAR\_KO\_VS\_IFNB\_KO\_CD8\_NEG\_SPLEEN\_DC\_DN | MSigDB lists | GSE12392\_IFNAR\_KO\_VS\_IFNB\_KO\_CD8\_NEG\_SPLEEN\_DC\_DN | 147 | 15 | 12187 | 179 | Nfkbia,Il4ra,Il6,Cxcl1,Csf3,Socs3,Nfkbiz,Adamts1,Thbs1,Rasip1,Nfkb1,Mmp8,Tnip1,Errfi1,Plaur | | 3.928e-09 | -19.36 | - | gene3d domains | 3.30.950.30 | 6 | 5 | 6647 | 99 | Slfn5,Slfn3,Slfn2,Slfn4,Slfn9 | | 4.014e-09 | -19.33 | BIOCARTA\_LAIR\_PATHWAY | MSigDB lists | BIOCARTA\_LAIR\_PATHWAY | 11 | 6 | 12187 | 179 | Selp,Tnf,Vcam1,Icam1,Il1a,Il6 | | 4.099e-09 | -19.31 | GSE37416\_0H\_VS\_6H\_F\_TULARENSIS\_LVS\_NEUTROPHIL\_DN | MSigDB lists | GSE37416\_0H\_VS\_6H\_F\_TULARENSIS\_LVS\_NEUTROPHIL\_DN | 171 | 16 | 12187 | 179 | Banp,Pik3r5,Nfkbie,Gpr84,Nfkbia,Tnf,Maff,Il4ra,Plaur,Cxcl16,Icam1,Cdkn1a,Ier3,Tiparp,Nfkb1,Gadd45g | | 4.143e-09 | -19.30 | neutrophil chemotaxis | biological process | GO:0030593 | 50 | 10 | 13711 | 214 | Il1rn,Ccl4,Ccl7,S100a9,C5ar1,Cxcl1,Cxcl9,S100a8,Ccl2,Ccl12 | | 4.216e-09 | -19.28 | positive regulation of phosphorus metabolic process | biological process | GO:0010562 | 896 | 39 | 13711 | 214 | Cdkn1a,Il6,Akap12,Il1a,Icam1,Lgals9,P2ry6,Ccnd2,Pik3r1,Gadd45g,Gadd45b,Ccl7,Parp14,Thbs1,Sdc4,Parp9,Map3k6,Fzd4,Map3k8,Plaur,Ptgs2,Pik3r5,Ccl4,Ccl2,Ksr1,Xdh,Plek,Tnf,Cflar,Ripk1,Csf3,Il1rn,Ncf1,Ccl12,Iqgap1,Ptpn1,C5ar1,Irgm2,Mmp8 | | 4.216e-09 | -19.28 | positive regulation of phosphate metabolic process | biological process | GO:0045937 | 896 | 39 | 13711 | 214 | Plaur,Pik3r5,Ptgs2,Ccl4,Parp9,Sdc4,Map3k6,Fzd4,Map3k8,P2ry6,Lgals9,Gadd45b,Ccnd2,Gadd45g,Pik3r1,Parp14,Ccl7,Thbs1,Cdkn1a,Il6,Il1a,Akap12,Icam1,Irgm2,Mmp8,Ccl12,Iqgap1,Ptpn1,C5ar1,Ripk1,Csf3,Cflar,Ncf1,Il1rn,Ccl2,Xdh,Ksr1,Plek,Tnf | | 4.217e-09 | -19.28 | myeloid leukocyte migration | biological process | GO:0097529 | 81 | 12 | 13711 | 214 | C5ar1,Cxcl1,Ccl12,S100a8,Ccl4,S100a9,Il17ra,Cxcl9,Ccl2,Ccl7,Il1rn,Retnlg | | 4.261e-09 | -19.27 | GSE17721\_LPS\_VS\_POLYIC\_1H\_BMDC\_UP | MSigDB lists | GSE17721\_LPS\_VS\_POLYIC\_1H\_BMDC\_UP | 148 | 15 | 12187 | 179 | Timp1,Tiparp,Il1a,Cebpd,Cdkn1a,Hspa5,Icam1,Slfn3,Zfp36,Errfi1,Gadd45b,Maff,Msr1,Slfn4,Tnfsf10 | | 4.261e-09 | -19.27 | GSE34156\_UNTREATED\_VS\_6H\_NOD2\_LIGAND\_TREATED\_MONOCYTE\_DN | MSigDB lists | GSE34156\_UNTREATED\_VS\_6H\_NOD2\_LIGAND\_TREATED\_MONOCYTE\_DN | 148 | 15 | 12187 | 179 | Phf11d,C3ar1,Tnfsf10,Gbp3,Cmpk2,Usp18,Xaf1,Parp12,Irf2,Ifit3b,Phf11b,Znfx1,Luc7l3,Samd9l,Gadd45g | | 4.300e-09 | -19.26 | GSE7348\_UNSTIM\_VS\_TOLERIZED\_AND\_LPS\_STIM\_MACROPHAGE\_DN | MSigDB lists | GSE7348\_UNSTIM\_VS\_TOLERIZED\_AND\_LPS\_STIM\_MACROPHAGE\_DN | 126 | 14 | 12187 | 179 | Cflar,Arid5b,Ptges,Phf11d,Parp12,Tnfaip3,Map3k8,Casp4,Isg15,Tnip1,Samd9l,Nfkb1,Stx11,Phf11b | | 4.300e-09 | -19.26 | GO\_CELLULAR\_RESPONSE\_TO\_BIOTIC\_STIMULUS | MSigDB lists | GO\_CELLULAR\_RESPONSE\_TO\_BIOTIC\_STIMULUS | 126 | 14 | 12187 | 179 | Sbno2,Cmpk2,Csf3,Il6,Ccl12,Tnfaip3,Nfkbia,Tnf,Cxcl16,Icam1,Cd14,Tnip3,Nfkb1,Hspa5 | | 4.315e-09 | -19.26 | positive regulation of innate immune response | biological process | GO:0045089 | 139 | 15 | 13711 | 214 | Irak3,Irf7,Cd14,Tnip1,Ifi204,Lgals9,Tnf,Nfkbia,Pik3ap1,Irgm1,Ifi211,Irgm2,Ifi209,Tnip3,Parp9 | | 4.394e-09 | -19.24 | GO\_POSITIVE\_REGULATION\_OF\_INFLAMMATORY\_RESPONSE | MSigDB lists | GO\_POSITIVE\_REGULATION\_OF\_INFLAMMATORY\_RESPONSE | 69 | 11 | 12187 | 179 | Ccl2,Ccl4,Tnf,Il6,Ccl12,S100a9,Tnip1,Ptgs2,Osmr,S100a8,Il17ra | | 4.394e-09 | -19.24 | GO\_REGULATION\_OF\_TUMOR\_NECROSIS\_FACTOR\_SUPERFAMILY\_CYTOKINE\_PRODUCTION | MSigDB lists | GO\_REGULATION\_OF\_TUMOR\_NECROSIS\_FACTOR\_SUPERFAMILY\_CYTOKINE\_PRODUCTION | 69 | 11 | 12187 | 179 | Zfp36,Irak3,Hspb1,Errfi1,Cd14,Bcl3,Tnfaip3,Ripk1,Ccl12,Thbs1,Pik3r1 | | 4.460e-09 | -19.23 | regulation of molecular function | biological process | GO:0065009 | 2276 | 70 | 13711 | 214 | Oasl2,Casp4,Nfkbia,Gem,Il6,Cdkn1a,Dtx3l,Trib1,Ccl7,Gadd45b,Pik3r1,Ccnd2,Timp1,Fzd4,Map3k6,Parp9,Sdc4,Rasip1,Ccl4,Ptgs2,Trim25,Tnf,Acod1,Zfp36,Ncf1,Sele,Hspb1,Ripk1,Cflar,Rhoc,Dnajb1,C5ar1,Hp,Serpina3f,Mmp8,Angptl4,Ifit2,Icam1,Rgs16,Sox11,Errfi1,Thbs1,Oasl1,Gadd45g,P2ry6,Lgals9,Map3k8,Birc3,Nfkb1,Pik3r5,Plaur,Tnfaip3,Plek,Xdh,Tnfsf10,Ksr1,Ccl2,Irak3,Arid5b,Il1rn,Tnfaip8,Csf3,Ptpn1,S100a8,Iqgap1,A2m,Ccl12,S100a9,Irgm2,Wfdc21 | | 4.480e-09 | -19.22 | BOYAULT\_LIVER\_CANCER\_SUBCLASS\_G5\_DN | MSigDB lists | BOYAULT\_LIVER\_CANCER\_SUBCLASS\_G5\_DN | 28 | 8 | 12187 | 179 | Bcl3,Ifi211,Icam1,Ifi44,Ifi209,Tgm2,Ifi207,Ifi204 | | 4.536e-09 | -19.21 | QI\_PLASMACYTOMA\_UP | MSigDB lists | QI\_PLASMACYTOMA\_UP | 197 | 17 | 12187 | 179 | Rgs16,Ptges,Irgm2,Nfkbie,Tnfaip3,Rassf4,Ccl4,Tgm2,Casp4,Nfkbia,Tnf,Il4ra,Ptpn1,Irf2,Plek,Dnajb1,Sox11 | | 4.678e-09 | -19.18 | GSE2405\_S\_AUREUS\_VS\_A\_PHAGOCYTOPHILUM\_NEUTROPHIL\_UP | MSigDB lists | GSE2405\_S\_AUREUS\_VS\_A\_PHAGOCYTOPHILUM\_NEUTROPHIL\_UP | 149 | 15 | 12187 | 179 | Bcl3,Gbp3,Adamts9,Xaf1,Tor3a,Oasl1,Slfn9,Ifitm3,Parp9,Csf2rb,Trim25,Hspb1,Plek,Timp1,Nfkb1 | | 4.778e-09 | -19.16 | SENESE\_HDAC1\_TARGETS\_UP | MSigDB lists | SENESE\_HDAC1\_TARGETS\_UP | 370 | 23 | 12187 | 179 | Gem,Robo4,Nfkb2,Birc3,Nfkbia,Trib1,Bach1,Errfi1,Ifi209,Ifi207,Ifi204,Ifi211,Osmr,Iqgap1,Maff,Ccnd2,Tgm2,Nfe2l2,Tnfaip3,Ccl12,Nfkbiz,Il1a,Cebpd | | 4.808e-09 | -19.15 | GO\_POSITIVE\_REGULATION\_OF\_MOLECULAR\_FUNCTION | MSigDB lists | GO\_POSITIVE\_REGULATION\_OF\_MOLECULAR\_FUNCTION | 1413 | 49 | 12187 | 179 | Ptpn1,Map3k6,Sgk1,Hspa5,Rgs16,S100a8,Ifi211,Arid5b,Ripk1,Gadd45b,Ccl2,Ccnd2,Trim25,Icam1,Plek,Saa1,Ifi204,Ifi209,Cdkn1a,Tnfsf10,S100a9,Fzd4,Cflar,Irak3,Sgk3,Nfkb1,Gadd45g,Dnajb1,Thbs1,Iqgap1,Pik3r5,Csf3,Ccl12,Il6,Rhoc,Ccl4,Map3k8,Plaur,Errfi1,Csf2rb,Sele,Ifi207,Nfkb2,Xdh,A2m,C5ar1,Sdc4,Nfkbia,Tnf | | 4.859e-09 | -19.14 | Schlafen | interpro domains | IPR029684 | 6 | 5 | 13788 | 212 | Slfn3,Slfn5,Slfn9,Slfn4,Slfn2 | | 4.859e-09 | -19.14 | Schlafen\_AlbA\_2\_dom | interpro domains | IPR007421 | 6 | 5 | 13788 | 212 | Slfn9,Slfn3,Slfn5,Slfn2,Slfn4 | | 4.859e-09 | -19.14 | Schlafen\_AlbA\_2\_dom\_sf | interpro domains | IPR038461 | 6 | 5 | 13788 | 212 | Slfn3,Slfn5,Slfn9,Slfn4,Slfn2 | | 4.908e-09 | -19.13 | ONDER\_CDH1\_TARGETS\_2\_DN | MSigDB lists | ONDER\_CDH1\_TARGETS\_2\_DN | 309 | 21 | 12187 | 179 | Parp12,Tnfaip3,Il4ra,Birc3,Ccnd2,Sdc4,S100a8,Tnfsf10,Xdh,Csf3,S100a9,Cxcl1,Ptges,Il1a,Il1rn,Ier3,Lcn2,Rbm47,Icam1,Ptgs2,Herc6 | | 5.115e-09 | -19.09 | leukocyte cell-cell adhesion | biological process | GO:0007159 | 38 | 9 | 13711 | 214 | S100a8,Icam1,Tnf,Msn,Tnip1,Selp,Vcam1,Sele,S100a9 | | 5.119e-09 | -19.09 | LEI\_MYB\_TARGETS | MSigDB lists | LEI\_MYB\_TARGETS | 252 | 19 | 12187 | 179 | S100a9,Arid5b,Cflar,S100a8,Tgm2,Ccnd2,Ccl12,Il6,Tnip1,Ifitm2,Saa1,Isg15,Clic4,Msn,Hspb1,Dnajb1,Cdkn1a,Lcn2,Il1rn | | 5.217e-09 | -19.07 | AlbA\_2 | pfam domains | PF04326 | 6 | 5 | 12881 | 201 | Slfn2,Slfn9,Slfn5,Slfn3,Slfn4 | | 6.014e-09 | -18.93 | HUMMERICH\_SKIN\_CANCER\_PROGRESSION\_UP | MSigDB lists | HUMMERICH\_SKIN\_CANCER\_PROGRESSION\_UP | 71 | 11 | 12187 | 179 | Il4ra,Rhoc,Gadd45b,Ifitm3,Saa1,Il17ra,Cdkn1a,Hspa5,Vcam1,Il1rn,Lcn2 | | 6.109e-09 | -18.91 | KRASNOSELSKAYA\_ILF3\_TARGETS\_UP | MSigDB lists | KRASNOSELSKAYA\_ILF3\_TARGETS\_UP | 29 | 8 | 12187 | 179 | Tap1,Ifi207,Ifi204,Ifit3b,Ifi211,Xaf1,Isg15,Ifi209 | | 6.483e-09 | -18.85 | KEGG\_CYTOKINE\_CYTOKINE\_RECEPTOR\_INTERACTION | MSigDB lists | KEGG\_CYTOKINE\_CYTOKINE\_RECEPTOR\_INTERACTION | 130 | 14 | 12187 | 179 | Osmr,Il17ra,Tnfsf10,Cxcl16,Csf2rb,Csf3,Cxcl1,Il1a,Il6,Ccl12,Il4ra,Tnf,Ccl2,Ccl4 | | 7.009e-09 | -18.78 | MODULE\_208 | MSigDB lists | MODULE\_208 | 72 | 11 | 12187 | 179 | Ifitm3,Selp,Csf3,Ifitm2,Isg15,Cd14,Tnfsf10,Ifi44,Thbs1,Vcam1,Cebpd | | 7.915e-09 | -18.65 | GSE2706\_2H\_VS\_8H\_R848\_STIM\_DC\_DN | MSigDB lists | GSE2706\_2H\_VS\_8H\_R848\_STIM\_DC\_DN | 132 | 14 | 12187 | 179 | Parp9,Ifi204,Tnip3,Samd9l,Ifi207,Ifi209,Thbs1,Ifi44,Tnfsf10,Cmpk2,Ifi211,Dtx3l,Parp14,Xaf1 | | 8.018e-09 | -18.64 | MISSIAGLIA\_REGULATED\_BY\_METHYLATION\_UP | MSigDB lists | MISSIAGLIA\_REGULATED\_BY\_METHYLATION\_UP | 91 | 12 | 12187 | 179 | Isg15,Ptgs2,Plaur,Tap1,Sdc4,Cdkn1a,Birc3,Mxd4,Tnfaip3,Atf3,Lcn2,Timp1 | | 8.079e-09 | -18.63 | SENESE\_HDAC3\_TARGETS\_UP | MSigDB lists | SENESE\_HDAC3\_TARGETS\_UP | 413 | 24 | 12187 | 179 | Slfn5,Nfkbiz,Rgs16,Iqgap1,Ifi211,Ccl12,Tnfaip3,Rhoc,Ifit2,Ccnd2,Errfi1,Atf3,Ifi207,Ifi204,Ifit3b,Ifi209,Nfkb2,Robo4,Gem,Oasl1,Bach1,Trib1,Nfkbia,Birc3 | | 8.213e-09 | -18.62 | GSE7218\_UNSTIM\_VS\_ANTIGEN\_STIM\_THROUGH\_IGG\_BCELL\_DN | MSigDB lists | GSE7218\_UNSTIM\_VS\_ANTIGEN\_STIM\_THROUGH\_IGG\_BCELL\_DN | 111 | 13 | 12187 | 179 | Casp4,Tnf,Gadd45b,S100a9,Nfkbie,Gem,Rgs16,Ifi44,Atf3,Lcn2,Gadd45g,Il1rn,Irak3 | | 8.227e-09 | -18.62 | MODULE\_108 | MSigDB lists | MODULE\_108 | 30 | 8 | 12187 | 179 | Ccl2,Ccl4,Ccl12,Il1a,Cxcl1,C3ar1,C5ar1,Plaur | | 8.515e-09 | -18.58 | cell chemotaxis | biological process | GO:0060326 | 146 | 15 | 13711 | 214 | Ccl12,Cxcl16,S100a8,Cxcl1,C5ar1,Il17ra,Ccl4,S100a9,Vcam1,Ccl2,Saa1,Cxcl9,Retnlg,Ccl7,Il1rn | | 8.811e-09 | -18.55 | LTE2\_UP.V1\_DN | MSigDB lists | LTE2\_UP.V1\_DN | 156 | 15 | 12187 | 179 | Atf3,Ifi44,Phf11b,Ifit3b,Irf9,Tap1,Isg15,Herc6,Oasl1,Parp12,Usp18,Gem,Irf7,Ptges,Phf11d | | 9.141e-09 | -18.51 | neutrophil migration | biological process | GO:1990266 | 54 | 10 | 13711 | 214 | Il1rn,S100a9,Ccl4,Ccl7,S100a8,Ccl12,Ccl2,C5ar1,Cxcl1,Cxcl9 | | 9.546e-09 | -18.47 | GO\_ACUTE\_INFLAMMATORY\_RESPONSE | MSigDB lists | GO\_ACUTE\_INFLAMMATORY\_RESPONSE | 43 | 9 | 12187 | 179 | Saa1,Ptges,S100a8,Icam1,Vcam1,Il1a,Hp,Il1rn,Il6 | | 1.005e-08 | -18.42 | defense response to bacterium | biological process | GO:0042742 | 106 | 13 | 13711 | 214 | Hp,Isg15,C5ar1,Gbp3,Gbp7,Bcl3,Gbp6,Irgm2,Tnf,Lcn2,Ncf1,Tnfaip8,Iigp1 | | 1.032e-08 | -18.39 | GO\_CYTOKINE\_ACTIVITY | MSigDB lists | GO\_CYTOKINE\_ACTIVITY | 93 | 12 | 12187 | 179 | Cxcl1,Csf3,Cxcl16,Tnfsf10,Ccl4,Ccl2,Tnf,Ccl12,Il6,Timp1,Il1rn,Il1a | | 1.093e-08 | -18.33 | KEGG\_TOLL\_LIKE\_RECEPTOR\_SIGNALING\_PATHWAY | MSigDB lists | KEGG\_TOLL\_LIKE\_RECEPTOR\_SIGNALING\_PATHWAY | 75 | 11 | 12187 | 179 | Nfkb1,Cd14,Pik3r1,Map3k8,Tnf,Nfkbia,Ccl4,Il6,Ripk1,Irf7,Pik3r5 | | 1.095e-08 | -18.33 | KIM\_LRRC3B\_TARGETS | MSigDB lists | KIM\_LRRC3B\_TARGETS | 31 | 8 | 12187 | 179 | Ifi204,Ifi207,Ifi209,Ifi44,Tap1,Isg15,Ifitm2,Ifi211 | | 1.095e-08 | -18.33 | HAN\_JNK\_SINGALING\_UP | MSigDB lists | HAN\_JNK\_SINGALING\_UP | 31 | 8 | 12187 | 179 | Isg15,Gbp3,Irf9,Ccnd2,Runx1,Sgk1,Ifit3b,Usp18 | | 1.144e-08 | -18.29 | GSE7219\_WT\_VS\_NIK\_NFKB2\_KO\_DC\_UP | MSigDB lists | GSE7219\_WT\_VS\_NIK\_NFKB2\_KO\_DC\_UP | 159 | 15 | 12187 | 179 | Rnf213,Clic4,Pik3ap1,Zfp36,Dnajb1,Socs3,Ifi44,Atf3,Irf7,Gem,Nfkbia,Usp18,Ccl4,Tnf,Gadd45b | | 1.225e-08 | -18.22 | GHANDHI\_BYSTANDER\_IRRADIATION\_UP | MSigDB lists | GHANDHI\_BYSTANDER\_IRRADIATION\_UP | 59 | 10 | 12187 | 179 | Ptges,Cxcl1,Ptgs2,Irak3,Icam1,Nfkbiz,Birc3,Tnfaip3,Gadd45g,Il1a | | 1.238e-08 | -18.21 | TLR4 (Toll-like receptor 4) signaling pathway | Lipid Maps pathways | TLR4 | 61 | 17 | 354 | 26 | Icam1,Ptgs2,Tnfaip3,Nfkb2,Ripk1,Nfkb1,Nfkbie,Map3k8,Ccl4,Vcam1,Irf7,Cd14,Il6,Nfkbia,Tnf,Il1a,Irak3 | | 1.246e-08 | -18.20 | GSE25088\_ROSIGLITAZONE\_VS\_IL4\_AND\_ROSIGLITAZONE\_STIM\_STAT6\_KO\_MACROPHAGE\_DAY10\_DN | MSigDB lists | GSE25088\_ROSIGLITAZONE\_VS\_IL4\_AND\_ROSIGLITAZONE\_STIM\_STAT6\_KO\_MACROPHAGE\_DAY10\_DN | 160 | 15 | 12187 | 179 | Ier3,Il1rn,Il1a,Ifit3b,Hspa5,Plaur,Cxcl16,Tap1,Pik3ap1,Parp12,Gadd45b,Parp14,Tgm2,Nfkbia,Dtx3l | | 1.270e-08 | -18.18 | BROCKE\_APOPTOSIS\_REVERSED\_BY\_IL6 | MSigDB lists | BROCKE\_APOPTOSIS\_REVERSED\_BY\_IL6 | 115 | 13 | 12187 | 179 | Bcl3,Irf9,Rgs16,Tnfsf10,Sbno2,Zfp36,Icam1,Socs3,Sgk1,Maff,Map3k8,Gadd45b,Ccl12 | | 1.279e-08 | -18.17 | GO\_POSITIVE\_REGULATION\_OF\_MULTICELLULAR\_ORGANISMAL\_PROCESS | MSigDB lists | GO\_POSITIVE\_REGULATION\_OF\_MULTICELLULAR\_ORGANISMAL\_PROCESS | 1046 | 40 | 12187 | 179 | Saa1,Ptgs2,Icam1,Hspb1,Plek,Adamts1,Ifi209,Zbtb16,Ifi204,Ifi207,C5ar1,C3ar1,S100a9,Irf7,Nfkb2,Runx1,Tnf,Trib1,Selp,Isg15,Cd14,Irak3,Il17ra,Sox11,Thbs1,Sgk1,Nfkb1,Il1a,Csf3,Ifi211,Angptl4,Bcl3,Pik3r1,Il4ra,Hcar2,Tnfaip3,Ripk1,Ccl12,Nfe2l2,Il6 | | 1.357e-08 | -18.12 | GSE17721\_CTRL\_VS\_GARDIQUIMOD\_12H\_BMDC\_DN | MSigDB lists | GSE17721\_CTRL\_VS\_GARDIQUIMOD\_12H\_BMDC\_DN | 161 | 15 | 12187 | 179 | Slfn4,Ccl12,Ifit2,Maff,Parp14,Nfkbia,Ccl2,Casp4,Slfn3,Map3k6,Tagln2,Timp1,Nfkb1,Tiparp,Cdkn1a | | 1.357e-08 | -18.12 | GSE37533\_UNTREATED\_VS\_PIOGLIZATONE\_TREATED\_CD4\_TCELL\_PPARG1\_AND\_FOXP3\_TRASDUCED\_UP | MSigDB lists | GSE37533\_UNTREATED\_VS\_PIOGLIZATONE\_TREATED\_CD4\_TCELL\_PPARG1\_AND\_FOXP3\_TRASDUCED\_UP | 161 | 15 | 12187 | 179 | Nfkb2,Igsf6,Tnfaip8,Ccl4,Ccl2,Sdc4,Maff,Il6,Tubb6,Tnip1,Clic4,Plaur,Tnip3,Il1rn,Il1a | | 1.357e-08 | -18.12 | GO\_RESPONSE\_TO\_TUMOR\_NECROSIS\_FACTOR | MSigDB lists | GO\_RESPONSE\_TO\_TUMOR\_NECROSIS\_FACTOR | 161 | 15 | 12187 | 179 | Vcam1,Nfkb1,Ptgs2,Cd14,Icam1,Sele,Cxcl16,Tnf,Birc3,Ccl4,Ccl2,Ripk1,Ccl12,Il6,Nfe2l2 | | 1.452e-08 | -18.05 | GO\_REGULATION\_OF\_VIRAL\_GENOME\_REPLICATION | MSigDB lists | GO\_REGULATION\_OF\_VIRAL\_GENOME\_REPLICATION | 60 | 10 | 12187 | 179 | Tnip1,Ifi211,Ifitm3,Isg15,Ifitm2,Oasl1,Ifi204,Ifi207,Ifi209,Tnf | | 1.455e-08 | -18.05 | HINATA\_NFKB\_IMMU\_INF | MSigDB lists | HINATA\_NFKB\_IMMU\_INF | 13 | 6 | 12187 | 179 | Cxcl1,Icam1,Nfkbia,Ccl12,Il6,Nfkb1 | | 1.455e-08 | -18.05 | MODULE\_537 | MSigDB lists | MODULE\_537 | 13 | 6 | 12187 | 179 | Birc3,Cflar,Il1a,Nfkb1,Ier3,Tnfaip3 | | 1.476e-08 | -18.03 | GSE22140\_GERMFREE\_VS\_SPF\_ARTHRITIC\_MOUSE\_CD4\_TCELL\_UP | MSigDB lists | GSE22140\_GERMFREE\_VS\_SPF\_ARTHRITIC\_MOUSE\_CD4\_TCELL\_UP | 162 | 15 | 12187 | 179 | Tnfsf10,Olfml2b,Irf7,Il6,Oasl1,Gadd45b,Xaf1,Ifit2,Irf9,Plek,Isg15,Ifitm2,Ifit3b,Ifi44,Thbs1 | | 1.549e-08 | -17.98 | GSE6269\_FLU\_VS\_E\_COLI\_INF\_PBMC\_UP | MSigDB lists | GSE6269\_FLU\_VS\_E\_COLI\_INF\_PBMC\_UP | 139 | 14 | 12187 | 179 | Herc6,Isg15,Ifitm2,Ifitm3,Ifi207,Ifi204,Ifi44,Ifi209,Irf7,Ifi211,Parp12,Oasl1,Usp18,Xaf1 | | 1.549e-08 | -17.98 | GSE30971\_CTRL\_VS\_LPS\_STIM\_MACROPHAGE\_WBP7\_KO\_2H\_UP | MSigDB lists | GSE30971\_CTRL\_VS\_LPS\_STIM\_MACROPHAGE\_WBP7\_KO\_2H\_UP | 139 | 14 | 12187 | 179 | Clic4,Irak3,Ptgs2,Ier3,Nfkb1,Tnip3,Il1a,Socs3,Rgs16,Cxcl1,Gadd45b,Il6,Nfkbia,Tnf | | 1.699e-08 | -17.89 | GSE24634\_IL4\_VS\_CTRL\_TREATED\_NAIVE\_CD4\_TCELL\_DAY3\_DN | MSigDB lists | GSE24634\_IL4\_VS\_CTRL\_TREATED\_NAIVE\_CD4\_TCELL\_DAY3\_DN | 140 | 14 | 12187 | 179 | Thbs1,Ifit3b,Cybb,Il1a,Ccl12,Parp12,Ifitm3,Cflar,Isg15,Ifitm2,Irf7,P2ry6,Tap1,Tnfsf10 | | 1.699e-08 | -17.89 | GSE34205\_HEALTHY\_VS\_FLU\_INF\_INFANT\_PBMC\_DN | MSigDB lists | GSE34205\_HEALTHY\_VS\_FLU\_INF\_INFANT\_PBMC\_DN | 140 | 14 | 12187 | 179 | S100a9,Irf7,S100a8,Cmpk2,Usp18,Xaf1,Parp12,Oasl1,Ifitm2,Herc6,Isg15,Ifitm3,Ifi44,Mmp8 | | 1.699e-08 | -17.89 | GSE37605\_TREG\_VS\_TCONV\_NOD\_FOXP3\_FUSION\_GFP\_DN | MSigDB lists | GSE37605\_TREG\_VS\_TCONV\_NOD\_FOXP3\_FUSION\_GFP\_DN | 140 | 14 | 12187 | 179 | Sdc4,Ccl12,Gadd45b,Cxcl1,Cflar,Adamts9,Nfkbiz,Adamts1,Thbs1,Atf3,Ier3,Il1rn,Akap12,Selp | | 1.740e-08 | -17.87 | TSAI\_RESPONSE\_TO\_IONIZING\_RADIATION | MSigDB lists | TSAI\_RESPONSE\_TO\_IONIZING\_RADIATION | 118 | 13 | 12187 | 179 | Nfkbie,Cflar,Irf9,Tnfsf10,Nfkb2,Tnfaip8,Nfkbia,Ccl4,Birc3,Sgk1,Cdkn1a,Tnfaip3,Nfkb1 | | 1.743e-08 | -17.86 | regulation of apoptotic process | biological process | GO:0042981 | 1233 | 46 | 13711 | 214 | Ccl12,S100a8,Ptpn1,Bcl3,S100a9,Sgk3,Tnfsf10,Xdh,Tnfaip3,Zbtb16,Tnfaip8,Irak3,Il1rn,Nfkb1,Birc3,Plaur,Mt1,Icam1,Gadd45g,Thbs1,Hspa5,Tgm2,C5ar1,Ifit2,Angptl4,Nfe2l2,Zfp36,Atf3,Tnf,Cflar,Ripk1,Hspb1,Timp1,Ier3,Sgk1,Ptgs2,Cdkn1a,Ip6k2,Il6,Akap12,Casp4,Ccnd2,Hcar2,Pik3r1,Gadd45b,Socs3 | | 1.770e-08 | -17.85 | negative regulation of protein metabolic process | biological process | GO:0051248 | 904 | 38 | 13711 | 214 | Tnf,Tnfaip3,Zfp36,Atf3,Xdh,Hspb1,Irak3,Cflar,Tnfaip8,Tnip1,Ptpn1,Prg4,A2m,Serpina3f,Bcl3,Wfdc21,Errfi1,Il6,Cdkn1a,Socs3,Dtx3l,Thbs1,Parp14,Trib1,Ctla2a,Gadd45g,Pik3r1,Banp,Gadd45b,Timp1,Gbp4,Birc3,Nfkb1,Isg15,Rasip1,Spi1,Ptgs2,Plaur | | 1.863e-08 | -17.80 | GSE1432\_CTRL\_VS\_IFNG\_1H\_MICROGLIA\_DN | MSigDB lists | GSE1432\_CTRL\_VS\_IFNG\_1H\_MICROGLIA\_DN | 141 | 14 | 12187 | 179 | Plek,Zfp36,Cdkn1a,Ifit3b,Socs3,Gadd45g,Angptl4,Rgs16,Pygm,Ifit2,Trib1,Bach1,Gadd45b,Ccl12 | | 1.863e-08 | -17.80 | GSE36888\_UNTREATED\_VS\_IL2\_TREATED\_TCELL\_17H\_DN | MSigDB lists | GSE36888\_UNTREATED\_VS\_IL2\_TREATED\_TCELL\_17H\_DN | 141 | 14 | 12187 | 179 | Ccl4,Il6,Tnfaip3,AA467197,Ptges,Cxcl1,Nfkb2,Nfkbiz,Tnip3,Tnip1,Ptgs2,Plaur,Pik3ap1,Icam1 | | 1.917e-08 | -17.77 | CHEN\_LVAD\_SUPPORT\_OF\_FAILING\_HEART\_UP | MSigDB lists | CHEN\_LVAD\_SUPPORT\_OF\_FAILING\_HEART\_UP | 79 | 11 | 12187 | 179 | Gadd45b,Pik3r1,Zfp189,Nfkbia,Zfp36,Gadd45g,Tiparp,Cebpd,Atf3,Cdkn1a,Zbtb16 | | 2.018e-08 | -17.72 | CASTELLANO\_NRAS\_TARGETS\_UP | MSigDB lists | CASTELLANO\_NRAS\_TARGETS\_UP | 62 | 10 | 12187 | 179 | Usp18,Ifi209,Lcn2,Ifi207,Il1rn,Ifi204,Ifi211,Isg15,Irgm2,Tap1 | | 2.024e-08 | -17.72 | GO\_IMMUNE\_EFFECTOR\_PROCESS | MSigDB lists | GO\_IMMUNE\_EFFECTOR\_PROCESS | 304 | 20 | 12187 | 179 | Ifi207,Ifi204,Ifit3b,Ifi209,Irf9,Icam1,Trim25,Ifitm2,Isg15,Ifitm3,Il6,Oasl1,Il4ra,Pik3r1,Ifit2,Sbno2,Irf7,Bcl3,Trim56,Ifi211 | | 2.040e-08 | -17.71 | GSE9960\_HEALTHY\_VS\_GRAM\_POS\_SEPSIS\_PBMC\_DN | MSigDB lists | GSE9960\_HEALTHY\_VS\_GRAM\_POS\_SEPSIS\_PBMC\_DN | 142 | 14 | 12187 | 179 | Ifi44,Samd9l,Ifitm3,Isg15,Herc6,Hspb1,Ip6k2,Ifit2,Il4ra,Birc3,AA467197,Tnfaip3,Irf7,Nfkb2 | | 2.075e-08 | -17.69 | Cytokine-cytokine receptor interaction | KEGG pathways | ko04060 | 143 | 16 | 5248 | 107 | Tnf,Cxcl9,Tnfsf10,Il6,Csf3,Cxcl16,Ccl7,Ccl2,Il4ra,Osmr,Il17ra,Il1a,Ccl4,Csf2rb,Cxcl1,Ccl12 | | 2.075e-08 | -17.69 | Cytokine-cytokine receptor interaction | KEGG pathways | mmu04060 | 143 | 16 | 5248 | 107 | Ccl4,Il1a,Csf2rb,Cxcl1,Ccl12,Ccl7,Ccl2,Osmr,Il4ra,Il17ra,Csf3,Cxcl16,Tnf,Cxcl9,Tnfsf10,Il6 | | 2.144e-08 | -17.66 | MODULE\_64 | MSigDB lists | MODULE\_64 | 336 | 21 | 12187 | 179 | Rgs16,Tnfsf10,Igsf6,Cxcl1,S100a9,C5ar1,Csf3,C3ar1,Ccl12,Il6,Ccl4,Ccl2,Map3k8,Il4ra,Csf2rb,Plaur,Icam1,Cd14,Akap12,Il1a,Cdkn1a | | 2.214e-08 | -17.63 | regulation of cell communication | biological process | GO:0010646 | 2617 | 75 | 13711 | 214 | Plaur,Pik3r5,Irgm1,Nfkb1,Birc3,Map3k8,Lgals9,P2ry6,Gadd45g,Thbs1,Errfi1,Rgs16,Sox11,Icam1,Irgm2,Bcl3,S100a9,Sgk3,Ccl12,Tnip3,Iqgap1,S100a8,Ptpn1,Csf3,Irak3,Il1rn,Ccl2,Ksr1,Tnfsf10,Xdh,Plek,Tnfaip3,Ier3,Trim25,Ptgs2,Runx1,Ccl4,Rasip1,Parp9,Fam107a,Fzd4,Map3k6,Timp1,Hcar2,Pik3r1,Gadd45b,Ccl7,Trib1,Parp14,Socs3,Irf7,Il6,Il1a,Akap12,Nfkbia,Casp4,Selp,Mmp8,Nfe2l2,Hspa5,Tgm2,C5ar1,Rnf213,Trim30a,Tnip1,Fstl1,Cd14,Cflar,Ripk1,Hspb1,Ncf1,Atf3,Pik3ap1,Acod1,Tnf | | 2.233e-08 | -17.62 | HALLMARK\_ALLOGRAFT\_REJECTION | MSigDB lists | HALLMARK\_ALLOGRAFT\_REJECTION | 143 | 14 | 12187 | 179 | Irf7,Bcl3,Igsf6,Tnf,Ccnd2,Il4ra,Ccl4,Ccl2,Spi1,Ccl12,Il6,Icam1,Tap1,Timp1 | | 2.362e-08 | -17.56 | GSE18791\_CTRL\_VS\_NEWCASTLE\_VIRUS\_DC\_14H\_DN | MSigDB lists | GSE18791\_CTRL\_VS\_NEWCASTLE\_VIRUS\_DC\_14H\_DN | 121 | 13 | 12187 | 179 | Ifit2,Tnf,Parp14,Xaf1,Tnfaip3,Irf7,Cmpk2,Gbp3,Ifi44,Ifit3b,Herc6,Isg15,Trim25 | | 2.380e-08 | -17.55 | GO\_REGULATION\_OF\_TYPE\_I\_INTERFERON\_PRODUCTION | MSigDB lists | GO\_REGULATION\_OF\_TYPE\_I\_INTERFERON\_PRODUCTION | 100 | 12 | 12187 | 179 | Tnfaip3,Nfkb2,Ifi211,Trim56,Irf7,Nfkb1,Ifi204,Ifi207,Ifi209,Trim25,Isg15,Cd14 | | 2.419e-08 | -17.54 | GSE23502\_WT\_VS\_HDC\_KO\_MYELOID\_DERIVED\_SUPPRESSOR\_CELL\_BM\_DN | MSigDB lists | GSE23502\_WT\_VS\_HDC\_KO\_MYELOID\_DERIVED\_SUPPRESSOR\_CELL\_BM\_DN | 168 | 15 | 12187 | 179 | Ptpn1,Csf2rb,Irf9,Rbm47,Ptgs2,Tiparp,Il1rn,Sbno2,Bcl3,C5ar1,Gem,Dtx3l,Ripk1,Rassf4,Tgm2 | | 2.419e-08 | -17.54 | GSE24726\_WT\_VS\_E2\_2\_KO\_PDC\_DN | MSigDB lists | GSE24726\_WT\_VS\_E2\_2\_KO\_PDC\_DN | 168 | 15 | 12187 | 179 | Parp9,Plek,Tagln2,Isg15,Cebpd,Dnajb1,Nfkb2,Pik3r5,Irf7,Bcl3,Cflar,Nfe2l2,Cybb,Hcar2,Usp18 | | 2.419e-08 | -17.54 | GSE17721\_12H\_VS\_24H\_LPS\_BMDC\_UP | MSigDB lists | GSE17721\_12H\_VS\_24H\_LPS\_BMDC\_UP | 168 | 15 | 12187 | 179 | Ifit3b,Atf3,Isg15,Sgk3,P2ry6,Plek,Casp4,Gpr84,Nfkbia,Tor3a,Birc3,Il6,Parp12,Gbp4,Gbp6 | | 2.515e-08 | -17.50 | GILMORE\_CORE\_NFKB\_PATHWAY | MSigDB lists | GILMORE\_CORE\_NFKB\_PATHWAY | 14 | 6 | 12187 | 179 | Nfkb1,Nfkbia,Nfkbiz,Nfkb2,Bcl3,Nfkbie | | 2.636e-08 | -17.45 | PID\_IL23\_PATHWAY | MSigDB lists | PID\_IL23\_PATHWAY | 23 | 7 | 12187 | 179 | Pik3r1,Tnf,Nfkbia,Socs3,Nfkb1,Il6,Ccl12 | | 2.666e-08 | -17.44 | KEGG\_NOD\_LIKE\_RECEPTOR\_SIGNALING\_PATHWAY | MSigDB lists | KEGG\_NOD\_LIKE\_RECEPTOR\_SIGNALING\_PATHWAY | 48 | 9 | 12187 | 179 | Cxcl1,Ccl2,Nfkbia,Tnf,Birc3,Ccl12,Tnfaip3,Il6,Nfkb1 | | 2.706e-08 | -17.43 | NFKB\_Q6 | MSigDB lists | NFKB\_Q6 | 195 | 16 | 12187 | 179 | Luc7l3,Il1rn,Vcam1,Ip6k2,Icam1,Tnip1,Ptgs2,Cybb,Nfkbia,Runx1,Map3k8,Nfkb2,Rrp8,Bcl3,Cxcl1,Ptges | | 2.734e-08 | -17.42 | cytokine receptor binding | molecular function | GO:0005126 | 183 | 16 | 13516 | 211 | Tnf,Ccl12,Ccl7,Il1a,Cflar,Csf3,Il1rn,Cxcl1,Tnfsf10,Cxcl16,Ccl4,Pik3r1,Il6,Cxcl9,Ripk1,Ccl2 | | 2.845e-08 | -17.38 | regulation of signaling | biological process | GO:0023051 | 2632 | 75 | 13711 | 214 | Casp4,Il1a,Akap12,Nfkbia,Il6,Socs3,Irf7,Ccl7,Trib1,Parp14,Hcar2,Pik3r1,Gadd45b,Timp1,Fam107a,Fzd4,Map3k6,Parp9,Rasip1,Ccl4,Ptgs2,Runx1,Ier3,Trim25,Acod1,Tnf,Pik3ap1,Atf3,Hspb1,Ncf1,Cflar,Cd14,Ripk1,Tnip1,Fstl1,Rnf213,Trim30a,Tgm2,C5ar1,Hspa5,Nfe2l2,Mmp8,Selp,Icam1,Rgs16,Sox11,Errfi1,Thbs1,Gadd45g,Lgals9,P2ry6,Map3k8,Birc3,Nfkb1,Irgm1,Pik3r5,Plaur,Tnfaip3,Plek,Ksr1,Tnfsf10,Xdh,Ccl2,Irak3,Il1rn,Csf3,Ptpn1,Tnip3,S100a8,Iqgap1,Ccl12,Sgk3,S100a9,Bcl3,Irgm2 | | 2.851e-08 | -17.37 | regulation of programmed cell death | biological process | GO:0043067 | 1253 | 46 | 13711 | 214 | Zfp36,Atf3,Tnf,Cflar,Ripk1,Hspb1,Hspa5,Tgm2,C5ar1,Ifit2,Angptl4,Nfe2l2,Cdkn1a,Ip6k2,Il6,Akap12,Casp4,Pik3r1,Hcar2,Ccnd2,Gadd45b,Socs3,Timp1,Ier3,Sgk1,Ptgs2,Tnfsf10,Xdh,Tnfaip3,Zbtb16,Tnfaip8,Irak3,Il1rn,Ccl12,S100a8,Ptpn1,Bcl3,S100a9,Sgk3,Mt1,Icam1,Gadd45g,Thbs1,Nfkb1,Birc3,Plaur | | 2.987e-08 | -17.33 | regulation of cell death | biological process | GO:0010941 | 1386 | 49 | 13711 | 214 | Ifit2,Angptl4,Nfe2l2,Hp,Hspa5,Tgm2,C5ar1,Cflar,Ripk1,Hspb1,Zfp36,Atf3,Tnf,Ier3,Sgk1,Ptgs2,Timp1,Hcar2,Pik3r1,Ccnd2,Gadd45b,Socs3,Cdkn1a,Il6,Ip6k2,Akap12,Casp4,Bcl3,S100a9,Sgk3,Ccl12,S100a8,Ptpn1,Csf3,Zbtb16,Tnfaip8,Il1rn,Irak3,Tnfsf10,Xdh,Tnfaip3,Plaur,Nfkb1,Birc3,Gadd45g,Thbs1,Sox11,Mt1,Icam1 | | 3.071e-08 | -17.30 | GO\_REGULATION\_OF\_CYSTEINE\_TYPE\_ENDOPEPTIDASE\_ACTIVITY | MSigDB lists | GO\_REGULATION\_OF\_CYSTEINE\_TYPE\_ENDOPEPTIDASE\_ACTIVITY | 171 | 15 | 12187 | 179 | Tnf,Birc3,Ripk1,Il6,Ifi211,S100a9,Tnfaip8,S100a8,Xdh,Tnfsf10,Ifi209,Thbs1,Ifi204,Ifi207,Plaur | | 3.071e-08 | -17.30 | GO\_ACTIVATION\_OF\_INNATE\_IMMUNE\_RESPONSE | MSigDB lists | GO\_ACTIVATION\_OF\_INNATE\_IMMUNE\_RESPONSE | 171 | 15 | 12187 | 179 | Ifi211,Irf7,Birc3,Nfkbia,Tnfaip3,Ripk1,Tnip1,Cd14,Irak3,Pik3ap1,Ifi209,Tnip3,Nfkb1,Ifi204,Ifi207 | | 3.198e-08 | -17.26 | activation of immune response | biological process | GO:0002253 | 185 | 16 | 13711 | 214 | Ifi209,Nfkbiz,C5ar1,Tnip3,Ifi211,Irgm2,Tnf,Pik3ap1,Nfkbia,Fyb,C3ar1,Irak3,Cd14,Tnip1,Lgals9,Ifi204 | | 3.224e-08 | -17.25 | HAHTOLA\_MYCOSIS\_FUNGOIDES\_CD4\_UP | MSigDB lists | HAHTOLA\_MYCOSIS\_FUNGOIDES\_CD4\_UP | 49 | 9 | 12187 | 179 | Ccl4,Trib1,Cdkn1a,Atf3,Ier3,Cebpd,Cxcl1,Ptgs2,Plaur | | 3.235e-08 | -17.25 | THEILGAARD\_NEUTROPHIL\_AT\_SKIN\_WOUND\_UP | MSigDB lists | THEILGAARD\_NEUTROPHIL\_AT\_SKIN\_WOUND\_UP | 65 | 10 | 12187 | 179 | Nfkb1,Ccl12,Ier3,Nfe2l2,Tnf,Trib1,Zfp36,Plek,Cflar,Cxcl1 | | 3.290e-08 | -17.23 | negative regulation of cellular process | biological process | GO:0048523 | 3771 | 96 | 13711 | 214 | C5ar1,Clic4,Hspa5,Serpina3f,Nfe2l2,Rasd1,Noct,Angptl4,Acod1,Pik3ap1,Atf3,Trim30a,Rnf213,Timp1,Fam107a,Slfn2,Isg15,Cebpd,Rasip1,Xaf1,Ifi211,Runx1,Il4ra,Nfkbia,Ip6k2,Slfn3,Parp14,Ccnd2,Ptpn1,Tnip3,Iqgap1,Wfdc21,Znfx1,Tnfaip3,Il1rn,Tnfaip8,Csf3,Zbtb16,Birc3,Nfkbie,Mt1,Icam1,Rgs16,Bbs12,Gadd45g,Lgals9,Rrp8,Dnajb1,Prg4,Hp,Robo4,Tnf,Zfp36,Hspb1,Ifi207,Cflar,Ripk1,Tnip1,Ifi209,Fzd4,Sbno2,Sdc4,Ptges,Parp9,Spi1,Sgk1,Ptgs2,Ier3,Il1a,Il6,Cdkn1a,Socs3,Dtx3l,Trib1,Ctla2a,Pik3r1,Gadd45b,A2m,Ccl12,Sgk3,Bcl3,Plek,Xdh,Arid5b,Irak3,Adamts9,Ifi204,Gbp4,Nfkb1,Bach1,Plaur,Mxd4,Sox11,Errfi1,Thbs1,Ifitm3 | | 3.335e-08 | -17.22 | CRX\_DN.V1\_DN | MSigDB lists | CRX\_DN.V1\_DN | 103 | 12 | 12187 | 179 | Tagln2,Cdkn1a,Socs3,Ksr1,Timp1,Nfkb1,Cebpd,Bcl3,Osmr,Map3k8,Tgm2,Tubb6 | | 3.462e-08 | -17.18 | regulation of immune response | biological process | GO:0050776 | 516 | 27 | 13711 | 214 | Tnip3,A2m,Tap1,Nfkbiz,C5ar1,Irgm2,Pik3ap1,Tnfaip3,Tnf,Acod1,Trim30a,Ifi204,Tnip1,Cd14,C3ar1,Irak3,Parp9,Ifi209,Irgm1,Ifi211,Il6,Nfkbia,Fyb,Il4ra,Lgals9,Parp14,Irf7 | | 3.468e-08 | -17.18 | GSE42021\_CD24INT\_VS\_CD24LOW\_TCONV\_THYMUS\_DN | MSigDB lists | GSE42021\_CD24INT\_VS\_CD24LOW\_TCONV\_THYMUS\_DN | 148 | 14 | 12187 | 179 | Ifi44,Phf11b,Ifit3b,Irf9,Ifitm2,Herc6,Isg15,Parp12,Oasl1,Ifit2,Usp18,Tnfsf10,Irf7,Phf11d | | 3.591e-08 | -17.14 | GSE17721\_0.5H\_VS\_24H\_CPG\_BMDC\_DN | MSigDB lists | GSE17721\_0.5H\_VS\_24H\_CPG\_BMDC\_DN | 173 | 15 | 12187 | 179 | Gbp3,Irgm2,Gbp6,Gbp4,Oasl1,Gadd45b,Parp14,Usp18,Ccnd2,Birc3,Tap1,Slfn9,Tnip1,Cp,Fstl1 | | 3.591e-08 | -17.14 | GSE44649\_WT\_VS\_MIR155\_KO\_ACTIVATED\_CD8\_TCELL\_UP | MSigDB lists | GSE44649\_WT\_VS\_MIR155\_KO\_ACTIVATED\_CD8\_TCELL\_UP | 173 | 15 | 12187 | 179 | Parp14,Casp4,Irgm2,Cflar,Gbp7,Sbno2,Ifit3b,Znfx1,Il1rn,Slfn5,Slfn9,Herc6,Clic4,Parp9,Tap1 | | 3.698e-08 | -17.11 | MODULE\_171 | MSigDB lists | MODULE\_171 | 84 | 11 | 12187 | 179 | Ifitm3,Selp,Isg15,Ifitm2,Cd14,Cebpd,Thbs1,Ifi44,Vcam1,Tnfsf10,Csf3 | | 3.722e-08 | -17.11 | GO\_POSITIVE\_REGULATION\_OF\_NF\_KAPPAB\_TRANSCRIPTION\_FACTOR\_ACTIVITY | MSigDB lists | GO\_POSITIVE\_REGULATION\_OF\_NF\_KAPPAB\_TRANSCRIPTION\_FACTOR\_ACTIVITY | 104 | 12 | 12187 | 179 | Cflar,S100a9,Icam1,Irak3,Trim25,S100a8,Nfkb2,Tnf,Nfkbia,Nfkb1,Ripk1,Il6 | | 3.722e-08 | -17.11 | GSE43863\_NAIVE\_VS\_MEMORY\_TH1\_CD4\_TCELL\_D150\_LCMV\_UP | MSigDB lists | GSE43863\_NAIVE\_VS\_MEMORY\_TH1\_CD4\_TCELL\_D150\_LCMV\_UP | 104 | 12 | 12187 | 179 | Ifit3b,Samd9l,Cebpd,Atf3,Rnf213,Isg15,Tap1,Parp14,Bach1,Ccl12,Cmpk2,Rtp4 | | 3.756e-08 | -17.10 | negative regulation of immune system process | biological process | GO:0002683 | 355 | 22 | 13711 | 214 | Pik3r1,Lgals9,Trim30a,Thbs1,Irak3,Zbtb16,Parp14,Trib1,Zfp36,Tnf,Acod1,Il4ra,Tnfaip3,Pik3ap1,Sox11,Nfkbia,Runx1,Nfe2l2,A2m,Tap1,Sdc4,Ccl12 | | 3.779e-08 | -17.09 | GSE30083\_SP2\_VS\_SP4\_THYMOCYTE\_DN | MSigDB lists | GSE30083\_SP2\_VS\_SP4\_THYMOCYTE\_DN | 149 | 14 | 12187 | 179 | Tap1,Il17ra,Plek,Zbtb16,Socs3,Ifit3b,Sgk1,Ifi44,Ms4a6d,Gem,Xdh,Sdc4,Sntb2,Cybb | | 3.880e-08 | -17.06 | GSE45365\_NK\_CELL\_VS\_BCELL\_UP | MSigDB lists | GSE45365\_NK\_CELL\_VS\_BCELL\_UP | 174 | 15 | 12187 | 179 | Thbs1,Vcam1,Atf3,Selp,Zfp36,Icam1,Birc3,Ccl2,Bach1,Il6,Tnfaip3,Gem,Arid5b,Ptges,S100a8 | | 3.880e-08 | -17.06 | GSE25085\_FETAL\_BM\_VS\_ADULT\_BM\_SP4\_THYMIC\_IMPLANT\_DN | MSigDB lists | GSE25085\_FETAL\_BM\_VS\_ADULT\_BM\_SP4\_THYMIC\_IMPLANT\_DN | 174 | 15 | 12187 | 179 | Tnip1,Saa1,Icam1,Plek,Hspa5,Atf3,Il1rn,Hp,Nfkbie,Nfkbia,Gpr84,Casp4,Birc3,Tnfaip3,Rassf4 | | 4.116e-08 | -17.01 | GSE14699\_NAIVE\_VS\_DELETIONAL\_TOLERANCE\_CD8\_TCELL\_DN | MSigDB lists | GSE14699\_NAIVE\_VS\_DELETIONAL\_TOLERANCE\_CD8\_TCELL\_DN | 150 | 14 | 12187 | 179 | Irgm2,Rbm39,Rtp4,Cmpk2,Ifit2,Nfe2l2,Rnf213,Slfn9,Slfn5,Trim25,Clic4,Parp9,Sgk1,Ifit3b | | 4.190e-08 | -16.99 | GSE41867\_NAIVE\_VS\_DAY6\_LCMV\_EFFECTOR\_CD8\_TCELL\_UP | MSigDB lists | GSE41867\_NAIVE\_VS\_DAY6\_LCMV\_EFFECTOR\_CD8\_TCELL\_UP | 175 | 15 | 12187 | 179 | Cflar,Gbp6,Dtx3l,Xdh,S100a8,Nfkbia,Ifit2,Msr1,Gbp4,Rnf213,Trim25,Nfkbiz,Thbs1,Ksr1,Samd9l | | 4.226e-08 | -16.98 | cellular response to interferon-gamma | biological process | GO:0071346 | 80 | 11 | 13711 | 214 | Gbp4,Gbp3,Ccl12,Ccl4,Irgm1,Stx11,Gbp6,Gbp7,Acod1,Ccl2,Ccl7 | | 4.370e-08 | -16.95 | HINATA\_NFKB\_TARGETS\_FIBROBLAST\_UP | MSigDB lists | HINATA\_NFKB\_TARGETS\_FIBROBLAST\_UP | 67 | 10 | 12187 | 179 | Tnfaip3,Ccl12,Ier3,Il6,Nfkb1,Nfkbia,Nfkb2,Icam1,Tnfaip8,Sele | | 4.478e-08 | -16.92 | SCHUETZ\_BREAST\_CANCER\_DUCTAL\_INVASIVE\_UP | MSigDB lists | SCHUETZ\_BREAST\_CANCER\_DUCTAL\_INVASIVE\_UP | 288 | 19 | 12187 | 179 | Msr1,Ccnd2,Tgm2,Tubb6,C3ar1,Gem,Ifi211,Olfml2b,Sgk1,Ifi209,Fstl1,Ifi207,Col4a1,Ms4a6d,Ifi204,Irak3,Msn,Csf2rb,Plaur | | 4.479e-08 | -16.92 | GO\_CYTOKINE\_RECEPTOR\_BINDING | MSigDB lists | GO\_CYTOKINE\_RECEPTOR\_BINDING | 151 | 14 | 12187 | 179 | Il1rn,Il1a,Cxcl16,Ripk1,Ccl12,Il6,Ccl4,Ccl2,Tnf,Pik3r1,Tnfsf10,Cxcl1,Cflar,Csf3 | | 4.523e-08 | -16.91 | GO\_REGULATION\_OF\_I\_KAPPAB\_KINASE\_NF\_KAPPAB\_SIGNALING | MSigDB lists | GO\_REGULATION\_OF\_I\_KAPPAB\_KINASE\_NF\_KAPPAB\_SIGNALING | 176 | 15 | 12187 | 179 | Cflar,Bcl3,Tnfsf10,Tnf,Birc3,Nfkbia,Rhoc,Ripk1,Tnfaip3,Ptgs2,Tnip1,Trim25,Hspb1,Tnip3,Il1a | | 4.661e-08 | -16.88 | PHONG\_TNF\_RESPONSE\_VIA\_P38\_PARTIAL | MSigDB lists | PHONG\_TNF\_RESPONSE\_VIA\_P38\_PARTIAL | 128 | 13 | 12187 | 179 | Plaur,Thbs1,Cdkn1a,Cebpd,Ier3,Tiparp,Cxcl1,Nfkbie,Nfkb2,Il4ra,Maff,Il6,Tnfaip3 | | 4.770e-08 | -16.86 | GO\_POSITIVE\_REGULATION\_OF\_RESPONSE\_TO\_EXTERNAL\_STIMULUS | MSigDB lists | GO\_POSITIVE\_REGULATION\_OF\_RESPONSE\_TO\_EXTERNAL\_STIMULUS | 203 | 16 | 12187 | 179 | Cxcl1,S100a9,C5ar1,C3ar1,Osmr,S100a8,Ccl4,Ccl2,Tnf,Ccl12,Il6,Tnip1,Ptgs2,Il17ra,Hspb1,Thbs1 | | 4.817e-08 | -16.85 | positive regulation of immune response | biological process | GO:0050778 | 360 | 22 | 13711 | 214 | Il6,Nfkbia,Fyb,Pik3ap1,Acod1,Il4ra,Tnf,Ifi204,Lgals9,Tnip1,Cd14,Irak3,C3ar1,Irf7,Parp9,Tnip3,C5ar1,Ifi209,Nfkbiz,Irgm2,Irgm1,Ifi211 | | 4.871e-08 | -16.84 | HALLMARK\_COMPLEMENT | MSigDB lists | HALLMARK\_COMPLEMENT | 152 | 14 | 12187 | 179 | Pik3r5,Irf7,S100a9,Tnfaip3,Il6,Casp4,Maff,Plaur,Irf2,Plek,Timp1,Mmp8,Cp,Hspa5 | | 5.051e-08 | -16.80 | negative regulation of viral life cycle | biological process | GO:1903901 | 64 | 10 | 13711 | 214 | Ifitm3,Oasl1,Ifitm6,Trim56,Resf1,Trim25,Oasl2,Tnf,Ifitm2,Isg15 | | 5.058e-08 | -16.80 | NABA\_MATRISOME\_ASSOCIATED | MSigDB lists | NABA\_MATRISOME\_ASSOCIATED | 386 | 22 | 12187 | 179 | S100a8,Adamts9,Tnfsf10,Csf3,A2m,S100a9,Cxcl1,Angptl4,Il6,Ccl12,Lgals9,Tnf,Ccl2,Sdc4,Ccl4,Tgm2,Il1a,Timp1,Mmp8,Il1rn,Adamts1,Fstl1 | | 5.293e-08 | -16.75 | GSE40443\_INDUCED\_VS\_TOTAL\_TREG\_DN | MSigDB lists | GSE40443\_INDUCED\_VS\_TOTAL\_TREG\_DN | 153 | 14 | 12187 | 179 | Cmpk2,Bcl3,Irgm2,Usp18,Msn,Irf9,Ptpn1,Slfn9,Tagln2,Rnf213,Ms4a6d,Socs3,Sgk1,Ifi44 | | 5.293e-08 | -16.75 | GSE37534\_UNTREATED\_VS\_ROSIGLITAZONE\_TREATED\_CD4\_TCELL\_PPARG1\_AND\_FOXP3\_TRASDUCED\_DN | MSigDB lists | GSE37534\_UNTREATED\_VS\_ROSIGLITAZONE\_TREATED\_CD4\_TCELL\_PPARG1\_AND\_FOXP3\_TRASDUCED\_DN | 153 | 14 | 12187 | 179 | Herc6,Icam1,Trim25,Zfp36,Tap1,Ifit3b,Atf3,Ifit2,Maff,Xaf1,Trib1,Usp18,Il6,Parp12 | | 5.496e-08 | -16.72 | positive regulation of tumor necrosis factor superfamily cytokine production | biological process | GO:1903557 | 82 | 11 | 13711 | 214 | Mmp8,Ccl4,Cybb,Ccl2,Akap12,Pik3r1,Lgals9,Hspb1,Thbs1,Cd14,Ripk1 | | 5.496e-08 | -16.72 | positive regulation of tumor necrosis factor production | biological process | GO:0032760 | 82 | 11 | 13711 | 214 | Mmp8,Ccl4,Ccl2,Cybb,Akap12,Lgals9,Pik3r1,Cd14,Ripk1,Hspb1,Thbs1 | | 5.560e-08 | -16.71 | REACTOME\_RIG\_I\_MDA5\_MEDIATED\_INDUCTION\_OF\_IFN\_ALPHA\_BETA\_PATHWAYS | MSigDB lists | REACTOME\_RIG\_I\_MDA5\_MEDIATED\_INDUCTION\_OF\_IFN\_ALPHA\_BETA\_PATHWAYS | 52 | 9 | 12187 | 179 | Tnfaip3,Ripk1,Nfkbia,Trim25,Nfkb2,Irf2,Isg15,Saa1,Irf7 | | 5.793e-08 | -16.66 | GO\_NEGATIVE\_REGULATION\_OF\_RESPONSE\_TO\_STIMULUS | MSigDB lists | GO\_NEGATIVE\_REGULATION\_OF\_RESPONSE\_TO\_STIMULUS | 1060 | 39 | 12187 | 179 | Thbs1,Hspa5,Nfkb1,Il1a,Rnf213,Cd14,Irak3,Pik3ap1,Ptpn1,Tap1,Il4ra,Ccl12,Tnfaip3,Ripk1,Nfe2l2,Ifi211,Rgs16,Ifi209,Socs3,Ifi204,Tnip3,Il1rn,Hp,Ifi207,Atf3,Saa1,Tnip1,Hspb1,Icam1,Plek,Plaur,Errfi1,Tnf,Trib1,Nfkbia,Cflar,A2m,Xdh,Tnfsf10 | | 6.154e-08 | -16.60 | GSE9988\_ANTI\_TREM1\_AND\_LPS\_VS\_VEHICLE\_TREATED\_MONOCYTES\_UP | MSigDB lists | GSE9988\_ANTI\_TREM1\_AND\_LPS\_VS\_VEHICLE\_TREATED\_MONOCYTES\_UP | 131 | 13 | 12187 | 179 | Il1a,Tnip3,Ier3,Nfkbiz,Plek,Plaur,Ptgs2,Map3k8,Tnf,Gem,Cflar,Banp,Cxcl1 | | 6.218e-08 | -16.59 | GO\_NEGATIVE\_REGULATION\_OF\_INNATE\_IMMUNE\_RESPONSE | MSigDB lists | GO\_NEGATIVE\_REGULATION\_OF\_INNATE\_IMMUNE\_RESPONSE | 38 | 8 | 12187 | 179 | Ifi209,Ifi207,Ifi204,Tnfaip3,A2m,Ifi211,Irak3,Tap1 | | 6.250e-08 | -16.59 | positive regulation of reactive oxygen species metabolic process | biological process | GO:2000379 | 83 | 11 | 13711 | 214 | Il6,Xdh,Cdkn1a,Icam1,Tnf,Acod1,Cxcl1,Ptgs2,Thbs1,Nfe2l2,Mmp8 | | 6.268e-08 | -16.59 | ACEVEDO\_FGFR1\_TARGETS\_IN\_PROSTATE\_CANCER\_MODEL\_UP | MSigDB lists | ACEVEDO\_FGFR1\_TARGETS\_IN\_PROSTATE\_CANCER\_MODEL\_UP | 207 | 16 | 12187 | 179 | Plaur,Cxcl16,Socs3,Ifi44,Thbs1,Lcn2,Cp,Timp1,Fzd4,Bcl3,Cxcl1,Irgm2,Rtp4,Usp18,Tnfaip3,Ccl12 | | 6.664e-08 | -16.52 | positive regulation of multicellular organismal process | biological process | GO:0051240 | 1512 | 51 | 13711 | 214 | Selp,Nfe2l2,Mmp8,Cxcl1,C5ar1,Hspa5,Hspb1,Cd14,Cflar,Ripk1,Tnf,Sgk1,Ptgs2,Runx1,Cebpd,Ifi211,Ccl4,Isg15,Fzd4,Hcar2,Pik3r1,Irf7,Trib1,Il6,Casp4,Il4ra,Akap12,Il1a,Bcl3,S100a9,Iqgap1,Nfkbiz,Lcn2,Ifi204,Irak3,C3ar1,Il1rn,Zbtb16,Csf3,Ccl2,Tnfaip3,Plek,Il17ra,Rhoj,Adamts1,Gadd45g,Lgals9,Thbs1,Cybb,Icam1,Sox11 | | 6.729e-08 | -16.51 | GO\_POSITIVE\_REGULATION\_OF\_LEUKOCYTE\_MIGRATION | MSigDB lists | GO\_POSITIVE\_REGULATION\_OF\_LEUKOCYTE\_MIGRATION | 70 | 10 | 12187 | 179 | Ccl4,Thbs1,Tnf,Il6,Ccl12,Cxcl1,C3ar1,C5ar1,Selp,Icam1 | | 6.768e-08 | -16.51 | GSE42021\_CD24HI\_VS\_CD24LOW\_TCONV\_THYMUS\_DN | MSigDB lists | GSE42021\_CD24HI\_VS\_CD24LOW\_TCONV\_THYMUS\_DN | 156 | 14 | 12187 | 179 | Tgm2,Il4ra,Maff,Ifit2,Oasl1,Tnfaip3,Bach1,Cxcl1,Tnfsf10,Rtp4,Ifit3b,Tap1,Plaur,Zfp36 | | 7.017e-08 | -16.47 | GO\_CELL\_CHEMOTAXIS | MSigDB lists | GO\_CELL\_CHEMOTAXIS | 110 | 12 | 12187 | 179 | S100a8,Il17ra,Cxcl16,Saa1,C5ar1,S100a9,Cxcl1,Il6,Ccl12,Ccl2,Ccl4,Vcam1 | | 7.376e-08 | -16.42 | RPS14\_DN.V1\_UP | MSigDB lists | RPS14\_DN.V1\_UP | 133 | 13 | 12187 | 179 | Ifi204,Ifi207,Ms4a6d,Ifi209,Cdkn1a,Rbm47,Cd14,Ccl12,Sdc4,Birc3,Igsf6,Ifi211,C3ar1 | | 7.674e-08 | -16.38 | negative regulation of metabolic process | biological process | GO:0009892 | 2285 | 67 | 13711 | 214 | Lgals9,Gadd45g,Banp,Msr1,Thbs1,Errfi1,Sox11,Mxd4,Plaur,Bach1,Nfkbie,Nfkb1,Birc3,Gbp4,Ifi204,Zbtb16,Tnfaip8,Arid5b,Irak3,Xdh,Plek,Tnfaip3,Znfx1,Wfdc21,Bcl3,A2m,Iqgap1,Ptpn1,Pik3r1,Hcar2,Gadd45b,Parp14,Trib1,Ctla2a,Socs3,Dtx3l,Cdkn1a,Il6,Ier3,Ptgs2,Runx1,Spi1,Cebpd,Rasip1,Ifi211,Isg15,Sbno2,Parp9,Timp1,Tiparp,Ifi209,Trim30a,Tnip1,Cflar,Hspb1,Ifi207,Zfp36,Atf3,Tnf,Noct,Rasd1,Mmp8,Serpina3f,Prg4,Hp,Dnajb1,Rrp8 | | 7.675e-08 | -16.38 | GO\_DEFENSE\_RESPONSE\_TO\_BACTERIUM | MSigDB lists | GO\_DEFENSE\_RESPONSE\_TO\_BACTERIUM | 90 | 11 | 12187 | 179 | Il6,Gbp4,Hp,Tnf,S100a8,S100a9,Bcl3,Gbp6,Isg15,C5ar1,Selp | | 7.947e-08 | -16.35 | GSE19401\_UNSTIM\_VS\_RETINOIC\_ACID\_STIM\_FOLLICULAR\_DC\_DN | MSigDB lists | GSE19401\_UNSTIM\_VS\_RETINOIC\_ACID\_STIM\_FOLLICULAR\_DC\_DN | 158 | 14 | 12187 | 179 | Tnf,Ccl4,Nfkbia,Sdc4,Tnfaip3,Cflar,Gem,Nfkb2,Thbs1,Vcam1,Nfkb1,Gadd45g,Atf3,Tnip1 | | 8.027e-08 | -16.34 | negative regulation of catalytic activity | biological process | GO:0043086 | 573 | 28 | 13711 | 214 | Cflar,Tnfaip8,Hspb1,Irak3,Tnfaip3,Tnf,Zfp36,Serpina3f,Wfdc21,Angptl4,Ptpn1,A2m,Hp,Trib1,Dtx3l,Thbs1,Gadd45g,Gadd45b,Cdkn1a,Errfi1,Il6,Rasip1,Plaur,Ptgs2,Birc3,Timp1,Parp9,Nfkb1 | | 8.465e-08 | -16.28 | GO\_REGULATION\_OF\_MAPK\_CASCADE | MSigDB lists | GO\_REGULATION\_OF\_MAPK\_CASCADE | 503 | 25 | 12187 | 179 | Ksr1,Atf3,Saa1,Tnip1,Icam1,Errfi1,Tnf,Trib1,C5ar1,Fzd4,Xdh,Thbs1,Gadd45g,Il1a,Map3k6,Irak3,Ptpn1,Map3k8,Ccl2,Ccl4,Il6,Gadd45b,Ripk1,Ccl12,Pik3r5 | | 8.604e-08 | -16.27 | GSE11961\_GERMINAL\_CENTER\_BCELL\_DAY7\_VS\_PLASMA\_CELL\_DAY7\_UP | MSigDB lists | GSE11961\_GERMINAL\_CENTER\_BCELL\_DAY7\_VS\_PLASMA\_CELL\_DAY7\_UP | 159 | 14 | 12187 | 179 | Hspa5,Ifi44,Parp9,Clic4,Slfn3,AA467197,Oasl1,Ccnd2,Birc3,Sbno2,Tnfsf10,Slfn4,Cflar,Dtx3l | | 8.811e-08 | -16.24 | GSE6269\_HEALTHY\_VS\_STAPH\_PNEUMO\_INF\_PBMC\_DN | MSigDB lists | GSE6269\_HEALTHY\_VS\_STAPH\_PNEUMO\_INF\_PBMC\_DN | 135 | 13 | 12187 | 179 | Cd14,Plek,Trim25,Plaur,Csf2rb,Socs3,Il1rn,Lcn2,Bcl3,S100a9,S100a8,Spi1,Cybb | | 8.868e-08 | -16.24 | KIM\_GLIS2\_TARGETS\_UP | MSigDB lists | KIM\_GLIS2\_TARGETS\_UP | 72 | 10 | 12187 | 179 | Osmr,Icam1,Angptl4,Isg15,Ifitm3,Ccl12,Col4a1,Casp4,Vcam1,Socs3 | | 8.964e-08 | -16.23 | Cytokine Signaling in Immune system | REACTOME pathways | R-MMU-1280215 | 364 | 22 | 6297 | 105 | Usp18,Nfkb2,Socs3,Il1a,Il6,Il4ra,Il1rn,Irak3,Birc3,Pik3r1,Tnf,Map3k8,Irf9,Osmr,Csf3,Isg15,Csf2rb,Il17ra,Ksr1,Ptpn1,Lgals9,Nfkb1 | | 9.087e-08 | -16.21 | DUTTA\_APOPTOSIS\_VIA\_NFKB | MSigDB lists | DUTTA\_APOPTOSIS\_VIA\_NFKB | 27 | 7 | 12187 | 179 | Tnfaip3,Ier3,Gadd45b,Tnf,Birc3,Tnfsf10,Cflar | | 9.310e-08 | -16.19 | GSE20715\_0H\_VS\_24H\_OZONE\_LUNG\_DN | MSigDB lists | GSE20715\_0H\_VS\_24H\_OZONE\_LUNG\_DN | 160 | 14 | 12187 | 179 | Maff,Trib1,Cflar,Banp,Osmr,Zbtb16,Timp1,Col4a1,Hp,Cp,Lcn2,Map3k6,Saa1,Rbm47 | | 9.310e-08 | -16.19 | GSE17721\_LPS\_VS\_PAM3CSK4\_2H\_BMDC\_UP | MSigDB lists | GSE17721\_LPS\_VS\_PAM3CSK4\_2H\_BMDC\_UP | 160 | 14 | 12187 | 179 | Sdc4,Ccl2,Ccl4,Tnf,Il6,Ccl12,Arid5b,Csf3,Ifit3b,Il1a,Isg15,Plek,Zfp36,Sele | | 1.007e-07 | -16.11 | GSE17721\_POLYIC\_VS\_CPG\_1H\_BMDC\_DN | MSigDB lists | GSE17721\_POLYIC\_VS\_CPG\_1H\_BMDC\_DN | 161 | 14 | 12187 | 179 | Tgm2,Gadd45b,Hcar2,A2m,Nfkbie,Slfn4,Il1rn,Tiparp,Tnip1,Cd14,Saa1,Errfi1,Slfn3,Zfp36 | | 1.165e-07 | -15.97 | NFKAPPAB\_01 | MSigDB lists | NFKAPPAB\_01 | 189 | 15 | 12187 | 179 | Tnip1,Icam1,Msn,Cxcl16,Vcam1,Il1a,Il1rn,Bcl3,Cxcl1,Nfkb2,Birc3,Tnf,Map3k8,Sdc4,Gadd45b | | 1.175e-07 | -15.96 | GSE32986\_CURDLAN\_LOWDOSE\_VS\_CURDLAN\_HIGHDOSE\_STIM\_DC\_DN | MSigDB lists | GSE32986\_CURDLAN\_LOWDOSE\_VS\_CURDLAN\_HIGHDOSE\_STIM\_DC\_DN | 163 | 14 | 12187 | 179 | Il1rn,Ifit3b,Plaur,Cxcl16,Ccl12,Gadd45b,Ifit2,Tor3a,Tgm2,Ccl4,Casp4,Spi1,Sdc4,Cflar | | 1.175e-07 | -15.96 | GSE1432\_1H\_VS\_6H\_IFNG\_MICROGLIA\_DN | MSigDB lists | GSE1432\_1H\_VS\_6H\_IFNG\_MICROGLIA\_DN | 163 | 14 | 12187 | 179 | Birc3,Casp4,Sntb2,Parp12,Nfkbie,Irf7,Phf11d,Tnfsf10,Ifi44,Phf11b,Akap12,Herc6,Tap1,Csf2rb | | 1.175e-07 | -15.96 | GSE17721\_LPS\_VS\_PAM3CSK4\_1H\_BMDC\_DN | MSigDB lists | GSE17721\_LPS\_VS\_PAM3CSK4\_1H\_BMDC\_DN | 163 | 14 | 12187 | 179 | Tnip1,Ptgs2,Icam1,Vcam1,Fstl1,Nfkbiz,Cdkn1a,Cxcl1,Nfkbia,Ccnd2,Map3k8,Birc3,Parp12,Gadd45b | | 1.175e-07 | -15.96 | GSE17721\_CTRL\_VS\_POLYIC\_4H\_BMDC\_DN | MSigDB lists | GSE17721\_CTRL\_VS\_POLYIC\_4H\_BMDC\_DN | 163 | 14 | 12187 | 179 | Irgm2,Hcar2,Ccl12,Parp12,Casp4,Pik3ap1,Tap1,Ptgs2,Il1rn,Ms4a6d,Znfx1,Cebpd,Atf3,Cdkn1a | | 1.175e-07 | -15.96 | GSE9988\_LPS\_VS\_LPS\_AND\_ANTI\_TREM1\_MONOCYTE\_UP | MSigDB lists | GSE9988\_LPS\_VS\_LPS\_AND\_ANTI\_TREM1\_MONOCYTE\_UP | 163 | 14 | 12187 | 179 | Pik3r5,Birc3,Map3k8,Sdc4,Cybb,Nfe2l2,Oasl1,Tnfaip3,Tnip1,Pik3ap1,Clic4,Thbs1,Nfkbiz,Socs3 | | 1.175e-07 | -15.96 | GSE17721\_0.5H\_VS\_4H\_CPG\_BMDC\_DN | MSigDB lists | GSE17721\_0.5H\_VS\_4H\_CPG\_BMDC\_DN | 163 | 14 | 12187 | 179 | Aff1,Nfkb1,Cp,Ifitm2,Tap1,Icam1,Tgm2,Tor3a,Birc3,Ptges,Irgm2,Gbp7,Tnfsf10,Rtp4 | | 1.288e-07 | -15.87 | DELYS\_THYROID\_CANCER\_UP | MSigDB lists | DELYS\_THYROID\_CANCER\_UP | 340 | 20 | 12187 | 179 | C3ar1,Cxcl1,Igsf6,Runx1,Ccnd2,Msr1,Tgm2,Ccl2,Sdc4,Map3k6,Icam1,Cdkn1a,Thbs1,Timp1,Il1rn,Tiparp,Hp,Ms4a6d,Ier3,Lcn2 | | 1.352e-07 | -15.82 | GO\_REGULATION\_OF\_LEUKOCYTE\_MIGRATION | MSigDB lists | GO\_REGULATION\_OF\_LEUKOCYTE\_MIGRATION | 95 | 11 | 12187 | 179 | Cxcl1,C3ar1,C5ar1,Selp,Msn,Icam1,Ccl4,Thbs1,Tnf,Il6,Ccl12 | | 1.356e-07 | -15.81 | GSE30971\_WBP7\_HET\_VS\_KO\_MACROPHAGE\_DN | MSigDB lists | GSE30971\_WBP7\_HET\_VS\_KO\_MACROPHAGE\_DN | 140 | 13 | 12187 | 179 | Bach1,Il6,Gadd45b,Tnfaip8,Csf3,Cxcl1,Tnip3,Ier3,Dnajb1,Socs3,Pik3ap1,P2ry6,Ptgs2 | | 1.356e-07 | -15.81 | NAGASHIMA\_NRG1\_SIGNALING\_UP | MSigDB lists | NAGASHIMA\_NRG1\_SIGNALING\_UP | 140 | 13 | 12187 | 179 | Tiparp,Ier3,Atf3,Sgk1,Dnajb1,Zfp36,Plaur,Tubb6,Gadd45b,Maff,Map3k8,Trib1,Gem | | 1.393e-07 | -15.79 | chemokine activity | molecular function | GO:0008009 | 27 | 7 | 13516 | 211 | Cxcl9,Cxcl1,Ccl2,Ccl4,Ccl7,Cxcl16,Ccl12 | | 1.427e-07 | -15.76 | GO\_REGULATION\_OF\_INTERLEUKIN\_1\_PRODUCTION | MSigDB lists | GO\_REGULATION\_OF\_INTERLEUKIN\_1\_PRODUCTION | 42 | 8 | 12187 | 179 | Errfi1,Hspb1,Ifi211,Saa1,Tnfaip3,Ifi204,Ifi207,Ifi209 | | 1.476e-07 | -15.73 | GSE17721\_12H\_VS\_24H\_PAM3CSK4\_BMDC\_UP | MSigDB lists | GSE17721\_12H\_VS\_24H\_PAM3CSK4\_BMDC\_UP | 166 | 14 | 12187 | 179 | Gem,Bcl3,Rrp8,S100a8,Ccl2,Rhoc,Gadd45b,Nfe2l2,Icam1,P2ry6,Cdkn1a,Fstl1,Ifit3b,Kcna5 | | 1.478e-07 | -15.73 | negative regulation of nitrogen compound metabolic process | biological process | GO:0051172 | 1880 | 58 | 13711 | 214 | Isg15,Sbno2,Parp9,Ifi209,Timp1,Ier3,Ptgs2,Runx1,Spi1,Rasip1,Cebpd,Ifi211,Cdkn1a,Il6,Pik3r1,Gadd45b,Parp14,Trib1,Ctla2a,Socs3,Dtx3l,Prg4,Dnajb1,Rrp8,Noct,Rasd1,Serpina3f,Zfp36,Atf3,Tnf,Tnip1,Cflar,Hspb1,Ifi207,Nfkbie,Nfkb1,Birc3,Gbp4,Plaur,Bach1,Errfi1,Sox11,Mxd4,Gadd45g,Banp,Thbs1,A2m,Ptpn1,Wfdc21,Bcl3,Xdh,Tnfaip3,Znfx1,Ifi204,Tnfaip8,Zbtb16,Arid5b,Irak3 | | 1.480e-07 | -15.73 | TONKS\_TARGETS\_OF\_RUNX1\_RUNX1T1\_FUSION\_SUSTAINED\_IN\_GRANULOCYTE\_UP | MSigDB lists | TONKS\_TARGETS\_OF\_RUNX1\_RUNX1T1\_FUSION\_SUSTAINED\_IN\_GRANULOCYTE\_UP | 18 | 6 | 12187 | 179 | Ifi207,Ifi204,Hspb1,Ifi209,Arid5b,Ifi211 | | 1.501e-07 | -15.71 | MODULE\_345 | MSigDB lists | MODULE\_345 | 76 | 10 | 12187 | 179 | Cebpd,Parp14,Ifi44,Thbs1,Tap1,Tnfsf10,Cd14,Csf3,Isg15,Ifitm3 | | 1.501e-07 | -15.71 | MODULE\_292 | MSigDB lists | MODULE\_292 | 76 | 10 | 12187 | 179 | Vcam1,Thbs1,Ifi44,Cebpd,Cd14,Isg15,Csf3,Ifitm2,Ifitm3,Tnfsf10 | | 1.501e-07 | -15.71 | GO\_LEUKOCYTE\_CHEMOTAXIS | MSigDB lists | GO\_LEUKOCYTE\_CHEMOTAXIS | 76 | 10 | 12187 | 179 | Il17ra,S100a8,Cxcl16,C5ar1,Saa1,S100a9,Ccl12,Il6,Ccl4,Ccl2 | | 1.529e-07 | -15.69 | GO\_REGULATION\_OF\_SEQUENCE\_SPECIFIC\_DNA\_BINDING\_TRANSCRIPTION\_FACTOR\_ACTIVITY | MSigDB lists | GO\_REGULATION\_OF\_SEQUENCE\_SPECIFIC\_DNA\_BINDING\_TRANSCRIPTION\_FACTOR\_ACTIVITY | 280 | 18 | 12187 | 179 | Fzd4,Arid5b,S100a9,Cflar,Nfkb2,S100a8,Nfkbia,Trib1,Tnf,Tnfaip3,Ripk1,Il6,Sgk3,Icam1,Irak3,Trim25,Sgk1,Nfkb1 | | 1.535e-07 | -15.69 | DAZARD\_UV\_RESPONSE\_CLUSTER\_G28 | MSigDB lists | DAZARD\_UV\_RESPONSE\_CLUSTER\_G28 | 10 | 5 | 12187 | 179 | Cxcl1,Ptgs2,Atf3,Ier3,Gadd45b | | 1.535e-07 | -15.69 | GO\_CHRONIC\_INFLAMMATORY\_RESPONSE | MSigDB lists | GO\_CHRONIC\_INFLAMMATORY\_RESPONSE | 10 | 5 | 12187 | 179 | Ptges,Vcam1,Thbs1,Tnf,S100a8 | | 1.535e-07 | -15.69 | BIOCARTA\_GRANULOCYTES\_PATHWAY | MSigDB lists | BIOCARTA\_GRANULOCYTES\_PATHWAY | 10 | 5 | 12187 | 179 | Icam1,Il1a,Tnf,Selp,Csf3 | | 1.547e-07 | -15.68 | toll-like receptor signaling pathway | biological process | GO:0002224 | 40 | 8 | 13711 | 214 | Nfkbia,Pik3ap1,Tnf,Tnip3,Cd14,Irak3,Lgals9,Tnip1 | | 1.586e-07 | -15.66 | pattern recognition receptor signaling pathway | biological process | GO:0002221 | 55 | 9 | 13711 | 214 | Tnip3,Nfkbia,Pik3ap1,Tnf,Irgm2,Lgals9,Tnip1,Cd14,Irak3 | | 1.586e-07 | -15.66 | innate immune response-activating signal transduction | biological process | GO:0002758 | 55 | 9 | 13711 | 214 | Tnip3,Tnf,Pik3ap1,Nfkbia,Tnip1,Lgals9,Irgm2,Irak3,Cd14 | | 1.590e-07 | -15.65 | GSE19923\_HEB\_KO\_VS\_HEB\_AND\_E2A\_KO\_DP\_THYMOCYTE\_DN | MSigDB lists | GSE19923\_HEB\_KO\_VS\_HEB\_AND\_E2A\_KO\_DP\_THYMOCYTE\_DN | 167 | 14 | 12187 | 179 | Nfkb2,Slfn4,Il6,Gadd45b,Ccl4,Ccl2,Nfkbia,Ccnd2,Tnf,Ptpn1,Slfn3,Ier3,Atf3,Nfkb1 | | 1.590e-07 | -15.65 | GSE22282\_HYPOXIA\_VS\_NORMOXIA\_MYELOID\_DC\_UP | MSigDB lists | GSE22282\_HYPOXIA\_VS\_NORMOXIA\_MYELOID\_DC\_UP | 167 | 14 | 12187 | 179 | Hp,Il1rn,Stx11,Cxcl16,Sele,Icam1,Tnip1,Saa1,Tnfaip3,Nfkbia,Gpr84,Tnf,Igsf6,Nfkbie | | 1.590e-07 | -15.65 | GSE37301\_LYMPHOID\_PRIMED\_MPP\_VS\_COMMON\_LYMPHOID\_PROGENITOR\_DN | MSigDB lists | GSE37301\_LYMPHOID\_PRIMED\_MPP\_VS\_COMMON\_LYMPHOID\_PROGENITOR\_DN | 167 | 14 | 12187 | 179 | Il1rn,Cebpd,Rasd1,Znfx1,Sgk1,Ifit3b,Trim25,Map3k6,Cd14,Gadd45b,Ccl12,Usp18,Cmpk2,Xdh | | 1.590e-07 | -15.65 | NABA\_SECRETED\_FACTORS | MSigDB lists | NABA\_SECRETED\_FACTORS | 167 | 14 | 12187 | 179 | Ccl12,Il6,Ccl4,Ccl2,Tnf,Tnfsf10,S100a8,Cxcl1,Angptl4,S100a9,Csf3,Il1rn,Il1a,Fstl1 | | 1.606e-07 | -15.64 | GO\_POSITIVE\_REGULATION\_OF\_IMMUNE\_RESPONSE | MSigDB lists | GO\_POSITIVE\_REGULATION\_OF\_IMMUNE\_RESPONSE | 378 | 21 | 12187 | 179 | Ifi204,Nfkb1,Tnip3,Ifi207,Ifi209,Irak3,Pik3ap1,Tnip1,Cd14,Tnfaip3,Ripk1,Il6,Nfkbia,Tnf,Il4ra,Pik3r1,Birc3,Ifi211,Irf7,C5ar1,C3ar1 | | 1.684e-07 | -15.60 | Apoptosis | WikiPathways | WP1254 | 72 | 12 | 3756 | 96 | Tnf,Ripk1,Cflar,Irf2,Casp4,Nfkb1,Irf7,Pik3r1,Nfkbie,Nfkbia,Birc3,Tnfsf10 | | 1.713e-07 | -15.58 | GSE26343\_WT\_VS\_NFAT5\_KO\_MACROPHAGE\_DN | MSigDB lists | GSE26343\_WT\_VS\_NFAT5\_KO\_MACROPHAGE\_DN | 168 | 14 | 12187 | 179 | Gbp3,Irf7,Csf3,Nfe2l2,Oasl1,Tubb6,Casp4,Map3k8,Ptpn1,Irf9,Irak3,Saa1,Znfx1,Socs3 | | 1.732e-07 | -15.57 | BROWNE\_HCMV\_INFECTION\_4HR\_UP | MSigDB lists | BROWNE\_HCMV\_INFECTION\_4HR\_UP | 43 | 8 | 12187 | 179 | Ifit3b,Thbs1,Ifit2,Oasl1,Lcn2,Ripk1,Isg15,Ptgs2 | | 1.741e-07 | -15.56 | GSE30083\_SP3\_VS\_SP4\_THYMOCYTE\_DN | MSigDB lists | GSE30083\_SP3\_VS\_SP4\_THYMOCYTE\_DN | 143 | 13 | 12187 | 179 | Xdh,Irf7,Gem,Ripk1,Rhoc,Cybb,Tor3a,Sdc4,Sntb2,Irf9,Plek,Ifi44,Sgk1 | | 1.741e-07 | -15.56 | GSE360\_T\_GONDII\_VS\_M\_TUBERCULOSIS\_MAC\_DN | MSigDB lists | GSE360\_T\_GONDII\_VS\_M\_TUBERCULOSIS\_MAC\_DN | 143 | 13 | 12187 | 179 | Fstl1,Cdkn1a,Ptgs2,Ptpn1,Zfp36,Ccl4,Zfp189,Il6,Cxcl1,Ptges,Nfkb2,Sbno2,Pygm | | 1.756e-07 | -15.55 | GO\_POSITIVE\_REGULATION\_OF\_INNATE\_IMMUNE\_RESPONSE | MSigDB lists | GO\_POSITIVE\_REGULATION\_OF\_INNATE\_IMMUNE\_RESPONSE | 195 | 15 | 12187 | 179 | Ifi204,Nfkb1,Tnip3,Ifi207,Ifi209,Irak3,Pik3ap1,Tnip1,Cd14,Tnfaip3,Ripk1,Nfkbia,Birc3,Ifi211,Irf7 | | 1.867e-07 | -15.49 | GSE43863\_NAIVE\_VS\_LY6C\_LOW\_CXCR5NEG\_CD4\_EFF\_TCELL\_D6\_LCMV\_UP | MSigDB lists | GSE43863\_NAIVE\_VS\_LY6C\_LOW\_CXCR5NEG\_CD4\_EFF\_TCELL\_D6\_LCMV\_UP | 98 | 11 | 12187 | 179 | Vcam1,Atf3,Sele,Ccl4,Gbp4,AA467197,Ccl12,Cxcl1,Gbp6,Nfkbie,Nfkb2 | | 1.915e-07 | -15.47 | symbiont-containing vacuole | cellular component | GO:0020003 | 10 | 5 | 13825 | 212 | Irgm2,Gbp6,Gbp3,Iigp1,Gbp7 | | 1.966e-07 | -15.44 | GO\_POSITIVE\_REGULATION\_OF\_MAPK\_CASCADE | MSigDB lists | GO\_POSITIVE\_REGULATION\_OF\_MAPK\_CASCADE | 349 | 20 | 12187 | 179 | Ccl4,Ccl2,Map3k8,Tnf,Ccl12,Ripk1,Gadd45b,Il6,Pik3r5,Fzd4,C5ar1,Xdh,Thbs1,Ksr1,Il1a,Gadd45g,Map3k6,Saa1,Ptpn1,Icam1 | | 1.966e-07 | -15.44 | MITSIADES\_RESPONSE\_TO\_APLIDIN\_UP | MSigDB lists | MITSIADES\_RESPONSE\_TO\_APLIDIN\_UP | 349 | 20 | 12187 | 179 | Ifitm3,Icam1,Ip6k2,Timp1,Ksr1,Irf7,Bcl3,Arid5b,Gem,C3ar1,Tnfsf10,Tor3a,Trib1,Nfkbia,Runx1,Maff,Nfe2l2,Oasl1,Gadd45b,Bach1 | | 1.984e-07 | -15.43 | GSE22601\_IMMATURE\_CD4\_SINGLE\_POSITIVE\_VS\_DOUBLE\_POSITIVE\_THYMOCYTE\_DN | MSigDB lists | GSE22601\_IMMATURE\_CD4\_SINGLE\_POSITIVE\_VS\_DOUBLE\_POSITIVE\_THYMOCYTE\_DN | 170 | 14 | 12187 | 179 | Parp14,Tor3a,Nfe2l2,Gbp4,Gbp6,Irgm2,Tnfaip8,Mxd4,Irf9,Tap1,Plek,Il17ra,Trim25,Icam1 | | 1.986e-07 | -15.43 | LI\_INDUCED\_T\_TO\_NATURAL\_KILLER\_UP | MSigDB lists | LI\_INDUCED\_T\_TO\_NATURAL\_KILLER\_UP | 225 | 16 | 12187 | 179 | Errfi1,Ifitm3,Ifitm2,Ier3,Nfkb1,Stx11,Samd9l,Gadd45g,Cdkn1a,Sgk1,Tnfsf10,AA467197,Tubb6,Ccl4,Ccnd2,Tnf | | 2.007e-07 | -15.42 | HIRSCH\_CELLULAR\_TRANSFORMATION\_SIGNATURE\_UP | MSigDB lists | HIRSCH\_CELLULAR\_TRANSFORMATION\_SIGNATURE\_UP | 197 | 15 | 12187 | 179 | Casp4,Il6,Bcl3,Irf7,Csf3,Sbno2,Tnfaip8,Osmr,Socs3,Timp1,Il1a,Plaur,Irf2,Irf9,Tap1 | | 2.007e-07 | -15.42 | BIOCARTA\_IL1R\_PATHWAY | MSigDB lists | BIOCARTA\_IL1R\_PATHWAY | 30 | 7 | 12187 | 179 | Irak3,Tnf,Nfkbia,Il1rn,Nfkb1,Il1a,Il6 | | 2.132e-07 | -15.36 | cell surface receptor signaling pathway | biological process | GO:0007166 | 1296 | 45 | 13711 | 214 | Plek,Cxcl9,Ccl2,Irak3,Il1rn,Arid5b,C3ar1,Lcn2,Nfkbiz,Ptpn1,Ifitm2,Iqgap1,Col4a1,Ccl12,Fyb,Ifitm3,Iigp1,P2ry6,Osmr,Kcna5,Plaur,Tnf,Hspb1,Cd14,Ripk1,Cxcl1,C5ar1,Hp,Csf2rb,Il4ra,Il1a,Nfkbia,Il6,Socs3,Irf7,Dtx3l,Ccl7,Pik3r1,Hcar2,Tiparp,Fzd4,Isg15,Ifitm6,Ccl4,Spi1 | | 2.180e-07 | -15.34 | P53\_DN.V2\_UP | MSigDB lists | P53\_DN.V2\_UP | 79 | 10 | 12187 | 179 | Angptl4,Cxcl1,P2ry6,Nfkbia,Sdc4,Xaf1,Ccl4,Ier3,Tnfaip3,Il1rn | | 2.185e-07 | -15.34 | regulation of cell migration | biological process | GO:0030334 | 753 | 32 | 13711 | 214 | Rhoc,Adamts9,Sele,Hspb1,C3ar1,Il1rn,Ccl2,Robo4,Selp,Msn,Nfe2l2,Ccl12,Cxcl16,Iqgap1,Clic4,Hspa5,C5ar1,Lgals9,P2ry6,Pik3r1,Trib1,Thbs1,Il1a,Akap12,Icam1,Ptgs2,Ccl4,Sdc4,Adamts1,Rhoj,Fam107a,Timp1 | | 2.221e-07 | -15.32 | GSE360\_T\_GONDII\_VS\_B\_MALAYI\_HIGH\_DOSE\_DC\_UP | MSigDB lists | GSE360\_T\_GONDII\_VS\_B\_MALAYI\_HIGH\_DOSE\_DC\_UP | 146 | 13 | 12187 | 179 | Nfkb2,Cflar,Gadd45b,Tnfaip3,Nfkbia,Tap1,Ifitm3,Tagln2,Il1a,Nfkb1,Stx11,Ifit3b,Socs3 | | 2.221e-07 | -15.32 | GSE360\_L\_DONOVANI\_VS\_M\_TUBERCULOSIS\_MAC\_DN | MSigDB lists | GSE360\_L\_DONOVANI\_VS\_M\_TUBERCULOSIS\_MAC\_DN | 146 | 13 | 12187 | 179 | Il6,Tnfaip3,Casp4,Tnf,Zfp189,Cdkn1a,Pygm,Sbno2,Nfkb2,Zfp36,Ptges,Ptgs2,Cflar | | 2.233e-07 | -15.31 | negative regulation of cellular metabolic process | biological process | GO:0031324 | 2049 | 61 | 13711 | 214 | Ptpn1,A2m,Iqgap1,Wfdc21,Bcl3,Plek,Tnfaip3,Znfx1,Xdh,Tnfaip8,Zbtb16,Arid5b,Irak3,Ifi204,Birc3,Gbp4,Nfkbie,Nfkb1,Bach1,Plaur,Sox11,Mxd4,Errfi1,Thbs1,Gadd45g,Rrp8,Dnajb1,Prg4,Hp,Rasd1,Serpina3f,Noct,Tnf,Zfp36,Atf3,Cflar,Hspb1,Ifi207,Trim30a,Tnip1,Ifi209,Timp1,Sbno2,Isg15,Parp9,Spi1,Cebpd,Rasip1,Ifi211,Ier3,Ptgs2,Runx1,Cdkn1a,Il6,Ctla2a,Parp14,Trib1,Socs3,Dtx3l,Pik3r1,Gadd45b | | 2.298e-07 | -15.29 | GO\_REGULATION\_OF\_PEPTIDASE\_ACTIVITY | MSigDB lists | GO\_REGULATION\_OF\_PEPTIDASE\_ACTIVITY | 257 | 17 | 12187 | 179 | Plaur,Thbs1,Ifi209,Ifi207,Timp1,Ifi204,S100a9,A2m,Ifi211,Tnfsf10,Xdh,S100a8,Tnfaip8,Birc3,Tnf,Il6,Ripk1 | | 2.301e-07 | -15.28 | TAKEDA\_TARGETS\_OF\_NUP98\_HOXA9\_FUSION\_8D\_UP | MSigDB lists | TAKEDA\_TARGETS\_OF\_NUP98\_HOXA9\_FUSION\_8D\_UP | 100 | 11 | 12187 | 179 | Irf7,Xaf1,Usp18,Ifit2,Ptgs2,Herc6,Rnf213,Isg15,Ifit3b,Thbs1,Ifi44 | | 2.361e-07 | -15.26 | positive regulation of multi-organism process | biological process | GO:0043902 | 270 | 18 | 13711 | 214 | Parp9,Tnip3,Ifi209,Cxcl1,Irgm2,Irgm1,Ifi211,Pik3ap1,Nfkbia,Acod1,Tnf,Trim30a,Lgals9,Ifi204,Tnip1,Cd14,Irf7,Irak3 | | 2.380e-07 | -15.25 | response to interleukin-1 | biological process | GO:0070555 | 75 | 10 | 13711 | 214 | Nfkb1,Il6,Ccl2,Ccl12,Acod1,Il1a,Sele,Irak3,Ccl4,Ccl7 | | 2.405e-07 | -15.24 | GSE30971\_CTRL\_VS\_LPS\_STIM\_MACROPHAGE\_WBP7\_KO\_4H\_UP | MSigDB lists | GSE30971\_CTRL\_VS\_LPS\_STIM\_MACROPHAGE\_WBP7\_KO\_4H\_UP | 147 | 13 | 12187 | 179 | Csf3,Rgs16,Tnf,Nfkbia,Il6,Gadd45b,Ptgs2,Pik3ap1,Irak3,Socs3,Il1a,Tnip3,Ier3 | | 2.405e-07 | -15.24 | GSE360\_L\_MAJOR\_VS\_B\_MALAYI\_HIGH\_DOSE\_DC\_UP | MSigDB lists | GSE360\_L\_MAJOR\_VS\_B\_MALAYI\_HIGH\_DOSE\_DC\_UP | 147 | 13 | 12187 | 179 | Tnfaip3,Nfkbia,Spi1,Tnfaip8,Rgs16,Cflar,Gadd45g,Il1rn,Ier3,Cdkn1a,Vcam1,Icam1,Plaur | | 2.463e-07 | -15.22 | NFKB\_Q6\_01 | MSigDB lists | NFKB\_Q6\_01 | 173 | 14 | 12187 | 179 | Vcam1,Luc7l3,Tnip3,Il1rn,Tnip1,Icam1,Nfkbia,Birc3,Gadd45b,Rrp8,Bcl3,Ptges,Csf3,Nfkb2 | | 2.550e-07 | -15.18 | AZARE\_NEOPLASTIC\_TRANSFORMATION\_BY\_STAT3\_UP | MSigDB lists | AZARE\_NEOPLASTIC\_TRANSFORMATION\_BY\_STAT3\_UP | 101 | 11 | 12187 | 179 | Ptgs2,Csf3,Ifi211,S100a9,S100a8,Clic4,Ccnd2,Ifi209,Thbs1,Ifi204,Ifi207 | | 2.603e-07 | -15.16 | GSE17721\_LPS\_VS\_GARDIQUIMOD\_1H\_BMDC\_UP | MSigDB lists | GSE17721\_LPS\_VS\_GARDIQUIMOD\_1H\_BMDC\_UP | 148 | 13 | 12187 | 179 | Sele,Slfn3,Timp1,Atf3,Nfkbiz,Rtp4,Slfn4,Arid5b,Cxcl1,Gadd45b,Il6,Tnf,Ccl2 | | 2.603e-07 | -15.16 | GSE21774\_CD62L\_POS\_CD56\_BRIGHT\_VS\_CD62L\_NEG\_CD56\_DIM\_NK\_CELL\_UP | MSigDB lists | GSE21774\_CD62L\_POS\_CD56\_BRIGHT\_VS\_CD62L\_NEG\_CD56\_DIM\_NK\_CELL\_UP | 148 | 13 | 12187 | 179 | Arid5b,Ncf1,Cd14,Igsf6,P2ry6,Pik3ap1,Tnf,Spi1,Cybb,Tubb6,Timp1,Rassf4,Lcn2 | | 2.645e-07 | -15.15 | GSE37416\_0H\_VS\_3H\_F\_TULARENSIS\_LVS\_NEUTROPHIL\_DN | MSigDB lists | GSE37416\_0H\_VS\_3H\_F\_TULARENSIS\_LVS\_NEUTROPHIL\_DN | 174 | 14 | 12187 | 179 | Tiparp,Stx11,Cdkn1a,Plaur,Cxcl16,Nfe2l2,Nfkbia,Tnf,Map3k8,Il4ra,Maff,Ptges,Pik3r5,Nfkbie | | 2.720e-07 | -15.12 | GO\_POSITIVE\_REGULATION\_OF\_CELL\_COMMUNICATION | MSigDB lists | GO\_POSITIVE\_REGULATION\_OF\_CELL\_COMMUNICATION | 1170 | 40 | 12187 | 179 | Map3k6,Selp,Pik3ap1,Ptpn1,Thbs1,Sox11,Akap12,Il1a,Gadd45g,Nfkb1,Csf3,Pik3r5,S100a8,Pik3r1,Map3k8,Ccl2,Ccl4,Hcar2,Rhoc,Gadd45b,Il6,Ccl12,Ripk1,Ptgs2,Saa1,Trim25,Icam1,Plaur,Socs3,Ksr1,Atf3,Cflar,C5ar1,Fzd4,Irf7,S100a9,Tnfsf10,Xdh,Birc3,Tnf | | 2.816e-07 | -15.08 | GSE19888\_ADENOSINE\_A3R\_INH\_VS\_ACT\_IN\_MAST\_CELL\_DN | MSigDB lists | GSE19888\_ADENOSINE\_A3R\_INH\_VS\_ACT\_IN\_MAST\_CELL\_DN | 149 | 13 | 12187 | 179 | Ifit3b,Znfx1,Cp,Ifitm3,Rnf213,Parp14,Ifit2,Lgals9,Gbp4,Gbp7,Gbp6,Gbp3,Xdh | | 2.822e-07 | -15.08 | GO\_REGULATION\_OF\_RESPONSE\_TO\_CYTOKINE\_STIMULUS | MSigDB lists | GO\_REGULATION\_OF\_RESPONSE\_TO\_CYTOKINE\_STIMULUS | 102 | 11 | 12187 | 179 | Socs3,Il1rn,Parp9,Ptpn1,Irak3,Usp18,Birc3,Tnf,Ripk1,Tnfaip3,Irf7 | | 2.822e-07 | -15.08 | GSE2706\_R848\_VS\_LPS\_2H\_STIM\_DC\_DN | MSigDB lists | GSE2706\_R848\_VS\_LPS\_2H\_STIM\_DC\_DN | 102 | 11 | 12187 | 179 | Ifit2,Ifi44,Ifit3b,Tnip3,Il1a,Znfx1,Oasl1,Ptgs2,Herc6,Isg15,Pik3ap1 | | 2.838e-07 | -15.07 | VERHAAK\_GLIOBLASTOMA\_MESENCHYMAL | MSigDB lists | VERHAAK\_GLIOBLASTOMA\_MESENCHYMAL | 175 | 14 | 12187 | 179 | Cd14,Rbm47,Plaur,Phf11b,Thbs1,Timp1,C5ar1,Phf11d,Iqgap1,Tnfaip8,Il4ra,Msr1,Casp4,Tnfaip3 | | 2.838e-07 | -15.07 | GO\_POSITIVE\_REGULATION\_OF\_SEQUENCE\_SPECIFIC\_DNA\_BINDING\_TRANSCRIPTION\_FACTOR\_ACTIVITY | MSigDB lists | GO\_POSITIVE\_REGULATION\_OF\_SEQUENCE\_SPECIFIC\_DNA\_BINDING\_TRANSCRIPTION\_FACTOR\_ACTIVITY | 175 | 14 | 12187 | 179 | Cflar,S100a9,Fzd4,Arid5b,S100a8,Nfkb2,Tnf,Nfkbia,Il6,Ripk1,Irak3,Icam1,Trim25,Nfkb1 | | 2.911e-07 | -15.05 | Measles | KEGG pathways | mmu05162 | 93 | 12 | 5248 | 107 | Pik3r1,Ccnd2,Nfkbia,Il1a,Tnfaip3,Tnfsf10,Irf9,Irf7,Msn,Nfkb1,Il6,Mx2 | | 2.911e-07 | -15.05 | Measles | KEGG pathways | ko05162 | 93 | 12 | 5248 | 107 | Mx2,Il6,Msn,Nfkb1,Tnfsf10,Irf9,Irf7,Tnfaip3,Il1a,Nfkbia,Ccnd2,Pik3r1 | | 2.933e-07 | -15.04 | KRIGE\_RESPONSE\_TO\_TOSEDOSTAT\_24HR\_UP | MSigDB lists | KRIGE\_RESPONSE\_TO\_TOSEDOSTAT\_24HR\_UP | 614 | 27 | 12187 | 179 | Ifi211,Phf11d,Bcl3,Rgs16,S100a8,Gpr84,Maff,Ccl12,Cybb,Slfn9,Tap1,Phf11b,S100a9,Rbm39,Errfi1,Clic4,Apold1,Plek,Ifit3b,Ifi209,Cdkn1a,Atf3,Ifi204,Il1rn,Ksr1,Ifi207,Samd9l | | 2.964e-07 | -15.03 | NFKB\_C | MSigDB lists | NFKB\_C | 203 | 15 | 12187 | 179 | Icam1,Ptgs2,Tnip1,Tnip3,Il1rn,Il1a,Luc7l3,Lcn2,Vcam1,Nfkb2,Ptges,Bcl3,Gadd45b,Map3k8,Nfkbia | | 2.969e-07 | -15.03 | cellular response to interleukin-1 | biological process | GO:0071347 | 59 | 9 | 13711 | 214 | Ccl2,Ccl12,Nfkb1,Il6,Il1a,Acod1,Ccl4,Ccl7,Irak3 | | 3.002e-07 | -15.02 | DASU\_IL6\_SIGNALING\_UP | MSigDB lists | DASU\_IL6\_SIGNALING\_UP | 46 | 8 | 12187 | 179 | Ccl12,Ier3,Il6,Maff,Irf9,Zfp36,Ptges,Gem | | 3.002e-07 | -15.02 | GO\_GRANULOCYTE\_MIGRATION | MSigDB lists | GO\_GRANULOCYTE\_MIGRATION | 46 | 8 | 12187 | 179 | S100a8,Il17ra,S100a9,Saa1,C5ar1,Ccl12,Ccl2,Ccl4 | | 3.017e-07 | -15.01 | GESERICK\_TERT\_TARGETS\_DN | MSigDB lists | GESERICK\_TERT\_TARGETS\_DN | 20 | 6 | 12187 | 179 | Nfkbiz,Gadd45b,Ier3,Gadd45g,Errfi1,Zfp36 | | 3.069e-07 | -15.00 | positive regulation of cell migration | biological process | GO:0030335 | 468 | 24 | 13711 | 214 | Ccl2,C3ar1,Sele,Hspb1,Rhoc,C5ar1,Hspa5,Iqgap1,Cxcl16,Ccl12,Nfe2l2,Selp,Icam1,Akap12,Il1a,Thbs1,Pik3r1,P2ry6,Lgals9,Fam107a,Rhoj,Adamts1,Ccl4,Ptgs2 | | 3.234e-07 | -14.94 | mouse chr11|11 B1.2 | chromosome location | mouse chr11|11 B1.2 | 11 | 5 | 14556 | 220 | 9930111J21Rik2,Tgtp1,Irgm1,Gm5431,Ifi47 | | 3.264e-07 | -14.94 | GSE43863\_TH1\_VS\_LY6C\_INT\_CXCR5POS\_MEMORY\_CD4\_TCELL\_UP | MSigDB lists | GSE43863\_TH1\_VS\_LY6C\_INT\_CXCR5POS\_MEMORY\_CD4\_TCELL\_UP | 177 | 14 | 12187 | 179 | Socs3,Atf3,Ksr1,Ncf1,Tap1,Icam1,Birc3,AA467197,Gbp4,Il6,Cflar,Irgm2,Gbp6,Nfkb2 | | 3.276e-07 | -14.93 | Toll-like receptor signaling pathway | KEGG pathways | mmu04620 | 77 | 11 | 5248 | 107 | Cd14,Ccl4,Cxcl9,Ripk1,Irf7,Pik3r1,Map3k8,Tnf,Nfkb1,Il6,Nfkbia | | 3.276e-07 | -14.93 | Toll-like receptor signaling pathway | KEGG pathways | ko04620 | 77 | 11 | 5248 | 107 | Ccl4,Cd14,Map3k8,Pik3r1,Nfkbia,Tnf,Irf7,Cxcl9,Ripk1,Il6,Nfkb1 | | 3.289e-07 | -14.93 | GSE41867\_MEMORY\_VS\_EXHAUSTED\_CD8\_TCELL\_DAY30\_LCMV\_UP | MSigDB lists | GSE41867\_MEMORY\_VS\_EXHAUSTED\_CD8\_TCELL\_DAY30\_LCMV\_UP | 151 | 13 | 12187 | 179 | Sgk3,Icam1,Plek,Ms4a6d,Cebpd,Gbp7,Gbp3,Ifit2,Lgals9,Parp14,Tor3a,Spi1,Cybb | | 3.325e-07 | -14.92 | regulation of locomotion | biological process | GO:0040012 | 847 | 34 | 13711 | 214 | P2ry6,Lgals9,Pik3r1,Trib1,Thbs1,Akap12,Il1a,Icam1,Trim25,Trim56,Ptgs2,Ccl4,Adamts1,Sdc4,Rhoj,Fam107a,Timp1,Rhoc,Adamts9,Il1rn,C3ar1,Hspb1,Sele,Ccl2,Robo4,Msn,Selp,Nfe2l2,Cxcl16,Ccl12,Iqgap1,Clic4,Hspa5,C5ar1 | | 3.326e-07 | -14.92 | GO\_ACTIVATION\_OF\_IMMUNE\_RESPONSE | MSigDB lists | GO\_ACTIVATION\_OF\_IMMUNE\_RESPONSE | 295 | 18 | 12187 | 179 | Tnfaip3,Ripk1,Pik3r1,Birc3,Nfkbia,C5ar1,C3ar1,Ifi211,Irf7,Tnip3,Nfkb1,Ifi204,Ifi207,Ifi209,Irak3,Pik3ap1,Tnip1,Cd14 | | 3.377e-07 | -14.90 | negative regulation of macromolecule metabolic process | biological process | GO:0010605 | 2073 | 61 | 13711 | 214 | Socs3,Dtx3l,Ctla2a,Parp14,Trib1,Pik3r1,Gadd45b,Il6,Cdkn1a,Rasip1,Cebpd,Ifi211,Spi1,Ptgs2,Runx1,Timp1,Ifi209,Tiparp,Isg15,Sbno2,Parp9,Hspb1,Ifi207,Cflar,Tnip1,Tnf,Atf3,Zfp36,Serpina3f,Mmp8,Rasd1,Noct,Rrp8,Dnajb1,Prg4,Thbs1,Msr1,Banp,Gadd45g,Lgals9,Mxd4,Sox11,Errfi1,Bach1,Plaur,Gbp4,Birc3,Nfkb1,Nfkbie,Arid5b,Irak3,Zbtb16,Tnfaip8,Ifi204,Tnfaip3,Znfx1,Xdh,Bcl3,Wfdc21,Ptpn1,A2m | | 3.418e-07 | -14.89 | negative regulation of cellular protein metabolic process | biological process | GO:0032269 | 848 | 34 | 13711 | 214 | Serpina3f,Wfdc21,Ptpn1,A2m,Hspb1,Irak3,Cflar,Tnfaip8,Tnip1,Tnf,Tnfaip3,Atf3,Zfp36,Xdh,Rasip1,Spi1,Ptgs2,Plaur,Gbp4,Timp1,Birc3,Isg15,Socs3,Thbs1,Dtx3l,Trib1,Parp14,Ctla2a,Gadd45g,Pik3r1,Gadd45b,Il6,Errfi1,Cdkn1a | | 3.467e-07 | -14.87 | extracellular membrane-bounded organelle | cellular component | GO:0065010 | 11 | 5 | 13825 | 212 | Irgm2,Gbp6,Iigp1,Gbp3,Gbp7 | | 3.500e-07 | -14.87 | GO\_ACTIVATION\_OF\_CYSTEINE\_TYPE\_ENDOPEPTIDASE\_ACTIVITY | MSigDB lists | GO\_ACTIVATION\_OF\_CYSTEINE\_TYPE\_ENDOPEPTIDASE\_ACTIVITY | 83 | 10 | 12187 | 179 | Ifi211,S100a9,Xdh,Tnfsf10,S100a8,Ifi209,Tnf,Ripk1,Ifi204,Ifi207 | | 3.538e-07 | -14.85 | negative regulation of cytokine production | biological process | GO:0001818 | 193 | 15 | 13711 | 214 | Mmp8,Bcl3,Gbp4,Prg4,Nfkb1,Irak3,Thbs1,Lgals9,Trim30a,Tnf,Tnfaip3,Acod1,Zfp36,Errfi1,Il6 | | 3.551e-07 | -14.85 | GSE29617\_CTRL\_VS\_DAY7\_TIV\_FLU\_VACCINE\_PBMC\_2008\_UP | MSigDB lists | GSE29617\_CTRL\_VS\_DAY7\_TIV\_FLU\_VACCINE\_PBMC\_2008\_UP | 152 | 13 | 12187 | 179 | Bach1,Nfe2l2,Ifit2,C5ar1,Cxcl1,Bcl3,Tiparp,Stx11,Atf3,Cdkn1a,Sgk1,Socs3,Ptgs2 | | 3.572e-07 | -14.84 | NAGASHIMA\_EGF\_SIGNALING\_UP | MSigDB lists | NAGASHIMA\_EGF\_SIGNALING\_UP | 47 | 8 | 12187 | 179 | Atf3,Ier3,Tiparp,Trib1,Dnajb1,Maff,Zfp36,Gem | | 3.578e-07 | -14.84 | GO\_MYELOID\_LEUKOCYTE\_MIGRATION | MSigDB lists | GO\_MYELOID\_LEUKOCYTE\_MIGRATION | 64 | 9 | 12187 | 179 | Ccl4,Ccl2,S100a9,C5ar1,Saa1,Ccl12,Il6,Il17ra,S100a8 | | 3.642e-07 | -14.83 | immune response-activating signal transduction | biological process | GO:0002757 | 143 | 13 | 13711 | 214 | Cd14,Irak3,C3ar1,Lgals9,Tnip1,Nfkbia,Fyb,Pik3ap1,Tnf,Irgm2,C5ar1,Nfkbiz,Tnip3 | | 3.692e-07 | -14.81 | AGE-RAGE signaling pathway in diabetic complications | KEGG pathways | ko04933 | 95 | 12 | 5248 | 107 | Cybb,Icam1,Tnf,Vcam1,Il6,Nfkb1,Il1a,Ccl12,Col4a1,Ccl2,Pik3r1,Sele | | 3.692e-07 | -14.81 | AGE-RAGE signaling pathway in diabetic complications | KEGG pathways | mmu04933 | 95 | 12 | 5248 | 107 | Cybb,Vcam1,Tnf,Icam1,Nfkb1,Il6,Ccl12,Il1a,Pik3r1,Ccl2,Col4a1,Sele | | 3.747e-07 | -14.80 | GSE2770\_TGFB\_AND\_IL4\_VS\_IL12\_TREATED\_ACT\_CD4\_TCELL\_6H\_UP | MSigDB lists | GSE2770\_TGFB\_AND\_IL4\_VS\_IL12\_TREATED\_ACT\_CD4\_TCELL\_6H\_UP | 179 | 14 | 12187 | 179 | Birc3,Parp14,Usp18,Gbp7,Iqgap1,Rtp4,Sbno2,Aff1,Samd9l,Atf3,Rnf213,Trim25,Parp9,Msn | | 3.747e-07 | -14.80 | GSE32034\_LY6C\_HIGH\_VS\_LOW\_MONOCYTE\_DN | MSigDB lists | GSE32034\_LY6C\_HIGH\_VS\_LOW\_MONOCYTE\_DN | 179 | 14 | 12187 | 179 | Ifit3b,Irf9,Parp9,Slfn9,Tnip1,Parp12,Gbp4,Usp18,Parp14,Ifit2,Xdh,Cmpk2,Rtp4,Gbp6 | | 3.764e-07 | -14.79 | GSE7509\_UNSTIM\_VS\_FCGRIIB\_STIM\_DC\_DN | MSigDB lists | GSE7509\_UNSTIM\_VS\_FCGRIIB\_STIM\_DC\_DN | 128 | 12 | 12187 | 179 | Clic4,Icam1,Isg15,Nfkbia,Usp18,Casp4,Xaf1,Ifit2,Rgs16,Tnfsf10,Sbno2,Angptl4 | | 3.799e-07 | -14.78 | GGGNNTTTCC\_NFKB\_Q6\_01 | MSigDB lists | GGGNNTTTCC\_NFKB\_Q6\_01 | 105 | 11 | 12187 | 179 | Il6,Ccnd2,Birc3,Nfkbia,Nfkb2,Gem,Ptges,Bcl3,Il1rn,Csf2rb,Tnip1 | | 3.832e-07 | -14.77 | GSE41867\_DAY6\_EFFECTOR\_VS\_DAY30\_EXHAUSTED\_CD8\_TCELL\_LCMV\_CLONE13\_UP | MSigDB lists | GSE41867\_DAY6\_EFFECTOR\_VS\_DAY30\_EXHAUSTED\_CD8\_TCELL\_LCMV\_CLONE13\_UP | 153 | 13 | 12187 | 179 | Tor3a,Parp14,Lgals9,Parp12,Znfx1,Irf7,Dtx3l,Irgm2,Ifitm3,Gbp3,Xdh,Slfn3,Slfn4 | | 4.055e-07 | -14.72 | negative regulation of viral genome replication | biological process | GO:0045071 | 45 | 8 | 13711 | 214 | Ifitm2,Tnf,Oasl2,Isg15,Oasl1,Ifitm6,Ifitm3,Resf1 | | 4.058e-07 | -14.72 | apoptotic process | biological process | GO:0006915 | 696 | 30 | 13711 | 214 | C5ar1,Rrp8,S100a8,S100a9,Ifit2,Bcl3,Tnfaip3,Tnf,Tnfsf10,Tnfaip8,Ripk1,Cflar,Irak3,Ncf1,Ifi204,Lcn2,Birc3,Nfkb1,Spi1,Xaf1,Ier3,Sgk1,Il1a,Casp4,Cdkn1a,Il6,Gadd45b,Pik3r1,Hcar2,Gadd45g | | 4.101e-07 | -14.71 | GO\_POSITIVE\_REGULATION\_OF\_REACTIVE\_OXYGEN\_SPECIES\_METABOLIC\_PROCESS | MSigDB lists | GO\_POSITIVE\_REGULATION\_OF\_REACTIVE\_OXYGEN\_SPECIES\_METABOLIC\_PROCESS | 65 | 9 | 12187 | 179 | Ripk1,Nfe2l2,Il6,Cdkn1a,Tnf,Thbs1,Icam1,Xdh,Ptgs2 | | 4.132e-07 | -14.70 | GO\_REGULATION\_OF\_SYMBIOSIS\_ENCOMPASSING\_MUTUALISM\_THROUGH\_PARASITISM | MSigDB lists | GO\_REGULATION\_OF\_SYMBIOSIS\_ENCOMPASSING\_MUTUALISM\_THROUGH\_PARASITISM | 154 | 13 | 12187 | 179 | Ifi211,Tnf,Ccl4,Oasl1,Ifitm3,Ifitm2,Isg15,Tnip1,Trim25,Tap1,Ifi209,Ifi204,Ifi207 | | 4.172e-07 | -14.69 | BIOCARTA\_NFKB\_PATHWAY | MSigDB lists | BIOCARTA\_NFKB\_PATHWAY | 21 | 6 | 12187 | 179 | Ripk1,Tnfaip3,Il1a,Nfkb1,Nfkbia,Tnf | | 4.292e-07 | -14.66 | NFKAPPAB65\_01 | MSigDB lists | NFKAPPAB65\_01 | 181 | 14 | 12187 | 179 | Csf2rb,Cxcl16,Icam1,Tnip1,Ier3,Nfkb2,Bcl3,Rrp8,Ptges,Gadd45b,Nfkbia,Sdc4,Birc3,Map3k8 | | 4.352e-07 | -14.65 | response to type I interferon | biological process | GO:0034340 | 20 | 6 | 13711 | 214 | Trim56,Isg15,Ifitm6,Irf7,Ifitm3,Ifitm2 | | 4.453e-07 | -14.62 | GSE33162\_HDAC3\_KO\_VS\_HDAC3\_KO\_MACROPHAGE\_UP | MSigDB lists | GSE33162\_HDAC3\_KO\_VS\_HDAC3\_KO\_MACROPHAGE\_UP | 155 | 13 | 12187 | 179 | Gbp4,Parp12,Il4ra,Parp14,Usp18,Olfml2b,Rtp4,Gbp6,Ptges,Ifit3b,Parp9,Tap1,Irf9 | | 4.677e-07 | -14.58 | positive regulation of cell motility | biological process | GO:2000147 | 479 | 24 | 13711 | 214 | Icam1,Akap12,Il1a,Thbs1,Pik3r1,Lgals9,P2ry6,Fam107a,Rhoj,Adamts1,Ccl4,Ptgs2,Ccl2,Hspb1,Sele,C3ar1,Rhoc,C5ar1,Hspa5,Iqgap1,Ccl12,Cxcl16,Nfe2l2,Selp | | 4.689e-07 | -14.57 | GNF2\_HCK | MSigDB lists | GNF2\_HCK | 66 | 9 | 12187 | 179 | Spi1,Stx11,Cybb,Ms4a6d,C5ar1,Cd14,S100a9,Igsf6,Tnfsf10 | | 4.796e-07 | -14.55 | GSE45365\_NK\_CELL\_VS\_BCELL\_MCMV\_INFECTION\_DN | MSigDB lists | GSE45365\_NK\_CELL\_VS\_BCELL\_MCMV\_INFECTION\_DN | 156 | 13 | 12187 | 179 | Il4ra,Sdc4,Nfkbia,Rassf4,Tnfaip3,Gadd45b,Sbno2,Fstl1,Nfkb1,Tnip1,Plek,Ptpn1,Tap1 | | 4.845e-07 | -14.54 | GSE2128\_CTRL\_VS\_MIMETOPE\_NEGATIVE\_SELECTION\_DP\_THYMOCYTE\_C57BL6\_UP | MSigDB lists | GSE2128\_CTRL\_VS\_MIMETOPE\_NEGATIVE\_SELECTION\_DP\_THYMOCYTE\_C57BL6\_UP | 131 | 12 | 12187 | 179 | Ifi204,Nfkb1,Ifi207,Cdkn1a,Ifi209,Thbs1,Icam1,Cybb,Gadd45b,Tnf,S100a8,Ifi211 | | 4.905e-07 | -14.53 | GO\_PATTERN\_RECOGNITION\_RECEPTOR\_SIGNALING\_PATHWAY | MSigDB lists | GO\_PATTERN\_RECOGNITION\_RECEPTOR\_SIGNALING\_PATHWAY | 86 | 10 | 12187 | 179 | Nfkbia,Birc3,Ripk1,Tnfaip3,Tnip3,Tnip1,Irf7,Cd14,Irak3,Pik3ap1 | | 5.013e-07 | -14.51 | GO\_POSITIVE\_REGULATION\_OF\_BIOSYNTHETIC\_PROCESS | MSigDB lists | GO\_POSITIVE\_REGULATION\_OF\_BIOSYNTHETIC\_PROCESS | 1340 | 43 | 12187 | 179 | Zbtb16,Ifi207,Atf3,Ptgs2,Tnip1,Hspb1,Tnf,Nfkbia,C3ar1,Nfkb2,Thbs1,Nfkb1,Il1a,Akap12,Irak3,Ccl12,Il6,Nfe2l2,Csf3,Banp,Sbno2,Ifi209,Ifi204,Icam1,Trim25,Runx1,Bach1,Cflar,Irf7,S100a9,Fzd4,Sox11,Hspa5,Cebpd,Irf2,Maff,Pik3r1,Spi1,Ripk1,Ifi211,Bcl3,Arid5b,S100a8 | | 5.016e-07 | -14.51 | regulation of symbiosis, encompassing mutualism through parasitism | biological process | GO:0043903 | 172 | 14 | 13711 | 214 | Trim56,Trim25,Resf1,Ifitm6,Ccl4,Isg15,Cxcl1,Ifitm2,Trim30a,Oasl1,Ifitm3,Zfp36,Tnf,Oasl2 | | 5.047e-07 | -14.50 | MODULE\_312 | MSigDB lists | MODULE\_312 | 34 | 7 | 12187 | 179 | Ccnd2,Birc3,Ier3,Nfkb1,Il1a,Cflar,Tnfsf10 | | 5.047e-07 | -14.50 | DORN\_ADENOVIRUS\_INFECTION\_24HR\_DN | MSigDB lists | DORN\_ADENOVIRUS\_INFECTION\_24HR\_DN | 34 | 7 | 12187 | 179 | Nfkb2,Ier3,Il6,Nfe2l2,Ifit2,Sgk1,Nfkbia | | 5.047e-07 | -14.50 | MANTOVANI\_NFKB\_TARGETS\_UP | MSigDB lists | MANTOVANI\_NFKB\_TARGETS\_UP | 34 | 7 | 12187 | 179 | Vcam1,Casp4,Nfkbiz,Cp,Ccl12,Saa1,Osmr | | 5.047e-07 | -14.50 | GEISS\_RESPONSE\_TO\_DSRNA\_UP | MSigDB lists | GEISS\_RESPONSE\_TO\_DSRNA\_UP | 34 | 7 | 12187 | 179 | Cflar,Ccl4,Nfkbia,Tnfaip3,Plaur,Tap1,Atf3 | | 5.246e-07 | -14.46 | GO\_POSITIVE\_REGULATION\_OF\_PHOSPHORUS\_METABOLIC\_PROCESS | MSigDB lists | GO\_POSITIVE\_REGULATION\_OF\_PHOSPHORUS\_METABOLIC\_PROCESS | 755 | 30 | 12187 | 179 | Sdc4,Tnf,Xdh,Fzd4,C5ar1,Ksr1,Socs3,Cdkn1a,Plaur,Icam1,Plek,Saa1,Ccl12,Ripk1,Gadd45b,Il6,Ccl4,Ccl2,Map3k8,Ccnd2,Iqgap1,Pik3r5,Csf3,Akap12,Gadd45g,Il1a,Thbs1,Hspa5,Ptpn1,Map3k6 | | 5.348e-07 | -14.44 | GHANDHI\_DIRECT\_IRRADIATION\_UP | MSigDB lists | GHANDHI\_DIRECT\_IRRADIATION\_UP | 67 | 9 | 12187 | 179 | Il1a,Icam1,Il6,Tnfaip3,Birc3,Ptgs2,Cdkn1a,Nfkbiz,Cxcl1 | | 5.368e-07 | -14.44 | regulation of cell motility | biological process | GO:2000145 | 784 | 32 | 13711 | 214 | C3ar1,Il1rn,Hspb1,Sele,Adamts9,Rhoc,Robo4,Ccl2,Nfe2l2,Msn,Selp,C5ar1,Clic4,Hspa5,Iqgap1,Cxcl16,Ccl12,Thbs1,Trib1,Pik3r1,P2ry6,Lgals9,Icam1,Akap12,Il1a,Ccl4,Ptgs2,Timp1,Fam107a,Rhoj,Adamts1,Sdc4 | | 5.555e-07 | -14.40 | GSE360\_DC\_VS\_MAC\_B\_MALAYI\_HIGH\_DOSE\_DN | MSigDB lists | GSE360\_DC\_VS\_MAC\_B\_MALAYI\_HIGH\_DOSE\_DN | 158 | 13 | 12187 | 179 | S100a8,Rgs16,Ifi211,Rhoc,Maff,Plaur,Isg15,Cd14,Ifi204,Ifi207,Cebpd,Ifi209,Ifi44 | | 5.555e-07 | -14.40 | GSE5455\_EX\_VIVO\_VS\_POST\_24H\_INCUBATION\_MONOCYTES\_FROM\_TUMOR\_BEARING\_MOUSE\_DN | MSigDB lists | GSE5455\_EX\_VIVO\_VS\_POST\_24H\_INCUBATION\_MONOCYTES\_FROM\_TUMOR\_BEARING\_MOUSE\_DN | 158 | 13 | 12187 | 179 | Ifit3b,Sgk1,Samd9l,Slfn5,Rnf213,Irf9,Irak3,Xaf1,Usp18,Il4ra,Bcl3,Tnfsf10,Rtp4 | | 5.555e-07 | -14.40 | JISON\_SICKLE\_CELL\_DISEASE\_UP | MSigDB lists | JISON\_SICKLE\_CELL\_DISEASE\_UP | 158 | 13 | 12187 | 179 | Nfkbie,Selp,Tap1,Clic4,Plek,Ifit3b,Ifi44,Cdkn1a,Ifit2,Oasl1,Gadd45b,Atf3,Timp1 | | 5.667e-07 | -14.38 | FARMER\_BREAST\_CANCER\_CLUSTER\_1 | MSigDB lists | FARMER\_BREAST\_CANCER\_CLUSTER\_1 | 22 | 6 | 12187 | 179 | Isg15,Herc6,Ifi44,Xaf1,Ifit3b,Oasl1 | | 5.690e-07 | -14.38 | SWEET\_LUNG\_CANCER\_KRAS\_DN | MSigDB lists | SWEET\_LUNG\_CANCER\_KRAS\_DN | 339 | 19 | 12187 | 179 | Gbp7,S100a9,Rhoj,Ptges,S100a8,Gbp3,Nfkbia,Usp18,Gadd45b,Ifitm3,Clic4,Sox11,Ifit3b,Zbtb16,Ms4a6d,Akap12,Hp,Cp,Rasip1 | | 5.880e-07 | -14.35 | positive regulation of inflammatory response | biological process | GO:0050729 | 103 | 11 | 13711 | 214 | Tnip1,Ptgs2,Il17ra,Mmp8,S100a9,Il6,S100a8,Tgm2,Nfkbiz,Tnf,Nfkbia | | 5.973e-07 | -14.33 | GSE36009\_UNSTIM\_VS\_LPS\_STIM\_DC\_UP | MSigDB lists | GSE36009\_UNSTIM\_VS\_LPS\_STIM\_DC\_UP | 159 | 13 | 12187 | 179 | Atf3,Ksr1,Gadd45g,Ptpn1,Irf9,Tap1,Slfn9,Ifitm3,Hcar2,Parp14,Gbp3,Tnfsf10,Gbp7 | | 5.973e-07 | -14.33 | GSE360\_L\_DONOVANI\_VS\_B\_MALAYI\_LOW\_DOSE\_DC\_UP | MSigDB lists | GSE360\_L\_DONOVANI\_VS\_B\_MALAYI\_LOW\_DOSE\_DC\_UP | 159 | 13 | 12187 | 179 | Birc3,Gadd45b,Tnfaip3,Irf7,Gem,Nfkbie,Cflar,Ier3,Gadd45g,Nfkb1,Tnip1,Isg15,Csf2rb | | 6.014e-07 | -14.32 | regulation of tumor necrosis factor biosynthetic process | biological process | GO:0042534 | 21 | 6 | 13711 | 214 | Bcl3,Errfi1,Cybb,Akap12,Hspb1,Thbs1 | | 6.101e-07 | -14.31 | GO\_NEGATIVE\_REGULATION\_OF\_DEFENSE\_RESPONSE | MSigDB lists | GO\_NEGATIVE\_REGULATION\_OF\_DEFENSE\_RESPONSE | 110 | 11 | 12187 | 179 | Ifi211,A2m,Saa1,Tap1,Irak3,Socs3,Ifi209,Tnfaip3,Ifi204,Nfkb1,Ifi207 | | 6.232e-07 | -14.29 | GO\_REGULATION\_OF\_INTERLEUKIN\_1\_BETA\_PRODUCTION | MSigDB lists | GO\_REGULATION\_OF\_INTERLEUKIN\_1\_BETA\_PRODUCTION | 35 | 7 | 12187 | 179 | Ifi211,Hspb1,Errfi1,Ifi209,Ifi204,Ifi207,Tnfaip3 | | 6.232e-07 | -14.29 | GO\_CELLULAR\_RESPONSE\_TO\_GLUCOSE\_STARVATION | MSigDB lists | GO\_CELLULAR\_RESPONSE\_TO\_GLUCOSE\_STARVATION | 35 | 7 | 12187 | 179 | Nfe2l2,Ifi207,Ifi204,Rrp8,Ifi211,Hspa5,Ifi209 | | 6.328e-07 | -14.27 | regulation of viral process | biological process | GO:0050792 | 150 | 13 | 13711 | 214 | Resf1,Trim25,Trim56,Ccl4,Ifitm6,Isg15,Ifitm2,Trim30a,Oasl1,Ifitm3,Zfp36,Oasl2,Tnf | | 6.419e-07 | -14.26 | GSE24142\_EARLY\_THYMIC\_PROGENITOR\_VS\_DN2\_THYMOCYTE\_FETAL\_UP | MSigDB lists | GSE24142\_EARLY\_THYMIC\_PROGENITOR\_VS\_DN2\_THYMOCYTE\_FETAL\_UP | 160 | 13 | 12187 | 179 | Ier3,Mxd4,Hspa5,Fstl1,Plek,Icam1,Irf9,Birc3,Il4ra,Usp18,Tgm2,Pygm,Gem | | 6.626e-07 | -14.23 | GENTILE\_UV\_HIGH\_DOSE\_DN | MSigDB lists | GENTILE\_UV\_HIGH\_DOSE\_DN | 277 | 17 | 12187 | 179 | Zfp36,Ptpn1,Isg15,Tiparp,Cebpd,Atf3,Thbs1,Tnfaip8,Gem,Arid5b,Bach1,Ripk1,Tnfaip3,Nfe2l2,Runx1,Il4ra,Maff | | 6.781e-07 | -14.20 | KEGG\_JAK\_STAT\_SIGNALING\_PATHWAY | MSigDB lists | KEGG\_JAK\_STAT\_SIGNALING\_PATHWAY | 89 | 10 | 12187 | 179 | Il6,Socs3,Ccnd2,Pik3r1,Il4ra,Csf2rb,Irf9,Osmr,Pik3r5,Csf3 | | 6.781e-07 | -14.20 | GO\_NEGATIVE\_REGULATION\_OF\_IMMUNE\_RESPONSE | MSigDB lists | GO\_NEGATIVE\_REGULATION\_OF\_IMMUNE\_RESPONSE | 89 | 10 | 12187 | 179 | Tap1,Irak3,Ifi211,A2m,Tnfaip3,Ifi204,Ifi207,Ifi209,Tnf,Il4ra | | 6.781e-07 | -14.20 | GO\_ZYMOGEN\_ACTIVATION | MSigDB lists | GO\_ZYMOGEN\_ACTIVATION | 89 | 10 | 12187 | 179 | Ifi207,Ifi204,Ripk1,Tnf,Ifi209,S100a8,Tnfsf10,Xdh,S100a9,Ifi211 | | 6.894e-07 | -14.19 | GSE2770\_UNTREATED\_VS\_IL12\_TREATED\_ACT\_CD4\_TCELL\_6H\_UP | MSigDB lists | GSE2770\_UNTREATED\_VS\_IL12\_TREATED\_ACT\_CD4\_TCELL\_6H\_UP | 161 | 13 | 12187 | 179 | Aff1,Atf3,Tap1,Ptpn1,Zfp36,Icam1,Maff,AA467197,Gbp4,Trim56,Banp,Gbp6,Gbp7 | | 6.894e-07 | -14.19 | GSE14769\_UNSTIM\_VS\_20MIN\_LPS\_BMDM\_DN | MSigDB lists | GSE14769\_UNSTIM\_VS\_20MIN\_LPS\_BMDM\_DN | 161 | 13 | 12187 | 179 | Map3k8,Tnf,Usp18,Nfkbia,Ccl4,Sgk1,Nfkbiz,Socs3,Gadd45g,Ier3,Cd14,Zfp36,Parp9 | | 7.253e-07 | -14.14 | positive regulation of molecular function | biological process | GO:0044093 | 1399 | 46 | 13711 | 214 | Ncf1,Sele,Ripk1,Cflar,Rhoc,Tnf,Mmp8,Dnajb1,Dtx3l,Trib1,Ccl7,Gadd45b,Ccnd2,Casp4,Il6,Cdkn1a,Ccl4,Trim25,Fzd4,Map3k6,Parp9,Sdc4,Il1rn,Irak3,Arid5b,Csf3,Plek,Xdh,Tnfsf10,Ccl2,S100a9,Irgm2,Wfdc21,Ptpn1,S100a8,Iqgap1,Ccl12,Thbs1,Gadd45g,P2ry6,Lgals9,Icam1,Rgs16,Pik3r5,Plaur,Map3k8 | | 7.367e-07 | -14.12 | immune response-regulating signaling pathway | biological process | GO:0002764 | 152 | 13 | 13711 | 214 | Cd14,Irak3,C3ar1,Lgals9,Tnip1,Pik3ap1,Fyb,Nfkbia,Tnf,Irgm2,Nfkbiz,C5ar1,Tnip3 | | 7.400e-07 | -14.12 | GSE10325\_BCELL\_VS\_LUPUS\_BCELL\_DN | MSigDB lists | GSE10325\_BCELL\_VS\_LUPUS\_BCELL\_DN | 162 | 13 | 12187 | 179 | Tiparp,Ifi44,Ifit3b,Rbm47,Tap1,Herc6,Isg15,Oasl1,Xaf1,Usp18,Rtp4,Cflar,Irf7 | | 7.400e-07 | -14.12 | GSE23502\_WT\_VS\_HDC\_KO\_MYELOID\_DERIVED\_SUPPRESSOR\_CELL\_COLON\_TUMOR\_UP | MSigDB lists | GSE23502\_WT\_VS\_HDC\_KO\_MYELOID\_DERIVED\_SUPPRESSOR\_CELL\_COLON\_TUMOR\_UP | 162 | 13 | 12187 | 179 | Ifit3b,Vcam1,Ifitm2,Ifitm3,Tagln2,Tgm2,Gbp4,Gbp6,C5ar1,Fzd4,S100a9,S100a8,Igsf6 | | 7.425e-07 | -14.11 | GO\_RESPONSE\_TO\_EXTRACELLULAR\_STIMULUS | MSigDB lists | GO\_RESPONSE\_TO\_EXTRACELLULAR\_STIMULUS | 345 | 19 | 12187 | 179 | Ifi211,Rrp8,Ccl12,Il6,Nfe2l2,Ptgs2,Icam1,Zfp36,Adamts1,Cdkn1a,Ifi209,Hspa5,Vcam1,Fstl1,Socs3,Ifi204,Ifi207,Cp,Atf3 | | 7.533e-07 | -14.10 | TAKEDA\_TARGETS\_OF\_NUP98\_HOXA9\_FUSION\_10D\_DN | MSigDB lists | TAKEDA\_TARGETS\_OF\_NUP98\_HOXA9\_FUSION\_10D\_DN | 90 | 10 | 12187 | 179 | Olfml2b,A2m,Slfn9,Cxcl1,C5ar1,Rassf4,Mmp8,Ccl2,Ccl4,Sgk1 | | 7.563e-07 | -14.09 | XU\_HGF\_TARGETS\_INDUCED\_BY\_AKT1\_6HR | MSigDB lists | XU\_HGF\_TARGETS\_INDUCED\_BY\_AKT1\_6HR | 13 | 5 | 12187 | 179 | Plaur,Maff,Isg15,Thbs1,Irf7 | | 7.563e-07 | -14.09 | LEE\_LIVER\_CANCER\_HEPATOBLAST | MSigDB lists | LEE\_LIVER\_CANCER\_HEPATOBLAST | 13 | 5 | 12187 | 179 | Ptgs2,Ccnd2,Timp1,Il6,Plaur | | 7.641e-07 | -14.08 | VARELA\_ZMPSTE24\_TARGETS\_UP | MSigDB lists | VARELA\_ZMPSTE24\_TARGETS\_UP | 36 | 7 | 12187 | 179 | Cd14,Zbtb16,Cdkn1a,Gadd45b,Rgs16,Atf3,Gadd45g | | 7.833e-07 | -14.06 | GO\_TOLL\_LIKE\_RECEPTOR\_SIGNALING\_PATHWAY | MSigDB lists | GO\_TOLL\_LIKE\_RECEPTOR\_SIGNALING\_PATHWAY | 70 | 9 | 12187 | 179 | Tnip3,Ripk1,Birc3,Nfkbia,Pik3ap1,Irak3,Irf7,Cd14,Tnip1 | | 7.864e-07 | -14.06 | GSE30083\_SP1\_VS\_SP3\_THYMOCYTE\_DN | MSigDB lists | GSE30083\_SP1\_VS\_SP3\_THYMOCYTE\_DN | 137 | 12 | 12187 | 179 | Ms4a6d,Zbtb16,Ifi44,Sgk1,Il17ra,Slfn5,Herc6,Rnf213,Usp18,Iqgap1,Irf7,Irgm2 | | 7.864e-07 | -14.06 | GSE36888\_UNTREATED\_VS\_IL2\_TREATED\_STAT5\_AB\_KNOCKIN\_TCELL\_17H\_UP | MSigDB lists | GSE36888\_UNTREATED\_VS\_IL2\_TREATED\_STAT5\_AB\_KNOCKIN\_TCELL\_17H\_UP | 137 | 12 | 12187 | 179 | Tnfaip3,Tnip3,Slc24a4,Casp4,Vcam1,Runx1,Plaur,Arid5b,Cxcl1,Ptges,Ptgs2,C5ar1 | | 8.162e-07 | -14.02 | response to interferon-alpha | biological process | GO:0035455 | 22 | 6 | 13711 | 214 | Ifitm3,Ifitm6,Ifitm2,Tgtp1,Ifi204,Ifit2 | | 8.328e-07 | -14.00 | CHICAS\_RB1\_TARGETS\_CONFLUENT | MSigDB lists | CHICAS\_RB1\_TARGETS\_CONFLUENT | 454 | 22 | 12187 | 179 | Ifi209,Thbs1,Vcam1,Ifi204,Ifi207,Samd9l,Ifitm3,Ifitm2,Hspb1,Errfi1,Parp9,Ifit2,Xaf1,Tgm2,Sntb2,Sdc4,Tubb6,Rassf4,Dtx3l,Ifi211,Arid5b,Rhoj | | 8.371e-07 | -13.99 | regulation of cell population proliferation | biological process | GO:0042127 | 1270 | 43 | 13711 | 214 | Cflar,Ripk1,Zbtb16,Csf3,C3ar1,Tnfaip3,Tnf,Ccl2,Zfp36,Atf3,Ksr1,Xdh,Vcam1,Tgm2,Cxcl1,C5ar1,Ccl12,Prg4,Trib1,Ifitm3,Thbs1,Lgals9,P2ry6,Ccnd2,Pik3r1,Il1a,Sox11,Nfkbia,Il4ra,Slfn3,Cdkn1a,Il6,Errfi1,Kcna5,Rasip1,Ptgs2,Runx1,Osmr,Timp1,Ptges,Sdc4,Adamts1,Slfn2 | | 8.514e-07 | -13.98 | GSE19401\_UNSTIM\_VS\_RETINOIC\_ACID\_AND\_PAM2CSK4\_STIM\_FOLLICULAR\_DC\_DN | MSigDB lists | GSE19401\_UNSTIM\_VS\_RETINOIC\_ACID\_AND\_PAM2CSK4\_STIM\_FOLLICULAR\_DC\_DN | 164 | 13 | 12187 | 179 | Il6,Gadd45b,Sdc4,Map3k8,Tnf,Olfml2b,Cxcl1,Gem,C3ar1,Timp1,Thbs1,Icam1,Tnip1 | | 8.514e-07 | -13.98 | GSE42724\_MEMORY\_BCELL\_VS\_PLASMABLAST\_UP | MSigDB lists | GSE42724\_MEMORY\_BCELL\_VS\_PLASMABLAST\_UP | 164 | 13 | 12187 | 179 | Il6,Usp18,Parp14,Il4ra,Cmpk2,Irf7,Cflar,Nfkb1,Il1rn,Clic4,Icam1,Slfn5,Tagln2 | | 8.519e-07 | -13.98 | programmed cell death | biological process | GO:0012501 | 721 | 30 | 13711 | 214 | Nfkb1,Birc3,Sgk1,Ier3,Xaf1,Spi1,Il6,Cdkn1a,Casp4,Il1a,Gadd45b,Pik3r1,Hcar2,Gadd45g,S100a8,C5ar1,Rrp8,Bcl3,Ifit2,S100a9,Tnfsf10,Tnf,Tnfaip3,Ifi204,Lcn2,Ncf1,Irak3,Tnfaip8,Ripk1,Cflar | | 8.752e-07 | -13.95 | KOKKINAKIS\_METHIONINE\_DEPRIVATION\_48HR\_UP | MSigDB lists | KOKKINAKIS\_METHIONINE\_DEPRIVATION\_48HR\_UP | 114 | 11 | 12187 | 179 | Cflar,Plaur,Tnfsf10,Hspb1,Rtp4,Nfkbia,Cdkn1a,Birc3,Il6,Timp1,Il1a | | 8.757e-07 | -13.95 | regulation of cytokine-mediated signaling pathway | biological process | GO:0001959 | 86 | 10 | 13711 | 214 | Parp9,Il6,Casp4,Irgm2,Ripk1,Parp14,Irak3,Il1rn,Irgm1,Irf7 | | 8.783e-07 | -13.95 | ICSBP\_Q6 | MSigDB lists | ICSBP\_Q6 | 192 | 14 | 12187 | 179 | Nfkb1,Col4a1,Thbs1,Ifi44,Ifit3b,Cxcl16,Tap1,Isg15,Tubb6,Parp12,Usp18,Rbm39,Dtx3l,Angptl4 | | 8.832e-07 | -13.94 | GRAESSMANN\_APOPTOSIS\_BY\_DOXORUBICIN\_UP | MSigDB lists | GRAESSMANN\_APOPTOSIS\_BY\_DOXORUBICIN\_UP | 948 | 34 | 12187 | 179 | Tor3a,Oasl1,Parp12,Irf7,Irgm2,Ifit3b,Ifi209,Cdkn1a,Ifi44,Atf3,Ifi204,Ifi207,Zfp36,Tgm2,Usp18,Il4ra,Ccl12,Ifi211,Cxcl1,Nfkbie,Gbp7,Csf3,Rgs16,Cmpk2,Rtp4,Dnajb1,Thbs1,Mxd4,Tiparp,Cd14,Isg15,Ptpn1,Tap1,P2ry6 | | 8.858e-07 | -13.94 | FRIDMAN\_SENESCENCE\_UP | MSigDB lists | FRIDMAN\_SENESCENCE\_UP | 71 | 9 | 12187 | 179 | Cdkn1a,Ifi209,Thbs1,Ifi204,Ifi207,Tnfaip3,Isg15,Ifi211,Irf7 | | 9.055e-07 | -13.91 | positive regulation of cellular component movement | biological process | GO:0051272 | 497 | 24 | 13711 | 214 | Ccl4,Ptgs2,Fam107a,Adamts1,Rhoj,Thbs1,Lgals9,P2ry6,Pik3r1,Il1a,Akap12,Icam1,Nfe2l2,Selp,Hspa5,C5ar1,Ccl12,Cxcl16,Iqgap1,Sele,Hspb1,C3ar1,Rhoc,Ccl2 | | 9.190e-07 | -13.90 | GSE27859\_MACROPHAGE\_VS\_DC\_UP | MSigDB lists | GSE27859\_MACROPHAGE\_VS\_DC\_UP | 139 | 12 | 12187 | 179 | Sdc4,Tgm2,Parp14,Map3k8,Tnf,Igsf6,Nfkbie,Cflar,Atf3,Tnip3,Nfkb1,Pik3ap1 | | 9.190e-07 | -13.90 | GSE15330\_LYMPHOID\_MULTIPOTENT\_VS\_MEGAKARYOCYTE\_ERYTHROID\_PROGENITOR\_IKAROS\_KO\_DN | MSigDB lists | GSE15330\_LYMPHOID\_MULTIPOTENT\_VS\_MEGAKARYOCYTE\_ERYTHROID\_PROGENITOR\_IKAROS\_KO\_DN | 139 | 12 | 12187 | 179 | Ccl4,Casp4,Gem,Phf11d,S100a9,S100a8,Phf11b,Plek,Rbm47,Pik3ap1,Clic4,Errfi1 | | 9.225e-07 | -13.90 | GO\_CELLULAR\_RESPONSE\_TO\_LIPID | MSigDB lists | GO\_CELLULAR\_RESPONSE\_TO\_LIPID | 350 | 19 | 12187 | 179 | Cd14,Icam1,P2ry6,Cxcl16,Errfi1,Msn,Adamts1,Sgk1,Nfkb1,Tnip3,Csf3,Fzd4,Cmpk2,Sbno2,Tnf,Nfkbia,Tnfaip3,Ccl12,Il6 | | 9.341e-07 | -13.88 | GAJATE\_RESPONSE\_TO\_TRABECTEDIN\_UP | MSigDB lists | GAJATE\_RESPONSE\_TO\_TRABECTEDIN\_UP | 53 | 8 | 12187 | 179 | Casp4,Cdkn1a,Atf3,Oasl1,Il6,Tnfaip3,Isg15,Clic4 | | 9.419e-07 | -13.88 | host cell cytoplasm | cellular component | GO:0030430 | 13 | 5 | 13825 | 212 | Gbp7,Gbp3,Iigp1,Gbp6,Irgm2 | | 9.419e-07 | -13.88 | host cell cytoplasm part | cellular component | GO:0033655 | 13 | 5 | 13825 | 212 | Iigp1,Gbp3,Gbp7,Irgm2,Gbp6 | | 9.445e-07 | -13.87 | positive regulation of cytokine biosynthetic process | biological process | GO:0042108 | 50 | 8 | 13711 | 214 | Bcl3,Thbs1,Hspb1,Cybb,Il6,Tnf,Akap12,Il1a | | 9.489e-07 | -13.87 | lymphocyte migration | biological process | GO:0072676 | 35 | 7 | 13711 | 214 | Msn,Ccl7,Ccl4,Ccl12,Ccl2,Cxcl16,Icam1 | | 9.674e-07 | -13.85 | regulation of multicellular organismal process | biological process | GO:0051239 | 2546 | 69 | 13711 | 214 | Noct,Selp,Mmp8,Nfe2l2,Prg4,Hspa5,Cxcl1,C5ar1,Maff,Trim30a,Cd14,Cflar,Ripk1,Hspb1,Ncf1,Zfp36,Acod1,Tnf,Ptgs2,Sgk1,Runx1,Ccl4,Spi1,Cebpd,Ifi211,Isg15,Fzd4,Timp1,Hcar2,Pik3r1,Trib1,Ctla2a,Irf7,Il6,Akap12,Il1a,Nfkbia,Casp4,Il4ra,Bcl3,S100a9,Iqgap1,Nfkbiz,Lcn2,Ifi204,Zbtb16,Csf3,Adamts9,C3ar1,Il1rn,Irak3,Ccl2,Xdh,Plek,Tnfaip3,Il17ra,Kcna5,Adamts1,Rhoj,Nfkb1,Apold1,Gbp4,Lgals9,Gadd45g,Thbs1,Errfi1,Cybb,Sox11,Icam1 | | 9.776e-07 | -13.84 | GSE23308\_WT\_VS\_MINERALCORTICOID\_REC\_KO\_MACROPHAGE\_DN | MSigDB lists | GSE23308\_WT\_VS\_MINERALCORTICOID\_REC\_KO\_MACROPHAGE\_DN | 166 | 13 | 12187 | 179 | Ccl4,Gpr84,Casp4,Maff,Birc3,Ccl12,Tnfaip3,Nfe2l2,Gbp7,Nfkbiz,Znfx1,Parp9,Msn | | 9.901e-07 | -13.83 | negative regulation of molecular function | biological process | GO:0044092 | 847 | 33 | 13711 | 214 | Thbs1,Dtx3l,Trib1,Gadd45b,Gadd45g,Nfkbia,Gem,Sox11,Il6,Errfi1,Cdkn1a,Rasip1,Ptgs2,Plaur,Timp1,Birc3,Nfkb1,Parp9,Irak3,Hspb1,Tnfaip8,Cflar,Tnf,Tnfaip3,Acod1,Zfp36,Serpina3f,Angptl4,Wfdc21,Ifit2,Ptpn1,Hp,A2m | | 9.925e-07 | -13.82 | GSE39820\_IL1B\_IL6\_VS\_IL1B\_IL6\_IL23A\_TREATED\_CD4\_TCELL\_UP | MSigDB lists | GSE39820\_IL1B\_IL6\_VS\_IL1B\_IL6\_IL23A\_TREATED\_CD4\_TCELL\_UP | 140 | 12 | 12187 | 179 | Dtx3l,Gbp7,Gbp6,Irgm2,Cmpk2,Irf9,Gbp3,Sntb2,Usp18,Bach1,Parp12,Gbp4 | | 9.925e-07 | -13.82 | GSE6269\_HEALTHY\_VS\_STAPH\_AUREUS\_INF\_PBMC\_UP | MSigDB lists | GSE6269\_HEALTHY\_VS\_STAPH\_AUREUS\_INF\_PBMC\_UP | 140 | 12 | 12187 | 179 | Ifi204,Ifi207,Ifi209,Ifi44,Ifitm3,Herc6,Isg15,Parp12,Oasl1,Xaf1,Ifi211,Irf7 | | 9.976e-07 | -13.82 | KAMIKUBO\_MYELOID\_CEBPA\_NETWORK | MSigDB lists | KAMIKUBO\_MYELOID\_CEBPA\_NETWORK | 24 | 6 | 12187 | 179 | S100a9,Hp,S100a8,Mmp8,Cybb,Lcn2 | | 1.024e-06 | -13.79 | MODULE\_177 | MSigDB lists | MODULE\_177 | 93 | 10 | 12187 | 179 | Ifi211,Nfkb1,Ifi204,Ifi207,Tnfaip3,Ifi209,Il4ra,Sgk1,Nfkbia,Ifit3b | | 1.047e-06 | -13.77 | GSE23502\_BM\_VS\_COLON\_TUMOR\_MYELOID\_DERIVED\_SUPPRESSOR\_CELL\_UP | MSigDB lists | GSE23502\_BM\_VS\_COLON\_TUMOR\_MYELOID\_DERIVED\_SUPPRESSOR\_CELL\_UP | 167 | 13 | 12187 | 179 | Sbno2,Gbp7,Gem,Cxcl1,Ccnd2,Trib1,Casp4,Rbm47,Csf2rb,Tagln2,Tiparp,Stx11,Ier3 | | 1.068e-06 | -13.75 | GO\_INTRACELLULAR\_SIGNAL\_TRANSDUCTION | MSigDB lists | GO\_INTRACELLULAR\_SIGNAL\_TRANSDUCTION | 1281 | 41 | 12187 | 179 | Ripk1,Tnfaip3,Ccl12,Rhoc,Map3k8,Rhou,Pik3r1,Iqgap1,Ifi211,Pik3r5,Rrp8,Bcl3,Rhoj,Nfkb1,Sox11,Sgk1,Ptpn1,Cd14,Selp,Map3k6,Sgk3,Trib1,Nfkbia,Casp4,Tnf,Birc3,Nfkb2,Irf7,Gem,Rasd1,Ifi204,Ksr1,Ifi207,Vcam1,Socs3,Cdkn1a,Ifi209,Csf2rb,Hspb1,Sele,Plek | | 1.090e-06 | -13.73 | negative regulation of cytokine biosynthetic process | biological process | GO:0042036 | 23 | 6 | 13711 | 214 | Bcl3,Errfi1,Zfp36,Il6,Nfkb1,Prg4 | | 1.104e-06 | -13.72 | GO\_IMMUNE\_SYSTEM\_DEVELOPMENT | MSigDB lists | GO\_IMMUNE\_SYSTEM\_DEVELOPMENT | 425 | 21 | 12187 | 179 | Ifi209,Vcam1,Zbtb16,Ifi207,Tiparp,Ifi204,Herc6,Plek,Rbm47,Pik3r1,Runx1,Tnf,Spi1,Il6,Tnfaip3,C3ar1,Csf3,Bcl3,Ifi211,Sbno2,Nfkb2 | | 1.120e-06 | -13.70 | PICCALUGA\_ANGIOIMMUNOBLASTIC\_LYMPHOMA\_UP | MSigDB lists | PICCALUGA\_ANGIOIMMUNOBLASTIC\_LYMPHOMA\_UP | 168 | 13 | 12187 | 179 | Osmr,Adamts9,A2m,Rassf4,Fam107a,Ccl12,Irak3,Col4a1,Cp,Samd9l,Adamts1,Fstl1,Vcam1 | | 1.120e-06 | -13.70 | GSE26030\_TH1\_VS\_TH17\_DAY15\_POST\_POLARIZATION\_DN | MSigDB lists | GSE26030\_TH1\_VS\_TH17\_DAY15\_POST\_POLARIZATION\_DN | 168 | 13 | 12187 | 179 | P2ry6,Slfn3,Icam1,Ifitm3,Ncf1,Tagln2,Cebpd,Rtp4,Slfn4,Gbp3,Rassf4,Lgals9,Msr1 | | 1.132e-06 | -13.69 | MARKEY\_RB1\_CHRONIC\_LOF\_DN | MSigDB lists | MARKEY\_RB1\_CHRONIC\_LOF\_DN | 94 | 10 | 12187 | 179 | Ms4a6d,Cp,Ccl12,Il6,Msr1,Spi1,Slfn4,Slfn3,C3ar1,Cd14 | | 1.160e-06 | -13.67 | positive regulation of locomotion | biological process | GO:0040017 | 504 | 24 | 13711 | 214 | Ccl4,Ptgs2,Fam107a,Rhoj,Adamts1,Thbs1,Pik3r1,Lgals9,P2ry6,Icam1,Il1a,Akap12,Nfe2l2,Selp,C5ar1,Hspa5,Iqgap1,Ccl12,Cxcl16,Sele,Hspb1,C3ar1,Rhoc,Ccl2 | | 1.162e-06 | -13.66 | positive regulation of cytokine-mediated signaling pathway | biological process | GO:0001961 | 36 | 7 | 13711 | 214 | Parp9,Casp4,Irgm2,Ripk1,Parp14,Irf7,Irgm1 | | 1.162e-06 | -13.66 | GO\_REGULATION\_OF\_MONONUCLEAR\_CELL\_MIGRATION | MSigDB lists | GO\_REGULATION\_OF\_MONONUCLEAR\_CELL\_MIGRATION | 14 | 5 | 12187 | 179 | Ccl12,C3ar1,Thbs1,Tnf,C5ar1 | | 1.162e-06 | -13.66 | GO\_POSITIVE\_REGULATION\_OF\_COAGULATION | MSigDB lists | GO\_POSITIVE\_REGULATION\_OF\_COAGULATION | 14 | 5 | 12187 | 179 | S100a9,Selp,Thbs1,Nfe2l2,Plek | | 1.163e-06 | -13.66 | chemokine receptor binding | molecular function | GO:0042379 | 36 | 7 | 13516 | 211 | Ccl2,Cxcl1,Cxcl9,Ccl4,Ccl7,Cxcl16,Ccl12 | | 1.245e-06 | -13.60 | GSE24634\_TEFF\_VS\_TCONV\_DAY5\_IN\_CULTURE\_DN | MSigDB lists | GSE24634\_TEFF\_VS\_TCONV\_DAY5\_IN\_CULTURE\_DN | 143 | 12 | 12187 | 179 | Il17ra,Nfkb2,Ifitm2,Phf11d,Arid5b,Irf7,Il1rn,Runx1,Phf11b,Ifi44,Il4ra,Ifit3b | | 1.245e-06 | -13.60 | GSE34156\_NOD2\_LIGAND\_VS\_TLR1\_TLR2\_LIGAND\_6H\_TREATED\_MONOCYTE\_DN | MSigDB lists | GSE34156\_NOD2\_LIGAND\_VS\_TLR1\_TLR2\_LIGAND\_6H\_TREATED\_MONOCYTE\_DN | 143 | 12 | 12187 | 179 | Cd14,Sgk3,Clic4,Csf2rb,Cxcl16,Rbm47,Timp1,C3ar1,Igsf6,Msr1,AA467197,Cybb | | 1.245e-06 | -13.60 | GSE4984\_UNTREATED\_VS\_GALECTIN1\_TREATED\_DC\_UP | MSigDB lists | GSE4984\_UNTREATED\_VS\_GALECTIN1\_TREATED\_DC\_UP | 143 | 12 | 12187 | 179 | Spi1,Gpr84,Rhou,Bcl3,S100a9,Cflar,Sbno2,S100a8,Igsf6,Vcam1,Aff1,Zfp36 | | 1.249e-06 | -13.59 | TAKAO\_RESPONSE\_TO\_UVB\_RADIATION\_DN | MSigDB lists | TAKAO\_RESPONSE\_TO\_UVB\_RADIATION\_DN | 95 | 10 | 12187 | 179 | Ifi211,Zfp36,Nfkb2,Ifi209,Ccnd2,Dnajb1,Ifi204,Ifi207,Tnfaip3,Cebpd | | 1.251e-06 | -13.59 | molecular function regulator | molecular function | GO:0098772 | 1245 | 42 | 13516 | 211 | Tnf,Parp9,Il1a,Trib1,Cflar,Csf3,Rgs16,Tnfsf10,Hspb1,Cxcl16,Cdkn1a,Ccl4,Wfdc21,Irgm2,Socs3,Retnlg,Dtx3l,Cxcl9,Angptl4,Ccl2,Birc3,Ccnd2,A2m,Sgk1,Ccl12,Ncf1,Kcna5,Ccl7,Gem,Serpina3f,Il1rn,Timp1,Cxcl1,Dnajb1,Sgk3,Saa1,Iqgap1,Lgals9,Tnfaip8,Pik3r1,Pik3r5,Il6 | | 1.251e-06 | -13.59 | positive regulation of leukocyte migration | biological process | GO:0002687 | 111 | 11 | 13711 | 214 | C5ar1,Ccl12,Ccl4,Selp,Icam1,Il1a,Ccl2,C3ar1,Thbs1,Sele,Lgals9 | | 1.262e-06 | -13.58 | regulation of protein serine/threonine kinase activity | biological process | GO:0071900 | 401 | 21 | 13711 | 214 | Map3k8,Map3k6,Fzd4,Ptpn1,Iqgap1,Rasip1,Pik3r5,Irgm2,Tnfaip3,Tnf,Ksr1,Cdkn1a,Irak3,Il1rn,Thbs1,Trib1,Ripk1,Gadd45b,Gadd45g,Ccnd2,Lgals9 | | 1.264e-06 | -13.58 | regulation of protein kinase activity | biological process | GO:0045859 | 618 | 27 | 13711 | 214 | Sdc4,Map3k8,Fzd4,Map3k6,Pik3r5,Rasip1,Il6,Errfi1,Cdkn1a,Ccnd2,Gadd45g,Gadd45b,Lgals9,Thbs1,Trib1,Iqgap1,C5ar1,Ptpn1,Irgm2,Ksr1,Tnfaip3,Tnf,Hspb1,Il1rn,Irak3,Ncf1,Ripk1 | | 1.271e-06 | -13.58 | WINTER\_HYPOXIA\_METAGENE | MSigDB lists | WINTER\_HYPOXIA\_METAGENE | 198 | 14 | 12187 | 179 | Angptl4,Nfkb2,Tgm2,Tnfaip3,Il6,Ptgs2,Icam1,Plaur,Errfi1,Cdkn1a,Hspa5,Thbs1,Nfkb1,Cp | | 1.281e-06 | -13.57 | GSE19198\_CTRL\_VS\_IL21\_TREATED\_TCELL\_6H\_UP | MSigDB lists | GSE19198\_CTRL\_VS\_IL21\_TREATED\_TCELL\_6H\_UP | 170 | 13 | 12187 | 179 | Tnip1,Ifitm2,Ifitm3,Csf2rb,Plek,Aff1,Ifi209,Ifi207,Ifi204,Ifi211,Cxcl1,Ccl2,Birc3 | | 1.297e-06 | -13.56 | ONO\_AML1\_TARGETS\_DN | MSigDB lists | ONO\_AML1\_TARGETS\_DN | 25 | 6 | 12187 | 179 | Slfn3,Slfn4,Il17ra,Casp4,Socs3,Il4ra | | 1.297e-06 | -13.56 | TSAI\_RESPONSE\_TO\_RADIATION\_THERAPY | MSigDB lists | TSAI\_RESPONSE\_TO\_RADIATION\_THERAPY | 25 | 6 | 12187 | 179 | Col4a1,Il6,Isg15,Thbs1,Ifit2,A2m | | 1.315e-06 | -13.54 | Interleukin\_8-like\_sf | interpro domains | IPR036048 | 24 | 6 | 13788 | 212 | Ccl12,Ccl4,Ccl2,Ccl7,Cxcl9,Cxcl1 | | 1.315e-06 | -13.54 | Chemokine\_IL8-like\_dom | interpro domains | IPR001811 | 24 | 6 | 13788 | 212 | Cxcl1,Ccl2,Ccl4,Cxcl9,Ccl7,Ccl12 | | 1.317e-06 | -13.54 | regulation of I-kappaB kinase/NF-kappaB signaling | biological process | GO:0043122 | 135 | 12 | 13711 | 214 | Tnip3,Tgm2,Trim25,Tnfsf10,Il1a,Pik3ap1,Tnf,Tnfaip3,Tnip1,Ripk1,Il1rn,Hspb1 | | 1.325e-06 | -13.53 | SCY | smart domains | SM00199 | 22 | 6 | 7188 | 123 | Ccl7,Ccl12,Ccl4,Ccl2,Cxcl1,Cxcl9 | | 1.341e-06 | -13.52 | GSE36888\_UNTREATED\_VS\_IL2\_TREATED\_STAT5\_AB\_KNOCKIN\_TCELL\_6H\_UP | MSigDB lists | GSE36888\_UNTREATED\_VS\_IL2\_TREATED\_STAT5\_AB\_KNOCKIN\_TCELL\_6H\_UP | 144 | 12 | 12187 | 179 | S100a8,S100a9,Ptges,Ier3,Ksr1,Tnip3,Nfkbiz,Socs3,Thbs1,P2ry6,Pik3ap1,Cd14 | | 1.341e-06 | -13.52 | GSE41087\_WT\_VS\_FOXP3\_MUT\_ANTI\_CD3\_CD28\_STIM\_CD4\_TCELL\_UP | MSigDB lists | GSE41087\_WT\_VS\_FOXP3\_MUT\_ANTI\_CD3\_CD28\_STIM\_CD4\_TCELL\_UP | 144 | 12 | 12187 | 179 | Bach1,Tnfaip3,Birc3,Sdc4,Nfkbia,Nfkbie,Cflar,Bcl3,Nfkb1,Atf3,Csf2rb,Tnip1 | | 1.341e-06 | -13.52 | GSE21774\_CD62L\_POS\_CD56\_DIM\_VS\_CD62L\_NEG\_CD56\_DIM\_NK\_CELL\_DN | MSigDB lists | GSE21774\_CD62L\_POS\_CD56\_DIM\_VS\_CD62L\_NEG\_CD56\_DIM\_NK\_CELL\_DN | 144 | 12 | 12187 | 179 | Arid5b,Igsf6,Spi1,Tor3a,Gadd45b,Tubb6,Cd14,Ncf1,Lcn2,Il1a,Hp,Mmp8 | | 1.369e-06 | -13.50 | REACTOME\_INNATE\_IMMUNE\_SYSTEM | MSigDB lists | REACTOME\_INNATE\_IMMUNE\_SYSTEM | 171 | 13 | 12187 | 179 | Irak3,Trim25,Irf2,Isg15,Saa1,Cd14,Nfkb2,Irf7,Tnfaip3,Ripk1,Birc3,Casp4,Nfkbia | | 1.369e-06 | -13.50 | GSE43955\_1H\_VS\_20H\_ACT\_CD4\_TCELL\_UP | MSigDB lists | GSE43955\_1H\_VS\_20H\_ACT\_CD4\_TCELL\_UP | 171 | 13 | 12187 | 179 | Saa1,Tnip1,Aff1,Il1rn,Rhoj,Cxcl1,Tnfaip8,Msr1,Maff,Il4ra,Tnf,Nfe2l2,Il6 | | 1.382e-06 | -13.49 | NAKAMURA\_TUMOR\_ZONE\_PERIPHERAL\_VS\_CENTRAL\_DN | MSigDB lists | NAKAMURA\_TUMOR\_ZONE\_PERIPHERAL\_VS\_CENTRAL\_DN | 468 | 22 | 12187 | 179 | Ptgs2,Cxcl16,Dnajb1,Socs3,Hspa5,Lcn2,Cebpd,Cp,Hp,Timp1,Ksr1,Arid5b,S100a9,Bcl3,Trim56,Tnfsf10,Xdh,S100a8,Trib1,Birc3,Il6,Bach1 | | 1.416e-06 | -13.47 | BUYTAERT\_PHOTODYNAMIC\_THERAPY\_STRESS\_UP | MSigDB lists | BUYTAERT\_PHOTODYNAMIC\_THERAPY\_STRESS\_UP | 707 | 28 | 12187 | 179 | Cxcl1,Ifi211,Nfe2l2,Il6,Maff,Ip6k2,Il1a,Tiparp,Cebpd,Ier3,Mxd4,Dnajb1,Rbm39,Gem,Irf7,Bach1,Oasl1,Trib1,Nfkbia,Zfp36,Trim25,Irf9,Ptgs2,Ifi207,Ifi204,Atf3,Cdkn1a,Ifi209 | | 1.420e-06 | -13.46 | HUANG\_DASATINIB\_RESISTANCE\_UP | MSigDB lists | HUANG\_DASATINIB\_RESISTANCE\_UP | 75 | 9 | 12187 | 179 | Osmr,Msn,Ifi211,Ifi204,Samd9l,Ifi207,Ifi209,Fstl1,Ifit3b | | 1.427e-06 | -13.46 | IL8 | pfam domains | PF00048 | 24 | 6 | 12881 | 201 | Ccl7,Cxcl1,Ccl12,Cxcl9,Ccl4,Ccl2 | | 1.444e-06 | -13.45 | GSE30971\_WBP7\_HET\_VS\_KO\_MACROPHAGE\_2H\_LPS\_STIM\_DN | MSigDB lists | GSE30971\_WBP7\_HET\_VS\_KO\_MACROPHAGE\_2H\_LPS\_STIM\_DN | 145 | 12 | 12187 | 179 | Il4ra,Bach1,Il6,Gadd45b,Csf3,Cxcl1,Tnfaip8,Socs3,Tnip3,Ier3,Ptgs2,Pik3ap1 | | 1.444e-06 | -13.45 | GSE360\_CTRL\_VS\_L\_MAJOR\_DC\_DN | MSigDB lists | GSE360\_CTRL\_VS\_L\_MAJOR\_DC\_DN | 145 | 12 | 12187 | 179 | Ier3,Timp1,Il1rn,Tap1,Ptpn1,Hspb1,Ifitm3,Ccl12,Tnfaip3,Trib1,Tnf,Cflar | | 1.462e-06 | -13.44 | GSE29618\_BCELL\_VS\_MONOCYTE\_DAY7\_FLU\_VACCINE\_DN | MSigDB lists | GSE29618\_BCELL\_VS\_MONOCYTE\_DAY7\_FLU\_VACCINE\_DN | 172 | 13 | 12187 | 179 | Trib1,C5ar1,Sgk1,Atf3,Cebpd,Il1rn,Stx11,Cd14,Plaur,Icam1,Irak3,Il17ra,Rbm47 | | 1.462e-06 | -13.44 | GSE9037\_WT\_VS\_IRAK4\_KO\_LPS\_4H\_STIM\_BMDM\_UP | MSigDB lists | GSE9037\_WT\_VS\_IRAK4\_KO\_LPS\_4H\_STIM\_BMDM\_UP | 172 | 13 | 12187 | 179 | Gbp4,Map3k8,Nfkb2,Gbp7,Gbp6,Nfkbie,Csf3,Nfkb1,Il1rn,Cdkn1a,Tap1,Clic4,Herc6 | | 1.463e-06 | -13.44 | GSE40274\_CTRL\_VS\_FOXP3\_AND\_LEF1\_TRANSDUCED\_ACTIVATED\_CD4\_TCELL\_UP | MSigDB lists | GSE40274\_CTRL\_VS\_FOXP3\_AND\_LEF1\_TRANSDUCED\_ACTIVATED\_CD4\_TCELL\_UP | 120 | 11 | 12187 | 179 | C3ar1,S100a9,Apold1,Tnfaip8,Map3k8,Maff,Gpr84,Nfkbiz,Stx11,Bach1,Cebpd | | 1.516e-06 | -13.40 | Fluid shear stress and atherosclerosis | KEGG pathways | mmu05418 | 128 | 13 | 5248 | 107 | Nfe2l2,Cybb,Il1a,Ccl12,Sele,Ncf1,Nfkb1,Icam1,Tnf,Pik3r1,Ccl2,Vcam1,Sdc4 | | 1.516e-06 | -13.40 | Fluid shear stress and atherosclerosis | KEGG pathways | ko05418 | 128 | 13 | 5248 | 107 | Cybb,Icam1,Tnf,Vcam1,Sdc4,Nfkb1,Ncf1,Il1a,Ccl12,Nfe2l2,Pik3r1,Ccl2,Sele | | 1.522e-06 | -13.40 | GOUYER\_TATI\_TARGETS\_UP | MSigDB lists | GOUYER\_TATI\_TARGETS\_UP | 7 | 4 | 12187 | 179 | Lcn2,Ccl12,Zbtb16,Tnf | | 1.527e-06 | -13.39 | negative regulation of response to stimulus | biological process | GO:0048585 | 1299 | 43 | 13711 | 214 | Ptpn1,Ccl12,Tap1,A2m,Tnip3,Sgk3,Bcl3,Plek,Tnfaip3,Xdh,Il1rn,Irak3,Nfkb1,Plaur,Rgs16,Icam1,Errfi1,Thbs1,Lgals9,Hspa5,Nfe2l2,Pik3ap1,Tnf,Acod1,Zfp36,Atf3,Ripk1,Cflar,Hspb1,Rnf213,Trim30a,Tnip1,Rasip1,Ier3,Ptgs2,Nfkbia,Il4ra,Cdkn1a,Il6,Ctla2a,Trib1,Parp14,Socs3 | | 1.553e-06 | -13.38 | GSE40685\_TREG\_VS\_FOXP3\_KO\_TREG\_PRECURSOR\_DN | MSigDB lists | GSE40685\_TREG\_VS\_FOXP3\_KO\_TREG\_PRECURSOR\_DN | 146 | 12 | 12187 | 179 | Tnf,Ifit2,Socs3,Parp14,Sntb2,Casp4,Znfx1,Ifitm3,Ifitm2,P2ry6,Gbp3,Tnfsf10 | | 1.561e-06 | -13.37 | GSE15330\_LYMPHOID\_MULTIPOTENT\_VS\_PRO\_BCELL\_DN | MSigDB lists | GSE15330\_LYMPHOID\_MULTIPOTENT\_VS\_PRO\_BCELL\_DN | 173 | 13 | 12187 | 179 | Bcl3,Tnfaip8,Casp4,Maff,Ccnd2,Parp9,Clic4,Trim25,Il17ra,Socs3,Sgk1,Ms4a6d,Akap12 | | 1.586e-06 | -13.35 | negative regulation of apoptotic process | biological process | GO:0043066 | 743 | 30 | 13711 | 214 | Cflar,Tnfaip8,Ripk1,Hspb1,Il1rn,Tnf,Tnfaip3,Bcl3,Angptl4,Nfe2l2,Sgk3,Ccl12,Ptpn1,Hspa5,C5ar1,Pik3r1,Ccnd2,Socs3,Thbs1,Cdkn1a,Il6,Mt1,Icam1,Ier3,Plaur,Ptgs2,Sgk1,Nfkb1,Birc3,Timp1 | | 1.656e-06 | -13.31 | GERHOLD\_ADIPOGENESIS\_DN | MSigDB lists | GERHOLD\_ADIPOGENESIS\_DN | 57 | 8 | 12187 | 179 | Xdh,Ier3,Cebpd,Ccl12,Il4ra,Thbs1,Ccnd2,Dnajb1 | | 1.665e-06 | -13.31 | GO\_REGULATION\_OF\_VASCULAR\_ENDOTHELIAL\_GROWTH\_FACTOR\_PRODUCTION | MSigDB lists | GO\_REGULATION\_OF\_VASCULAR\_ENDOTHELIAL\_GROWTH\_FACTOR\_PRODUCTION | 26 | 6 | 12187 | 179 | C5ar1,C3ar1,Ptgs2,Il1a,Ccl12,Il6 | | 1.665e-06 | -13.31 | GSE41176\_WT\_VS\_TAK1\_KO\_ANTI\_IGM\_STIM\_BCELL\_6H\_DN | MSigDB lists | GSE41176\_WT\_VS\_TAK1\_KO\_ANTI\_IGM\_STIM\_BCELL\_6H\_DN | 174 | 13 | 12187 | 179 | Socs3,Nfkb1,Ptgs2,Tnip1,Birc3,Il4ra,Nfkbia,Ccl4,Tnfaip3,C3ar1,Cflar,Tnfaip8,Igsf6 | | 1.665e-06 | -13.31 | GSE17721\_POLYIC\_VS\_PAM3CSK4\_1H\_BMDC\_DN | MSigDB lists | GSE17721\_POLYIC\_VS\_PAM3CSK4\_1H\_BMDC\_DN | 174 | 13 | 12187 | 179 | Slfn3,Zfp36,Icam1,Slfn4,Errfi1,Tnip1,Hcar2,Tiparp,Ccl12,Birc3,Trib1,Ccl2,Tgm2 | | 1.665e-06 | -13.31 | BILD\_HRAS\_ONCOGENIC\_SIGNATURE | MSigDB lists | BILD\_HRAS\_ONCOGENIC\_SIGNATURE | 174 | 13 | 12187 | 179 | Nfkbiz,Timp1,Il1a,Akap12,Ier3,Ptgs2,Zfp36,Plaur,Runx1,Sdc4,Trib1,Cxcl1,Angptl4 | | 1.724e-06 | -13.27 | GSE18791\_CTRL\_VS\_NEWCASTLE\_VIRUS\_DC\_18H\_DN | MSigDB lists | GSE18791\_CTRL\_VS\_NEWCASTLE\_VIRUS\_DC\_18H\_DN | 122 | 11 | 12187 | 179 | Irf7,Dtx3l,Tnfsf10,Gbp3,Parp14,Tnf,Ptgs2,Herc6,Tap1,Adamts1,Znfx1 | | 1.776e-06 | -13.24 | GSE39152\_BRAIN\_VS\_SPLEEN\_CD103\_NEG\_MEMORY\_CD8\_TCELL\_DN | MSigDB lists | GSE39152\_BRAIN\_VS\_SPLEEN\_CD103\_NEG\_MEMORY\_CD8\_TCELL\_DN | 175 | 13 | 12187 | 179 | Clic4,Csf2rb,Ccdc58,Ptpn1,Il17ra,Icam1,Atf3,Gadd45g,Gbp7,Bach1,Ccl4,Casp4,Il4ra | | 1.776e-06 | -13.24 | GSE22196\_HEALTHY\_VS\_OBESE\_MOUSE\_SKIN\_GAMMADELTA\_TCELL\_DN | MSigDB lists | GSE22196\_HEALTHY\_VS\_OBESE\_MOUSE\_SKIN\_GAMMADELTA\_TCELL\_DN | 175 | 13 | 12187 | 179 | Ccl4,Tnfaip3,Cxcl1,S100a9,Gem,S100a8,Thbs1,Atf3,Ier3,Tiparp,Nfkb1,Tnip1,Plaur | | 1.776e-06 | -13.24 | GSE17721\_0.5H\_VS\_8H\_POLYIC\_BMDC\_DN | MSigDB lists | GSE17721\_0.5H\_VS\_8H\_POLYIC\_BMDC\_DN | 175 | 13 | 12187 | 179 | Slfn3,Trim25,Msn,Cdkn1a,Adamts1,Il1rn,Atf3,Luc7l3,Irgm2,Slfn4,Map3k8,Tgm2,Hcar2 | | 1.779e-06 | -13.24 | GO\_NEGATIVE\_REGULATION\_OF\_CYSTEINE\_TYPE\_ENDOPEPTIDASE\_ACTIVITY | MSigDB lists | GO\_NEGATIVE\_REGULATION\_OF\_CYSTEINE\_TYPE\_ENDOPEPTIDASE\_ACTIVITY | 77 | 9 | 12187 | 179 | Thbs1,Birc3,Ifi209,Ifi207,Ifi204,Il6,Ifi211,Tnfaip8,Plaur | | 1.794e-06 | -13.23 | GSE28737\_FOLLICULAR\_VS\_MARGINAL\_ZONE\_BCELL\_BCL6\_HET\_DN | MSigDB lists | GSE28737\_FOLLICULAR\_VS\_MARGINAL\_ZONE\_BCELL\_BCL6\_HET\_DN | 148 | 12 | 12187 | 179 | Dtx3l,Gbp6,Herc6,Angptl4,Irf7,Slfn4,Slfn3,Tap1,Msn,Ccnd2,Gpr84,Gbp4 | | 1.820e-06 | -13.22 | GRUETZMANN\_PANCREATIC\_CANCER\_UP | MSigDB lists | GRUETZMANN\_PANCREATIC\_CANCER\_UP | 298 | 17 | 12187 | 179 | Ifitm2,Ptgs2,Msn,Tap1,Plaur,Atf3,Lcn2,Akap12,Il1rn,Gem,Cflar,Rgs16,Iqgap1,Casp4,Il4ra,Birc3,Tnfaip3 | | 1.831e-06 | -13.21 | receptor ligand activity | molecular function | GO:0048018 | 249 | 16 | 13516 | 211 | Cxcl16,Saa1,Ccl4,Il6,Cxcl9,Retnlg,Ccl2,Tnf,Ccl12,Ccl7,Il1a,Il1rn,Csf3,Cxcl1,Timp1,Tnfsf10 | | 1.834e-06 | -13.21 | GBP\_C | interpro domains | IPR037684 | 7 | 4 | 13788 | 212 | Gbp3,Gbp7,Gbp6,Gbp4 | | 1.870e-06 | -13.19 | WU\_CELL\_MIGRATION | MSigDB lists | WU\_CELL\_MIGRATION | 123 | 11 | 12187 | 179 | Col4a1,Il1a,Akap12,Tubb6,Thbs1,Adamts1,Tgm2,Msn,Ptgs2,Arid5b,Ptges | | 1.924e-06 | -13.16 | CHICAS\_RB1\_TARGETS\_SENESCENT | MSigDB lists | CHICAS\_RB1\_TARGETS\_SENESCENT | 440 | 21 | 12187 | 179 | Il1a,Akap12,Rasd1,Sox11,Vcam1,Nfkbiz,Ifit3b,Irf9,Ptgs2,Isg15,Tnfaip3,AA467197,Il6,Birc3,Tnfaip8,Rbm39,Osmr,Dtx3l,Gem,Csf3,Cxcl1 | | 1.926e-06 | -13.16 | GSE28737\_WT\_VS\_BCL6\_HET\_MARGINAL\_ZONE\_BCELL\_DN | MSigDB lists | GSE28737\_WT\_VS\_BCL6\_HET\_MARGINAL\_ZONE\_BCELL\_DN | 149 | 12 | 12187 | 179 | Stx11,Cdkn1a,Pik3ap1,Rbm47,Icam1,Isg15,Rhoc,Tnfaip3,Ripk1,Casp4,Trib1,Gbp7 | | 1.934e-06 | -13.16 | AMIT\_EGF\_RESPONSE\_40\_HELA | MSigDB lists | AMIT\_EGF\_RESPONSE\_40\_HELA | 41 | 7 | 12187 | 179 | Sgk1,Il6,Atf3,Ier3,Ptgs2,Bcl3,Zfp36 | | 1.934e-06 | -13.16 | LEE\_LIVER\_CANCER\_DENA\_UP | MSigDB lists | LEE\_LIVER\_CANCER\_DENA\_UP | 41 | 7 | 12187 | 179 | S100a9,Tagln2,Plek,Thbs1,Vcam1,Col4a1,Lcn2 | | 1.934e-06 | -13.16 | MODULE\_254 | MSigDB lists | MODULE\_254 | 41 | 7 | 12187 | 179 | Ccnd2,Birc3,Nfkb1,Il1a,Ier3,Cflar,Tnfsf10 | | 2.006e-06 | -13.12 | regulation of response to cytokine stimulus | biological process | GO:0060759 | 94 | 10 | 13711 | 214 | Irgm2,Parp14,Ripk1,Il1rn,Irak3,Irgm1,Irf7,Parp9,Il6,Casp4 | | 2.018e-06 | -13.11 | SENESE\_HDAC1\_AND\_HDAC2\_TARGETS\_UP | MSigDB lists | SENESE\_HDAC1\_AND\_HDAC2\_TARGETS\_UP | 177 | 13 | 12187 | 179 | Nfkbiz,Il1a,Plaur,Tgm2,Maff,Birc3,Ccnd2,Tnfaip3,Robo4,Gem,Nfkb2,Osmr,Iqgap1 | | 2.067e-06 | -13.09 | GSE25123\_IL4\_VS\_IL4\_AND\_ROSIGLITAZONE\_STIM\_PPARG\_KO\_MACROPHAGE\_DAY10\_DN | MSigDB lists | GSE25123\_IL4\_VS\_IL4\_AND\_ROSIGLITAZONE\_STIM\_PPARG\_KO\_MACROPHAGE\_DAY10\_DN | 150 | 12 | 12187 | 179 | AA467197,Gpr84,Tnf,Cxcl1,Il1rn,Timp1,Il1a,Aff1,Errfi1,Trim25,Pik3ap1,Ptgs2 | | 2.114e-06 | -13.07 | regulation of viral life cycle | biological process | GO:1903900 | 117 | 11 | 13711 | 214 | Isg15,Ifitm2,Trim56,Resf1,Trim25,Ifitm6,Tnf,Oasl2,Trim30a,Oasl1,Ifitm3 | | 2.209e-06 | -13.02 | MODULE\_88 | MSigDB lists | MODULE\_88 | 520 | 23 | 12187 | 179 | Ccnd2,Il4ra,Tgm2,Trib1,Ccl12,Fam107a,S100a9,S100a8,Xdh,Sbno2,Tnfsf10,Cdkn1a,Il1rn,Kcna5,Col4a1,Hp,Cp,Gadd45g,Ier3,Lcn2,Isg15,Cd14,Sele | | 2.218e-06 | -13.02 | GSE9988\_ANTI\_TREM1\_VS\_CTRL\_TREATED\_MONOCYTES\_UP | MSigDB lists | GSE9988\_ANTI\_TREM1\_VS\_CTRL\_TREATED\_MONOCYTES\_UP | 151 | 12 | 12187 | 179 | Sgk1,Cdkn1a,Atf3,Rasd1,Ptgs2,Plaur,Tnf,Trib1,Gadd45b,Gem,Banp,Cxcl1 | | 2.245e-06 | -13.01 | Fadd (Fas (TNFRSF6)-associated via death domain) | protein interactions | 14082 | 14 | 5 | 6802 | 115 | Tnf,Cflar,Ripk1,Tnfaip3,Birc3 | | 2.255e-06 | -13.00 | signaling receptor activator activity | molecular function | GO:0030546 | 253 | 16 | 13516 | 211 | Saa1,Cxcl16,Ccl4,Ccl2,Retnlg,Cxcl9,Il6,Ccl12,Tnf,Ccl7,Csf3,Il1rn,Il1a,Tnfsf10,Timp1,Cxcl1 | | 2.293e-06 | -12.99 | LEE\_LIVER\_CANCER\_E2F1\_UP | MSigDB lists | LEE\_LIVER\_CANCER\_E2F1\_UP | 42 | 7 | 12187 | 179 | Xdh,Lcn2,Tagln2,Casp4,Usp18,Ifit2,Ifi44 | | 2.293e-06 | -12.99 | KHETCHOUMIAN\_TRIM24\_TARGETS\_UP | MSigDB lists | KHETCHOUMIAN\_TRIM24\_TARGETS\_UP | 42 | 7 | 12187 | 179 | Icam1,Cybb,Col4a1,Cp,Tgm2,Vcam1,Cdkn1a | | 2.320e-06 | -12.97 | cell death | biological process | GO:0008219 | 757 | 30 | 13711 | 214 | Tnf,Tnfaip3,Tnfsf10,Irak3,Ncf1,Ripk1,Tnfaip8,Cflar,Ifi204,Lcn2,C5ar1,Rrp8,S100a8,S100a9,Bcl3,Ifit2,Casp4,Il1a,Il6,Cdkn1a,Gadd45b,Pik3r1,Gadd45g,Hcar2,Birc3,Nfkb1,Xaf1,Spi1,Sgk1,Ier3 | | 2.352e-06 | -12.96 | LU\_AGING\_BRAIN\_UP | MSigDB lists | LU\_AGING\_BRAIN\_UP | 239 | 15 | 12187 | 179 | Ifi209,Aff1,Tiparp,Ifi204,Ifi207,Hp,Cebpd,Clic4,Msn,Tgm2,Nfkbia,Nfe2l2,Ifi211,Irf7,Iqgap1 | | 2.373e-06 | -12.95 | PETROVA\_ENDOTHELIUM\_LYMPHATIC\_VS\_BLOOD\_DN | MSigDB lists | PETROVA\_ENDOTHELIUM\_LYMPHATIC\_VS\_BLOOD\_DN | 126 | 11 | 12187 | 179 | Icam1,Sele,Selp,Isg15,Ccl12,Fam107a,Ier3,Il6,Cdkn1a,Il4ra,Tgm2 | | 2.377e-06 | -12.95 | GSE22935\_24H\_VS\_48H\_MBOVIS\_BCG\_STIM\_MACROPHAGE\_UP | MSigDB lists | GSE22935\_24H\_VS\_48H\_MBOVIS\_BCG\_STIM\_MACROPHAGE\_UP | 152 | 12 | 12187 | 179 | Gbp4,Ms4a6d,Parp14,Birc3,Ifit2,Rgs16,Trim25,Slfn9,Gbp6,Gbp7,Dtx3l,Irgm2 | | 2.377e-06 | -12.95 | GSE17721\_POLYIC\_VS\_PAM3CSK4\_2H\_BMDC\_DN | MSigDB lists | GSE17721\_POLYIC\_VS\_PAM3CSK4\_2H\_BMDC\_DN | 152 | 12 | 12187 | 179 | Gpr84,Pik3r1,Rhou,Map3k8,Igsf6,Nfkbiz,Thbs1,Il1rn,Nfkb1,Ptgs2,Errfi1,Il17ra | | 2.377e-06 | -12.95 | GSE42724\_NAIVE\_VS\_MEMORY\_BCELL\_UP | MSigDB lists | GSE42724\_NAIVE\_VS\_MEMORY\_BCELL\_UP | 152 | 12 | 12187 | 179 | Nfkbie,Gbp7,Cflar,Tnfsf10,Msr1,Birc3,Hcar2,Ptgs2,Tnip1,Plin4,Cxcl16,Hp | | 2.383e-06 | -12.95 | negative regulation of programmed cell death | biological process | GO:0043069 | 758 | 30 | 13711 | 214 | Ccnd2,Pik3r1,Thbs1,Socs3,Cdkn1a,Il6,Icam1,Mt1,Plaur,Ier3,Sgk1,Ptgs2,Nfkb1,Birc3,Timp1,Ripk1,Tnfaip8,Cflar,Il1rn,Hspb1,Tnfaip3,Tnf,Angptl4,Bcl3,Nfe2l2,Sgk3,Ccl12,Ptpn1,Hspa5,C5ar1 | | 2.391e-06 | -12.94 | regulation of vascular endothelial growth factor production | biological process | GO:0010574 | 26 | 6 | 13711 | 214 | Il1a,C5ar1,Ccl2,Il6,C3ar1,Ptgs2 | | 2.391e-06 | -12.94 | regulation of viral entry into host cell | biological process | GO:0046596 | 26 | 6 | 13711 | 214 | Ifitm2,Ifitm6,Ifitm3,Trim30a,Trim25,Trim56 | | 2.391e-06 | -12.94 | regulation of epithelial cell apoptotic process | biological process | GO:1904035 | 75 | 9 | 13711 | 214 | Icam1,Tnf,Tnfaip3,Zfp36,Ccl12,Thbs1,Nfe2l2,Cflar,Angptl4 | | 2.462e-06 | -12.91 | positive regulation of response to cytokine stimulus | biological process | GO:0060760 | 40 | 7 | 13711 | 214 | Casp4,Parp9,Ripk1,Parp14,Irgm1,Irf7,Irgm2 | | 2.462e-06 | -12.91 | negative regulation of innate immune response | biological process | GO:0045824 | 40 | 7 | 13711 | 214 | Acod1,Tnfaip3,A2m,Tap1,Parp14,Irak3,Lgals9 | | 2.470e-06 | -12.91 | GO\_REGULATION\_OF\_CYTOKINE\_BIOSYNTHETIC\_PROCESS | MSigDB lists | GO\_REGULATION\_OF\_CYTOKINE\_BIOSYNTHETIC\_PROCESS | 60 | 8 | 12187 | 179 | Thbs1,Tnf,Il6,Il1a,Nfkb1,Bcl3,Errfi1,Hspb1 | | 2.471e-06 | -12.91 | GO\_NEGATIVE\_REGULATION\_OF\_CATALYTIC\_ACTIVITY | MSigDB lists | GO\_NEGATIVE\_REGULATION\_OF\_CATALYTIC\_ACTIVITY | 603 | 25 | 12187 | 179 | Ptpn1,Irak3,Thbs1,Nfkb1,Angptl4,Ifi211,Tnfaip8,Iqgap1,Il6,Gadd45b,Tnfaip3,Errfi1,Plaur,Hspb1,Socs3,Ifi209,Cdkn1a,Ifi207,Hp,Ifi204,Timp1,A2m,Trib1,Birc3,Tnf | | 2.539e-06 | -12.88 | regulation of hemopoiesis | biological process | GO:1903706 | 318 | 18 | 13711 | 214 | Zfp36,Il6,Il4ra,Tnf,Nfkbia,Pik3r1,Lgals9,Csf3,Ripk1,Trib1,Ctla2a,Zbtb16,Isg15,Cxcl1,Nfkbiz,Runx1,Nfe2l2,Spi1 | | 2.547e-06 | -12.88 | GSE17721\_CTRL\_VS\_PAM3CSK4\_1H\_BMDC\_DN | MSigDB lists | GSE17721\_CTRL\_VS\_PAM3CSK4\_1H\_BMDC\_DN | 153 | 12 | 12187 | 179 | Igsf6,Slfn4,Cflar,Hcar2,Map3k8,Birc3,Ccl2,Icam1,Slfn3,Errfi1,Atf3,Cdkn1a | | 2.547e-06 | -12.88 | GSE13485\_DAY1\_VS\_DAY3\_YF17D\_VACCINE\_PBMC\_DN | MSigDB lists | GSE13485\_DAY1\_VS\_DAY3\_YF17D\_VACCINE\_PBMC\_DN | 153 | 12 | 12187 | 179 | Phf11b,Ifi44,Ifit3b,Sgk3,Herc6,Isg15,Parp9,Tap1,Xaf1,Usp18,Phf11d,Cmpk2 | | 2.547e-06 | -12.88 | GSE10325\_CD4\_TCELL\_VS\_MYELOID\_DN | MSigDB lists | GSE10325\_CD4\_TCELL\_VS\_MYELOID\_DN | 153 | 12 | 12187 | 179 | Cdkn1a,Msr1,Trib1,Cybb,Tubb6,Nfkbie,Icam1,Plek,Rbm47,Clic4,Csf2rb,Plaur | | 2.547e-06 | -12.88 | GSE17721\_0.5H\_VS\_4H\_LPS\_BMDC\_DN | MSigDB lists | GSE17721\_0.5H\_VS\_4H\_LPS\_BMDC\_DN | 153 | 12 | 12187 | 179 | Trim25,Rbm47,Timp1,Nfkb1,Iqgap1,Irgm2,Parp14,Tor3a,Ccl2,Tnf,Zfp189,Birc3 | | 2.590e-06 | -12.86 | GSE43957\_UNTREATED\_VS\_NACL\_TREATED\_ANTI\_CD3\_CD28\_STIM\_CD4\_TCELL\_DN | MSigDB lists | GSE43957\_UNTREATED\_VS\_NACL\_TREATED\_ANTI\_CD3\_CD28\_STIM\_CD4\_TCELL\_DN | 181 | 13 | 12187 | 179 | Errfi1,Clic4,Sele,Il17ra,Icam1,Saa1,Selp,Gadd45b,Bach1,Tubb6,Ccl2,Il4ra,Map3k8 | | 2.599e-06 | -12.86 | P-loop\_NTPase | interpro domains | IPR027417 | 732 | 29 | 13788 | 212 | Tap1,Gbp7,F830016B08Rik,Ifi47,Rhoc,Ifi44,Cmpk2,Rhou,Tor3a,Gem,Mx2,Slfn5,9930111J21Rik2,Gm5431,Tgtp1,Gbp3,Rasd1,Iqgap1,Sbno2,Iigp1,Slfn9,Gbp6,Mx1,Znfx1,Irgm1,Irgm2,Gbp4,Rhoj,Gm4841 | | 2.611e-06 | -12.86 | regulation of smooth muscle cell proliferation | biological process | GO:0048660 | 144 | 12 | 13711 | 214 | Tgm2,Adamts1,Ptgs2,Tnf,Tnfaip3,Cdkn1a,Il6,Trib1,Thbs1,C3ar1,P2ry6,Pik3r1 | | 2.627e-06 | -12.85 | GO\_REGULATION\_OF\_HYDROLASE\_ACTIVITY | MSigDB lists | GO\_REGULATION\_OF\_HYDROLASE\_ACTIVITY | 995 | 34 | 12187 | 179 | Hspa5,Thbs1,Dnajb1,Rasip1,Ptpn1,Ccl2,Ccl4,Rhoc,Il6,Ccl12,Ripk1,Angptl4,Ifi211,S100a8,Tnfaip8,Iqgap1,Rgs16,Ifi209,Ifi207,Timp1,Ifi204,Sele,Plek,Icam1,Plaur,Csf2rb,Errfi1,Birc3,Tnf,C5ar1,S100a9,A2m,Tnfsf10,Xdh | | 2.638e-06 | -12.85 | NABA\_MATRISOME | MSigDB lists | NABA\_MATRISOME | 565 | 24 | 12187 | 179 | Fstl1,Adamts1,Col4a1,Timp1,Il1rn,S100a9,A2m,Tnfsf10,Sdc4,Lgals9,Tnf,Thbs1,Il1a,Mmp8,Cxcl1,Angptl4,Csf3,S100a8,Adamts9,Ccl2,Ccl4,Tgm2,Il6,Ccl12 | | 2.659e-06 | -12.84 | AMIT\_SERUM\_RESPONSE\_40\_MCF10A | MSigDB lists | AMIT\_SERUM\_RESPONSE\_40\_MCF10A | 28 | 6 | 12187 | 179 | Atf3,Clic4,Zfp36,Il1a,Cxcl1,Thbs1 | | 2.662e-06 | -12.84 | regulation of kinase activity | biological process | GO:0043549 | 682 | 28 | 13711 | 214 | Sdc4,Map3k8,Map3k6,Fzd4,Pik3r5,Rasip1,Il6,Errfi1,Cdkn1a,Pik3r1,Gadd45g,Ccnd2,Gadd45b,Lgals9,Thbs1,Trib1,Iqgap1,C5ar1,Ptpn1,Irgm2,Ksr1,Tnfaip3,Tnf,Hspb1,Il1rn,Irak3,Ncf1,Ripk1 | | 2.727e-06 | -12.81 | TONKS\_TARGETS\_OF\_RUNX1\_RUNX1T1\_FUSION\_HSC\_UP | MSigDB lists | TONKS\_TARGETS\_OF\_RUNX1\_RUNX1T1\_FUSION\_HSC\_UP | 154 | 12 | 12187 | 179 | Ifi211,Irf7,Arid5b,Nfkb2,Runx1,Rhoc,Hspb1,Irf9,Ifi209,Fstl1,Ifi204,Ifi207 | | 2.727e-06 | -12.81 | GSE17974\_CTRL\_VS\_ACT\_IL4\_AND\_ANTI\_IL12\_72H\_CD4\_TCELL\_UP | MSigDB lists | GSE17974\_CTRL\_VS\_ACT\_IL4\_AND\_ANTI\_IL12\_72H\_CD4\_TCELL\_UP | 154 | 12 | 12187 | 179 | Maff,Casp4,Trib1,Dtx3l,Pik3r5,S100a8,Gbp3,Sgk1,Nfkbiz,Socs3,Herc6,Plek | | 2.841e-06 | -12.77 | GO\_CELLULAR\_HOMEOSTASIS | MSigDB lists | GO\_CELLULAR\_HOMEOSTASIS | 489 | 22 | 12187 | 179 | S100a9,C5ar1,C3ar1,Kcna5,Cp,Ncf1,Saa1,Clic4,Icam1,Tgm2,Ccl2,Pik3r1,Ccl12,Nfe2l2,Il6,Slc24a4,Cybb,Pygm,S100a8,Sgk1,Lcn2,Sgk3 | | 2.855e-06 | -12.77 | DANG\_REGULATED\_BY\_MYC\_DN | MSigDB lists | DANG\_REGULATED\_BY\_MYC\_DN | 212 | 14 | 12187 | 179 | Irf7,A2m,Vcam1,Fstl1,Thbs1,Cdkn1a,Ier3,Akap12,Gadd45g,Msn,Ptpn1,Errfi1,Plaur,Hspb1 | | 2.879e-06 | -12.76 | CHIARADONNA\_NEOPLASTIC\_TRANSFORMATION\_KRAS\_UP | MSigDB lists | CHIARADONNA\_NEOPLASTIC\_TRANSFORMATION\_KRAS\_UP | 104 | 10 | 12187 | 179 | Vcam1,Casp4,Nfkbia,Ccl12,Nfe2l2,Tubb6,Tiparp,Timp1,Bach1,Rgs16 | | 2.919e-06 | -12.74 | GSE45365\_NK\_CELL\_VS\_CD8\_TCELL\_MCMV\_INFECTION\_DN | MSigDB lists | GSE45365\_NK\_CELL\_VS\_CD8\_TCELL\_MCMV\_INFECTION\_DN | 155 | 12 | 12187 | 179 | Nfkb2,Gadd45b,Tnfaip3,Rassf4,Nfkbia,Sdc4,Birc3,Tap1,Ptpn1,Tnip1,Nfkb1,Cdkn1a | | 2.930e-06 | -12.74 | acute inflammatory response | biological process | GO:0002526 | 41 | 7 | 13711 | 214 | Cxcl1,Icam1,Il1a,Hp,Il6,Saa1,Il1rn | | 3.009e-06 | -12.71 | GO\_LEUKOCYTE\_MIGRATION\_INVOLVED\_IN\_INFLAMMATORY\_RESPONSE | MSigDB lists | GO\_LEUKOCYTE\_MIGRATION\_INVOLVED\_IN\_INFLAMMATORY\_RESPONSE | 8 | 4 | 12187 | 179 | Sele,S100a8,Ccl12,S100a9 | | 3.025e-06 | -12.71 | regulation of transferase activity | biological process | GO:0051338 | 767 | 30 | 13711 | 214 | Lgals9,Pik3r1,Ccnd2,Gadd45g,Gadd45b,Trib1,Dtx3l,Thbs1,Cdkn1a,Il6,Errfi1,Pik3r5,Rasip1,Sdc4,Map3k6,Fzd4,Map3k8,Ripk1,Hspb1,Il1rn,Irak3,Ncf1,Ksr1,Zfp36,Tnfaip3,Tnf,Irgm2,Iqgap1,Ptpn1,C5ar1 | | 3.063e-06 | -12.70 | BLALOCK\_ALZHEIMERS\_DISEASE\_UP | MSigDB lists | BLALOCK\_ALZHEIMERS\_DISEASE\_UP | 1384 | 42 | 12187 | 179 | Tnfsf10,Igsf6,Irf7,Cflar,Gem,Oasl1,Casp4,Nfkbia,Icam1,Zfp36,Tnip1,Ifitm2,Luc7l3,Ifi204,Ifi207,Cp,Vcam1,Socs3,Zbtb16,Ifi209,Aff1,Rgs16,Adamts9,Iqgap1,Tnfaip8,Banp,Cxcl1,Ifi211,Tnfaip3,Fam107a,Gadd45b,Il6,Maff,Tap1,Il17ra,Mxd4,Cebpd,Tiparp,Nfkb1,Il1a,Gadd45g,Sgk1 | | 3.079e-06 | -12.69 | host intracellular part | cellular component | GO:0033646 | 16 | 5 | 13825 | 212 | Irgm2,Gbp6,Iigp1,Gbp3,Gbp7 | | 3.079e-06 | -12.69 | intracellular region of host | cellular component | GO:0043656 | 16 | 5 | 13825 | 212 | Gbp3,Iigp1,Gbp7,Irgm2,Gbp6 | | 3.116e-06 | -12.68 | MARTINELLI\_IMMATURE\_NEUTROPHIL\_UP | MSigDB lists | MARTINELLI\_IMMATURE\_NEUTROPHIL\_UP | 3 | 3 | 12187 | 179 | Cybb,Mmp8,Lcn2 | | 3.122e-06 | -12.68 | GSE17721\_12H\_VS\_24H\_CPG\_BMDC\_UP | MSigDB lists | GSE17721\_12H\_VS\_24H\_CPG\_BMDC\_UP | 156 | 12 | 12187 | 179 | Rhoc,Gbp4,Msr1,Pik3r1,Ccl2,Trib1,Gbp6,Ptges,Il1a,Dnajb1,Zfp36,Clic4 | | 3.177e-06 | -12.66 | GO\_REGULATION\_OF\_EXTRINSIC\_APOPTOTIC\_SIGNALING\_PATHWAY\_VIA\_DEATH\_DOMAIN\_RECEPTORS | MSigDB lists | GO\_REGULATION\_OF\_EXTRINSIC\_APOPTOTIC\_SIGNALING\_PATHWAY\_VIA\_DEATH\_DOMAIN\_RECEPTORS | 44 | 7 | 12187 | 179 | Cflar,Icam1,Tnfsf10,Thbs1,Atf3,Ripk1,Tnfaip3 | | 3.177e-06 | -12.66 | FURUKAWA\_DUSP6\_TARGETS\_PCI35\_UP | MSigDB lists | FURUKAWA\_DUSP6\_TARGETS\_PCI35\_UP | 44 | 7 | 12187 | 179 | Phf11b,AA467197,Phf11d,Herc6,Saa1,Parp9,Rtp4 | | 3.317e-06 | -12.62 | GO\_REGULATION\_OF\_MULTI\_ORGANISM\_PROCESS | MSigDB lists | GO\_REGULATION\_OF\_MULTI\_ORGANISM\_PROCESS | 346 | 18 | 12187 | 179 | Ifi209,Ifi207,Ifi204,Timp1,Tnip1,Ifitm2,Isg15,Ifitm3,Tap1,Trim25,Trib1,Ccl4,Birc3,Tnf,Zfp189,Oasl1,Tnfaip3,Ifi211 | | 3.338e-06 | -12.61 | GSE29949\_MICROGLIA\_BRAIN\_VS\_MONOCYTE\_BONE\_MARROW\_DN | MSigDB lists | GSE29949\_MICROGLIA\_BRAIN\_VS\_MONOCYTE\_BONE\_MARROW\_DN | 157 | 12 | 12187 | 179 | Il6,Gem,Nfkbie,Ifi211,Cxcl1,Ifi44,Ifi209,Ifi207,Ifi204,Mmp8,Atf3,Isg15 | | 3.338e-06 | -12.61 | GO\_NEGATIVE\_REGULATION\_OF\_APOPTOTIC\_SIGNALING\_PATHWAY | MSigDB lists | GO\_NEGATIVE\_REGULATION\_OF\_APOPTOTIC\_SIGNALING\_PATHWAY | 157 | 12 | 12187 | 179 | Cflar,Tnfsf10,Tnf,Ripk1,Tnfaip3,Nfe2l2,Ptpn1,Plaur,Hspb1,Icam1,Thbs1,Il1a | | 3.338e-06 | -12.61 | GSE11961\_FOLLICULAR\_BCELL\_VS\_GERMINAL\_CENTER\_BCELL\_DAY7\_DN | MSigDB lists | GSE11961\_FOLLICULAR\_BCELL\_VS\_GERMINAL\_CENTER\_BCELL\_DAY7\_DN | 157 | 12 | 12187 | 179 | Ifi44,Cdkn1a,Znfx1,Rnf213,Slfn9,Pik3ap1,Il4ra,Ccnd2,Trib1,Ripk1,Dtx3l,Fzd4 | | 3.356e-06 | -12.60 | positive regulation of hemostasis | biological process | GO:1900048 | 16 | 5 | 13711 | 214 | Selp,Nfe2l2,Thbs1,Plek,S100a9 | | 3.356e-06 | -12.60 | positive regulation of blood coagulation | biological process | GO:0030194 | 16 | 5 | 13711 | 214 | Selp,Plek,S100a9,Nfe2l2,Thbs1 | | 3.361e-06 | -12.60 | MODULE\_436 | MSigDB lists | MODULE\_436 | 83 | 9 | 12187 | 179 | Parp14,Thbs1,Cebpd,Cd14,Ifitm3,Isg15,Csf3,Tnfsf10,Tap1 | | 3.477e-06 | -12.57 | TONKS\_TARGETS\_OF\_RUNX1\_RUNX1T1\_FUSION\_ERYTHROCYTE\_UP | MSigDB lists | TONKS\_TARGETS\_OF\_RUNX1\_RUNX1T1\_FUSION\_ERYTHROCYTE\_UP | 131 | 11 | 12187 | 179 | Tubb6,Il1rn,Ifi204,Ifi207,Ifi209,Ifi44,Socs3,Hspb1,Irak3,C3ar1,Ifi211 | | 3.509e-06 | -12.56 | GO\_REGULATION\_OF\_KINASE\_ACTIVITY | MSigDB lists | GO\_REGULATION\_OF\_KINASE\_ACTIVITY | 615 | 25 | 12187 | 179 | Map3k6,Ptpn1,Irak3,Hspa5,Thbs1,Gadd45g,Pik3r5,Iqgap1,Pik3r1,Ccnd2,Map3k8,Il6,Gadd45b,Ripk1,Tnfaip3,Saa1,Errfi1,Hspb1,Socs3,Cdkn1a,Fzd4,C5ar1,Trib1,Sdc4,Tnf | | 3.566e-06 | -12.54 | GSE16385\_ROSIGLITAZONE\_IFNG\_TNF\_VS\_IL4\_STIM\_MACROPHAGE\_UP | MSigDB lists | GSE16385\_ROSIGLITAZONE\_IFNG\_TNF\_VS\_IL4\_STIM\_MACROPHAGE\_UP | 158 | 12 | 12187 | 179 | Map3k8,Maff,Ccl4,Trib1,Tnfaip3,Gem,Adamts9,Hspa5,Nfkbiz,Dnajb1,Irak3,Zfp36 | | 3.566e-06 | -12.54 | GSE17721\_CTRL\_VS\_GARDIQUIMOD\_8H\_BMDC\_DN | MSigDB lists | GSE17721\_CTRL\_VS\_GARDIQUIMOD\_8H\_BMDC\_DN | 158 | 12 | 12187 | 179 | Gbp3,Pik3ap1,Il17ra,Igsf6,Cd14,Slfn9,Map3k6,Znfx1,Nfkb1,Nfkbia,Birc3,Ifit2 | | 3.586e-06 | -12.54 | symbiont-containing vacuole membrane | cellular component | GO:0020005 | 8 | 4 | 13825 | 212 | Gbp6,Gbp7,Gbp3,Iigp1 | | 3.602e-06 | -12.53 | BURTON\_ADIPOGENESIS\_2 | MSigDB lists | BURTON\_ADIPOGENESIS\_2 | 63 | 8 | 12187 | 179 | Rhou,Map3k6,Ptges,Rhoj,Timp1,Col4a1,Clic4,Xdh | | 3.602e-06 | -12.53 | MAHAJAN\_RESPONSE\_TO\_IL1A\_UP | MSigDB lists | MAHAJAN\_RESPONSE\_TO\_IL1A\_UP | 63 | 8 | 12187 | 179 | Cxcl1,Trim25,Thbs1,Ifit2,Il6,Oasl1,Ripk1,Il1a | | 3.624e-06 | -12.53 | HIN200/IF120x | interpro domains | IPR004021 | 8 | 4 | 13788 | 212 | Ifi209,Ifi211,Ifi204,Ifi207 | | 3.631e-06 | -12.53 | GO\_POSITIVE\_REGULATION\_OF\_CATALYTIC\_ACTIVITY | MSigDB lists | GO\_POSITIVE\_REGULATION\_OF\_CATALYTIC\_ACTIVITY | 1198 | 38 | 12187 | 179 | Gadd45b,Ripk1,Ccl12,Rhoc,Ccl2,Ccl4,Ccnd2,Map3k8,Rgs16,S100a8,Iqgap1,Ifi211,Pik3r5,Gadd45g,Dnajb1,Hspa5,Thbs1,Ptpn1,Map3k6,Sdc4,Tnf,Tnfsf10,Xdh,S100a9,A2m,Fzd4,C5ar1,Cflar,Ifi207,Ifi204,Cdkn1a,Ifi209,Errfi1,Csf2rb,Sele,Plek,Icam1,Saa1 | | 3.716e-06 | -12.50 | GO\_REGULATION\_OF\_ACUTE\_INFLAMMATORY\_RESPONSE | MSigDB lists | GO\_REGULATION\_OF\_ACUTE\_INFLAMMATORY\_RESPONSE | 45 | 7 | 12187 | 179 | Tnf,Il6,A2m,C3ar1,Ptgs2,C5ar1,Osmr | | 3.745e-06 | -12.50 | cellular response to toxic substance | biological process | GO:0097237 | 124 | 11 | 13711 | 214 | Tnf,Tnfaip3,Il6,Ccl7,Ripk1,Lcn2,Nfkb1,S100a8,Hp,Nfe2l2,S100a9 | | 3.809e-06 | -12.48 | GSE2405\_0H\_VS\_12H\_A\_PHAGOCYTOPHILUM\_STIM\_NEUTROPHIL\_UP | MSigDB lists | GSE2405\_0H\_VS\_12H\_A\_PHAGOCYTOPHILUM\_STIM\_NEUTROPHIL\_UP | 159 | 12 | 12187 | 179 | Tap1,Irf9,Slfn5,Nfkb1,Gadd45g,Ifit3b,Cmpk2,Irf7,Oasl1,Ccnd2,Parp14,Xaf1 | | 3.809e-06 | -12.48 | GSE360\_DC\_VS\_MAC\_DN | MSigDB lists | GSE360\_DC\_VS\_MAC\_DN | 159 | 12 | 12187 | 179 | S100a9,Irf7,Cd14,Ifi211,Pygm,Maff,Ifi209,Cp,Ifi207,Ifi204,Nfkb1,Atf3 | | 3.825e-06 | -12.47 | negative regulation of cell death | biological process | GO:0060548 | 859 | 32 | 13711 | 214 | Ier3,Plaur,Sgk1,Ptgs2,Nfkb1,Birc3,Timp1,Ccnd2,Pik3r1,Socs3,Thbs1,Cdkn1a,Il6,Sox11,Mt1,Icam1,Bcl3,Angptl4,Sgk3,Nfe2l2,Ccl12,Ptpn1,Hspa5,C5ar1,Cflar,Ripk1,Tnfaip8,Csf3,Hspb1,Il1rn,Tnf,Tnfaip3 | | 3.836e-06 | -12.47 | HIN | pfam domains | PF02760 | 8 | 4 | 12881 | 201 | Ifi211,Ifi204,Ifi207,Ifi209 | | 3.919e-06 | -12.45 | regulation of endopeptidase activity | biological process | GO:0052548 | 264 | 16 | 13711 | 214 | Casp4,Tnf,Il6,Tnfsf10,Xdh,Thbs1,Cflar,Tnfaip8,Timp1,Birc3,A2m,S100a8,Serpina3f,S100a9,Ptgs2,Plaur | | 4.079e-06 | -12.41 | GO\_NEGATIVE\_REGULATION\_OF\_MOLECULAR\_FUNCTION | MSigDB lists | GO\_NEGATIVE\_REGULATION\_OF\_MOLECULAR\_FUNCTION | 790 | 29 | 12187 | 179 | Ptpn1,Irak3,Nfkb1,Thbs1,Hspa5,Sox11,Iqgap1,Tnfaip8,Ifi211,Angptl4,Il6,Gadd45b,Tnfaip3,Ifit2,Plaur,Errfi1,Hspb1,Ifi207,Hp,Ifi204,Timp1,Socs3,Cdkn1a,Ifi209,A2m,Nfkbia,Trib1,Birc3,Tnf | | 4.090e-06 | -12.41 | PID\_AMB2\_NEUTROPHILS\_PATHWAY | MSigDB lists | PID\_AMB2\_NEUTROPHILS\_PATHWAY | 30 | 6 | 12187 | 179 | Icam1,Nfkb1,Il6,Plaur,Tnf,Selp | | 4.090e-06 | -12.41 | AMIT\_EGF\_RESPONSE\_60\_MCF10A | MSigDB lists | AMIT\_EGF\_RESPONSE\_60\_MCF10A | 30 | 6 | 12187 | 179 | Trib1,Cxcl1,Ptgs2,Ier3,Il6,Tnfaip3 | | 4.090e-06 | -12.41 | BIOCARTA\_DEATH\_PATHWAY | MSigDB lists | BIOCARTA\_DEATH\_PATHWAY | 30 | 6 | 12187 | 179 | Cflar,Birc3,Nfkbia,Nfkb1,Ripk1,Tnfsf10 | | 4.090e-06 | -12.41 | ONO\_FOXP3\_TARGETS\_DN | MSigDB lists | ONO\_FOXP3\_TARGETS\_DN | 30 | 6 | 12187 | 179 | Casp4,Il4ra,Cdkn1a,Slfn3,Il17ra,Slfn4 | | 4.090e-06 | -12.41 | regulation of endothelial cell apoptotic process | biological process | GO:2000351 | 43 | 7 | 13711 | 214 | Angptl4,Nfe2l2,Thbs1,Ccl12,Tnf,Tnfaip3,Icam1 | | 4.215e-06 | -12.38 | regulation of cellular component movement | biological process | GO:0051270 | 863 | 32 | 13711 | 214 | Nfe2l2,Selp,Msn,C5ar1,Clic4,Hspa5,Iqgap1,Ccl12,Cxcl16,Hspb1,Sele,Il1rn,C3ar1,Adamts9,Rhoc,Robo4,Ccl2,Ccl4,Ptgs2,Timp1,Fam107a,Rhoj,Sdc4,Adamts1,Thbs1,Trib1,Pik3r1,Lgals9,P2ry6,Icam1,Il1a,Akap12 | | 4.337e-06 | -12.35 | GSE11961\_FOLLICULAR\_BCELL\_VS\_MEMORY\_BCELL\_DAY7\_UP | MSigDB lists | GSE11961\_FOLLICULAR\_BCELL\_VS\_MEMORY\_BCELL\_DAY7\_UP | 161 | 12 | 12187 | 179 | Pik3ap1,Parp9,Irf9,Herc6,Rnf213,Samd9l,Ifit3b,Cmpk2,Pik3r5,Irf7,Xaf1,Trib1 | | 4.360e-06 | -12.34 | MODULE\_6 | MSigDB lists | MODULE\_6 | 284 | 16 | 12187 | 179 | Cd14,Lcn2,Ier3,Atf3,Cdkn1a,Tnfsf10,S100a8,S100a9,Gem,Ccl12,Fam107a,Tnfaip3,Gadd45b,Tgm2,Il4ra,Maff | | 4.413e-06 | -12.33 | positive regulation of catalytic activity | biological process | GO:0043085 | 1081 | 37 | 13711 | 214 | Wfdc21,Irgm2,S100a9,Ccl12,S100a8,Iqgap1,Ptpn1,Dnajb1,Rhoc,Ripk1,Cflar,Ncf1,Il1rn,Sele,Ccl2,Xdh,Tnfsf10,Plek,Tnf,Pik3r5,Ccl4,Sdc4,Fzd4,Map3k6,Map3k8,Lgals9,Gadd45b,Ccnd2,Gadd45g,Trib1,Ccl7,Dtx3l,Thbs1,Cdkn1a,Rgs16,Icam1,Casp4 | | 4.415e-06 | -12.33 | GSE6269\_HEALTHY\_VS\_E\_COLI\_INF\_PBMC\_DN | MSigDB lists | GSE6269\_HEALTHY\_VS\_E\_COLI\_INF\_PBMC\_DN | 109 | 10 | 12187 | 179 | Trim25,Irak3,Plek,Plaur,Msn,Tagln2,Cxcl1,Cybb,Bach1,Ccl2 | | 4.514e-06 | -12.31 | TARTE\_PLASMA\_CELL\_VS\_PLASMABLAST\_UP | MSigDB lists | TARTE\_PLASMA\_CELL\_VS\_PLASMABLAST\_UP | 252 | 15 | 12187 | 179 | S100a9,S100a8,Nfkb2,Runx1,Birc3,Il4ra,Nfkbia,Tnfaip3,Hspb1,Ifi44,Vcam1,Fstl1,Hp,Col4a1,Lcn2 | | 4.575e-06 | -12.29 | GO\_POSITIVE\_REGULATION\_OF\_GENE\_EXPRESSION | MSigDB lists | GO\_POSITIVE\_REGULATION\_OF\_GENE\_EXPRESSION | 1307 | 40 | 12187 | 179 | S100a8,Sbno2,Csf3,Banp,Ifi211,Bcl3,Arid5b,Ripk1,Nfe2l2,Il6,Maff,Pik3r1,Spi1,Irak3,Irf2,Nfkb1,Il1a,Cebpd,Sox11,Hspa5,Thbs1,Nfkb2,Cflar,S100a9,Fzd4,Irf7,Bach1,Tnf,Runx1,Nfkbia,Trim25,Icam1,Zfp36,Msn,Tnip1,Ifi204,Ifi207,Atf3,Ifi209,Zbtb16 | | 4.593e-06 | -12.29 | regulation of leukocyte migration | biological process | GO:0002685 | 152 | 12 | 13711 | 214 | C5ar1,Ccl12,Ccl4,Msn,Selp,Il1a,Icam1,Ccl2,Thbs1,C3ar1,Sele,Lgals9 | | 4.605e-06 | -12.29 | GO\_NEGATIVE\_REGULATION\_OF\_PROTEIN\_METABOLIC\_PROCESS | MSigDB lists | GO\_NEGATIVE\_REGULATION\_OF\_PROTEIN\_METABOLIC\_PROCESS | 839 | 30 | 12187 | 179 | Tnfaip3,Il6,Gadd45b,Spi1,Tnfaip8,Ifi211,Banp,Bcl3,Nfkb1,Thbs1,Irak3,Ptpn1,Isg15,Tnf,Birc3,Trib1,Xdh,A2m,Timp1,Ifi204,Ifi207,Atf3,Cdkn1a,Ifi209,Socs3,Hspb1,Zfp36,Errfi1,Plaur,Tnip1 | | 4.625e-06 | -12.28 | GSE2197\_IMMUNOSUPPRESSIVE\_DNA\_VS\_UNTREATED\_IN\_DC\_UP | MSigDB lists | GSE2197\_IMMUNOSUPPRESSIVE\_DNA\_VS\_UNTREATED\_IN\_DC\_UP | 162 | 12 | 12187 | 179 | Atf3,Socs3,Ifi44,Irf9,Zfp36,Pik3ap1,Icam1,Ptgs2,Hcar2,Birc3,Rgs16,Gbp7 | | 4.625e-06 | -12.28 | GSE19772\_CTRL\_VS\_HCMV\_INF\_MONOCYTES\_UP | MSigDB lists | GSE19772\_CTRL\_VS\_HCMV\_INF\_MONOCYTES\_UP | 162 | 12 | 12187 | 179 | Xdh,Rgs16,Irgm2,Tor3a,Usp18,Ifit2,Irf9,Tap1,Saa1,Nfkb1,Hp,Akap12 | | 4.625e-06 | -12.28 | GSE22601\_DOUBLE\_POSITIVE\_VS\_CD8\_SINGLE\_POSITIVE\_THYMOCYTE\_UP | MSigDB lists | GSE22601\_DOUBLE\_POSITIVE\_VS\_CD8\_SINGLE\_POSITIVE\_THYMOCYTE\_UP | 162 | 12 | 12187 | 179 | Nfkbie,Trim56,Ccnd2,Il4ra,Tor3a,Parp14,Irak3,Trim25,Il17ra,Irf9,Gadd45g,Ier3 | | 4.627e-06 | -12.28 | regulation of cysteine-type endopeptidase activity involved in apoptotic process | biological process | GO:0043281 | 179 | 13 | 13711 | 214 | Plaur,Ptgs2,S100a9,S100a8,Birc3,Cflar,Tnfaip8,Thbs1,Il6,Tnfsf10,Xdh,Casp4,Tnf | | 4.662e-06 | -12.28 | GO\_REGULATION\_OF\_MYELOID\_CELL\_DIFFERENTIATION | MSigDB lists | GO\_REGULATION\_OF\_MYELOID\_CELL\_DIFFERENTIATION | 135 | 11 | 12187 | 179 | Zbtb16,Isg15,Ripk1,Runx1,Tnf,Pik3r1,Trib1,Nfkbia,Spi1,Csf3,Irf7 | | 4.662e-06 | -12.28 | GO\_RESPONSE\_TO\_STARVATION | MSigDB lists | GO\_RESPONSE\_TO\_STARVATION | 135 | 11 | 12187 | 179 | Zfp36,Rrp8,Ifi211,Ifi207,Ifi204,Nfe2l2,Atf3,Hspa5,Cdkn1a,Ifi209,Fstl1 | | 4.695e-06 | -12.27 | acute-phase response | biological process | GO:0006953 | 17 | 5 | 13711 | 214 | Il6,Saa1,Hp,Il1rn,Il1a | | 4.695e-06 | -12.27 | negative regulation of viral entry into host cell | biological process | GO:0046597 | 17 | 5 | 13711 | 214 | Trim25,Trim56,Ifitm2,Ifitm6,Ifitm3 | | 4.734e-06 | -12.26 | GO\_REGULATION\_OF\_PROTEIN\_SERINE\_THREONINE\_KINASE\_ACTIVITY | MSigDB lists | GO\_REGULATION\_OF\_PROTEIN\_SERINE\_THREONINE\_KINASE\_ACTIVITY | 391 | 19 | 12187 | 179 | Trib1,Tnf,Map3k8,Ccnd2,Gadd45b,Ripk1,Tnfaip3,Fzd4,Pik3r5,C5ar1,Iqgap1,Thbs1,Cdkn1a,Gadd45g,Saa1,Map3k6,Ptpn1,Hspb1,Irak3 | | 4.929e-06 | -12.22 | GSE15930\_STIM\_VS\_STIM\_AND\_IL12\_48H\_CD8\_T\_CELL\_DN | MSigDB lists | GSE15930\_STIM\_VS\_STIM\_AND\_IL12\_48H\_CD8\_T\_CELL\_DN | 163 | 12 | 12187 | 179 | Socs3,Usp18,Casp4,Map3k8,Il6,Ksr1,Gadd45g,Bcl3,Selp,Ifitm3,Gem,Tnfsf10 | | 4.935e-06 | -12.22 | GO\_CELLULAR\_RESPONSE\_TO\_OXYGEN\_CONTAINING\_COMPOUND | MSigDB lists | GO\_CELLULAR\_RESPONSE\_TO\_OXYGEN\_CONTAINING\_COMPOUND | 627 | 25 | 12187 | 179 | Cd14,P2ry6,Cxcl16,Ptpn1,Nfkb1,Csf3,Cmpk2,Sbno2,Pik3r1,Spi1,Nfe2l2,Il6,Ccl12,Tnfaip3,Ptgs2,Icam1,Msn,Errfi1,Adamts1,Col4a1,Tnip3,Fzd4,Tnf,Nfkbia,Casp4 | | 4.973e-06 | -12.21 | GO\_REGULATION\_OF\_PHOSPHORUS\_METABOLIC\_PROCESS | MSigDB lists | GO\_REGULATION\_OF\_PHOSPHORUS\_METABOLIC\_PROCESS | 1214 | 38 | 12187 | 179 | Xdh,Fzd4,C5ar1,Trib1,Sdc4,Tnf,Plaur,Errfi1,Plek,Hspb1,Icam1,Tnip1,Saa1,Atf3,Ksr1,Socs3,Cdkn1a,Iqgap1,Pik3r5,Csf3,Il6,Gadd45b,Ripk1,Ccl12,Tnfaip3,Ccl2,Ccl4,Pik3r1,Ccnd2,Map3k8,Ptpn1,Irak3,Map3k6,Il1a,Akap12,Gadd45g,Hspa5,Thbs1 | | 4.988e-06 | -12.21 | MODULE\_92 | MSigDB lists | MODULE\_92 | 87 | 9 | 12187 | 179 | Il6,Ccl12,Il1a,Ccl2,Ccl4,Tnfsf10,S100a9,Csf3,Isg15 | | 5.009e-06 | -12.20 | GSE46606\_IRF4HIGH\_VS\_IRF4MID\_CD40L\_IL2\_IL5\_DAY1\_STIMULATED\_BCELL\_DN | MSigDB lists | GSE46606\_IRF4HIGH\_VS\_IRF4MID\_CD40L\_IL2\_IL5\_DAY1\_STIMULATED\_BCELL\_DN | 136 | 11 | 12187 | 179 | Gbp3,Cmpk2,Ccl4,Ccnd2,Gadd45b,Slfn9,Isg15,Parp9,Hspa5,Atf3,Samd9l | | 5.010e-06 | -12.20 | BURTON\_ADIPOGENESIS\_1 | MSigDB lists | BURTON\_ADIPOGENESIS\_1 | 31 | 6 | 12187 | 179 | Ier3,Il6,Cebpd,Sgk1,Maff,Errfi1 | | 5.010e-06 | -12.20 | GO\_POSITIVE\_REGULATION\_OF\_INTERLEUKIN\_1\_PRODUCTION | MSigDB lists | GO\_POSITIVE\_REGULATION\_OF\_INTERLEUKIN\_1\_PRODUCTION | 31 | 6 | 12187 | 179 | Hspb1,Ifi204,Ifi207,Ifi209,Saa1,Ifi211 | | 5.010e-06 | -12.20 | GO\_INTRINSIC\_APOPTOTIC\_SIGNALING\_PATHWAY\_IN\_RESPONSE\_TO\_DNA\_DAMAGE\_BY\_P53\_CLASS\_MEDIATOR | MSigDB lists | GO\_INTRINSIC\_APOPTOTIC\_SIGNALING\_PATHWAY\_IN\_RESPONSE\_TO\_DNA\_DAMAGE\_BY\_P53\_CLASS\_MEDIATOR | 31 | 6 | 12187 | 179 | Ifi207,Ifi204,Cdkn1a,Ifi209,Bcl3,Ifi211 | | 5.024e-06 | -12.20 | KEGG\_RIG\_I\_LIKE\_RECEPTOR\_SIGNALING\_PATHWAY | MSigDB lists | KEGG\_RIG\_I\_LIKE\_RECEPTOR\_SIGNALING\_PATHWAY | 47 | 7 | 12187 | 179 | Trim25,Irf7,Isg15,Ripk1,Nfkb1,Nfkbia,Tnf | | 5.145e-06 | -12.18 | GO\_INTRINSIC\_APOPTOTIC\_SIGNALING\_PATHWAY\_IN\_RESPONSE\_TO\_DNA\_DAMAGE | MSigDB lists | GO\_INTRINSIC\_APOPTOTIC\_SIGNALING\_PATHWAY\_IN\_RESPONSE\_TO\_DNA\_DAMAGE | 66 | 8 | 12187 | 179 | Ifi211,Bcl3,Cdkn1a,Tnf,Ifi209,Pik3r1,Ifi204,Ifi207 | | 5.266e-06 | -12.15 | IRF7\_01 | MSigDB lists | IRF7\_01 | 193 | 13 | 12187 | 179 | Irf2,Cxcl16,Tap1,Isg15,Stx11,Angptl4,Dtx3l,Parp12,Oasl1,Xaf1,Usp18,Ifit2,Map3k8 | | 5.392e-06 | -12.13 | GO\_REGULATION\_OF\_CELLULAR\_COMPONENT\_MOVEMENT | MSigDB lists | GO\_REGULATION\_OF\_CELLULAR\_COMPONENT\_MOVEMENT | 589 | 24 | 12187 | 179 | Robo4,C3ar1,C5ar1,Sdc4,Trib1,Tnf,Msn,Clic4,Hspb1,Icam1,Ptgs2,Timp1,Cxcl1,Il6,Ccl12,Ccl4,Pik3r1,Cxcl16,P2ry6,Sgk3,Selp,Hspa5,Sgk1,Thbs1 | | 5.425e-06 | -12.12 | HELLER\_HDAC\_TARGETS\_SILENCED\_BY\_METHYLATION\_DN | MSigDB lists | HELLER\_HDAC\_TARGETS\_SILENCED\_BY\_METHYLATION\_DN | 224 | 14 | 12187 | 179 | Hspb1,Ifi209,Phf11b,Dnajb1,Ifi207,Ifi204,Atf3,Cflar,Bcl3,Ifi211,Phf11d,Maff,Ccnd2,Trib1 | | 5.592e-06 | -12.09 | GSE17721\_PAM3CSK4\_VS\_GADIQUIMOD\_12H\_BMDC\_DN | MSigDB lists | GSE17721\_PAM3CSK4\_VS\_GADIQUIMOD\_12H\_BMDC\_DN | 165 | 12 | 12187 | 179 | Parp9,Irf2,S100a8,Ptges,Gbp6,Gbp4,Tiparp,Rhoc,Parp14,Ccl2,Ccnd2,Sgk1 | | 5.592e-06 | -12.09 | GSE10325\_LUPUS\_CD4\_TCELL\_VS\_LUPUS\_MYELOID\_DN | MSigDB lists | GSE10325\_LUPUS\_CD4\_TCELL\_VS\_LUPUS\_MYELOID\_DN | 165 | 12 | 12187 | 179 | Cybb,Rassf4,Igsf6,C5ar1,S100a9,Il1rn,Cebpd,Rbm47,Clic4,Plaur,Csf2rb,Cd14 | | 5.592e-06 | -12.09 | GSE2770\_TGFB\_AND\_IL4\_ACT\_VS\_ACT\_CD4\_TCELL\_48H\_UP | MSigDB lists | GSE2770\_TGFB\_AND\_IL4\_ACT\_VS\_ACT\_CD4\_TCELL\_48H\_UP | 165 | 12 | 12187 | 179 | Cdkn1a,Il1a,Tnip1,Sdc4,Rhou,Tnf,Nfe2l2,Tubb6,Banp,Pik3r5,Tnfaip8,Rtp4 | | 5.610e-06 | -12.09 | defense response to Gram-positive bacterium | biological process | GO:0050830 | 45 | 7 | 13711 | 214 | Tnf,C5ar1,Gbp3,Tnfaip8,Ncf1,Gbp6,Gbp7 | | 5.769e-06 | -12.06 | GSE6269\_FLU\_VS\_STAPH\_AUREUS\_INF\_PBMC\_DN | MSigDB lists | GSE6269\_FLU\_VS\_STAPH\_AUREUS\_INF\_PBMC\_DN | 138 | 11 | 12187 | 179 | Timp1,Il1rn,Socs3,Spi1,Cdkn1a,Plaur,Sbno2,Icam1,S100a8,Plek,S100a9 | | 5.770e-06 | -12.06 | JECHLINGER\_EPITHELIAL\_TO\_MESENCHYMAL\_TRANSITION\_UP | MSigDB lists | JECHLINGER\_EPITHELIAL\_TO\_MESENCHYMAL\_TRANSITION\_UP | 67 | 8 | 12187 | 179 | Ccl12,Ifit3b,Gbp3,S100a8,Bcl3,Irf7,Isg15,Ifitm3 | | 5.809e-06 | -12.06 | GO\_INTERFERON\_GAMMA\_MEDIATED\_SIGNALING\_PATHWAY | MSigDB lists | GO\_INTERFERON\_GAMMA\_MEDIATED\_SIGNALING\_PATHWAY | 48 | 7 | 12187 | 179 | Irf2,Irf9,Trim25,Icam1,Irf7,Oasl1,Vcam1 | | 5.890e-06 | -12.04 | host cell part | cellular component | GO:0033643 | 18 | 5 | 13825 | 212 | Irgm2,Gbp6,Gbp3,Iigp1,Gbp7 | | 5.896e-06 | -12.04 | RUTELLA\_RESPONSE\_TO\_HGF\_VS\_CSF2RB\_AND\_IL4\_DN | MSigDB lists | RUTELLA\_RESPONSE\_TO\_HGF\_VS\_CSF2RB\_AND\_IL4\_DN | 195 | 13 | 12187 | 179 | Cebpd,Gadd45b,Ms4a6d,Sdc4,Ccl2,Cdkn1a,Runx1,Birc3,Hspb1,Tnfaip8,Zfp36,Cflar,Map3k6 | | 5.951e-06 | -12.03 | GSE22886\_NAIVE\_CD8\_TCELL\_VS\_MONOCYTE\_DN | MSigDB lists | GSE22886\_NAIVE\_CD8\_TCELL\_VS\_MONOCYTE\_DN | 166 | 12 | 12187 | 179 | Ms4a6d,Bach1,Cybb,Cebpd,S100a8,Igsf6,Irak3,Csf2rb,C5ar1,Cd14,S100a9,Bcl3 | | 5.951e-06 | -12.03 | GSE15330\_HSC\_VS\_LYMPHOID\_PRIMED\_MULTIPOTENT\_PROGENITOR\_UP | MSigDB lists | GSE15330\_HSC\_VS\_LYMPHOID\_PRIMED\_MULTIPOTENT\_PROGENITOR\_UP | 166 | 12 | 12187 | 179 | Ifitm2,Isg15,Ifitm3,Zfp36,Icam1,Irf9,Clic4,Ptpn1,Sgk1,Socs3,Birc3,Parp12 | | 6.001e-06 | -12.02 | receptor regulator activity | molecular function | GO:0030545 | 273 | 16 | 13516 | 211 | Tnfsf10,Timp1,Cxcl1,Csf3,Il1rn,Il1a,Ccl7,Ccl12,Tnf,Ccl2,Retnlg,Cxcl9,Il6,Ccl4,Saa1,Cxcl16 | | 6.011e-06 | -12.02 | GO\_REGULATION\_OF\_HEMOPOIESIS | MSigDB lists | GO\_REGULATION\_OF\_HEMOPOIESIS | 226 | 14 | 12187 | 179 | Isg15,Zbtb16,Irf7,Csf3,Trib1,Spi1,Nfkbia,Tnf,Runx1,Pik3r1,Il4ra,Ripk1,Il6,Nfe2l2 | | 6.028e-06 | -12.02 | MODULE\_456 | MSigDB lists | MODULE\_456 | 89 | 9 | 12187 | 179 | Stx11,Tnfaip3,Il6,Tnf,Cdkn1a,Hspa5,Nfkbia,Sele,Nfkb2 | | 6.186e-06 | -11.99 | GSE22611\_MUTANT\_NOD2\_VS\_CTRL\_TRANSDUCED\_HEK293T\_CELL\_UP | MSigDB lists | GSE22611\_MUTANT\_NOD2\_VS\_CTRL\_TRANSDUCED\_HEK293T\_CELL\_UP | 139 | 11 | 12187 | 179 | Zbtb16,Icam1,Clic4,Ptgs2,Rhoc,Il6,Gadd45b,Map3k8,Tnf,Csf3,Cxcl1 | | 6.186e-06 | -11.99 | GSE9960\_GRAM\_POS\_VS\_GRAM\_NEG\_AND\_POS\_SEPSIS\_PBMC\_DN | MSigDB lists | GSE9960\_GRAM\_POS\_VS\_GRAM\_NEG\_AND\_POS\_SEPSIS\_PBMC\_DN | 139 | 11 | 12187 | 179 | Bcl3,Ncf1,A2m,Tnip1,Nfkbie,Nfkb2,Birc3,Lcn2,Rasip1,Hp,Il1a | | 6.246e-06 | -11.98 | regulation of peptidase activity | biological process | GO:0052547 | 306 | 17 | 13711 | 214 | Thbs1,Cflar,Tnfaip8,Casp4,Tnf,Il6,Tnfsf10,Xdh,Serpina3f,S100a9,Ptgs2,Wfdc21,Plaur,Timp1,Birc3,A2m,S100a8 | | 6.331e-06 | -11.97 | HALLMARK\_IL2\_STAT5\_SIGNALING | MSigDB lists | HALLMARK\_IL2\_STAT5\_SIGNALING | 167 | 12 | 12187 | 179 | Ifitm3,Selp,Nfkbiz,Rgs16,Tnfsf10,Gbp3,Gadd45b,Maff,Il4ra,Ccnd2,Map3k8,Tgm2 | | 6.361e-06 | -11.97 | YAN\_ESCAPE\_FROM\_ANOIKIS | MSigDB lists | YAN\_ESCAPE\_FROM\_ANOIKIS | 19 | 5 | 12187 | 179 | Vcam1,Irf7,Slfn4,Nfkb1,Slfn3 | | 6.361e-06 | -11.97 | WORSCHECH\_TUMOR\_EVASION\_AND\_TOLEROGENICITY\_UP | MSigDB lists | WORSCHECH\_TUMOR\_EVASION\_AND\_TOLEROGENICITY\_UP | 19 | 5 | 12187 | 179 | Nfkb1,Il6,Ccl12,Irf7,Ccl4 | | 6.361e-06 | -11.97 | GO\_CHEMOKINE\_ACTIVITY | MSigDB lists | GO\_CHEMOKINE\_ACTIVITY | 19 | 5 | 12187 | 179 | Ccl12,Cxcl16,Ccl2,Cxcl1,Ccl4 | | 6.444e-06 | -11.95 | Guanylate-bd/ATL\_C | interpro domains | IPR003191 | 9 | 4 | 13788 | 212 | Gbp4,Gbp3,Gbp7,Gbp6 | | 6.444e-06 | -11.95 | HIN-200 | interpro domains | IPR040205 | 9 | 4 | 13788 | 212 | Ifi209,Ifi204,Ifi211,Ifi207 | | 6.616e-06 | -11.93 | GSE18791\_CTRL\_VS\_NEWCASTLE\_VIRUS\_DC\_2H\_DN | MSigDB lists | GSE18791\_CTRL\_VS\_NEWCASTLE\_VIRUS\_DC\_2H\_DN | 114 | 10 | 12187 | 179 | Ptgs2,Isg15,Gem,Irf7,Errfi1,Ifit2,Tnf,Ifit3b,Dnajb1,Gadd45b | | 6.668e-06 | -11.92 | regulation of ERK1 and ERK2 cascade | biological process | GO:0070372 | 244 | 15 | 13711 | 214 | Tnip1,P2ry6,Cflar,Ccl7,Il6,Atf3,Errfi1,Ccl2,Icam1,Il1a,Akap12,Ccl4,Ccl12,C5ar1,Ptpn1 | | 6.694e-06 | -11.91 | SANA\_RESPONSE\_TO\_IFNG\_UP | MSigDB lists | SANA\_RESPONSE\_TO\_IFNG\_UP | 49 | 7 | 12187 | 179 | Parp14,Samd9l,Rnf213,Dtx3l,Tnfsf10,Parp9,Gbp3 | | 6.732e-06 | -11.91 | GSE43700\_UNTREATED\_VS\_IL10\_TREATED\_PBMC\_DN | MSigDB lists | GSE43700\_UNTREATED\_VS\_IL10\_TREATED\_PBMC\_DN | 168 | 12 | 12187 | 179 | Ccl4,Map3k8,Maff,Il6,Gbp7,Xdh,Socs3,Ifit3b,Cebpd,Gadd45g,Selp,Errfi1 | | 6.820e-06 | -11.90 | GBP\_C | pfam domains | PF02841 | 9 | 4 | 12881 | 201 | Gbp6,Gbp7,Gbp4,Gbp3 | | 6.964e-06 | -11.87 | ISRE\_01 | MSigDB lists | ISRE\_01 | 198 | 13 | 12187 | 179 | Isg15,Irf2,Irf9,Ifi44,Thbs1,Ifit3b,Dtx3l,Angptl4,Rtp4,Rbm39,Ifit2,Xaf1,Usp18 | | 7.155e-06 | -11.85 | HALLMARK\_EPITHELIAL\_MESENCHYMAL\_TRANSITION | MSigDB lists | HALLMARK\_EPITHELIAL\_MESENCHYMAL\_TRANSITION | 169 | 12 | 12187 | 179 | Tnfaip3,Gadd45b,Il6,Tgm2,Sdc4,Gem,Timp1,Col4a1,Thbs1,Vcam1,Fstl1,Plaur | | 7.185e-06 | -11.84 | GO\_POSITIVE\_REGULATION\_OF\_PROTEIN\_MODIFICATION\_PROCESS | MSigDB lists | GO\_POSITIVE\_REGULATION\_OF\_PROTEIN\_MODIFICATION\_PROCESS | 858 | 30 | 12187 | 179 | Ccl2,Ccl4,Ccnd2,Map3k8,Gadd45b,Il6,Ccl12,Ripk1,Pik3r5,Csf3,Iqgap1,Thbs1,Hspa5,Gadd45g,Il1a,Map3k6,Ptpn1,Sdc4,Birc3,Tnf,Fzd4,C5ar1,Xdh,Socs3,Cdkn1a,Ksr1,Saa1,Plaur,Plek,Icam1 | | 7.215e-06 | -11.84 | HARRIS\_HYPOXIA | MSigDB lists | HARRIS\_HYPOXIA | 69 | 8 | 12187 | 179 | Plaur,Ptgs2,Cp,Nfkb1,Il6,Ccl12,Cdkn1a,Tgm2 | | 7.355e-06 | -11.82 | PID\_HIV\_NEF\_PATHWAY | MSigDB lists | PID\_HIV\_NEF\_PATHWAY | 33 | 6 | 12187 | 179 | Nfkb1,Ripk1,Tnf,Cflar,Birc3,Nfkbia | | 7.528e-06 | -11.80 | HIN\_200 | prosite domains | PS50834 | 8 | 4 | 8845 | 164 | Ifi211,Ifi204,Ifi209,Ifi207 | | 7.587e-06 | -11.79 | Osteoclast differentiation | KEGG pathways | mmu04380 | 105 | 11 | 5248 | 107 | Il1a,Cybb,Nfkb2,Socs3,Nfkbia,Ncf1,Spi1,Nfkb1,Tnf,Pik3r1,Irf9 | | 7.587e-06 | -11.79 | Osteoclast differentiation | KEGG pathways | ko04380 | 105 | 11 | 5248 | 107 | Cybb,Nfkb2,Tnf,Irf9,Socs3,Ncf1,Nfkb1,Il1a,Pik3r1,Nfkbia,Spi1 | | 7.601e-06 | -11.79 | GSE22140\_GERMFREE\_VS\_SPF\_MOUSE\_CD4\_TCELL\_DN | MSigDB lists | GSE22140\_GERMFREE\_VS\_SPF\_MOUSE\_CD4\_TCELL\_DN | 170 | 12 | 12187 | 179 | Ifitm2,Timp1,Nfkb1,Ier3,Ifi44,Thbs1,Ifit3b,Olfml2b,Oasl1,Pik3r1,Ifit2,Ccl4 | | 7.601e-06 | -11.79 | GSE17721\_CTRL\_VS\_CPG\_6H\_BMDC\_DN | MSigDB lists | GSE17721\_CTRL\_VS\_CPG\_6H\_BMDC\_DN | 170 | 12 | 12187 | 179 | Slfn3,Tap1,Tiparp,Znfx1,Atf3,Irgm2,Ptges,Slfn4,Pygm,Maff,Parp14,Ccl12 | | 7.601e-06 | -11.79 | GSE9006\_TYPE\_1\_DIABETES\_AT\_DX\_VS\_4MONTH\_POST\_DX\_PBMC\_UP | MSigDB lists | GSE9006\_TYPE\_1\_DIABETES\_AT\_DX\_VS\_4MONTH\_POST\_DX\_PBMC\_UP | 170 | 12 | 12187 | 179 | Hp,Stx11,Socs3,Sgk1,Hspa5,Zfp36,Tnfaip3,Cybb,Trib1,Nfkbia,Nfkb2,Cxcl1 | | 7.626e-06 | -11.78 | MODULE\_55 | MSigDB lists | MODULE\_55 | 520 | 22 | 12187 | 179 | Gadd45b,Fam107a,Ccl12,Trib1,Tgm2,Il4ra,Ccnd2,Tnfsf10,Sbno2,Xdh,S100a8,S100a9,Ier3,Lcn2,Col4a1,Gadd45g,Cp,Hp,Il1rn,Sele,Cd14,Isg15 | | 7.688e-06 | -11.78 | GNF2\_INPP5D | MSigDB lists | GNF2\_INPP5D | 50 | 7 | 12187 | 179 | Phf11b,Ifi209,Ifi207,Ifi204,Ifi211,Phf11d,Tap1 | | 7.809e-06 | -11.76 | GO\_RESPONSE\_TO\_INORGANIC\_SUBSTANCE | MSigDB lists | GO\_RESPONSE\_TO\_INORGANIC\_SUBSTANCE | 368 | 18 | 12187 | 179 | Ptges,Iqgap1,S100a8,Tnfaip3,Il6,Nfe2l2,Ptgs2,Cd14,Icam1,Clic4,Hspa5,Thbs1,Vcam1,Kcna5,Nfkb1,Hp,Cp,Il1a | | 8.045e-06 | -11.73 | GO\_POSITIVE\_REGULATION\_OF\_TYPE\_I\_INTERFERON\_PRODUCTION | MSigDB lists | GO\_POSITIVE\_REGULATION\_OF\_TYPE\_I\_INTERFERON\_PRODUCTION | 70 | 8 | 12187 | 179 | Nfkb2,Irf7,Cd14,Ifi211,Ifi207,Nfkb1,Ifi204,Ifi209 | | 8.045e-06 | -11.73 | HERNANDEZ\_ABERRANT\_MITOSIS\_BY\_DOCETACEL\_2NM\_UP | MSigDB lists | HERNANDEZ\_ABERRANT\_MITOSIS\_BY\_DOCETACEL\_2NM\_UP | 70 | 8 | 12187 | 179 | Ifi211,Ifi207,Ifi204,Il6,Ifi44,Sgk1,Pik3r1,Ifi209 | | 8.071e-06 | -11.73 | GSE36009\_WT\_VS\_NLRP10\_KO\_DC\_UP | MSigDB lists | GSE36009\_WT\_VS\_NLRP10\_KO\_DC\_UP | 171 | 12 | 12187 | 179 | Il6,Parp12,Il4ra,Slfn4,Gbp3,Gbp7,Trim56,Tiparp,Atf3,Pik3ap1,Slfn3,Slfn5 | | 8.071e-06 | -11.73 | BOQUEST\_STEM\_CELL\_DN | MSigDB lists | BOQUEST\_STEM\_CELL\_DN | 171 | 12 | 12187 | 179 | Sele,Apold1,Csf2rb,Selp,Stx11,Rasip1,Adamts9,Csf3,Cflar,Fam107a,Xaf1,Tgm2 | | 8.128e-06 | -11.72 | GO\_NEGATIVE\_REGULATION\_OF\_PEPTIDASE\_ACTIVITY | MSigDB lists | GO\_NEGATIVE\_REGULATION\_OF\_PEPTIDASE\_ACTIVITY | 143 | 11 | 12187 | 179 | A2m,Ifi211,Plaur,Tnfaip8,Birc3,Thbs1,Ifi209,Il6,Ifi207,Ifi204,Timp1 | | 8.380e-06 | -11.69 | GO\_CELLULAR\_EXTRAVASATION | MSigDB lists | GO\_CELLULAR\_EXTRAVASATION | 20 | 5 | 12187 | 179 | Ccl12,Sele,Vcam1,Selp,Tnf | | 8.380e-06 | -11.69 | TIAN\_TNF\_SIGNALING\_NOT\_VIA\_NFKB | MSigDB lists | TIAN\_TNF\_SIGNALING\_NOT\_VIA\_NFKB | 20 | 5 | 12187 | 179 | Maff,Ptges,Tiparp,Rgs16,Ier3 | | 8.567e-06 | -11.67 | GSE18893\_TCONV\_VS\_TREG\_2H\_CULTURE\_DN | MSigDB lists | GSE18893\_TCONV\_VS\_TREG\_2H\_CULTURE\_DN | 172 | 12 | 12187 | 179 | Socs3,Cdkn1a,Sgk1,Nfe2l2,Parp12,Ncf1,Ifitm3,Irgm2,Dtx3l,Ptpn1,Tap1,Icam1 | | 8.600e-06 | -11.66 | positive regulation of coagulation | biological process | GO:0050820 | 19 | 5 | 13711 | 214 | Selp,Nfe2l2,Thbs1,S100a9,Plek | | 8.600e-06 | -11.66 | response to copper ion | biological process | GO:0046688 | 19 | 5 | 13711 | 214 | Mt1,Nfe2l2,Il1a,Mt2,Cp | | 8.666e-06 | -11.66 | negative regulation of response to external stimulus | biological process | GO:0032102 | 281 | 16 | 13711 | 214 | Ier3,Nfkb1,A2m,Tap1,Ccl12,Socs3,Irak3,Thbs1,Parp14,Ctla2a,Trib1,Lgals9,Tnfaip3,Acod1,Zfp36,Cdkn1a | | 8.689e-06 | -11.65 | GSE360\_T\_GONDII\_VS\_B\_MALAYI\_LOW\_DOSE\_DC\_UP | MSigDB lists | GSE360\_T\_GONDII\_VS\_B\_MALAYI\_LOW\_DOSE\_DC\_UP | 144 | 11 | 12187 | 179 | Nfkb2,Cflar,Tnfaip3,Nfkbia,Trim25,Tap1,Ifitm3,Tagln2,Tnip1,Nfkb1,Ifit3b | | 8.689e-06 | -11.65 | GSE34156\_TLR1\_TLR2\_LIGAND\_VS\_NOD2\_AND\_TLR1\_TLR2\_LIGAND\_24H\_TREATED\_MONOCYTE\_UP | MSigDB lists | GSE34156\_TLR1\_TLR2\_LIGAND\_VS\_NOD2\_AND\_TLR1\_TLR2\_LIGAND\_24H\_TREATED\_MONOCYTE\_UP | 144 | 11 | 12187 | 179 | Msr1,Cybb,AA467197,C3ar1,Igsf6,Timp1,Cd14,Rbm47,Csf2rb,Cxcl16,Clic4 | | 8.702e-06 | -11.65 | cellular extravasation | biological process | GO:0045123 | 32 | 6 | 13711 | 214 | Ccl2,Selp,Tnf,Icam1,Vcam1,Sele | | 8.802e-06 | -11.64 | GO\_INTRINSIC\_APOPTOTIC\_SIGNALING\_PATHWAY\_BY\_P53\_CLASS\_MEDIATOR | MSigDB lists | GO\_INTRINSIC\_APOPTOTIC\_SIGNALING\_PATHWAY\_BY\_P53\_CLASS\_MEDIATOR | 51 | 7 | 12187 | 179 | Ifi209,Cdkn1a,Ifi204,Ifi207,Ifi211,Rrp8,Bcl3 | | 8.821e-06 | -11.64 | GO\_POSITIVE\_REGULATION\_OF\_MACROPHAGE\_CHEMOTAXIS | MSigDB lists | GO\_POSITIVE\_REGULATION\_OF\_MACROPHAGE\_CHEMOTAXIS | 10 | 4 | 12187 | 179 | Ccl12,C5ar1,C3ar1,Thbs1 | | 8.821e-06 | -11.64 | GO\_LEUKOCYTE\_ADHESION\_TO\_VASCULAR\_ENDOTHELIAL\_CELL | MSigDB lists | GO\_LEUKOCYTE\_ADHESION\_TO\_VASCULAR\_ENDOTHELIAL\_CELL | 10 | 4 | 12187 | 179 | Tnf,Selp,Vcam1,Sele | | 8.821e-06 | -11.64 | GO\_PERK\_MEDIATED\_UNFOLDED\_PROTEIN\_RESPONSE | MSigDB lists | GO\_PERK\_MEDIATED\_UNFOLDED\_PROTEIN\_RESPONSE | 10 | 4 | 12187 | 179 | Hspa5,Ccl12,Nfe2l2,Atf3 | | 8.821e-06 | -11.64 | SHIN\_B\_CELL\_LYMPHOMA\_CLUSTER\_5 | MSigDB lists | SHIN\_B\_CELL\_LYMPHOMA\_CLUSTER\_5 | 10 | 4 | 12187 | 179 | Tnf,Bcl3,Socs3,Il6 | | 8.821e-06 | -11.64 | REACTOME\_EXTRINSIC\_PATHWAY\_FOR\_APOPTOSIS | MSigDB lists | REACTOME\_EXTRINSIC\_PATHWAY\_FOR\_APOPTOSIS | 10 | 4 | 12187 | 179 | Ripk1,Tnfsf10,Cflar,Tnf | | 8.821e-06 | -11.64 | LI\_WILMS\_TUMOR\_ANAPLASTIC\_DN | MSigDB lists | LI\_WILMS\_TUMOR\_ANAPLASTIC\_DN | 10 | 4 | 12187 | 179 | Ifi211,Ifi209,Ifi207,Ifi204 | | 8.823e-06 | -11.64 | GRAHAM\_CML\_QUIESCENT\_VS\_NORMAL\_DIVIDING\_UP | MSigDB lists | GRAHAM\_CML\_QUIESCENT\_VS\_NORMAL\_DIVIDING\_UP | 34 | 6 | 12187 | 179 | Maff,Il1rn,Gadd45b,Ptgs2,Bcl3,Cxcl1 | | 8.823e-06 | -11.64 | STAMBOLSKY\_TARGETS\_OF\_MUTATED\_TP53\_DN | MSigDB lists | STAMBOLSKY\_TARGETS\_OF\_MUTATED\_TP53\_DN | 34 | 6 | 12187 | 179 | Irf9,Dtx3l,Isg15,Herc6,Irf7,Xaf1 | | 8.854e-06 | -11.63 | negative regulation of defense response | biological process | GO:0031348 | 162 | 12 | 13711 | 214 | Tnfaip3,Acod1,Zfp36,Parp14,Ctla2a,Socs3,Irak3,Lgals9,A2m,Nfkb1,Tap1,Ier3 | | 9.006e-06 | -11.62 | GSE9960\_GRAM\_NEG\_VS\_GRAM\_NEG\_AND\_POS\_SEPSIS\_PBMC\_UP | MSigDB lists | GSE9960\_GRAM\_NEG\_VS\_GRAM\_NEG\_AND\_POS\_SEPSIS\_PBMC\_UP | 118 | 10 | 12187 | 179 | Ifitm2,Ifitm3,Ifi44,Il4ra,Ifit2,Ifit3b,Usp18,Parp14,Xaf1,Samd9l | | 9.089e-06 | -11.61 | GSE22935\_WT\_VS\_MYD88\_KO\_MACROPHAGE\_UP | MSigDB lists | GSE22935\_WT\_VS\_MYD88\_KO\_MACROPHAGE\_UP | 173 | 12 | 12187 | 179 | Cybb,Tnfaip3,Nfkbie,Igsf6,Xdh,Fstl1,Stx11,Ptgs2,Ncf1,Il17ra,Rbm47,Csf2rb | | 9.140e-06 | -11.60 | negative regulation of response to biotic stimulus | biological process | GO:0002832 | 67 | 8 | 13711 | 214 | Acod1,Tnfaip3,Tap1,A2m,Irak3,Parp14,Trib1,Lgals9 | | 9.176e-06 | -11.60 | positive regulation of developmental process | biological process | GO:0051094 | 1253 | 40 | 13711 | 214 | Hspa5,Nfkbiz,Cxcl1,C5ar1,Iqgap1,S100a9,Nfe2l2,Noct,Cxcl9,Tnfaip3,Tnf,Zfp36,Cflar,Ripk1,Zbtb16,Adamts9,Csf3,Hspb1,Irak3,C3ar1,Ifi204,Fzd4,Isg15,Adamts1,Rhoj,Cebpd,Sgk1,Ptgs2,Runx1,Il1a,Sox11,Il4ra,Il6,Cybb,Msr1,Trib1,Socs3,Thbs1,Lgals9,Pik3r1 | | 9.284e-06 | -11.59 | GSE21360\_TERTIARY\_VS\_QUATERNARY\_MEMORY\_CD8\_TCELL\_DN | MSigDB lists | GSE21360\_TERTIARY\_VS\_QUATERNARY\_MEMORY\_CD8\_TCELL\_DN | 145 | 11 | 12187 | 179 | Lcn2,Ifitm2,Icam1,Nfkbia,Usp18,Sdc4,Cybb,Cflar,Pik3r5,S100a9,Cmpk2 | | 9.428e-06 | -11.57 | positive regulation of hemopoiesis | biological process | GO:1903708 | 163 | 12 | 13711 | 214 | Il6,Tnf,Il4ra,Lgals9,Csf3,Zbtb16,Trib1,Ripk1,Isg15,Cxcl1,Nfkbiz,Runx1 | | 9.472e-06 | -11.57 | REACTOME\_SIGNALING\_BY\_ILS | MSigDB lists | REACTOME\_SIGNALING\_BY\_ILS | 94 | 9 | 12187 | 179 | Irak3,Nfkb2,Csf2rb,Map3k8,Pik3r1,Socs3,Il1rn,Il1a,Il6 | | 9.639e-06 | -11.55 | GSE4748\_CTRL\_VS\_LPS\_STIM\_DC\_3H\_UP | MSigDB lists | GSE4748\_CTRL\_VS\_LPS\_STIM\_DC\_3H\_UP | 174 | 12 | 12187 | 179 | Irgm2,Angptl4,Rhoj,Iqgap1,Rtp4,Errfi1,Adamts1,Ifi44,Fstl1,Ifit3b,Akap12,Ripk1 | | 9.709e-06 | -11.54 | GO\_REGULATION\_OF\_REACTIVE\_OXYGEN\_SPECIES\_METABOLIC\_PROCESS | MSigDB lists | GO\_REGULATION\_OF\_REACTIVE\_OXYGEN\_SPECIES\_METABOLIC\_PROCESS | 119 | 10 | 12187 | 179 | Thbs1,Cdkn1a,Tnf,Hp,Il6,Nfe2l2,Ripk1,Ptgs2,Icam1,Xdh | | 9.747e-06 | -11.54 | GO\_POSITIVE\_REGULATION\_OF\_TRANSCRIPTION\_FROM\_RNA\_POLYMERASE\_II\_PROMOTER | MSigDB lists | GO\_POSITIVE\_REGULATION\_OF\_TRANSCRIPTION\_FROM\_RNA\_POLYMERASE\_II\_PROMOTER | 738 | 27 | 12187 | 179 | Cebpd,Il1a,Nfkb1,Hspa5,Sox11,Irf2,Nfe2l2,Il6,Ripk1,Spi1,Maff,Pik3r1,Sbno2,Bcl3,Ifi211,Csf3,Atf3,Ifi207,Ifi204,Ifi209,Tnip1,Bach1,Nfkbia,Tnf,Runx1,Nfkb2,Irf7 | | 9.914e-06 | -11.52 | GSE5589\_LPS\_VS\_LPS\_AND\_IL10\_STIM\_IL10\_KO\_MACROPHAGE\_45MIN\_DN | MSigDB lists | GSE5589\_LPS\_VS\_LPS\_AND\_IL10\_STIM\_IL10\_KO\_MACROPHAGE\_45MIN\_DN | 146 | 11 | 12187 | 179 | Irgm2,S100a8,Clic4,Ifit2,Msr1,Xaf1,Ifit3b,Samd9l,Cp,Gadd45g,Tnfaip3 | | 9.914e-06 | -11.52 | GSE360\_DC\_VS\_MAC\_M\_TUBERCULOSIS\_UP | MSigDB lists | GSE360\_DC\_VS\_MAC\_M\_TUBERCULOSIS\_UP | 146 | 11 | 12187 | 179 | Trib1,Ccl4,Il6,Gadd45b,Oasl1,Ripk1,Ccl12,Irf9,Ifit3b,Nfkb1,Il1rn | | 1.005e-05 | -11.51 | GO\_I\_KAPPAB\_KINASE\_NF\_KAPPAB\_SIGNALING | MSigDB lists | GO\_I\_KAPPAB\_KINASE\_NF\_KAPPAB\_SIGNALING | 52 | 7 | 12187 | 179 | Nfkb1,Ripk1,Nfkb2,Birc3,Tnf,Bcl3,Cd14 | | 1.015e-05 | -11.50 | HOSHIDA\_LIVER\_CANCER\_SUBCLASS\_S1 | MSigDB lists | HOSHIDA\_LIVER\_CANCER\_SUBCLASS\_S1 | 205 | 13 | 12187 | 179 | Ier3,Col4a1,Ifi207,Ifi204,Ifi209,Msn,Plaur,Cybb,Ccnd2,Iqgap1,Ifi211,C3ar1,Gem | | 1.040e-05 | -11.47 | host | cellular component | GO:0018995 | 20 | 5 | 13825 | 212 | Iigp1,Gbp3,Gbp7,Irgm2,Gbp6 | | 1.040e-05 | -11.47 | host cell | cellular component | GO:0043657 | 20 | 5 | 13825 | 212 | Irgm2,Gbp6,Gbp3,Iigp1,Gbp7 | | 1.058e-05 | -11.46 | GSE3982\_EOSINOPHIL\_VS\_NEUTROPHIL\_DN | MSigDB lists | GSE3982\_EOSINOPHIL\_VS\_NEUTROPHIL\_DN | 147 | 11 | 12187 | 179 | Ptges,Ifitm3,Tnfsf10,Irf2,Plek,Icam1,Apold1,Trib1,Atf3,Tnfaip3,Il1rn | | 1.061e-05 | -11.45 | Guanylate-bd\_C\_sf | interpro domains | IPR036543 | 10 | 4 | 13788 | 212 | Gbp3,Gbp7,Gbp6,Gbp4 | | 1.083e-05 | -11.43 | GSE4748\_CTRL\_VS\_CYANOBACTERIUM\_LPSLIKE\_STIM\_DC\_3H\_DN | MSigDB lists | GSE4748\_CTRL\_VS\_CYANOBACTERIUM\_LPSLIKE\_STIM\_DC\_3H\_DN | 176 | 12 | 12187 | 179 | Gbp3,Tnfsf10,C3ar1,AA467197,Ccl12,Tubb6,Ccl4,Ccl2,Msr1,Cxcl16,Atf3,Ier3 | | 1.090e-05 | -11.43 | negative regulation of signal transduction | biological process | GO:0009968 | 991 | 34 | 13711 | 214 | Thbs1,Socs3,Parp14,Trib1,Icam1,Nfkbia,Rgs16,Errfi1,Il6,Rasip1,Ptgs2,Ier3,Plaur,Irak3,Il1rn,Hspb1,Ripk1,Cflar,Tnip1,Rnf213,Trim30a,Tnf,Tnfaip3,Acod1,Plek,Pik3ap1,Xdh,Atf3,Sgk3,Nfe2l2,Bcl3,Hspa5,Ptpn1,Tnip3 | | 1.100e-05 | -11.42 | negative regulation of phosphate metabolic process | biological process | GO:0045936 | 498 | 22 | 13711 | 214 | Iqgap1,Ptpn1,Birc3,Gbp4,Ier3,Rasip1,Cdkn1a,Il6,Errfi1,Atf3,Xdh,Plek,Tnfaip3,Tnf,Gadd45g,Tnip1,Gadd45b,Parp14,Trib1,Hspb1,Socs3,Irak3 | | 1.100e-05 | -11.42 | negative regulation of phosphorus metabolic process | biological process | GO:0010563 | 498 | 22 | 13711 | 214 | Errfi1,Il6,Atf3,Xdh,Cdkn1a,Tnfaip3,Tnf,Plek,Tnip1,Gadd45g,Gadd45b,Hspb1,Socs3,Irak3,Parp14,Trib1,Iqgap1,Gbp4,Birc3,Ptpn1,Ier3,Rasip1 | | 1.126e-05 | -11.39 | regulation of interferon-gamma-mediated signaling pathway | biological process | GO:0060334 | 10 | 4 | 13711 | 214 | Parp9,Irgm2,Parp14,Irgm1 | | 1.126e-05 | -11.39 | regulation of response to interferon-gamma | biological process | GO:0060330 | 10 | 4 | 13711 | 214 | Irgm1,Parp14,Irgm2,Parp9 | | 1.129e-05 | -11.39 | GSE43955\_TH0\_VS\_TGFB\_IL6\_TH17\_ACT\_CD4\_TCELL\_52H\_DN | MSigDB lists | GSE43955\_TH0\_VS\_TGFB\_IL6\_TH17\_ACT\_CD4\_TCELL\_52H\_DN | 148 | 11 | 12187 | 179 | Il17ra,Errfi1,Saa1,Il1rn,Tiparp,Bach1,Gadd45g,Cebpd,Map3k8,Socs3,Fstl1 | | 1.129e-05 | -11.39 | GSE3982\_BCELL\_VS\_CENT\_MEMORY\_CD4\_TCELL\_UP | MSigDB lists | GSE3982\_BCELL\_VS\_CENT\_MEMORY\_CD4\_TCELL\_UP | 148 | 11 | 12187 | 179 | Cdkn1a,Cd14,Csf2rb,Plek,Tor3a,Trib1,Map3k8,Tubb6,Cybb,Irf7,S100a8 | | 1.132e-05 | -11.39 | positive regulation of vascular endothelial growth factor production | biological process | GO:0010575 | 20 | 5 | 13711 | 214 | Il1a,C3ar1,C5ar1,Il6,Ptgs2 | | 1.143e-05 | -11.38 | MODULE\_291 | MSigDB lists | MODULE\_291 | 53 | 7 | 12187 | 179 | Spi1,Ier3,Cd14,S100a9,C3ar1,Saa1,C5ar1 | | 1.143e-05 | -11.38 | GO\_POSITIVE\_REGULATION\_OF\_LEUKOCYTE\_CHEMOTAXIS | MSigDB lists | GO\_POSITIVE\_REGULATION\_OF\_LEUKOCYTE\_CHEMOTAXIS | 53 | 7 | 12187 | 179 | C3ar1,C5ar1,Cxcl1,Thbs1,Ccl4,Il6,Ccl12 | | 1.147e-05 | -11.38 | GO\_REGULATION\_OF\_VASCULATURE\_DEVELOPMENT | MSigDB lists | GO\_REGULATION\_OF\_VASCULATURE\_DEVELOPMENT | 177 | 12 | 12187 | 179 | Thbs1,Runx1,Il6,Ccl12,Tnfaip3,Il1a,Angptl4,C3ar1,Ptgs2,C5ar1,Xdh,Hspb1 | | 1.203e-05 | -11.33 | GSE17721\_CTRL\_VS\_PAM3CSK4\_2H\_BMDC\_DN | MSigDB lists | GSE17721\_CTRL\_VS\_PAM3CSK4\_2H\_BMDC\_DN | 149 | 11 | 12187 | 179 | Arid5b,Cflar,Ptgs2,Map3k6,Slfn4,Slfn3,Zbtb16,Parp14,Pik3r1,Il1rn,Cp | | 1.211e-05 | -11.32 | GO\_TRANSCRIPTIONAL\_REPRESSOR\_ACTIVITY\_RNA\_POLYMERASE\_II\_TRANSCRIPTION\_REGULATORY\_REGION\_SEQUENCE\_SPECIFIC\_BINDING | MSigDB lists | GO\_TRANSCRIPTIONAL\_REPRESSOR\_ACTIVITY\_RNA\_POLYMERASE\_II\_TRANSCRIPTION\_REGULATORY\_REGION\_SEQUENCE\_SPECIFIC\_BINDING | 122 | 10 | 12187 | 179 | Ifi211,Arid5b,Atf3,Nfkb1,Ifi204,Bach1,Ifi207,Zbtb16,Spi1,Ifi209 | | 1.214e-05 | -11.32 | DAZARD\_RESPONSE\_TO\_UV\_NHEK\_UP | MSigDB lists | DAZARD\_RESPONSE\_TO\_UV\_NHEK\_UP | 178 | 12 | 12187 | 179 | S100a9,Cxcl1,Gem,Ptgs2,Zfp36,Trib1,Sgk1,Atf3,Il6,Gadd45b,Ier3,Tnfaip3 | | 1.214e-05 | -11.32 | LEE\_RECENT\_THYMIC\_EMIGRANT | MSigDB lists | LEE\_RECENT\_THYMIC\_EMIGRANT | 178 | 12 | 12187 | 179 | Tnfsf10,Cmpk2,Ifi211,Tnfaip3,Rhou,Tagln2,Isg15,Ifi207,Ifi204,Nfkbiz,Socs3,Ifi209 | | 1.232e-05 | -11.30 | ONKEN\_UVEAL\_MELANOMA\_UP | MSigDB lists | ONKEN\_UVEAL\_MELANOMA\_UP | 661 | 25 | 12187 | 179 | Ccnd2,Iqgap1,Tnfaip8,Phf11d,Ifi211,Ier3,Cebpd,Phf11b,Sgk1,Ifitm3,Isg15,Parp12,Trib1,Gem,Luc7l3,Ifi204,Col4a1,Ifi207,Cdkn1a,Ifi209,Clic4,Msn,Irf9,Tagln2,Ifitm2 | | 1.247e-05 | -11.29 | SAGIV\_CD24\_TARGETS\_DN | MSigDB lists | SAGIV\_CD24\_TARGETS\_DN | 36 | 6 | 12187 | 179 | Thbs1,Cdkn1a,Atf3,Oasl1,Gem,Icam1 | | 1.247e-05 | -11.29 | ZHU\_CMV\_8\_HR\_UP | MSigDB lists | ZHU\_CMV\_8\_HR\_UP | 36 | 6 | 12187 | 179 | Ptgs2,Isg15,Irf2,Il6,Ripk1,Tnfaip3 | | 1.278e-05 | -11.27 | positive regulation of programmed cell death | biological process | GO:0043068 | 541 | 23 | 13711 | 214 | Gadd45b,Hcar2,Gadd45g,Thbs1,Cdkn1a,Ip6k2,Il6,Akap12,Casp4,Plaur,Ptgs2,Tnfaip8,Ripk1,Zbtb16,Xdh,Tnfsf10,Atf3,Tnf,Ifit2,S100a9,Ccl12,S100a8,Tgm2 | | 1.282e-05 | -11.26 | GSE36392\_TYPE\_2\_MYELOID\_VS\_EOSINOPHIL\_IL25\_TREATED\_LUNG\_DN | MSigDB lists | GSE36392\_TYPE\_2\_MYELOID\_VS\_EOSINOPHIL\_IL25\_TREATED\_LUNG\_DN | 150 | 11 | 12187 | 179 | Irf9,Isg15,Herc6,Lcn2,Stx11,Ifit3b,S100a8,AA467197,Gadd45b,Hcar2,Tnf | | 1.297e-05 | -11.25 | YORDY\_RECIPROCAL\_REGULATION\_BY\_ETS1\_AND\_SP100\_DN | MSigDB lists | YORDY\_RECIPROCAL\_REGULATION\_BY\_ETS1\_AND\_SP100\_DN | 54 | 7 | 12187 | 179 | Irf9,Icam1,Ptges,Angptl4,Samd9l,Dnajb1,Socs3 | | 1.301e-05 | -11.25 | SMIRNOV\_CIRCULATING\_ENDOTHELIOCYTES\_IN\_CANCER\_UP | MSigDB lists | SMIRNOV\_CIRCULATING\_ENDOTHELIOCYTES\_IN\_CANCER\_UP | 123 | 10 | 12187 | 179 | Rgs16,Irak3,Ptges,Cd14,C5ar1,C3ar1,Ier3,Cdkn1a,Thbs1,Maff | | 1.304e-05 | -11.25 | GO\_REGULATION\_OF\_PROTEIN\_MODIFICATION\_PROCESS | MSigDB lists | GO\_REGULATION\_OF\_PROTEIN\_MODIFICATION\_PROCESS | 1315 | 39 | 12187 | 179 | Fzd4,C5ar1,Xdh,Trib1,Sdc4,Tnf,Birc3,Tnip1,Saa1,Errfi1,Plaur,Icam1,Hspb1,Plek,Socs3,Cdkn1a,Atf3,Ksr1,Pik3r5,Csf3,Iqgap1,Ccl4,Ccl2,Spi1,Map3k8,Ccnd2,Tnfaip3,Ccl12,Ripk1,Gadd45b,Il6,Isg15,Map3k6,Ptpn1,Irak3,Hspa5,Thbs1,Il1a,Gadd45g | | 1.313e-05 | -11.24 | GO\_APOPTOTIC\_SIGNALING\_PATHWAY | MSigDB lists | GO\_APOPTOTIC\_SIGNALING\_PATHWAY | 242 | 14 | 12187 | 179 | Cd14,Ifi204,Il1a,Ifi207,Ifi209,Cdkn1a,Tnfsf10,Ifi211,Bcl3,Rrp8,Ripk1,Tnf,Pik3r1,Casp4 | | 1.343e-05 | -11.22 | regulation of interleukin-6 production | biological process | GO:0032675 | 116 | 10 | 13711 | 214 | Il6,Prg4,Tnfaip3,Tnf,Il1a,Lgals9,Trim30a,Il1rn,Irak3,Mmp8 | | 1.351e-05 | -11.21 | GO\_REGULATION\_OF\_DNA\_BINDING | MSigDB lists | GO\_REGULATION\_OF\_DNA\_BINDING | 75 | 8 | 12187 | 179 | Ifi209,Sox11,Nfkbia,Ifi207,Ifi204,Bcl3,Ifi211,Plaur | | 1.351e-05 | -11.21 | GO\_MYELOID\_LEUKOCYTE\_DIFFERENTIATION | MSigDB lists | GO\_MYELOID\_LEUKOCYTE\_DIFFERENTIATION | 75 | 8 | 12187 | 179 | Ifi209,Tnf,Spi1,Ifi207,Ifi204,Csf3,Ifi211,Sbno2 | | 1.365e-05 | -11.20 | GSE46242\_CTRL\_VS\_EGR2\_DELETED\_ANERGIC\_TH1\_CD4\_TCELL\_DN | MSigDB lists | GSE46242\_CTRL\_VS\_EGR2\_DELETED\_ANERGIC\_TH1\_CD4\_TCELL\_DN | 151 | 11 | 12187 | 179 | Ccl4,Tor3a,Birc3,Rassf4,Cybb,Bcl3,Nfkbie,Nfkb2,Gbp3,Cdkn1a,Nfkb1 | | 1.370e-05 | -11.20 | GRANDVAUX\_IFN\_RESPONSE\_NOT\_VIA\_IRF3 | MSigDB lists | GRANDVAUX\_IFN\_RESPONSE\_NOT\_VIA\_IRF3 | 11 | 4 | 12187 | 179 | Irf7,Ifitm3,Irgm2,Irf9 | | 1.370e-05 | -11.20 | GO\_REGULATION\_OF\_TUMOR\_NECROSIS\_FACTOR\_BIOSYNTHETIC\_PROCESS | MSigDB lists | GO\_REGULATION\_OF\_TUMOR\_NECROSIS\_FACTOR\_BIOSYNTHETIC\_PROCESS | 11 | 4 | 12187 | 179 | Errfi1,Hspb1,Bcl3,Thbs1 | | 1.370e-05 | -11.20 | GO\_REGULATION\_OF\_MACROPHAGE\_CHEMOTAXIS | MSigDB lists | GO\_REGULATION\_OF\_MACROPHAGE\_CHEMOTAXIS | 11 | 4 | 12187 | 179 | Ccl12,C5ar1,Thbs1,C3ar1 | | 1.382e-05 | -11.19 | positive regulation of stress-activated MAPK cascade | biological process | GO:0032874 | 142 | 11 | 13711 | 214 | Gadd45g,Gadd45b,Mmp8,Ripk1,Ncf1,Il1rn,Xdh,Il1a,Ptpn1,Fzd4,Tnf | | 1.398e-05 | -11.18 | GSE1112\_HY\_CD8AB\_VS\_HY\_CD8AA\_THYMOCYTE\_RTOC\_CULTURE\_UP | MSigDB lists | GSE1112\_HY\_CD8AB\_VS\_HY\_CD8AA\_THYMOCYTE\_RTOC\_CULTURE\_UP | 124 | 10 | 12187 | 179 | Tiparp,Samd9l,Tnf,Ccnd2,Vcam1,Parp14,Rtp4,Gbp3,Ptgs2,Banp | | 1.419e-05 | -11.16 | Regulation of necroptotic cell death | REACTOME pathways | R-MMU-5675482 | 10 | 4 | 6297 | 105 | Cflar,Ripk1,Tnfsf10,Birc3 | | 1.419e-05 | -11.16 | RIPK1-mediated regulated necrosis | REACTOME pathways | R-MMU-5213460 | 10 | 4 | 6297 | 105 | Ripk1,Cflar,Tnfsf10,Birc3 | | 1.419e-05 | -11.16 | Regulated Necrosis | REACTOME pathways | R-MMU-5218859 | 10 | 4 | 6297 | 105 | Ripk1,Cflar,Tnfsf10,Birc3 | | 1.428e-05 | -11.16 | GO\_POSITIVE\_REGULATION\_OF\_LOCOMOTION | MSigDB lists | GO\_POSITIVE\_REGULATION\_OF\_LOCOMOTION | 312 | 16 | 12187 | 179 | Ccl12,Il6,Tnf,Pik3r1,Ccl4,C5ar1,C3ar1,Cxcl1,Thbs1,Hspa5,Hspb1,Icam1,P2ry6,Cxcl16,Selp,Ptgs2 | | 1.442e-05 | -11.15 | cell migration | biological process | GO:0016477 | 664 | 26 | 13711 | 214 | Rhoc,Retnlg,Sele,Il1rn,Arid5b,Ccl2,Saa1,Cxcl9,Tnf,Tnfaip3,Selp,Msn,S100a9,Vcam1,Ccl12,Cxcl16,S100a8,Iqgap1,C5ar1,Cxcl1,Ccl7,Thbs1,Icam1,Il17ra,Ccl4,Sdc4 | | 1.449e-05 | -11.14 | positive regulation of JNK cascade | biological process | GO:0046330 | 117 | 10 | 13711 | 214 | Ripk1,Mmp8,Il1rn,Ncf1,Gadd45b,Gadd45g,Fzd4,Il1a,Ptpn1,Tnf | | 1.453e-05 | -11.14 | GSE43955\_1H\_VS\_20H\_ACT\_CD4\_TCELL\_WITH\_TGFB\_IL6\_DN | MSigDB lists | GSE43955\_1H\_VS\_20H\_ACT\_CD4\_TCELL\_WITH\_TGFB\_IL6\_DN | 152 | 11 | 12187 | 179 | Casp4,Birc3,Osmr,Irf7,C3ar1,Csf3,Il1a,Nfkbiz,Csf2rb,Clic4,Ptgs2 | | 1.453e-05 | -11.14 | GSE21379\_WT\_VS\_SAP\_KO\_TFH\_CD4\_TCELL\_DN | MSigDB lists | GSE21379\_WT\_VS\_SAP\_KO\_TFH\_CD4\_TCELL\_DN | 152 | 11 | 12187 | 179 | Bach1,Ms4a6d,Gbp4,Oasl1,Ifi44,Zbtb16,Ccl4,Icam1,Rtp4,Gbp7,Gbp6 | | 1.453e-05 | -11.14 | GSE22589\_HIV\_VS\_HIV\_AND\_SIV\_INFECTED\_DC\_UP | MSigDB lists | GSE22589\_HIV\_VS\_HIV\_AND\_SIV\_INFECTED\_DC\_UP | 152 | 11 | 12187 | 179 | Slfn4,Slfn3,Errfi1,Gem,Bcl3,Il1a,Tnfaip3,Il6,Tnf,Nfkbia,Gpr84 | | 1.453e-05 | -11.14 | GSE41176\_WT\_VS\_TAK1\_KO\_ANTI\_IGM\_STIM\_BCELL\_6H\_UP | MSigDB lists | GSE41176\_WT\_VS\_TAK1\_KO\_ANTI\_IGM\_STIM\_BCELL\_6H\_UP | 152 | 11 | 12187 | 179 | Zfp36,Sbno2,Ptgs2,Cflar,Cxcl1,Gadd45g,Ier3,Adamts1,Map3k8,Ccl4,Socs3 | | 1.470e-05 | -11.13 | GO\_REGULATION\_OF\_TOLL\_LIKE\_RECEPTOR\_SIGNALING\_PATHWAY | MSigDB lists | GO\_REGULATION\_OF\_TOLL\_LIKE\_RECEPTOR\_SIGNALING\_PATHWAY | 37 | 6 | 12187 | 179 | Irf7,Cd14,Birc3,Tnfaip3,Irak3,Pik3ap1 | | 1.470e-05 | -11.13 | FRIDMAN\_IMMORTALIZATION\_DN | MSigDB lists | FRIDMAN\_IMMORTALIZATION\_DN | 37 | 6 | 12187 | 179 | Ifi209,Cdkn1a,Ifi211,Irf7,Ifi204,Ifi207 | | 1.477e-05 | -11.12 | positive regulation of stress-activated protein kinase signaling cascade | biological process | GO:0070304 | 143 | 11 | 13711 | 214 | Xdh,Tnf,Ptpn1,Il1a,Fzd4,Gadd45g,Gadd45b,Ncf1,Il1rn,Mmp8,Ripk1 | | 1.483e-05 | -11.12 | CCR2 chemokine receptor binding | molecular function | GO:0031727 | 4 | 3 | 13516 | 211 | Ccl12,Ccl2,Ccl7 | | 1.491e-05 | -11.11 | GO\_RESPONSE\_TO\_INTERLEUKIN\_1 | MSigDB lists | GO\_RESPONSE\_TO\_INTERLEUKIN\_1 | 76 | 8 | 12187 | 179 | Icam1,Irak3,Sele,Nfkb1,Ccl12,Il6,Ccl4,Ccl2 | | 1.500e-05 | -11.11 | GSE14386\_UNTREATED\_VS\_IFNA\_TREATED\_ACT\_PBMC\_MS\_PATIENT\_DN | MSigDB lists | GSE14386\_UNTREATED\_VS\_IFNA\_TREATED\_ACT\_PBMC\_MS\_PATIENT\_DN | 125 | 10 | 12187 | 179 | Parp12,Ccl12,Stx11,Ifit3b,Maff,Birc3,Ifi44,Rgs16,Rtp4,Irf7 | | 1.500e-05 | -11.11 | GSE1740\_UNSTIM\_VS\_IFNA\_STIMULATED\_MCSF\_DERIVED\_MACROPHAGE\_DN | MSigDB lists | GSE1740\_UNSTIM\_VS\_IFNA\_STIMULATED\_MCSF\_DERIVED\_MACROPHAGE\_DN | 125 | 10 | 12187 | 179 | Tnf,Parp14,Samd9l,Ptgs2,Cxcl1,Irf7,Rtp4,Gbp3,Plaur,Tap1 | | 1.546e-05 | -11.08 | HOXA9\_DN.V1\_UP | MSigDB lists | HOXA9\_DN.V1\_UP | 153 | 11 | 12187 | 179 | Ifi204,Ms4a6d,Ifi207,Ifi209,Rbm47,Sgk3,Isg15,Parp12,S100a8,Ifi211,S100a9 | | 1.546e-05 | -11.08 | GSE28737\_FOLLICULAR\_VS\_MARGINAL\_ZONE\_BCELL\_DN | MSigDB lists | GSE28737\_FOLLICULAR\_VS\_MARGINAL\_ZONE\_BCELL\_DN | 153 | 11 | 12187 | 179 | Parp9,Irf2,Cmpk2,Rtp4,Icam1,Slfn9,Irgm2,Znfx1,Samd9l,Cdkn1a,Ccnd2 | | 1.546e-05 | -11.08 | GSE29618\_MONOCYTE\_VS\_PDC\_UP | MSigDB lists | GSE29618\_MONOCYTE\_VS\_PDC\_UP | 153 | 11 | 12187 | 179 | C5ar1,S100a9,Spi1,Trib1,Ifitm2,Cd14,Irak3,Msn,Il1rn,Timp1,Ier3 | | 1.548e-05 | -11.08 | MODULE\_3 | MSigDB lists | MODULE\_3 | 350 | 17 | 12187 | 179 | Plaur,Fstl1,Cdkn1a,Ifi209,Sgk1,Thbs1,Ier3,Ifi204,Col4a1,Ifi207,Akap12,Ifi211,Iqgap1,Tgm2,Casp4,Maff,Ccl12 | | 1.602e-05 | -11.04 | PEDRIOLI\_MIR31\_TARGETS\_DN | MSigDB lists | PEDRIOLI\_MIR31\_TARGETS\_DN | 280 | 15 | 12187 | 179 | Vcam1,Ifit3b,Nfkbiz,Samd9l,Akap12,Rasd1,Icam1,Sele,Ifit2,Rhou,Birc3,Tnfaip3,Cxcl1,Adamts9,Nfkb2 | | 1.609e-05 | -11.04 | GO\_POSITIVE\_REGULATION\_OF\_PEPTIDASE\_ACTIVITY | MSigDB lists | GO\_POSITIVE\_REGULATION\_OF\_PEPTIDASE\_ACTIVITY | 126 | 10 | 12187 | 179 | Ripk1,Ifi204,Ifi207,Tnf,Ifi209,Xdh,Tnfsf10,S100a8,Ifi211,S100a9 | | 1.625e-05 | -11.03 | regulation of cysteine-type endopeptidase activity | biological process | GO:2000116 | 201 | 13 | 13711 | 214 | S100a8,Birc3,Ptgs2,Plaur,S100a9,Tnfsf10,Il6,Xdh,Casp4,Tnf,Thbs1,Cflar,Tnfaip8 | | 1.634e-05 | -11.02 | regulation of MAP kinase activity | biological process | GO:0043405 | 263 | 15 | 13711 | 214 | Pik3r5,Iqgap1,Ptpn1,Map3k6,Fzd4,Map3k8,Gadd45g,Gadd45b,Ripk1,Trib1,Irak3,Thbs1,Il1rn,Ksr1,Tnf | | 1.644e-05 | -11.02 | GSE8835\_HEALTHY\_VS\_CLL\_CD8\_TCELL\_DN | MSigDB lists | GSE8835\_HEALTHY\_VS\_CLL\_CD8\_TCELL\_DN | 154 | 11 | 12187 | 179 | Oasl1,Hp,Col4a1,Nfkbiz,Il4ra,Gbp3,Errfi1,S100a8,Ptges,Tnip1,Csf3 | | 1.644e-05 | -11.02 | GSE43955\_TH0\_VS\_TGFB\_IL6\_TH17\_ACT\_CD4\_TCELL\_10H\_DN | MSigDB lists | GSE43955\_TH0\_VS\_TGFB\_IL6\_TH17\_ACT\_CD4\_TCELL\_10H\_DN | 154 | 11 | 12187 | 179 | Il4ra,Ccl4,Il1rn,Gadd45g,Ripk1,Cebpd,Il6,Selp,Saa1,Il17ra,Gbp3 | | 1.647e-05 | -11.01 | G\_GB1\_RHD3\_dom | interpro domains | IPR030386 | 11 | 4 | 13788 | 212 | Gbp4,Gbp6,Gbp7,Gbp3 | | 1.647e-05 | -11.01 | Guanylate-bd\_N | interpro domains | IPR015894 | 11 | 4 | 13788 | 212 | Gbp6,Gbp7,Gbp3,Gbp4 | | 1.647e-05 | -11.01 | Chemokine\_CC\_CS | interpro domains | IPR000827 | 11 | 4 | 13788 | 212 | Ccl7,Ccl2,Ccl12,Ccl4 | | 1.656e-05 | -11.01 | GO\_POSITIVE\_REGULATION\_OF\_HYDROLASE\_ACTIVITY | MSigDB lists | GO\_POSITIVE\_REGULATION\_OF\_HYDROLASE\_ACTIVITY | 716 | 26 | 12187 | 179 | Iqgap1,S100a8,Rgs16,Ifi211,Rhoc,Ripk1,Ccl12,Ccl4,Ccl2,Ptpn1,Hspa5,Dnajb1,Xdh,Tnfsf10,C5ar1,A2m,S100a9,Tnf,Icam1,Sele,Plek,Errfi1,Csf2rb,Ifi204,Ifi207,Ifi209 | | 1.658e-05 | -11.01 | GO\_CELLULAR\_RESPONSE\_TO\_INTERLEUKIN\_1 | MSigDB lists | GO\_CELLULAR\_RESPONSE\_TO\_INTERLEUKIN\_1 | 56 | 7 | 12187 | 179 | Il6,Ccl12,Nfkb1,Ccl2,Ccl4,Icam1,Irak3 | | 1.709e-05 | -10.98 | positive regulation of protein serine/threonine kinase activity | biological process | GO:0071902 | 264 | 15 | 13711 | 214 | Iqgap1,Ptpn1,Map3k6,Fzd4,Map3k8,Irgm2,Pik3r5,Tnf,Lgals9,Gadd45g,Ccnd2,Gadd45b,Ripk1,Thbs1,Il1rn | | 1.710e-05 | -10.98 | SMID\_BREAST\_CANCER\_BASAL\_UP | MSigDB lists | SMID\_BREAST\_CANCER\_BASAL\_UP | 467 | 20 | 12187 | 179 | Fam107a,Ccl12,S100a9,Angptl4,Ifi211,S100a8,Rtp4,Sox11,Ifi209,Socs3,Cp,Ifi207,Ifi204,Lcn2,Ptgs2,Tagln2,Icam1,Msn,Tap1,Clic4 | | 1.722e-05 | -10.97 | other organism part | cellular component | GO:0044217 | 22 | 5 | 13825 | 212 | Gbp3,Iigp1,Gbp7,Irgm2,Gbp6 | | 1.722e-05 | -10.97 | other organism cell | cellular component | GO:0044216 | 22 | 5 | 13825 | 212 | Gbp7,Gbp3,Iigp1,Gbp6,Irgm2 | | 1.725e-05 | -10.97 | KEGG\_CYTOSOLIC\_DNA\_SENSING\_PATHWAY | MSigDB lists | KEGG\_CYTOSOLIC\_DNA\_SENSING\_PATHWAY | 38 | 6 | 12187 | 179 | Nfkb1,Ripk1,Il6,Ccl4,Nfkbia,Irf7 | | 1.730e-05 | -10.96 | GO\_RESPONSE\_TO\_METAL\_ION | MSigDB lists | GO\_RESPONSE\_TO\_METAL\_ION | 248 | 14 | 12187 | 179 | Hspa5,Thbs1,Vcam1,Nfkb1,Cp,Il1a,Ptgs2,Cd14,Icam1,Clic4,Il6,Ptges,Iqgap1,S100a8 | | 1.743e-05 | -10.96 | GBP | pfam domains | PF02263 | 11 | 4 | 12881 | 201 | Gbp6,Gbp7,Gbp4,Gbp3 | | 1.747e-05 | -10.95 | retina vasculature morphogenesis in camera-type eye | biological process | GO:0061299 | 11 | 4 | 13711 | 214 | Fzd4,Clic4,Col4a1,Rhoj | | 1.748e-05 | -10.95 | GSE360\_LOW\_DOSE\_B\_MALAYI\_VS\_M\_TUBERCULOSIS\_MAC\_DN | MSigDB lists | GSE360\_LOW\_DOSE\_B\_MALAYI\_VS\_M\_TUBERCULOSIS\_MAC\_DN | 155 | 11 | 12187 | 179 | Irak3,Irf2,Il1rn,Osmr,Olfml2b,Pygm,Msr1,Zfp189,Runx1,Sntb2,Ripk1 | | 1.748e-05 | -10.95 | GSE43863\_TFH\_VS\_LY6C\_INT\_CXCR5POS\_EFFECTOR\_CD4\_TCELL\_DN | MSigDB lists | GSE43863\_TFH\_VS\_LY6C\_INT\_CXCR5POS\_EFFECTOR\_CD4\_TCELL\_DN | 155 | 11 | 12187 | 179 | Il6,Gadd45b,Irgm2,Bcl3,Ptges,Il1rn,Stx11,Ksr1,Nfkbiz,Socs3,Ifitm3 | | 1.748e-05 | -10.95 | GSE25088\_IL4\_VS\_IL4\_AND\_ROSIGLITAZONE\_STIM\_STAT6\_KO\_MACROPHAGE\_DAY10\_DN | MSigDB lists | GSE25088\_IL4\_VS\_IL4\_AND\_ROSIGLITAZONE\_STIM\_STAT6\_KO\_MACROPHAGE\_DAY10\_DN | 155 | 11 | 12187 | 179 | Dtx3l,Pik3r5,Nfkb2,Pik3ap1,Cxcl16,Hspa5,Ifit3b,Nfkb1,Il1a,Lcn2,Ier3 | | 1.748e-05 | -10.95 | positive regulation of cell death | biological process | GO:0010942 | 591 | 24 | 13711 | 214 | Tgm2,Ccl12,Hp,S100a8,S100a9,Plaur,Ifit2,Ptgs2,Akap12,Casp4,Tnf,Cdkn1a,Ip6k2,Tnfsf10,Il6,Atf3,Xdh,Zbtb16,Ripk1,Tnfaip8,Thbs1,Gadd45g,Hcar2,Gadd45b | | 1.755e-05 | -10.95 | GO\_ACUTE\_PHASE\_RESPONSE | MSigDB lists | GO\_ACUTE\_PHASE\_RESPONSE | 23 | 5 | 12187 | 179 | Saa1,Hp,Il1a,Il1rn,Il6 | | 1.781e-05 | -10.94 | Metal sequestration by antimicrobial proteins | REACTOME pathways | R-MMU-6799990 | 4 | 3 | 6297 | 105 | Lcn2,S100a9,S100a8 | | 1.845e-05 | -10.90 | negative regulation of phosphorylation | biological process | GO:0042326 | 403 | 19 | 13711 | 214 | Birc3,Ptpn1,Gbp4,Ier3,Rasip1,Cdkn1a,Errfi1,Il6,Atf3,Xdh,Tnfaip3,Tnip1,Gadd45g,Gadd45b,Parp14,Trib1,Hspb1,Socs3,Irak3 | | 1.856e-05 | -10.89 | GSE12366\_GC\_VS\_NAIVE\_BCELL\_DN | MSigDB lists | GSE12366\_GC\_VS\_NAIVE\_BCELL\_DN | 156 | 11 | 12187 | 179 | Ifit2,Ifi209,Ifi207,Ifi204,Dtx3l,Herc6,Ifitm3,Ifi211,Irf9,Msn,Parp9 | | 1.856e-05 | -10.89 | GSE42021\_TCONV\_PLN\_VS\_CD24HI\_TCONV\_THYMUS\_UP | MSigDB lists | GSE42021\_TCONV\_PLN\_VS\_CD24HI\_TCONV\_THYMUS\_UP | 156 | 11 | 12187 | 179 | Maff,Tgm2,Bach1,Atf3,Arid5b,Cxcl1,Rtp4,Zfp36,Icam1,Tap1,Sbno2 | | 1.856e-05 | -10.89 | GSE17721\_CTRL\_VS\_POLYIC\_2H\_BMDC\_DN | MSigDB lists | GSE17721\_CTRL\_VS\_POLYIC\_2H\_BMDC\_DN | 156 | 11 | 12187 | 179 | Ripk1,Hcar2,Gpr84,Pygm,Arid5b,Cflar,C5ar1,Luc7l3,Cdkn1a,Icam1,Cd14 | | 1.865e-05 | -10.89 | PLASARI\_TGFB1\_TARGETS\_10HR\_DN | MSigDB lists | PLASARI\_TGFB1\_TARGETS\_10HR\_DN | 217 | 13 | 12187 | 179 | Usp18,Pik3r1,Ifit2,Rtp4,Arid5b,C3ar1,Il1rn,Ifit3b,Vcam1,Adamts1,Parp9,Plek,Herc6 | | 1.893e-05 | -10.87 | GO\_REGULATION\_OF\_APOPTOTIC\_SIGNALING\_PATHWAY | MSigDB lists | GO\_REGULATION\_OF\_APOPTOTIC\_SIGNALING\_PATHWAY | 284 | 15 | 12187 | 179 | Plaur,Ptpn1,Hspb1,Icam1,Thbs1,Atf3,Il1a,S100a9,Cflar,Tnfsf10,S100a8,Tnf,Nfe2l2,Tnfaip3,Ripk1 | | 1.971e-05 | -10.83 | GSE17721\_CTRL\_VS\_CPG\_4H\_BMDC\_DN | MSigDB lists | GSE17721\_CTRL\_VS\_CPG\_4H\_BMDC\_DN | 157 | 11 | 12187 | 179 | Aff1,Tgm2,Ms4a6d,Nfkb1,Il1rn,Atf3,Znfx1,Gbp7,Ptges,Pik3ap1,Gbp3 | | 1.971e-05 | -10.83 | GSE46606\_DAY1\_VS\_DAY3\_CD40L\_IL2\_IL5\_STIMULATED\_BCELL\_DN | MSigDB lists | GSE46606\_DAY1\_VS\_DAY3\_CD40L\_IL2\_IL5\_STIMULATED\_BCELL\_DN | 157 | 11 | 12187 | 179 | Rnf213,Ifi207,Il1a,Ifi204,Sgk1,Ifi209,Tnfaip8,Dtx3l,Ifi211,Birc3,Runx1 | | 1.987e-05 | -10.83 | MODULE\_174 | MSigDB lists | MODULE\_174 | 79 | 8 | 12187 | 179 | Cp,Ier3,Spi1,C3ar1,Saa1,C5ar1,S100a9,Cd14 | | 2.014e-05 | -10.81 | WUNDER\_INFLAMMATORY\_RESPONSE\_AND\_CHOLESTEROL\_UP | MSigDB lists | WUNDER\_INFLAMMATORY\_RESPONSE\_AND\_CHOLESTEROL\_UP | 39 | 6 | 12187 | 179 | Cmpk2,Gbp4,Gbp6,Ifi44,Cd14,Nfkbiz | | 2.014e-05 | -10.81 | TAVOR\_CEBPA\_TARGETS\_UP | MSigDB lists | TAVOR\_CEBPA\_TARGETS\_UP | 39 | 6 | 12187 | 179 | Akap12,Il1rn,S100a9,Trib1,C3ar1,Ccnd2 | | 2.032e-05 | -10.80 | ROETH\_TERT\_TARGETS\_UP | MSigDB lists | ROETH\_TERT\_TARGETS\_UP | 12 | 4 | 12187 | 179 | Ifi44,Herc6,Ifit3b,Oasl1 | | 2.092e-05 | -10.77 | GSE6092\_B\_BURGDOFERI\_VS\_B\_BURGDORFERI\_AND\_IFNG\_STIM\_ENDOTHELIAL\_CELL\_DN | MSigDB lists | GSE6092\_B\_BURGDOFERI\_VS\_B\_BURGDORFERI\_AND\_IFNG\_STIM\_ENDOTHELIAL\_CELL\_DN | 158 | 11 | 12187 | 179 | Gbp3,Pik3r5,Cd14,Nfkbie,Tnfaip3,Gadd45b,Lcn2,Stx11,Tnip3,Cp,Trib1 | | 2.092e-05 | -10.77 | GSE41867\_DAY15\_EFFECTOR\_VS\_DAY30\_EXHAUSTED\_CD8\_TCELL\_LCMV\_CLONE13\_UP | MSigDB lists | GSE41867\_DAY15\_EFFECTOR\_VS\_DAY30\_EXHAUSTED\_CD8\_TCELL\_LCMV\_CLONE13\_UP | 158 | 11 | 12187 | 179 | Ccl4,Parp14,Znfx1,Stx11,Irf7,Herc6,Dtx3l,Saa1,Irgm2,Rtp4,Cmpk2 | | 2.097e-05 | -10.77 | BOSCO\_TH1\_CYTOTOXIC\_MODULE | MSigDB lists | BOSCO\_TH1\_CYTOTOXIC\_MODULE | 58 | 7 | 12187 | 179 | Isg15,Herc6,Gbp6,Usp18,Cmpk2,Rtp4,Gbp4 | | 2.108e-05 | -10.77 | CHARAFE\_BREAST\_CANCER\_LUMINAL\_VS\_MESENCHYMAL\_DN | MSigDB lists | CHARAFE\_BREAST\_CANCER\_LUMINAL\_VS\_MESENCHYMAL\_DN | 396 | 18 | 12187 | 179 | Msn,Ptgs2,Ifi207,Akap12,Col4a1,Ifi204,Timp1,Socs3,Fstl1,Sgk1,Ifi209,Osmr,Cxcl1,Ifi211,Il6,Tnfaip3,Bach1,Tubb6 | | 2.123e-05 | -10.76 | Jak-STAT signaling pathway | KEGG pathways | mmu04630 | 96 | 10 | 5248 | 107 | Cdkn1a,Csf2rb,Csf3,Il6,Socs3,Il4ra,Osmr,Pik3r1,Irf9,Ccnd2 | | 2.123e-05 | -10.76 | Jak-STAT signaling pathway | KEGG pathways | ko04630 | 96 | 10 | 5248 | 107 | Cdkn1a,Csf2rb,Csf3,Il4ra,Osmr,Socs3,Il6,Irf9,Pik3r1,Ccnd2 | | 2.125e-05 | -10.76 | regulation of viral genome replication | biological process | GO:0045069 | 75 | 8 | 13711 | 214 | Ifitm3,Oasl1,Ifitm6,Resf1,Ifitm2,Oasl2,Tnf,Isg15 | | 2.180e-05 | -10.73 | GO\_NEGATIVE\_REGULATION\_OF\_EXTRINSIC\_APOPTOTIC\_SIGNALING\_PATHWAY | MSigDB lists | GO\_NEGATIVE\_REGULATION\_OF\_EXTRINSIC\_APOPTOTIC\_SIGNALING\_PATHWAY | 80 | 8 | 12187 | 179 | Cflar,Icam1,Tnfsf10,Tnf,Thbs1,Il1a,Tnfaip3,Ripk1 | | 2.209e-05 | -10.72 | Lung fibrosis | WikiPathways | WP3632 | 48 | 8 | 3756 | 96 | Nfe2l2,Timp1,Ccl2,Il6,Ccl4,Tnf,Csf3,Mt2 | | 2.219e-05 | -10.72 | GSE2826\_XID\_VS\_BTK\_KO\_BCELL\_DN | MSigDB lists | GSE2826\_XID\_VS\_BTK\_KO\_BCELL\_DN | 159 | 11 | 12187 | 179 | Lcn2,Ccl12,Hp,Il1a,Ccl2,Vcam1,Tap1,Plaur,Sele,S100a8,S100a9 | | 2.219e-05 | -10.72 | GSE17721\_CTRL\_VS\_PAM3CSK4\_4H\_BMDC\_DN | MSigDB lists | GSE17721\_CTRL\_VS\_PAM3CSK4\_4H\_BMDC\_DN | 159 | 11 | 12187 | 179 | Hp,Nfkb1,Ksr1,Sntb2,Socs3,Parp14,Ccnd2,Iqgap1,Igsf6,Ifitm2,Cflar | | 2.315e-05 | -10.67 | negative regulation of peptidase activity | biological process | GO:0010466 | 150 | 11 | 13711 | 214 | Wfdc21,Plaur,Ptgs2,Tnfaip8,Cflar,Thbs1,Serpina3f,Il6,A2m,Birc3,Timp1 | | 2.331e-05 | -10.67 | GO\_CELLULAR\_RESPONSE\_TO\_STARVATION | MSigDB lists | GO\_CELLULAR\_RESPONSE\_TO\_STARVATION | 105 | 9 | 12187 | 179 | Hspa5,Ifi209,Cdkn1a,Ifi207,Ifi204,Atf3,Nfe2l2,Rrp8,Ifi211 | | 2.331e-05 | -10.67 | GO\_POSITIVE\_REGULATION\_OF\_STRESS\_ACTIVATED\_PROTEIN\_KINASE\_SIGNALING\_CASCADE | MSigDB lists | GO\_POSITIVE\_REGULATION\_OF\_STRESS\_ACTIVATED\_PROTEIN\_KINASE\_SIGNALING\_CASCADE | 105 | 9 | 12187 | 179 | Tnf,Gadd45g,Il1a,Gadd45b,Ripk1,Map3k6,Fzd4,Ptpn1,Xdh | | 2.353e-05 | -10.66 | GSE20715\_0H\_VS\_6H\_OZONE\_LUNG\_DN | MSigDB lists | GSE20715\_0H\_VS\_6H\_OZONE\_LUNG\_DN | 160 | 11 | 12187 | 179 | Socs3,Fstl1,Hp,Cp,Cd14,Errfi1,Trib1,Rhoc,Cxcl1,Bcl3,Osmr | | 2.353e-05 | -10.66 | GSE17721\_CTRL\_VS\_LPS\_2H\_BMDC\_DN | MSigDB lists | GSE17721\_CTRL\_VS\_LPS\_2H\_BMDC\_DN | 160 | 11 | 12187 | 179 | Arid5b,Nfkb2,Birc3,Map3k8,Cdkn1a,Casp4,Nfkbia,Ccl2,Vcam1,Timp1,Atf3 | | 2.353e-05 | -10.66 | GSE29618\_MONOCYTE\_VS\_PDC\_DAY7\_FLU\_VACCINE\_UP | MSigDB lists | GSE29618\_MONOCYTE\_VS\_PDC\_DAY7\_FLU\_VACCINE\_UP | 160 | 11 | 12187 | 179 | Sgk1,Trib1,Timp1,Il1rn,Cebpd,Ier3,C5ar1,S100a9,Cd14,Igsf6,Msn | | 2.353e-05 | -10.66 | GSE23321\_CENTRAL\_MEMORY\_VS\_NAIVE\_CD8\_TCELL\_UP | MSigDB lists | GSE23321\_CENTRAL\_MEMORY\_VS\_NAIVE\_CD8\_TCELL\_UP | 160 | 11 | 12187 | 179 | Irf7,S100a9,S100a8,Ptgs2,Nfkbiz,Sgk1,Hspa5,Lcn2,Tiparp,Mmp8,Il1a | | 2.365e-05 | -10.65 | cellular response to interleukin-6 | biological process | GO:0071354 | 23 | 5 | 13711 | 214 | Spi1,Sbno2,Il6,Nfkb1,Selp | | 2.365e-05 | -10.65 | lymphocyte chemotaxis | biological process | GO:0048247 | 23 | 5 | 13711 | 214 | Cxcl16,Ccl2,Ccl12,Ccl7,Ccl4 | | 2.373e-05 | -10.65 | GO\_CELLULAR\_RESPONSE\_TO\_EXTERNAL\_STIMULUS | MSigDB lists | GO\_CELLULAR\_RESPONSE\_TO\_EXTERNAL\_STIMULUS | 222 | 13 | 12187 | 179 | Icam1,Ptgs2,Nfkb1,Ifi204,Ifi207,Atf3,Cdkn1a,Adamts1,Ifi209,Hspa5,Ifi211,Rrp8,Nfe2l2 | | 2.409e-05 | -10.63 | Cytokines and Inflammatory Response | WikiPathways | WP222 | 15 | 5 | 3756 | 96 | Il6,Cxcl1,Csf3,Tnf,Il1a | | 2.412e-05 | -10.63 | negative regulation of endopeptidase activity | biological process | GO:0010951 | 124 | 10 | 13711 | 214 | Timp1,Birc3,Il6,A2m,Thbs1,Serpina3f,Tnfaip8,Cflar,Ptgs2,Plaur | | 2.417e-05 | -10.63 | RIG-I/MDA5 mediated induction of IFN-alpha/beta pathways | REACTOME pathways | R-MMU-168928 | 36 | 6 | 6297 | 105 | Irf7,Nfkb1,Irf2,Nfkb2,Tnfaip3,Nfkbia | | 2.479e-05 | -10.61 | response to glucocorticoid | biological process | GO:0051384 | 56 | 7 | 13711 | 214 | Sgk1,Pik3r1,Il1rn,Il6,Zfp36,Tnf,Fam107a | | 2.494e-05 | -10.60 | GSE16522\_MEMORY\_VS\_NAIVE\_ANTI\_CD3CD28\_STIM\_CD8\_TCELL\_DN | MSigDB lists | GSE16522\_MEMORY\_VS\_NAIVE\_ANTI\_CD3CD28\_STIM\_CD8\_TCELL\_DN | 161 | 11 | 12187 | 179 | Casp4,Lgals9,Ccnd2,Gbp4,Irf7,Gbp6,Irgm2,Xdh,Ncf1,Rnf213,Ptpn1 | | 2.494e-05 | -10.60 | GSE21063\_WT\_VS\_NFATC1\_KO\_3H\_ANTI\_IGM\_STIM\_BCELL\_DN | MSigDB lists | GSE21063\_WT\_VS\_NFATC1\_KO\_3H\_ANTI\_IGM\_STIM\_BCELL\_DN | 161 | 11 | 12187 | 179 | Parp12,Gbp4,AA467197,Tnfsf10,Gbp3,Gbp6,Gbp7,Irgm2,Tap1,Parp9,Tnip1 | | 2.494e-05 | -10.60 | GSE7852\_LN\_VS\_FAT\_TCONV\_DN | MSigDB lists | GSE7852\_LN\_VS\_FAT\_TCONV\_DN | 161 | 11 | 12187 | 179 | Hspa5,Cdkn1a,Nfkbiz,Col4a1,Ier3,Atf3,Ripk1,Ifitm2,Tagln2,Plek,Iqgap1 | | 2.494e-05 | -10.60 | GSE23925\_DARK\_ZONE\_VS\_NAIVE\_BCELL\_DN | MSigDB lists | GSE23925\_DARK\_ZONE\_VS\_NAIVE\_BCELL\_DN | 161 | 11 | 12187 | 179 | Tnfaip3,Ccl4,Dnajb1,Trib1,Nfkbiz,Tnf,Maff,Hspa5,Ptpn1,Zfp36,Gem | | 2.530e-05 | -10.58 | Apoptosis | KEGG pathways | mmu04210 | 119 | 11 | 5248 | 107 | Gadd45g,Tnf,Birc3,Ripk1,Tnfsf10,Nfkb1,Csf2rb,Cflar,Pik3r1,Nfkbia,Gadd45b | | 2.530e-05 | -10.58 | Apoptosis | KEGG pathways | ko04210 | 119 | 11 | 5248 | 107 | Nfkbia,Gadd45b,Cflar,Pik3r1,Csf2rb,Nfkb1,Tnf,Birc3,Ripk1,Tnfsf10,Gadd45g | | 2.580e-05 | -10.57 | GSE36392\_MAC\_VS\_NEUTROPHIL\_IL25\_TREATED\_LUNG\_DN | MSigDB lists | GSE36392\_MAC\_VS\_NEUTROPHIL\_IL25\_TREATED\_LUNG\_DN | 133 | 10 | 12187 | 179 | Slfn3,Slfn4,Errfi1,Cd14,Il1a,Il1rn,Cdkn1a,Nfkbiz,Trib1,Socs3 | | 2.589e-05 | -10.56 | eosinophil chemotaxis | biological process | GO:0048245 | 12 | 4 | 13711 | 214 | Ccl7,Ccl4,Ccl12,Ccl2 | | 2.589e-05 | -10.56 | toll-like receptor 4 signaling pathway | biological process | GO:0034142 | 12 | 4 | 13711 | 214 | Tnip3,Nfkbia,Cd14,Pik3ap1 | | 2.589e-05 | -10.56 | eosinophil migration | biological process | GO:0072677 | 12 | 4 | 13711 | 214 | Ccl4,Ccl7,Ccl12,Ccl2 | | 2.619e-05 | -10.55 | regulation of cytokine secretion | biological process | GO:0050707 | 152 | 11 | 13711 | 214 | Mmp8,Il17ra,Casp4,Tnfaip3,Tnf,Il4ra,Il1a,Akap12,Il6,Cd14,Lgals9 | | 2.627e-05 | -10.55 | MODULE\_241 | MSigDB lists | MODULE\_241 | 60 | 7 | 12187 | 179 | Ier3,Spi1,C3ar1,Saa1,C5ar1,Cd14,S100a9 | | 2.627e-05 | -10.55 | GO\_REGULATION\_OF\_LEUKOCYTE\_CHEMOTAXIS | MSigDB lists | GO\_REGULATION\_OF\_LEUKOCYTE\_CHEMOTAXIS | 60 | 7 | 12187 | 179 | C3ar1,C5ar1,Cxcl1,Thbs1,Ccl4,Il6,Ccl12 | | 2.642e-05 | -10.54 | GO\_REGULATION\_OF\_ERK1\_AND\_ERK2\_CASCADE | MSigDB lists | GO\_REGULATION\_OF\_ERK1\_AND\_ERK2\_CASCADE | 162 | 11 | 12187 | 179 | C5ar1,Tnf,Ccl2,Ccl4,Il6,Ccl12,Tnip1,Icam1,Errfi1,Ptpn1,Atf3 | | 2.642e-05 | -10.54 | GSE2405\_0H\_VS\_3H\_A\_PHAGOCYTOPHILUM\_STIM\_NEUTROPHIL\_UP | MSigDB lists | GSE2405\_0H\_VS\_3H\_A\_PHAGOCYTOPHILUM\_STIM\_NEUTROPHIL\_UP | 162 | 11 | 12187 | 179 | Col4a1,Timp1,Csf2rb,Ifitm2,Cybb,Casp4,Nfkbia,Tgm2,Tor3a,Adamts9,Rhoj | | 2.642e-05 | -10.54 | GO\_CELLULAR\_RESPONSE\_TO\_EXTRACELLULAR\_STIMULUS | MSigDB lists | GO\_CELLULAR\_RESPONSE\_TO\_EXTRACELLULAR\_STIMULUS | 162 | 11 | 12187 | 179 | Icam1,Ifi211,Rrp8,Ifi204,Ifi207,Atf3,Nfe2l2,Cdkn1a,Ifi209,Adamts1,Hspa5 | | 2.642e-05 | -10.54 | GSE3039\_ALPHAALPHA\_VS\_ALPHABETA\_CD8\_TCELL\_UP | MSigDB lists | GSE3039\_ALPHAALPHA\_VS\_ALPHABETA\_CD8\_TCELL\_UP | 162 | 11 | 12187 | 179 | Il6,Sdc4,Gpr84,Ccl4,Il4ra,Maff,Ptges,Ier3,Socs3,Cdkn1a,Cd14 | | 2.642e-05 | -10.54 | GSE557\_CIITA\_KO\_VS\_I\_AB\_KO\_DC\_UP | MSigDB lists | GSE557\_CIITA\_KO\_VS\_I\_AB\_KO\_DC\_UP | 162 | 11 | 12187 | 179 | Slfn4,Slfn3,Sbno2,Gbp3,Gbp7,Ncf1,Samd9l,Map3k8,Lgals9,Xaf1,Socs3 | | 2.642e-05 | -10.54 | GSE40277\_EOS\_AND\_LEF1\_TRANSDUCED\_VS\_CTRL\_CD4\_TCELL\_UP | MSigDB lists | GSE40277\_EOS\_AND\_LEF1\_TRANSDUCED\_VS\_CTRL\_CD4\_TCELL\_UP | 162 | 11 | 12187 | 179 | Gbp4,Birc3,Aff1,Casp4,Parp14,Icam1,Irf9,Clic4,Irf2,Gbp6,Bcl3 | | 2.650e-05 | -10.54 | Tnfaip3 (tumor necrosis factor, alpha-induced protein 3) | protein interactions | 21929 | 22 | 5 | 6802 | 115 | Tnfaip3,Ripk1,Tnf,Tnip3,Tnip1 | | 2.706e-05 | -10.52 | DORN\_ADENOVIRUS\_INFECTION\_12HR\_DN | MSigDB lists | DORN\_ADENOVIRUS\_INFECTION\_12HR\_DN | 25 | 5 | 12187 | 179 | Sgk1,Nfkbia,Nfe2l2,Ier3,Il6 | | 2.709e-05 | -10.52 | LEE\_LIVER\_CANCER\_MYC\_E2F1\_UP | MSigDB lists | LEE\_LIVER\_CANCER\_MYC\_E2F1\_UP | 41 | 6 | 12187 | 179 | Ifi44,Ifit2,Usp18,Tagln2,Icam1,Lcn2 | | 2.709e-05 | -10.52 | LIAN\_LIPA\_TARGETS\_3M | MSigDB lists | LIAN\_LIPA\_TARGETS\_3M | 41 | 6 | 12187 | 179 | Igsf6,S100a9,Cxcl1,C3ar1,Mmp8,Msr1 | | 2.709e-05 | -10.52 | ZHENG\_IL22\_SIGNALING\_UP | MSigDB lists | ZHENG\_IL22\_SIGNALING\_UP | 41 | 6 | 12187 | 179 | S100a9,Cd14,S100a8,Sbno2,Hp,Timp1 | | 2.712e-05 | -10.52 | ZHU\_CMV\_ALL\_UP | MSigDB lists | ZHU\_CMV\_ALL\_UP | 107 | 9 | 12187 | 179 | Irf2,Tap1,Isg15,Ptgs2,Nfkb1,Ripk1,Tnfaip3,Il6,Nfkbia | | 2.801e-05 | -10.48 | GO\_RESPONSE\_TO\_ORGANIC\_CYCLIC\_COMPOUND | MSigDB lists | GO\_RESPONSE\_TO\_ORGANIC\_CYCLIC\_COMPOUND | 694 | 25 | 12187 | 179 | P2ry6,Irak3,Selp,Tiparp,Nfkb1,Sgk1,Thbs1,Pygm,Ptges,Il6,Ccl12,Msr1,Il4ra,Trim25,Icam1,Msn,Errfi1,Ptgs2,Il1rn,Cdkn1a,Adamts1,Vcam1,Socs3,Tnf,Nfkbia | | 2.825e-05 | -10.47 | regulation of hydrolase activity | biological process | GO:0051336 | 948 | 32 | 13711 | 214 | Iqgap1,S100a8,A2m,Ccl12,Dnajb1,Angptl4,Wfdc21,Serpina3f,S100a9,Xdh,Tnfsf10,Ccl2,Tnf,Plek,Rhoc,Sele,Tnfaip8,Cflar,Timp1,Birc3,Ptgs2,Plaur,Rasip1,Ccl4,Il6,Oasl2,Icam1,Casp4,Rgs16,Thbs1,Oasl1,Ccl7 | | 2.845e-05 | -10.47 | RUTELLA\_RESPONSE\_TO\_HGF\_UP | MSigDB lists | RUTELLA\_RESPONSE\_TO\_HGF\_UP | 330 | 16 | 12187 | 179 | Sgk1,Tnip3,Il1a,Gadd45g,Hspb1,Rbm47,Tgm2,Ccl2,Sdc4,Runx1,Birc3,Ccl12,Cybb,Cflar,C3ar1,Tnfaip8 | | 2.860e-05 | -10.46 | positive regulation of wound healing | biological process | GO:0090303 | 39 | 6 | 13711 | 214 | S100a9,Nfe2l2,Thbs1,Selp,Plek,Ccl2 | | 2.882e-05 | -10.45 | GO\_REGULATION\_OF\_PROTEOLYSIS | MSigDB lists | GO\_REGULATION\_OF\_PROTEOLYSIS | 525 | 21 | 12187 | 179 | Plaur,Ifi207,Ifi204,Timp1,Thbs1,Ifi209,Tnfsf10,Xdh,S100a8,Tnfaip8,A2m,S100a9,Ifi211,C3ar1,C5ar1,Il6,Nfe2l2,Ripk1,Trib1,Birc3,Tnf | | 2.892e-05 | -10.45 | GO\_NEGATIVE\_REGULATION\_OF\_GENE\_EXPRESSION | MSigDB lists | GO\_NEGATIVE\_REGULATION\_OF\_GENE\_EXPRESSION | 1113 | 34 | 12187 | 179 | Ifi204,Ifi207,Rasd1,Atf3,Cdkn1a,Ifi209,Zbtb16,Zfp36,Bach1,Zfp189,Tnf,Birc3,Trib1,Nfkbia,Nfkb2,Xdh,A2m,Irf7,Nfkb1,Tiparp,Mxd4,Sox11,Thbs1,Irak3,Irf2,Ifitm3,Tnfaip3,Ccl4,Spi1,Sbno2,Ifi211,Arid5b,Rrp8,Bcl3 | | 2.901e-05 | -10.45 | UROSEVIC\_RESPONSE\_TO\_IMIQUIMOD | MSigDB lists | UROSEVIC\_RESPONSE\_TO\_IMIQUIMOD | 13 | 4 | 12187 | 179 | Oasl1,Il6,Isg15,Irf7 | | 2.901e-05 | -10.45 | GO\_TOLL\_LIKE\_RECEPTOR\_4\_SIGNALING\_PATHWAY | MSigDB lists | GO\_TOLL\_LIKE\_RECEPTOR\_4\_SIGNALING\_PATHWAY | 13 | 4 | 12187 | 179 | Pik3ap1,Tnip3,Cd14,Nfkbia | | 2.901e-05 | -10.45 | TSAI\_DNAJB4\_TARGETS\_UP | MSigDB lists | TSAI\_DNAJB4\_TARGETS\_UP | 13 | 4 | 12187 | 179 | Irf9,Ifit3b,Cdkn1a,Isg15 | | 2.931e-05 | -10.44 | ST\_FAS\_SIGNALING\_PATHWAY | MSigDB lists | ST\_FAS\_SIGNALING\_PATHWAY | 61 | 7 | 12187 | 179 | Nfkbie,Hspb1,Nfkb2,Nfkbia,Il1a,Nfkb1,Ripk1 | | 2.931e-05 | -10.44 | PID\_REG\_GR\_PATHWAY | MSigDB lists | PID\_REG\_GR\_PATHWAY | 61 | 7 | 12187 | 179 | Cdkn1a,Sgk1,Spi1,Nfkb1,Il6,Icam1,Sele | | 2.931e-05 | -10.44 | GNF2\_CD53 | MSigDB lists | GNF2\_CD53 | 61 | 7 | 12187 | 179 | Tap1,Ifi204,Ifi207,Ifi211,Phf11d,Phf11b,Ifi209 | | 2.935e-05 | -10.44 | GO\_CELLULAR\_CHEMICAL\_HOMEOSTASIS | MSigDB lists | GO\_CELLULAR\_CHEMICAL\_HOMEOSTASIS | 406 | 18 | 12187 | 179 | Saa1,Sgk3,Clic4,Icam1,Sgk1,Lcn2,Cp,Kcna5,S100a9,C3ar1,C5ar1,Pygm,S100a8,Ccl2,Tgm2,Pik3r1,Ccl12,Slc24a4 | | 2.950e-05 | -10.43 | response to interleukin-6 | biological process | GO:0070741 | 24 | 5 | 13711 | 214 | Spi1,Nfkb1,Il6,Selp,Sbno2 | | 2.952e-05 | -10.43 | THUM\_SYSTOLIC\_HEART\_FAILURE\_UP | MSigDB lists | THUM\_SYSTOLIC\_HEART\_FAILURE\_UP | 331 | 16 | 12187 | 179 | Clic4,Selp,Cd14,Cp,Ms4a6d,Thbs1,Vcam1,Tnfsf10,C3ar1,Cxcl1,S100a9,Oasl1,Gadd45b,Msr1,Ccl4,Tgm2 | | 2.961e-05 | -10.43 | GSE46606\_IRF4HIGH\_VS\_WT\_CD40L\_IL2\_IL5\_DAY1\_STIMULATED\_BCELL\_DN | MSigDB lists | GSE46606\_IRF4HIGH\_VS\_WT\_CD40L\_IL2\_IL5\_DAY1\_STIMULATED\_BCELL\_DN | 164 | 11 | 12187 | 179 | Clic4,Parp9,Gbp7,Ptges,Slfn5,Znfx1,Maff,Aff1,Nfkbiz,Casp4,Vcam1 | | 2.961e-05 | -10.43 | HALLMARK\_KRAS\_SIGNALING\_UP | MSigDB lists | HALLMARK\_KRAS\_SIGNALING\_UP | 164 | 11 | 12187 | 179 | Trib1,Birc3,Ccnd2,Tnfaip3,Gadd45g,Akap12,Angptl4,Ptgs2,C3ar1,Rgs16,Plaur | | 2.998e-05 | -10.41 | regulation of vasculature development | biological process | GO:1901342 | 277 | 15 | 13711 | 214 | C5ar1,Adamts1,Rhoj,Nfe2l2,Ptgs2,Runx1,Il1a,Ccl2,Cybb,Xdh,Cflar,Adamts9,Hspb1,C3ar1,Thbs1 | | 3.010e-05 | -10.41 | Signaling by Interleukins | REACTOME pathways | R-MMU-449147 | 299 | 16 | 6297 | 105 | Il4ra,Il6,Il1rn,Il1a,Socs3,Nfkb2,Il17ra,Ksr1,Csf2rb,Nfkb1,Lgals9,Map3k8,Pik3r1,Irak3,Osmr,Csf3 | | 3.029e-05 | -10.40 | GO\_POSITIVE\_REGULATION\_OF\_CELL\_DEATH | MSigDB lists | GO\_POSITIVE\_REGULATION\_OF\_CELL\_DEATH | 486 | 20 | 12187 | 179 | Tgm2,Tnf,Ifit2,Gadd45b,Il6,Ripk1,Hcar2,S100a9,Tnfsf10,Xdh,S100a8,Zbtb16,Thbs1,Cdkn1a,Atf3,Hp,Gadd45g,Ptgs2,Plaur,Ip6k2 | | 3.062e-05 | -10.39 | GO\_WOUND\_HEALING | MSigDB lists | GO\_WOUND\_HEALING | 332 | 16 | 12187 | 179 | Saa1,Irf2,Plaur,Plek,Hspb1,Il1a,Timp1,A2m,Pik3r5,Cflar,S100a8,Sdc4,Maff,Pik3r1,Il6,Rhoc | | 3.122e-05 | -10.37 | GO\_NEGATIVE\_REGULATION\_OF\_DNA\_BINDING | MSigDB lists | GO\_NEGATIVE\_REGULATION\_OF\_DNA\_BINDING | 42 | 6 | 12187 | 179 | Sox11,Ifi209,Ifi211,Nfkbia,Ifi204,Ifi207 | | 3.122e-05 | -10.37 | ZHOU\_TNF\_SIGNALING\_30MIN | MSigDB lists | ZHOU\_TNF\_SIGNALING\_30MIN | 42 | 6 | 12187 | 179 | Tnfaip3,Il6,Hspb1,Tagln2,Nfkbia,Ptgs2 | | 3.129e-05 | -10.37 | GO\_NEGATIVE\_REGULATION\_OF\_CYTOKINE\_PRODUCTION | MSigDB lists | GO\_NEGATIVE\_REGULATION\_OF\_CYTOKINE\_PRODUCTION | 136 | 10 | 12187 | 179 | Isg15,Bcl3,Irak3,Trim25,Errfi1,Tnf,Thbs1,Nfkb1,Tnfaip3,Il6 | | 3.132e-05 | -10.37 | GSE20715\_0H\_VS\_48H\_OZONE\_TLR4\_KO\_LUNG\_DN | MSigDB lists | GSE20715\_0H\_VS\_48H\_OZONE\_TLR4\_KO\_LUNG\_DN | 165 | 11 | 12187 | 179 | Osmr,Ptpn1,Cd14,Cxcl1,Gadd45g,Lcn2,Thbs1,Cdkn1a,Rhou,Dnajb1,Socs3 | | 3.132e-05 | -10.37 | GSE39152\_CD103\_NEG\_VS\_POS\_MEMORY\_CD8\_TCELL\_DN | MSigDB lists | GSE39152\_CD103\_NEG\_VS\_POS\_MEMORY\_CD8\_TCELL\_DN | 165 | 11 | 12187 | 179 | C5ar1,Ccl4,Il4ra,Ptpn1,Zfp36,Ncf1,Ier3,Gadd45g,Timp1,Tiparp,Socs3 | | 3.132e-05 | -10.37 | GSE29618\_MONOCYTE\_VS\_MDC\_DAY7\_FLU\_VACCINE\_UP | MSigDB lists | GSE29618\_MONOCYTE\_VS\_MDC\_DAY7\_FLU\_VACCINE\_UP | 165 | 11 | 12187 | 179 | Cxcl1,Cd14,S100a9,C5ar1,C3ar1,Irak3,Icam1,S100a8,Nfkbia,Ier3,Cybb | | 3.132e-05 | -10.37 | GSE11961\_MARGINAL\_ZONE\_BCELL\_VS\_MEMORY\_BCELL\_DAY7\_UP | MSigDB lists | GSE11961\_MARGINAL\_ZONE\_BCELL\_VS\_MEMORY\_BCELL\_DAY7\_UP | 165 | 11 | 12187 | 179 | Slfn9,Slfn5,Herc6,Irf7,Rgs16,Cmpk2,Trib1,Usp18,Tor3a,Xaf1,Ifit2 | | 3.137e-05 | -10.37 | GO\_CELL\_MOTILITY | MSigDB lists | GO\_CELL\_MOTILITY | 612 | 23 | 12187 | 179 | Thbs1,Cxcl16,Parp9,Il17ra,Selp,Ccl12,Il6,Rhoc,Ccl4,Ccl2,Pik3r1,S100a8,Cxcl1,Arid5b,Vcam1,Msn,Icam1,Sele,Saa1,Sdc4,Tnf,S100a9,C5ar1 | | 3.226e-05 | -10.34 | Adaptive Immune System | REACTOME pathways | R-MMU-1280218 | 442 | 20 | 6297 | 105 | Tap1,Vcam1,Cdkn1a,Icam1,Dtx3l,Rnf213,Ifitm3,Nfkbie,Cybb,Pik3ap1,Pik3r1,Ncf1,Map3k8,Nfkbia,Fyb,Nfkb1,Herc6,Xdh,Ifitm2,Ier3 | | 3.261e-05 | -10.33 | Interferon alpha/beta signaling | REACTOME pathways | R-MMU-909733 | 12 | 4 | 6297 | 105 | Usp18,Ptpn1,Irf9,Socs3 | | 3.310e-05 | -10.32 | GO\_MYD88\_INDEPENDENT\_TOLL\_LIKE\_RECEPTOR\_SIGNALING\_PATHWAY | MSigDB lists | GO\_MYD88\_INDEPENDENT\_TOLL\_LIKE\_RECEPTOR\_SIGNALING\_PATHWAY | 26 | 5 | 12187 | 179 | Irf7,Cd14,Birc3,Ripk1,Tnip3 | | 3.310e-05 | -10.32 | GNF2\_SPI1 | MSigDB lists | GNF2\_SPI1 | 26 | 5 | 12187 | 179 | Spi1,C5ar1,Tnfsf10,Igsf6,Bach1 | | 3.312e-05 | -10.32 | GSE40274\_FOXP3\_VS\_FOXP3\_AND\_GATA1\_TRANSDUCED\_ACTIVATED\_CD4\_TCELL\_DN | MSigDB lists | GSE40274\_FOXP3\_VS\_FOXP3\_AND\_GATA1\_TRANSDUCED\_ACTIVATED\_CD4\_TCELL\_DN | 166 | 11 | 12187 | 179 | Irf9,Ifitm2,Herc6,Rnf213,Ncf1,Gadd45g,Ifi44,Bcl3,Irf7,Oasl1,Birc3 | | 3.312e-05 | -10.32 | GSE14769\_UNSTIM\_VS\_240MIN\_LPS\_BMDM\_DN | MSigDB lists | GSE14769\_UNSTIM\_VS\_240MIN\_LPS\_BMDM\_DN | 166 | 11 | 12187 | 179 | Il1rn,Atf3,Cdkn1a,Map3k8,Tor3a,Tgm2,Irf9,Csf2rb,Clic4,Gbp7,Slfn5 | | 3.333e-05 | -10.31 | GO\_INTRINSIC\_APOPTOTIC\_SIGNALING\_PATHWAY | MSigDB lists | GO\_INTRINSIC\_APOPTOTIC\_SIGNALING\_PATHWAY | 137 | 10 | 12187 | 179 | Casp4,Pik3r1,Cdkn1a,Tnf,Ifi209,Ifi207,Ifi204,Bcl3,Rrp8,Ifi211 | | 3.333e-05 | -10.31 | GSE2706\_2H\_VS\_8H\_R848\_AND\_LPS\_STIM\_DC\_DN | MSigDB lists | GSE2706\_2H\_VS\_8H\_R848\_AND\_LPS\_STIM\_DC\_DN | 137 | 10 | 12187 | 179 | Isg15,Herc6,Ifitm3,Phf11d,Irf9,Parp9,Birc3,Phf11b,Xaf1,AA467197 | | 3.336e-05 | -10.31 | cellular process | biological process | GO:0009987 | 10077 | 182 | 13711 | 214 | Msr1,Iigp1,Bbs12,Lgals9,Gadd45g,Rgs16,Nfkb2,Fyb,Mt1,Rtp4,Birc3,Map3k8,Rhoj,Tnfaip8,Luc7l3,Aff1,Znfx1,Tnfaip3,Ccl2,Depp1,Vcam1,Cxcl16,Tnip3,Iqgap1,Col4a1,Ccl7,Ccnd2,Akap12,Il4ra,Slfn9,Ip6k2,Gm5431,Tubb6,Mx2,Cebpd,Rasip1,Xaf1,Trim25,Runx1,Sele,Ncf1,Trim30a,Cp,Acod1,Atf3,Mmp8,Nfe2l2,Noct,Angptl4,Clic4,Tgm2,C5ar1,Maff,Gm4841,Ifitm3,Thbs1,P2ry6,Cemip2,Errfi1,Kcna5,Bach1,Plaur,Trim56,Slfn5,Ifi204,Plek,Ksr1,Xdh,Usp18,Sgk3,Irgm2,Gbp6,Bcl3,Gbp3,Ccl12,Trib1,Socs3,Gem,Il1a,Tgtp2,Cdkn1a,Ccl4,Rbm47,Ifitm6,Ier3,Sgk1,Ptges,Sbno2,Parp9,Cflar,Cd14,Slc24a4,Rhoc,Fstl1,Robo4,Tnf,Ifit2,Cxcl1,Icam1,Rassf4,Irgm1,Pik3r5,Zbtb16,Csf3,Il1rn,C3ar1,Lcn2,Retnlg,Cxcl9,S100a9,Wfdc21,Ifitm2,Ptpn1,Nfkbiz,Parp14,Hcar2,Nfkbia,Herc6,Ifi211,Fam107a,Map3k6,Tiparp,Timp1,Isg15,Rnf213,Pik3ap1,Rhou,Rasd1,Olfml2b,Gbp7,Selp,Msn,Hspa5,Mx1,Banp,Rbm39,Sox11,Cybb,Il17ra,Osmr,Gbp4,Tagln2,F830016B08Rik,Adamts1,Nfkb1,Ifi47,Adamts9,Arid5b,Pygm,Irak3,Parp12,Tnfsf10,Samd9l,A2m,S100a8,Irf7,Dtx3l,Pik3r1,Gadd45b,Casp4,Oasl2,Il6,Spi1,Stx11,Ptgs2,Fzd4,Ifi209,Sdc4,Tgtp1,Cmpk2,Gpr84,Ripk1,Hspb1,Ifi207,Tnip1,Saa1,Zfp36,Csf2rb,Rrp8,Dnajb1,Mt2,Hp | | 3.397e-05 | -10.29 | G\_GB1\_RHD3 | prosite domains | PS51715 | 11 | 4 | 8845 | 164 | Gbp4,Gbp6,Gbp3,Gbp7 | | 3.397e-05 | -10.29 | SMALL\_CYTOKINES\_CC | prosite domains | PS00472 | 11 | 4 | 8845 | 164 | Ccl2,Ccl12,Ccl7,Ccl4 | | 3.405e-05 | -10.29 | GOZGIT\_ESR1\_TARGETS\_DN | MSigDB lists | GOZGIT\_ESR1\_TARGETS\_DN | 531 | 21 | 12187 | 179 | Tnfsf10,Arid5b,Irf7,Tnfaip3,Ifit2,Birc3,Xaf1,Tgm2,Usp18,Cxcl16,Irf9,Herc6,Timp1,Samd9l,Cp,Rasd1,Lcn2,Thbs1,Sgk1,Nfkbiz,Ifit3b | | 3.405e-05 | -10.29 | positive regulation of apoptotic process | biological process | GO:0043065 | 536 | 22 | 13711 | 214 | Il6,Ip6k2,Casp4,Akap12,Gadd45b,Gadd45g,Hcar2,Thbs1,Ptgs2,Plaur,Xdh,Atf3,Tnfsf10,Tnf,Ripk1,Tnfaip8,Zbtb16,S100a8,Ccl12,Tgm2,Ifit2,S100a9 | | 3.469e-05 | -10.27 | interspecies interaction between organisms | biological process | GO:0044419 | 216 | 13 | 13711 | 214 | Gbp6,Irgm2,Gbp7,Ccl4,S100a9,Irgm1,Vcam1,Gbp3,Tnip1,Ripk1,Ncf1,Ccl2,Icam1 | | 3.484e-05 | -10.26 | DAPIN | interpro domains | IPR004020 | 13 | 4 | 13788 | 212 | Ifi209,Ifi211,Ifi204,Ifi207 | | 3.484e-05 | -10.26 | Chemokine\_b/g/d | interpro domains | IPR039809 | 13 | 4 | 13788 | 212 | Ccl12,Ccl4,Ccl2,Ccl7 | | 3.501e-05 | -10.26 | GSE18893\_TCONV\_VS\_TREG\_24H\_CULTURE\_DN | MSigDB lists | GSE18893\_TCONV\_VS\_TREG\_24H\_CULTURE\_DN | 167 | 11 | 12187 | 179 | Irf7,Bcl3,Rnf213,Cflar,Cmpk2,Ifit3b,Sdc4,Ifi44,Ifit2,Oasl1,Akap12 | | 3.501e-05 | -10.26 | GSE16385\_ROSIGLITAZONE\_IL4\_VS\_IL4\_ALONE\_STIM\_MACROPHAGE\_12H\_UP | MSigDB lists | GSE16385\_ROSIGLITAZONE\_IL4\_VS\_IL4\_ALONE\_STIM\_MACROPHAGE\_12H\_UP | 167 | 11 | 12187 | 179 | Zfp36,Rbm47,Csf2rb,Tap1,Phf11d,Tubb6,Kcna5,Ms4a6d,Parp12,Phf11b,Pik3r1 | | 3.505e-05 | -10.26 | response to corticosteroid | biological process | GO:0031960 | 59 | 7 | 13711 | 214 | Il1rn,Sgk1,Pik3r1,Fam107a,Tnf,Zfp36,Il6 | | 3.549e-05 | -10.25 | KEGG\_CHEMOKINE\_SIGNALING\_PATHWAY | MSigDB lists | KEGG\_CHEMOKINE\_SIGNALING\_PATHWAY | 138 | 10 | 12187 | 179 | Cxcl16,Ncf1,Cxcl1,Pik3r5,Nfkb1,Ccl12,Pik3r1,Ccl2,Nfkbia,Ccl4 | | 3.585e-05 | -10.24 | GO\_POSITIVE\_REGULATION\_OF\_TUMOR\_NECROSIS\_FACTOR\_SUPERFAMILY\_CYTOKINE\_PRODUCTION | MSigDB lists | GO\_POSITIVE\_REGULATION\_OF\_TUMOR\_NECROSIS\_FACTOR\_SUPERFAMILY\_CYTOKINE\_PRODUCTION | 43 | 6 | 12187 | 179 | Hspb1,Ripk1,Ccl12,Thbs1,Pik3r1,Cd14 | | 3.611e-05 | -10.23 | GO\_NEGATIVE\_REGULATION\_OF\_HYDROLASE\_ACTIVITY | MSigDB lists | GO\_NEGATIVE\_REGULATION\_OF\_HYDROLASE\_ACTIVITY | 265 | 14 | 12187 | 179 | Tnfaip8,Iqgap1,A2m,Angptl4,Ifi211,Il6,Birc3,Tnf,Plaur,Ifi207,Ifi204,Timp1,Thbs1,Ifi209 | | 3.631e-05 | -10.22 | FULCHER\_INFLAMMATORY\_RESPONSE\_LECTIN\_VS\_LPS\_UP | MSigDB lists | FULCHER\_INFLAMMATORY\_RESPONSE\_LECTIN\_VS\_LPS\_UP | 452 | 19 | 12187 | 179 | Tagln2,P2ry6,Plaur,Il1a,Gadd45g,Il1rn,Nfkbie,Cxcl1,Igsf6,Birc3,Maff,Runx1,Ccl2,Spi1,Tgm2,Tubb6,Cybb,Rhoc,Il6 | | 3.664e-05 | -10.21 | positive regulation of interferon-gamma-mediated signaling pathway | biological process | GO:0060335 | 5 | 3 | 13711 | 214 | Irgm1,Irgm2,Parp9 | | 3.664e-05 | -10.21 | positive regulation of response to interferon-gamma | biological process | GO:0060332 | 5 | 3 | 13711 | 214 | Irgm2,Parp9,Irgm1 | | 3.678e-05 | -10.21 | blood vessel morphogenesis | biological process | GO:0048514 | 351 | 17 | 13711 | 214 | Rnf213,Thbs1,Adamts9,Cemip2,Ccl2,Robo4,Angptl4,Ptgs2,Rasip1,Spi1,Col4a1,Apold1,Rhoj,Ccl12,Tiparp,Fzd4,Clic4 | | 3.685e-05 | -10.21 | PYRIN | pfam domains | PF02758 | 13 | 4 | 12881 | 201 | Ifi204,Ifi207,Ifi211,Ifi209 | | 3.694e-05 | -10.21 | adhesion of symbiont to host | biological process | GO:0044406 | 13 | 4 | 13711 | 214 | Gbp7,Gbp6,Icam1,Gbp3 | | 3.699e-05 | -10.20 | GSE17721\_12H\_VS\_24H\_POLYIC\_BMDC\_UP | MSigDB lists | GSE17721\_12H\_VS\_24H\_POLYIC\_BMDC\_UP | 168 | 11 | 12187 | 179 | Usp18,Aff1,Gadd45b,Il6,S100a9,Cxcl1,Ptges,Tnip1,Slfn9,Nfkb2,Igsf6 | | 3.699e-05 | -10.20 | GSE17721\_0.5H\_VS\_4H\_GARDIQUIMOD\_BMDC\_DN | MSigDB lists | GSE17721\_0.5H\_VS\_4H\_GARDIQUIMOD\_BMDC\_DN | 168 | 11 | 12187 | 179 | Msn,Plaur,Il17ra,Atf3,Fstl1,Arid5b,Nfkbia,Tor3a,Tgm2,Birc3,Zfp189 | | 3.699e-05 | -10.20 | GSE42724\_NAIVE\_VS\_MEMORY\_BCELL\_DN | MSigDB lists | GSE42724\_NAIVE\_VS\_MEMORY\_BCELL\_DN | 168 | 11 | 12187 | 179 | Clic4,Parp9,Slfn9,Sgk3,Luc7l3,Nfkbiz,Aff1,Gbp7,Sdc4,Gpr84,Casp4 | | 3.699e-05 | -10.20 | GSE40274\_CTRL\_VS\_FOXP3\_TRANSDUCED\_ACTIVATED\_CD4\_TCELL\_DN | MSigDB lists | GSE40274\_CTRL\_VS\_FOXP3\_TRANSDUCED\_ACTIVATED\_CD4\_TCELL\_DN | 168 | 11 | 12187 | 179 | Sgk1,Parp14,Sdc4,Samd9l,Bach1,Irgm2,Gbp7,Ncf1,Arid5b,Irak3,Trim25 | | 3.699e-05 | -10.20 | GSE17721\_CTRL\_VS\_CPG\_8H\_BMDC\_DN | MSigDB lists | GSE17721\_CTRL\_VS\_CPG\_8H\_BMDC\_DN | 168 | 11 | 12187 | 179 | Hcar2,Ccl12,Gbp4,Parp14,Dnajb1,Nfkbia,Rbm47,Gbp6,Ptges,Tagln2,Arid5b | | 3.776e-05 | -10.18 | TONKS\_TARGETS\_OF\_RUNX1\_RUNX1T1\_FUSION\_HSC\_DN | MSigDB lists | TONKS\_TARGETS\_OF\_RUNX1\_RUNX1T1\_FUSION\_HSC\_DN | 139 | 10 | 12187 | 179 | Il17ra,Csf2rb,Cflar,S100a9,Il1rn,Cybb,Ccl12,Birc3,Spi1,Zbtb16 | | 3.776e-05 | -10.18 | GSE42021\_CD24INT\_VS\_CD24LOW\_TCONV\_THYMUS\_UP | MSigDB lists | GSE42021\_CD24INT\_VS\_CD24LOW\_TCONV\_THYMUS\_UP | 139 | 10 | 12187 | 179 | Zfp36,Icam1,Pygm,Ptpn1,Bcl3,Arid5b,Cxcl1,Cebpd,Ccl12,Maff | | 3.776e-05 | -10.18 | GSE11961\_FOLLICULAR\_BCELL\_VS\_PLASMA\_CELL\_DAY7\_UP | MSigDB lists | GSE11961\_FOLLICULAR\_BCELL\_VS\_PLASMA\_CELL\_DAY7\_UP | 139 | 10 | 12187 | 179 | Nfkbiz,Ccl4,Tgm2,Socs3,Maff,Slfn9,Nfkbie,Isg15,Adamts9,Icam1 | | 3.820e-05 | -10.17 | adaptive immune response based on somatic recombination of immune receptors built from immunoglobulin superfamily domains | biological process | GO:0002460 | 105 | 9 | 13711 | 214 | Nfkb2,Il4ra,Icam1,Il6,Stx11,Irf7,C3ar1,Bcl3,Gadd45g | | 3.878e-05 | -10.16 | CREL\_01 | MSigDB lists | CREL\_01 | 200 | 12 | 12187 | 179 | Bcl3,Ptges,Nfkb2,Birc3,Map3k8,Nfkbia,Sdc4,Gadd45b,Tnip1,Icam1,Csf2rb,Ier3 | | 3.887e-05 | -10.16 | Death Receptor Signalling | REACTOME pathways | R-MMU-73887 | 39 | 6 | 6297 | 105 | Tnfsf10,Ripk1,Tnf,Birc3,Cflar,Tnfaip3 | | 3.891e-05 | -10.15 | regulation of developmental process | biological process | GO:0050793 | 2184 | 57 | 13711 | 214 | Tnf,Rhou,Zfp36,Ripk1,Cflar,Hspb1,Rhoc,Hspa5,Cxcl1,C5ar1,Maff,Nfe2l2,Msn,Noct,Nfkbia,Il1a,Il4ra,Cdkn1a,Il6,Ctla2a,Trib1,Socs3,Pik3r1,Fzd4,Timp1,Isg15,Spi1,Cebpd,Runx1,Sgk1,Ptgs2,Cxcl9,Tnfaip3,Ccl2,Xdh,Zbtb16,Adamts9,Csf3,Irak3,Il1rn,C3ar1,Ifi204,Nfkbiz,Ccl12,Iqgap1,S100a9,Sox11,Icam1,Cybb,Errfi1,Bbs12,Msr1,Thbs1,Lgals9,Adamts1,Apold1,Rhoj | | 3.907e-05 | -10.15 | GSE5589\_LPS\_VS\_LPS\_AND\_IL6\_STIM\_IL6\_KO\_MACROPHAGE\_45MIN\_DN | MSigDB lists | GSE5589\_LPS\_VS\_LPS\_AND\_IL6\_STIM\_IL6\_KO\_MACROPHAGE\_45MIN\_DN | 169 | 11 | 12187 | 179 | Birc3,Ccnd2,Tgm2,S100a8,Iqgap1,C5ar1,Angptl4,Il1a,Samd9l,Icam1,Trim25 | | 3.921e-05 | -10.15 | RUTELLA\_RESPONSE\_TO\_CSF2RB\_AND\_IL4\_UP | MSigDB lists | RUTELLA\_RESPONSE\_TO\_CSF2RB\_AND\_IL4\_UP | 267 | 14 | 12187 | 179 | Cdkn1a,Il1a,Tnip3,Map3k6,Rbm47,Hspb1,Birc3,Runx1,Ccl2,Sdc4,Tgm2,Gadd45b,Cflar,Tnfaip8 | | 3.921e-05 | -10.15 | GO\_REGULATION\_OF\_MAP\_KINASE\_ACTIVITY | MSigDB lists | GO\_REGULATION\_OF\_MAP\_KINASE\_ACTIVITY | 267 | 14 | 12187 | 179 | Map3k6,Saa1,Ptpn1,Irak3,Thbs1,Gadd45g,Pik3r5,Fzd4,C5ar1,Trib1,Tnf,Map3k8,Ripk1,Gadd45b | | 3.970e-05 | -10.13 | DEATH-like\_dom\_sf | interpro domains | IPR011029 | 61 | 7 | 13788 | 212 | Birc3,Casp4,Irak3,Cflar,Nfkb1,Nfkb2,Ripk1 | | 4.015e-05 | -10.12 | GO\_CHEMOKINE\_RECEPTOR\_BINDING | MSigDB lists | GO\_CHEMOKINE\_RECEPTOR\_BINDING | 27 | 5 | 12187 | 179 | Cxcl16,Ccl12,Ccl4,Cxcl1,Ccl2 | | 4.015e-05 | -10.12 | GO\_POSITIVE\_REGULATION\_OF\_INTERLEUKIN\_1\_BETA\_PRODUCTION | MSigDB lists | GO\_POSITIVE\_REGULATION\_OF\_INTERLEUKIN\_1\_BETA\_PRODUCTION | 27 | 5 | 12187 | 179 | Ifi211,Ifi209,Hspb1,Ifi204,Ifi207 | | 4.015e-05 | -10.12 | GO\_REGULATION\_OF\_GRANULOCYTE\_CHEMOTAXIS | MSigDB lists | GO\_REGULATION\_OF\_GRANULOCYTE\_CHEMOTAXIS | 27 | 5 | 12187 | 179 | C5ar1,Thbs1,C3ar1,Cxcl1,Ccl12 | | 4.015e-05 | -10.12 | BOYAULT\_LIVER\_CANCER\_SUBCLASS\_G56\_DN | MSigDB lists | BOYAULT\_LIVER\_CANCER\_SUBCLASS\_G56\_DN | 14 | 4 | 12187 | 179 | Trib1,Maff,Il4ra,Ier3 | | 4.015e-05 | -10.12 | GRANDVAUX\_IRF3\_TARGETS\_UP | MSigDB lists | GRANDVAUX\_IRF3\_TARGETS\_UP | 14 | 4 | 12187 | 179 | Ifit3b,Ifit2,Isg15,Ifi44 | | 4.015e-05 | -10.12 | BIOCARTA\_INFLAM\_PATHWAY | MSigDB lists | BIOCARTA\_INFLAM\_PATHWAY | 14 | 4 | 12187 | 179 | Tnf,Csf3,Il1a,Il6 | | 4.015e-05 | -10.12 | GSE18804\_SPLEEN\_MACROPHAGE\_VS\_BRAIN\_TUMORAL\_MACROPHAGE\_UP | MSigDB lists | GSE18804\_SPLEEN\_MACROPHAGE\_VS\_BRAIN\_TUMORAL\_MACROPHAGE\_UP | 140 | 10 | 12187 | 179 | Cxcl1,Ptgs2,Ier3,Atf3,Ccl12,Tnfaip3,Sdc4,Nfkbia,Rhou,Map3k8 | | 4.028e-05 | -10.12 | GO\_POSITIVE\_REGULATION\_OF\_CHEMOTAXIS | MSigDB lists | GO\_POSITIVE\_REGULATION\_OF\_CHEMOTAXIS | 87 | 8 | 12187 | 179 | Thbs1,Ccl4,Il6,Ccl12,C3ar1,C5ar1,Cxcl1,Hspb1 | | 4.028e-05 | -10.12 | KEEN\_RESPONSE\_TO\_ROSIGLITAZONE\_DN | MSigDB lists | KEEN\_RESPONSE\_TO\_ROSIGLITAZONE\_DN | 87 | 8 | 12187 | 179 | Nfkbiz,Atf3,Gadd45b,Cxcl1,Cd14,S100a9,Slfn4,Slfn3 | | 4.058e-05 | -10.11 | GO\_NEGATIVE\_REGULATION\_OF\_CELL\_COMMUNICATION | MSigDB lists | GO\_NEGATIVE\_REGULATION\_OF\_CELL\_COMMUNICATION | 939 | 30 | 12187 | 179 | Tnfsf10,Xdh,Cflar,Tnf,Nfkbia,Trib1,Plek,Icam1,Hspb1,Plaur,Errfi1,Ptgs2,Tnip1,Tnip3,Il1rn,Atf3,Socs3,Rgs16,Il6,Nfe2l2,Ripk1,Tnfaip3,Pik3ap1,Irak3,Ptpn1,Rnf213,Cd14,Il1a,Hspa5,Thbs1 | | 4.101e-05 | -10.10 | PID\_IL4\_2PATHWAY | MSigDB lists | PID\_IL4\_2PATHWAY | 44 | 6 | 12187 | 179 | Spi1,Parp14,Socs3,Il4ra,Pik3r1,Selp | | 4.101e-05 | -10.10 | PID\_IL6\_7\_PATHWAY | MSigDB lists | PID\_IL6\_7\_PATHWAY | 44 | 6 | 12187 | 179 | Pik3r1,Socs3,A2m,Timp1,Cebpd,Il6 | | 4.125e-05 | -10.10 | GSE17721\_LPS\_VS\_PAM3CSK4\_12H\_BMDC\_UP | MSigDB lists | GSE17721\_LPS\_VS\_PAM3CSK4\_12H\_BMDC\_UP | 170 | 11 | 12187 | 179 | Cflar,Nfkbie,Rhoc,Sdc4,Nfkbia,Clic4,Tagln2,Znfx1,Cdkn1a,Sgk1,Vcam1 | | 4.125e-05 | -10.10 | GO\_LEUKOCYTE\_CELL\_CELL\_ADHESION | MSigDB lists | GO\_LEUKOCYTE\_CELL\_CELL\_ADHESION | 170 | 11 | 12187 | 179 | Sele,S100a8,Icam1,Msn,Selp,S100a9,Bcl3,Tnip1,Il6,Tnf,Vcam1 | | 4.125e-05 | -10.10 | GSE20754\_WT\_VS\_TCF1\_KO\_MEMORY\_CD8\_TCELL\_UP | MSigDB lists | GSE20754\_WT\_VS\_TCF1\_KO\_MEMORY\_CD8\_TCELL\_UP | 170 | 11 | 12187 | 179 | C3ar1,Pik3r5,Birc3,Maff,Nfe2l2,Gadd45b,Tnfaip3,Tnip1,Icam1,Il1a,Atf3 | | 4.125e-05 | -10.10 | GSE43955\_TH0\_VS\_TGFB\_IL6\_IL23\_TH17\_ACT\_CD4\_TCELL\_52H\_DN | MSigDB lists | GSE43955\_TH0\_VS\_TGFB\_IL6\_IL23\_TH17\_ACT\_CD4\_TCELL\_52H\_DN | 170 | 11 | 12187 | 179 | Ifitm3,Osmr,Zfp36,Il17ra,Clic4,Xdh,Rhou,Casp4,Sntb2,Cebpd,Ier3 | | 4.252e-05 | -10.07 | binding | molecular function | GO:0005488 | 9835 | 178 | 13516 | 211 | Aff1,Xaf1,Banp,Msn,Tnfaip3,Rgs16,Il1a,Nfe2l2,Zfp36,Xdh,Stx11,Ifit2,Adamts1,Cd14,Map3k6,Luc7l3,Trim30a,Gadd45b,Akap12,Sdc4,S100a9,A2m,Nfkbie,Fzd4,Map3k8,Timp1,Rhoj,Gbp6,Isg15,Rtp4,Fam107a,Csf3,Cflar,Ifit3b,Resf1,Cemip2,Mmp8,Irak3,Parp12,Oasl1,Ifi211,Casp4,Nfkb1,Retnlg,Sbno2,Oasl2,Lcn2,Ccnd2,Rbm47,Olfml2b,Tap1,Ptges,Usp18,Thbs1,Sele,Tnip1,Ier3,Tnf,Hcar2,Gbp3,Tnfsf10,Cp,Cxcl16,Rhou,Prg4,Herc6,Ifi207,Ksr1,C5ar1,Fyb,Tor3a,Cmpk2,Cxcl9,Nfkb2,Pygm,Gbp4,Gem,Kcna5,Cxcl1,Mx2,Noct,Apold1,Bach1,Irf7,Pik3r1,Atf3,Hspa5,Errfi1,Ifi204,Clic4,Bcl3,Zbtb16,Ncf1,Sgk1,Plaur,Il4ra,Cybb,Sox11,Dnajb1,Vcam1,Ifi209,Iigp1,Csf2rb,Rnf213,Runx1,Tiparp,Cdkn1a,Rrp8,Osmr,Nfkbia,Mt2,Fstl1,C3ar1,Robo4,Phf11d,Nfkbiz,Rasip1,Bbs12,Il6,Parp9,Arid5b,Hspb1,Apol10b,Irgm2,Sntb2,Ripk1,Dtx3l,P2ry6,Il1rn,S100a8,Msr1,Sgk3,Samd9l,Trim56,Pik3ap1,Rhoc,Mt1,Tgtp1,Trib1,Slfn5,Irf9,Slfn9,Rbm39,Tubb6,Socs3,Col4a1,Ccl4,Ip6k2,Irgm1,Angptl4,Znfx1,Ccl7,Cebpd,Icam1,Ptgs2,Selp,Saa1,Iqgap1,Tgm2,Tnip3,Mxd4,Parp14,Hp,Irf2,Maff,Spi1,Birc3,Ccl2,Ccl12,Rasd1,Trim25,Ptpn1,Lgals9,Gadd45g,Il17ra,Mx1,Gbp7,Plek | | 4.286e-05 | -10.06 | Spinal Cord Injury | WikiPathways | WP2432 | 84 | 10 | 3756 | 96 | Col4a1,Ccl2,Rhoc,Il6,Tnf,Il1a,Selp,Icam1,Cxcl1,Zfp36 | | 4.300e-05 | -10.05 | GO\_POSITIVE\_REGULATION\_OF\_PROTEIN\_SERINE\_THREONINE\_KINASE\_ACTIVITY | MSigDB lists | GO\_POSITIVE\_REGULATION\_OF\_PROTEIN\_SERINE\_THREONINE\_KINASE\_ACTIVITY | 235 | 13 | 12187 | 179 | Ptpn1,Saa1,Map3k6,Gadd45g,Thbs1,Iqgap1,C5ar1,Pik3r5,Fzd4,Ripk1,Gadd45b,Ccnd2,Tnf | | 4.353e-05 | -10.04 | GSE24726\_WT\_VS\_E2\_22\_KO\_PDC\_DAY4\_POST\_DELETION\_DN | MSigDB lists | GSE24726\_WT\_VS\_E2\_22\_KO\_PDC\_DAY4\_POST\_DELETION\_DN | 171 | 11 | 12187 | 179 | Irak3,Pik3r5,Ptges,Bcl3,Irf7,Cd14,Nfkbie,Tnfaip3,Cybb,Tgm2,Birc3 | | 4.353e-05 | -10.04 | GSE22935\_WT\_VS\_MYD88\_KO\_MACROPHAGE\_48H\_MBOVIS\_BCG\_STIM\_DN | MSigDB lists | GSE22935\_WT\_VS\_MYD88\_KO\_MACROPHAGE\_48H\_MBOVIS\_BCG\_STIM\_DN | 171 | 11 | 12187 | 179 | Tnfaip8,Tnfaip3,Rhoc,Cybb,Bach1,Nfkbia,P2ry6,Ncf1,Stx11,Fstl1,Socs3 | | 4.376e-05 | -10.04 | MARZEC\_IL2\_SIGNALING\_UP | MSigDB lists | MARZEC\_IL2\_SIGNALING\_UP | 88 | 8 | 12187 | 179 | Csf2rb,Tnfsf10,Tnfaip8,Ccnd2,Birc3,Ier3,Gadd45b,Il1a | | 4.392e-05 | -10.03 | regulation of apoptotic signaling pathway | biological process | GO:2001233 | 356 | 17 | 13711 | 214 | Thbs1,Hspb1,Ripk1,Cflar,Tnf,Tnfaip3,Icam1,Atf3,Tnfsf10,Sgk3,Nfe2l2,S100a9,Ptgs2,Ier3,Plaur,Ptpn1,S100a8 | | 4.451e-05 | -10.02 | antimicrobial humoral response | biological process | GO:0019730 | 26 | 5 | 13711 | 214 | Ccl2,Bcl3,S100a9,Cxcl9,Cxcl1 | | 4.453e-05 | -10.02 | MANTOVANI\_VIRAL\_GPCR\_SIGNALING\_UP | MSigDB lists | MANTOVANI\_VIRAL\_GPCR\_SIGNALING\_UP | 65 | 7 | 12187 | 179 | Iqgap1,Osmr,Cp,Ccl12,Saa1,Vcam1,Nfkbiz | | 4.533e-05 | -10.00 | GSE360\_T\_GONDII\_VS\_M\_TUBERCULOSIS\_DC\_DN | MSigDB lists | GSE360\_T\_GONDII\_VS\_M\_TUBERCULOSIS\_DC\_DN | 142 | 10 | 12187 | 179 | Cd14,C3ar1,Tap1,S100a8,Ccl4,Ifit3b,Hspa5,Ccl12,Atf3,Mmp8 | | 4.533e-05 | -10.00 | GSE13522\_WT\_VS\_IFNAR\_KO\_SKING\_T\_CRUZI\_Y\_STRAIN\_INF\_UP | MSigDB lists | GSE13522\_WT\_VS\_IFNAR\_KO\_SKING\_T\_CRUZI\_Y\_STRAIN\_INF\_UP | 142 | 10 | 12187 | 179 | Cmpk2,Trim25,Gbp3,Parp9,Irgm2,Ncf1,Stx11,Birc3,Nfkbiz,Trib1 | | 4.565e-05 | -9.99 | positive regulation of cell differentiation | biological process | GO:0045597 | 927 | 31 | 13711 | 214 | Cxcl9,Tnf,Zfp36,Adamts9,Ripk1,Csf3,Zbtb16,Cflar,Hspb1,Ifi204,Hspa5,Nfkbiz,Iqgap1,S100a9,Nfe2l2,Noct,Sox11,Il4ra,Il6,Trib1,Msr1,Socs3,Lgals9,Pik3r1,Fzd4,Adamts1,Isg15,Cebpd,Runx1,Sgk1,Ptgs2 | | 4.591e-05 | -9.99 | GSE1925\_CTRL\_VS\_IFNG\_PRIMED\_MACROPHAGE\_24H\_IFNG\_STIM\_DN | MSigDB lists | GSE1925\_CTRL\_VS\_IFNG\_PRIMED\_MACROPHAGE\_24H\_IFNG\_STIM\_DN | 172 | 11 | 12187 | 179 | Irf9,Icam1,Ifitm3,Isg15,Nfkb1,Ifit3b,Gbp3,Tnfsf10,Irgm2,Usp18,Ccnd2 | | 4.591e-05 | -9.99 | GSE7852\_THYMUS\_VS\_FAT\_TCONV\_DN | MSigDB lists | GSE7852\_THYMUS\_VS\_FAT\_TCONV\_DN | 172 | 11 | 12187 | 179 | Maff,Gadd45b,Xdh,Hspa5,Nfkbiz,Col4a1,Atf3,Ier3,Ifitm2,Tagln2,Plek | | 4.591e-05 | -9.99 | GSE29618\_BCELL\_VS\_MONOCYTE\_DN | MSigDB lists | GSE29618\_BCELL\_VS\_MONOCYTE\_DN | 172 | 11 | 12187 | 179 | Timp1,Cebpd,Trib1,Il17ra,Rbm47,Plek,Plaur,C5ar1,Ptgs2,Cxcl1,Cd14 | | 4.594e-05 | -9.99 | Nfkbiz (nuclear factor of kappa light polypeptide gene enhancer in B cells inhibitor, zeta) | protein interactions | 80859 | 5 | 3 | 6802 | 115 | Il6,Nfkb2,Nfkb1 | | 4.594e-05 | -9.99 | Tmem173 (transmembrane protein 173) | protein interactions | 72512 | 5 | 3 | 6802 | 115 | Trim30a,Usp18,Trim56 | | 4.659e-05 | -9.97 | GO\_HOMEOSTATIC\_PROCESS | MSigDB lists | GO\_HOMEOSTATIC\_PROCESS | 946 | 30 | 12187 | 179 | Kcna5,Cp,Ncf1,Saa1,Clic4,Hspb1,Icam1,S100a9,C5ar1,C3ar1,Sgk1,Lcn2,Il1a,Bbs12,Sgk3,Tap1,Tgm2,Ccl2,Spi1,Pik3r1,Tnfaip3,Ccl12,Il6,Nfe2l2,Cybb,Slc24a4,Hcar2,Angptl4,Pygm,S100a8 | | 4.674e-05 | -9.97 | GO\_REGULATION\_OF\_EPITHELIAL\_CELL\_APOPTOTIC\_PROCESS | MSigDB lists | GO\_REGULATION\_OF\_EPITHELIAL\_CELL\_APOPTOTIC\_PROCESS | 45 | 6 | 12187 | 179 | Thbs1,Tnfaip3,Nfe2l2,Il6,Angptl4,Icam1 | | 4.674e-05 | -9.97 | CHANG\_IMMORTALIZED\_BY\_HPV31\_DN | MSigDB lists | CHANG\_IMMORTALIZED\_BY\_HPV31\_DN | 45 | 6 | 12187 | 179 | Lcn2,Il1rn,Trib1,Ifit2,Ifi44,Ptgs2 | | 4.674e-05 | -9.97 | ZHOU\_TNF\_SIGNALING\_4HR | MSigDB lists | ZHOU\_TNF\_SIGNALING\_4HR | 45 | 6 | 12187 | 179 | Ptges,Tnip1,Nfkbia,Nfkb1,Ccl12,Clic4 | | 4.813e-05 | -9.94 | SERVITJA\_ISLET\_HNF1A\_TARGETS\_UP | MSigDB lists | SERVITJA\_ISLET\_HNF1A\_TARGETS\_UP | 143 | 10 | 12187 | 179 | Ifitm3,Msn,Gbp3,Ifit2,Tgm2,Vcam1,Akap12,Rhoc,Atf3,Ccl12 | | 4.813e-05 | -9.94 | GSE360\_HIGH\_DOSE\_B\_MALAYI\_VS\_M\_TUBERCULOSIS\_MAC\_DN | MSigDB lists | GSE360\_HIGH\_DOSE\_B\_MALAYI\_VS\_M\_TUBERCULOSIS\_MAC\_DN | 143 | 10 | 12187 | 179 | Zfp36,Rgs16,Pygm,Ptgs2,Cxcl1,Bcl3,Timp1,Tnfaip3,Runx1,Zfp189 | | 4.829e-05 | -9.94 | GO\_NEGATIVE\_REGULATION\_OF\_EXTRINSIC\_APOPTOTIC\_SIGNALING\_PATHWAY\_VIA\_DEATH\_DOMAIN\_RECEPTORS | MSigDB lists | GO\_NEGATIVE\_REGULATION\_OF\_EXTRINSIC\_APOPTOTIC\_SIGNALING\_PATHWAY\_VIA\_DEATH\_DOMAIN\_RECEPTORS | 28 | 5 | 12187 | 179 | Cflar,Icam1,Ripk1,Tnfaip3,Tnfsf10 | | 4.829e-05 | -9.94 | ZHAN\_MULTIPLE\_MYELOMA\_DN | MSigDB lists | ZHAN\_MULTIPLE\_MYELOMA\_DN | 28 | 5 | 12187 | 179 | Cebpd,Lcn2,Vcam1,S100a9,A2m | | 4.842e-05 | -9.94 | GSE37416\_CTRL\_VS\_24H\_F\_TULARENSIS\_LVS\_NEUTROPHIL\_DN | MSigDB lists | GSE37416\_CTRL\_VS\_24H\_F\_TULARENSIS\_LVS\_NEUTROPHIL\_DN | 173 | 11 | 12187 | 179 | Irak3,Icam1,Nfkbie,Bcl3,Cxcl1,Gadd45b,Nfe2l2,Tnfaip3,Birc3,Trib1,Nfkbia | | 4.848e-05 | -9.93 | MODULE\_118 | MSigDB lists | MODULE\_118 | 308 | 15 | 12187 | 179 | Ccl12,Ccl4,Tgm2,Maff,Ccnd2,Tnf,Rgs16,S100a8,Olfml2b,Ier3,Col4a1,Akap12,Thbs1,Cdkn1a,Isg15 | | 4.865e-05 | -9.93 | GO\_POSITIVE\_REGULATION\_OF\_KINASE\_ACTIVITY | MSigDB lists | GO\_POSITIVE\_REGULATION\_OF\_KINASE\_ACTIVITY | 383 | 17 | 12187 | 179 | Ripk1,Gadd45b,Sdc4,Tnf,Ccnd2,Map3k8,Iqgap1,Pik3r5,Fzd4,C5ar1,Gadd45g,Cdkn1a,Thbs1,Hspa5,Ptpn1,Saa1,Map3k6 | | 4.903e-05 | -9.92 | MODULE\_27 | MSigDB lists | MODULE\_27 | 238 | 13 | 12187 | 179 | Ccl2,Il4ra,Cdkn1a,Ccl12,Cd14,C3ar1,Selp,C5ar1,Csf2rb,Plaur,P2ry6,Igsf6,Icam1 | | 5.021e-05 | -9.90 | GO\_RNA\_POLYMERASE\_II\_TRANSCRIPTION\_FACTOR\_ACTIVITY\_SEQUENCE\_SPECIFIC\_DNA\_BINDING | MSigDB lists | GO\_RNA\_POLYMERASE\_II\_TRANSCRIPTION\_FACTOR\_ACTIVITY\_SEQUENCE\_SPECIFIC\_DNA\_BINDING | 423 | 18 | 12187 | 179 | Nfe2l2,Bach1,Spi1,Runx1,Maff,Nfkb2,Ifi211,Arid5b,Irf7,Atf3,Cebpd,Ifi204,Nfkb1,Ifi207,Zbtb16,Ifi209,Sox11,Irf2 | | 5.031e-05 | -9.90 | KUMAR\_TARGETS\_OF\_MLL\_AF9\_FUSION | MSigDB lists | KUMAR\_TARGETS\_OF\_MLL\_AF9\_FUSION | 309 | 15 | 12187 | 179 | Cmpk2,Irf7,Ifi211,Nfe2l2,Usp18,Sdc4,Ifit2,Irf9,Isg15,Ifi207,Ifi204,Il1rn,Ifit3b,Vcam1,Ifi209 | | 5.074e-05 | -9.89 | chemokine-mediated signaling pathway | biological process | GO:0070098 | 43 | 6 | 13711 | 214 | Ccl7,Ccl4,Ccl2,Ccl12,Cxcl1,Cxcl9 | | 5.103e-05 | -9.88 | HALLMARK\_P53\_PATHWAY | MSigDB lists | HALLMARK\_P53\_PATHWAY | 174 | 11 | 12187 | 179 | Zbtb16,Cdkn1a,Ier3,Atf3,Mxd4,Il1a,Tap1,Ip6k2,Ccnd2,Rrp8,Rgs16 | | 5.109e-05 | -9.88 | type I interferon signaling pathway | biological process | GO:0060337 | 14 | 4 | 13711 | 214 | Ifitm2,Ifitm3,Ifitm6,Irf7 | | 5.109e-05 | -9.88 | cellular response to type I interferon | biological process | GO:0071357 | 14 | 4 | 13711 | 214 | Ifitm3,Ifitm6,Irf7,Ifitm2 | | 5.136e-05 | -9.88 | negative regulation of immune response | biological process | GO:0050777 | 109 | 9 | 13711 | 214 | Il4ra,Acod1,Tnf,Tnfaip3,Tap1,A2m,Parp14,Irak3,Lgals9 | | 5.136e-05 | -9.88 | positive regulation of cytokine secretion | biological process | GO:0050715 | 109 | 9 | 13711 | 214 | Il4ra,Tnf,Casp4,Akap12,Il1a,Il17ra,Lgals9,Cd14,Mmp8 | | 5.177e-05 | -9.87 | ENK\_UV\_RESPONSE\_KERATINOCYTE\_UP | MSigDB lists | ENK\_UV\_RESPONSE\_KERATINOCYTE\_UP | 424 | 18 | 12187 | 179 | Cdkn1a,Sgk1,Ier3,Atf3,Stx11,Tagln2,Isg15,Hspb1,Zfp36,Tgm2,Sdc4,Trib1,Maff,Tnfaip3,Gadd45b,Cxcl1,Gem,Rgs16 | | 5.181e-05 | -9.87 | PYRIN | smart domains | SM01289 | 13 | 4 | 7188 | 123 | Ifi204,Ifi209,Ifi207,Ifi211 | | 5.196e-05 | -9.86 | FEVR\_CTNNB1\_TARGETS\_UP | MSigDB lists | FEVR\_CTNNB1\_TARGETS\_UP | 505 | 20 | 12187 | 179 | Plek,Trim25,Tap1,Cxcl16,Gadd45g,Hp,Atf3,Cdkn1a,Ifit3b,Socs3,Cmpk2,Xdh,Irf7,Angptl4,Oasl1,Gadd45b,Tnfaip3,Maff,Usp18,Casp4 | | 5.220e-05 | -9.86 | SMID\_BREAST\_CANCER\_NORMAL\_LIKE\_UP | MSigDB lists | SMID\_BREAST\_CANCER\_NORMAL\_LIKE\_UP | 310 | 15 | 12187 | 179 | Gem,Ifi211,Cxcl1,Tnfaip8,Birc3,Pik3r1,Il6,Ccl12,Fam107a,Sele,Ifi209,Vcam1,Ifi207,Ms4a6d,Ifi204 | | 5.310e-05 | -9.84 | TONKS\_TARGETS\_OF\_RUNX1\_RUNX1T1\_FUSION\_SUSTAINDED\_IN\_ERYTHROCYTE\_UP | MSigDB lists | TONKS\_TARGETS\_OF\_RUNX1\_RUNX1T1\_FUSION\_SUSTAINDED\_IN\_ERYTHROCYTE\_UP | 46 | 6 | 12187 | 179 | Ifi207,Hspb1,Ifi204,Il1rn,Ifi211,Ifi209 | | 5.392e-05 | -9.83 | GO\_NEGATIVE\_REGULATION\_OF\_INTRACELLULAR\_SIGNAL\_TRANSDUCTION | MSigDB lists | GO\_NEGATIVE\_REGULATION\_OF\_INTRACELLULAR\_SIGNAL\_TRANSDUCTION | 348 | 16 | 12187 | 179 | Tnip1,Plek,Irak3,Hspb1,Plaur,Ptpn1,Errfi1,Thbs1,Socs3,Tnip3,Atf3,Xdh,Nfkbia,Nfe2l2,Tnfaip3,Ripk1 | | 5.413e-05 | -9.82 | GO\_RESPONSE\_TO\_INTERFERON\_ALPHA | MSigDB lists | GO\_RESPONSE\_TO\_INTERFERON\_ALPHA | 15 | 4 | 12187 | 179 | Ifitm2,Ifitm3,Ifit2,Ifit3b | | 5.413e-05 | -9.82 | MAHADEVAN\_RESPONSE\_TO\_MP470\_DN | MSigDB lists | MAHADEVAN\_RESPONSE\_TO\_MP470\_DN | 15 | 4 | 12187 | 179 | Gem,Maff,Atf3,Gadd45b | | 5.417e-05 | -9.82 | GO\_MYELOID\_CELL\_DIFFERENTIATION | MSigDB lists | GO\_MYELOID\_CELL\_DIFFERENTIATION | 145 | 10 | 12187 | 179 | Sbno2,Ifi211,Csf3,Ifi204,Ifi207,Zbtb16,Spi1,Tnf,Runx1,Ifi209 | | 5.574e-05 | -9.79 | LABBE\_WNT3A\_TARGETS\_UP | MSigDB lists | LABBE\_WNT3A\_TARGETS\_UP | 91 | 8 | 12187 | 179 | Gadd45g,Akap12,Cebpd,Sgk1,Ifit3b,Ccl2,Tgm2,Nfkb2 | | 5.743e-05 | -9.77 | MEK\_UP.V1\_DN | MSigDB lists | MEK\_UP.V1\_DN | 146 | 10 | 12187 | 179 | Oasl1,Atf3,Ifi44,Usp18,S100a8,Tap1,Irf9,Herc6,Isg15,Irf7 | | 5.743e-05 | -9.77 | GSE32901\_TH17\_EMRICHED\_VS\_TH17\_NEG\_CD4\_TCELL\_DN | MSigDB lists | GSE32901\_TH17\_EMRICHED\_VS\_TH17\_NEG\_CD4\_TCELL\_DN | 146 | 10 | 12187 | 179 | Dnajb1,Nfkbiz,Nfkbia,Nfkb1,Tnfaip3,Rtp4,Ip6k2,Zfp36,Il17ra,Irf9 | | 5.743e-05 | -9.77 | GSE27859\_MACROPHAGE\_VS\_CD11C\_INT\_F480\_HI\_MACROPHAGE\_UP | MSigDB lists | GSE27859\_MACROPHAGE\_VS\_CD11C\_INT\_F480\_HI\_MACROPHAGE\_UP | 146 | 10 | 12187 | 179 | Map3k8,Tnf,Parp14,Nfkbia,Nfkb1,Gadd45b,Atf3,Nfkbie,Plek,Pik3ap1 | | 5.743e-05 | -9.77 | GSE18281\_MEDULLARY\_THYMOCYTE\_VS\_WHOLE\_MEDULLA\_THYMUS\_DN | MSigDB lists | GSE18281\_MEDULLARY\_THYMOCYTE\_VS\_WHOLE\_MEDULLA\_THYMUS\_DN | 146 | 10 | 12187 | 179 | Ifi209,Parp14,Ifi207,Ifi204,Oasl1,Znfx1,Herc6,Ifitm2,Ifi211,Rtp4 | | 5.767e-05 | -9.76 | SARTIPY\_NORMAL\_AT\_INSULIN\_RESISTANCE\_UP | MSigDB lists | SARTIPY\_NORMAL\_AT\_INSULIN\_RESISTANCE\_UP | 29 | 5 | 12187 | 179 | Ccl12,Tubb6,Socs3,Ptges,Thbs1 | | 5.767e-05 | -9.76 | GO\_POSITIVE\_REGULATION\_OF\_WOUND\_HEALING | MSigDB lists | GO\_POSITIVE\_REGULATION\_OF\_WOUND\_HEALING | 29 | 5 | 12187 | 179 | Nfe2l2,Plek,S100a9,Selp,Thbs1 | | 5.767e-05 | -9.76 | GNF2\_FOS | MSigDB lists | GNF2\_FOS | 29 | 5 | 12187 | 179 | Ms4a6d,Zfp36,Stx11,Cybb,Nfkbia | | 5.767e-05 | -9.76 | DORN\_ADENOVIRUS\_INFECTION\_32HR\_DN | MSigDB lists | DORN\_ADENOVIRUS\_INFECTION\_32HR\_DN | 29 | 5 | 12187 | 179 | Nfkbia,Sgk1,Nfkb2,Ier3,Nfe2l2 | | 5.784e-05 | -9.76 | Transcriptional misregulation in cancer | KEGG pathways | mmu05202 | 130 | 11 | 5248 | 107 | Cd14,Runx1,Zbtb16,Ccnd2,Spi1,Aff1,Cdkn1a,Nfkbiz,Birc3,Nfkb1,Il6 | | 5.784e-05 | -9.76 | Transcriptional misregulation in cancer | KEGG pathways | ko05202 | 130 | 11 | 5248 | 107 | Ccnd2,Birc3,Nfkb1,Spi1,Il6,Cd14,Aff1,Runx1,Cdkn1a,Zbtb16,Nfkbiz | | 5.838e-05 | -9.75 | RIG-I-like receptor signaling pathway | KEGG pathways | ko04622 | 50 | 7 | 5248 | 107 | Tnf,Isg15,Irf7,Ripk1,Trim25,Nfkbia,Nfkb1 | | 5.838e-05 | -9.75 | Legionellosis | KEGG pathways | mmu05134 | 50 | 7 | 5248 | 107 | Cd14,Cxcl1,Nfkb2,Tnf,Nfkb1,Nfkbia,Il6 | | 5.838e-05 | -9.75 | RIG-I-like receptor signaling pathway | KEGG pathways | mmu04622 | 50 | 7 | 5248 | 107 | Isg15,Irf7,Ripk1,Tnf,Nfkb1,Trim25,Nfkbia | | 5.838e-05 | -9.75 | Legionellosis | KEGG pathways | ko05134 | 50 | 7 | 5248 | 107 | Il6,Nfkbia,Nfkb1,Tnf,Cxcl1,Nfkb2,Cd14 | | 5.972e-05 | -9.73 | JOHNSTONE\_PARVB\_TARGETS\_3\_UP | MSigDB lists | JOHNSTONE\_PARVB\_TARGETS\_3\_UP | 351 | 16 | 12187 | 179 | Cebpd,Timp1,Thbs1,Errfi1,Rbm47,Icam1,Tnip1,Ifitm3,Parp12,Trib1,Nfkbia,Tgm2,Tnfsf10,Osmr,Iqgap1,Bcl3 | | 6.014e-05 | -9.72 | DIRMEIER\_LMP1\_RESPONSE\_LATE\_UP | MSigDB lists | DIRMEIER\_LMP1\_RESPONSE\_LATE\_UP | 47 | 6 | 12187 | 179 | Nfkb2,Plek,Nfkb1,Tnip1,Il4ra,Cflar | | 6.084e-05 | -9.71 | GSE22886\_IGG\_IGA\_MEMORY\_BCELL\_VS\_BM\_PLASMA\_CELL\_DN | MSigDB lists | GSE22886\_IGG\_IGA\_MEMORY\_BCELL\_VS\_BM\_PLASMA\_CELL\_DN | 147 | 10 | 12187 | 179 | Cflar,Cd14,Rbm47,Runx1,Nfkbia,Trib1,Socs3,Bach1,Timp1,Mxd4 | | 6.197e-05 | -9.69 | positive regulation of MAP kinase activity | biological process | GO:0043406 | 197 | 12 | 13711 | 214 | Ripk1,Thbs1,Il1rn,Gadd45g,Gadd45b,Tnf,Pik3r5,Ptpn1,Fzd4,Map3k6,Map3k8,Iqgap1 | | 6.223e-05 | -9.68 | positive regulation of vasculature development | biological process | GO:1904018 | 167 | 11 | 13711 | 214 | Cybb,Rhoj,C5ar1,Il1a,Runx1,Ptgs2,Nfe2l2,C3ar1,Thbs1,Hspb1,Cflar | | 6.233e-05 | -9.68 | positive regulation of protein kinase activity | biological process | GO:0045860 | 403 | 18 | 13711 | 214 | Sdc4,Iqgap1,Map3k6,Fzd4,Ptpn1,Map3k8,Irgm2,Pik3r5,Cdkn1a,Tnf,Lgals9,Gadd45b,Gadd45g,Ccnd2,Ripk1,Ncf1,Thbs1,Il1rn | | 6.275e-05 | -9.68 | GSE9006\_HEALTHY\_VS\_TYPE\_1\_DIABETES\_PBMC\_4MONTH\_POST\_DX\_UP | MSigDB lists | GSE9006\_HEALTHY\_VS\_TYPE\_1\_DIABETES\_PBMC\_4MONTH\_POST\_DX\_UP | 178 | 11 | 12187 | 179 | Cybb,Tnfaip3,Atf3,Gadd45b,Pik3r1,Maff,Tor3a,Nfkbia,Nfkb2,Msn,Nfkbie | | 6.275e-05 | -9.68 | RIGGINS\_TAMOXIFEN\_RESISTANCE\_DN | MSigDB lists | RIGGINS\_TAMOXIFEN\_RESISTANCE\_DN | 178 | 11 | 12187 | 179 | Isg15,Ifitm2,Ifitm3,Arid5b,S100a8,Rbm47,Tnfsf10,Irf9,Pik3r1,Timp1,Cebpd | | 6.398e-05 | -9.66 | response to toxic substance | biological process | GO:0009636 | 262 | 14 | 13711 | 214 | Lcn2,Ripk1,Ccl7,Il6,Tnf,Tnfaip3,Mt1,Nfe2l2,Ccl4,S100a9,S100a8,Mt2,Hp,Nfkb1 | | 6.444e-05 | -9.65 | GSE29618\_MONOCYTE\_VS\_MDC\_UP | MSigDB lists | GSE29618\_MONOCYTE\_VS\_MDC\_UP | 148 | 10 | 12187 | 179 | Nfkbia,Bach1,Cybb,Ier3,C5ar1,Cd14,S100a9,Cxcl1,S100a8,Irak3 | | 6.444e-05 | -9.65 | STTTCRNTTT\_IRF\_Q6 | MSigDB lists | STTTCRNTTT\_IRF\_Q6 | 148 | 10 | 12187 | 179 | Col4a1,Ifi44,Ifit2,Ifit3b,Usp18,Xaf1,Tap1,Dtx3l,Isg15,Ncf1 | | 6.444e-05 | -9.65 | GSE17721\_PAM3CSK4\_VS\_CPG\_2H\_BMDC\_DN | MSigDB lists | GSE17721\_PAM3CSK4\_VS\_CPG\_2H\_BMDC\_DN | 148 | 10 | 12187 | 179 | Irf9,Icam1,Ccl12,Oasl1,Il1a,Ccl4,Ccl2,Tnf,Ifit2,Maff | | 6.741e-05 | -9.60 | regulation of localization | biological process | GO:0032879 | 2332 | 59 | 13711 | 214 | Gbp4,Adamts1,Rhoj,Kcna5,Il17ra,Sox11,Icam1,Cybb,Msr1,Thbs1,Lgals9,P2ry6,Ptpn1,Ccl12,Cxcl16,Iqgap1,S100a8,S100a9,Tnfaip3,Ccl2,Adamts9,Il1rn,C3ar1,Fam107a,Timp1,Sdc4,Parp9,Ccl4,Ier3,Sgk1,Ptgs2,Runx1,Gem,Il1a,Akap12,Nfkbia,Casp4,Il4ra,Il6,Trib1,Dtx3l,Pik3r1,Hcar2,Clic4,Hspa5,C5ar1,Cxcl1,Mmp8,Nfe2l2,Selp,Msn,Robo4,Tnf,Zfp36,Cd14,Ripk1,Sele,Hspb1,Rhoc | | 6.790e-05 | -9.60 | GNF2\_CARD15 | MSigDB lists | GNF2\_CARD15 | 48 | 6 | 12187 | 179 | Ms4a6d,Stx11,Cybb,S100a9,Cd14,Igsf6 | | 6.790e-05 | -9.60 | CROONQUIST\_STROMAL\_STIMULATION\_UP | MSigDB lists | CROONQUIST\_STROMAL\_STIMULATION\_UP | 48 | 6 | 12187 | 179 | Cxcl1,Ccl4,Tgm2,Nfkbia,Il6,Ier3 | | 6.790e-05 | -9.60 | PID\_CERAMIDE\_PATHWAY | MSigDB lists | PID\_CERAMIDE\_PATHWAY | 48 | 6 | 12187 | 179 | Ksr1,Nfkb1,Ripk1,Tnf,Birc3,Nfkbia | | 6.804e-05 | -9.60 | Hemostasis | REACTOME pathways | R-MMU-109582 | 391 | 18 | 6297 | 105 | Ptpn1,Irf7,Maff,Serpina3f,Timp1,Sdc4,Sele,Csf2rb,Pik3r1,Pik3r5,Irf2,Tagln2,Thbs1,Plaur,Plek,A2m,Hspa5,Selp | | 6.810e-05 | -9.59 | Tnfrsf1a (tumor necrosis factor receptor superfamily, member 1a) | protein interactions | 21937 | 14 | 4 | 6802 | 115 | Tnfaip3,Ripk1,Tnf,Tnip1 | | 6.821e-05 | -9.59 | GSE34156\_UNTREATED\_VS\_6H\_TLR1\_TLR2\_LIGAND\_TREATED\_MONOCYTE\_UP | MSigDB lists | GSE34156\_UNTREATED\_VS\_6H\_TLR1\_TLR2\_LIGAND\_TREATED\_MONOCYTE\_UP | 149 | 10 | 12187 | 179 | Cd14,Rbm47,Igsf6,Clic4,Csf2rb,Cxcl16,Msr1,Rhou,Cybb,AA467197 | | 6.821e-05 | -9.59 | GSE34179\_THPOK\_KO\_VS\_WT\_VA14I\_NKTCELL\_DN | MSigDB lists | GSE34179\_THPOK\_KO\_VS\_WT\_VA14I\_NKTCELL\_DN | 149 | 10 | 12187 | 179 | Trib1,Ccl4,Ccnd2,Rasip1,Gadd45b,Hcar2,Il1rn,Csf3,Clic4,Plek | | 6.821e-05 | -9.59 | GSE41867\_NAIVE\_VS\_DAY6\_LCMV\_ARMSTRONG\_EFFECTOR\_CD8\_TCELL\_DN | MSigDB lists | GSE41867\_NAIVE\_VS\_DAY6\_LCMV\_ARMSTRONG\_EFFECTOR\_CD8\_TCELL\_DN | 149 | 10 | 12187 | 179 | S100a8,Tnip1,Irf7,Stx11,Tnfaip3,Il6,Ier3,Atf3,Tnf,Nfkbia | | 6.838e-05 | -9.59 | DORN\_ADENOVIRUS\_INFECTION\_48HR\_DN | MSigDB lists | DORN\_ADENOVIRUS\_INFECTION\_48HR\_DN | 30 | 5 | 12187 | 179 | Il6,Nfe2l2,Ier3,Sgk1,Nfkbia | | 6.838e-05 | -9.59 | VALK\_AML\_WITH\_CEBPA | MSigDB lists | VALK\_AML\_WITH\_CEBPA | 30 | 5 | 12187 | 179 | Trib1,Mmp8,Tubb6,Hspb1,Lcn2 | | 6.838e-05 | -9.59 | GO\_REGULATION\_OF\_ENDOTHELIAL\_CELL\_APOPTOTIC\_PROCESS | MSigDB lists | GO\_REGULATION\_OF\_ENDOTHELIAL\_CELL\_APOPTOTIC\_PROCESS | 30 | 5 | 12187 | 179 | Thbs1,Angptl4,Icam1,Tnfaip3,Nfe2l2 | | 6.875e-05 | -9.59 | Apoptosis signaling pathway | Lipid Maps pathways | Apoptosis | 71 | 14 | 354 | 26 | Irak3,Pik3r1,Nfkbia,Tnf,Il1a,Cflar,Csf2rb,Birc3,Nfkbie,Nfkb1,Ripk1,Nfkb2,Tnfsf10,Pik3r5 | | 6.881e-05 | -9.58 | leukocyte tethering or rolling | biological process | GO:0050901 | 15 | 4 | 13711 | 214 | Selp,Sele,Vcam1,Tnf | | 6.932e-05 | -9.58 | regulation of myeloid cell differentiation | biological process | GO:0045637 | 169 | 11 | 13711 | 214 | Nfkbia,Tnf,Isg15,Zfp36,Csf3,Ripk1,Zbtb16,Trib1,Spi1,Runx1,Pik3r1 | | 7.033e-05 | -9.56 | MIKKELSEN\_MEF\_LCP\_WITH\_H3K4ME3 | MSigDB lists | MIKKELSEN\_MEF\_LCP\_WITH\_H3K4ME3 | 94 | 8 | 12187 | 179 | Xaf1,Casp4,Il1rn,Cp,Ccl12,Slfn4,Slfn3,Xdh | | 7.080e-05 | -9.56 | negative regulation of cell communication | biological process | GO:0010648 | 1086 | 34 | 13711 | 214 | Trim30a,Rnf213,Tnip1,Cflar,Ripk1,Hspb1,Irak3,Il1rn,Atf3,Xdh,Pik3ap1,Plek,Acod1,Tnfaip3,Tnf,Bcl3,Sgk3,Nfe2l2,Tnip3,Ptpn1,Hspa5,Trib1,Parp14,Socs3,Thbs1,Il6,Errfi1,Rgs16,Nfkbia,Icam1,Ier3,Plaur,Ptgs2,Rasip1 | | 7.086e-05 | -9.55 | negative regulation of protein phosphorylation | biological process | GO:0001933 | 370 | 17 | 13711 | 214 | Irak3,Hspb1,Socs3,Trib1,Parp14,Gadd45b,Tnip1,Gadd45g,Tnfaip3,Xdh,Errfi1,Atf3,Il6,Cdkn1a,Rasip1,Gbp4,Ptpn1 | | 7.094e-05 | -9.55 | KEGG\_PATHWAYS\_IN\_CANCER | MSigDB lists | KEGG\_PATHWAYS\_IN\_CANCER | 282 | 14 | 12187 | 179 | Ptgs2,Cdkn1a,Zbtb16,Nfkb1,Col4a1,Pik3r5,Fzd4,Nfkb2,Runx1,Birc3,Pik3r1,Nfkbia,Spi1,Il6 | | 7.134e-05 | -9.55 | BIOCARTA\_TNFR2\_PATHWAY | MSigDB lists | BIOCARTA\_TNFR2\_PATHWAY | 16 | 4 | 12187 | 179 | Nfkbia,Ripk1,Tnfaip3,Nfkb1 | | 7.134e-05 | -9.55 | PID\_NFKAPPAB\_ATYPICAL\_PATHWAY | MSigDB lists | PID\_NFKAPPAB\_ATYPICAL\_PATHWAY | 16 | 4 | 12187 | 179 | Pik3r1,Nfkbia,Bcl3,Nfkb1 | | 7.134e-05 | -9.55 | BIOCARTA\_RELA\_PATHWAY | MSigDB lists | BIOCARTA\_RELA\_PATHWAY | 16 | 4 | 12187 | 179 | Nfkbia,Tnf,Ripk1,Nfkb1 | | 7.134e-05 | -9.55 | GO\_RETINA\_VASCULATURE\_DEVELOPMENT\_IN\_CAMERA\_TYPE\_EYE | MSigDB lists | GO\_RETINA\_VASCULATURE\_DEVELOPMENT\_IN\_CAMERA\_TYPE\_EYE | 16 | 4 | 12187 | 179 | Fzd4,Rhoj,Clic4,Col4a1 | | 7.134e-05 | -9.55 | REACTOME\_RIP\_MEDIATED\_NFKB\_ACTIVATION\_VIA\_DAI | MSigDB lists | REACTOME\_RIP\_MEDIATED\_NFKB\_ACTIVATION\_VIA\_DAI | 16 | 4 | 12187 | 179 | Ripk1,Nfkb2,Saa1,Nfkbia | | 7.148e-05 | -9.55 | GO\_LEUKOCYTE\_DIFFERENTIATION | MSigDB lists | GO\_LEUKOCYTE\_DIFFERENTIATION | 213 | 12 | 12187 | 179 | Sbno2,Csf3,Bcl3,Ifi211,Ifi207,Ifi204,Il6,Pik3r1,Ifi209,Tnf,Spi1,Vcam1 | | 7.150e-05 | -9.55 | DAPIN | prosite domains | PS50824 | 13 | 4 | 8845 | 164 | Ifi204,Ifi211,Ifi209,Ifi207 | | 7.167e-05 | -9.54 | WNT\_UP.V1\_DN | MSigDB lists | WNT\_UP.V1\_DN | 121 | 9 | 12187 | 179 | Irf9,Csf2rb,Gbp3,Cmpk2,Irf7,Isg15,Ptgs2,Rasd1,Usp18 | | 7.169e-05 | -9.54 | negative regulation of apoptotic signaling pathway | biological process | GO:2001234 | 200 | 12 | 13711 | 214 | Ptgs2,Plaur,Ier3,Nfe2l2,Sgk3,Ptpn1,Hspb1,Ripk1,Cflar,Tnf,Tnfaip3,Icam1 | | 7.171e-05 | -9.54 | RUTELLA\_RESPONSE\_TO\_CSF2RB\_AND\_IL4\_DN | MSigDB lists | RUTELLA\_RESPONSE\_TO\_CSF2RB\_AND\_IL4\_DN | 247 | 13 | 12187 | 179 | S100a9,C3ar1,C5ar1,Ccl12,Cybb,Zfp36,Irak3,Cd14,Ifitm2,Cebpd,Ms4a6d,Gadd45g,Sgk1 | | 7.209e-05 | -9.54 | GO\_RESPONSE\_TO\_HEAT | MSigDB lists | GO\_RESPONSE\_TO\_HEAT | 70 | 7 | 12187 | 179 | Cd14,Socs3,Thbs1,Cdkn1a,Il6,Ccl12,Il1a | | 7.209e-05 | -9.54 | GO\_ENHANCER\_BINDING | MSigDB lists | GO\_ENHANCER\_BINDING | 70 | 7 | 12187 | 179 | Bach1,Tiparp,Nfkb1,Nfe2l2,Sox11,Spi1,Zbtb16 | | 7.216e-05 | -9.54 | GSE36078\_WT\_VS\_IL1R\_KO\_LUNG\_DC\_AFTER\_AD5\_INF\_UP | MSigDB lists | GSE36078\_WT\_VS\_IL1R\_KO\_LUNG\_DC\_AFTER\_AD5\_INF\_UP | 150 | 10 | 12187 | 179 | Sgk1,Cdkn1a,Ier3,AA467197,Ifitm2,Ifitm3,Rhoj,Pik3ap1,Plek,Rbm47 | | 7.381e-05 | -9.51 | enhancer binding | molecular function | GO:0035326 | 89 | 8 | 13516 | 211 | Tiparp,Bach1,Nfkb1,Irf7,Sox11,Zbtb16,Spi1,Nfe2l2 | | 7.585e-05 | -9.49 | WANG\_ESOPHAGUS\_CANCER\_VS\_NORMAL\_UP | MSigDB lists | WANG\_ESOPHAGUS\_CANCER\_VS\_NORMAL\_UP | 95 | 8 | 12187 | 179 | Ccl4,Parp14,Socs3,Sdc4,Tnfaip3,Atf3,Ifitm3,Csf2rb | | 7.612e-05 | -9.48 | negative regulation of signaling | biological process | GO:0023057 | 1090 | 34 | 13711 | 214 | Plek,Pik3ap1,Acod1,Tnfaip3,Tnf,Xdh,Atf3,Ripk1,Cflar,Irak3,Il1rn,Hspb1,Rnf213,Trim30a,Tnip1,Hspa5,Ptpn1,Tnip3,Sgk3,Nfe2l2,Bcl3,Nfkbia,Rgs16,Icam1,Errfi1,Il6,Trib1,Parp14,Thbs1,Socs3,Rasip1,Plaur,Ier3,Ptgs2 | | 7.631e-05 | -9.48 | GSE2826\_WT\_VS\_BTK\_KO\_BCELL\_DN | MSigDB lists | GSE2826\_WT\_VS\_BTK\_KO\_BCELL\_DN | 151 | 10 | 12187 | 179 | Znfx1,Ccl12,Hp,Il1a,Vcam1,Thbs1,S100a8,Cd14,S100a9,Gbp7 | | 7.631e-05 | -9.48 | GSE360\_L\_MAJOR\_VS\_B\_MALAYI\_LOW\_DOSE\_DC\_UP | MSigDB lists | GSE360\_L\_MAJOR\_VS\_B\_MALAYI\_LOW\_DOSE\_DC\_UP | 151 | 10 | 12187 | 179 | Gadd45g,Nfkb1,Il6,Ier3,Tnfaip3,Dnajb1,Icam1,Tap1,Ifitm3,Tnip1 | | 7.631e-05 | -9.48 | GSE43955\_1H\_VS\_60H\_ACT\_CD4\_TCELL\_DN | MSigDB lists | GSE43955\_1H\_VS\_60H\_ACT\_CD4\_TCELL\_DN | 151 | 10 | 12187 | 179 | A2m,Il17ra,Errfi1,Il4ra,Map3k8,Casp4,Bach1,Il1rn,Gadd45b,Ccl12 | | 7.644e-05 | -9.48 | KANG\_GIST\_WITH\_PDGFRA\_UP | MSigDB lists | KANG\_GIST\_WITH\_PDGFRA\_UP | 49 | 6 | 12187 | 179 | Ifi204,Ifi207,Ifi209,Isg15,Ifi211,Usp18 | | 7.644e-05 | -9.48 | KEGG\_HEMATOPOIETIC\_CELL\_LINEAGE | MSigDB lists | KEGG\_HEMATOPOIETIC\_CELL\_LINEAGE | 49 | 6 | 12187 | 179 | Cd14,Csf3,Il4ra,Tnf,Il6,Il1a | | 7.644e-05 | -9.48 | chr1q22 | MSigDB lists | chr1q22 | 49 | 6 | 12187 | 179 | Selp,Ifi209,Ifi211,Sele,Ifi207,Ifi204 | | 7.671e-05 | -9.48 | GSE26488\_WT\_VS\_HDAC7\_DELTAP\_TG\_OT2\_THYMOCYTE\_WITH\_PEPTIDE\_INJECTION\_DN | MSigDB lists | GSE26488\_WT\_VS\_HDAC7\_DELTAP\_TG\_OT2\_THYMOCYTE\_WITH\_PEPTIDE\_INJECTION\_DN | 182 | 11 | 12187 | 179 | S100a9,Isg15,Fstl1,Socs3,Ifit3b,Sdc4,Thbs1,Ier3,Tiparp,Tubb6,Cp | | 7.735e-05 | -9.47 | negative regulation of toll-like receptor signaling pathway | biological process | GO:0034122 | 29 | 5 | 13711 | 214 | Trim30a,Pik3ap1,Irak3,Acod1,Tnfaip3 | | 8.057e-05 | -9.43 | PLASARI\_TGFB1\_TARGETS\_1HR\_UP | MSigDB lists | PLASARI\_TGFB1\_TARGETS\_1HR\_UP | 31 | 5 | 12187 | 179 | Ptgs2,Gadd45g,Il6,Ier3,Gadd45b | | 8.057e-05 | -9.43 | WEINMANN\_ADAPTATION\_TO\_HYPOXIA\_DN | MSigDB lists | WEINMANN\_ADAPTATION\_TO\_HYPOXIA\_DN | 31 | 5 | 12187 | 179 | Il1a,Plaur,Il6,Ier3,Sdc4 | | 8.066e-05 | -9.43 | negative regulation of multicellular organismal process | biological process | GO:0051241 | 1047 | 33 | 13711 | 214 | Noct,Bcl3,Mmp8,Nfe2l2,Prg4,Trim30a,Ripk1,Adamts9,Zbtb16,Ncf1,Irak3,Ccl2,Zfp36,Xdh,Tnf,Acod1,Tnfaip3,Ptgs2,Runx1,Adamts1,Nfkb1,Timp1,Gbp4,Lgals9,Pik3r1,Trib1,Thbs1,Errfi1,Il6,Il1a,Sox11,Nfkbia,Il4ra | | 8.066e-05 | -9.43 | GSE17721\_CTRL\_VS\_GARDIQUIMOD\_1H\_BMDC\_DN | MSigDB lists | GSE17721\_CTRL\_VS\_GARDIQUIMOD\_1H\_BMDC\_DN | 152 | 10 | 12187 | 179 | Map3k8,Maff,Nfkbia,Ccl2,Il1rn,Hcar2,Tagln2,Arid5b,Errfi1,Gbp3 | | 8.125e-05 | -9.42 | positive regulation of ERK1 and ERK2 cascade | biological process | GO:0070374 | 172 | 11 | 13711 | 214 | Ccl4,C5ar1,Ccl12,Cflar,Ccl7,P2ry6,Il1a,Akap12,Icam1,Ccl2,Il6 | | 8.142e-05 | -9.42 | GO\_REGULATION\_OF\_EXTRINSIC\_APOPTOTIC\_SIGNALING\_PATHWAY | MSigDB lists | GO\_REGULATION\_OF\_EXTRINSIC\_APOPTOTIC\_SIGNALING\_PATHWAY | 123 | 9 | 12187 | 179 | Ripk1,Tnfaip3,Atf3,Il1a,Tnf,Thbs1,Tnfsf10,Icam1,Cflar | | 8.253e-05 | -9.40 | negative regulation of transferase activity | biological process | GO:0051348 | 235 | 13 | 13711 | 214 | Rasip1,Ptpn1,Trib1,Hspb1,Dtx3l,Irak3,Gadd45g,Gadd45b,Tnfaip3,Cdkn1a,Il6,Zfp36,Errfi1 | | 8.266e-05 | -9.40 | regulation of cell activation | biological process | GO:0050865 | 412 | 18 | 13711 | 214 | Plek,Sox11,Il4ra,Tnfaip3,Cdkn1a,Ccl2,Il6,Zbtb16,Ctla2a,Thbs1,Lgals9,C5ar1,Nfkbiz,Sdc4,Mmp8,Vcam1,Runx1,Selp | | 8.464e-05 | -9.38 | RUTELLA\_RESPONSE\_TO\_HGF\_DN | MSigDB lists | RUTELLA\_RESPONSE\_TO\_HGF\_DN | 184 | 11 | 12187 | 179 | Ms4a6d,Gadd45b,Cebpd,Cdkn1a,Zfp36,Irak3,Ifitm2,Map3k6,C5ar1,S100a9,Cd14 | | 8.522e-05 | -9.37 | KRAS.600.LUNG.BREAST\_UP.V1\_UP | MSigDB lists | KRAS.600.LUNG.BREAST\_UP.V1\_UP | 153 | 10 | 12187 | 179 | Birc3,Tnfaip3,Akap12,Kcna5,A2m,Cxcl1,Angptl4,Csf3,Ptgs2,Plaur | | 8.522e-05 | -9.37 | GSE41867\_DAY6\_VS\_DAY8\_LCMV\_CLONE13\_EFFECTOR\_CD8\_TCELL\_UP | MSigDB lists | GSE41867\_DAY6\_VS\_DAY8\_LCMV\_CLONE13\_EFFECTOR\_CD8\_TCELL\_UP | 153 | 10 | 12187 | 179 | Lgals9,Ccl4,Stx11,Rhoc,Ms4a6d,Ifitm3,Rnf213,Tnip1,Slfn4,Slfn3 | | 8.522e-05 | -9.37 | BERTUCCI\_MEDULLARY\_VS\_DUCTAL\_BREAST\_CANCER\_UP | MSigDB lists | BERTUCCI\_MEDULLARY\_VS\_DUCTAL\_BREAST\_CANCER\_UP | 153 | 10 | 12187 | 179 | Icam1,Tap1,Gbp3,Nfkb2,Dtx3l,Nfkbie,Cebpd,Birc3,Socs3,Vcam1 | | 8.522e-05 | -9.37 | WIERENGA\_STAT5A\_TARGETS\_DN | MSigDB lists | WIERENGA\_STAT5A\_TARGETS\_DN | 153 | 10 | 12187 | 179 | Plek,Il17ra,Cd14,Ifi211,Selp,Lcn2,Ifi207,Stx11,Ifi204,Ifi209 | | 8.553e-05 | -9.37 | Poly(ADP-ribose)pol\_cat\_dom | interpro domains | IPR012317 | 16 | 4 | 13788 | 212 | Parp9,Parp12,Parp14,Tiparp | | 8.582e-05 | -9.36 | LIAN\_LIPA\_TARGETS\_6M | MSigDB lists | LIAN\_LIPA\_TARGETS\_6M | 50 | 6 | 12187 | 179 | Mmp8,Msr1,Igsf6,C3ar1,S100a9,Cxcl1 | | 8.670e-05 | -9.35 | GO\_POSITIVE\_REGULATION\_OF\_HEMOPOIESIS | MSigDB lists | GO\_POSITIVE\_REGULATION\_OF\_HEMOPOIESIS | 124 | 9 | 12187 | 179 | Csf3,Isg15,Trib1,Zbtb16,Il4ra,Runx1,Tnf,Il6,Ripk1 | | 8.954e-05 | -9.32 | GO\_POSITIVE\_REGULATION\_OF\_TRANSPORT | MSigDB lists | GO\_POSITIVE\_REGULATION\_OF\_TRANSPORT | 700 | 24 | 12187 | 179 | Msn,Sele,Ptgs2,Saa1,Nfkbia,Sdc4,Tnf,S100a9,Il1a,Sgk1,Sox11,Il17ra,Cd14,Sgk3,Il6,Nfe2l2,Ccl12,Hcar2,Ccl4,Pik3r1,Il4ra,Rhou,S100a8,Csf3 | | 9.000e-05 | -9.32 | GSE25085\_FETAL\_LIVER\_VS\_FETAL\_BM\_SP4\_THYMIC\_IMPLANT\_DN | MSigDB lists | GSE25085\_FETAL\_LIVER\_VS\_FETAL\_BM\_SP4\_THYMIC\_IMPLANT\_DN | 154 | 10 | 12187 | 179 | Gbp4,Mmp8,Tubb6,Hp,Parp14,Msr1,Xdh,Trim25,Ifitm3,Gbp6 | | 9.063e-05 | -9.31 | leukocyte adhesion to vascular endothelial cell | biological process | GO:0061756 | 16 | 4 | 13711 | 214 | Sele,Vcam1,Tnf,Selp | | 9.063e-05 | -9.31 | T cell migration | biological process | GO:0072678 | 16 | 4 | 13711 | 214 | Msn,Ccl2,Cxcl16,Icam1 | | 9.211e-05 | -9.29 | GO\_CELLULAR\_RESPONSE\_TO\_STRESS | MSigDB lists | GO\_CELLULAR\_RESPONSE\_TO\_STRESS | 1280 | 36 | 12187 | 179 | Trib1,Casp4,Nfkbia,Zfp189,Tnf,Bach1,Irf7,Ifi209,Cdkn1a,Atf3,Ifi207,Ifi204,Ptgs2,Errfi1,Icam1,Trim25,Pik3r1,Map3k8,Nfe2l2,Il6,Ccl12,Tnfaip3,Ripk1,Bcl3,Rrp8,Ifi211,Dtx3l,Hspa5,Sgk1,Thbs1,Il1a,Nfkb1,Map3k6,Isg15,Ptpn1,Parp9 | | 9.223e-05 | -9.29 | GO\_LYMPHOCYTE\_CHEMOTAXIS | MSigDB lists | GO\_LYMPHOCYTE\_CHEMOTAXIS | 17 | 4 | 12187 | 179 | Cxcl16,Ccl12,Saa1,Ccl2 | | 9.223e-05 | -9.29 | GO\_TRANSCRIPTIONAL\_ACTIVATOR\_ACTIVITY\_RNA\_POLYMERASE\_II\_DISTAL\_ENHANCER\_SEQUENCE\_SPECIFIC\_BINDING | MSigDB lists | GO\_TRANSCRIPTIONAL\_ACTIVATOR\_ACTIVITY\_RNA\_POLYMERASE\_II\_DISTAL\_ENHANCER\_SEQUENCE\_SPECIFIC\_BINDING | 17 | 4 | 12187 | 179 | Spi1,Nfkb1,Bach1,Nfe2l2 | | 9.223e-05 | -9.29 | DAZARD\_UV\_RESPONSE\_CLUSTER\_G2 | MSigDB lists | DAZARD\_UV\_RESPONSE\_CLUSTER\_G2 | 17 | 4 | 12187 | 179 | Sgk1,Cxcl1,Atf3,Ier3 | | 9.326e-05 | -9.28 | DAVICIONI\_TARGETS\_OF\_PAX\_FOXO1\_FUSIONS\_UP | MSigDB lists | DAVICIONI\_TARGETS\_OF\_PAX\_FOXO1\_FUSIONS\_UP | 219 | 12 | 12187 | 179 | Tnfaip3,Sdc4,Il4ra,Maff,Ccnd2,Arid5b,Akap12,Timp1,Thbs1,Sgk1,Adamts1,Msn | | 9.383e-05 | -9.27 | negative regulation of proteolysis | biological process | GO:0045861 | 238 | 13 | 13711 | 214 | Il6,Cflar,Ctla2a,Tnfaip8,Thbs1,Pik3r1,Birc3,Timp1,A2m,Serpina3f,Wfdc21,Plaur,Ptgs2 | | 9.436e-05 | -9.27 | GNF2\_CD1D | MSigDB lists | GNF2\_CD1D | 32 | 5 | 12187 | 179 | Ms4a6d,Cybb,Igsf6,Stx11,Cd14 | | 9.436e-05 | -9.27 | SIG\_CD40PATHWAYMAP | MSigDB lists | SIG\_CD40PATHWAYMAP | 32 | 5 | 12187 | 179 | Nfkb1,Nfkb2,Nfkbie,Pik3r1,Nfkbia | | 9.436e-05 | -9.27 | PID\_IL1\_PATHWAY | MSigDB lists | PID\_IL1\_PATHWAY | 32 | 5 | 12187 | 179 | Irak3,Il1rn,Nfkb1,Il1a,Pik3r1 | | 9.443e-05 | -9.27 | SASSON\_RESPONSE\_TO\_GONADOTROPHINS\_DN | MSigDB lists | SASSON\_RESPONSE\_TO\_GONADOTROPHINS\_DN | 73 | 7 | 12187 | 179 | Akap12,Thbs1,Casp4,Trib1,Ptpn1,Cflar,Gem | | 9.500e-05 | -9.26 | GSE21063\_CTRL\_VS\_ANTI\_IGM\_STIM\_BCELL\_NFATC1\_KO\_16H\_UP | MSigDB lists | GSE21063\_CTRL\_VS\_ANTI\_IGM\_STIM\_BCELL\_NFATC1\_KO\_16H\_UP | 155 | 10 | 12187 | 179 | Casp4,Ccl4,Samd9l,Timp1,Ccl12,Ptges,Slfn3,Slfn4,Parp9,Xdh | | 9.500e-05 | -9.26 | GSE339\_EX\_VIVO\_VS\_IN\_CULTURE\_CD4POS\_DC\_DN | MSigDB lists | GSE339\_EX\_VIVO\_VS\_IN\_CULTURE\_CD4POS\_DC\_DN | 155 | 10 | 12187 | 179 | Il1rn,Tubb6,Rasd1,Cdkn1a,Ifit2,Pik3r1,Casp4,Irgm2,Cflar,Ptges | | 9.520e-05 | -9.26 | vasculature development | biological process | GO:0001944 | 455 | 19 | 13711 | 214 | Ccl2,Errfi1,Cemip2,Robo4,Rnf213,Adamts9,Thbs1,Socs3,Ccl12,Col4a1,Rhoj,Apold1,Fzd4,Clic4,Tiparp,Angptl4,Ptgs2,Spi1,Rasip1 | | 9.716e-05 | -9.24 | regulation of interleukin-1 production | biological process | GO:0032652 | 69 | 7 | 13711 | 214 | Ifi204,Lgals9,Ifi211,Hspb1,Errfi1,Tnfaip3,Casp4 | | 9.767e-05 | -9.23 | negative regulation of protein modification process | biological process | GO:0031400 | 535 | 21 | 13711 | 214 | Rasip1,Spi1,Isg15,Gbp4,Ptpn1,Tnip1,Gadd45g,Gadd45b,Hspb1,Socs3,Dtx3l,Irak3,Trib1,Parp14,Il6,Errfi1,Atf3,Xdh,Cdkn1a,Tnfaip3,Tnf | | 9.786e-05 | -9.23 | cell motility | biological process | GO:0048870 | 744 | 26 | 13711 | 214 | Ccl4,Il17ra,Sdc4,Ccl7,Thbs1,Icam1,S100a9,Vcam1,Msn,Selp,Cxcl1,C5ar1,Cxcl16,Ccl12,Iqgap1,S100a8,Il1rn,Arid5b,Sele,Retnlg,Rhoc,Cxcl9,Tnf,Tnfaip3,Ccl2,Saa1 | | 9.786e-05 | -9.23 | localization of cell | biological process | GO:0051674 | 744 | 26 | 13711 | 214 | S100a9,Vcam1,Msn,Selp,Cxcl1,C5ar1,Cxcl16,Ccl12,S100a8,Iqgap1,Arid5b,Il1rn,Sele,Retnlg,Rhoc,Cxcl9,Tnf,Tnfaip3,Ccl2,Saa1,Ccl4,Il17ra,Sdc4,Ccl7,Thbs1,Icam1 | | 9.812e-05 | -9.23 | GROSS\_HYPOXIA\_VIA\_ELK3\_AND\_HIF1A\_UP | MSigDB lists | GROSS\_HYPOXIA\_VIA\_ELK3\_AND\_HIF1A\_UP | 126 | 9 | 12187 | 179 | Sdc4,Nfkbia,Nfkbiz,Sgk1,Atf3,Angptl4,Rgs16,Errfi1,Zfp36 | | 9.979e-05 | -9.21 | GO\_RESPONSE\_TO\_WOUNDING | MSigDB lists | GO\_RESPONSE\_TO\_WOUNDING | 406 | 17 | 12187 | 179 | S100a8,Cflar,Pik3r5,A2m,Rhoc,Ccl12,Il6,Pik3r1,Maff,Sdc4,Hspb1,Plek,Irf2,Plaur,Saa1,Timp1,Il1a | | 1.002e-04 | -9.21 | GSE17721\_PAM3CSK4\_VS\_CPG\_4H\_BMDC\_DN | MSigDB lists | GSE17721\_PAM3CSK4\_VS\_CPG\_4H\_BMDC\_DN | 156 | 10 | 12187 | 179 | Tor3a,Ccl2,Ms4a6d,Rasd1,Ptgs2,Isg15,Gbp3,Clic4,Irf2,Irf9 | | 1.002e-04 | -9.21 | GSE23925\_LIGHT\_ZONE\_VS\_NAIVE\_BCELL\_UP | MSigDB lists | GSE23925\_LIGHT\_ZONE\_VS\_NAIVE\_BCELL\_UP | 156 | 10 | 12187 | 179 | Map3k8,Maff,Ccl4,Nfkbiz,Trib1,Tnfaip3,Atf3,Gem,Zfp36,Sbno2 | | 1.008e-04 | -9.20 | GO\_RESPONSE\_TO\_ABIOTIC\_STIMULUS | MSigDB lists | GO\_RESPONSE\_TO\_ABIOTIC\_STIMULUS | 797 | 26 | 12187 | 179 | Bach1,Tnf,Nfkbia,Ifi204,Kcna5,Ifi207,Ifi209,Cdkn1a,Vcam1,Socs3,Apold1,Icam1,Errfi1,Ptgs2,Ccl12,Il6,Pik3r1,Pygm,Angptl4,Ifi211,Bcl3,Nfkb1,Il1a,Sgk1,Thbs1,Cd14 | | 1.021e-04 | -9.19 | positive regulation of cell activation | biological process | GO:0050867 | 240 | 13 | 13711 | 214 | Vcam1,Mmp8,Selp,Runx1,Nfkbiz,Thbs1,Zbtb16,Lgals9,Il4ra,Plek,Il6,Cdkn1a,Ccl2 | | 1.036e-04 | -9.17 | GTP binding | molecular function | GO:0005525 | 309 | 15 | 13516 | 211 | Rasd1,Gem,Tgtp1,Hcar2,Gbp3,Iigp1,Rhou,Mx1,Rhoj,Gbp7,Tubb6,Tgm2,Rhoc,Gbp6,Irgm1 | | 1.044e-04 | -9.17 | ISHIKAWA\_STING\_SIGNALING | MSigDB lists | ISHIKAWA\_STING\_SIGNALING | 7 | 3 | 12187 | 179 | Irf2,Irf9,Irf7 | | 1.054e-04 | -9.16 | GO\_REGULATION\_OF\_TRANSFERASE\_ACTIVITY | MSigDB lists | GO\_REGULATION\_OF\_TRANSFERASE\_ACTIVITY | 753 | 25 | 12187 | 179 | Ccnd2,Map3k8,Pik3r1,Tnfaip3,Ripk1,Il6,Gadd45b,Pik3r5,Iqgap1,Hspa5,Thbs1,Gadd45g,Map3k6,Irak3,Ptpn1,Tnf,Sdc4,Trib1,C5ar1,Fzd4,Cdkn1a,Socs3,Saa1,Hspb1,Errfi1 | | 1.057e-04 | -9.15 | GSE43955\_TH0\_VS\_TGFB\_IL6\_TH17\_ACT\_CD4\_TCELL\_52H\_UP | MSigDB lists | GSE43955\_TH0\_VS\_TGFB\_IL6\_TH17\_ACT\_CD4\_TCELL\_52H\_UP | 157 | 10 | 12187 | 179 | Nfkbiz,Sgk1,Tnf,Nfe2l2,Il1a,Cxcl1,Tnip1,Gem,Plin4,Plaur | | 1.057e-04 | -9.15 | E2F1\_UP.V1\_DN | MSigDB lists | E2F1\_UP.V1\_DN | 157 | 10 | 12187 | 179 | Tgm2,Nfkbiz,Ifit2,Ccnd2,Adamts1,Sgk1,Ccl12,Nfkb1,Angptl4,Ptgs2 | | 1.073e-04 | -9.14 | NAKAYAMA\_SOFT\_TISSUE\_TUMORS\_PCA1\_UP | MSigDB lists | NAKAYAMA\_SOFT\_TISSUE\_TUMORS\_PCA1\_UP | 52 | 6 | 12187 | 179 | Il4ra,Thbs1,Ccl2,Timp1,Ccl12,Cd14 | | 1.073e-04 | -9.14 | TONKS\_TARGETS\_OF\_RUNX1\_RUNX1T1\_FUSION\_GRANULOCYTE\_UP | MSigDB lists | TONKS\_TARGETS\_OF\_RUNX1\_RUNX1T1\_FUSION\_GRANULOCYTE\_UP | 52 | 6 | 12187 | 179 | Ifi211,Arid5b,Ifi209,Hspb1,Ifi204,Ifi207 | | 1.073e-04 | -9.14 | SEITZ\_NEOPLASTIC\_TRANSFORMATION\_BY\_8P\_DELETION\_UP | MSigDB lists | SEITZ\_NEOPLASTIC\_TRANSFORMATION\_BY\_8P\_DELETION\_UP | 52 | 6 | 12187 | 179 | Ifit2,Herc6,Ifi44,Isg15,Ifit3b,Oasl1 | | 1.075e-04 | -9.14 | GO\_VASCULATURE\_DEVELOPMENT | MSigDB lists | GO\_VASCULATURE\_DEVELOPMENT | 369 | 16 | 12187 | 179 | Robo4,Fzd4,Rhoj,Angptl4,Spi1,Ccl12,Rnf213,Ptgs2,Apold1,Clic4,Errfi1,Thbs1,Socs3,Col4a1,Tiparp,Rasip1 | | 1.080e-04 | -9.13 | chemotaxis | biological process | GO:0006935 | 383 | 17 | 13711 | 214 | Cxcl9,Ccl2,Saa1,Ccl7,C3ar1,Il1rn,Lgals9,Retnlg,C5ar1,Cxcl1,Ccl12,Cxcl16,S100a8,Ccl4,S100a9,Vcam1,Il17ra | | 1.087e-04 | -9.13 | GO\_RECEPTOR\_BINDING | MSigDB lists | GO\_RECEPTOR\_BINDING | 991 | 30 | 12187 | 179 | S100a8,Rtp4,Csf3,Cxcl1,Ccl12,Ripk1,Il6,Pik3r1,Ccl4,Ccl2,Il17ra,Cxcl16,Ptpn1,Tap1,Il1a,Thbs1,Tnfsf10,Cflar,A2m,S100a9,Oasl1,Tnf,Icam1,Plaur,Msn,Saa1,Kcna5,Il1rn,Timp1,Vcam1 | | 1.087e-04 | -9.13 | positive regulation of transferase activity | biological process | GO:0051347 | 499 | 20 | 13711 | 214 | Iqgap1,Sdc4,Map3k8,Map3k6,Fzd4,Ptpn1,Pik3r5,Irgm2,Cdkn1a,Tnf,Gadd45b,Gadd45g,Ccnd2,Lgals9,Il1rn,Ncf1,Thbs1,Dtx3l,Trib1,Ripk1 | | 1.099e-04 | -9.12 | PEDERSEN\_METASTASIS\_BY\_ERBB2\_ISOFORM\_1 | MSigDB lists | PEDERSEN\_METASTASIS\_BY\_ERBB2\_ISOFORM\_1 | 33 | 5 | 12187 | 179 | Errfi1,Rasd1,Maff,Gem,Trib1 | | 1.103e-04 | -9.11 | Innate Immune System | REACTOME pathways | R-MMU-168249 | 860 | 29 | 6297 | 105 | Irf2,Cybb,Mmp8,Plaur,Nfkb2,Cdkn1a,C3ar1,Irf7,Nfkb1,Trim56,Ier3,Lcn2,Map3k8,S100a9,Pik3ap1,Pik3r1,Gpr84,S100a8,C5ar1,Tnfaip3,Cd14,Serpina3f,Hp,Ksr1,Csf2rb,Iqgap1,Cxcl1,Ncf1,Nfkbia | | 1.108e-04 | -9.11 | GO\_RESPONSE\_TO\_IONIZING\_RADIATION | MSigDB lists | GO\_RESPONSE\_TO\_IONIZING\_RADIATION | 128 | 9 | 12187 | 179 | Ifi207,Ifi204,Ccl12,Ifi209,Cdkn1a,Socs3,Vcam1,Icam1,Ifi211 | | 1.114e-04 | -9.10 | GSE17721\_POLYIC\_VS\_CPG\_8H\_BMDC\_UP | MSigDB lists | GSE17721\_POLYIC\_VS\_CPG\_8H\_BMDC\_UP | 158 | 10 | 12187 | 179 | Gbp6,C5ar1,Iqgap1,Tnfsf10,Sgk1,Tor3a,Ccl4,Akap12,Gbp4,Mxd4 | | 1.114e-04 | -9.10 | GSE37301\_RAG2\_KO\_VS\_RAG2\_AND\_ETS1\_KO\_NK\_CELL\_UP | MSigDB lists | GSE37301\_RAG2\_KO\_VS\_RAG2\_AND\_ETS1\_KO\_NK\_CELL\_UP | 158 | 10 | 12187 | 179 | Saa1,A2m,Cd14,Osmr,Xdh,Sntb2,Tiparp,Gadd45g,Ripk1,Lcn2 | | 1.122e-04 | -9.09 | GO\_ADAPTIVE\_IMMUNE\_RESPONSE\_BASED\_ON\_SOMATIC\_RECOMBINATION\_OF\_IMMUNE\_RECEPTORS\_BUILT\_FROM\_IMMUNOGLOBULIN\_SUPERFAMILY\_DOMAINS | MSigDB lists | GO\_ADAPTIVE\_IMMUNE\_RESPONSE\_BASED\_ON\_SOMATIC\_RECOMBINATION\_OF\_IMMUNE\_RECEPTORS\_BUILT\_FROM\_IMMUNOGLOBULIN\_SUPERFAMILY\_DOMAINS | 75 | 7 | 12187 | 179 | Nfkb2,Icam1,Irf7,Bcl3,C3ar1,Il6,Il4ra | | 1.122e-04 | -9.09 | GO\_REGULATION\_OF\_TRANSCRIPTION\_FACTOR\_IMPORT\_INTO\_NUCLEUS | MSigDB lists | GO\_REGULATION\_OF\_TRANSCRIPTION\_FACTOR\_IMPORT\_INTO\_NUCLEUS | 75 | 7 | 12187 | 179 | Csf3,Nfkbie,Ptgs2,Bcl3,Tnf,Pik3r1,Nfkbia | | 1.128e-04 | -9.09 | nucleoside-triphosphatase activity | molecular function | GO:0017111 | 665 | 24 | 13516 | 211 | Hspa5,Tgtp2,Gm4841,Rasd1,F830016B08Rik,Gem,Tgtp1,Tap1,Iigp1,Gbp3,Rnf213,Mx2,Rhou,Rhoj,Mx1,Irgm2,Gbp7,Tubb6,Tor3a,Ifi47,Gm5431,Irgm1,Rhoc,Gbp6 | | 1.171e-04 | -9.05 | positive regulation of macrophage chemotaxis | biological process | GO:0010759 | 17 | 4 | 13711 | 214 | Ccl2,C5ar1,Thbs1,C3ar1 | | 1.171e-04 | -9.05 | positive regulation of tumor necrosis factor biosynthetic process | biological process | GO:0042535 | 17 | 4 | 13711 | 214 | Hspb1,Thbs1,Akap12,Cybb | | 1.171e-04 | -9.05 | NAD+ ADP-ribosyltransferase activity | molecular function | GO:0003950 | 17 | 4 | 13516 | 211 | Parp9,Parp12,Tiparp,Parp14 | | 1.172e-04 | -9.05 | negative regulation of cell population proliferation | biological process | GO:0008285 | 542 | 21 | 13711 | 214 | Pik3r1,Lgals9,Ifitm3,Thbs1,Trib1,Zbtb16,Ripk1,Il6,Xdh,Slfn3,Cdkn1a,Tnf,Tnfaip3,Sox11,Il1a,Ptgs2,Runx1,Slfn2,Ccl12,Ptges,Sdc4 | | 1.172e-04 | -9.05 | FERRARI\_RESPONSE\_TO\_FENRETINIDE\_UP | MSigDB lists | FERRARI\_RESPONSE\_TO\_FENRETINIDE\_UP | 18 | 4 | 12187 | 179 | Cxcl1,Ptgs2,Atf3,Sele | | 1.172e-04 | -9.05 | WANG\_IMMORTALIZED\_BY\_HOXA9\_AND\_MEIS1\_UP | MSigDB lists | WANG\_IMMORTALIZED\_BY\_HOXA9\_AND\_MEIS1\_UP | 18 | 4 | 12187 | 179 | Igsf6,Rhoj,Ccnd2,Thbs1 | | 1.174e-04 | -9.05 | GSE17721\_POLYIC\_VS\_GARDIQUIMOD\_2H\_BMDC\_DN | MSigDB lists | GSE17721\_POLYIC\_VS\_GARDIQUIMOD\_2H\_BMDC\_DN | 159 | 10 | 12187 | 179 | Gpr84,Casp4,Socs3,Nfe2l2,Saa1,Csf3,C5ar1,Irf9,Icam1,Tnfaip8 | | 1.174e-04 | -9.05 | GSE31622\_WT\_VS\_KLF3\_KO\_BCELL\_DN | MSigDB lists | GSE31622\_WT\_VS\_KLF3\_KO\_BCELL\_DN | 159 | 10 | 12187 | 179 | Sele,Nfkb2,Ptpn1,Ifitm2,Pik3r5,Stx11,Znfx1,Ccl4,Socs3,Trib1 | | 1.174e-04 | -9.05 | GSE39820\_CTRL\_VS\_IL1B\_IL6\_CD4\_TCELL\_DN | MSigDB lists | GSE39820\_CTRL\_VS\_IL1B\_IL6\_CD4\_TCELL\_DN | 159 | 10 | 12187 | 179 | Gem,Plek,Zfp36,Hspa5,Socs3,Timp1,Il1rn,Nfe2l2,Tnfaip3,AA467197 | | 1.174e-04 | -9.05 | GSE15767\_MED\_VS\_SCS\_MAC\_LN\_UP | MSigDB lists | GSE15767\_MED\_VS\_SCS\_MAC\_LN\_UP | 159 | 10 | 12187 | 179 | Cybb,Thbs1,Msr1,Csf2rb,Rbm47,P2ry6,Ncf1,C5ar1,Ifitm3,Ifitm2 | | 1.174e-04 | -9.05 | GSE22886\_NAIVE\_CD4\_TCELL\_VS\_MONOCYTE\_DN | MSigDB lists | GSE22886\_NAIVE\_CD4\_TCELL\_VS\_MONOCYTE\_DN | 159 | 10 | 12187 | 179 | Spi1,Cebpd,Cybb,Cd14,S100a9,C5ar1,Csf2rb,S100a8,Igsf6,Irak3 | | 1.174e-04 | -9.05 | GSE5589\_IL6\_KO\_VS\_IL10\_KO\_LPS\_AND\_IL10\_STIM\_MACROPHAGE\_45MIN\_UP | MSigDB lists | GSE5589\_IL6\_KO\_VS\_IL10\_KO\_LPS\_AND\_IL10\_STIM\_MACROPHAGE\_45MIN\_UP | 159 | 10 | 12187 | 179 | Ccl4,Cdkn1a,Tnfaip3,Mmp8,Ncf1,Cd14,Nfkbie,Ifitm3,Rgs16,Plaur | | 1.176e-04 | -9.05 | HTLV-I infection | KEGG pathways | ko05166 | 218 | 14 | 5248 | 107 | Spi1,Zfp36,Nfkbia,Pik3r1,Ccnd2,Nfkb1,Il6,Vcam1,Icam1,Tnf,Cdkn1a,Atf3,Nfkb2,Fzd4 | | 1.176e-04 | -9.05 | HTLV-I infection | KEGG pathways | mmu05166 | 218 | 14 | 5248 | 107 | Zfp36,Spi1,Nfkbia,Pik3r1,Ccnd2,Nfkb1,Il6,Vcam1,Tnf,Icam1,Atf3,Cdkn1a,Nfkb2,Fzd4 | | 1.181e-04 | -9.04 | IRF\_Q6 | MSigDB lists | I
[truncated: 759,813 more chars]
