# Supplementary material for: Repeated LPS induces training and tolerance of microglial responses across brain regions
Source: J Neuroinflammation. 2024 Sep 20;21:233. doi: 10.1186/s12974-024-03198-1 (PMC11414187; doi:10.1186/s12974-024-03198-1)
Supplement: Supplementary file 7 — Supplementary Material 7. File S1: Homer software output for transcription factor motif analysis of 2xLPS-sensitive cluster gene promoters. Related to Figures 4A and C. [file 12974_2024_3198_MOESM7_ESM.zip › 2xLPS_cluster_genes_output/homerResults/motif18.info.html]

Motif 18

## Information for 9-TGGACRCTTT (Motif 18)

C
G
A
T
A
C
T
G
A
C
T
G
C
G
T
A
A
G
T
C
C
T
G
A
A
G
T
C
C
G
A
T
A
G
C
T
A
C
G
T
  
Reverse Opposite:  
