# Supplementary material for: Repeated LPS induces training and tolerance of microglial responses across brain regions
Source: J Neuroinflammation. 2024 Sep 20;21:233. doi: 10.1186/s12974-024-03198-1 (PMC11414187; doi:10.1186/s12974-024-03198-1)
Supplement: Supplementary file 8 — Supplementary Material 8. File S2: Homer software output for transcription factor motif analysis of 4xLPS-sensitive cluster gene promoters. [file 12974_2024_3198_MOESM8_ESM.zip › 4xLPS_cluster_genes_output/homerResults/motif15.similar.html]

motif15

## Information for motif15

C
G
T
A
T
A
G
C
G
A
C
T
C
G
T
A
A
T
C
G
A
T
G
C
G
A
T
C
G
C
A
T
C
A
T
G
A
C
T
G
  
Reverse Opposite:  

T
G
A
C
G
T
A
C
C
G
T
A
C
T
A
G
T
A
C
G
T
A
G
C
G
C
A
T
C
T
G
A
A
T
C
G
G
C
A
T
  

|  |  |
| --- | --- |
| p-value: | 1e-8 |
| log p-value: | -1.858e+01 |
| Information Content per bp: | 1.549 |
| Number of Target Sequences with motif | 21.0 |
| Percentage of Target Sequences with motif | 18.75% |
| Number of Background Sequences with motif | 545.9 |
| Percentage of Background Sequences with motif | 4.14% |
| Average Position of motif in Targets | 648.1 +/- 327.2bp |
| Average Position of motif in Background | 612.0 +/- 404.5bp |
| Strand Bias (log2 ratio + to - strand density) | -0.1 |
| Multiplicity (# of sites on avg that occur together) | 1.10 |
| Motif File: | file (matrix) reverse opposite |

### Similar de novo motifs found

|  |  |  |  |  |  |  |  |
| --- | --- | --- | --- | --- | --- | --- | --- |
| Rank | Match Score | Redundant Motif | P-value | log P-value | % of Targets | % of Background | Motif file |
