# Supplementary material for: Repeated LPS induces training and tolerance of microglial responses across brain regions
Source: J Neuroinflammation. 2024 Sep 20;21:233. doi: 10.1186/s12974-024-03198-1 (PMC11414187; doi:10.1186/s12974-024-03198-1)
Supplement: Supplementary file 8 — Supplementary Material 8. File S2: Homer software output for transcription factor motif analysis of 4xLPS-sensitive cluster gene promoters. [file 12974_2024_3198_MOESM8_ESM.zip › 4xLPS_cluster_genes_output/homerResults/motif3.info.html]

Motif 3

## Information for 1-GCAGATATAG (Motif 3)

C
T
A
G
A
G
T
C
C
G
T
A
A
C
T
G
T
C
G
A
A
C
G
T
C
G
T
A
A
C
G
T
C
T
G
A
A
C
T
G
  
Reverse Opposite:  
