# Supplementary material for: Repeated LPS induces training and tolerance of microglial responses across brain regions
Source: J Neuroinflammation. 2024 Sep 20;21:233. doi: 10.1186/s12974-024-03198-1 (PMC11414187; doi:10.1186/s12974-024-03198-1)
Supplement: Supplementary file 9 — Supplementary Material 9. File S3: Homer software output for transcription factor motif analysis of LPS-decreased cluster gene promoters. [file 12974_2024_3198_MOESM9_ESM.zip › LPSDownregulated_cluster_genes_output/homerResults/motif19.info.html]

Motif 19

## Information for 12-CTCTGGCAAGAA (Motif 19)

A
T
G
C
A
C
G
T
A
T
G
C
C
A
G
T
A
C
T
G
C
A
T
G
T
G
A
C
C
T
G
A
C
T
G
A
T
A
C
G
T
C
G
A
C
G
T
A
  
Reverse Opposite:  
